# Enantiodivergent Carbon–Phosphorus Bond Formation via Enzymatic Carbene Insertion

**DOI:** 10.1021/jacs.6c10375

**Published:** 2026-08-06

**Authors:** Hayden M. Carder, Ethan N. Lin, Daniel Roth, Frances H. Arnold

**Affiliations:** Division of Chemistry and Chemical Engineering, 6469 California Institute of Technology, Pasadena, California 91125, United States

## Abstract

Phosphorus is one of the six elements of life, yet enzymatic carbon–phosphorus (C–P) bond formation is exceedingly rare in nature. This gap in the biosynthetic toolkit is particularly consequential, given the prominence of stereogenic-at-phosphorus P­(V) scaffolds in pharmaceuticals and agrochemicals, where the absolute configuration at phosphorus often directly governs biological activity. Despite significant advances in the asymmetric synthesis of P-stereogenic compounds, a general enzymatic platform based on direct C–P bond formation has not been realized. Here, we describe the discovery and directed evolution of Aeropyrum pernix protoglobin (*Ape*Pgb) variants that catalyze carbene insertion into the P–H bonds of secondary phosphine oxides and H–phosphinates. Mechanistic investigations reveal that carbene P–H insertion proceeds through a stereoretentive kinetic resolution. Directed evolution of a single protein scaffold produced an enantiodivergent enzyme platform that selectively delivers either P-configuration with high enantioselectivity across a broad substrate scope. The evolution campaign further revealed latent activity for nitrene P–H insertion, opening a path toward P-stereogenic phosphinamides and phosphonamidates through future evolution.

## Graphical abstract

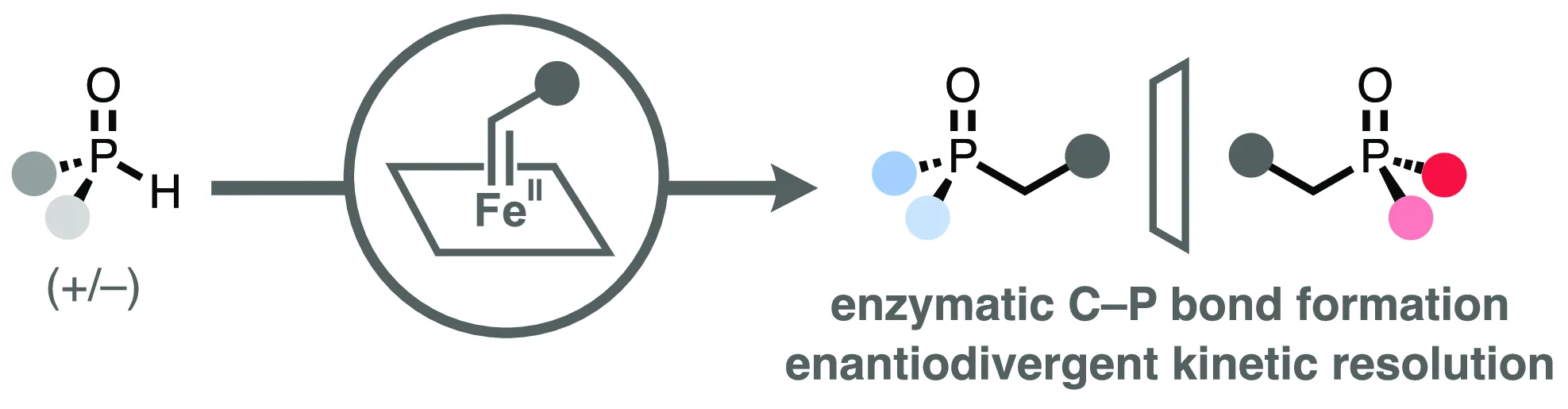

## Introduction

Phosphorus is indispensable to life. As a constituent of nucleic acids, phospholipids, ATP, and signaling intermediates, phosphorus chemistry underlies virtually every aspect of cellular function and is built almost exclusively on phosphorus­(V)–oxygen (P–O) bonds.^1,2^ Enzymatic C–P bond formation is vanishingly rare, with a single dedicated biosynthetic pathway initiated by phosphoenolpyruvate mutase (PEP mutase) responsible for all approximately 200 known metabolites (Figure 1A).^1,3^ Nevertheless, natural phosphonates and phosphinates—including potent enzyme inhibitors, membrane lipid components, and the herbicide glufosinate—demonstrate the biological potency of the C–P bond class.^4,5^ Expanding the enzymatic toolkit for C–P bond formation would provide new routes to valuable organophosphorus scaffolds that complement existing synthetic approaches.

**Figure 1:**
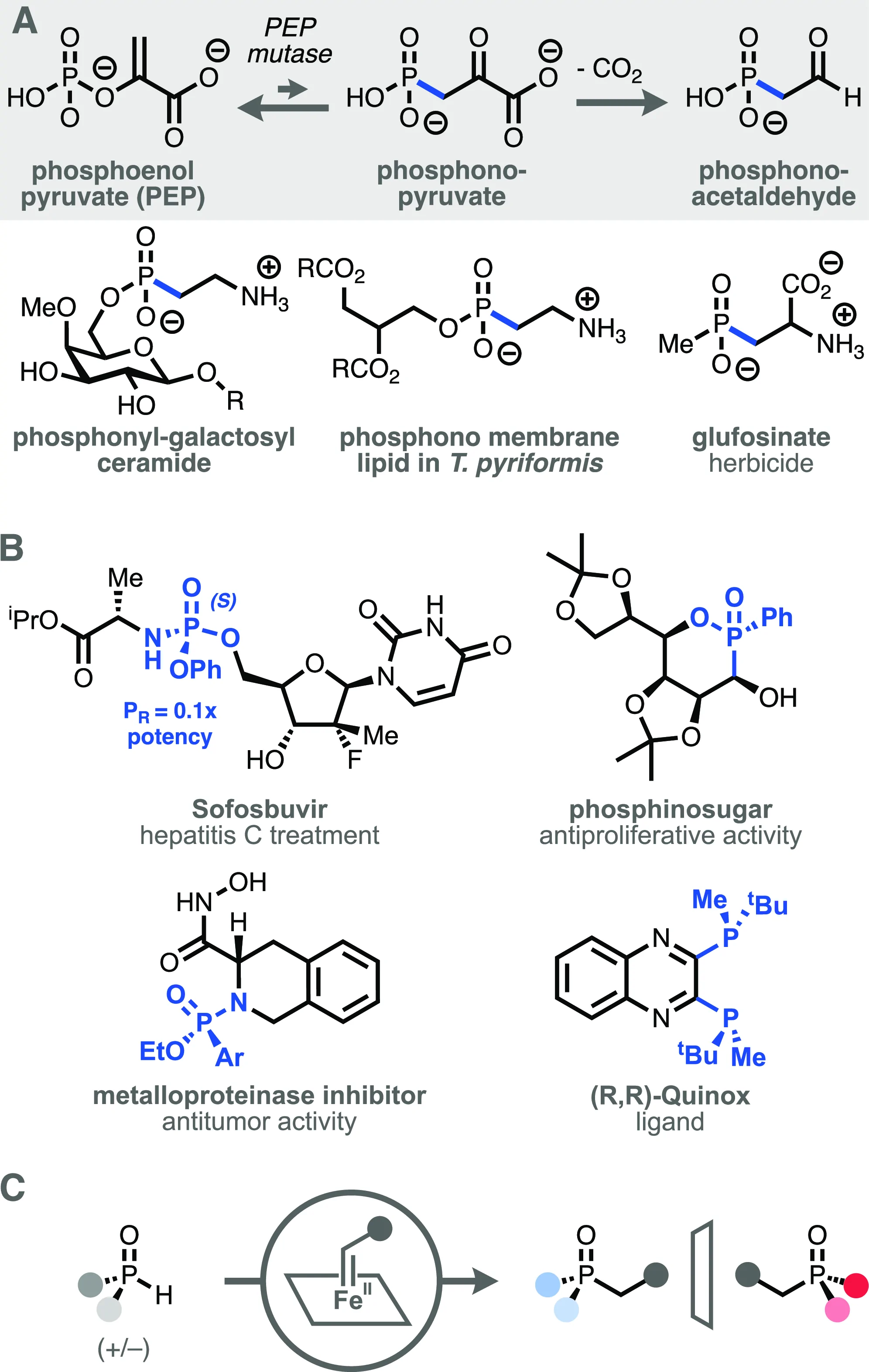
(A) Enzymatic C–P bond formation is rare in nature, confined to a dedicated biosynthetic pathway responsible for approximately 200 known metabolites. (B) Representative stereogenic-at-phosphorus compounds are utilized in medicine and catalysis. (C) Engineered *Ape*Pgb variants catalyze enantioselective carbene insertion into P–H bonds of secondary phosphine oxides and H–phosphinates.

In contrast to their limited occurrence in nature, stereogenic-at-phosphorus scaffolds—phosphine oxides, phosphinates, phosphonamidates, and related structures—occupy a prominent place in medicine, agrochemistry, and asymmetric catalysis. The absolute configuration at phosphorus directly determines the activation of the prodrug sofosbuvir,^6^ the antiproliferative selectivity of phosphinosugars,^7^ the activity of phosphinamide metalloproteinase and carbonic anhydrase inhibitors,^8–10^ and the enantioselectivity of phosphine ligands such as Quinox and DuanPhos (Figure 1B).^11^ Existing synthetic methods for P-stereogenic compound synthesis—including chiral auxiliary approaches,^12–14^ asymmetric metal catalysis,^15–17^ and hydrogen-bond donor catalysis^18–20^ —have enabled efficient access to diverse P-stereogenic scaffolds.

In contrast, enzymatic approaches have been constrained, reflecting biology’s near-exclusive reliance on P–O bond chemistry. Existing biocatalytic methods operate through selective P–O bond cleavage by phosphotriesterases^21^ or desymmetrization and resolution by lipases, the latter of which operate on substituents peripheral to the phosphorus stereocenter.^22,23^ The first enzymatic approach to address this limitation through direct C–P bond formation was recently reported by Mou and coworkers, who demonstrated enantioconvergent photoenzymatic hydrophosphinylation of activated alkenes using an engineered imine reductase that operates through a P-centered radical mechanism.^24^ Continued advancement of the synthetic toolkit for stereogenic-at-phosphorus compounds will require more enzymatic methods capable of direct and enantioselective C–P bond formation across complementary reactivity manifolds and diverse substrate classes.

The Arnold lab and others have developed heme proteins as versatile platforms for new-to-nature chemistry, establishing a foundation for addressing the challenge of enantioselective C–P bond formation. Engineered hemoproteins can generate reactive iron carbenoid and nitrenoid intermediates from carbene and nitrene precursors, respectively, enabling a diverse array of reactions not found in biology.^25,26^ These include carbene insertion into silicon–hydrogen bonds (Si–H),^27^ boron–hydrogen bonds (B–H),^28^ and nitrogen–hydrogen bonds (N–H),^29^ and, via nitrene transfer, primary amination of carbon–hydrogen bonds (C–H).^30–32^ Small-molecule catalysis has independently established that carbene and nitrene equivalents can directly functionalize P–H bonds,^33–36^ yet enantiocontrol at phosphorus has not been achieved in any of these systems. The fundamental challenge is molecular recognition: the short-lived intermediates in small-molecule-catalyzed reactions lack the organized contacts needed to differentiate the four substituents at a P­(V) stereocenter. Heme proteins offer a distinct solution: the chiral active site enforces enantiodiscrimination through a precise network of noncovalent interactions within a preorganized environment. We therefore hypothesized that a heme enzyme capable of X–H insertion could be engineered for enantioselective P–H insertion and report the discovery and directed evolution of Aeropyrum pernix protoglobin (*Ape*Pgb) variants that catalyze carbene insertion into the P–H bonds of secondary phosphine oxides and H–phosphinates (Figure 1C).

## Results and Discussion

We began by screening a broad panel of thermostable protoglobin variants for carbene P–H insertion activity. The panel, comprising approximately 300 carbene and nitrene transferases—including enzymes evolved for C–H insertion, cyclopropanation, ring expansion, and C–H amination—was expressed in *Escherichia coli* whole cells and screened against diphenylphosphine oxide (Ph_2_P­(O)­H) with ethyl diazoacetate (EDA) (see SI, Section 4.1). Initial hits were validated across three substrates chosen to represent the steric and electronic diversity of the intended scope: Ph_2_P­(O)­H, PhMeP­(O)H (**1a**), and Ph­(OEt)­P­(O)H (**2a**) (Figure 2A). A single variant, designated **G0**, showed a balanced activity profile across all three substrates, providing an optimal starting point for a directed evolution campaign aimed at broad substrate generality rather than narrow optimization on a single P–H bond type. Enzymatic activity was confirmed by control reactions (see SI, Section 4.2).

**Figure 2:**
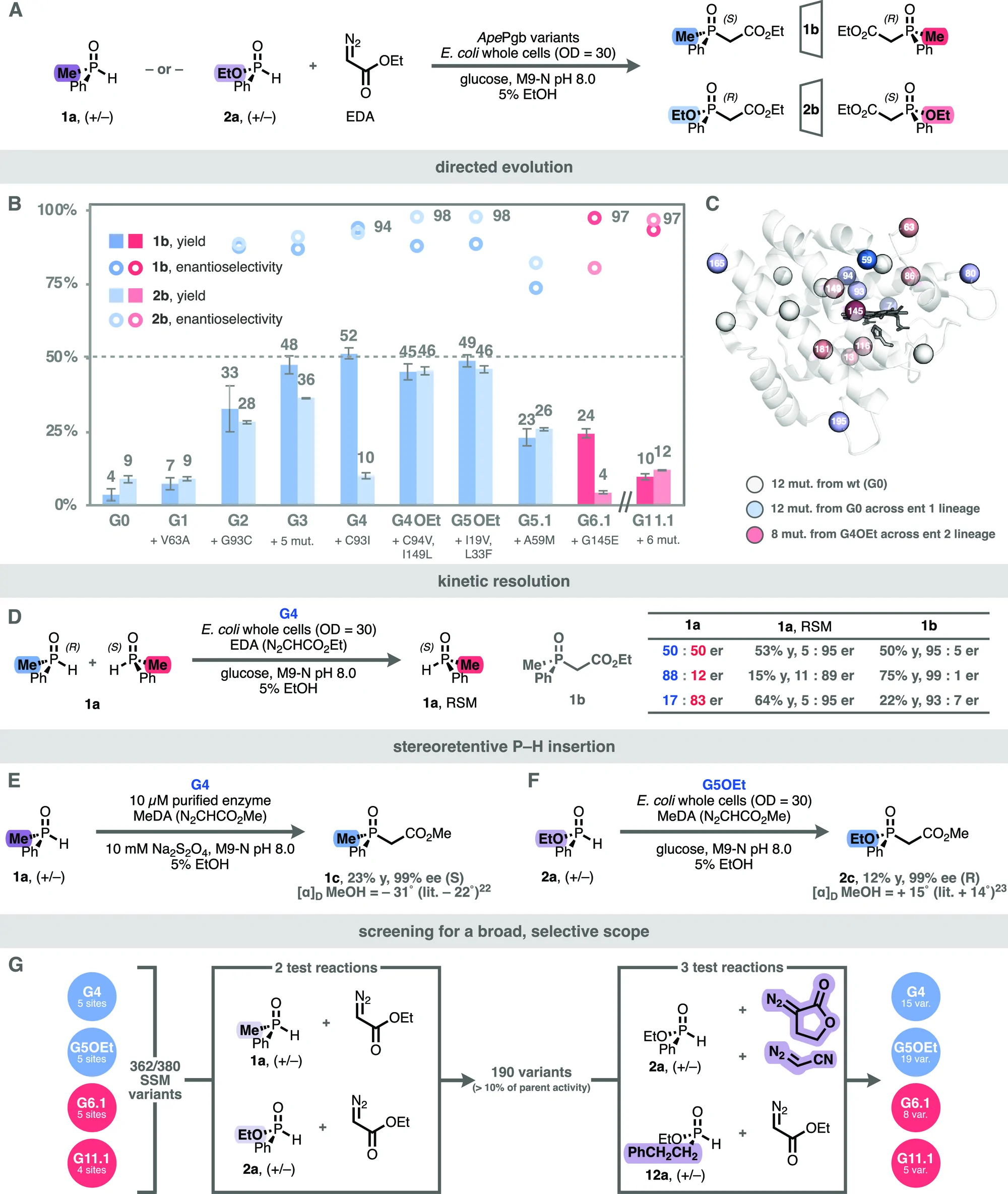
(A) Model reactions were conducted to test biocatalytic activity. (B) Divergent evolution of **G0** and the corresponding yield and enantioselectivity. The mutations acquired relative to the previous variant are shown below the bar graph. Data represent the average ± standard deviation (*n* = 3–6). The experiments were performed at analytical scale using whole-cell *E. coli* (Optical Density, OD_600_ = 30) expressing the *Ape*Pgb variants with 5 mM substrate and 3 mM EDA in M9-N buffer (pH = 8.0) with 5 v/v% EtOH and 25 mM glucose under anaerobic conditions. Yields were quantified by LC-MS based on calibration curves of the corresponding reference products. Enantioselectivities were measured by HPLC on a chiral phase. (C) A structural model based on the crystal structure of an *Ape*Pgb mutant (8EUM), with mutated residues from *wt-Ape*Pgb highlighted in white, mutations toward **P_S_-1b** and **P_R_-2b** highlighted in blue, and mutations toward **P_R_-1b** and **P_S_-2b** highlighted in red. (D) Carbene transfer reaction catalyzed by **G4** using enantioenriched **1a** and EDA. (E) Carbene transfer reaction catalyzed by **G4** using **1a** and MeDA. (F) Carbene transfer reaction catalyzed by **G5OEt** using **2a** and MeDA. (G) Generation of a tailored enzyme library enabling a broad, selective substrate scope. See the Supporting Information for more details.

Notably, most variants performed at or below the level of the hemin-catalyzed background reaction—including wild-type *Ape*Pgb and variants previously evolved for main-group X–H insertion, such as Si–H, B–H, and N–H insertion enzymes (see SI, Section 4). Initial activity emerged from variants evolved for an unrelated transformation, demonstrating that broader exploration of the hemoprotein distribution through the incorporation of non-natural mutations can unlock reactivities inaccessible to existing variant collections.

We selected PhMeP­(O)H (**1a**) with EDA as the primary reaction for directed evolution (see SI, Section 5 for full evolution details). Hits from each round of evolution were validated against both PhMeP­(O)H (**1a**) and Ph­(OEt)­P­(O)­H (**2a**), allowing for selection of variants with an optimal activity profile. Early rounds of evolution (**G1**–**G3**) focused on improving yield utilizing error-prone PCR (epPCR), as the low initial conversion of **G0** precluded reliable determination of enantioselectivity (Figure 2B). Among the mutations identified by epPCR, a substitution at residue 93 – a site previously recognized as critical for selectivity in *Ape*Pgb-catalyzed reactions – emerged as the key activating mutation. The resulting variant, **G3**, delivered PhMeP­(O)–CH_2_CO_2_Et (**P**_**S**_ **-1b**) in 48% yield and 87% ee. At this point, the evolution lineage branched to independently fine-tune activity and selectivity for each substrate class through targeted site-saturation mutagenesis (SSM), providing final variants **G4** and **G5OEt** for PhMeP­(O)–CH_2_CO_2_Et (**P**_**S**_ **-1b**) and Ph­(OEt)­P­(O)–CH_2_CO_2_Et (**P**_**R**_ **-2b**), respectively. Key mutations accumulated across positions in the B/E helix tunnel and distal cavity of *Ape*Pgb, consistent with reshaping substrate access and positioning within the active site (Figure 2C).

All initial hits from screening afforded the major product enantiomer in good enantioselectivity, and no variant identified during the **G0**–**G5OEt** evolution campaign significantly eroded or inverted enantioselectivity. This uniformity suggested that the hemoprotein active site strongly favors a single binding orientation for the P–H substrate, and that accessing the opposite enantiomer would require discrete remodeling of the active-site environment rather than incremental reoptimization of the existing lineage. To obtain the opposite P-configuration, we selected Ph­(OEt)­P­(O)H (**2a**) as the model reaction for the inversion campaign, as the shorter chiral-HPLC method reduced the screening burden. Beginning from **G4OEt**, SSM at active-site positions identified during the primary campaign were used to probe for selectivity inversion. Of the sites evaluated, only the introduction of A59M significantly diminished enantioselectivity, suggesting disruption of substrate positioning within the active site and providing a starting point from which further evolution could access the inverted selectivity landscape. Subsequent SSM at a neighboring residue identified G145E, delivering the product in the opposite enantiopode with excellent enantioselectivity. In this case, variant **G4OEt**-G145E had 14-fold lower activity than **G4OEt**-A59M/G145E (**G6.1**), establishing A59M as an enabling mutation that facilitated both the discovery of G145E and access to the inverted selectivity landscape.

Additional rounds of evolution from this G145E-containing variant (**G6.1**) aimed to improve activity on Ph­(OEt)­P­(O)H (**2a**), but yield improvements were limited despite extensive screening. This stagnation suggests that the A59M/G145E combination has positioned the lineage in a region of sequence space where further improvement through single-site mutagenesis is difficult. Nonetheless, the enantiodivergent platform provides access to both P-configurations across secondary phosphine oxides and H–phosphinates from a single protein scaffold, with **G4**/**G5OEt** delivering one enantiomer and **G6.1**/**G11.1** delivering the other, each with up to 98% ee.

The evolution data provided the first mechanistic insight into the basis of enantioselectivity. During the evolution campaign, yields consistently plateaued near 50% with concomitant enrichment of the residual starting material, suggesting that the enzyme was operating through a kinetic resolution. This hypothesis was confirmed by subjecting enantioenriched PhMeP­(O)H (**1a**) to **G4**, which revealed preferential consumption of **P**_**R**_ **-1a** with corresponding accumulation of **P**_**S**_ **-1a** in the recovered starting material (Figure 2D). To determine the absolute configuration of the product and whether the configuration at phosphorus is retained or inverted during the carbene insertion event, preparative-scale reactions of PhMeP­(O)­H (**1a**) and Ph­(OEt)­P­(O)H (**2a**) with MeDA were carried out and the products isolated for full characterization (Figure 2E and 2F). The absolute stereochemistry of the products was confirmed by comparison of the measured optical rotations with literature-reported values for compounds of known absolute stereochemistry. Together, these experiments establish that stereospecific carbene insertion proceeds with retention of configuration at phosphorus.

To overcome the inherent selectivity-activity relationship developed throughout enzyme evolution, a targeted library was designed from the four optimized variants – **G4**, **G5OEt**, **G6.1**, and **G11.1** – by subjecting them to site saturation mutagenesis (SSM) at active-site positions identified as most consequential for yield and enantioselectivity (Figure 2G). The resulting library was first filtered using the two model reactions (**1a** and **2a** with EDA), retaining approximately 190 variants showing greater than 10% of parent activity. The filtered variants were then evaluated against three additional substrate–reagent combinations representing the chemical diversity of the intended scope: Ph­(OEt)­P­(O)H (**2a**) with diazoacetonitrile (DAN), Ph­(OEt)­P­(O)­H (**2a**) with α-butyrolactone diazo compound (LAD), and PhCH_2_CH_2_(OEt)­P­(O)H (1**2a**) with EDA. Variants that performed better across any combination were consolidated into a master compilation plate comprising 46 unique variants drawn from all four optimized variants. This collection was then screened against the substrate scope, providing a matched set of optimized catalysts for each substrate–reagent combination and revealing how discrete active-site mutations modulate substrate recognition across structurally diverse P–H substrates (see SI, Section 5.3).^36^

We first evaluated a series of phenyl-alkyl secondary phosphine oxides to probe steric tolerance at phosphorus (Figure 3). Substrates bearing methyl, benzyl, isopropyl, and cyclohexyl substituents were readily functionalized with good to excellent yield and enantioselectivity for both P_R_ and P_S_ product configurations, demonstrating that the evolved variants accommodate a meaningful range of steric bulk at phosphorus without significant erosion of selectivity. Screening of the compilation plate identified variants bearing single active-site mutations that further improved performance on two sterically demanding substrates: a mutation at site 93 improved enantioselectivity for PhBnP­(O)–CH_2_CO_2_Et (**P**_**S**_ **-3b**) and PhCyP­(O)–CH_2_CO_2_Et (**P**_**R**_ **-5b**) over the optimized lineage variants, while a mutation at site 149 in **G11.1** improved the yield of PhCyP­(O)–CH_2_CO_2_Et (**P**_**S**_ **-5b**) from 19% to 45% while maintaining enantioselectivity (99% ee). These improvements illustrate the value of the compilation plate strategy, in which diversity generated during directed evolution can be selectively harnessed to address the specific requirements of individual substrates.

**Figure 3:**
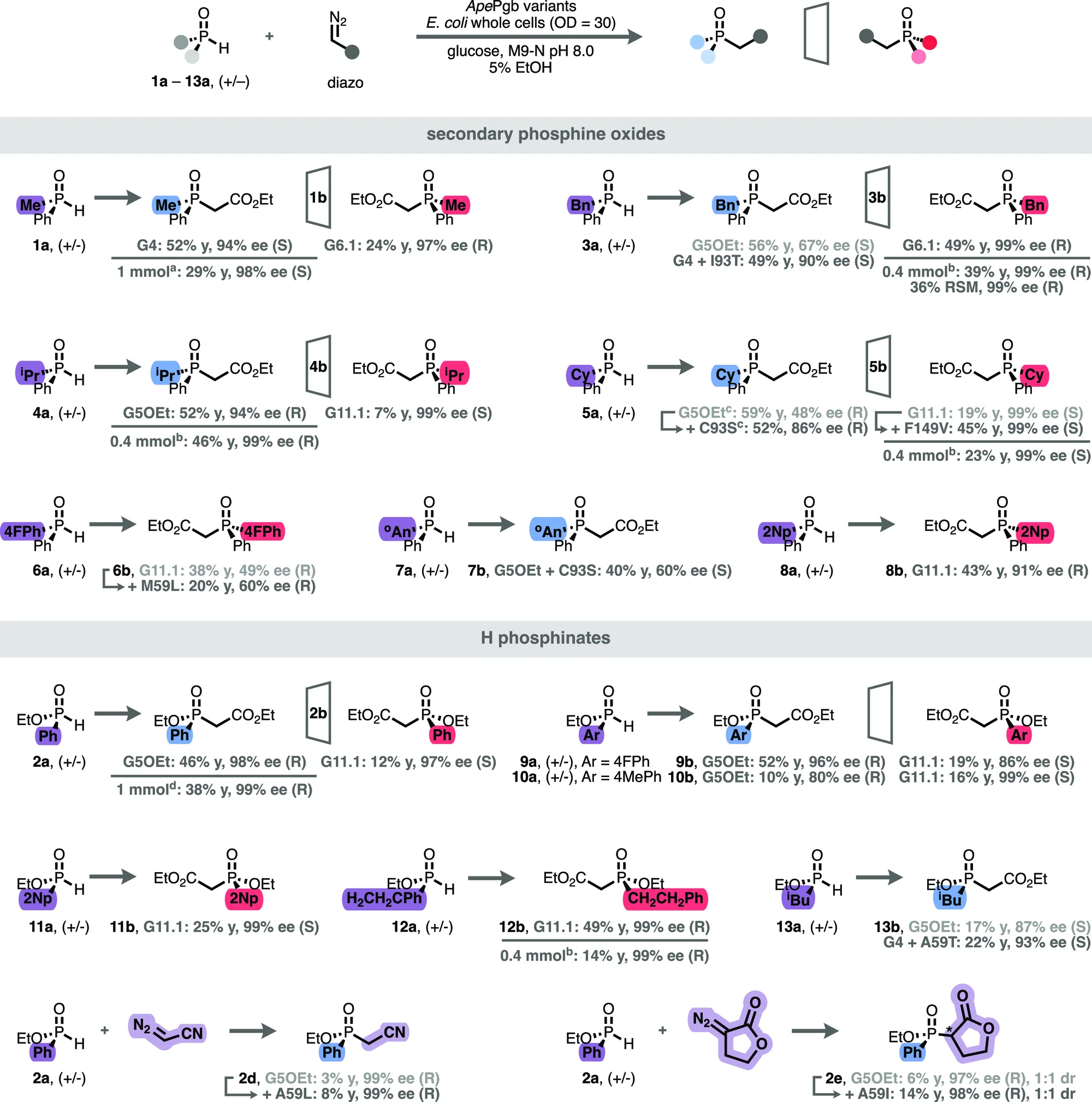
Substrate scope evaluated using selected *Ape*Pgb variants. Products from variants of G4 and G4OEt are highlighted in blue. Products from variants of G6.1 and G11.1 are highlighted in red. Data represent the average (*n* = 3). The experiments were performed at analytical scale using whole-cell *E. coli* (Optical Density, OD_600_ = 30) that expressed the *Ape*Pgb variants with 5 mM substrate and 3 mM carbenoid precursor in M9-N buffer (pH = 8.0) with 5 v/v% EtOH and 25 mM glucose under anaerobic conditions. Yields were quantified by LC-MS based on calibration curves of the corresponding reference products. Enantioselectivities were measured by HPLC on a chiral phase. ^a^Experiment performed on a 1 mmol scale with 25 mM [P] and 15 mM EDA, isolated yield reported. ^b^Experiment performed on a 0.4 mmol scale with 20 mM [P] and 12 mM EDA, isolated yield reported. ^c^Experiment performed with a shortened 1 h reaction time and 2.75 mM EDA. ^d^Experiment performed on a 1 mmol scale with 25 mM [P] and 15 mM EDA, ^1^H NMR yield reported. See the Supporting Information for more details.

Reactions within the secondary phosphine oxide scope were also demonstrated at a semipreparative scale with higher substrate loading (20–25 mM [P]). Selectivity was maintained or improved relative to analytical-scale conditions, though yields were somewhat diminished. A particularly noteworthy outcome emerged from the reaction of PhBnP­(O)­H (**3a**) with **G6.1**, in which both the carbene insertion product and the enantioenriched recovered starting material could be isolated in moderate yield with perfect enantioselectivity for each. This result demonstrates that the kinetic resolution can be exploited synthetically to deliver two enantioenriched P-stereogenic compounds, the product and the unreacted starting material, from a single reaction.

Phenyl-aryl secondary phosphine oxides (**6a**–**8a**) were well tolerated, though with poor enantioselectivity, a result that is nonetheless remarkable given that no selection pressure for diaryl-substituted substrates was applied following the identification of **G0**. The fact that variants **G11.1** and **G11.1**-M59L distinguish between phenyl and 4-fluorophenyl substituents at phosphorus, a subtle electronic difference with minimal steric component, suggests that the aryl group is embedded within the active site in a manner sensitive to its electronic character. Ph­(2Np)­P­(O)­H (**8a**) was a suitable substrate for **G11.1**, providing Ph­(2Np)­P­(O)–CH_2_CO_2_Et (**P**_**R**_ **-8b**) in 43% yield and 91% ee, further extending the scope to more sterically and electronically distinct aryl substituents.

We next evaluated the H–phosphinate substrate class, beginning with electronic and steric variation of the aryl substituent of Ph­(OEt)­P­(O)­H. Phenyl (**2a**), *para*-fluorophenyl (**9a**), *para*-tolyl (**10a**), and 2-naphthyl (**11a**) substituted H–phosphinates were all well-tolerated with good yield and enantioselectivity, demonstrating that the active site accommodates meaningful electronic and steric variation at the aryl group without significant erosion of enantioselectivity.

The scope extended to aliphatic H–phosphinates, a substrate class underrepresented in existing P-stereogenic synthesis methods. PhCH_2_CH_2_(OEt)­P­(O)H (**12a**) and ^*i*^Bu­(OEt)­P­(O)H (**13a**) were both accepted by variants from the compilation plate. The observed activity of **13a** (22% yield, 93% ee) is notable, given the lack of an aryl substituent present in both model substrates used for the evolution campaign. This result suggests that the active site can engage aliphatic P–H substrates through contacts beyond those established during lineage evolution, and we anticipate that targeted engineering will further expand the scope to additional aliphatic substrate classes.

Finally, alternative carbene precursors were evaluated on the H–phosphinate scaffold. Diazoacetonitrile (DAN) proved to be a competent precursor, extending the functional group diversity accessible at the newly formed C–P bond. The α-butyrolactone diazo compound (LAD) was also evaluated as a means of establishing two stereocenters in a single elementary step. While the product (**2e**) was obtained with excellent enantioselectivity (98% ee), the α-carbon stereocenter was found to be configurationally labile under the reaction conditions, resulting in a 1:1 diastereomeric mixture.

Inspired by previous work in our laboratory demonstrating that carbene transferases can be evolved into nitrene transferases,^30^ we hypothesized that variants already adapted to engage phosphorus scaffolds through carbene transfer might harbor latent nitrene P–H insertion activity. Access to such reactivity would extend the platform to direct P–N bond formation, providing P-stereogenic phosphinamides and phosphonamidates through a distinct reactive intermediate and bond-forming pathway. We evaluated *O*-pivaloylhydroxylamine triflate (PONT) as the nitrene precursor with Ph­(OEt)­P­(O)H (**2a**)­(Figure 4A). Whereas **G0** and other protoglobin variants from our library showed only trace activity, **G4** exhibited a 20-fold improvement, providing the phosphonamidate Ph­(OEt)­P­(O)–NH_2_ (**P**_**S**_ **-2f**) in 38% yield and 26% ee (Figure 4B). This result demonstrates that evolution for carbene P–H insertion enriches nitrene P–H insertion activity, consistent with the two reactions sharing active-site requirements for engaging the phosphorus substrate. A focused directed evolution campaign from **G4** improved yield to 35% with 49% ee (**G6N**) and identified a variant with inverted enantioselectivity (**G6N.1**, 16% yield, 31% ee). Activity also extended to secondary phosphine oxides, providing **P**_**R**_-Ph^*i*^PrP­(O)–NH_2_ in 34% yield and 27% ee. Demonstration of initial P–N bond formation activity, enantiodivergence, and cross-substrate reactivity establishes a clear foundation from which further directed evolution is expected to improve both yield and enantioselectivity.

**Figure 4:**
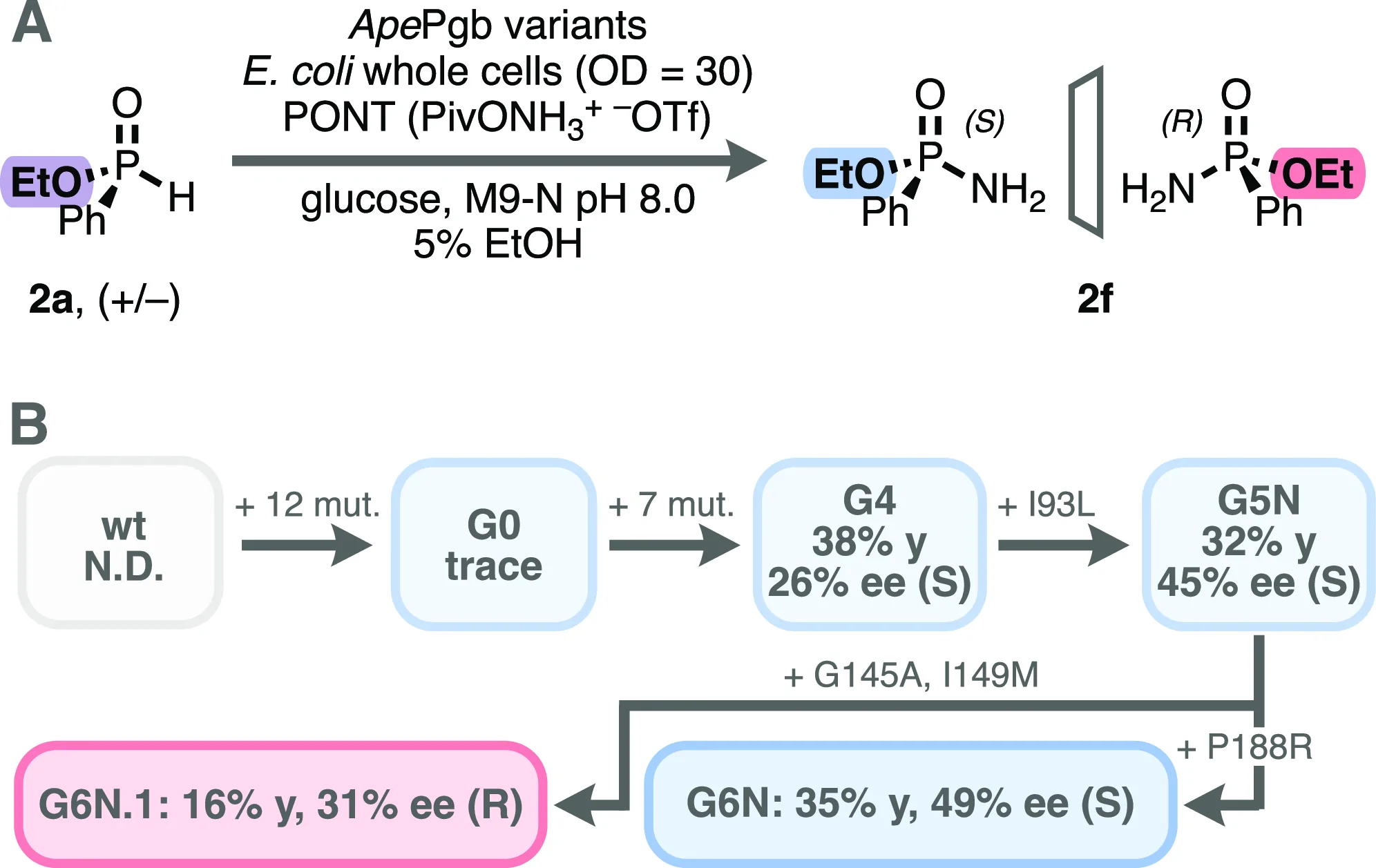
(A) Latent activity was discovered for nitrene insertion into the P–H bond of **2a**. (B) Divergent evolution of **G4** and the corresponding yield and enantioselectivity. Data represent the average (*n* = 3). The experiments were performed at analytical scale using whole-cell *E. coli* (Optical Density, OD_600_ = 30) expressing the *Ape*Pgb variants with 5 mM **2a** and 10 mM PONT in M9-N buffer (pH = 8.0) with 5 v/v% EtOH and 25 mM glucose under anaerobic conditions. See the Supporting Information for more details.

## Conclusion

In this work, we have described the engineering of *Aeropyrum pernix* protoglobin variants that catalyze enantioselective carbene and nitrene insertion into P–H bonds of secondary phosphine oxides and H–phosphinates—the first enzymatic reactions forming bonds directly at a phosphorus stereocenter through metallocarbenoid and metallonitrenoid intermediates. A dedicated directed evolution campaign, guided by dual-substrate validation at each round, produced enantiodivergent variants that deliver either P-configuration with good to excellent yield and enantioselectivity across a broad substrate scope. Mechanistic investigation established that selectivity arises from a stereoretentive kinetic resolution at phosphorus, a finding that, in practice, delivers two enantioenriched P-stereogenic compounds from a single reaction. A tailored 46-variant compilation plate systematically identified optimal enzymes for each substrate–reagent combination across structurally diverse P–H bond types. Screening of the phosphorus-adapted variant collection further revealed latent nitrene P–H insertion activity, extending the platform to the synthesis of enantioenriched P-stereogenic phosphinamides and phosphonamidates. These results add P–H to the growing repertoire of main-group X–H bonds functionalized enzymatically via carbene and nitrene transfer and demonstrate that broader exploration of the hemoprotein distribution can uncover reactivities absent from existing variant collections.

## Supplementary Material



## Notes

Materials and methods, experimental procedures, and copies of NMR spectra (PDF)
