# Supplementary material for: Enantiodivergent Carbon–Phosphorus Bond Formation via Enzymatic Carbene Insertion
Source: J Am Chem Soc. 2026 Aug 6;148(32):34791–7. doi: 10.1021/jacs.6c10375 (PMC13495749; doi:10.1021/jacs.6c10375)
Supplement: Supplementary file 1 [file ja6c10375_si_001.pdf]

Enantiodivergent Carbon–Phosphorus Bond Formation via Enzymatic Carbene Insertion

Hayden M. Carder, Ethan N. Lin, Daniel Roth, and Frances H. Arnold*

Division of Chemistry and Chemical Engineering, California Institute of Technology, Pasadena, California 91125, United States.

Table of Contents

1. General experimental methods 4

1.1 Materials and reagents 4

1.2 Instrumentation 6

2. Biological protocols 7

2.1 Transformation 7

2.1 Error-prone mutagenesis (epPCR) 7

2.3 Site-saturation mutagenesis (SSM) 9

2.4 Recombination via staggered extension process (StEP) 11

2.5 Reaction screening in 96-well plates 13

2.6 Validation of reaction in vials 14

2.7 Hemochrome assay 15

2.8 Protein purification 15

2.9 Whole plate sequencing (LevSeq) 16

3. Substrate and Authentic Standard Synthesis 17

3.1 Secondary phosphine oxides 17

3.2 H–phosphinates 19

3.3 Phosphine oxide product standards (carbene adducts) 21

3.4 Phosphinate product standards (carbene adducts) 39

3.5 Phosphinate product standards (nitrene adducts) 59

4. Discovery of Initial Activity and Control Reactions 62

4.1 Initial activity screening with $Ph_2P(O)H$ and EDA 62

4.2 Validation of initial hits and control reactions 64

4.3 Evaluating reactivity with WT *Ape*Pgb and known enzymes for X–H insertion 67

5. Directed Evolution 69

5.1 Carbene P–H Insertion: PhMeP(O)H (1a) 71

5.2 Carbene P–H Insertion: Ph(OEt)P(O)H (2a) 74

5.3 SSM Compilation Plate Screening for Substrate Scope 77

5.4 Nitrene P–H Insertion: Ph(OEt)P(O)H (2a) 85

6. DNA Sequences of Evolved Variants 87

6.1 Carbene P–H lineage 87

6.2 Selected variants from compilation plate screening 92
6.3 Nitrene P–H lineage 96
7. Substrate Scope 98
7.1 PhMeP(O)–CH_2CO_2Et (1b) 98
7.2 PhBnP(O)–CH_2CO_2Et (3b) 102
7.3 Ph^{i}PrP(O)–CH_2CO_2Et (4b) 107
7.4 PhCyP(O)–CH_2CO_2Et (5b) 112
7.5 Ph(4-FPh)P(O)–CH_2CO_2Et (6b) 117
7.6 Ph^{o}AnP(O)–CH_2CO_2Et (7b) 121
7.7 Ph(2-Np)P(O)–CH_2CO_2Et (8b) 124
7.8 Ph(OEt)P(O)–CH_2CO_2Et (2b) 127
7.9 (4-F)Ph(OEt)P(O)–CH_2CO_2Et (9b) 131
7.10 ^{p}Tol(OEt)P(O)–CH_2CO_2Et (10b) 135
7.11 (2-Np)(OEt)P(O)–CH_2CO_2Et (11b) 138
7.12 $PhCH_2CH_2$(OEt)P(O)–CH_2CO_2Et (12b) 141
7.13 ^{i}Bu(OEt)P(O)–CH_2CO_2Et (13b) 145
7.14 Ph(OEt)P(O)–CH_2CN (2d) 147
7.15 Ph(OEt)P(O)–alpha butyrolactone (2e) 150
7.16 Ph(OEt)P(O)–NH_2 (2f) 153
7.17 Ph^{i}PrP(O)–NH_2 (4c) 155
8. Mechanistic Studies and Absolute Stereochemistry 158
8.1 Synthesis and subjection of enantioenriched PhMeP(O)H (1a) 158
8.2 Scale up and isolation of PhMeP(O)–CH_2CO_2Me (1c) 163
8.3 Scale-up and isolation of Ph(OEt)P(O)–CH_2CO_2Me (2c) 166
8.4 Synthesis of enantioenriched phosphine oxides to assign absolute stereochemistry 169
9. References 175

1. General experimental methods

1.1 Materials and reagents

Chemistry: All commercially available reagents were purchased from Sigma-Aldrich, Alfa Aesar, Strem, Oakwood, Acros Organics, TCI, Chem-Impex, Combi-Blocks, or Ambeed and were used without purification unless otherwise indicated. Solvents for chromatography and extraction were purchased from Fisher Scientific as ACS grade or above and used without additional purification. Organic solutions were concentrated under reduced pressure on an IKA RV 10 rotary evaporator. Thin-layer chromatography (TLC) was performed on commercial Millipore Silica Gel 60 plates containing the F254 fluorescent indicator. Visualization of the developed chromatographs was performed by irradiation with UV light or by treating with $KMnO_4$ staining solution followed by heating. Flash column chromatography was performed using SiliaFlash P60 (230-400 mesh, Silicycle) or SiliaFlash R60 (20-45 µm, Silicycle). Ethyl diazoacetate was purchased from Sigma-Aldrich and amount added corrected for residual dichloromethane. Diazo reagents, diazoacetonitrile[1] (DAN) and α-butyrolactone diazo compound[2] (LAD) were synthesized according to reported procedures.

Media: Luria-Bertani (LB, L24400) and Terrific Broth (TB, T15000) media (Research Products International, RPI) were used for bacterial cell growth and protein expression. Distilled water (dH_2O) was used for growth media preparation. Media were supplemented with ampicillin (Amp) 0.1 mg/mL, and TB was additionally supplemented with glycerol according to the manufacturer's instructions. All media solutions were autoclaved for sterilization prior to use. Carbenicillin (Carb) 0.1 mg/mL was used to prepare LB_{carb} agar plates.

Reaction buffer: M9-N buffer was used for enzymatic reactions and was prepared from a 5X stock solution containing Na_2HPO_4 (34 g), KH_2PO_4 (15 g), and NaCl (2.5 g) in 1 L of double-distilled water (ddH_2O) followed by sterilization using an autoclave. The 1X M9-N buffer was then prepared by diluting 200 mL of the 5X stock solution with 797 mL of ddH_2O, followed by the addition of 1 mL $CaCl_2$ (0.1 M) and 2 mL $MgSO_4$ (1.0 M). The pH was adjusted to 8 using 1 M aq. NaOH solution.

Gel electrophoresis: The TAE buffer used in DNA gel electrophoresis was prepared from a 50X stock solution made by dissolving Tris free base (242 g), disodium EDTA (18.61 g), and glacial acetic acid (57.2 mL) in 1 L of ddH_2O. The 1X TAE buffer was then prepared by diluting 40 mL of the 50X stock solution with 1960 mL of dH_2O.

Cells and vector: T7 Express competent *Escherichia coli* (BL21) (New England Biolabs) cells were utilized for all experiments. Expression vector pET-22b(+) (Novagen) was used for cloning and expressing all enzyme variants. All genes had a C-terminal His_6-tag for purification and were inserted between the *Nde*I and *Xho*I restriction sites in the pET22b (+) vector (Novagen).

DNA Primers: Oligonucleotides were obtained from Integrated DNA Technologies (IDT DNA).

DNA amplification: PCRs were run using the Phusion® High Fidelity PCR Kit (New England Biolabs).

Gibson assembly: Gibson assembly mix was prepared by combining isothermal master mix and enzymes T5 exonuclease, Phusion® DNA polymerase, and Taq DNA ligase (New England Biolabs).[3]

Plasmid isolation and gel extraction: Monarch® Spin Plasmid Miniprep Kit (T1110L) Monarch® Spin DNA Gel Extraction Kit (T1120L)

1.2 Instrumentation

Nuclear magnetic resonance: ^{1}H and ^{13}C NMR spectra were recorded on a Bruker 400 MHz instrument at 25 °C referred to residual solvent peaks. Data are represented as follows: chemical shift, multiplicity (br = broad, s = singlet, d = doublet, t = triplet, q = quartet, quint = quintet, sext = sextet, sept = septet, m = multiplet), coupling constants (J) in Hertz (Hz), integration

High-resolution mass spectrometry (HRMS): spectra were acquired from the Caltech Mass Spectral Facility with an Agilent 6230 Series TOF LC/MS with an Agilent Jet Stream source in electrospray mode (ESI) and JEOL JMS-T2000GC AccuTOF using field ionization and field desorption (FI and FD).

Liquid-chromatography: High-performance liquid-chromatography mass spectroscopy (HPLC-MS) for standard reaction screening and assay yields were carried out using an Agilent 1200 series instrument equipped with an Agilent Poroshell C18 column (Agilent Poroshell 120 EC-C18, 4.6 x 50 mm, 2.7 µm) with a Poroshell 120 guard column (2.1 x 5 mm, 2.7 µm). Water and acetonitrile (MeCN) with 0.1% HPLC grade glacial acetic acid were used as eluents.

For normal phase chiral HPLC-UV, data were collected with an Agilent 1260 Infinity HPLC equipped with a Daivel Chiralpak IC, IB, IA, or OJH column (4.6 x 250 mm, 5 µm). Hexanes, isopropanol, and ethanol were used as eluents. Alternatively, enantiomeric excess was determined by an analytical chiral SFC using a Mettler SFC supercritical CO_2 analytical chromatography system or an Agilent 1260 Infinity II SFC-MS system (CO_2 = 1450 psi, column temperature = 40 °C) with Chiralcel IF-3 and IH-3 columns (4.6 mm x 25 cm); SIM detection at exact mass + 1 was performed.

Chromatographic purification was accomplished by flash chromatography using a Biotage Isolera One instrument with Sfär High-Capacity Duo columns.

Biological protocols: Sonication was performed using a Qsonica Q500 sonicator. UV-vis data were collected using a Shimadzu UV-1800 spectrophotometer. Centrifugation was carried out in an Allegra 25R tabletop centrifuge equipped with a TS-5.1-500 swinging bucket rotor.

2. Biological protocols

2.1 Transformation

T7 Express chemically competent *Escherichia coli* (*E. coli*) (BL21) (New England Biolabs) cells or electrocompetent *E. coli* were used for all experiments.

For chemically competent E. coli cells: Plasmids were mixed with competent cells on ice in PCR tubes. The mixture was kept on ice for 30 min, after which time transformation was accomplished by heat shocking the mixture in a 42 ºC water bath for 10 seconds. After a 5-minute recovery on ice, the cells were diluted with SOC medium and plated on LB_{carb} (carbenicillin) agar plates.

For electrocompetent E. coli cells: In case plasmids were constructed by Gibson cloning, dialysis of plasmids was performed prior to transformation using MF-Millipore™ 0.05 µm MCE membrane filters (non-sterile 13 mm) and ddH_2O for 20 minutes. Plasmids were mixed with competent cells in a cuvette (1 mm gap) on ice. The cells were electroporated with a BIORAD MicroPulser™ (Settings: Bacteria) and immediately resuspended with SOC or LB media. The cells were recovered by shaking at 37 °C for 10–30 minutes, and then they were plated on LB_{carb} agar plates. The agar plates with the plated cells were incubated at 37 ºC for 12–16 h and stored at 4 ºC until the libraries or single colonies were picked. Plasmids were isolated from stationary-phase cultures by miniprep (New England Biolabs), and Sanger sequencing was performed by Laragen, Inc. (Culver City, CA) using the T7 promoter and T7 terminator primers.

2.1 Error-prone mutagenesis (epPCR)

Random mutations were introduced using error-prone PCR (epPCR). Primers 005 and 006 were used to amplify the DNA region encoding the full-length protein of interest (**Table S1**). Primers 007 and 008 were used to amplify the backbone fragment (pET-22b(+) vector) containing a gene encoding for ampicillin resistance using Phusion polymerase, and the template plasmid DNA. PCRs were run using Taq polymerase (New England Biolabs) in the presence of 200, 300, 400, 500, and 600 µM $MnCl_2$ following a reported protocol (**Table S2** and **Table S3**).[4]

Upon completion of PCRs, the remaining template plasmid DNA was digested with the *Dpn*I restriction enzyme at 37 ºC for 1 hours. The PCR products were purified by gel electrophoresis (1% agarose in 1X TAE buffer containing 1X SYBR Gold nucleic acid Stain) and extracted using New England Biolabs DNA cleanup and gel extraction kit. Fragments were then assembled into a circular plasmid using the Gibson assembly protocol, incubating at 50 ºC for 1 hour.[3] Gibson products were used to transform competent *E. coli* cells. Libraries generated from 200, 300, 400, 500, and 600 µM $MnCl_2$ were screened, and the library with the desired error rate was further evaluated.

Table S1: Primers used in epPCR.

Primers	Sequence (5' → 3')
005	GA AAT AAT TTT GTT TAA CTT TAA GAA GGA GAT ATA CAT ATG
006	GCC GGA TCT CAG TGG TGG TGG TGG TGG TGC TCG AG
007	CAT ATG TAT ATC TCC TTC TTA AAG TTA AAC AAA ATT ATT CT
008	CTC GAG CAC CAC CAC CAC CAC CAC TGA GAT CCG GC

Table S2: Set-up of PCR reaction for epPCR library construction.

	$MnCl_2$ concentration (µM)		
	**300**	**400**	**500**
**Reagents**		**Amount (µL)**	
ddH_2O	49.4	39.4	29.4
10X Taq buffer	10	10	10
dNTP mix (10 mM)	4	4	4
$MnCl_2$ (1 mM)	30	40	50
Forward primer (10 µM)	2	2	2
Reverse primer (10 µM)	2	2	2
Plasmid of interest (5 – 50 ng/µL)	1	1	1
Taq DNA polymerase (5000 U/mL)	1.6	1.6	1.6
Total reaction volume	100	100	100

Table S3: Thermocycler conditions for Taq epPCR library construction.

Step	Temperature (°C)	Time (min:s)	Number of Cycles
Initial denaturation	98	05:00	1
Denaturation	98	00:30	
Annealing	55	00:30	30
Extension	72	01:30	
Extension	72	5:00	1
Hold	10	-	

2.3 Site-saturation mutagenesis (SSM)

SSM was performed using a modified QuikChange™ mutagenesis protocol using primers with NNK degenerate codons and primers within the ampicillin resistance cassette (UniF and UniR), forming the plasmid of interest in two pieces (Table S4) with an overlap for Gibson assembly in the gene encoding the protein of interest (and where the mutations are introduced) and an overlap for Gibson assembly in the gene encoding β-lactamase (which confers ampicillin resistance). Example primers for V63X are provided in **Table S4**. The PCR reactions were performed according to the protocols in **Table S5** and **Table S6**.

Upon completion of PCRs, the remaining template plasmid DNA was digested with the *Dpn*I restriction enzyme at 37 ºC for 1 hour. The PCR products were purified by gel electrophoresis (1% agarose in 1X TAE buffer containing 1X SYBR Gold nucleic acid Stain) and extracted using New England Biolabs DNA cleanup and gel extraction kit. Fragments were then assembled into a circular plasmid using the Gibson assembly protocol, incubating at 50 ºC for 1 hour.[3] Gibson products were used to transform competent *E. coli*.

Table S4: Primers used in SSM PCRs.

Primers	Sequence (5' → 3')
UniF	CCA ACT TAC TTC TGA CAA CGA TCG GAG GAC CGA AGG AGC TAA CCG CTT TTT TGC
UniR	CGA TCG TTG TCA GAA GTA AGT TGG CCG CAG TGT TAT CAC TCA TGG TTA TGG CAG
V63X_forward	GAC CTG GCG GGT GGT TGG NNK GCA TCA AAT GAG
V63X_reverse	CCA ACC ACC CGC CAG GTC AAG GAT CTC ATC

Table S5: Set-up of PCR reaction for SSM library construction.

Reagents	Amount (µL)
ddH_2O	32
5X HF buffer	10
dNTP mix (10 mM)	1
DMSO	0.5
Forward primer (10 µM)	2.5
Reverse primer (10 µM)	2.5
Plasmid of interest (5 – 50 ng/µL)	0.5
Phusion® High-Fidelity (2000 U/mL)	1
Total reaction volume	50

Table S6: Thermocycler conditions for SSM library construction.

Step	Temperature (°C)	Time (min:s)	Number of Cycles
Initial denaturation	98	00:30	1
Denaturation	98	00:10	
Annealing	Temperature gradient (55 – 72 °C)*	00:15	30
Extension	72	02:30	
Extension	72	10:00	1
Hold	10	-	

*The annealing temperature was adjusted to the T_m – 3 to 5 °C of the constructed SSM primer. In the case of the 22-codon method, the primer with the highest T_m was used as a reference.

2.4 Recombination via staggered extension process (StEP)

Staggered extension process (StEP) PCR was performed using an equimolar mixture of plasmids of the variants to be recombined following a reported procedure.[5] The initial PCR was performed using primers 005 and 006 (**Table S7** and **Table S8**). Multiple reactions were set up at varying annealing temperatures (50 → 70 ºC). Based on analytical gel analysis, the successful reaction performed at the lowest temperature was carried forward.

The PCR product was digested with *Dpn*I (1 µL) at 37 ºC for at least 1 hour. The digest DNA product was further amplified (2nd PCR reaction) without prior purification (**Table S9** and **Table S10**). Primers 005 and 006 were used to amplify the gene of interest.

The PCR products were purified by gel electrophoresis (1% agarose in 1X TAE buffer containing 1X SYBR Gold nucleic acid Stain) and extracted using New England Biolabs DNA cleanup and gel extraction kit. The PCR product was cloned into a pET22b (+) vector between restriction sites *Nde*I and *Xho*I to construct a circular plasmid using the Gibson assembly protocol, incubating at 50 ºC for 1 hour.[3] Gibson products were used to transform competent *E. coli* cells.

Table S7: Set-up of initial PCR reaction for StEP recombination library.

Reagents	Amount (µL)
ddH$_2$O	14.27
5X HF buffer	2
dNTP mix (10 mM)	0.4
Primer 005 (1 µM)	1
Primer 006 (1 µM)	1
Plasmid of interest (250 ng total)	1
Phusion® High-Fidelity (2000 U/mL)	0.33
Total reaction volume	20

Table S8: Thermocycler conditions for 1st PCR in StEP library construction.

Step	Temperature (°C)	Time (min:s)	Number of Cycles
Initial denaturation	95	05:00	1
Denaturation	95	00:10	
Annealing	55	00:01	120
Extension	Temperature gradient (50 – 72 °C)*	00:01	
Hold	10	-	

*PCR tubes were distributed at an equal distance, starting from the lowest temperature.

Table S9: Set-up of 2nd PCR reaction for StEP recombination library.

Reagents	Amount (µL)
ddH_2O	30
5X GC buffer	10
DMSO	2
dNTP mix (10 mM)	1
Primer 005 (10 µM)	2
Primer 006 (10 µM)	2
Digested DNA mixture	2.5
Phusion® High-Fidelity (2000 U/mL)	0.5
Total reaction volume	50

Table S10: Thermocycler conditions for 2nd PCR in StEP library construction.

Step	Temperature (°C)	Time (min:s)	Number of Cycles
Initial denaturation	98	00:30	1
Denaturation	98	00:10	
Annealing	Temperature gradient (55 – 72 °C)*	00:15	30
Extension	72	01:30	
Extension	72	10:00	1
Hold	10	-	

2.5 Reaction screening in 96-well plates

From agar plates containing *E. coli* BL21 cells transformed with protoglobin variant libraries, 86 single colonies were picked using sterile toothpicks and used to inoculate starter cultures (500 µL of LB_{amp}) in sterilized 96-well deep-well culture plates. Eight wells on each plate were inoculated with single colonies of the parent enzyme (parent controls), and two wells were inoculated with a sterile toothpick (sterile controls). These starter culture plates were sealed with air-permeable tape and grown at 37 °C and 220 rpm with 80% humidity in a Multitron Infors shaker for 12–16 hours. Subsequently, 50 µL of the starter culture were used to inoculate expression cultures (950 µL TB_{amp}) in a separate, sterilized 96-well deep-well plate. Similarly, 50 µL of the overnight culture were added to 50 µL of sterilized 50% glycerol to prepare a glycerol stock replica, which was stored at -80 °C. This overnight culture was also used to perform LevSeq following a reported procedure.[6] Expression cultures were incubated at 37 °C and 220 rpm with 80% humidity for 2 hours. The plates were then cooled on ice for 30 minutes before inducing protein expression with isopropyl β-D-1-thiogalactopyranoside (IPTG, 0.5 mM) and δ-aminolevulinic acid (ALA, 1.0 mM). Expression was carried out at 22 °C and 220 rpm for 20–24 hours. The expression cultures were then centrifuged (4,000x*g*, 10 minutes, 4 °C), and the supernatant was removed. The cell pellets were resuspended in 380 µL of M9-N (pH = 8) supplemented with glucose (25 mM) using an orbital shaker (900 rpm), and the plates were transferred into a Coy anaerobic chamber (~0–100 ppm O_2). Cell suspensions were supplemented with 20 µL of [P] (100 mM) and EDA (75 mM). The plates were sealed with an adhesive aluminum-foil seal and incubated on an orbital shaker (700 rpm) in the Coy anaerobic chamber at room temperature overnight.

For workup and analysis, the plates were removed from the Coy chamber and HPLC-grade MeCN (800 µL/well) was added to each well. The resulting suspensions were mixed by pipetting, then sealed and cooled for 20 min at -20 °C to further enhance precipitation of cell debris. The plates were then centrifuged (5,000x*g*, 15 minutes, 4 °C) to remove protein and cell debris from the supernatant. The supernatant (200 µL/well) was transferred to a polypropylene 96-well microtiter plate (Agilent), sealed with an NAL-96 pierceable film (USA Scientific), and subjected to reverse-phase HPLC-MS analysis. For better performing variants, 20 µL or 50 µL of the supernatant were used and were further diluted with MeCN to a total volume of 200 µL for HPLC analysis. Activity was determined by achiral HPLC-MS analysis using a Poroshell C18 column. The product MS peak was integrated using built-in analysis, and wells showing signals higher than that of the parent wells were identified as “hits”. In some cases, potential hit wells were further analyzed by normal phase chiral HPLC analysis to determine enantioselectivity using a specified method. To prepare a sample for normal phase HPLC, the MeCN from the supernatant was removed by blowing with air to provide ~400 µL; the aqueous phase was extracted with cyclohexane/ethyl acetate (1:1, 1 mL). Wells displaying improved activity and/or enantioselectivity were carried on to validation.

2.6 Validation of reaction in vials

Following screening, wells displaying an optimal combination of activity and enantioselectivity relative to parent controls were streaked from the corresponding glycerol stocks onto individual LB_{carb} agar plates. A single colony was picked and grown in 6 mL LB_{amp} overnight at 37 °C and 250 rpm. Subsequently, 1 mL of this starter culture was used to inoculate 50 mL of TB_{amp} in a 125-mL sterilized Erlenmeyer flask. The starter culture was also used to isolate variant plasmids using a miniprep kit, and their DNA sequence was determined by Sanger sequencing (Laragen, Inc., Culver City, CA). The expression culture was incubated at 37 °C and shaken at 220 rpm until the optical cell density at 600 nm (OD_{600}) reached ~0.8–1.0. The expression culture was then cooled on ice for 30 minutes before inducing protein expression with IPTG (0.5 mM) and ALA (1.0 mM). Expression was carried out at 22 °C and 220 rpm for 20–24 hours. The expression culture was then transferred to a 50-mL Falcon™ tube and centrifuged (4000x*g*, 10 minutes, 4 °C). The supernatant was removed, and the pellets were resuspended in M9-N buffer and normalized to an OD_{600} = 30 with M9-N buffer. Subsequently, an aliquot of the normalized cell suspension (360 µL) was added to a 2.0-mL screw-cap vial (Agilent) and glucose (20µL, 500 mM, $[glc]_{final}$ = 25 mM) and transferred into a Coy anaerobic chamber (~0–100 ppm O_2). For each variant, four technical replicates were performed. Cell suspensions were supplemented with 20 µL of [P] (100 mM) and EDA (75 mM). The vials were capped and incubated on an orbital shaker (700 rpm) in the Coy anaerobic chamber at room temperature overnight. The vials were then removed from the Coy chamber.

Three technical triplicates were quenched for yield determination using LCMS. For these samples, 800 µL of a 100 µM solution of papaverine hydrochloride dissolved in 95:5 (v/v) MeCN:H_2O were added to each vial. The solution was mixed while transferring to an Eppendorf tube and then cooled to -20 °C to precipitate cell debris (20 min). After centrifugation (14,000x*g*, 15 minutes, 4 °C), 50 µL of the supernatant were transferred into a 2.0-mL screw cap vial containing 950 µL MeCN. The internal standard (papaverine hydrochloride) and product peaks were integrated using built-in OpenLab CDS analysis software (Agilent) and quantified using calibration curves prepared and measured at the same time as sample analysis.

One replicate was quenched to determine enantioselectivity. For this sample, IPA (50 µL) and cyclohexane/ethyl acetate (1:1, 1 mL) were added. The solution was transferred to an Eppendorf tube. The sample was shaken at 2000 rpm for 10 min and then centrifuged to promote layer separation (14,000x*g*, 1 minute). The organic layer was transfer to a 2.0-mL screw cap vial containing a glass insert.

The variant displaying the optimal combination of activity and selectivity compared to the parent enzyme was carried forward in evolution.

2.7 Hemochrome assay

Heme concentration was determined using the hemochrome assay with clarified cell lysate.[7] Lysate was obtained by sonication (3 minutes, 1 second on, 2 seconds off, 35% amplitude, on wet ice) of whole cells resuspended in M9-N buffer to an OD_{600} = 30. The cell debris was removed by centrifugation (14,000x*g*, 10 minutes, 4 °C), and the supernatant was stored on ice until further use. To a cuvette, 500 µL of lysate and 500 µL of freshly prepared solution I [0.2 M NaOH, 40% (v/v) pyridine, 0.5 mM $K_3Fe(CN)_6$] were added. The UV-vis spectrum (380–620 nm) of the oxidized Fe(III) state was recorded immediately. Sodium dithionite (10 µL of 0.5 M solution in 0.5 M NaOH) was added, the mixture was thoroughly mixed through pipetting, and the UV-vis spectrum of the reduced Fe(II) state was promptly recorded. The heme concentration was calculated using Beer's law, with the extinction coefficient $\varepsilon[557r_{educed}–540_{oxidized}]$ = 23.98 $mM^{-3c}m^{-1}$ for heme, considering dilution factors (here: 2-fold).

2.8 Protein purification

E. coli transformed with pET22b(+) constructs encoding various protoglobin variants were grown in 25 mL LB_{amp} overnight. Subsequently, 5 mL of this starter culture were used to inoculate 1 L of TB_{amp} in a 2-L sterilized Erlenmeyer flask. The expression culture was incubated at 37 °C and shaken at 140 rpm until the OD_{600} reached ~0.8–1.0. The expression culture was then cooled on ice for 30 minutes before inducing protein expression with IPTG (0.5 mM) and ALA (1.0 mM). Expression was carried out at 22 °C, 180 rpm for 20–22 hours with a shaking radius of 25 mm. Once expression was finished, the cultures were centrifuged (4,000x*g*, 10 minutes, 4 °C) and stored overnight at -20 °C. After thawing, the wet cell pellet was weighed, and four times the wet cell weight amount of buffer A (50 mM KPi, 20 mM imidazole, 100 mM NaCl, pH = 7.5) was used to resuspend the pellet. Lysate was obtained by sonication (3 minutes, 1 second on, 2 seconds off, 35% amplitude) on wet ice. The cell debris was removed by centrifugation (14,000x*g*, 10 minutes, 4 °C). The supernatant was combined and applied to a 1-mL Ni^{2+}-NTA column equilibrated with buffer A at 4 °C on an AKTA Xpress protein purification system. The protein of interest was then eluted using buffer B (50 mM KPi, 150 mM or 300 mM imidazole, 100 mM NaCl, pH = 7.5). The pooled fractions were dialyzed in 4 L of storage buffer (50 mM KPi, 150 mM NaCl, pH 8.0) at 4 °C overnight. The dialysis buffer was exchanged, and another buffer exchange using storage buffer was performed at 4 °C overnight. The resultant protein solution was concentrated to a desired volume with a 10 or 20 kDa MWCO filter. The heme concentration was measured by the hemochrome assay, diluting 10 µL purified enzyme in 490 µL storage buffer. Purified proteins were aliquoted, flash-frozen with liquid nitrogen and stored at −80 °C.

2.9 Whole plate sequencing (LevSeq)

To obtain sequence data for 96-well plates containing *E. coli* harboring protoglobin variants, a reported protocol was followed using the indicated barcode plate.[6] Initially, a PCR master mix was prepared (**Table S11**). In a 384-well plate, 7 µL of the PCR master mix were transferred into each well. Then, 2 µL of 1-µM barcode-linked primer mix and 2 µL of the LB_{amp} overnight culture were added. After sealing, the colony PCR is performed using a thermocycler (**Table S12**).

Table S11: PCR reaction for LevSeq library.

Reagents	Amount (µL) for 1 plate	Amount (µL) for 8 plates
ddH_2O	770	5390
Thermopol Buffer	144	1008
DMSO (Mol-Bio grade)	57.6	408.2
dNTP mix (10 mM)	28.8	201.6
Taq DNA polymerase (5000 U/mL)	7.2	50.4

Table S12: Thermocycler conditions LevSeq library construction.

Step	Temperature (°C)	Time (min:s)	Number of Cycles
Initial denaturation	95	05:00	1
Denaturation	95	00:20	
Annealing	Touch down (68 → 63.5°C) (drop by 0.5°C / cycle)	00:25	9
Extension	68	02:00	
Denaturation	95	00:20	24
Annealing and Extension	68	01:30	
Extension	68	5:00	1
Hold	4	-	

The PCR products were combined per plate by taking 5 µL of each well. Ten µL of 6X loading dye were added to an aliquot of 50 µL of this combined sample and purified by gel electrophoresis (1% agarose in 1X TAE buffer containing 1X SYBR Gold nucleic acid Stain). Then desired bands were excised and extracted using New England Biolabs DNA cleanup and gel extraction kit. DNA concentrations were normalized and pooled and prepared for Oxford Nanopore sequencing following a standard protocol.[8] LevSeq data analysis was performed using the LevSeq software provided on the LevSeq Github page (https://github.com/fhalab/LevSeq).

3. Substrate and Authentic Standard Synthesis

3.1 Secondary phosphine oxides

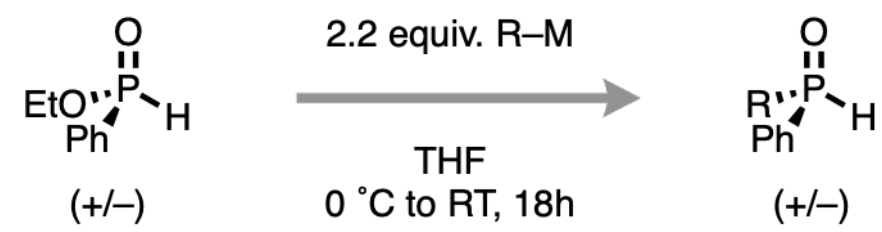

Secondary phosphine oxides were prepared according to previously reported procedures from commercially available ethyl phenylphosphinate.[9] An oven dried 40-mL vial equipped with a stir bar was evacuated and backfilled with N_2 three times. The vial was charged with the corresponding organometallic reagent (2.2 equiv, Mg or Li) and diluted with anhydrous THF (0.6 M final). The solution was cooled to 0 ˚C. A solution of ethyl phenylphosphinate (1 equiv, 6 M in THF) was added dropwise over 5 minutes. The mixture was allowed to warm to room temperature and stirred overnight. The reaction was quenched with 1 N HCl (3.0 equiv.), and the mixture was extracted with three times with DCM. The combined organic phases were washed with brine, dried over Na_2SO_4, filtered, and concentrated in *vacuo*. The crude product was purified by silica gel chromatography (hexanes/ethyl acetate or ethyl acetate/methanol, if necessary) to afford the desired product. All characterization data agreed with previously reported values.

Notes: Most substrates were prepared on a 3-mmol scale. If the organometallic reagent was not readily accessible, the Grignard was synthesized using the appropriate aryl bromide.

Methyl(phenyl)phosphine oxide (1a)[10]

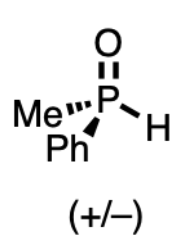

CAS 19315-13-0
MW 140.12
^{1}H NMR (400 MHz, $CDCl_3$) δ 7.76 – 7.66 (m, 2H), 7.65 (dq, *J* = 472.3, 3.9 Hz, 1H), 7.62 – 7.55 (m, 1H), 7.55 – 7.48 (m, 2H), 1.81 (ddd, *J* = 13.9, 3.8, 0.6 Hz, 3H).
^{31}P NMR (162 MHz, $CDCl_3$) δ 20.42.

Benzyl(phenyl)phosphine oxide (3a)[10]

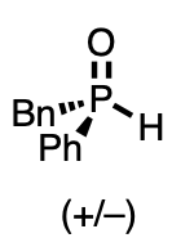

CAS 177334-60-0
MW 216.22
^{1}H NMR (400 MHz, $CDCl_3$) δ 7.60 – 7.38 (m, 5H), 7.48 (dt, *J* = 475.2, 3.8 Hz, 1H), 7.25 (m, 3H), 7.06 (dt, *J* = 7.4, 2.3 Hz, 2H), 3.55 – 3.29 (m, 2H).
^{31}P NMR (162 MHz, $CDCl_3$) δ 29.72.

Isopropyl(phenyl)phosphine oxide (4a)[9]

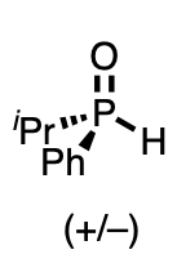

CAS 63762-20-9
MW 168.18
^{1}H NMR (400 MHz, $CDCl_3$) δ 7.72 – 7.65 (m, 2H), 7.62 – 7.55 (m, 1H), 7.54 – 7.47 (m, 2H), 7.83 – 6.63 (m, 1H), 2.14 (ddqd, *J* = 14.3, 11.3, 7.2, 2.5 Hz, 1H), 1.17 (ddd, *J* = 18.1, 12.4, 7.2 Hz, 6H).
^{31}P NMR (162 MHz, $CDCl_3$) δ 39.49.

Cyclohexyl(phenyl)phosphine oxide (5a)[10]

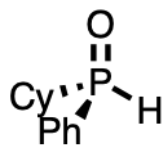

(+/–)

CAS 75354-35-7

MW 208.24

^{1}H NMR (400 MHz, $CDCl_3$) δ 7.71 – 7.64 (m, 2H), 7.62 – 7.54 (m, 1H), 7.51 (tdd, *J* = 8.3, 2.7, 1.1 Hz, 2H), 7.21 (dd, *J* = 457.2, 2.5 Hz, 1H), 1.99 – 1.75 (m, 5H), 1.70 (dt, *J* = 9.3, 2.1 Hz, 1H), 1.43 – 1.12 (m, 5H).

^{31}P NMR (162 MHz, $CDCl_3$) δ 36.81.

Para-fluorophenyl(phenyl)phosphine oxide (6a)[11]

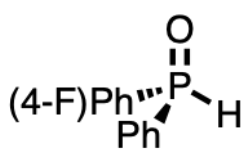

(+/–)

CAS 1478603-57-4

MW 220.18

^{1}H NMR (400 MHz, $CDCl_3$) δ 8.08 (d, *J* = 482.7 Hz, 1H), 7.75 – 7.65 (m, 4H), 7.62 – 7.56 (m, 1H), 7.55 – 7.48 (m, 2H), 7.20 (td, *J* = 8.7, 2.1 Hz, 2H).

^{19}F NMR (376 MHz, $CDCl_3$) δ -105.37 (d, *J* = 2.0 Hz, 1F).

^{31}P NMR (162 MHz, $CDCl_3$) δ 20.17.

Ortho-anisole(phenyl)phosphine oxide (7a)[10]

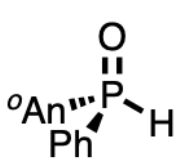

(+/–)

CAS 1061746-02-8.

MW 232.22

^{1}H NMR (400 MHz, $CDCl_3$) δ 8.18 (d, *J* = 499.3 Hz, 1H), 7.84 – 7.70 (m, 3H), 7.58 – 7.48 (m, 2H), 7.46 (tdd, *J* = 8.5, 3.0, 1.1 Hz, 2H), 7.16 – 7.07 (m, 1H), 6.92 (dd, *J* = 8.4, 5.7 Hz, 1H), 3.79 (d, *J* = 1.1 Hz, 3H).

^{31}P NMR (162 MHz, $CDCl_3$) δ 14.27.

Napthyl(phenyl)phosphine oxide (8a)[12]

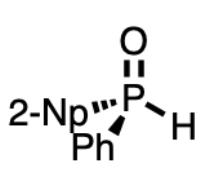

(+/–)

CAS N.D.

MW 252.25

^{1}H NMR (400 MHz, $CDCl_3$) δ 8.37 (d, *J* = 15.7 Hz, 1H), 8.21 (d, *J* = 488.6 Hz, 1H), 7.97 – 7.91 (m, 2H), 7.91 – 7.87 (m, 1H), 7.75 (ddt, *J* = 13.8, 6.9, 1.4 Hz, 2H), 7.67 – 7.54 (m, 4H), 7.51 (dddd, *J* = 8.4, 6.4, 3.3, 1.6 Hz, 2H).

^{31}P NMR (162 MHz, $CDCl_3$) δ 21.55.

3.2 H–phosphinates

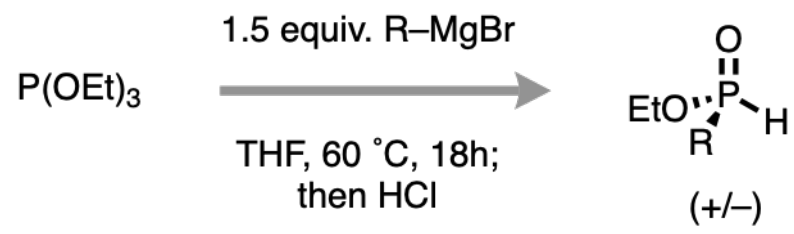

H–phosphinates were prepared according to previously reported procedures from commercially available triethyl phosphite.[9] An oven dried 40-mL vial equipped with a stir bar was evacuated and backfilled with N_2 three times. The vial was charged with the corresponding organometallic reagent (1.5 equiv, Mg or Li) and diluted with anhydrous THF (2.2 M final). A solution of triethyl phosphite (1 equiv, 5 M in THF) was added dropwise over 5 minutes. The mixture was heated to 60 ˚C and stirred overnight. The reaction was cooled to room temperature, filtered through a pad of celite, and quenched with 1 N HCl until pH ~ 2. The mixture was diluted with water and extracted with three times with EtOAc. The combined organic phases were washed with brine, dried over Na_2SO_4, filtered, and concentrated in *vacuo*. The crude product was purified by silica gel chromatography (hexanes/ethyl acetate or ethyl acetate/methanol, if necessary) to afford the desired product. All characterization data agreed with previously reported values.

Notes: Most substrates were prepared on a 15-mmol scale. If the organometallic reagent was not readily accessible, the Grignard was synthesized using the appropriate aryl bromide.

Ethyl (4-fluorophenyl)phosphinate (9a)[13]

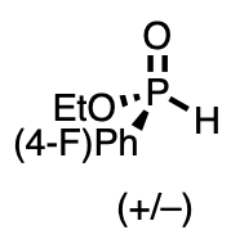

CAS 80094-64-0
MW 188.14
^{1}H NMR (400 MHz, $CDCl_3$) δ 7.86 – 7.75 (m, 2H), 7.59 (d, *J* = 566.5 Hz, 1H), 7.21 (td, *J* = 8.7, 2.5 Hz, 2H), 4.23 – 4.12 (m, 2H), 1.39 (t, *J* = 7.1 Hz, 3H).
^{19}F NMR (376 MHz, $CDCl_3$) δ -104.53 (d, *J* = 2.7 Hz, 1F).
^{31}P NMR (162 MHz, $CDCl_3$) δ 22.91.

Ethyl (p-tolyl)phosphinate (10a)[13]

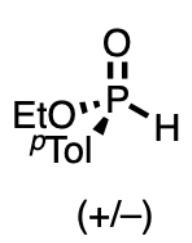

CAS 38766-17-5
MW 184.18
^{1}H NMR (400 MHz, $CDCl_3$) δ 7.72 – 7.58 (m, 2H), 7.56 (d, *J* = 561.2 Hz, 1H), 7.40 – 7.29 (m, 2H), 4.28 – 3.97 (m, 2H), 2.42 (d, *J* = 1.0 Hz, 3H), 1.37 (t, *J* = 7.1 Hz, 3H).
^{31}P NMR (162 MHz, $CDCl_3$) δ 25.02.

Ethyl (2-napthyl)phosphinate (11a)[13]

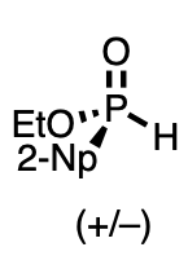

CAS 821009-67-0
MW 220.221
^{1}H NMR (400 MHz, $CDCl_3$) δ 8.48 – 8.35 (m, 2H), 8.02 – 7.86 (m, 3H), 7.74 (ddd, *J* = 11.7, 8.4, 1.5 Hz, 1H), 7.68 – 7.52 (m, 2H), 7.02 (s, 1H), 4.42 – 3.97 (m, 2H), 1.41 (t, *J* = 7.1 Hz, 3H).
^{31}P NMR (162 MHz, $CDCl_3$) δ 24.67.

Ethyl (phenethyl)phosphinate (12a)[13]

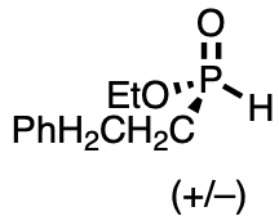

CAS 86552-39-8

MW 198.20

^{1}H NMR (400 MHz, $CDCl_3$) δ 7.34 – 7.27 (m, 2H), 7.25 – 7.18 (m, 3H), 7.10 (dt, *J* = 531.9, 1.9 Hz, 1H), 4.27 – 3.98 (m, 2H), 2.93 (dt, *J* = 12.7, 8.3 Hz, 2H), 2.30 – 2.05 (m, 2H), 1.37 (t, *J* = 7.0 Hz, 3H).

^{31}P NMR (162 MHz, $CDCl_3$) δ 36.79.

Ethyl (2-methylpropyl)phosphinate (13a)[13]

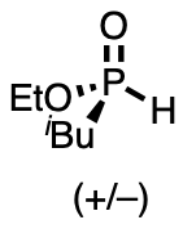

CAS 16259-93-1

MW 150.16

^{1}H NMR (400 MHz, $CDCl_3$) δ 7.15 (dt, *J* = 525.6, 2.1 Hz, 1H), 4.28 – 3.97 (m, 2H), 2.13 (ddt, *J* = 13.5, 12.6, 6.7 Hz, 1H), 1.80 – 1.58 (m, 2H), 1.36 (td, *J* = 7.1, 0.6 Hz, 3H), 1.06 (dt, *J* = 6.7, 1.0 Hz, 6H).

^{31}P NMR (162 MHz, $CDCl_3$) δ 37.37.

3.3 Phosphine oxide product standards (carbene adducts)

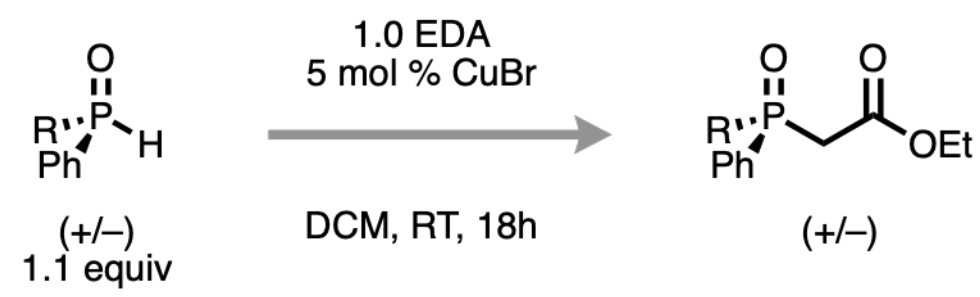

Authentic, racemic standards for the C–P bond formation between phosphine oxides and EDA were prepared according to previously reported procedures from synthesized substrates (see Section 3.1).[14] An oven dried 2-dram vial equipped with a stir bar was charged with the corresponding secondary phosphine oxide (1.1 equiv, 0.22 mmol) and copper(I) bromide (0.05 equiv., 0.01 mmol, 1.4 mg). The vial was evacuated and backfilled with N_2 three times. To the vial was added DCM (1 mL, 0.22 M) followed by EDA in DCM (1 equiv., 0.2 mmol, 25 μL). The reaction was stirred overnight (18 h) at room temperature. The reaction was concentrated *in vacuo* and crude material purified by silica gel chromatography (hexanes/ethyl acetate or ethyl acetate/methanol, if necessary) to afford the desired product (15-65% yield).

(Methyl-phenyl-phosphinoyl)-acetic acid ethyl ester (1b)

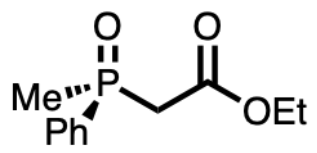

(+/–)

MW 226.21

^{1}H NMR (400 MHz, $CDCl_3$) δ 7.80 – 7.68 (m, 2H), 7.61 – 7.52 (m, 1H), 7.55 – 7.45 (m, 2H), 4.09 (qd, *J* = 7.2, 3.3 Hz, 2H), 3.22 – 3.01 (m, 2H), 1.93 (d, *J* = 13.5 Hz, 3H), 1.14 (t, *J* = 7.1 Hz, 3H).

^{13}C NMR (101 MHz, $CDCl_3$) δ 166.75 (d, *J* = 4.4 Hz), 132.69 (d, *J* = 100.9 Hz), 132.41 (d, *J* = 2.8 Hz), 130.25 (d, *J* = 9.8 Hz), 128.86 (d, *J* = 12.1 Hz), 61.72, 40.62 (d, *J* = 58.9 Hz), 16.02 (d, *J* = 72.8 Hz), 14.11.

^{31}P NMR (162 MHz, $CDCl_3$) δ 31.92.

HRMS (AccuTOF-FD) m/z: M+ calcd for C11H15O3P: 226.0753; found 226.0758.

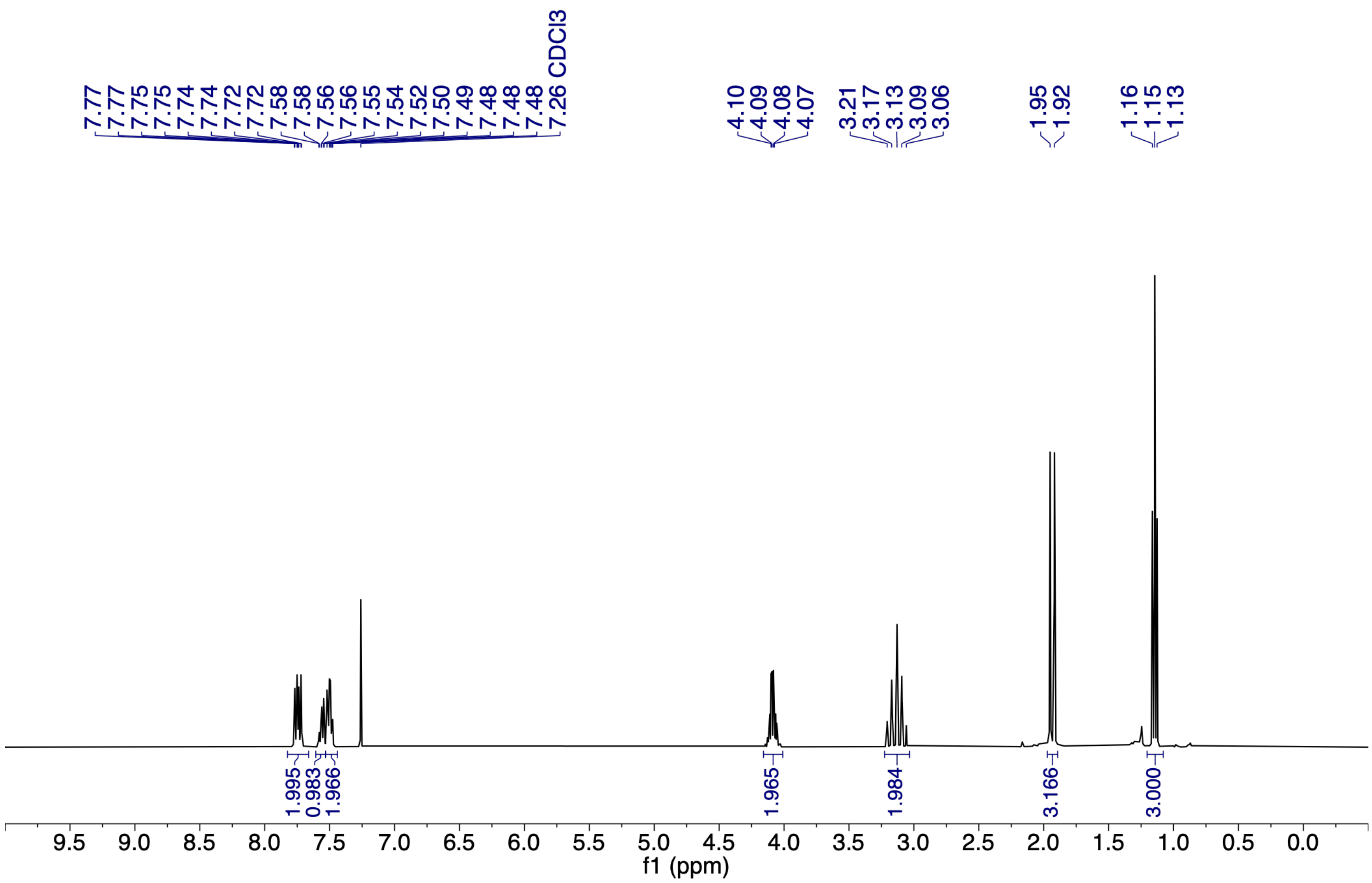

(Methyl-phenyl-phosphinoyl)-acetic acid ethyl ester (1b)

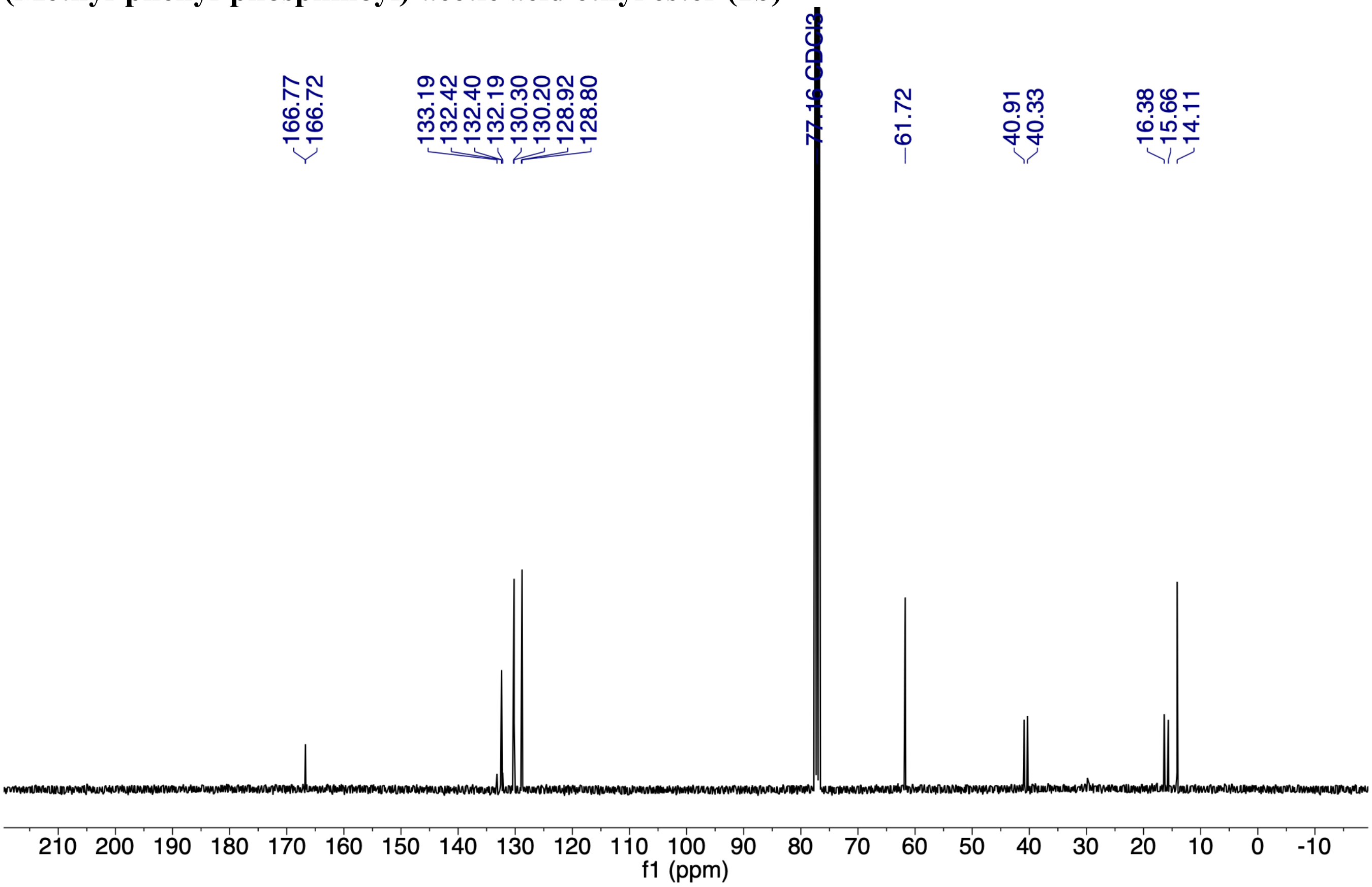

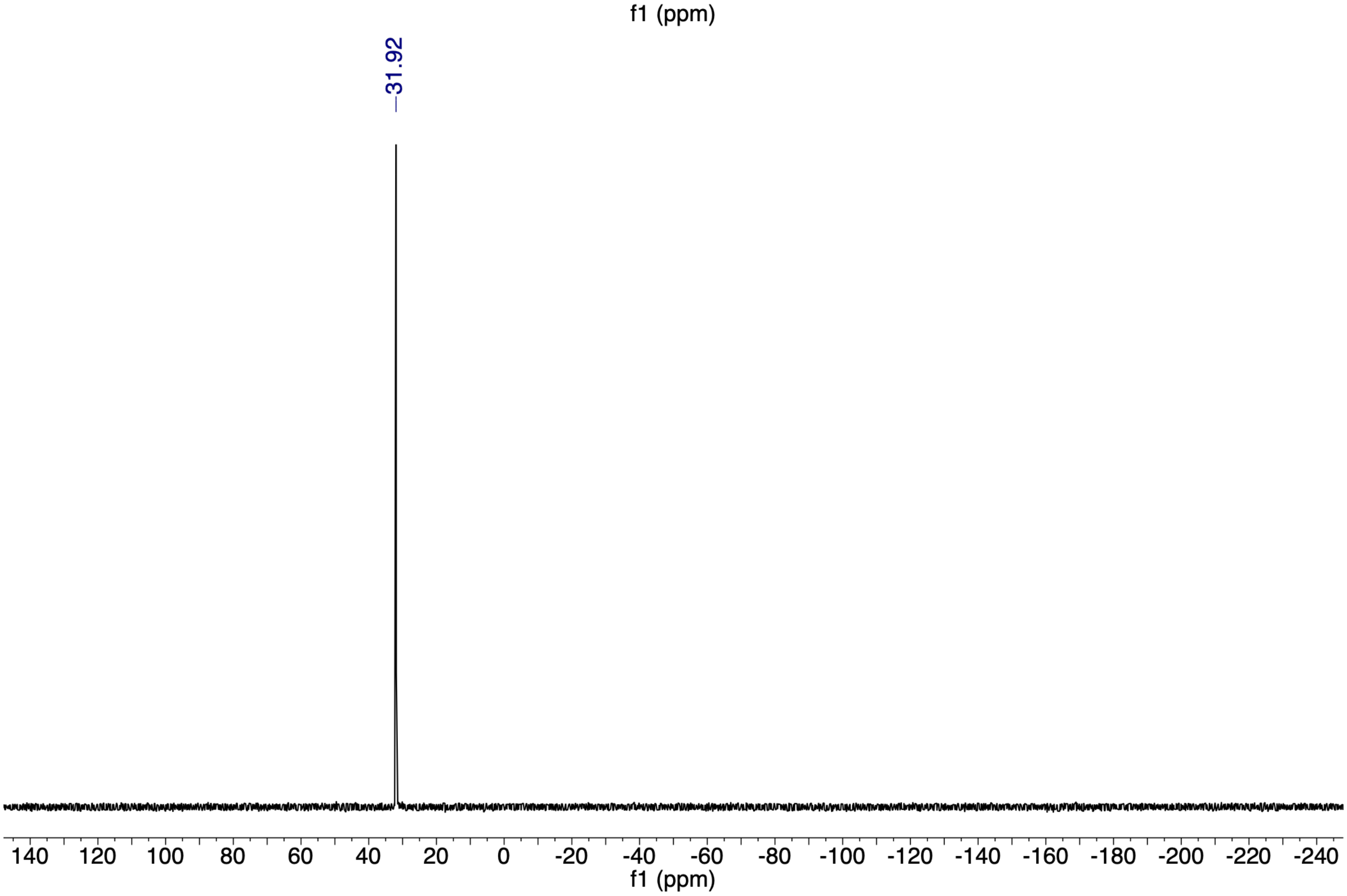

(Methyl-phenyl-phosphinoyl)-acetic acid methyl ester (1c)

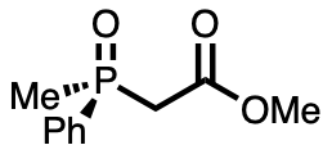

(+/–)

Synthesized using the outlined procedure with methyl diazo acetate instead of ethyl diazo acetate.

MW 212.18

^{1}H NMR (400 MHz, $CDCl_3$) δ 7.74 (ddd, J = 12.1, 8.3, 1.5 Hz, 2H), 7.62 – 7.42 (m, 3H), 3.65 (s, 3H), 3.29 – 2.92 (m, 2H), 1.93 (d, J = 13.5 Hz, 3H).

^{13}C NMR (101 MHz, $CDCl_3$) δ 167.23 (d, J = 4.6 Hz), 132.56 (d, J = 101.2 Hz), 132.48 (d, J = 2.8 Hz), 130.20 (d, J = 9.8 Hz), 128.92 (d, J = 12.1 Hz), 52.66, 40.42 (d, J = 58.8 Hz), 16.03 (d, J = 72.9 Hz).

^{31}P NMR (162 MHz, $CDCl_3$) δ 31.87.

HRMS (AccuTOF-FD) m/z: M+ calcd for C10H13O3P: 212.0597; found 212.0593.

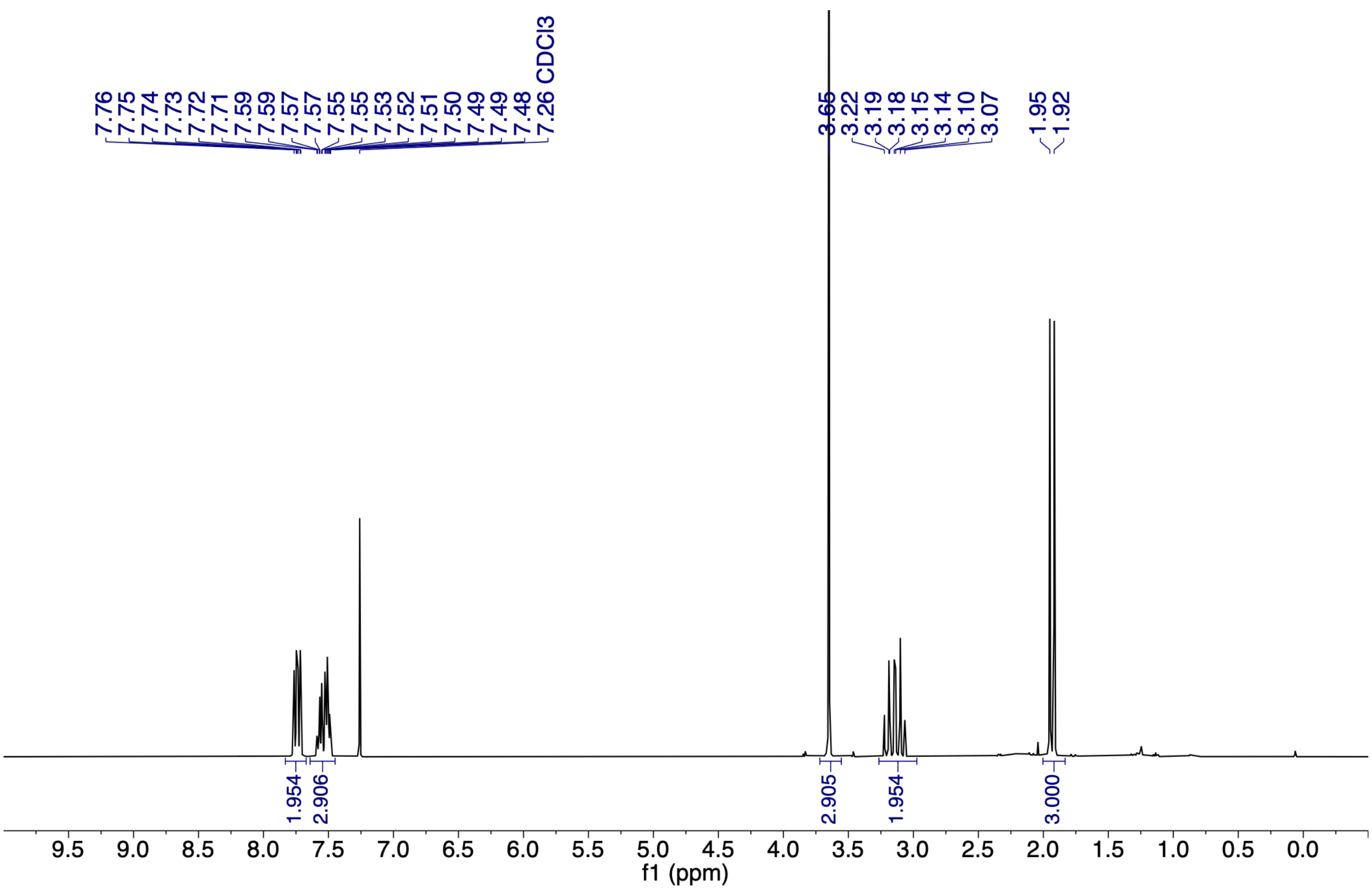

(Methyl-phenyl-phosphinoyl)-acetic acid methyl ester (1c)

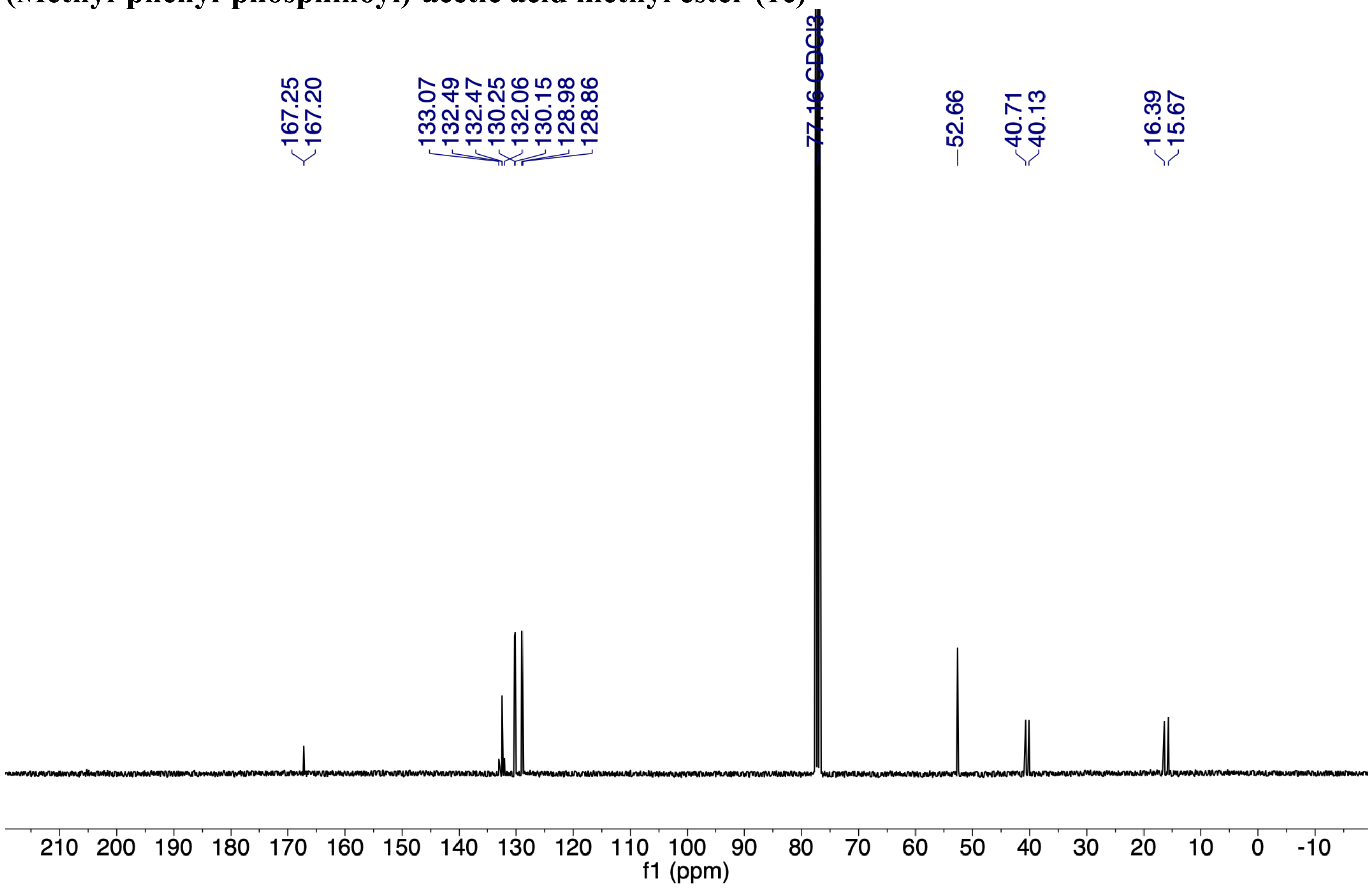

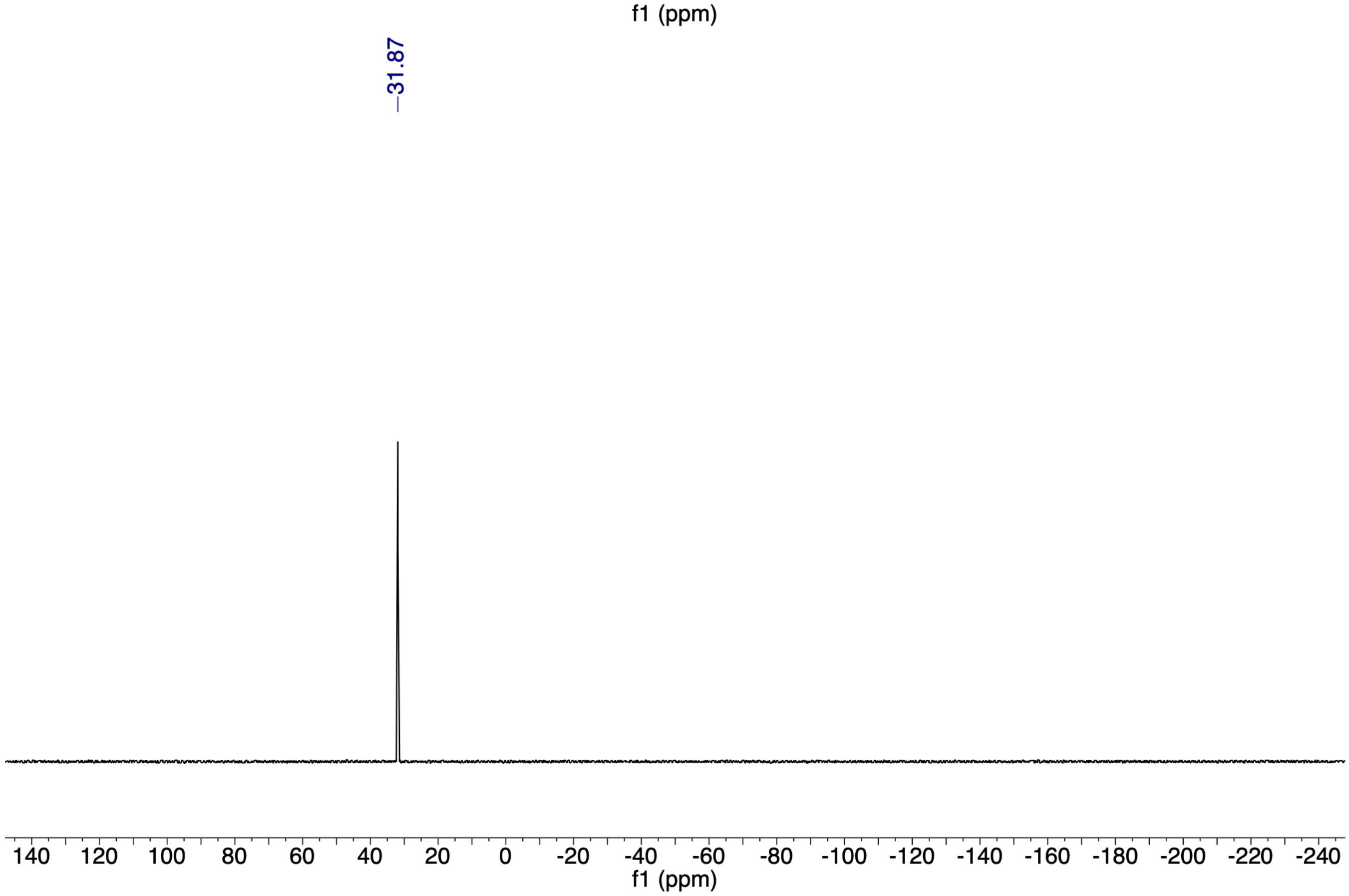

(Benzyl-phenyl-phosphinoyl)-acetic acid ethyl ester (3b)

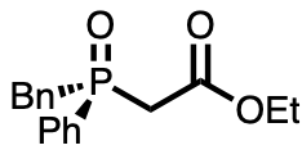

(+/–)

MW 302.31

^{1}H NMR (400 MHz, $CDCl_3$) δ 7.71 – 7.61 (m, 2H), 7.57 – 7.50 (m, 1H), 7.50 – 7.42 (m, 2H), 7.32 – 7.19 (m, 5H), 4.09 (qd, *J* = 7.1, 3.9 Hz, 2H), 3.70 – 3.46 (m, 2H), 3.18 – 3.01 (m, 2H), 1.14 (t, *J* = 7.1 Hz, 3H).

^{13}C NMR (101 MHz, $CDCl_3$) δ 166.87 (d, *J* = 3.7 Hz), 132.40 (d, *J* = 2.9 Hz), 130.98 (d, *J* = 8.1 Hz), 130.97 (d, *J* = 98.0 Hz), 130.94 (d, *J* = 9.0 Hz), 130.33 (d, *J* = 5.4 Hz), 128.82 (d, *J* = 2.9 Hz), 128.63 (d, *J* = 12.0 Hz), 127.28 (d, *J* = 3.3 Hz), 61.74, 37.67 (d, *J* = 65.7 Hz), 37.24 (d, *J* = 58.8 Hz), 14.10.

^{31}P NMR (162 MHz, $CDCl_3$) δ 32.19.

HRMS (AccuTOF-FD) m/z: M+ calcd for C17H19O3P: 302.1066; found 302.1073.

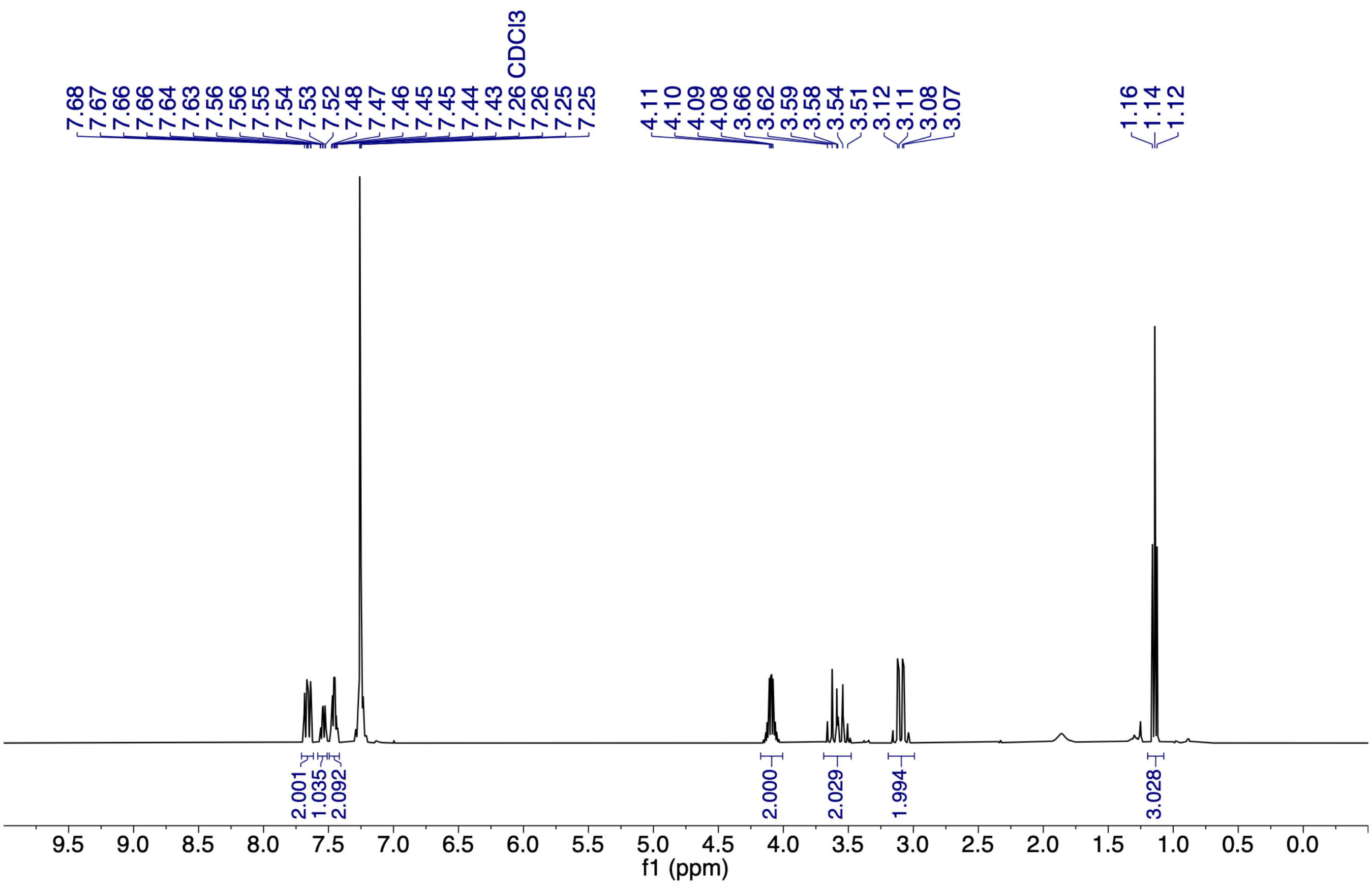

(Benzyl-phenyl-phosphinoyl)-acetic acid ethyl ester (3b)

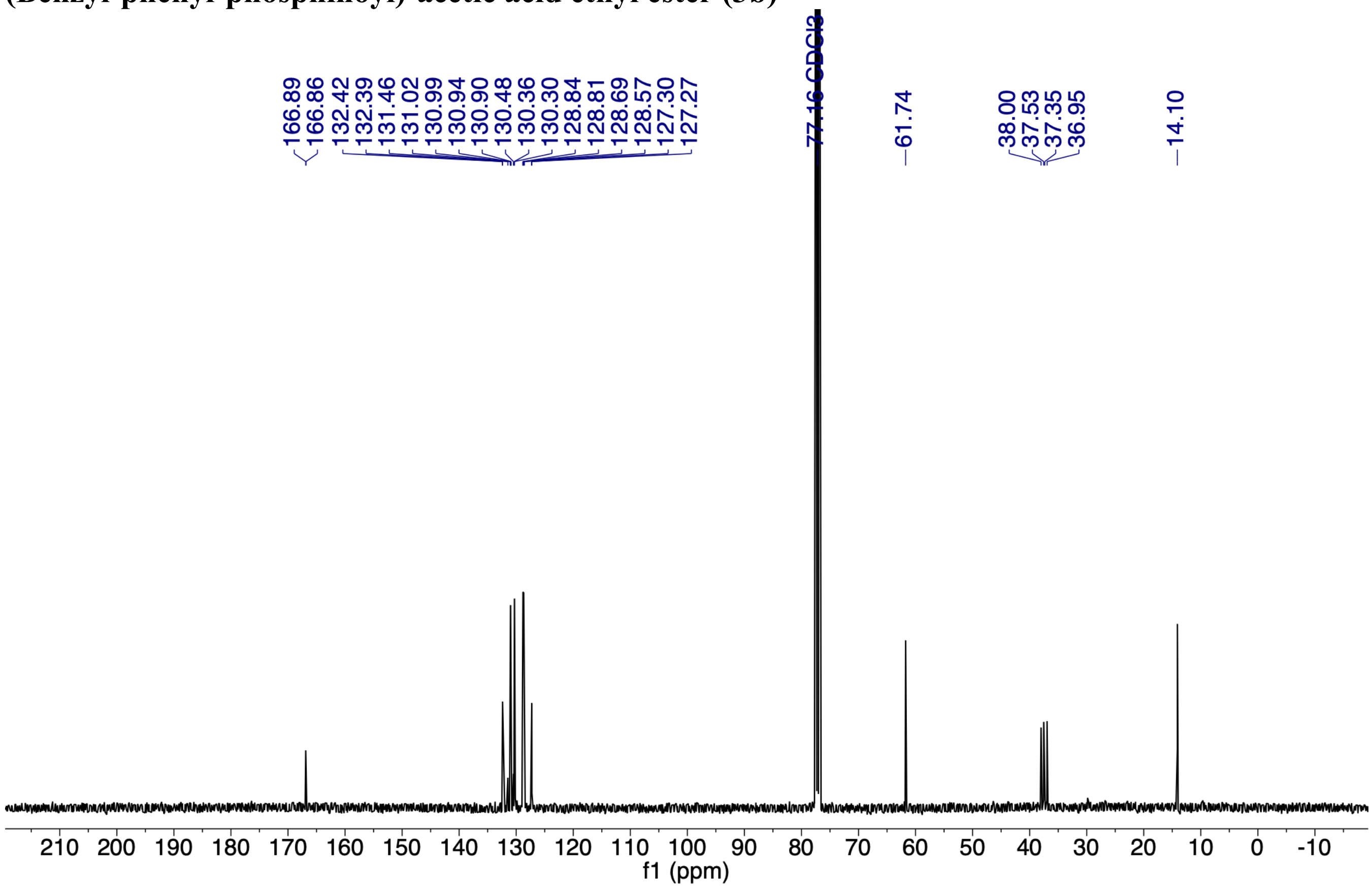

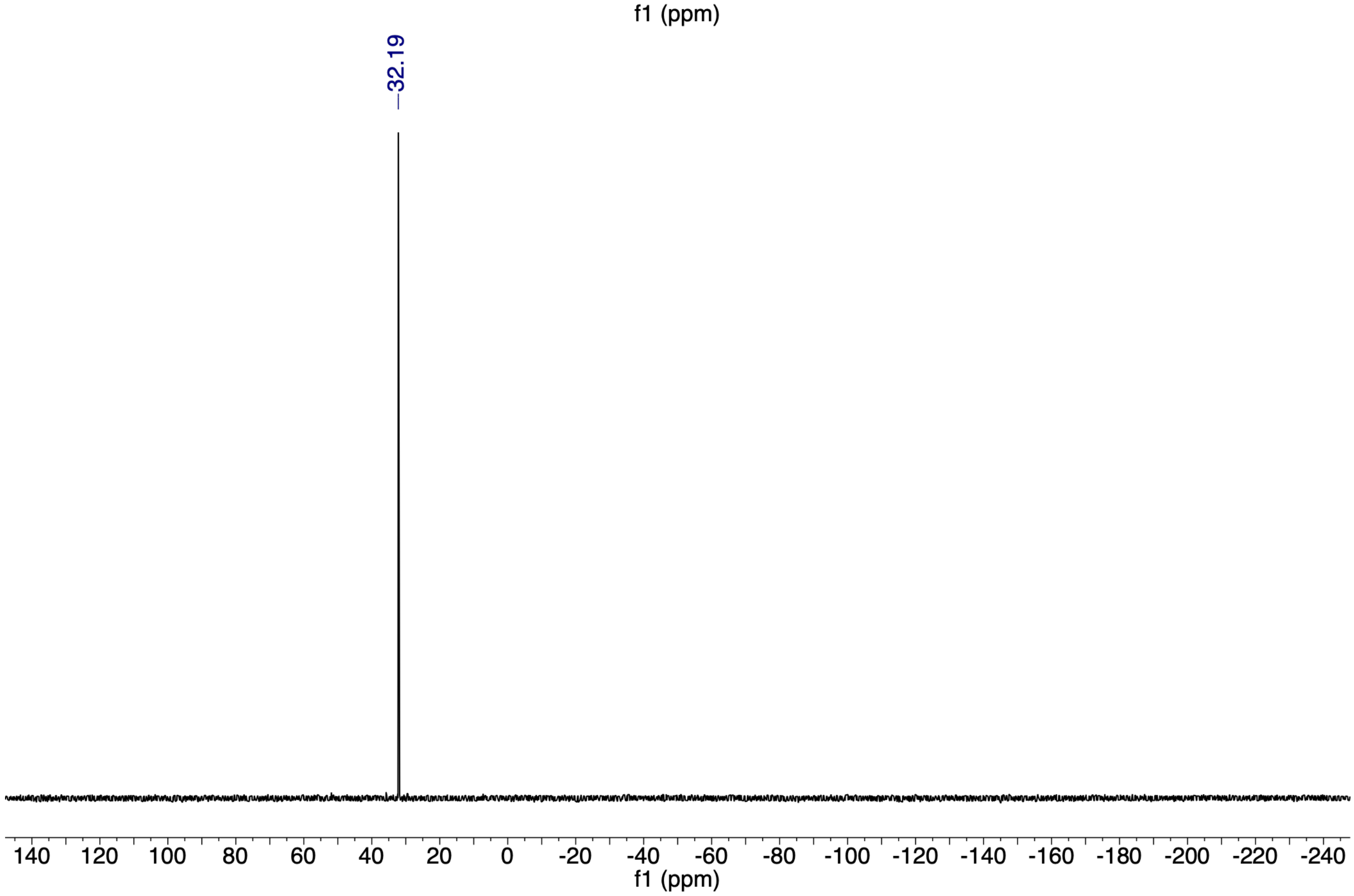

(Isopropyl-phenyl-phosphinoyl)-acetic acid ethyl ester (4b)

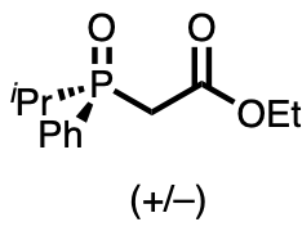

MW 254.27

^{1}H NMR (400 MHz, $CDCl_3$) δ 7.80 – 7.70 (m, 2H), 7.62 – 7.55 (m, 1H), 7.54 – 7.46 (m, 2H), 4.16 – 3.99 (m, 2H), 3.36 – 3.19 (m, 2H), 2.52 – 2.37 (m, J = 7.2 Hz, 1H), 1.36 (dd, J = 17.0, 7.1 Hz, 3H), 1.12 (t, J = 7.1 Hz, 3H), 1.10 (dd, J = 17.6, 7.2 Hz, 3H).

^{13}C NMR (101 MHz, $CDCl_3$) δ 166.52 (d, J = 3.5 Hz), 132.62 (d, J = 2.8 Hz), 131.14 (d, J = 9.0 Hz), 129.01 (d, J = 95.4 Hz), 128.84 (d, J = 11.7 Hz), 61.82, 36.13 (d, J = 55.4 Hz), 27.55 (d, J = 70.8 Hz), 15.28 (d, J = 2.6 Hz), 14.98 (d, J = 3.4 Hz), 14.05.

^{31}P NMR (162 MHz, $CDCl_3$) δ 44.69.

HRMS (AccuTOF-FD) m/z: M+ calcd for C13H19O3P: 254.1066; found 254.1069.

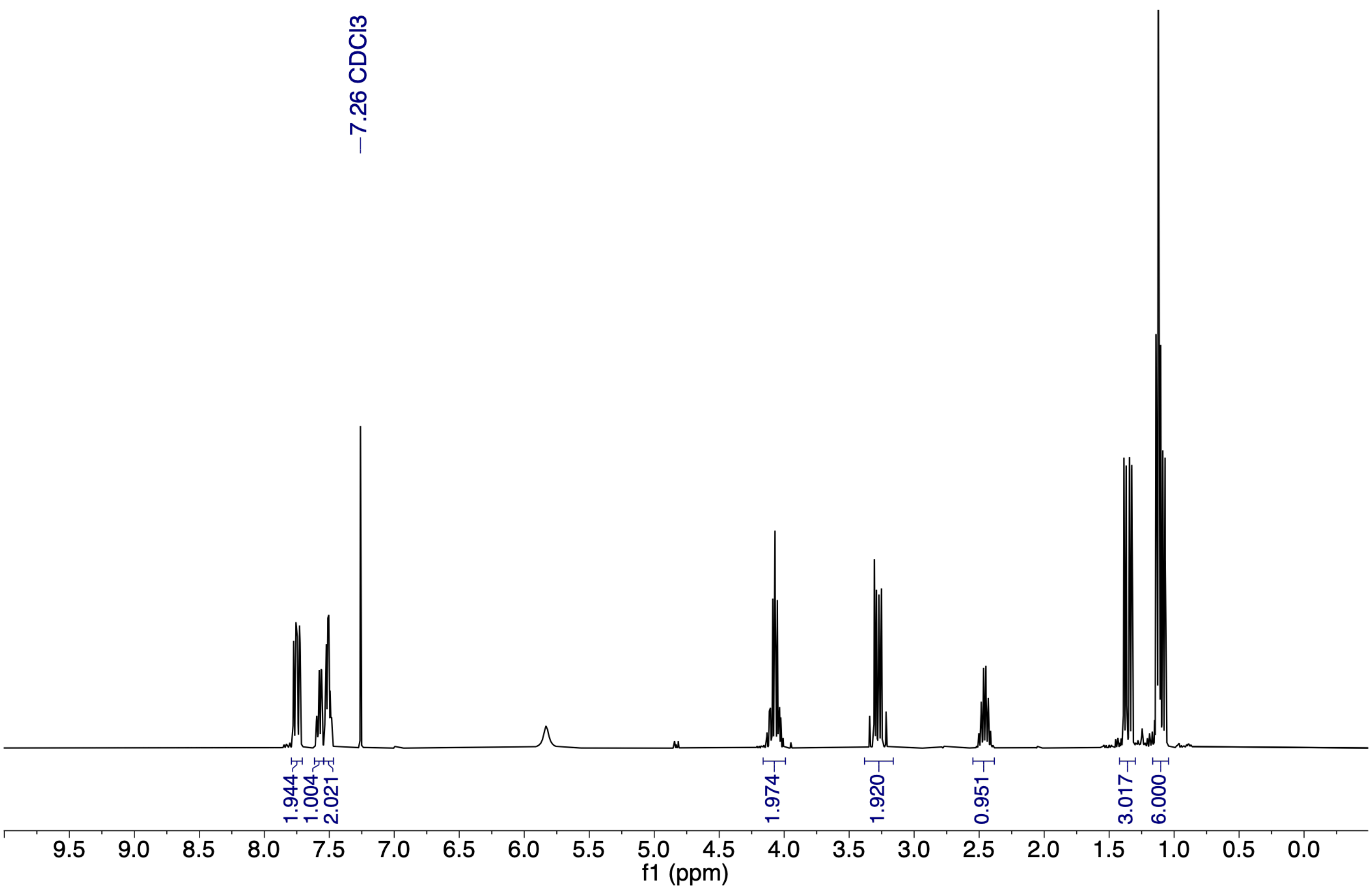

(Isopropyl-phenyl-phosphinoyl)-acetic acid ethyl ester (4b)

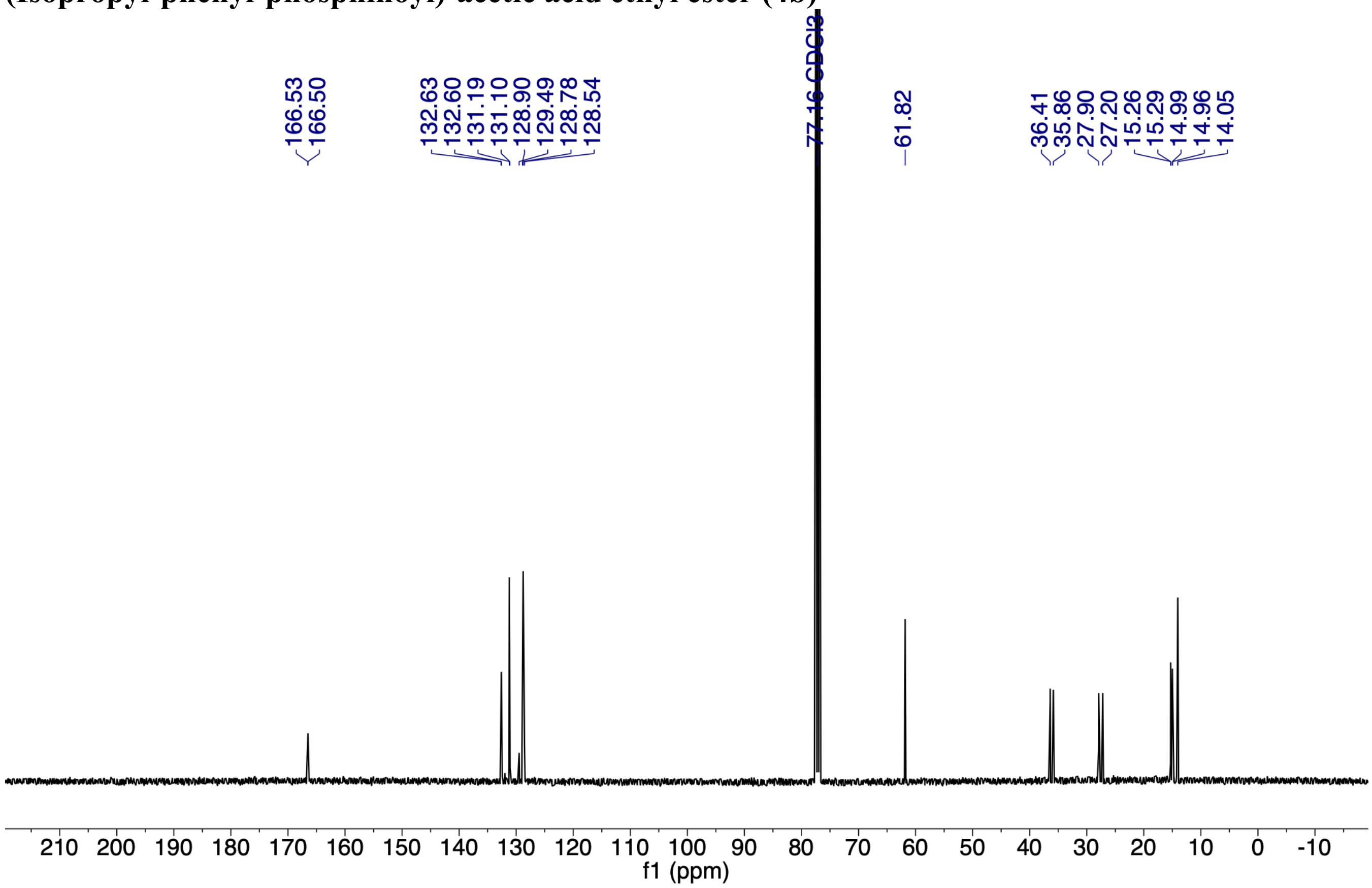

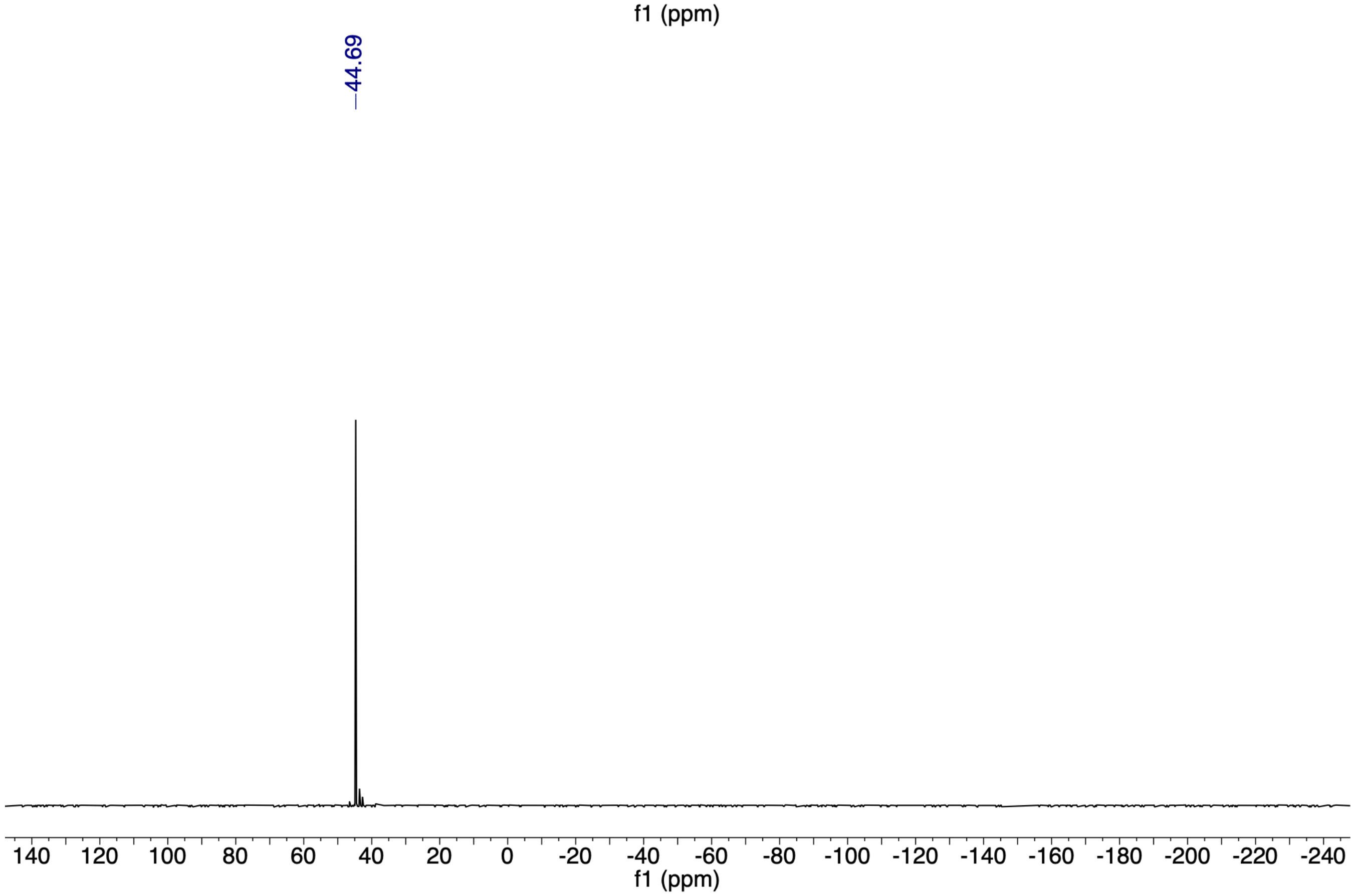

(Cyclohexyl-phenyl-phosphinoyl)-acetic acid ethyl ester (5b)

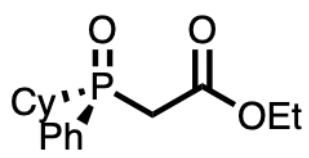

(+/–)

MW 294.33

^{1}H NMR (400 MHz, $CDCl_3$) δ 7.77 – 7.67 (m, 2H), 7.58 – 7.52 (m, 1H), 7.49 (dddd, *J* = 9.9, 4.4, 2.5, 1.2 Hz, 2H), 4.14 – 3.92 (m, 2H), 3.32 – 2.98 (m, 2H), 2.17 – 2.05 (m, 2H), 1.94 – 1.85 (m, 1H), 1.83 – 1.46 (m, 4H), 1.45 – 1.16 (m, 4H), 1.12 (t, *J* = 7.1 Hz, 3H).

^{13}C NMR (101 MHz, $CDCl_3$) δ 166.96 (d, *J* = 3.9 Hz), 132.12 (d, *J* = 2.6 Hz), 131.08 (d, *J* = 8.8 Hz), 130.43 (d, *J* = 94.0 Hz), 128.64 (d, *J* = 11.5 Hz), 61.60, 37.83 (d, *J* = 71.6 Hz), 36.38 (d, *J* = 54.3 Hz), 26.37 (d, *J* = 2.2 Hz), 26.32 – 26.13 (m), 25.88 – 25.68 (m), 24.88 (d, *J* = 2.8 Hz), 24.67 (d, *J* = 3.4 Hz), 14.09.

^{31}P NMR (162 MHz, $CDCl_3$) δ 37.88.

HRMS (AccuTOF-FD) m/z: M+ calcd for C16H23O3P: 294.1379; found 294.1385.

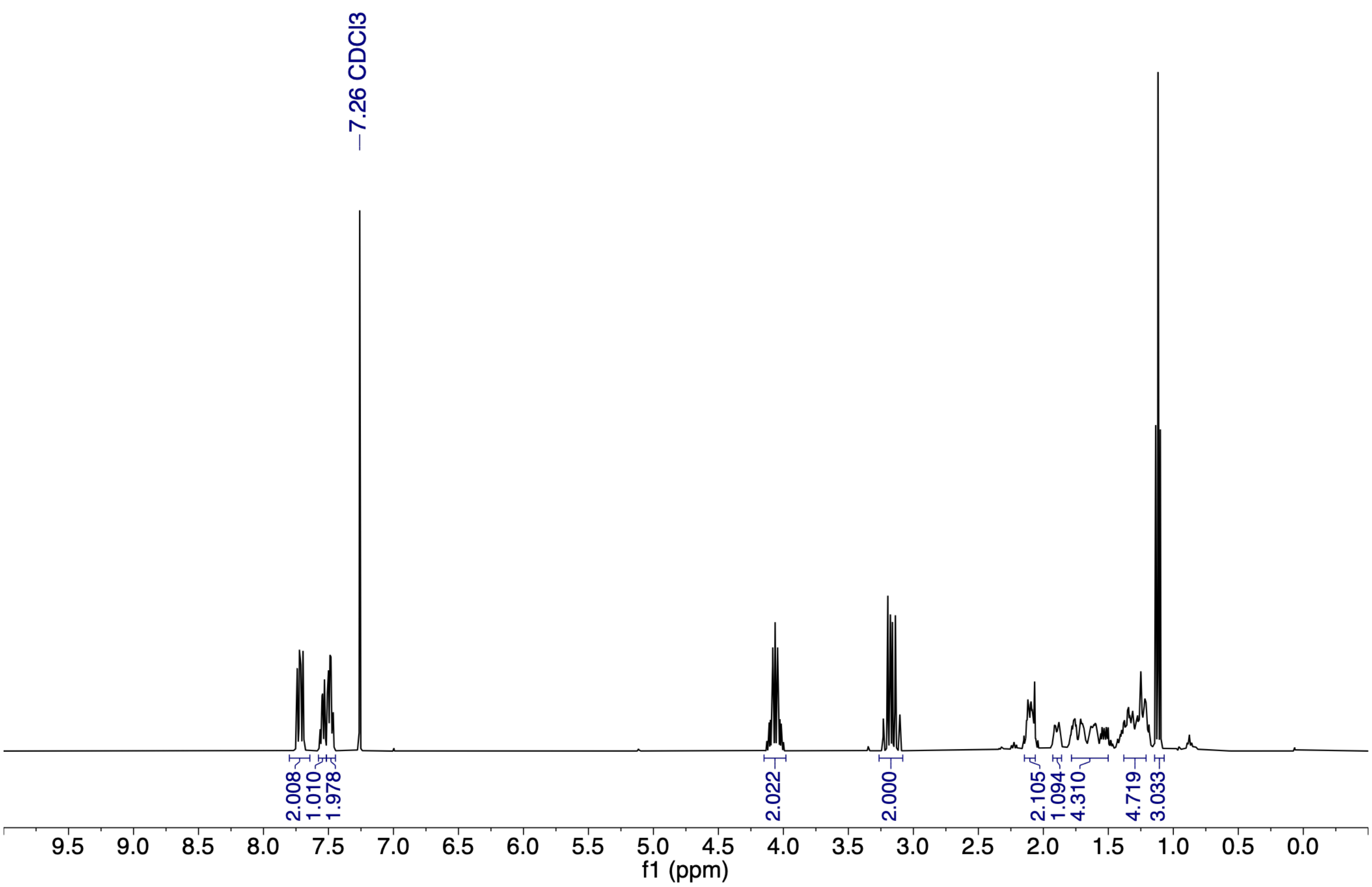

(Cyclohexyl-phenyl-phosphinoyl)-acetic acid ethyl ester (5b)

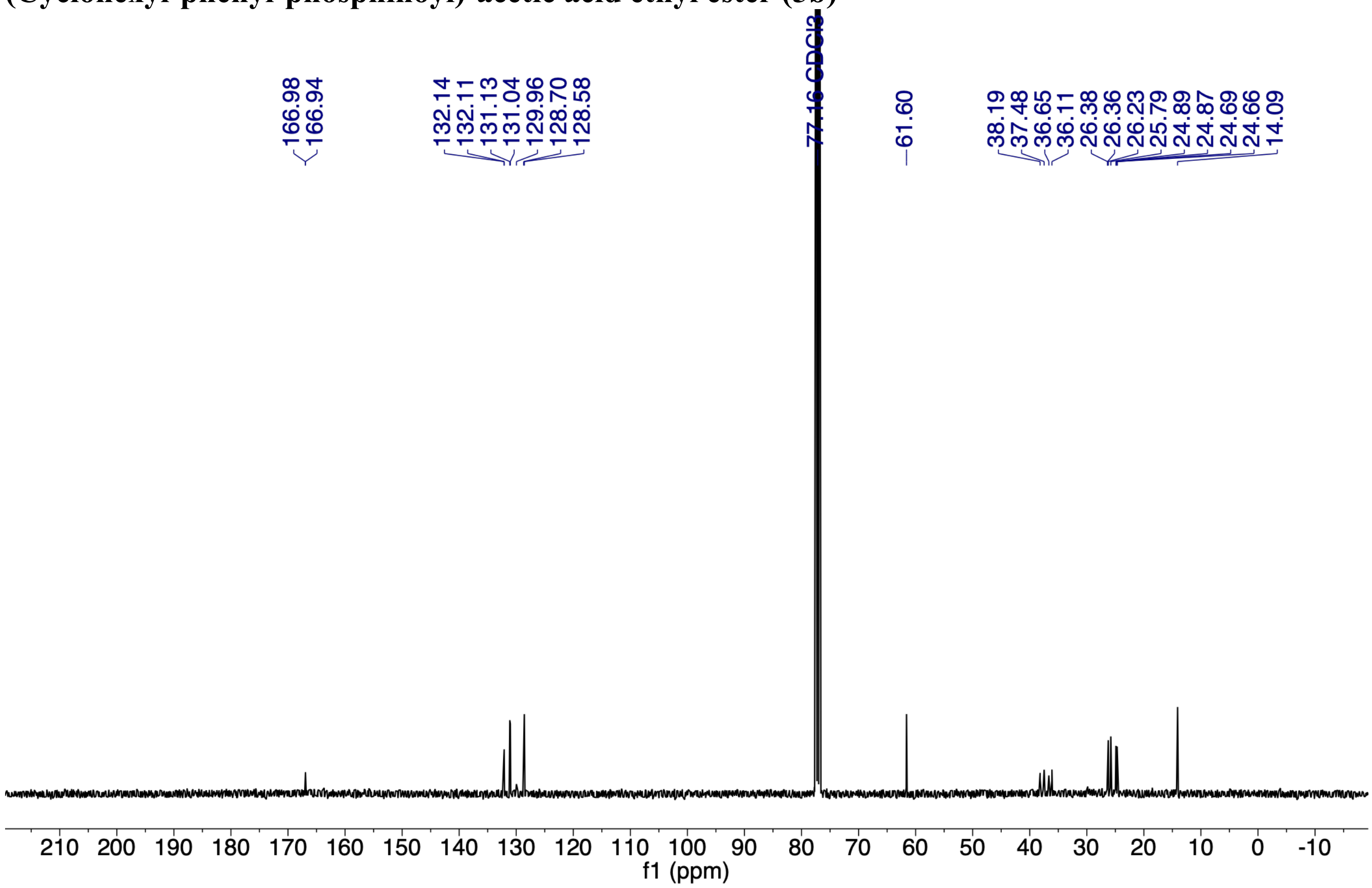

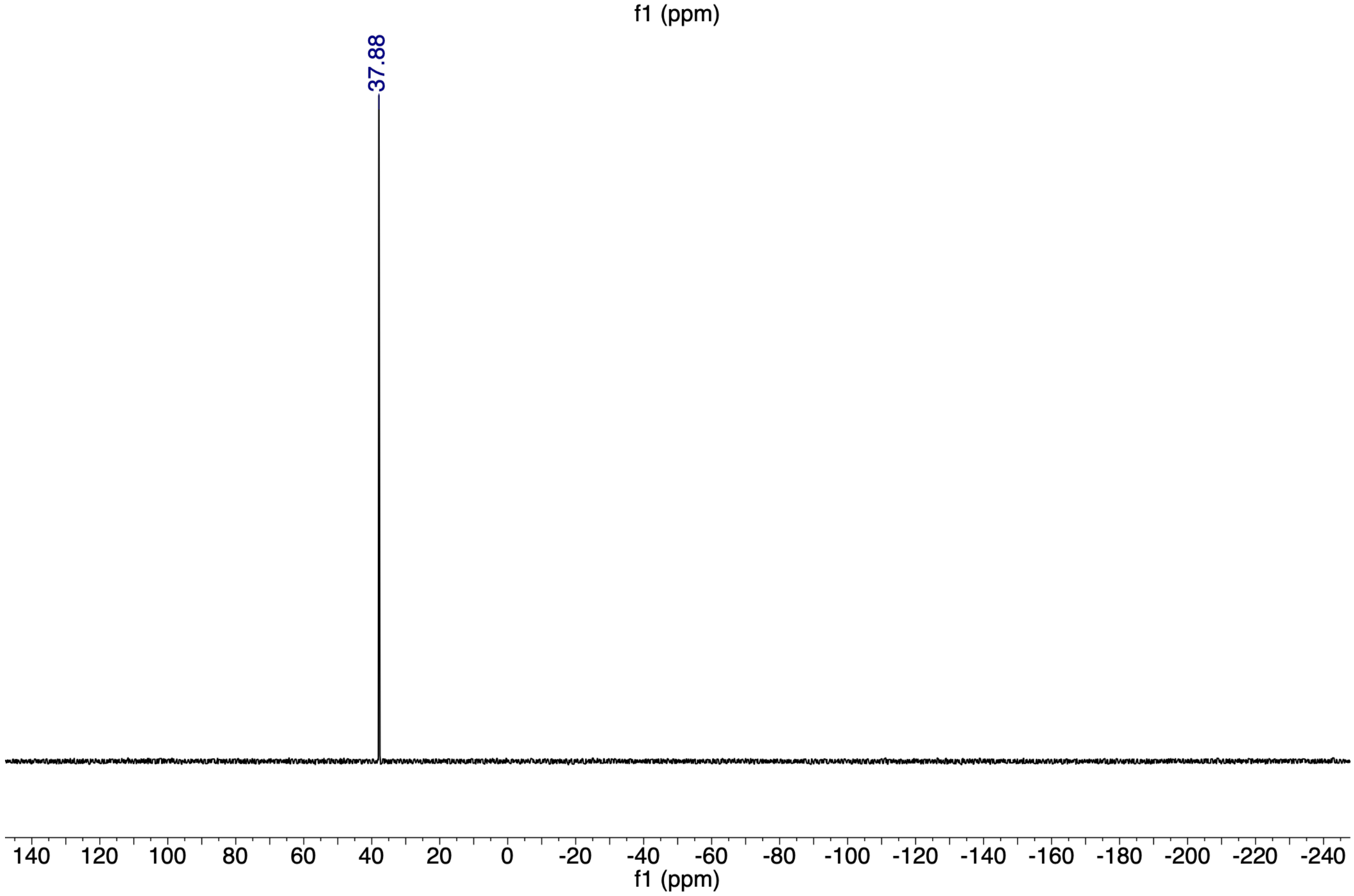

(Para-fluorophenyl-phenyl-phosphinoyl)-acetic acid ethyl ester (6b)

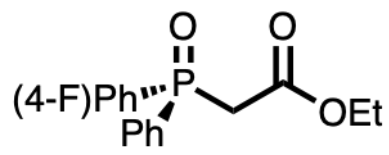

(+/–)

MW 306.27

^{1}H NMR (400 MHz, $CDCl_3$) δ 7.88 – 7.71 (m, 4H), 7.57 (td, *J* = 7.3, 1.5 Hz, 1H), 7.50 (tt, *J* = 7.0, 3.1 Hz, 2H), 7.18 (td, *J* = 8.7, 2.2 Hz, 2H), 4.01 (q, *J* = 7.1 Hz, 2H), 3.48 (d, *J* = 15.1 Hz, 2H), 1.05 (t, *J* = 7.1 Hz, 3H).

^{13}C NMR (101 MHz, $CDCl_3$) δ 166.20 (d, *J* = 5.1 Hz), 165.37 (dd, *J* = 254.4, 3.2 Hz), 133.93 (dd, *J* = 11.4, 8.8 Hz), 132.57 (d, *J* = 2.8 Hz), 131.72 (d, *J* = 107.8 Hz), 131.18 (d, *J* = 9.9 Hz), 128.87 (d, *J* = 12.4 Hz), 127.79 (d, *J* = 106.6 Hz), 116.18 (dd, *J* = 21.5, 13.5 Hz), 61.80, 39.44 (d, *J* = 61.2 Hz), 13.99.

^{19}F NMR (376 MHz, $CDCl_3$) δ -106.03 (d, *J* = 1.4 Hz, 1F).

^{31}P NMR (162 MHz, $CDCl_3$) δ 26.31.

HRMS (AccuTOF-FD) m/z: M+ calcd for C16H16FO3P: 306.0816; found 306.0817.

(Para-fluorophenyl-phenyl-phosphinoyl)-acetic acid ethyl ester (6b)

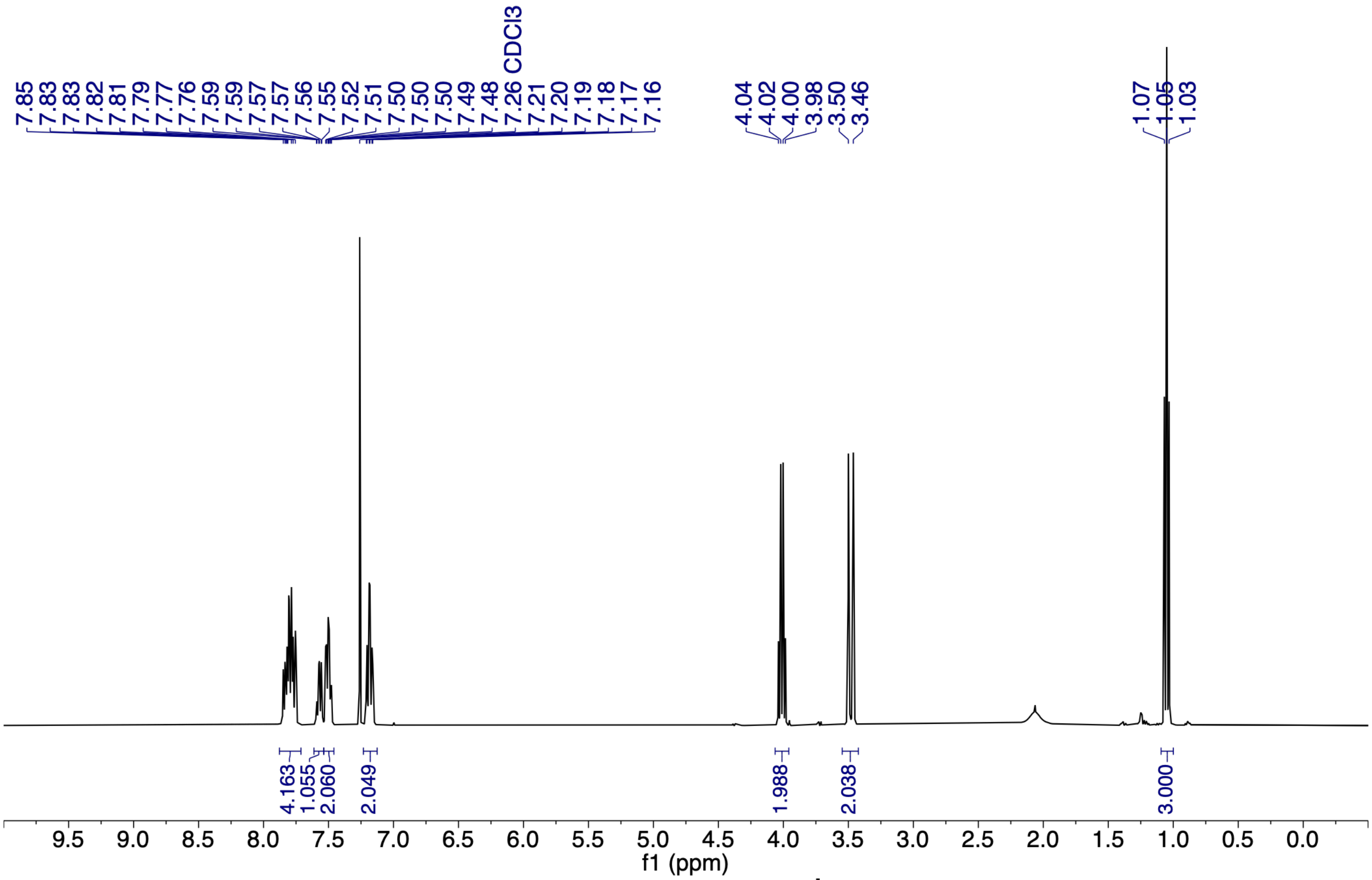

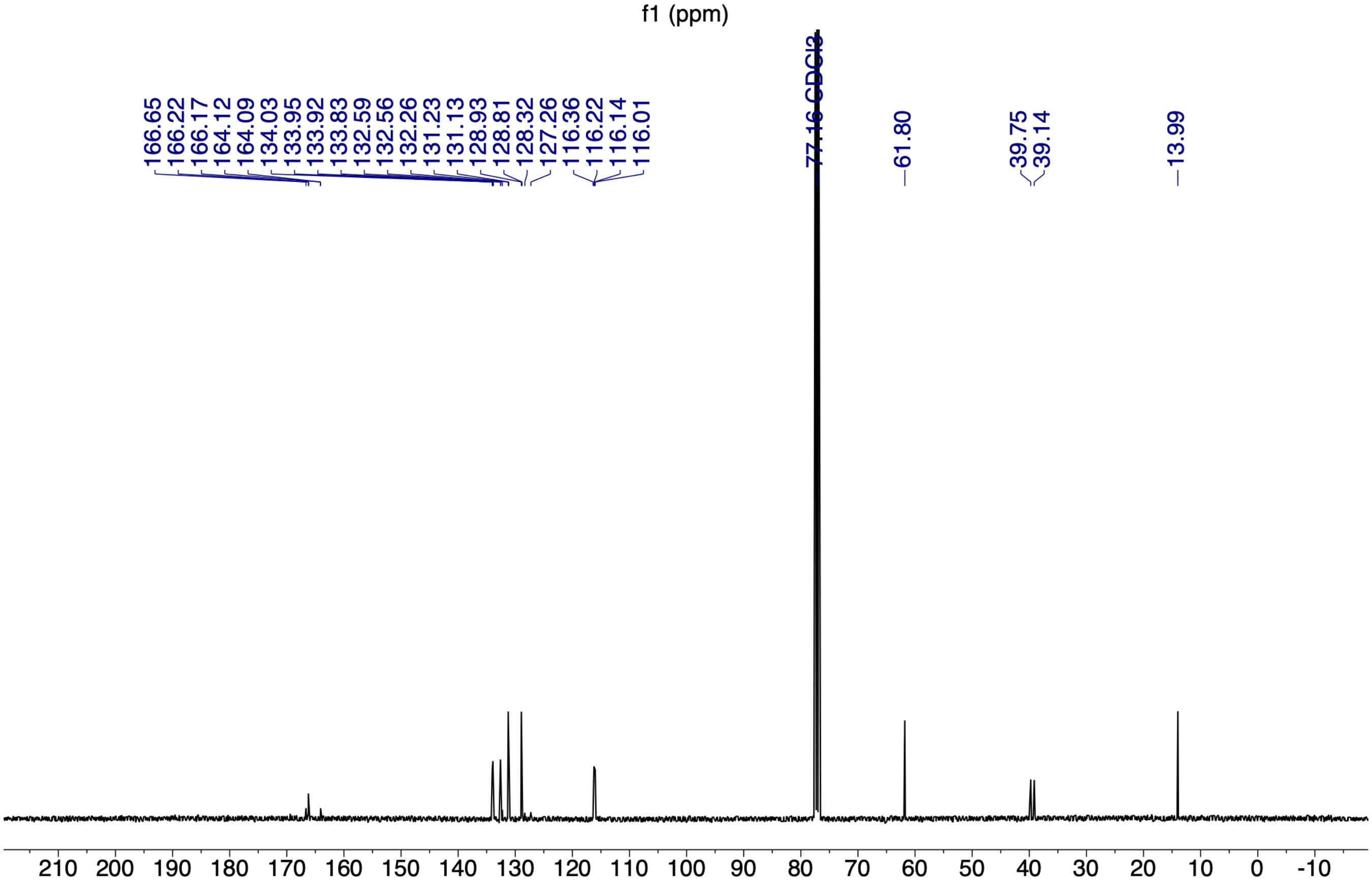

(Para-fluorophenyl-phenyl-phosphinoyl)-acetic acid ethyl ester (6b)

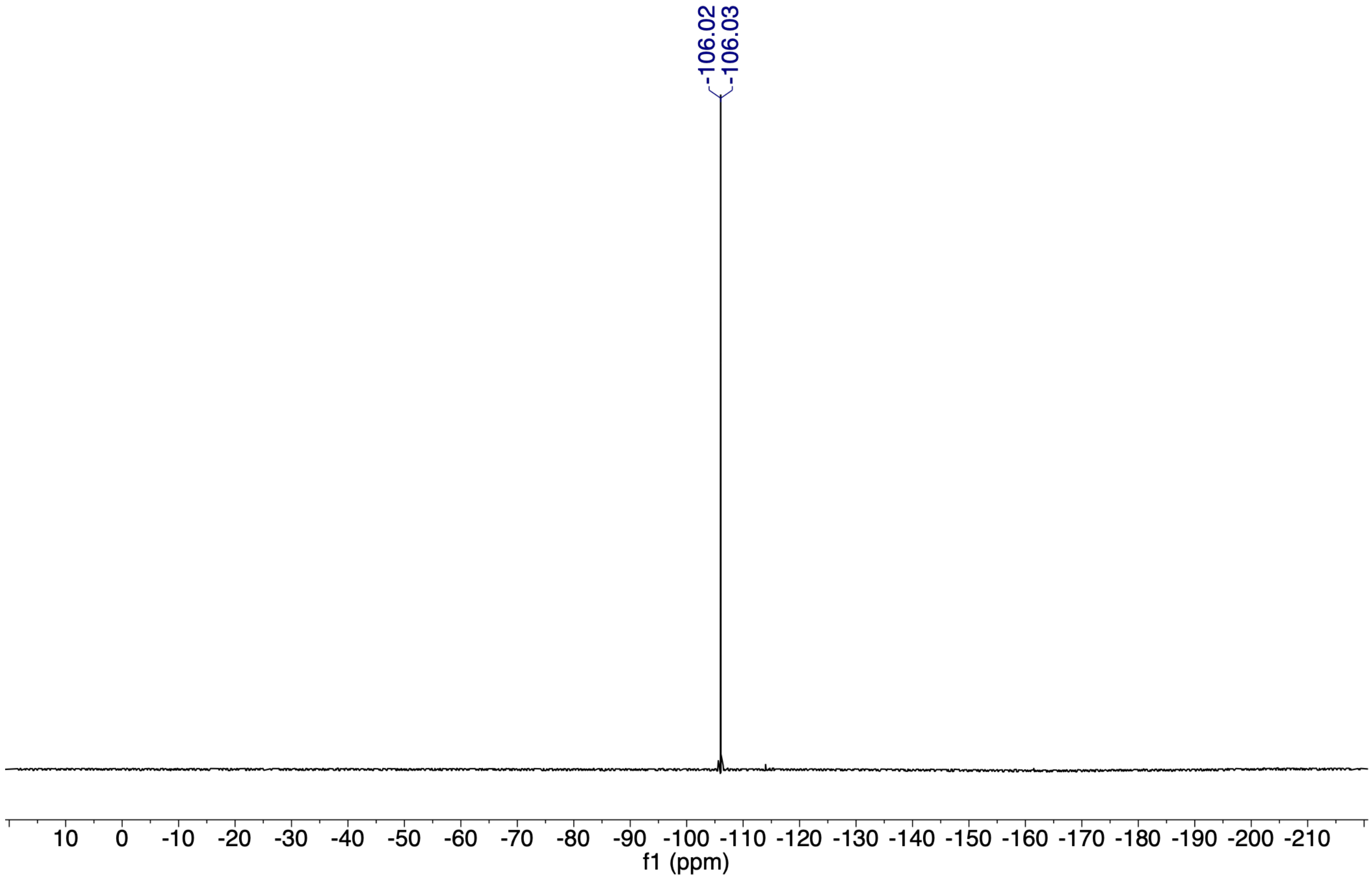

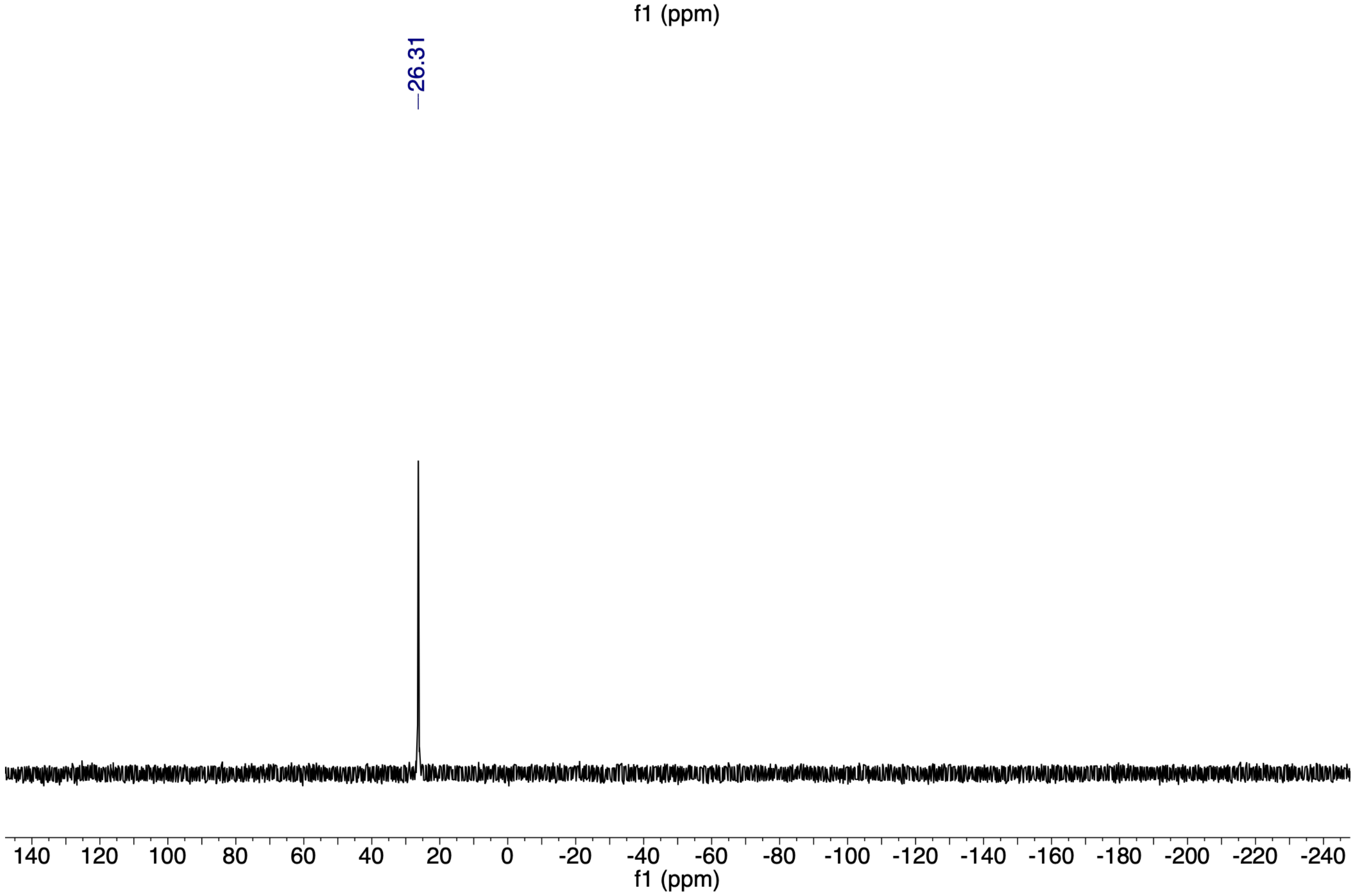

(Ortho-anisol-phenyl-phosphinoyl)-acetic acid ethyl ester (7b)

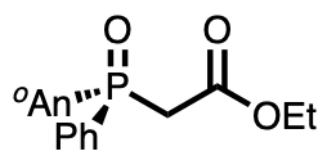

(+/–)

MW 318.31

^{1}H NMR (400 MHz, $CDCl_3$) δ 7.97 (ddd, *J* = 13.6, 7.6, 1.8 Hz, 1H), 7.89 – 7.77 (m, 2H), 7.64 – 7.45 (m, 2H), 7.48 – 7.38 (m, 2H), 7.11 (td, *J* = 7.5, 1.1 Hz, 1H), 6.97 – 6.88 (m, 1H), 4.04 (q, *J* = 7.1 Hz, 2H), 3.80 (s, 3H), 3.61 (d, *J* = 14.9 Hz, 2H), 1.03 (t, *J* = 7.1 Hz, 3H).

^{13}C NMR (101 MHz, $CDCl_3$) δ 166.67 (d, *J* = 6.3 Hz), 159.92 (d, *J* = 4.1 Hz), 134.64 (d, *J* = 5.9 Hz), 134.48 (d, *J* = 1.9 Hz), 133.15 (d, *J* = 106.4 Hz), 131.90 (d, *J* = 2.6 Hz), 130.90 (d, *J* = 10.3 Hz), 128.40 (d, *J* = 12.6 Hz), 121.35 (d, *J* = 11.6 Hz), 119.69 (d, *J* = 103.5 Hz), 110.92 (d, *J* = 6.8 Hz), 61.41, 55.54, 38.60 (d, *J* = 63.3 Hz), 14.00.

^{31}P NMR (162 MHz, $CDCl_3$) δ 25.33.

HRMS (AccuTOF-FD) m/z: M+ calcd for C17H19O4P: 318.1016; found 318.1025.

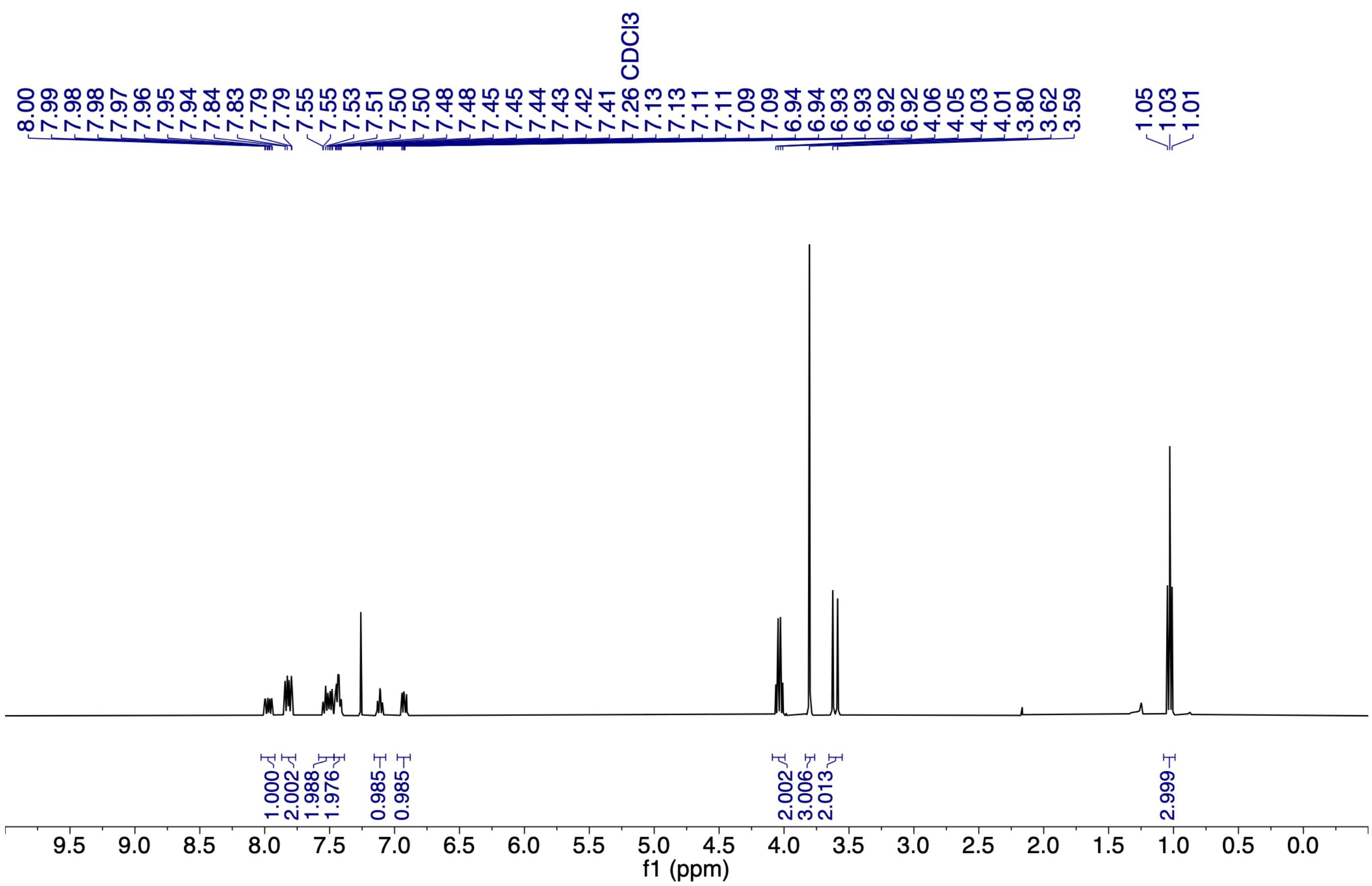

(Ortho-anisol-phenyl-phosphinoyl)-acetic acid ethyl ester (7b)

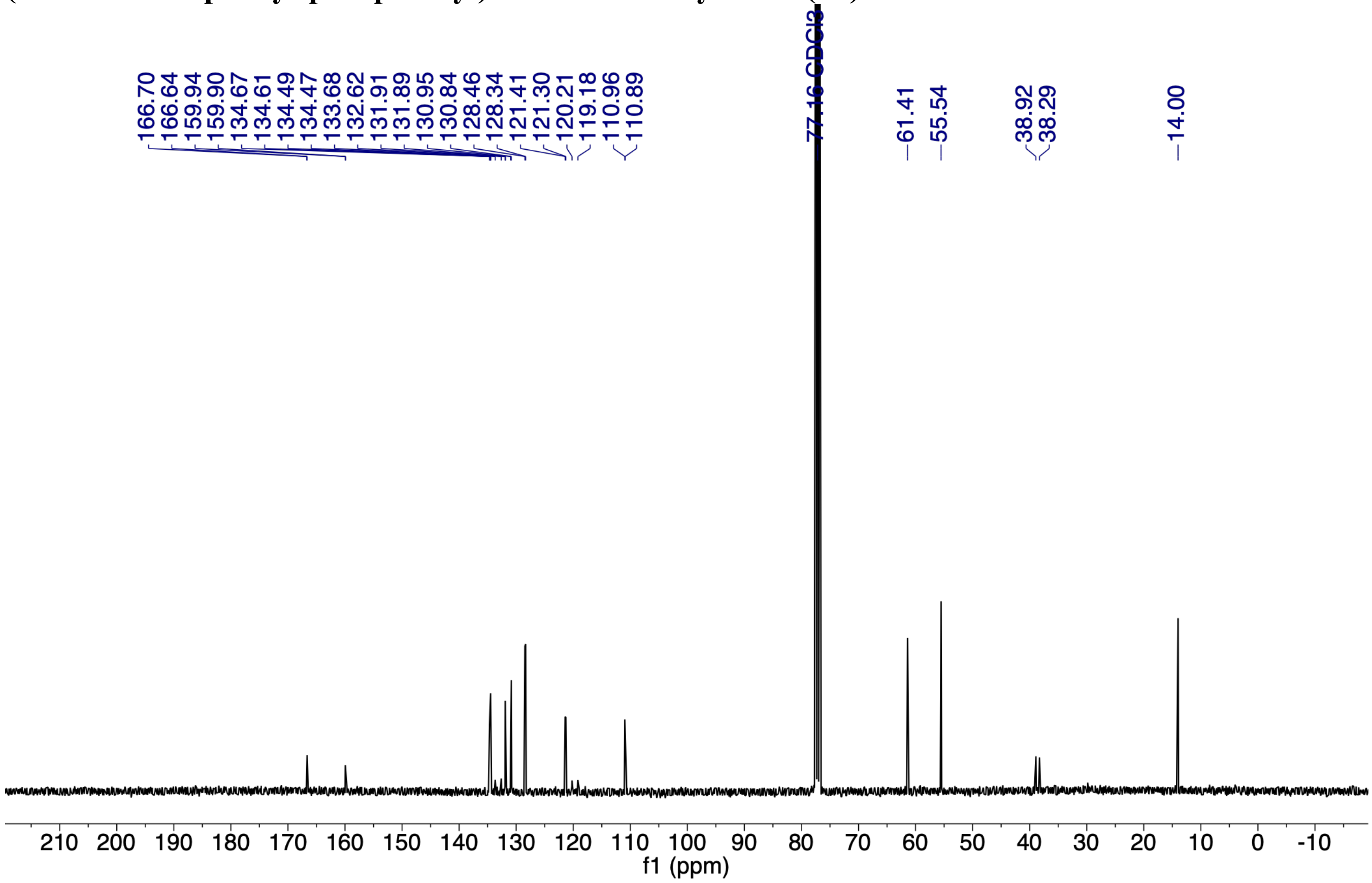

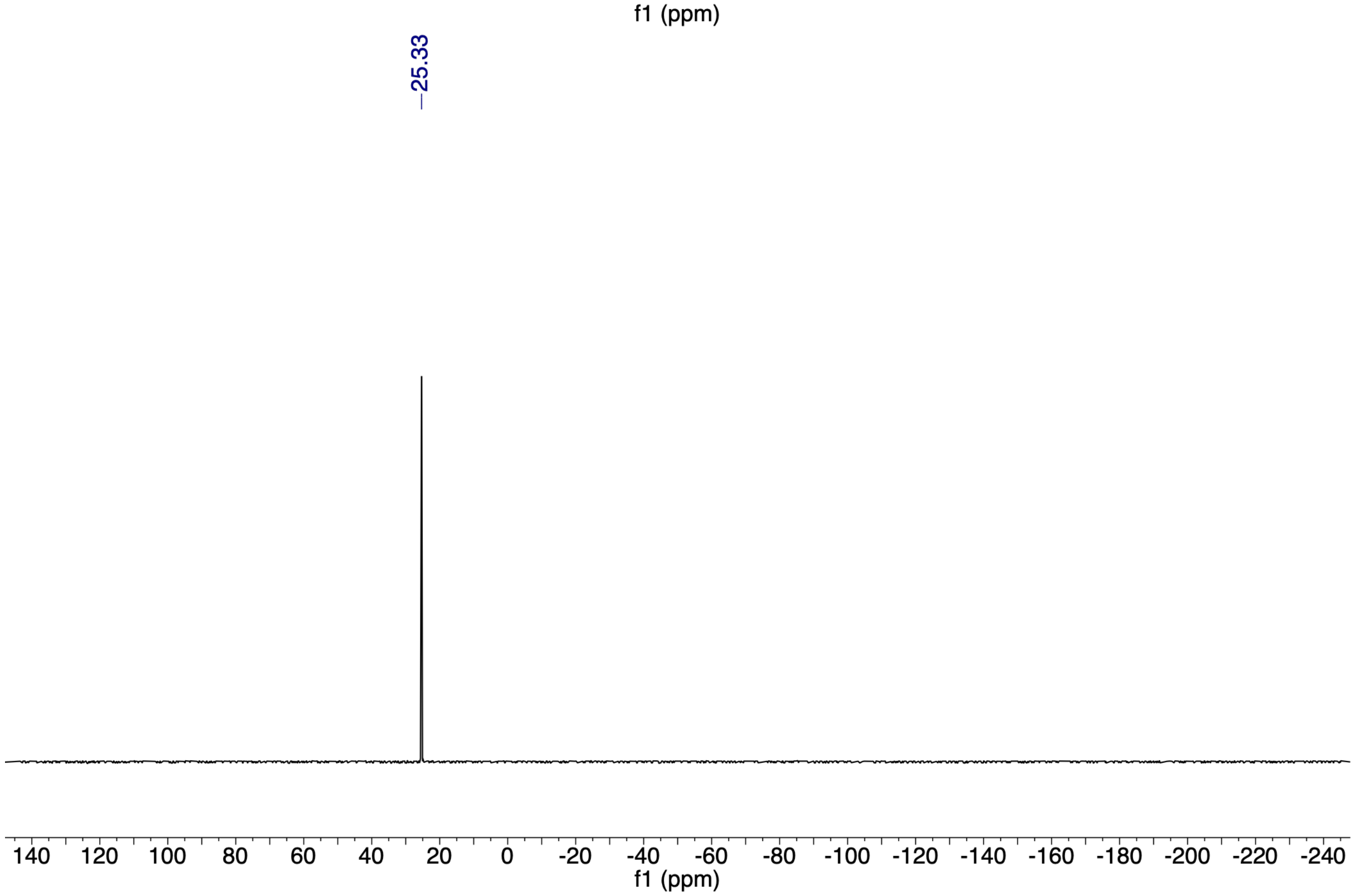

(2-napthyl-phenyl-phosphinoyl)-acetic acid ethyl ester (8b)

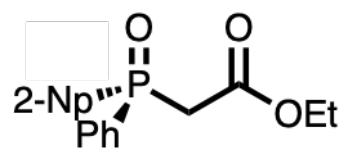

(+/–)

MW 338.34

^{1}H NMR (400 MHz, $CDCl_3$) δ 8.45 (dd, *J* = 14.4, 1.6 Hz, 1H), 7.93 (dd, *J* = 8.3, 2.4 Hz, 2H), 7.91 – 7.78 (m, 3H), 7.72 (ddd, *J* = 10.0, 8.4, 1.5 Hz, 1H), 7.65 – 7.52 (m, 3H), 7.49 (ddd, *J* = 6.8, 5.7, 2.4 Hz, 2H), 4.00 (q, *J* = 7.1 Hz, 2H), 3.58 (d, *J* = 14.8 Hz, 2H), 1.00 (t, *J* = 7.1 Hz, 3H).

^{13}C NMR (101 MHz, $CDCl_3$) δ 166.30 (d, *J* = 5.4 Hz), 134.94 (d, *J* = 2.6 Hz), 133.51 (d, *J* = 9.0 Hz), 132.56 (d, *J* = 13.4 Hz), 132.43 (d, *J* = 2.9 Hz), 132.06 (d, *J* = 104.3 Hz), 131.28 (d, *J* = 10.0 Hz), 129.14, 128.82 (d, *J* = 104.4 Hz), 128.81 (d, *J* = 12.4 Hz), 128.59 (d, *J* = 12.3 Hz), 128.56, 127.98, 127.23, 125.85 (d, *J* = 11.0 Hz), 61.73, 39.31 (d, *J* = 60.7 Hz), 13.95. [insufficient number of aromatic carbon peaks due to overlap]

^{31}P NMR (162 MHz, $CDCl_3$) δ 27.09.

HRMS (AccuTOF-FD) m/z: M+ calcd for C20H19O3P: 338.1066; found 338.1064.

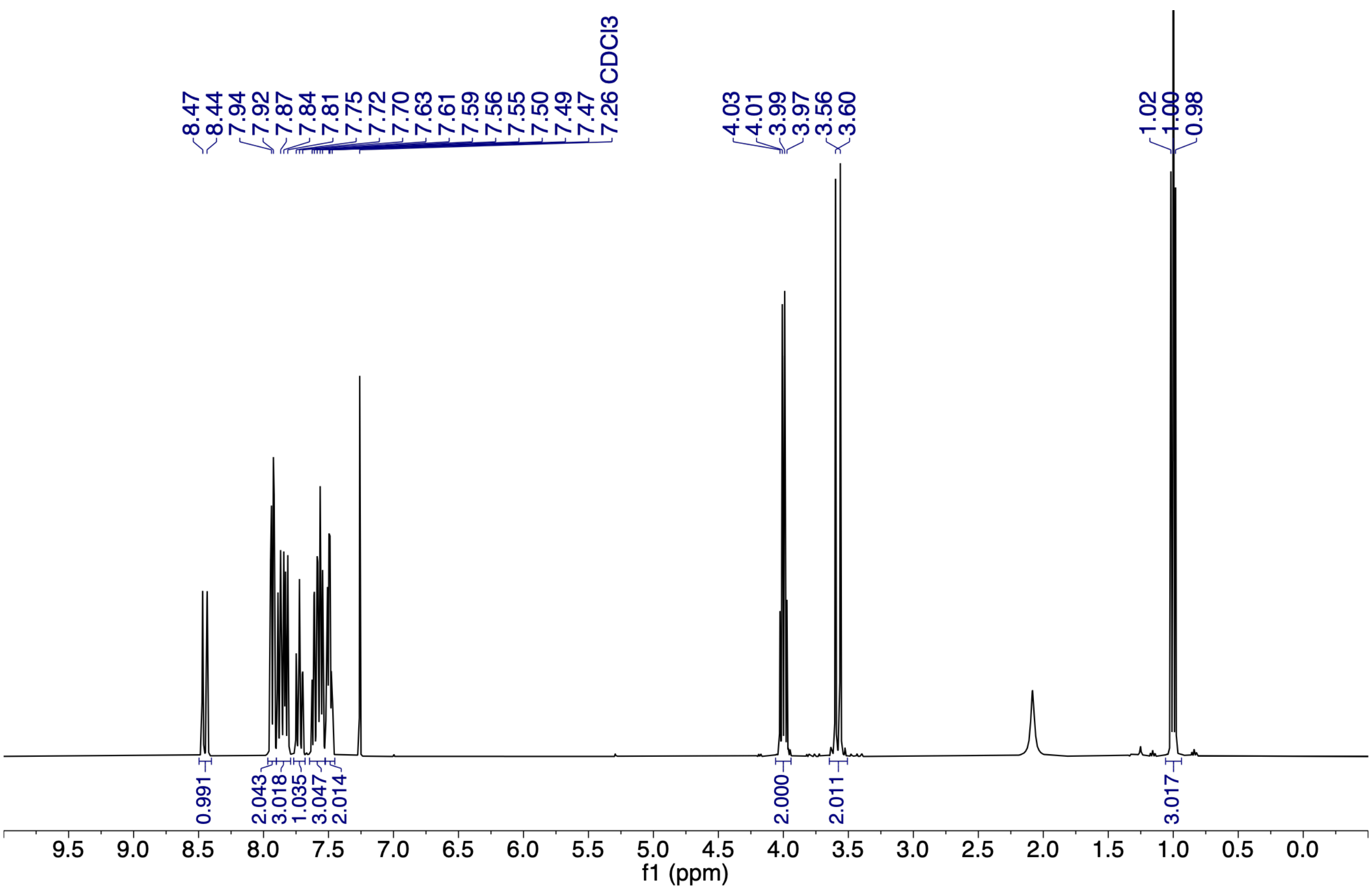

(2-napthyl-phenyl-phosphinoyl)-acetic acid ethyl ester (8b)

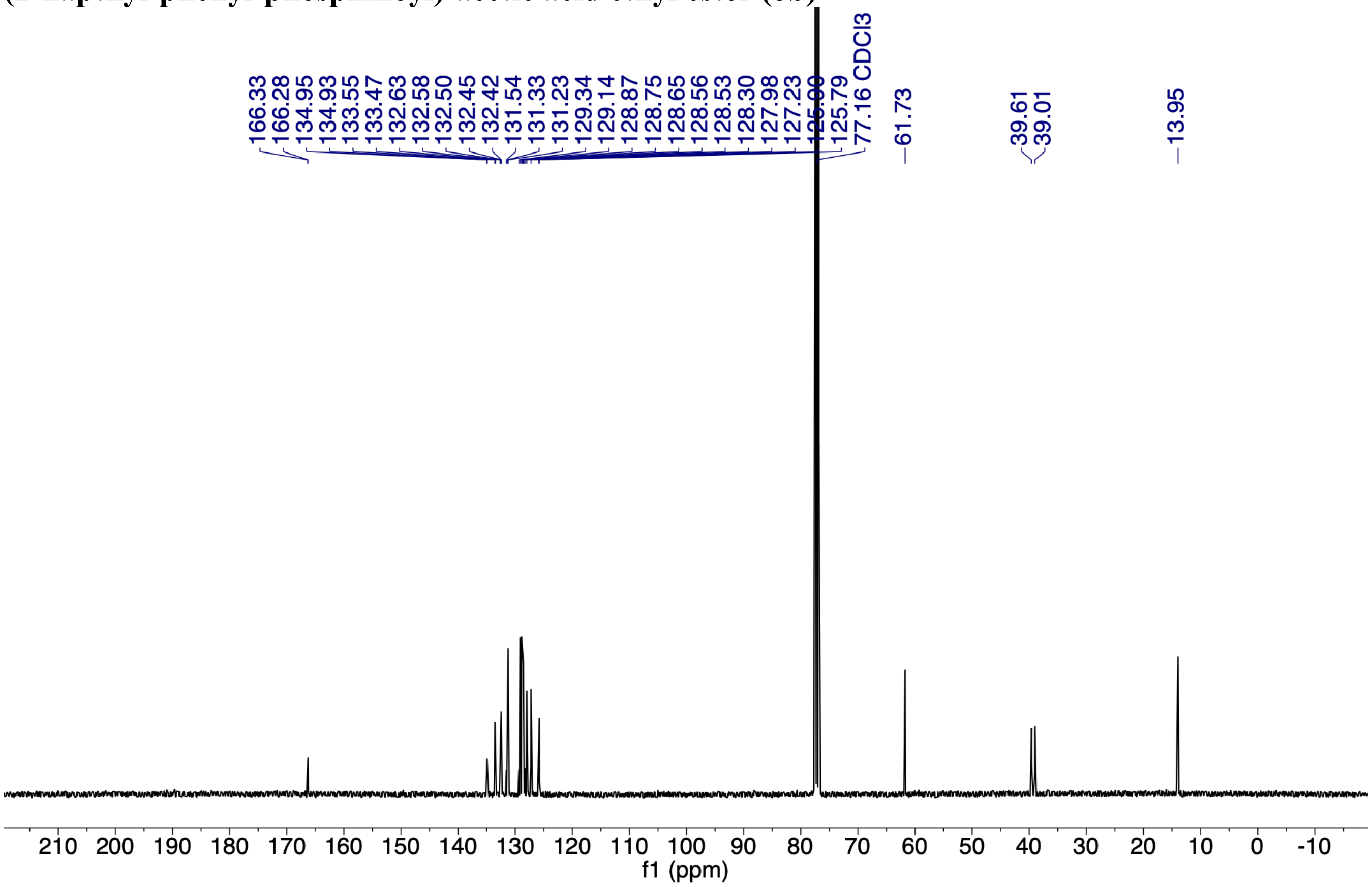

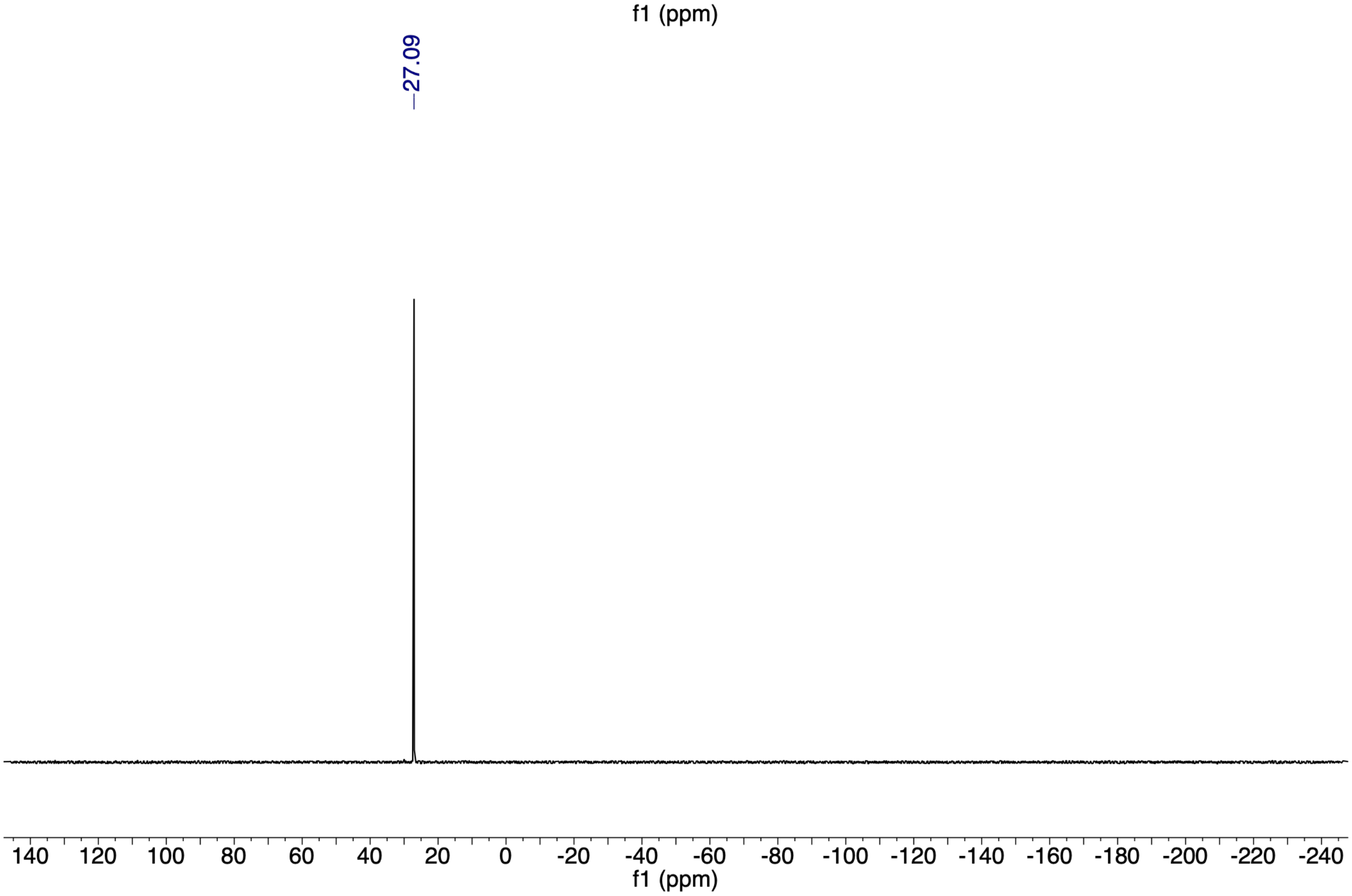

3.4 Phosphinate product standards (carbene adducts)

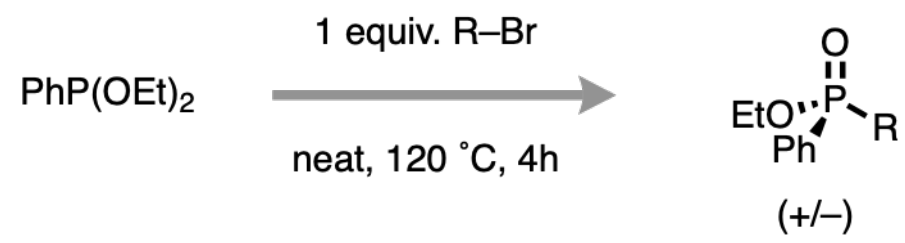

Authentic, racemic standards for the C–P bond formation between ethyl phenylphosphinate and carbene precursors were prepared with the Michaelis-Arbuzov reaction, according to a previously reported procedure.[15] An oven dried 2-dram vial equipped with a stir bar was charged with diethyl phenylphosphonite (1 equiv, 0.5 mmol, 96 µL) and the corresponding alkyl bromide (1 equiv, 0.5 mmol). The vial was evacuated and backfilled with N_2 three times. The vial was capped with a PTFE lined cap and stirred for 4 h at 120 ˚C. The crude material was purified by silica gel chromatography (hexanes/ethyl acetate or ethyl acetate/methanol, if necessary) to afford the desired product (72-92% yield).

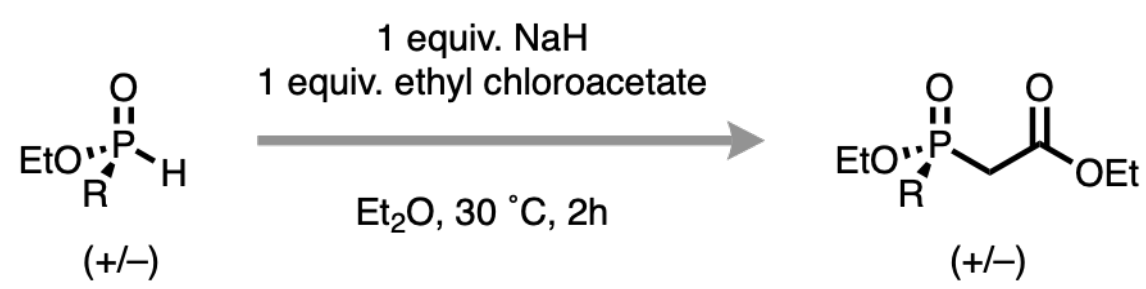

Authentic, racemic standards for the C–P bond formation between H-phosphinates and EDA were prepared according to a previously reported procedure.[16] An oven dried 2-dram vial equipped with a stir bar was charged with the corresponding H-phosphinate (1 equiv, 1 mmol). The vial was evacuated and backfilled with N_2 three times. To the vial was added Et_2O (2 mL, 0.5 M). Sodium hydride in oil dispersion, 60% (1 equiv, 1 mmol, 40 mg) was added in one portion. The reaction was stirred for 2 h at 30 ˚C. The reaction was cooled to 0 ˚C, and a solution of ethyl chloroacetate (1 equiv, 1 mmol, 107 µL) in Et_2O (1 mL) was added dropwise. The reaction was stirred for 1 h at 30 ˚C. The reaction was concentrated *in vacuo*, and the crude material was purified by silica gel chromatography (hexanes/ethyl acetate or ethyl acetate/methanol, if necessary) to afford the desired product (22-55% yield).

(Ethoxy-phenyl-phosphinoyl)-acetic acid ethyl ester (2b)

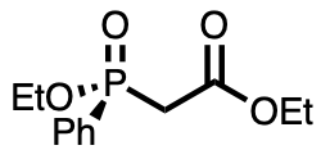

(+/–)

Synthesized with ethyl bromoacetate.

CAS 3699-71-6

MW 256.24

^{1}H NMR (400 MHz, $CDCl_3$) δ 7.90 – 7.73 (m, 2H), 7.64 – 7.52 (m, 1H), 7.52 – 7.38 (m, 2H), 4.25 – 3.95 (m, 4H), 3.12 (dd, *J* = 17.8, 1.9 Hz, 2H), 1.34 (t, *J* = 7.1 Hz, 3H), 1.13 (t, *J* = 7.1 Hz, 3H).

^{13}C NMR (101 MHz, $CDCl_3$) δ 166.00 (d, *J* = 4.8 Hz), 132.86 (d, *J* = 2.9 Hz), 131.88 (d, *J* = 10.2 Hz), 130.12 (d, *J* = 135.0 Hz), 128.70 (d, *J* = 13.3 Hz), 61.65 (d, *J* = 6.2 Hz), 61.54, 38.78 (d, *J* = 89.1 Hz), 16.53 (d, *J* = 6.6 Hz), 14.07.

^{31}P NMR (162 MHz, $CDCl_3$) δ 34.03.

HRMS (AccuTOF-FD) m/z: M+ calcd for C12H17O4P: 256.0859; found 256.0862.

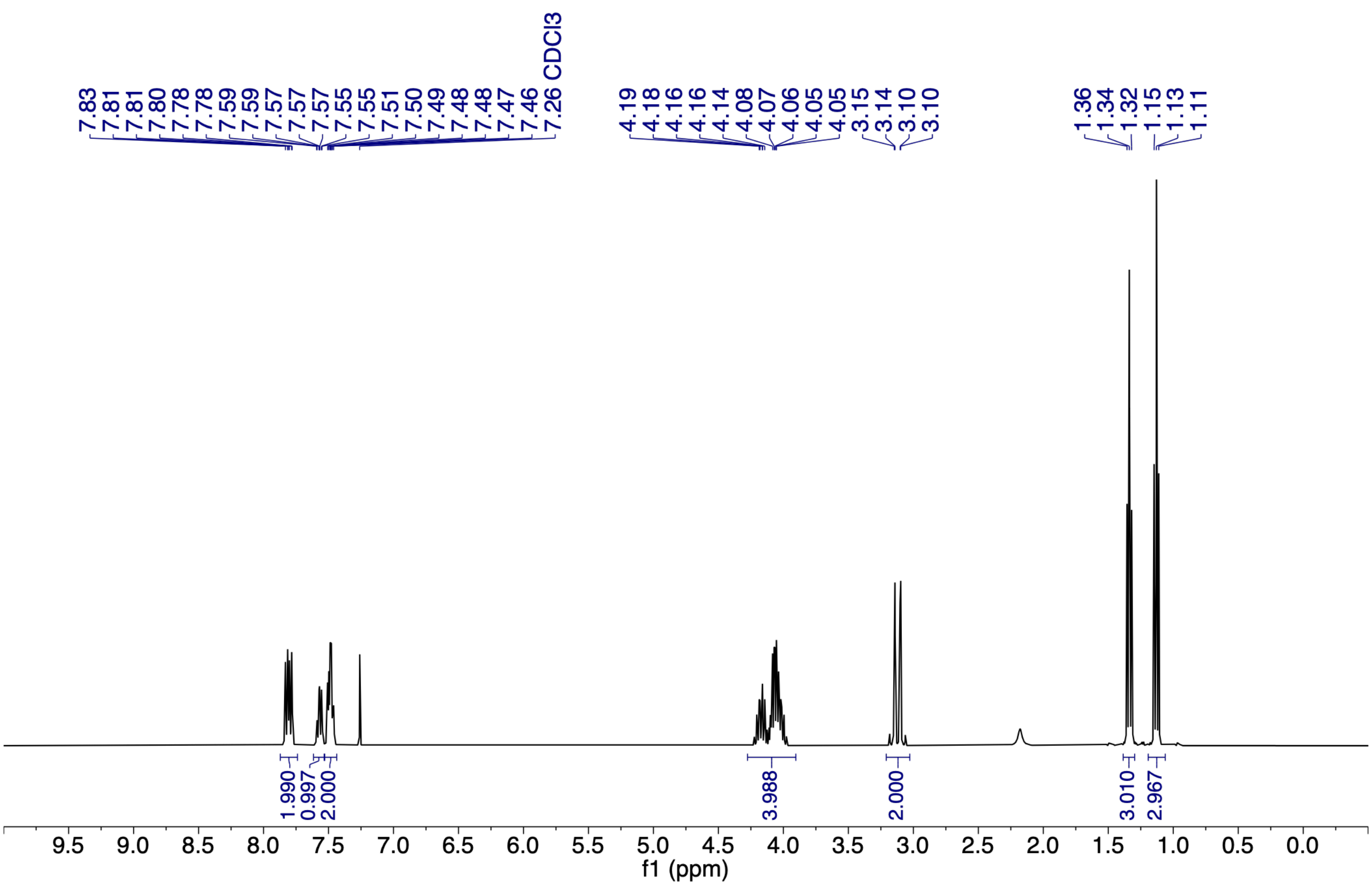

(Ethoxy-phenyl-phosphinoyl)-acetic acid ethyl ester (2b)

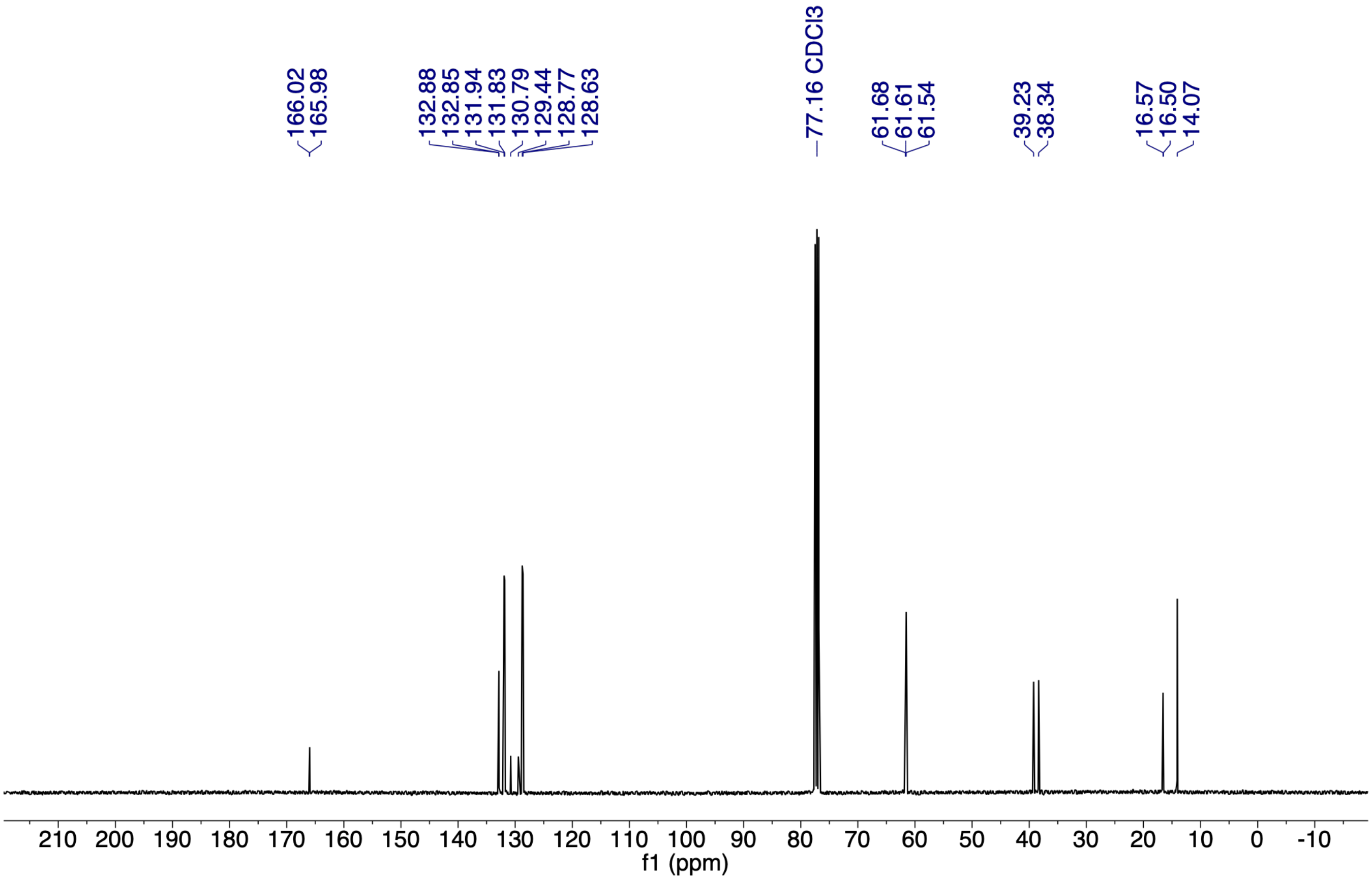

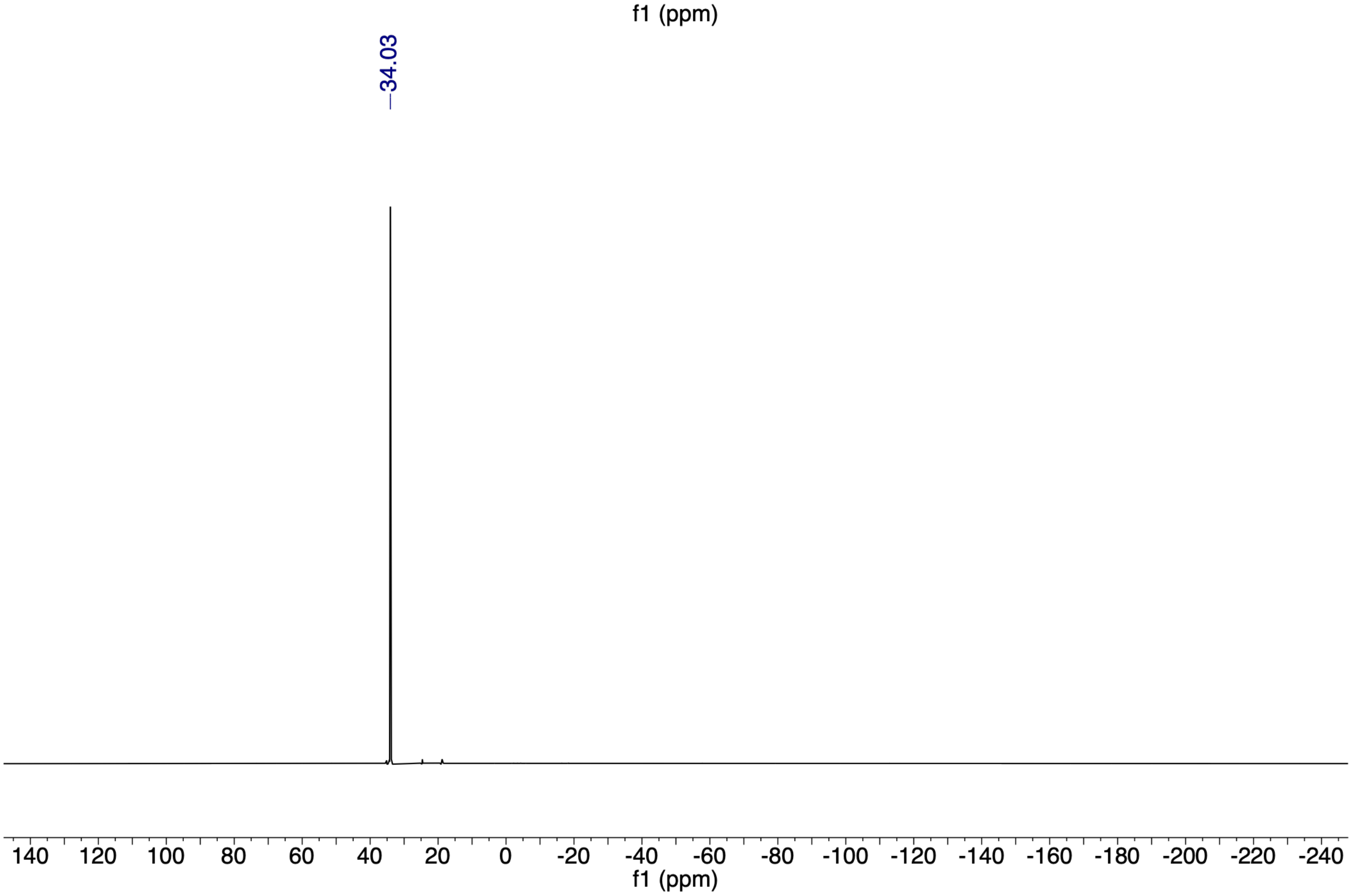

(Ethoxy-phenyl-phosphinoyl)-acetic acid methyl ester (2c)

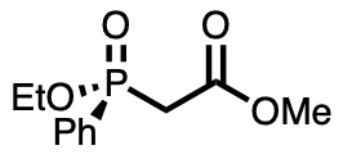

(+/–)

Synthesized with methyl bromoacetate.

CAS 95323-92-5

MW 242.21

^{1}H NMR (400 MHz, $CDCl_3$) δ 7.88 – 7.76 (m, 2H), 7.65 – 7.54 (m, 1H), 7.54 – 7.39 (m, 2H), 4.25 – 4.11 (m, 1H), 4.10 – 3.96 (m, 1H), 3.64 (s, 3H), 3.14 (d, *J* = 17.7 Hz, 2H), 1.34 (t, *J* = 7.0 Hz, 3H).

^{13}C NMR (101 MHz, $CDCl_3$) δ 166.45 (d, *J* = 5.0 Hz), 132.94 (d, *J* = 2.9 Hz), 131.80 (d, *J* = 10.3 Hz), 129.94 (d, *J* = 135.1 Hz), 128.75 (d, *J* = 13.3 Hz), 61.74 (d, *J* = 6.2 Hz), 52.53, 38.54 (d, *J* = 89.5 Hz), 16.51 (d, *J* = 6.5 Hz).

^{31}P NMR (162 MHz, $CDCl_3$) δ 33.88.

HRMS (AccuTOF-FD) m/z: M+ calcd for C11H15O4P: 242.0702; found 242.0697.

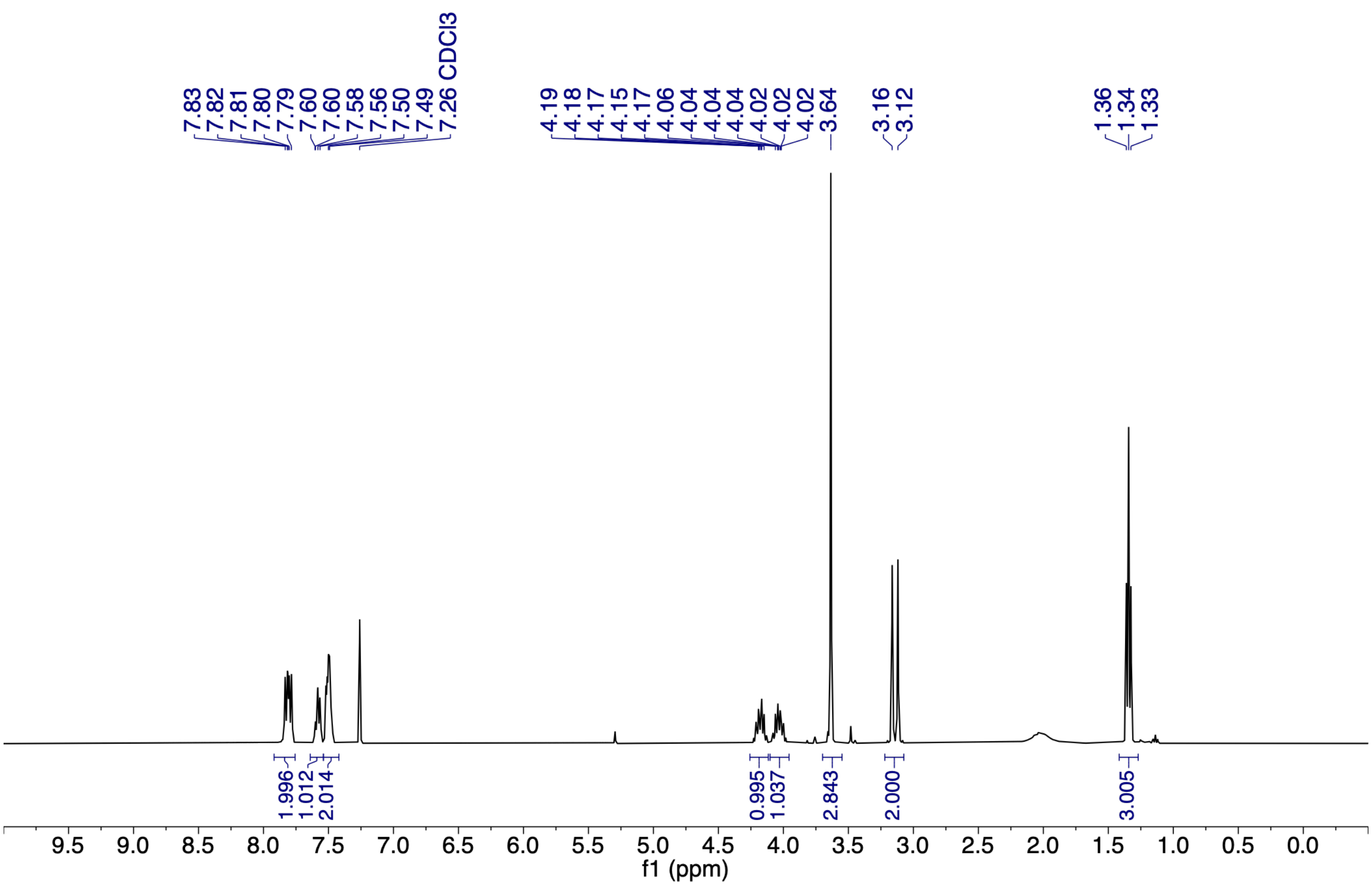

(Ethoxy-phenyl-phosphinoyl)-acetic acid methyl ester (2c)

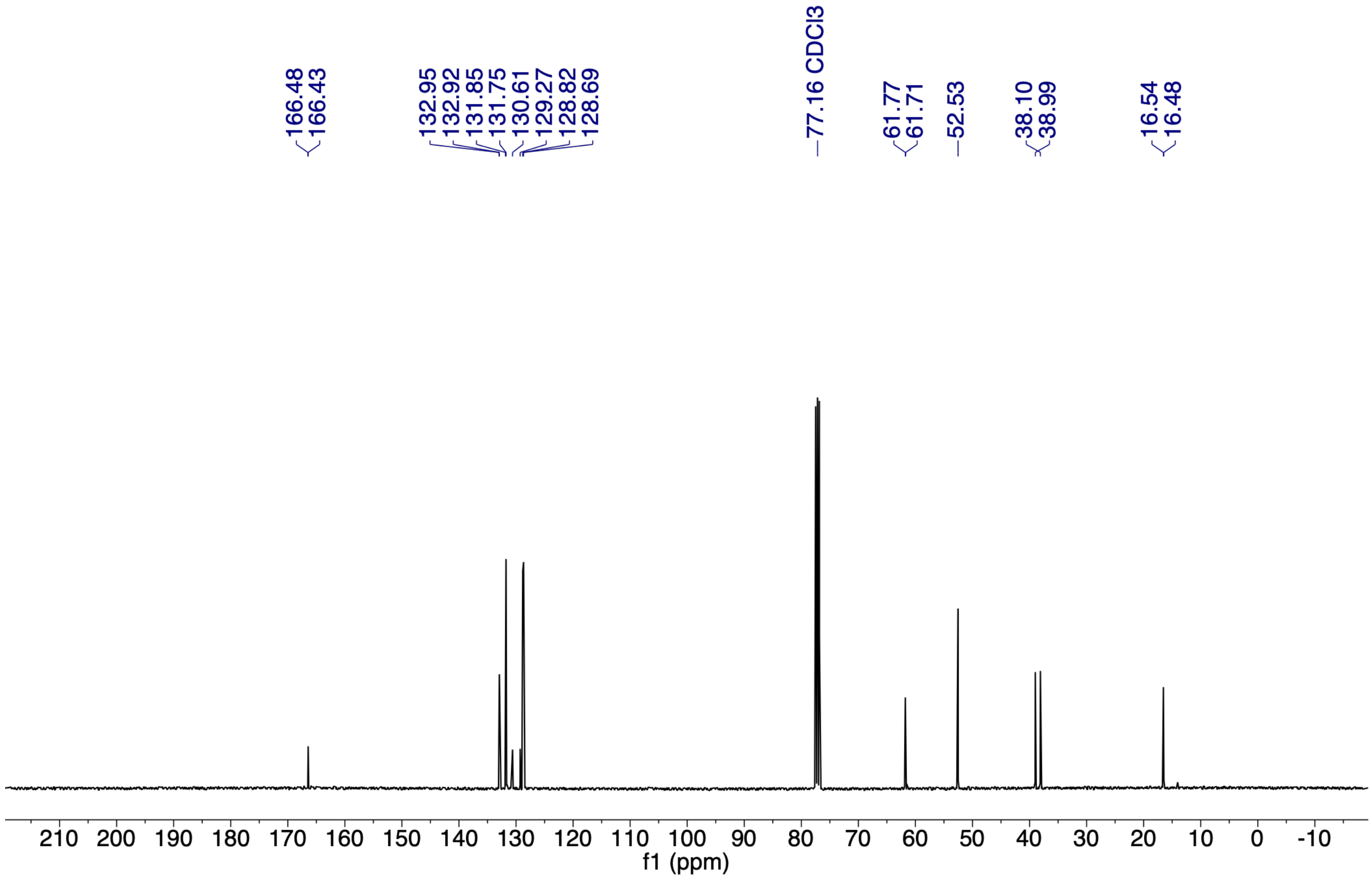

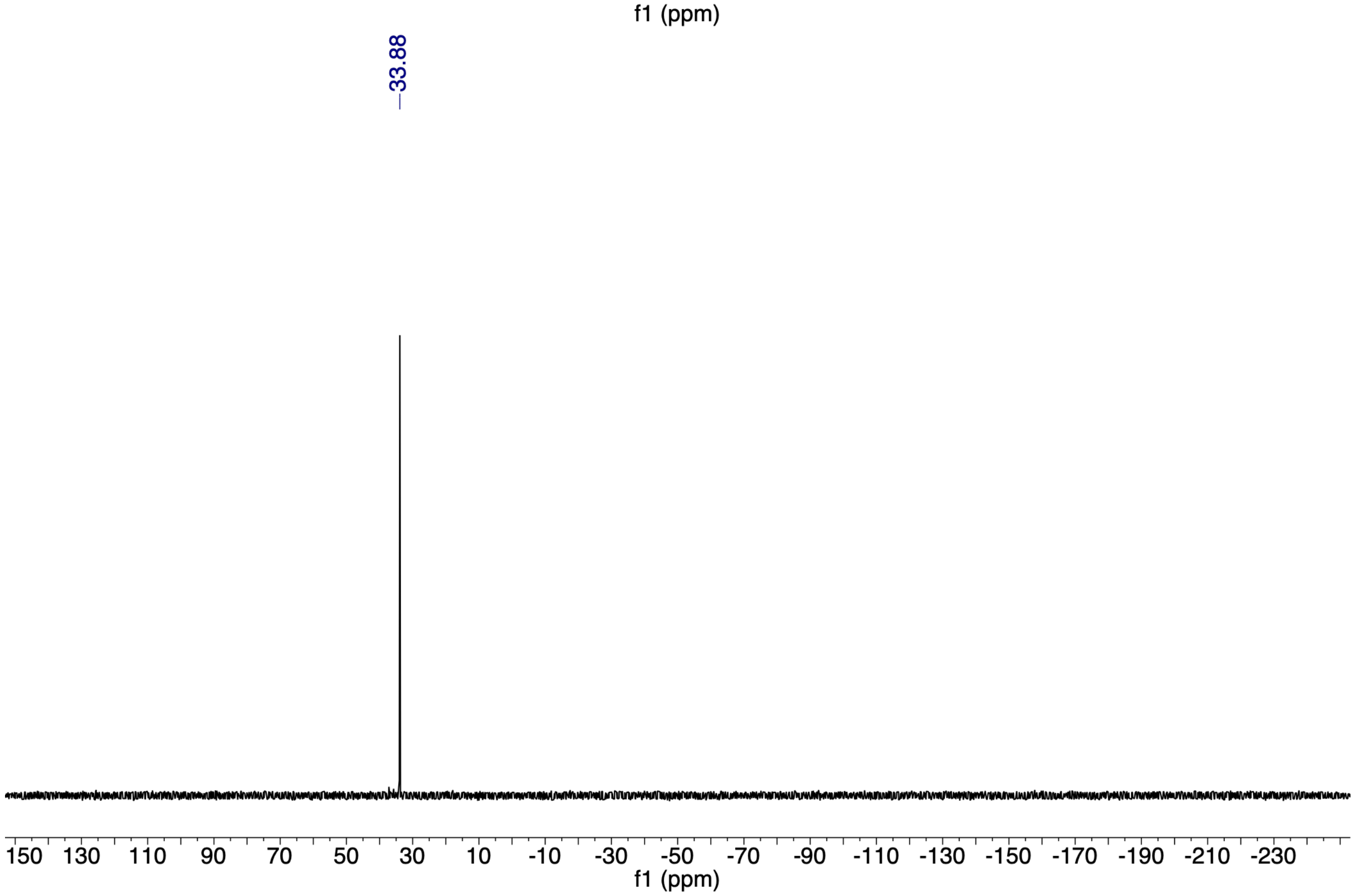

(Ethoxy-phenyl-phosphinoyl)-methyl cyanide (2d)

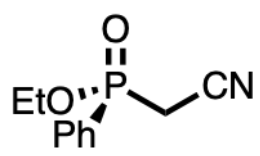

(+/–)

Synthesized with bromoacetonitrile.

CAS 64268-78-6

MW 209.19

^{1}H NMR (400 MHz, $CDCl_3$) δ 7.95 – 7.84 (m, 2H), 7.66 (td, *J* = 7.5, 1.4 Hz, 1H), 7.55 (td, *J* = 7.4, 4.0 Hz, 2H), 4.31 – 4.04 (m, 2H), 3.18 – 2.77 (m, 2H), 1.40 (t, *J* = 7.1 Hz, 3H).

^{13}C NMR (101 MHz, $CDCl_3$) δ 133.91 (d, *J* = 3.0 Hz), 132.18 (d, *J* = 10.3 Hz), 129.22 (d, *J* = 13.6 Hz), 128.03 (d, *J* = 138.3 Hz), 113.24 (d, *J* = 8.7 Hz), 62.74 (d, *J* = 6.2 Hz), 20.77 (d, *J* = 93.3 Hz), 16.52 (d, *J* = 6.3 Hz).

^{31}P NMR (162 MHz, $CDCl_3$) δ 29.32.

HRMS (AccuTOF-FD) m/z: M+ calcd for C10H12NO2P: 209.0600; found 209.0606.

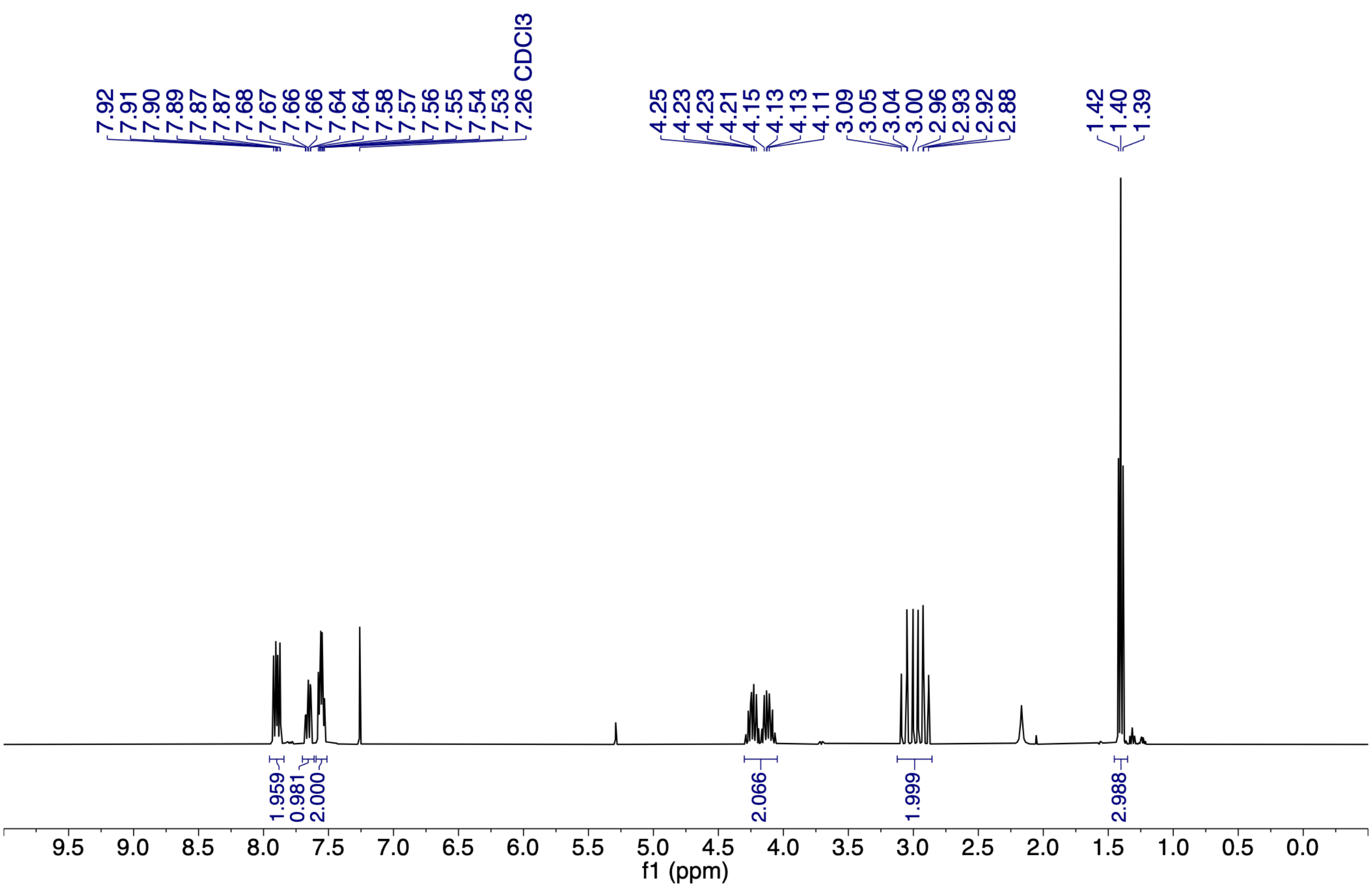

(Ethoxy-phenyl-phosphinoyl)-methyl cyanide (2d)

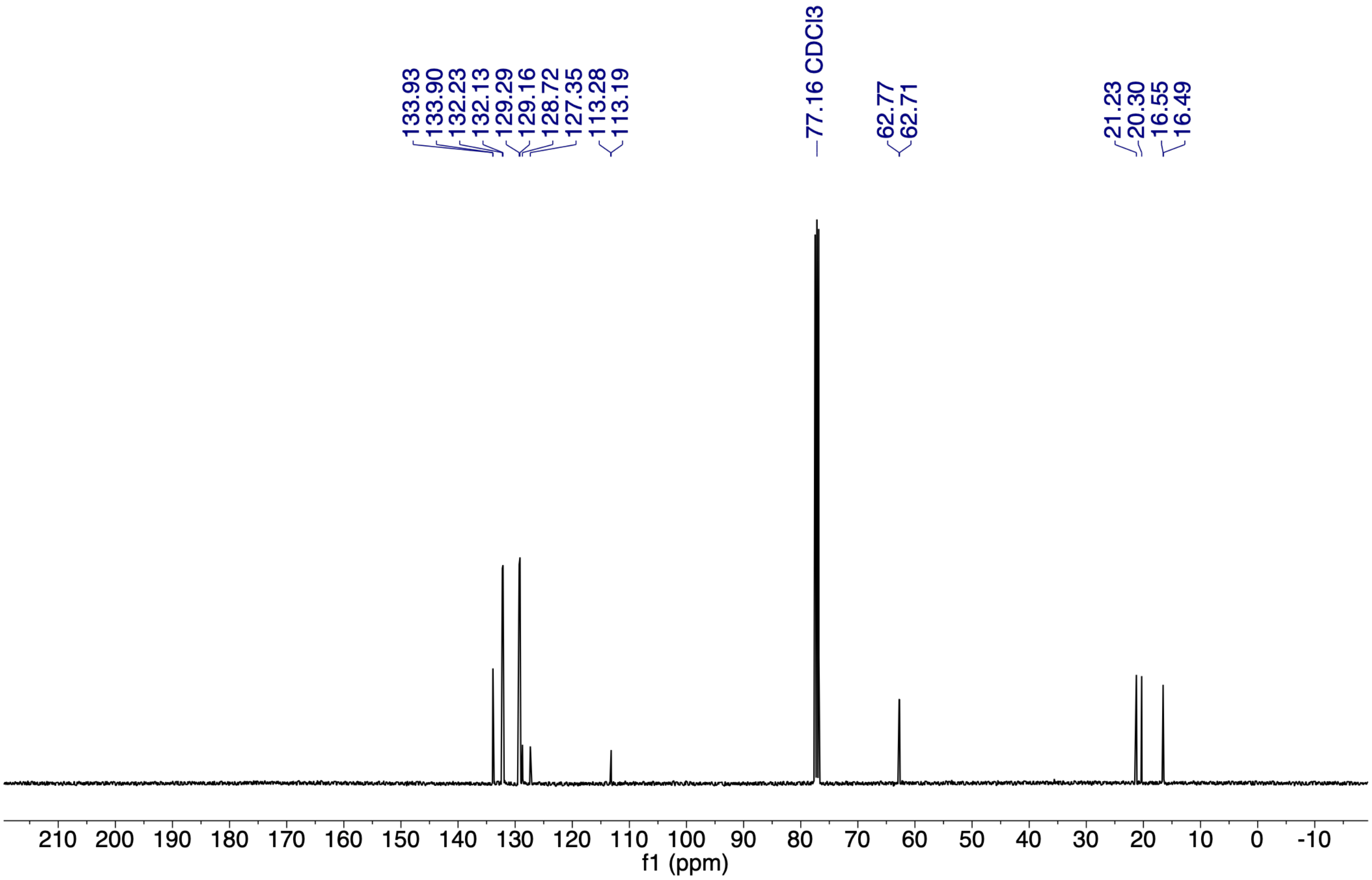

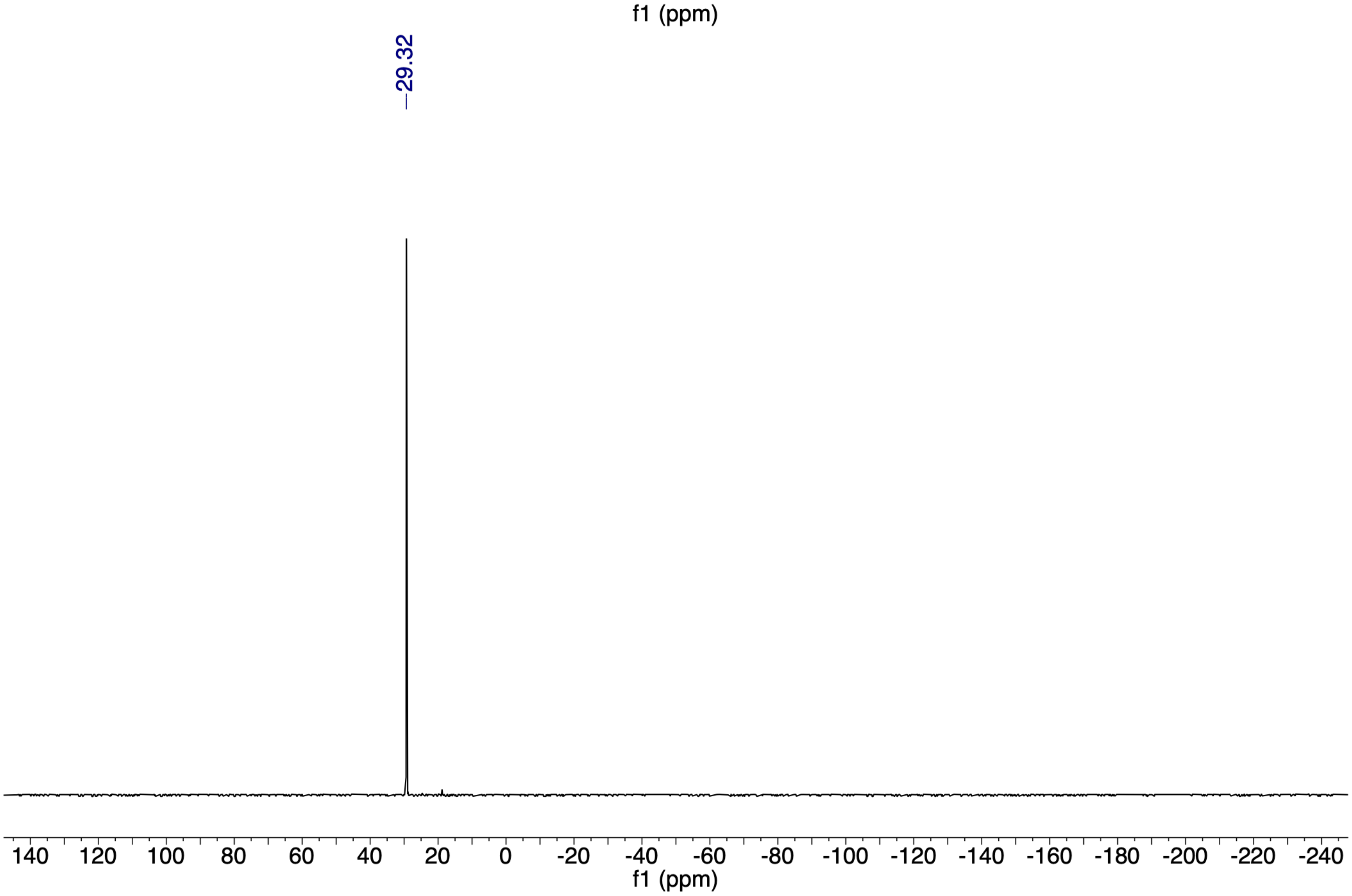

(Ethoxy-phenyl-phosphinoyl)-alpha butyrolactone (2e)

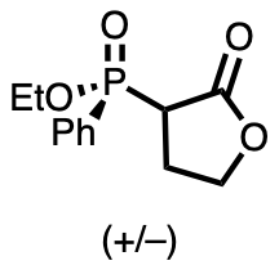

Synthesized with α-Bromo-γ-butyrolactone.

MW 254.22

^{1}H NMR (400 MHz, $CDCl_3$) δ 7.96 – 7.77 (m, 2H), 7.61 (t, *J* = 7.5 Hz, 1H), 7.56 – 7.43 (m, 2H), 4.51 – 4.39 (m, 0.5H), 4.35 (td, *J* = 8.7, 3.8 Hz, 0.5H), 4.29 – 4.13 (m, 1.5H), 4.12 – 3.92 (m, 1.5H), 3.25 (ddd, *J* = 20.7, 9.5, 6.4 Hz, 0.5H), 3.10 (ddd, *J* = 15.0, 10.3, 4.5 Hz, 0.5H), 2.78 – 2.39 (m, 2H), 1.37 (t, *J* = 7.1 Hz, 1.5H), 1.33 (t, *J* = 7.0 Hz, 1.5H).

^{13}C NMR (101 MHz, $CDCl_3$) δ 172.63 (d, *J* = 4.5 Hz), 172.38, 133.43 (d, *J* = 2.9 Hz), 133.30 (d, *J* = 2.9 Hz), 132.63 (d, *J* = 8.4 Hz), 132.53 (d, *J* = 8.7 Hz), 129.07 (d, *J* = 86.5 Hz), 129.00 (d, *J* = 8.9 Hz), 128.87 (d, *J* = 9.1 Hz), 127.75 (d, *J* = 85.9 Hz), 67.70 (d, *J* = 3.4 Hz), 67.32 (d, *J* = 4.5 Hz), 62.34 (d, *J* = 6.5 Hz), 62.07 (d, *J* = 6.3 Hz), 42.44 (d, *J* = 56.0 Hz), 41.50 (d, *J* = 57.4 Hz), 24.53 (d, *J* = 2.6 Hz), 24.10 (d, *J* = 1.9 Hz), 16.59, 16.53.

^{31}P NMR (162 MHz, $CDCl_3$) δ 35.89, 35.27.

HRMS (ESI-TOF) m/z: $[M+H]^+$ calcd for C12H16O4P: 255.0781; found 255.0782.

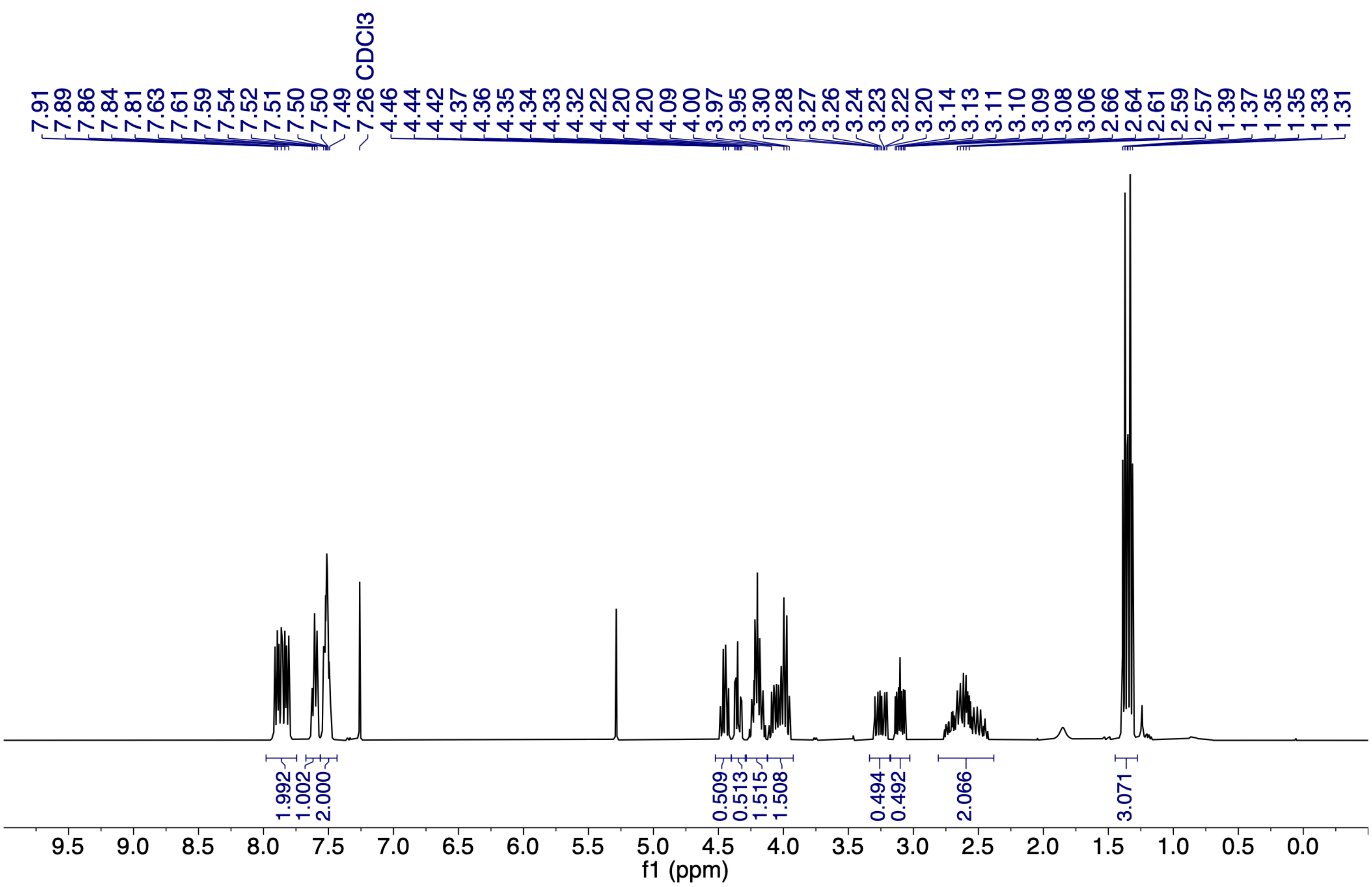

(Ethoxy-phenyl-phosphinoyl)-alpha butyrolactone (2e)

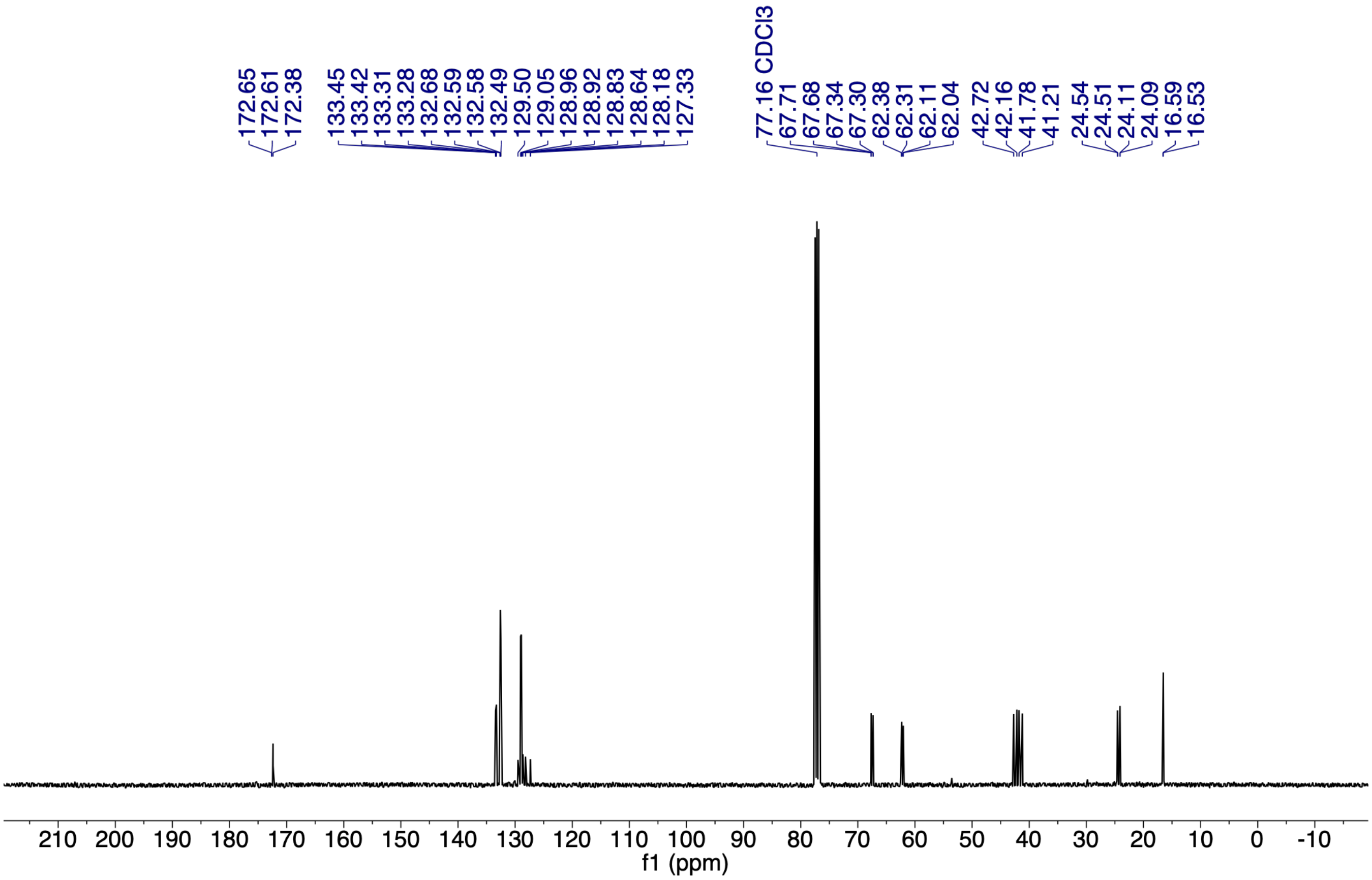

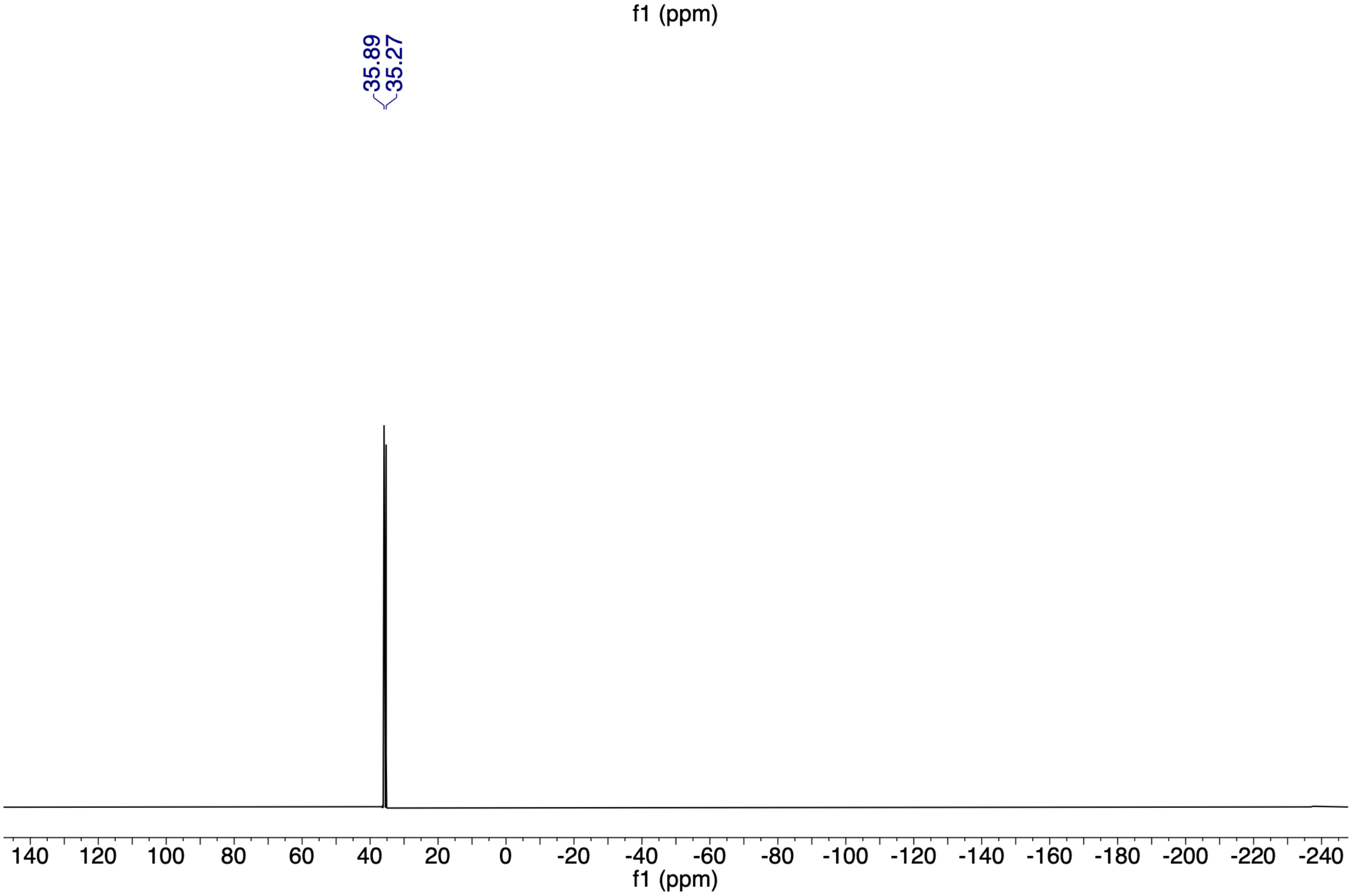

(Ethoxy-4-fluorophenyl-phosphinoyl)-acetic acid ethyl ester (9b)

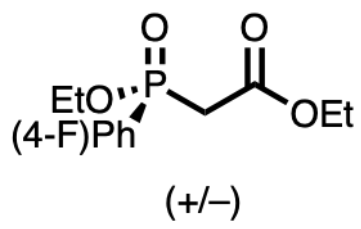

Synthesized from Ethyl (4-fluorophenyl)phosphinate.

MW 274.23

^{1}H NMR (400 MHz, $CDCl_3$) δ 7.89 – 7.73 (m, 2H), 7.18 (td, *J* = 8.7, 2.7 Hz, 2H), 4.25 – 3.92 (m, 4H), 3.12 (dd, *J* = 18.1, 3.2 Hz, 2H), 1.34 (t, *J* = 7.0 Hz, 3H), 1.16 (t, *J* = 7.1 Hz, 3H).

^{13}C NMR (101 MHz, $CDCl_3$) δ 165.94 (d, *J* = 4.7 Hz), 165.70 (dd, *J* = 254.3, 3.6 Hz), 134.60 (dd, *J* = 11.6, 9.0 Hz), 126.18 (dd, *J* = 138.6, 3.4 Hz), 116.11 (dd, *J* = 21.5, 14.5 Hz), 61.75 (d, *J* = 6.3 Hz), 61.63, 38.78 (d, *J* = 90.4 Hz), 16.54 (d, *J* = 6.5 Hz), 14.12.

^{19}F NMR (376 MHz, $CDCl_3$) δ -105.28 (s, 1F).

^{31}P NMR (162 MHz, $CDCl_3$) δ 33.01.

HRMS (AccuTOF-FI) m/z: M+ calcd for C12H16FO4P: 274.0765; found 274.0763.

(Ethoxy-4-fluorophenyl-phosphinoyl)-acetic acid ethyl ester (9b)

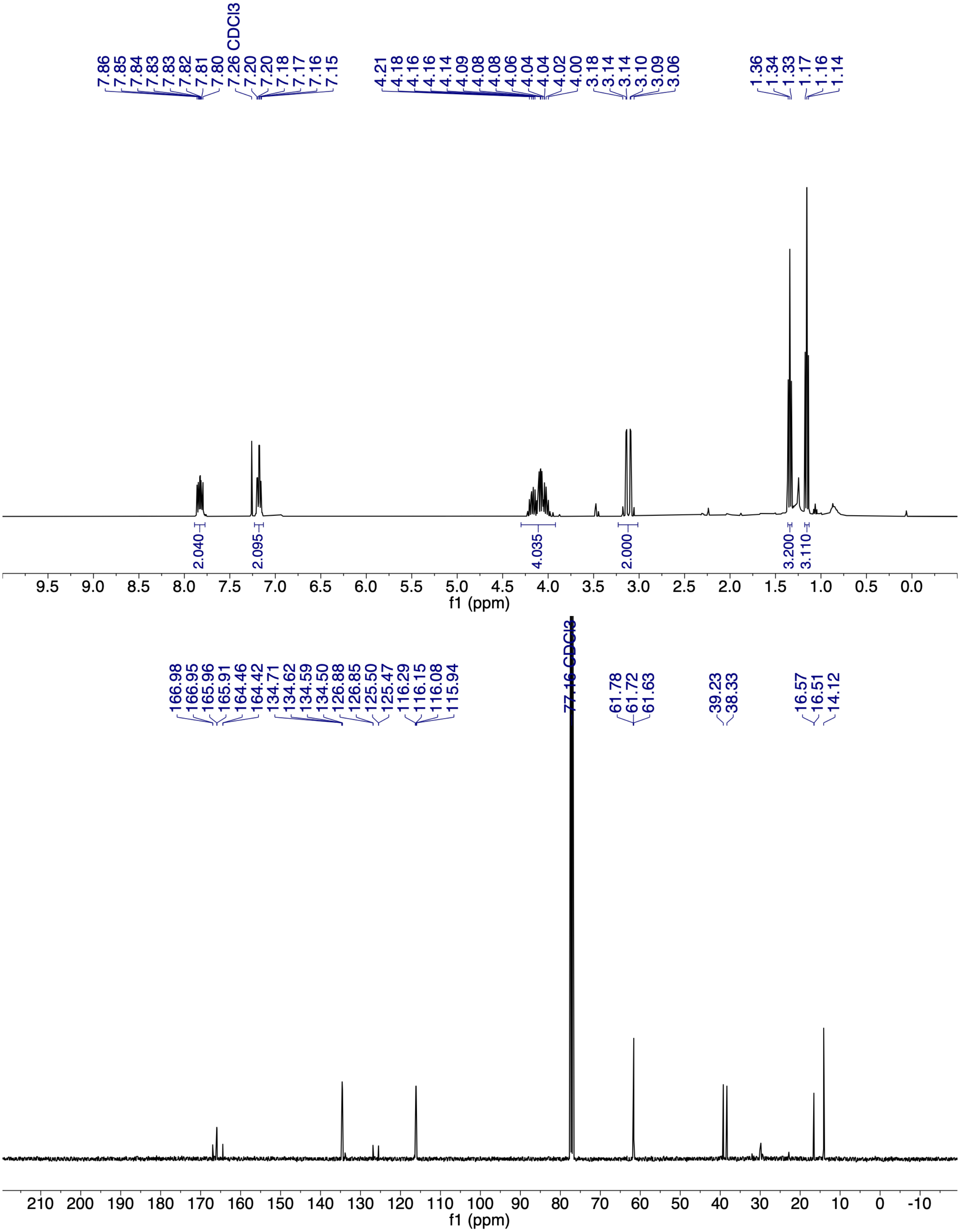

(Ethoxy-4-fluorophenyl-phosphinoyl)-acetic acid ethyl ester (9b)

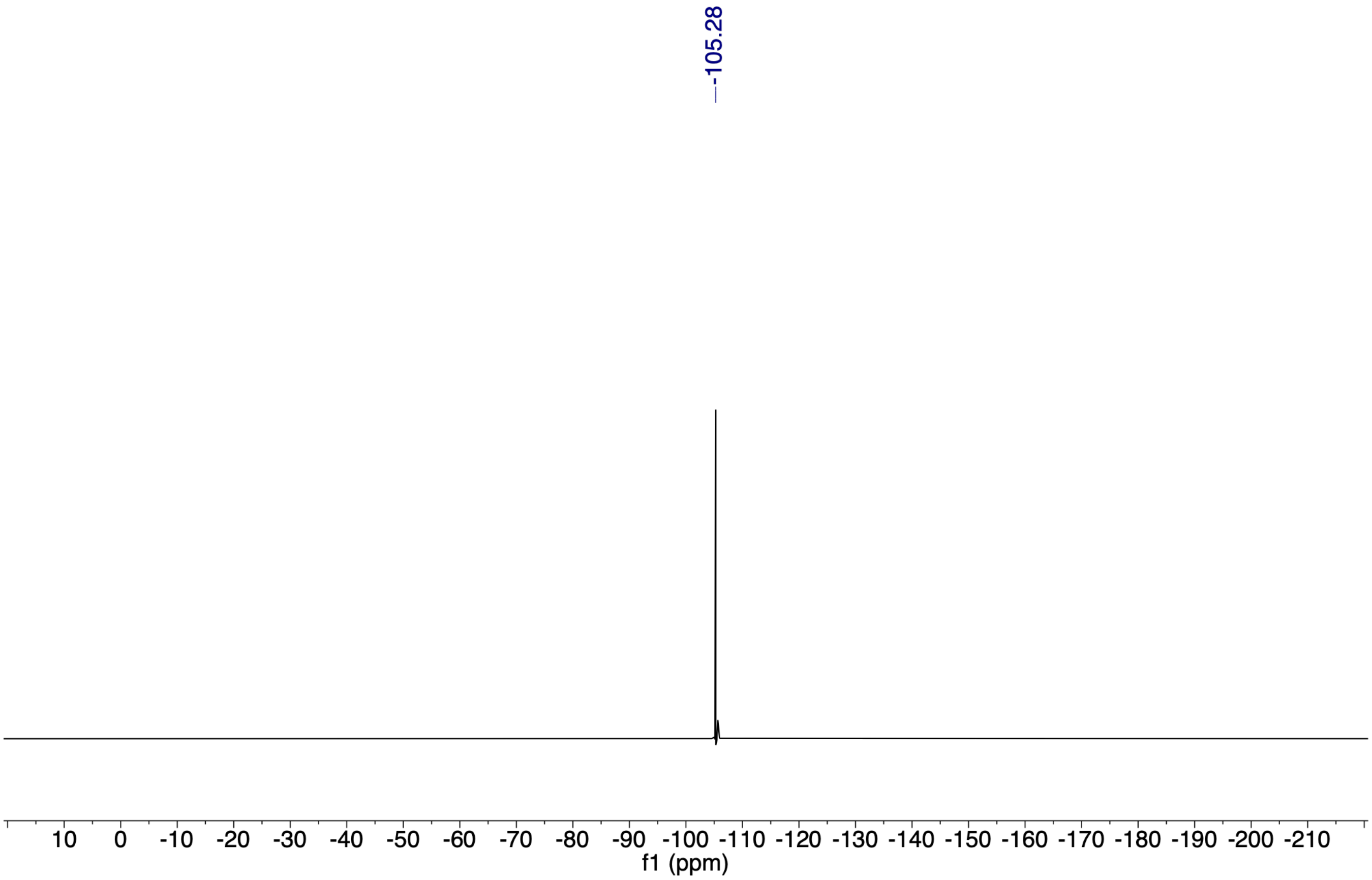

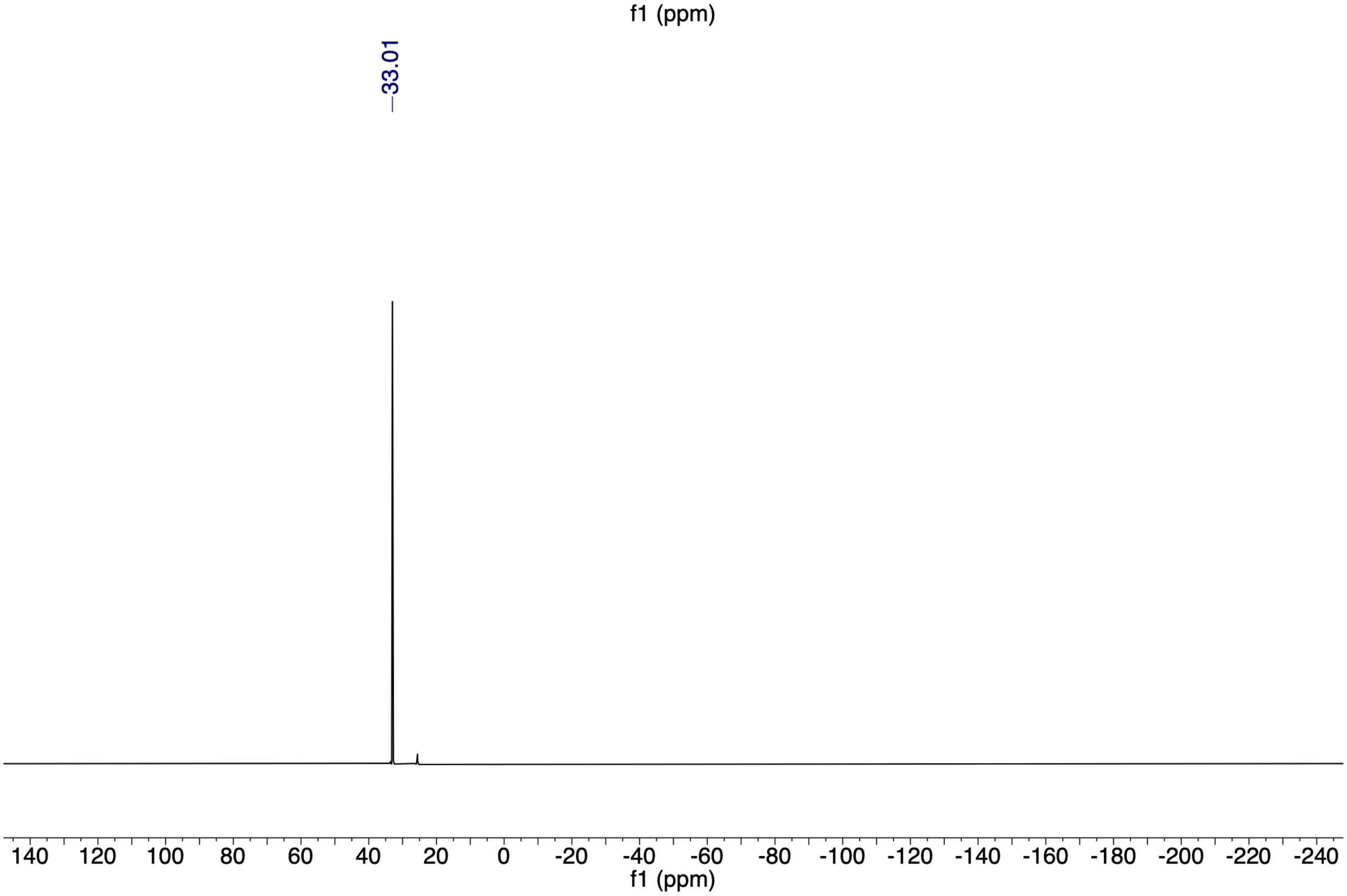

(Ethoxy-p-tolyl-phosphinoyl)-acetic acid ethyl ester (10b)

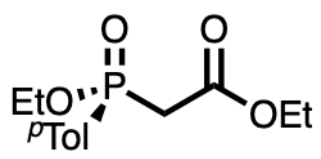

(+/–)

Synthesized from Ethyl (p-tolyl)phosphinate.

MW 270.26

^{1}H NMR (400 MHz, $CDCl_3$) δ 7.68 (dd, J = 12.3, 8.1 Hz, 2H), 7.33 – 7.25 (m, 2H), 4.22 – 3.93 (m, 4H), 3.17 – 3.02 (m, 2H), 2.51 – 2.33 (m, 3H), 1.33 (t, J = 7.0 Hz, 3H), 1.15 (t, J = 7.1 Hz, 3H).

^{13}C NMR (101 MHz, $CDCl_3$) δ 166.10 (d, J = 4.8 Hz), 143.53 (d, J = 3.0 Hz), 131.93 (d, J = 10.6 Hz), 129.45 (d, J = 13.7 Hz), 126.71 (d, J = 137.7 Hz), 61.52 (d, J = 6.2 Hz), 61.51, 38.82 (d, J = 89.3 Hz), 21.80 (d, J = 1.4 Hz), 16.53 (d, J = 6.6 Hz), 14.09.

^{31}P NMR (162 MHz, $CDCl_3$) δ 34.63.

HRMS (AccuTOF-FI) m/z: M+ calcd for C13H19O4P: 270.1016; found 270.1015.

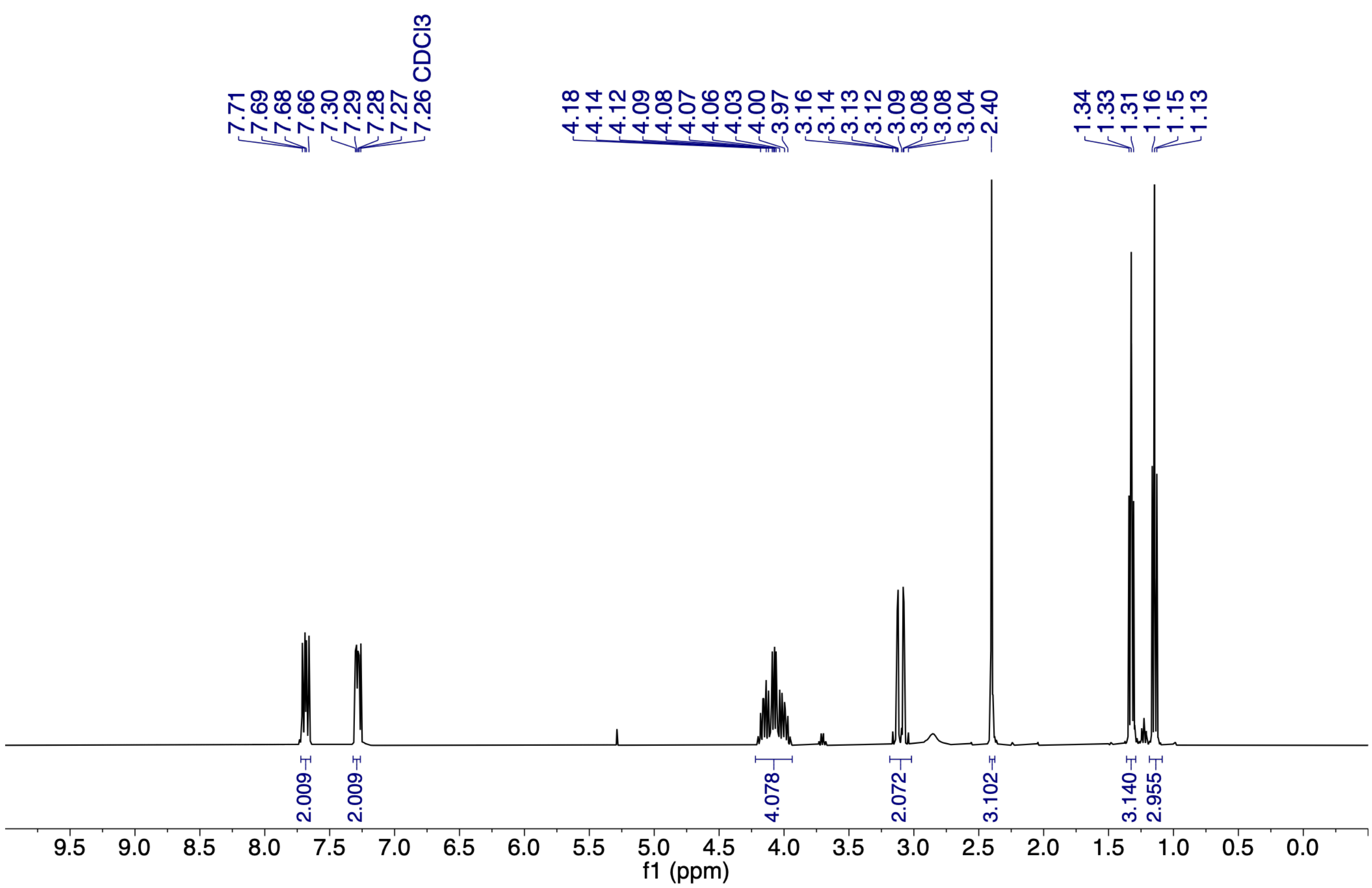

(Ethoxy-p-tolyl-phosphinoyl)-acetic acid ethyl ester (10b)

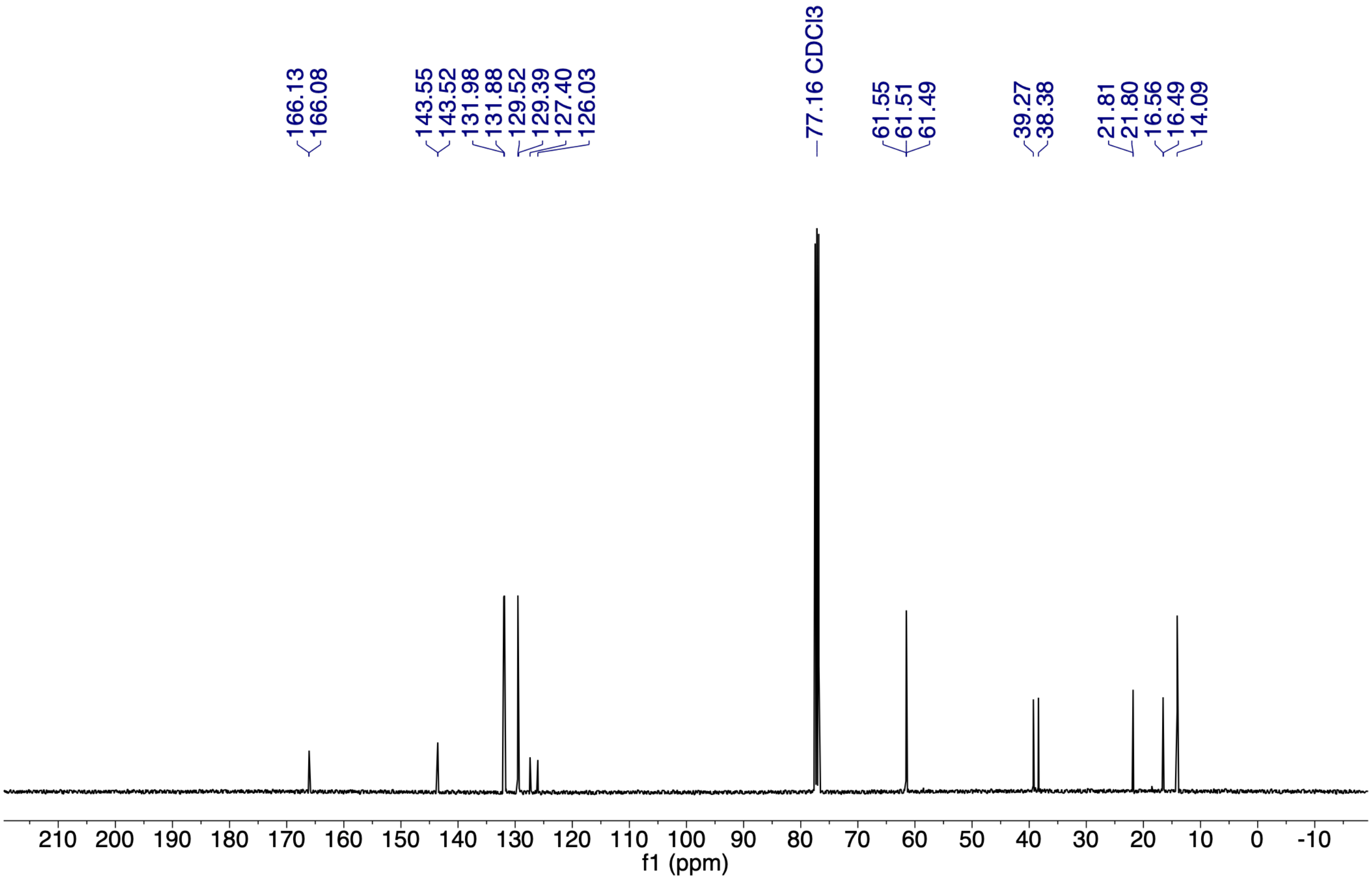

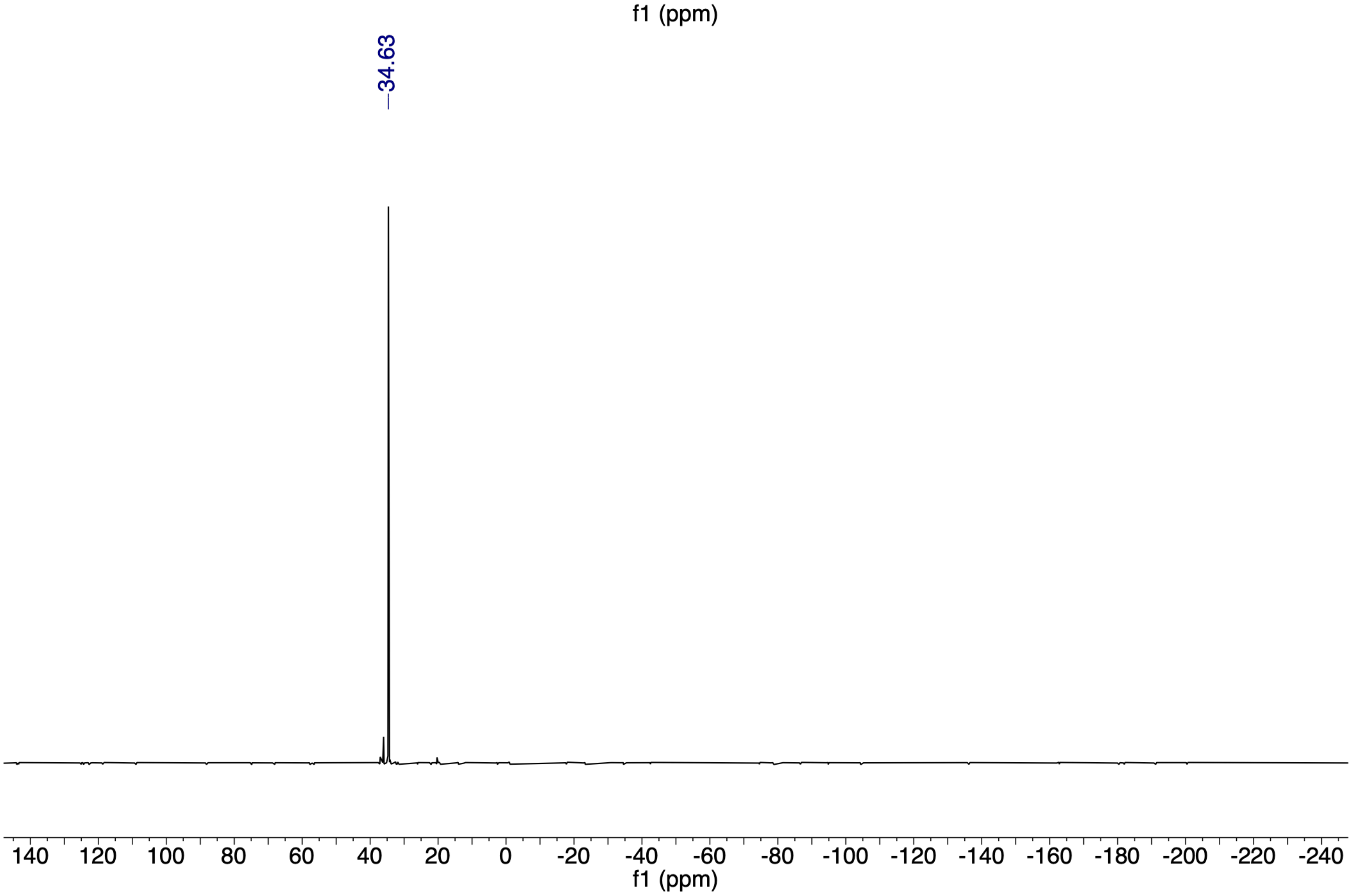

(Ethoxy-2-napthyl-phosphinoyl)-acetic acid ethyl ester (11b)

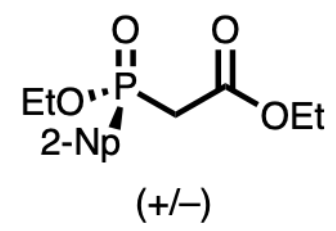

Synthesized from Ethyl (2-napthyl)phosphinate.

MW 306.30

^{1}H NMR (400 MHz, $CDCl_3$) δ 8.57 – 8.34 (m, 1H), 7.99 – 7.84 (m, 3H), 7.74 (ddd, *J* = 10.0, 8.3, 1.5 Hz, 1H), 7.66 – 7.48 (m, 2H), 4.29 – 3.82 (m, 4H), 3.20 (dd, *J* = 17.9, 2.1 Hz, 2H), 1.36 (t, *J* = 7.1 Hz, 3H), 1.10 (t, *J* = 7.1 Hz, 3H).

^{13}C NMR (101 MHz, $CDCl_3$) δ 166.00 (d, *J* = 4.8 Hz), 135.23 (d, *J* = 2.4 Hz), 134.46 (d, *J* = 9.9 Hz), 132.52 (d, *J* = 14.6 Hz), 129.16, 128.62, 128.55 (d, *J* = 12.6 Hz), 127.96, 127.18 (d, *J* = 135.0 Hz), 127.13, 126.20 (d, *J* = 10.9 Hz), 61.70 (d, *J* = 6.1 Hz), 61.55, 38.82 (d, *J* = 89.6 Hz), 16.57 (d, *J* = 6.4 Hz), 14.05.

^{31}P NMR (162 MHz, $CDCl_3$) δ 34.12.

HRMS (ESI-TOF) m/z: $[M+H]^+$ calcd for C16H19O4P: 307.1094; found 307.1097.

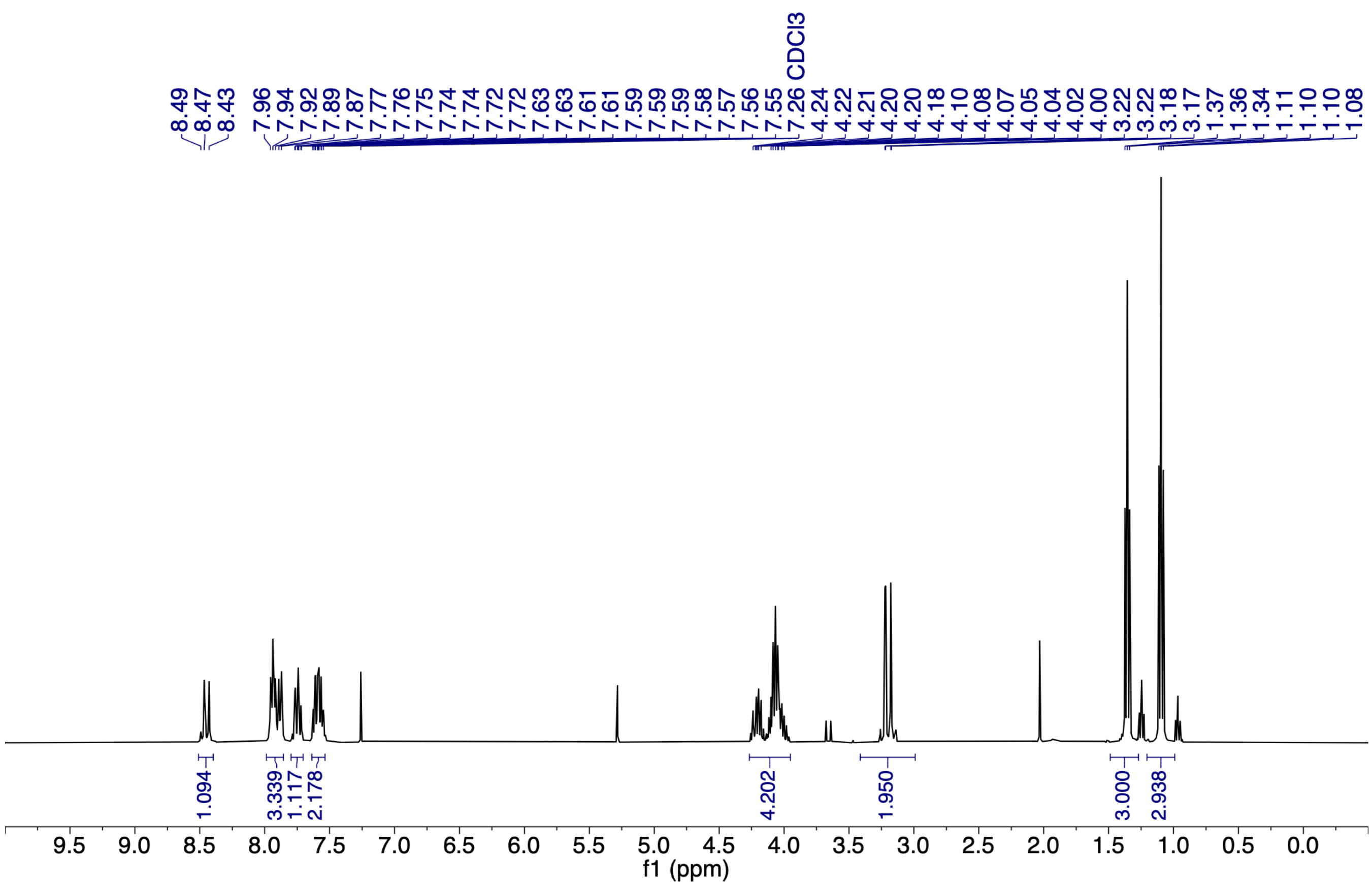

(Ethoxy-2-napthyl-phosphinoyl)-acetic acid ethyl ester (11b)

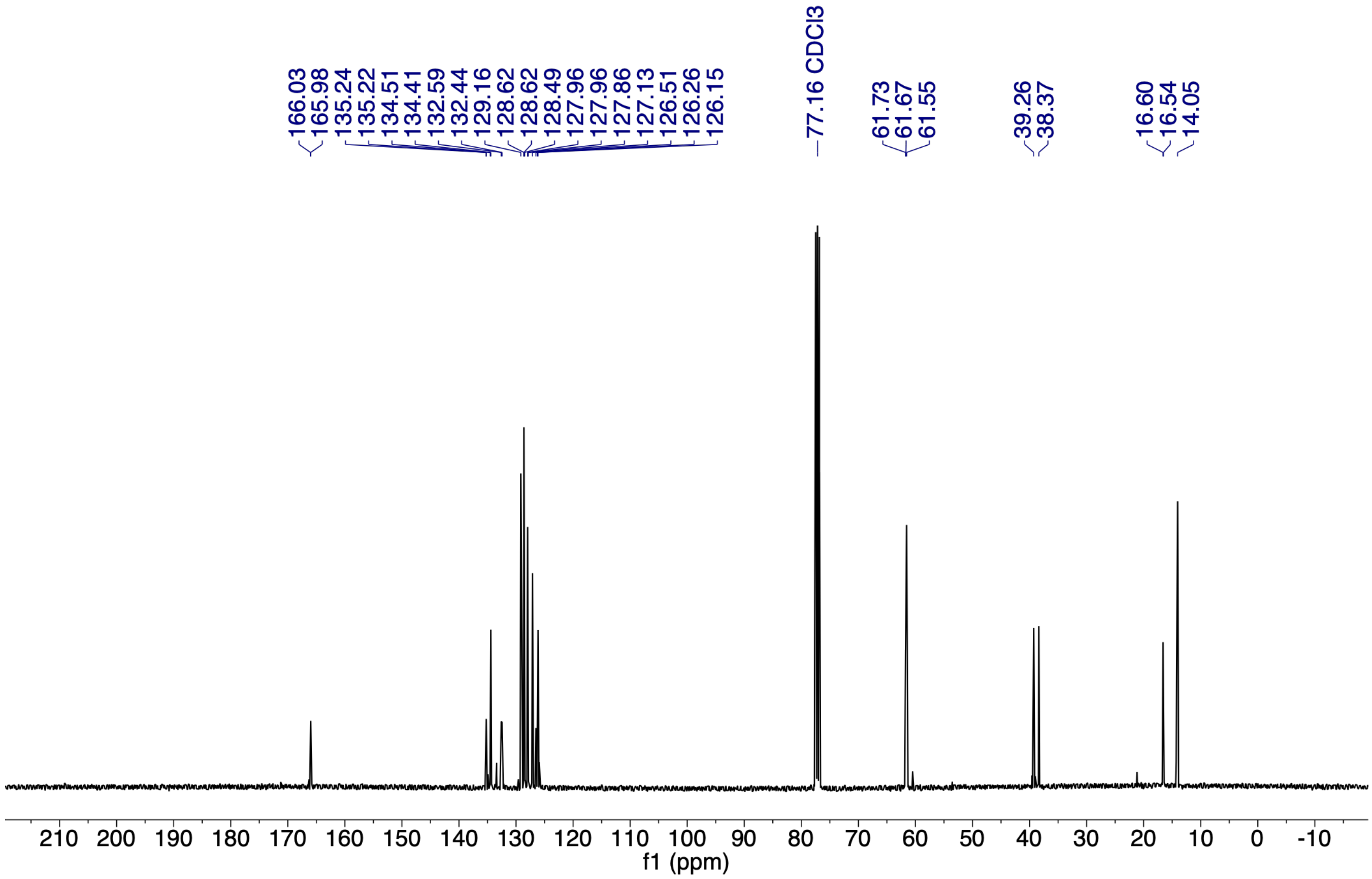

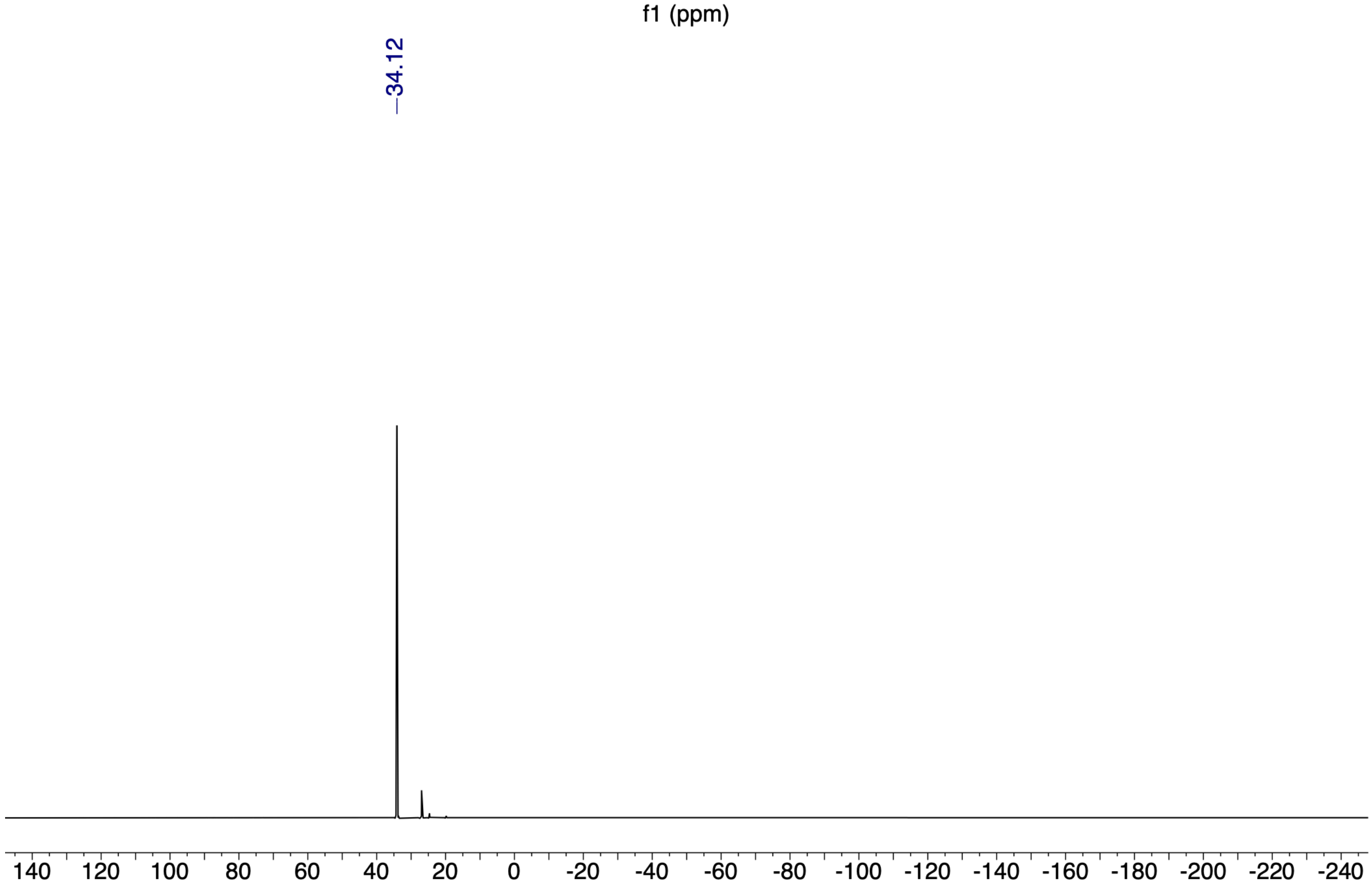

(Ethoxy-phenethyl-phosphinoyl)-acetic acid ethyl ester (12b)

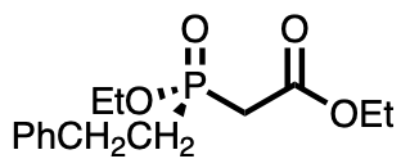

(+/–)

Synthesized from Ethyl (phenethyl)phosphinate.

MW 284.29

^{1}H NMR (400 MHz, $CDCl_3$) δ 7.33 – 7.27 (m, 2H), 7.25 – 7.16 (m, 3H), 4.26 – 4.06 (m, 4H), 3.07 – 2.85 (m, 4H), 2.21 (dddd, *J* = 14.8, 10.8, 6.9, 2.0 Hz, 2H), 1.33 (t, *J* = 7.1 Hz, 3H), 1.28 (t, *J* = 7.2 Hz, 3H).

^{13}C NMR (101 MHz, $CDCl_3$) δ 166.38 (d, *J* = 4.0 Hz), 140.66 (d, *J* = 16.2 Hz), 128.66, 128.16, 126.47, 61.61, 61.18 (d, *J* = 6.8 Hz), 36.68 (d, *J* = 76.2 Hz), 30.45 (d, *J* = 96.4 Hz), 27.57 (d, *J* = 3.6 Hz), 16.49 (d, *J* = 6.0 Hz), 14.14.

^{31}P NMR (162 MHz, $CDCl_3$) δ 46.67.

HRMS (AccuTOF-FI) m/z: M+ calcd for C14H21O4P: 284.1172; found 284.1171.

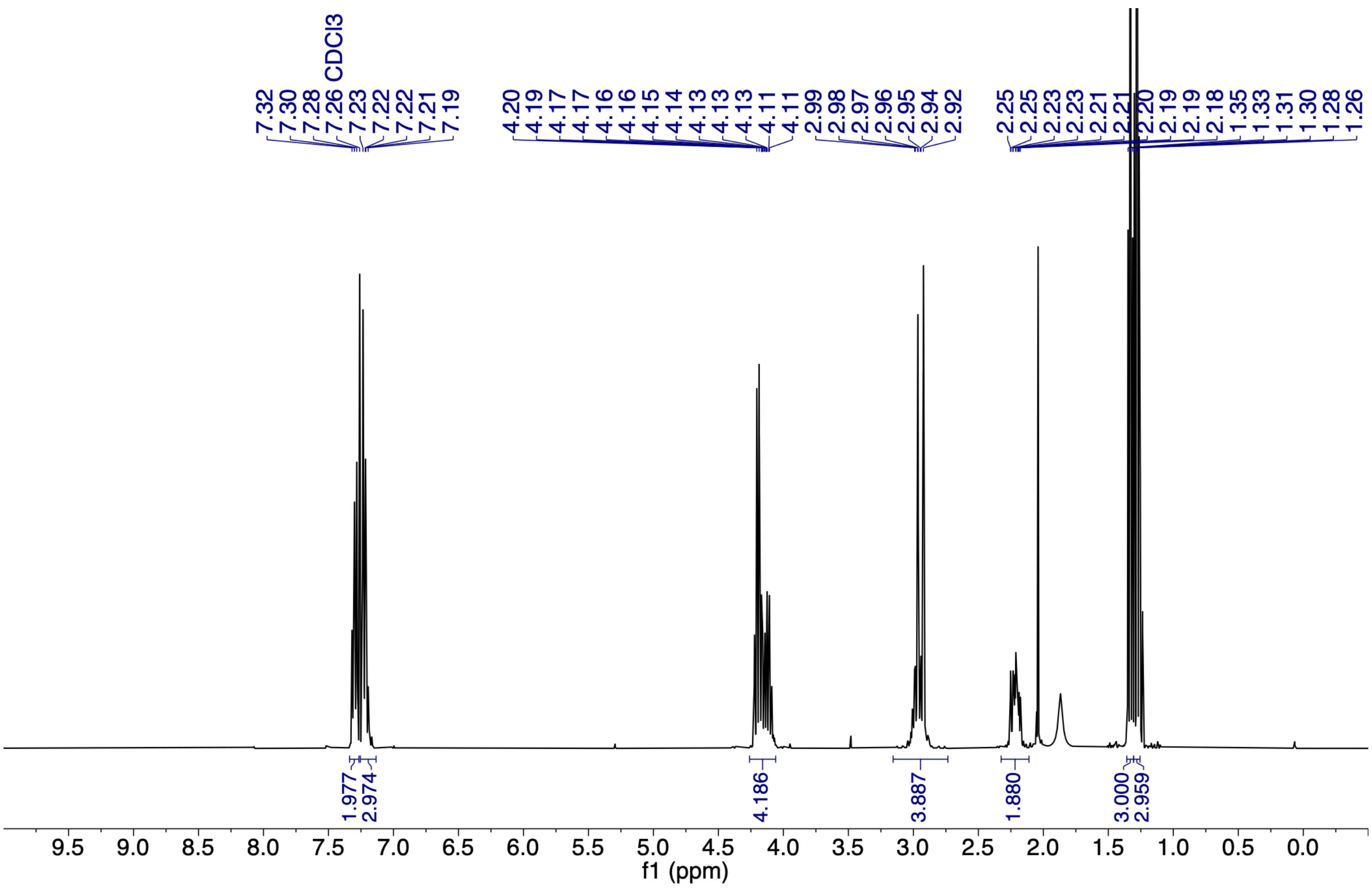

(Ethoxy-phenethyl-phosphinoyl)-acetic acid ethyl ester (12b)

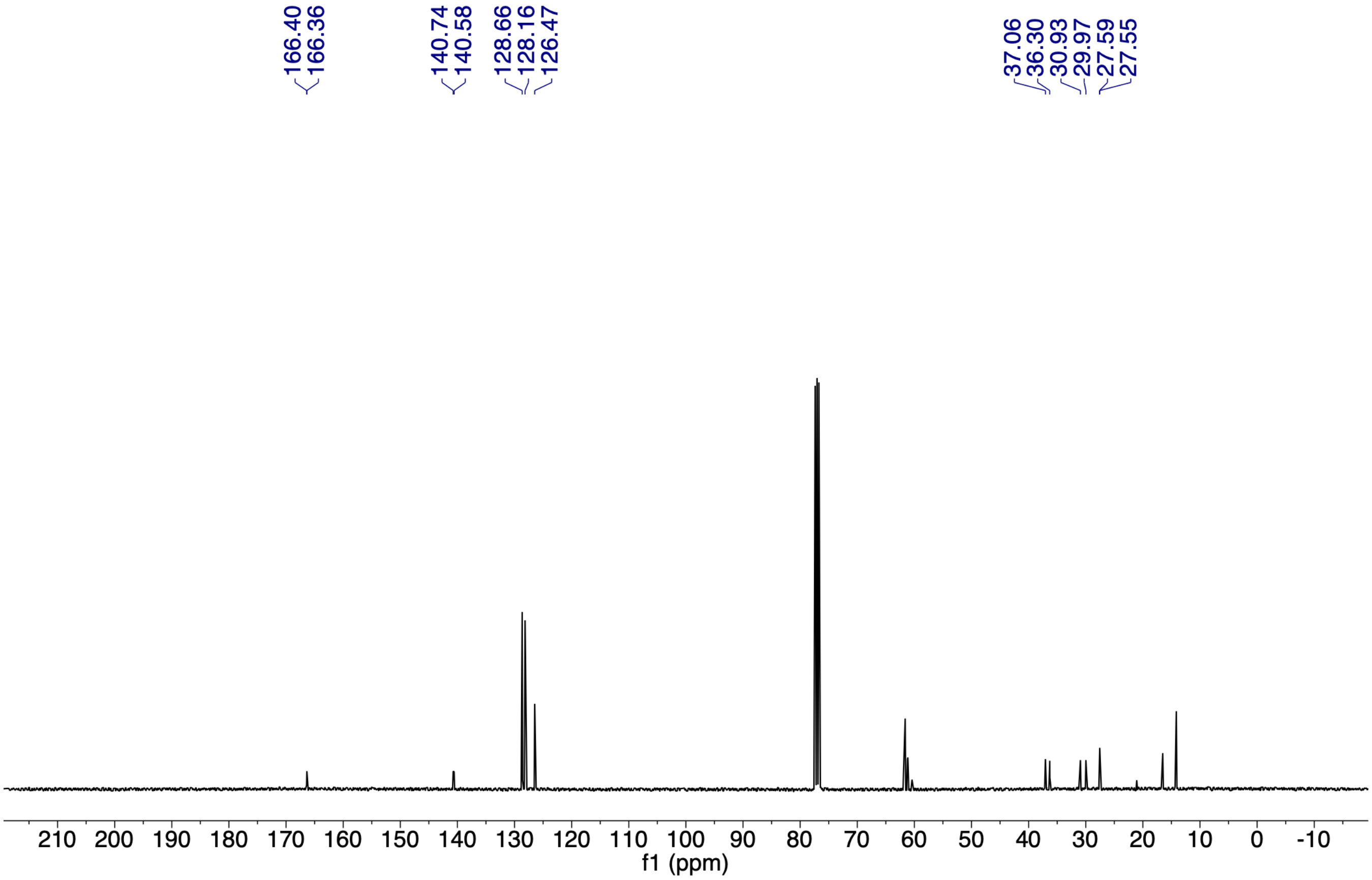

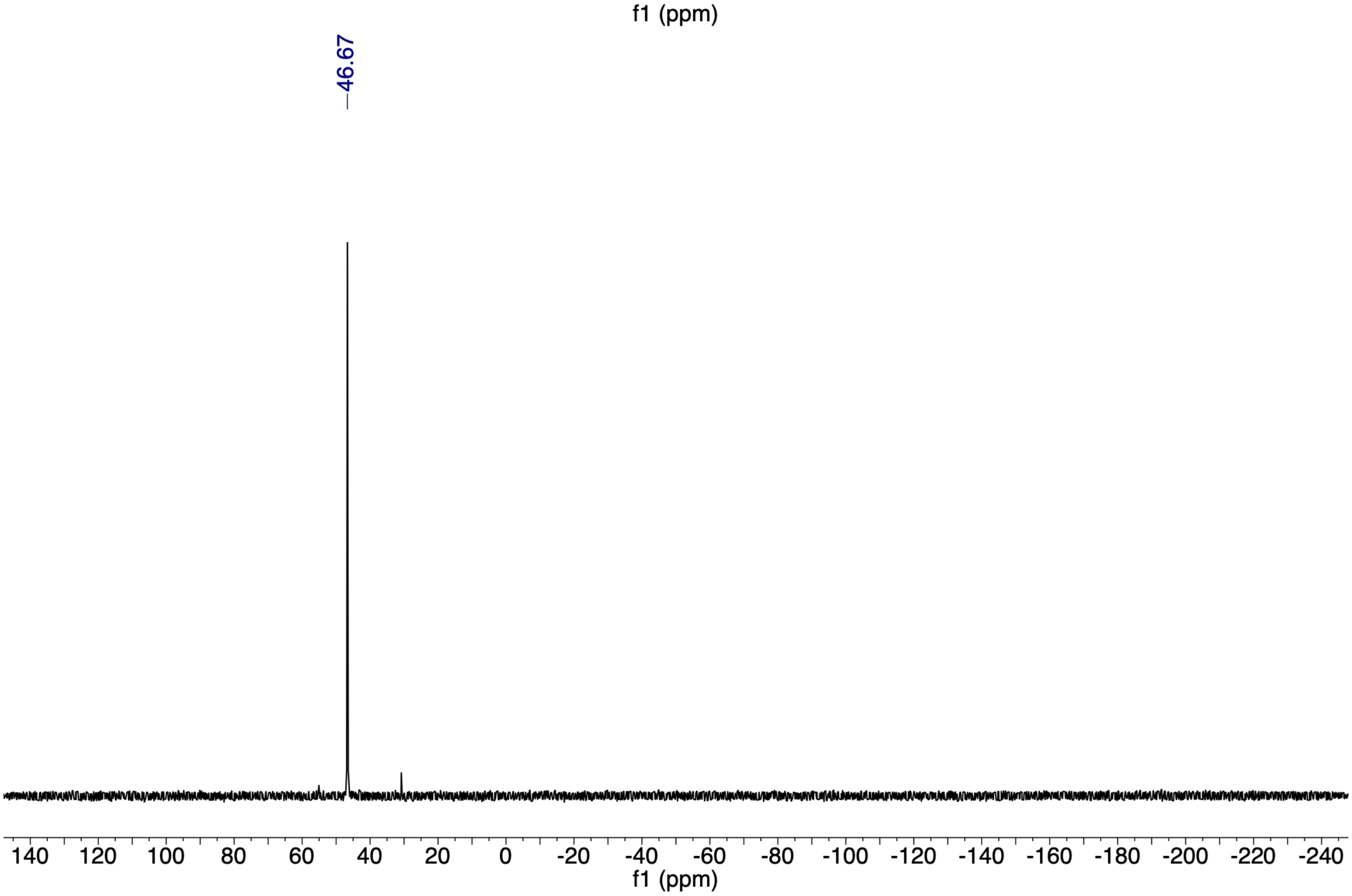

(Ethoxy-2-methylpropyl-phosphinoyl)-acetic acid ethyl ester (13b)

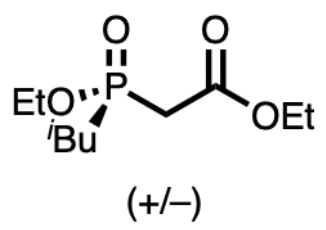

Synthesized from Ethyl (2-methylpropyl)phosphinate.

MW 236.25

^{1}H NMR (400 MHz, $CDCl_3$) δ 4.30 – 3.97 (m, 4H), 2.94 (d, *J* = 17.3 Hz, 2H), 2.17 (ddp, *J* = 13.3, 11.1, 6.7 Hz, 1H), 1.91 – 1.69 (m, 2H), 1.30 (dt, *J* = 16.8, 7.1 Hz, 6H), 1.07 (ddd, *J* = 6.6, 2.7, 0.9 Hz, 6H).

^{13}C NMR (101 MHz, $CDCl_3$) δ 166.73 (d, *J* = 4.3 Hz), 61.62, 60.97 (d, *J* = 6.9 Hz), 37.87 (d, *J* = 5.9 Hz), 37.02 (d, *J* = 28.8 Hz), 24.60 (d, *J* = 11.7 Hz), 24.25 (d, *J* = 9.5 Hz), 23.29 (d, *J* = 4.3 Hz), 16.59 (d, *J* = 6.2 Hz), 14.25.

^{31}P NMR (162 MHz, $CDCl_3$) δ 47.10.

HRMS (AccuTOF-FI) m/z: M+ calcd for C10H21O4P: 236.1172; found 236.1169.

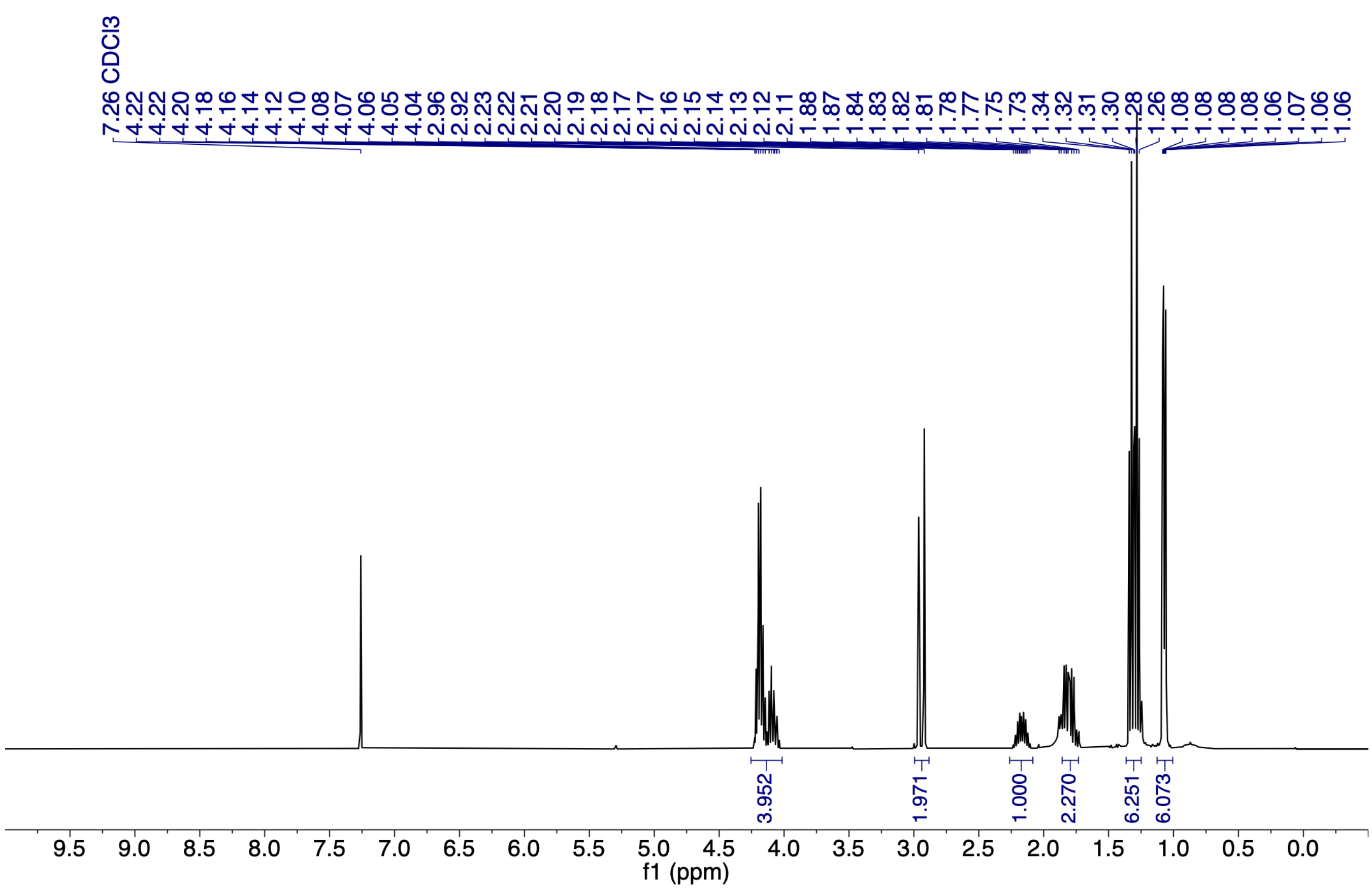

(Ethoxy-2-methylpropyl-phosphinoyl)-acetic acid ethyl ester (13b)

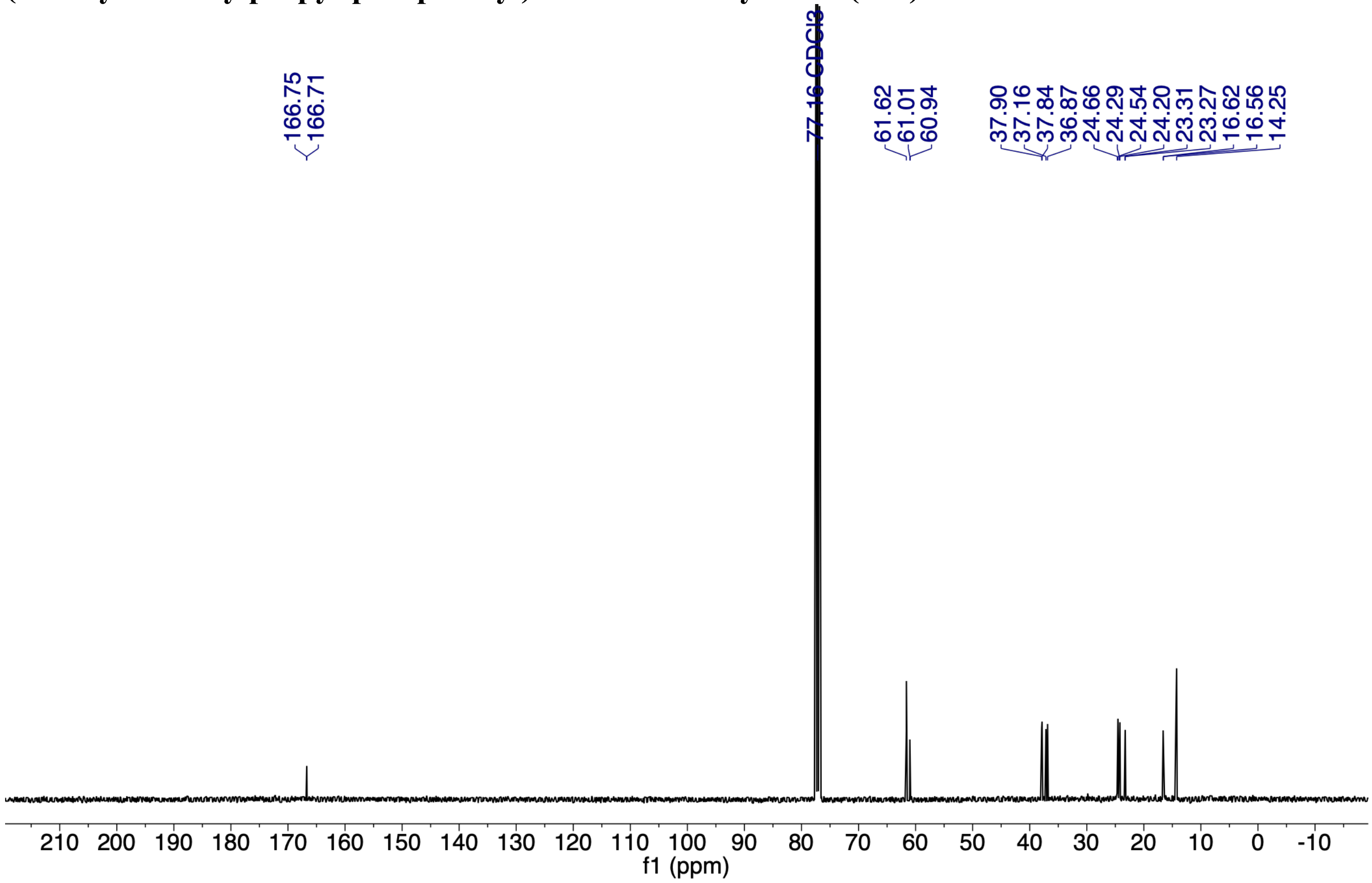

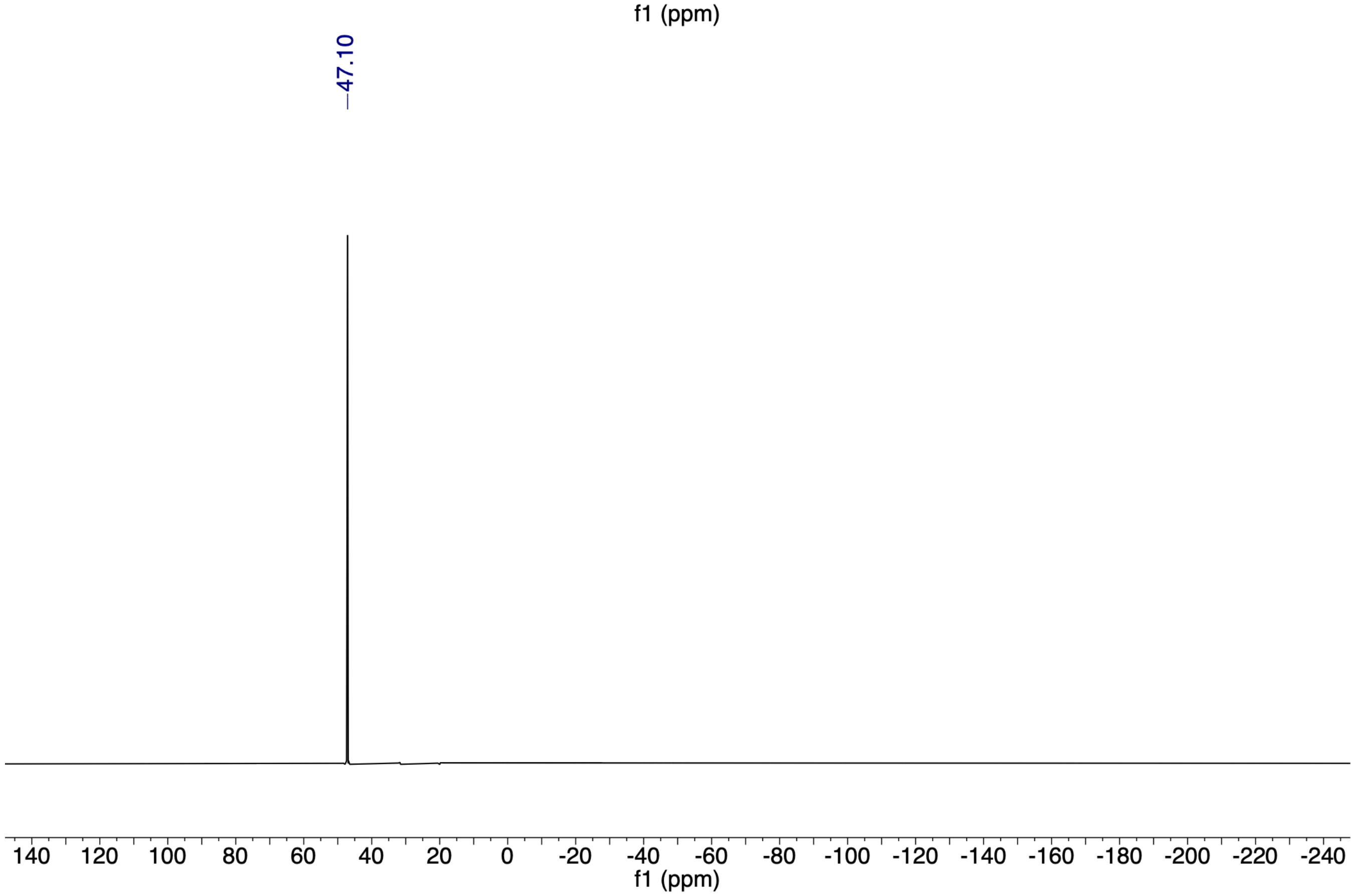

3.5 Phosphinate product standards (nitrene adducts)

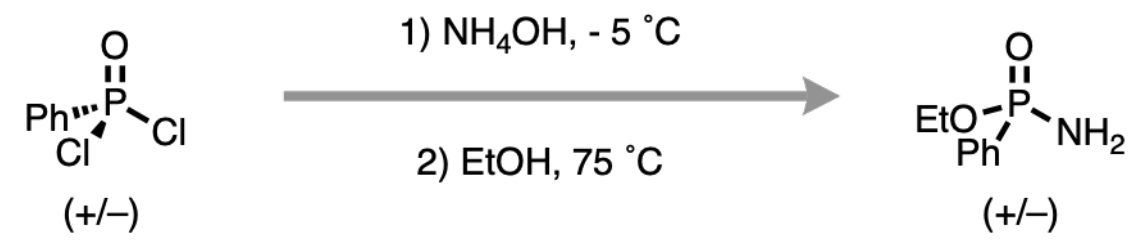

Phenylphosphonic acid monoethyl ester amide was prepared according to previously reported procedures from commercially available phenylphosphonic dichloride.[17] The crude product was purified by silica gel chromatography (ethyl acetate/methanol) to afford the desired product. All characterization data agreed with previously reported values.

Phenylphosphonic acid monoethyl ester amide (2f)

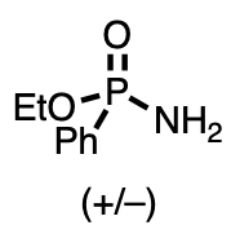

CAS 5326-06-7
MW 185.16
^{1}H NMR (400 MHz, DMSO) δ 7.72 (ddt, J = 12.8, 6.7, 1.6 Hz, 2H), 7.60 – 7.38 (m, 3H), 4.72 (d, J = 5.2 Hz, 2H), 4.07 – 3.71 (m, 2H), 1.19 (t, J = 7.0 Hz, 3H).
^{31}P NMR (162 MHz, DMSO) δ 22.58.

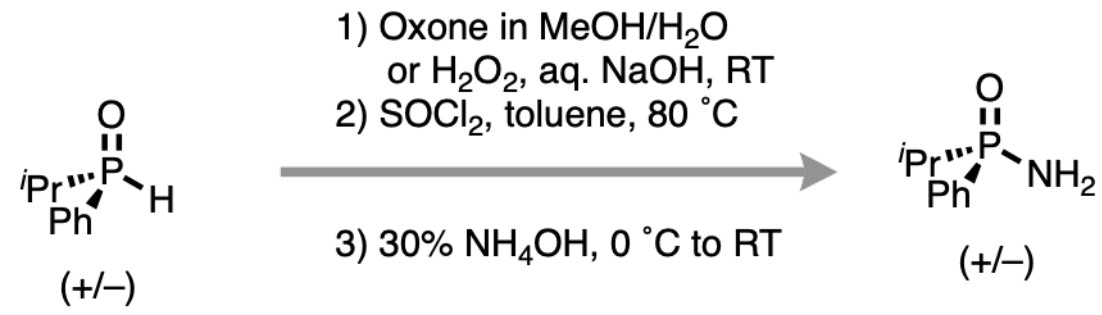

Isopropylphenylphosphinic amide was prepared according to previously reported procedures from isopropyl(phenyl)phosphine oxide.[18] Isopropyl(phenyl)phosphine oxide was oxidized to the acid, followed by halogenation, and nucleophilic displacement. All characterization data agreed with previously reported values.

Isopropylphenylphosphinic amide (4c)

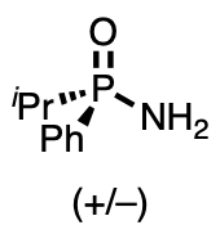

CAS 51028-16-1

MW 183.19

^{1}H NMR (400 MHz, $CDCl_3$) δ 7.91 – 7.78 (m, 2H), 7.65 – 7.51 (m, 1H), 7.51 – 7.40 (m, 2H), 2.79 (s, 2H), 2.18 – 1.95 (m, 1H), 1.18 (dd, *J* = 16.8, 7.1 Hz, 3H), 1.09 (dd, *J* = 17.3, 7.2 Hz, 3H).

^{13}C NMR (101 MHz, $CDCl_3$) δ 132.33 (d, *J* = 8.9 Hz), 131.92 (d, *J* = 2.7 Hz), 131.19 (d, *J* = 118.8 Hz), 128.35 (d, *J* = 11.8 Hz), 29.08 (d, *J* = 93.1 Hz), 16.31 (d, *J* = 2.2 Hz), 15.68 (d, *J* = 3.2 Hz).

^{31}P NMR (162 MHz, $CDCl_3$) δ 38.50.

HRMS (AccuTOF-FD) m/z: M+ calcd for C9H14NOP: 183.08075; found 183.08032.

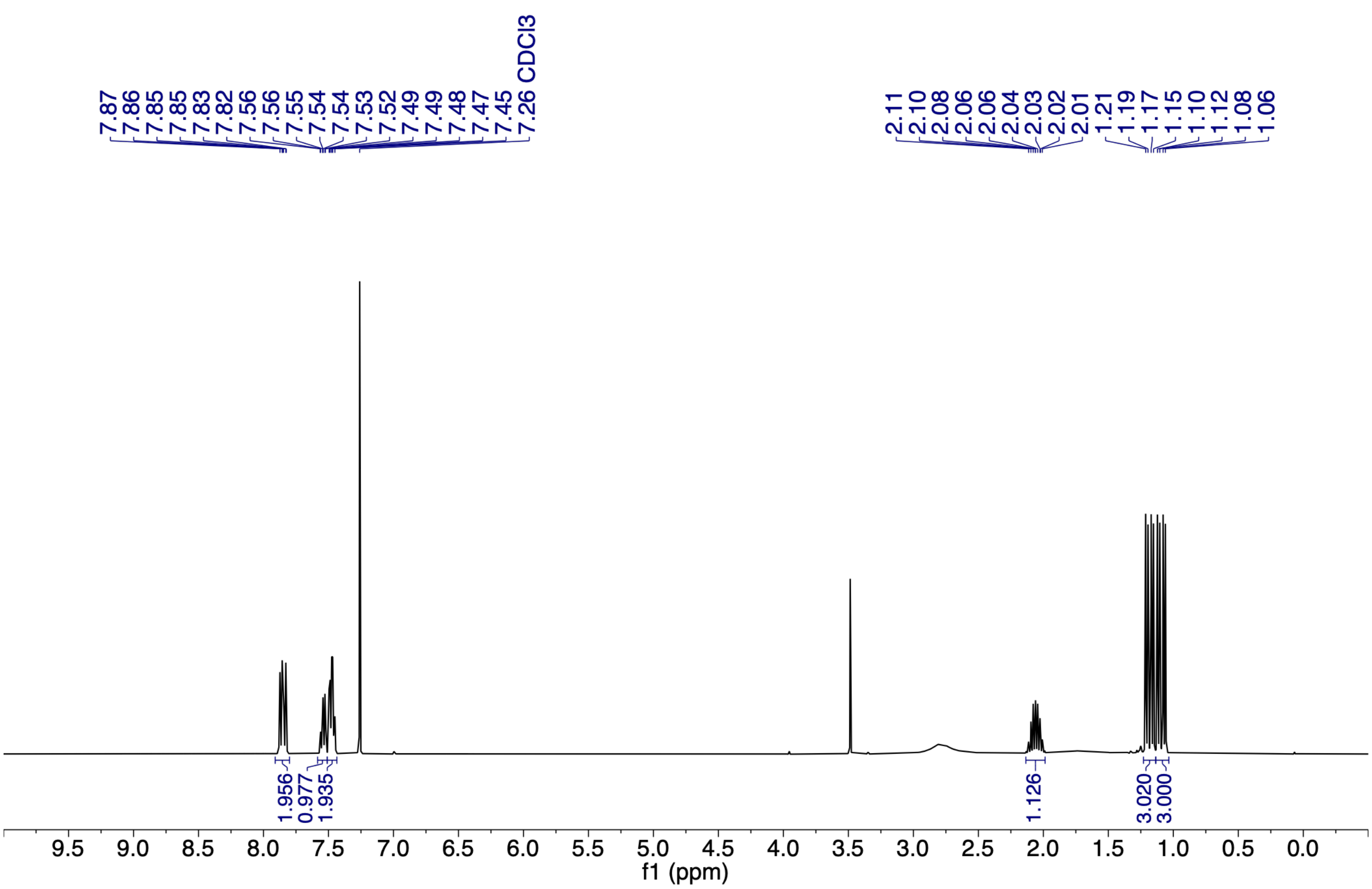

Isopropylphenylphosphinic amide (4c)

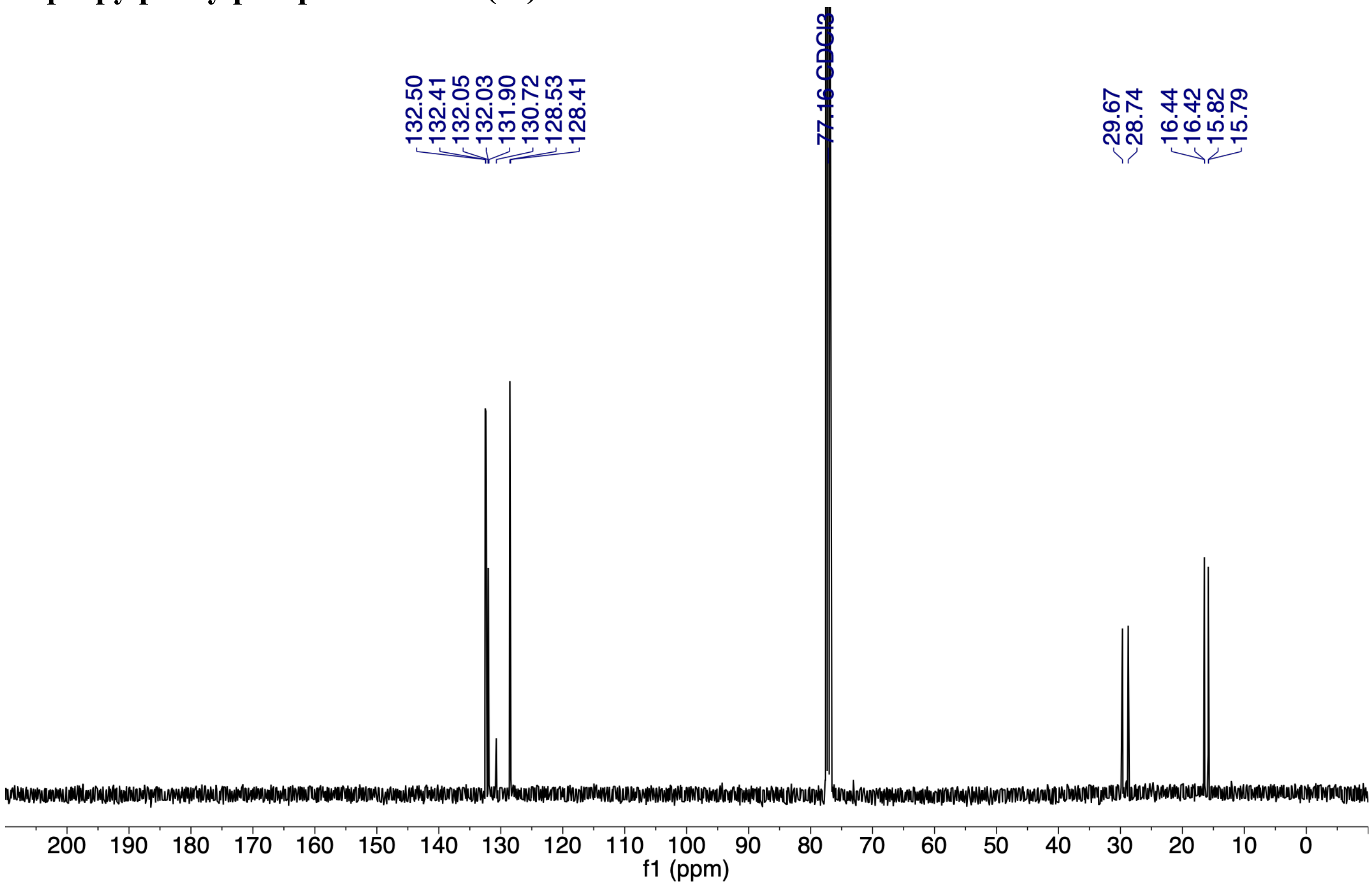

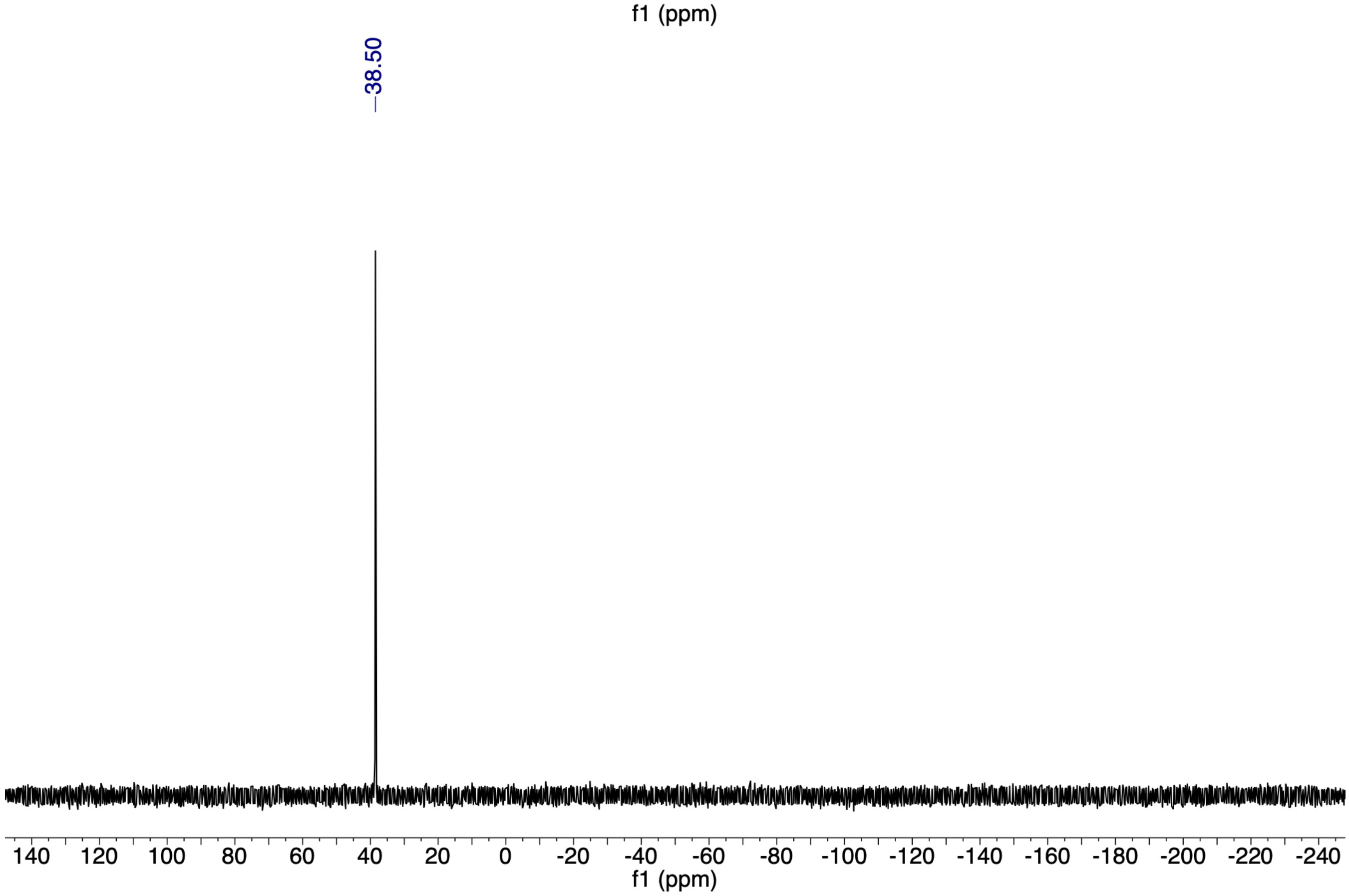

4. Discovery of Initial Activity and Control Reactions

4.1 Initial activity screening with $Ph_2P(O)H$ and EDA

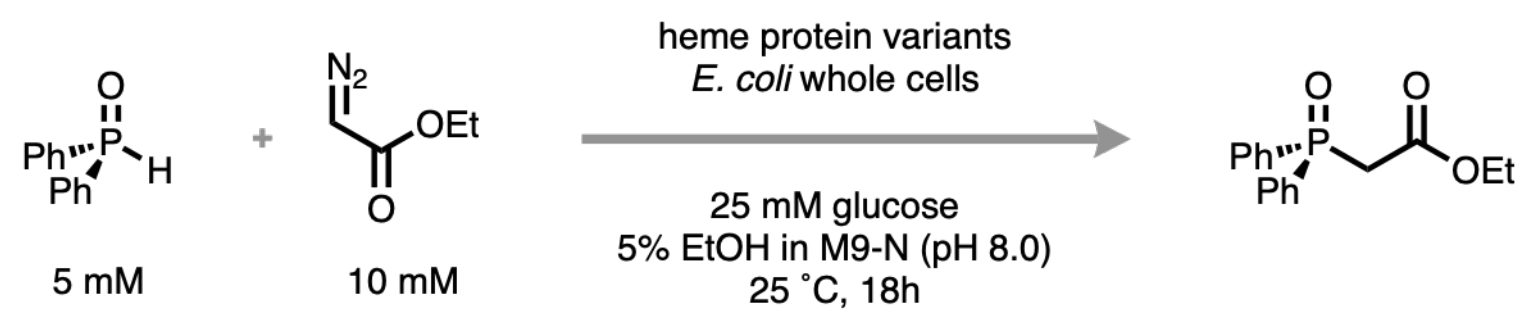

Four compilation plates drawn from our in-house engineered hemoprotein library were screened under whole-cell conditions for initial P–H insertion activity. Protoglobins were prioritized as the engineering scaffold owing to their exceptional thermostability and compact size relative to P450/P411 enzymes, properties that facilitate directed evolution campaigns by improving expression, stability under screening conditions, and tolerance to active-site mutations. The screen included wild-type and engineered variants of *Ape*Pgb bearing diverse combinations of active-site mutations at positions including residues responsible for axial ligation, distal pocket geometry, and substrate access tunnel architecture. The collection also included *Rma*Cyt variants evolved for carbene transfer reactions, as well as wild-type and engineered variants from eight additional protoglobin-bearing organisms, including Methanobacterium arcticum (*Ma*), Caldanaerobacter subterraneus (*Cth*), Methylacidiphilum infernorum (*Pfe*), Thermacetogenium phaeum (*Tam*), Thermodesulfobacterium commune (*Tda*), Thermosulfurimonas dismutans (*Thu*), Thermodesulforhabdus norvegica (*Tpe*), and Thermoanaerobacter aromatica (*Tar*).

The first collection (P1) consisted of engineered protoglobin carbene transferases designed for cyclopropanation of unsaturated exocyclic N-heterocycles.[19] The second collection (P2) consisted of a more diverse set of protoglobins, including wild types, carbene transferases designed for the formation of cis-trifluoromethyl-substituted cyclopropanes, one-carbon ring expansion of aziridines to azetidines, and C–H amination using hydroxylamine.[20–22] The third collection (P3) featured a broad collection of engineered protoglobins evolved for carbene and nitrene transfer reactions. Last, the fourth collection (P4) consisted of engineered protoglobin nitrene transferases designed for alpha amino ester formation by C–H insertion.[23] All libraries, organized in 96-well plates, were expressed in *E. coli* and subjected to high-throughput screening experimentation under whole-cell conditions using $Ph_2P(O)H$ and EDA. The activity was measured by subjecting it to reverse-phase HPLC-MS analysis. The top variants from each plate – P1-C3, P1-C8, P2-D6, P4-G2, and P4-F11 – were chosen for validation and control reactions.

Reverse Phase HPLC-MS: Agilent Poroshell 120 EC-C18, 1.5 mL/min, [A] 0.1% AcOH in Water, [B] 0.1% AcOH in Acetonitrile, scan and single ion monitoring $[M+H]^+$, gradient: 0–0.4 min: 5–50% B; 0.4––1.4 min: 50–75% B; 1.40–1.45 min: 75-95% B; 1.45–1.80 min: 95% B; 1.0–1.85 min: 95–5% B; 1.85–2.20 min: 5% B.

Table S13: Carbene reactivity compilation plate (P1).

	1	2	3	4	5	6	7	8	9	10	11	12
**A**	7E+03	2E+05	2E+05	1E+05	1E+05	5E+05	6E+05	4E+05	4E+05	2E+05	6E+05	3E+05
**B**	6E+05	5E+05	4E+05	2E+05	2E+05	1E+05	8E+04	6E+05	6E+05	4E+05	6E+04	6E+05
**C**	8E+05	7E+05	8E+05	7E+05	4E+05	4E+05	3E+05	1E+06	8E+05	4E+05	2E+05	2E+05
**D**	1E+05	5E+05	1E+05	8E+04	7E+04	9E+04	9E+04	1E+05	9E+04	9E+04	1E+05	2E+05
**E**	1E+05	7E+04	3E+05	1E+05	7E+04	8E+04	6E+04	7E+04	1E+05	1E+05	8E+04	1E+05
**F**	1E+05	9E+04	2E+05	2E+05	2E+05	1E+05	2E+05	7E+04	2E+05	1E+05	8E+04	1E+05
**G**	1E+05	4E+04	2E+04	9E+04	3E+04	2E+04	7E+04	4E+04	1E+05	1E+05	9E+04	1E+04
**H**	7E+04	7E+04	7E+04	1E+05	4E+04	4E+04	5E+04	4E+04	3E+04	4E+04	2E+05	2E+05

Table S14: Protoglobin compilation plate (P2).

	1	2	3	4	5	6	7	8	9	10	11	12
**A**	1E+05	1E+05	8E+04	1E+05	1E+05	9E+04	1E+05	1E+05	9E+04	1E+05	2E+05	3E+04
**B**	1E+05	1E+05	1E+05	2E+05	1E+05	1E+05	1E+05	1E+05	2E+05	1E+05	2E+05	2E+04
**C**	1E+04	1E+05	1E+05	9E+04	1E+05	8E+04	2E+05	9E+04	1E+05	9E+04	2E+05	5E+05
**D**	2E+05	9E+04	1E+05	7E+04	6E+04	1E+06	3E+05	8E+04	8E+04	8E+04	8E+04	6E+05
**E**	2E+05	2E+05	9E+04	1E+05	2E+05	1E+05	9E+04	9E+04	1E+05	2E+05	9E+04	6E+05
**F**	2E+05	3E+05	1E+05	2E+05	1E+05	1E+05	2E+05	1E+05	1E+05	2E+05	5E+05	6E+05
**G**	9E+04	3E+05	1E+05	1E+05	1E+05	3E+05	1E+05	1E+05	9E+04	2E+05	2E+05	3E+05
**H**	2E+05	6E+04	6E+04	8E+04	4E+04	4E+04	5E+04	5E+04	6E+04	6E+04	1E+05	1E+05

Table S15: Carbene/nitrene reactivity compilation plate (P3).

	1	2	3	4	5	6	7	8	9	10	11	12
**A**	7E+04	1E+05	1E+05	7E+04	6E+04	8E+03	4E+03	7E+04				
**B**	9E+04	5E+04	7E+04	1E+05	5E+04	4E+04	5E+03	3E+03				
**C**	9E+04	1E+04	7E+04	1E+05	6E+04	5E+04	7E+03	3E+03				
**D**	9E+04	1E+05	2E+05	1E+05	1E+05	8E+04	9E+04	8E+04				
**E**	4E+04	1E+05	9E+04	1E+05	9E+04	5E+04	7E+04	2E+05				
**F**	1E+05	1E+05	7E+04	2E+05	1E+05	1E+05	1E+05	2E+04				
**G**	1E+05	2E+05	7E+04	1E+05	1E+05	1E+05	7E+04	5E+04				
**H**	1E+05	1E+05	1E+05	8E+04	1E+05	1E+05	4E+05	1E+05				

Table S16: Nitrene reactivity compilation plate (P4).

	1	2	3	4	5	6	7	8	9	10	11	12
**A**		7E+04	1E+05	1E+05	2E+05	1E+05	1E+05	9E+04	9E+04	8E+04	1E+05	
**B**		2E+05	1E+05	2E+05	2E+05	3E+05	3E+05	2E+05	4E+05	6E+05	6E+05	
**C**		1E+05	3E+05	3E+05	5E+04	3E+05	2E+05	1E+05	1E+05	1E+05	5E+05	
**D**		1E+05	2E+05	7E+04	7E+04	8E+04	6E+04	9E+04	6E+04	1E+05	9E+04	
**E**		4E+04	6E+04	7E+04	8E+04	6E+04	1E+05	7E+04	1E+05	6E+04	2E+05	
**F**		8E+04	4E+04	1E+05	6E+04	7E+04	3E+05	6E+05	1E+05	2E+05	7E+05	
**G**		6E+05	2E+05	5E+05	2E+05	6E+05	3E+05	8E+04	1E+05	7E+04	2E+05	
**H**												

4.2 Validation of initial hits and control reactions

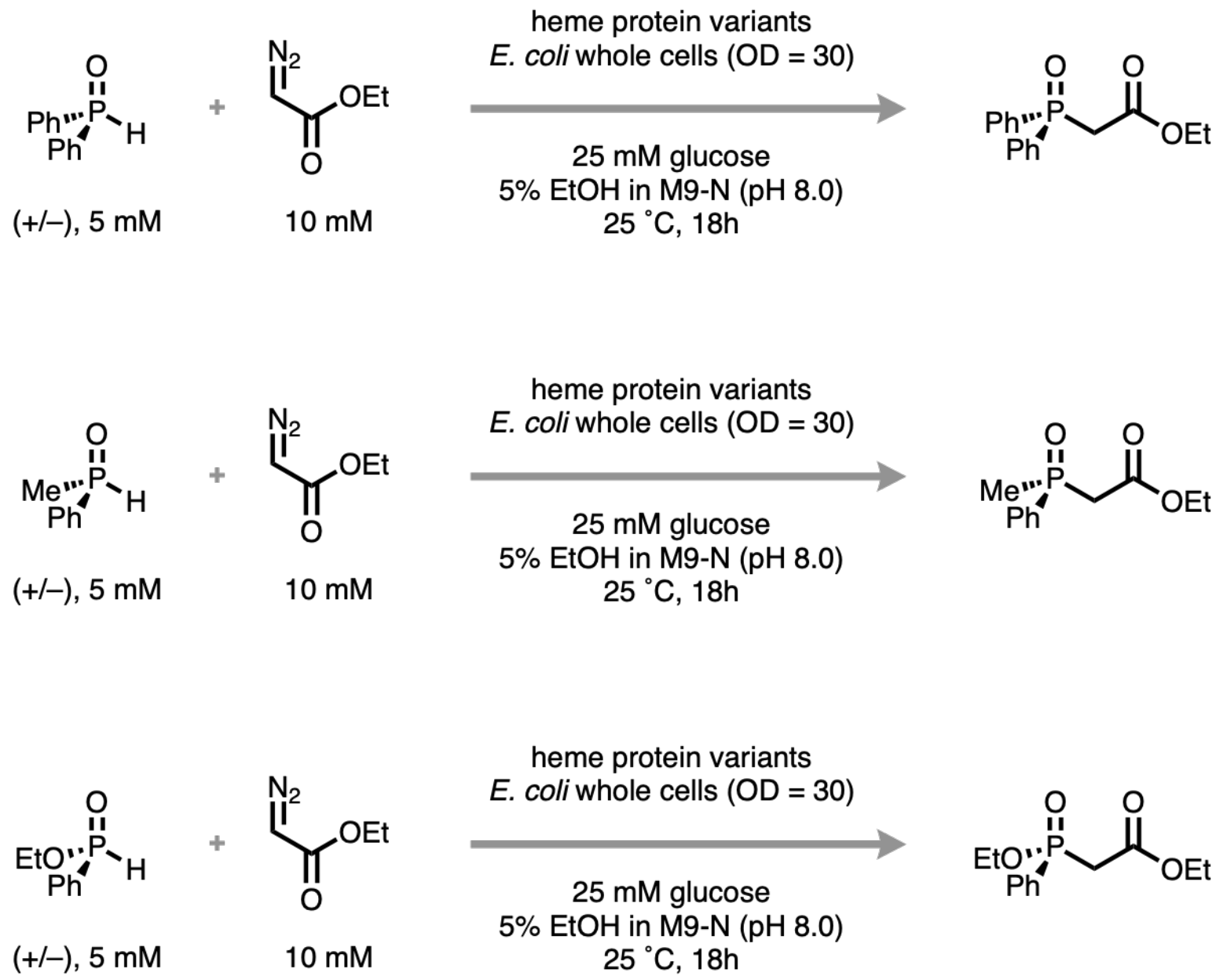

The top variants from initial screening – P1-C3, P1-C8, P2-D6, P4-G2, and P4-F11 – were chosen for validation under adjusted OD_{600} = 30 conditions across three substrates. Control reactions were also performed: 50 µM hemin (from a 10 mM stock solution, 0.1 M NaOH), 200 mM sodium dithionite (DT), 1 mg/mL bovine serum albumin (BSA). All reactions had enzyme variants that exhibited activity significantly above the hemin/DT background. In addition, $Ph_2P(O)H$ with P1-C8 exhibited a linear OD_{600} dependence on activity, confirming enzymatic activity.

P4-G2 and P4-F11, variants initially designed for alpha amino ester formation by C–H insertion with PONT were chosen as the starting point for a directed evolution campaign as they exhibited the highest activity across all three substrates.

Reverse Phase HPLC-MS: Agilent Poroshell 120 EC-C18, 1.5 mL/min, [A] 0.1% AcOH in Water, [B] 0.1% AcOH in Acetonitrile, scan and single ion monitoring $[M+H]^+$, gradient: 0–0.4 min: 5–50% B; 0.4–1.4 min: 50–75% B; 1.40–1.45 min: 75–95% B; 1.45–1.80 min: 95% B; 1.0–1.85 min: 95–5% B; 1.85–2.20 min: 5% B.

Note: The reaction replicates of P4-F11 with PhMeP(O)H (**1a**) and Ph(OEt)P(O)H (**2a**) were combined and quenching solvent (MeCN) removed by blowing with air. The samples were extracted with 200 µL EtOAc and organic layer analyzed by normal phase chiral HPLC. The product signal was insufficient to determine enantioselectivity and, thus, could not be used as a factor to determine the optimal variant to move forward with.

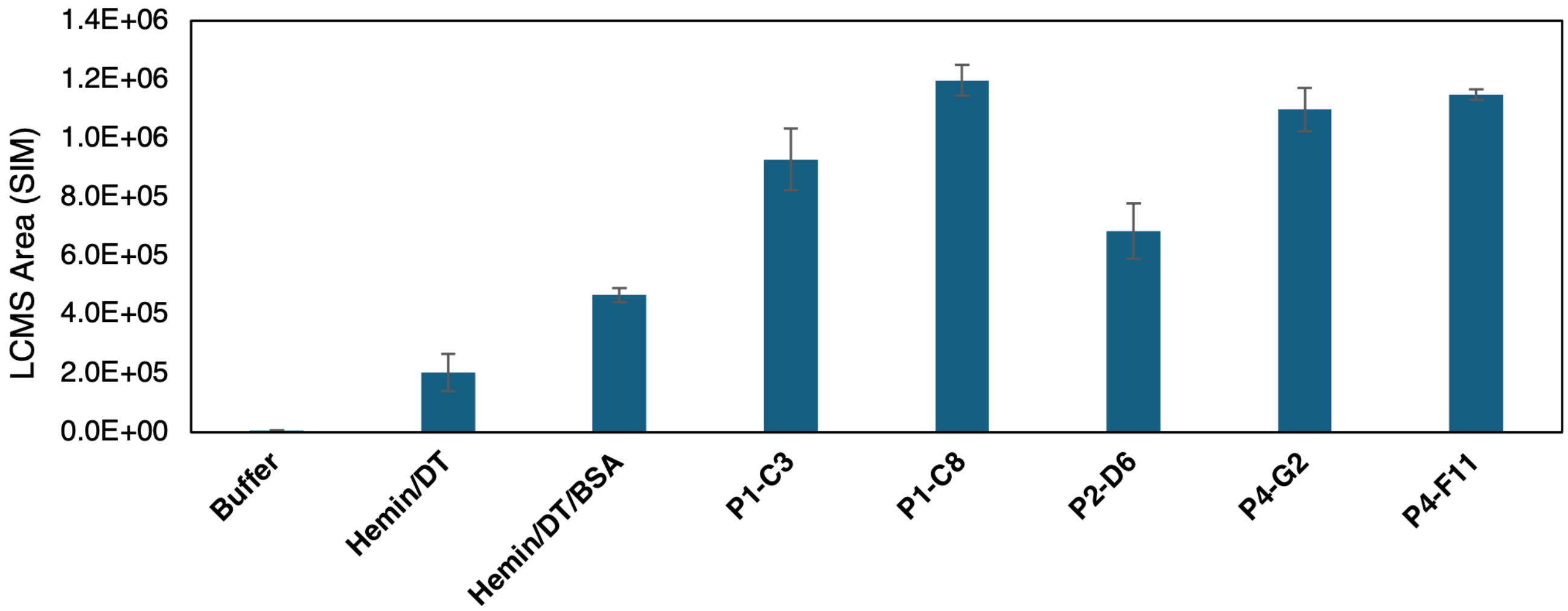

Figure S1: Validation of $Ph_2P(O)H$ reactivity with EDA.

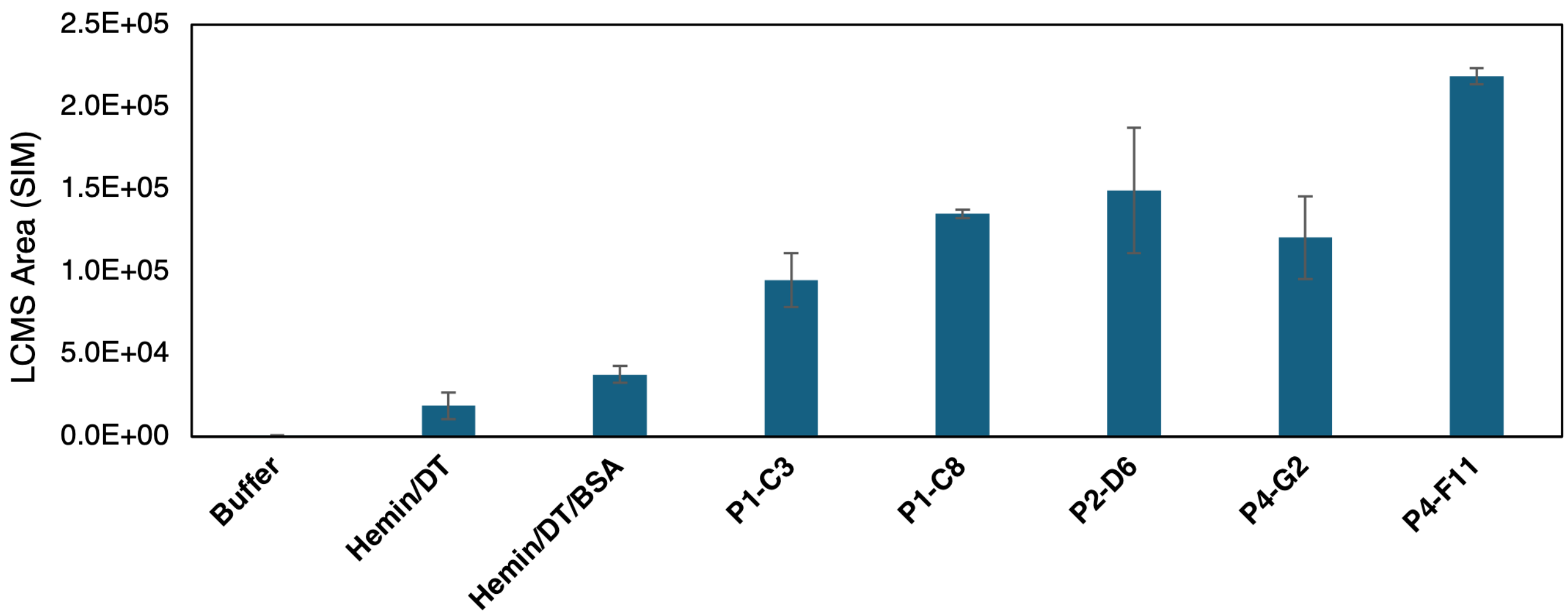

Figure S2: Validation of PhMeP(O)H (**1a**) reactivity with EDA.

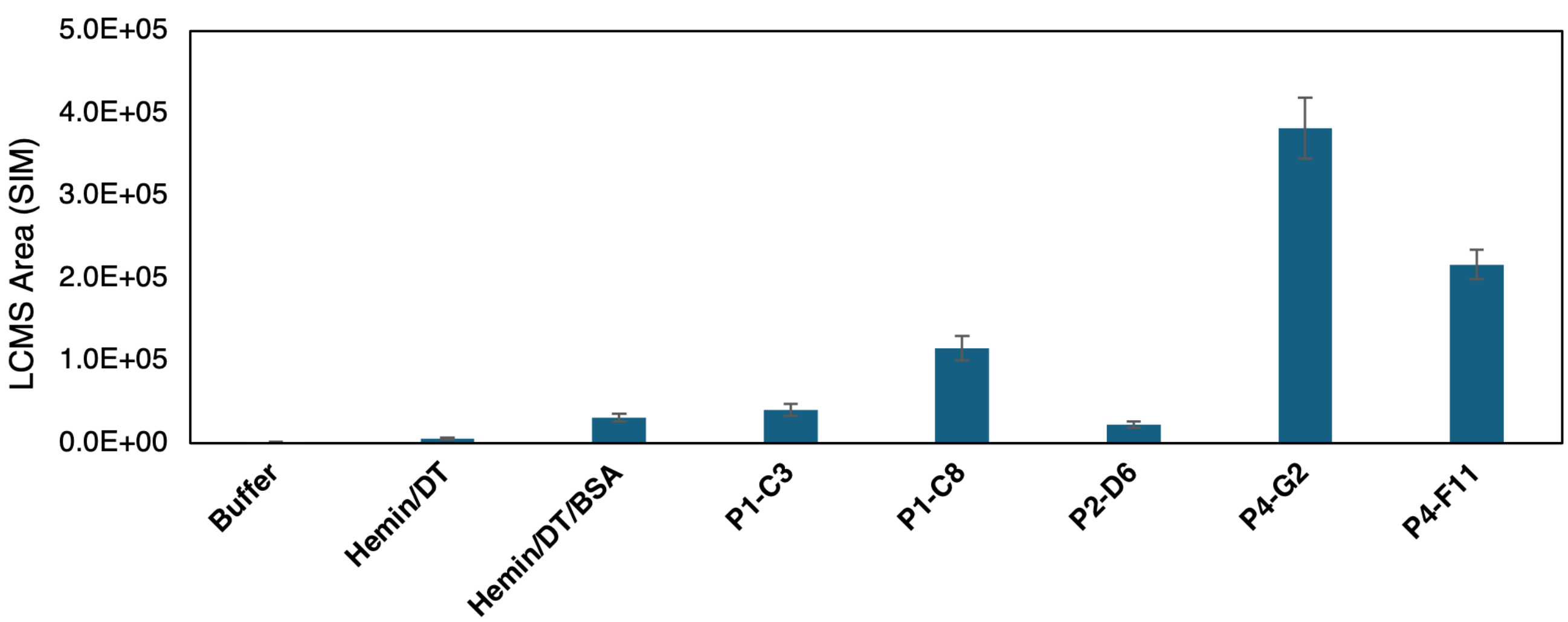

Figure S3: Validation of Ph(OEt)P(O)H (**2a**) reactivity with EDA.

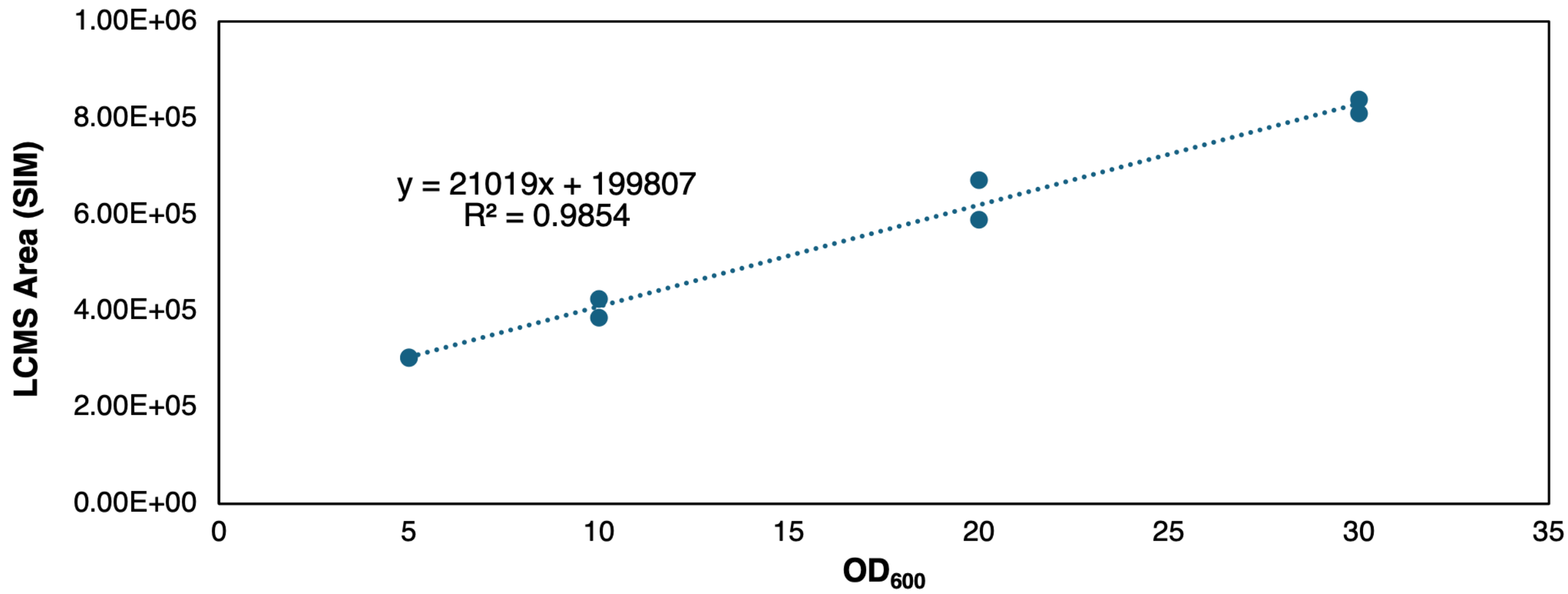

Figure S4: OD_{600} dependence of P1-C8 activity with $Ph_2P(O)H$ and EDA.

4.3 Evaluating reactivity with WT *Ape*Pgb and known enzymes for X–H insertion

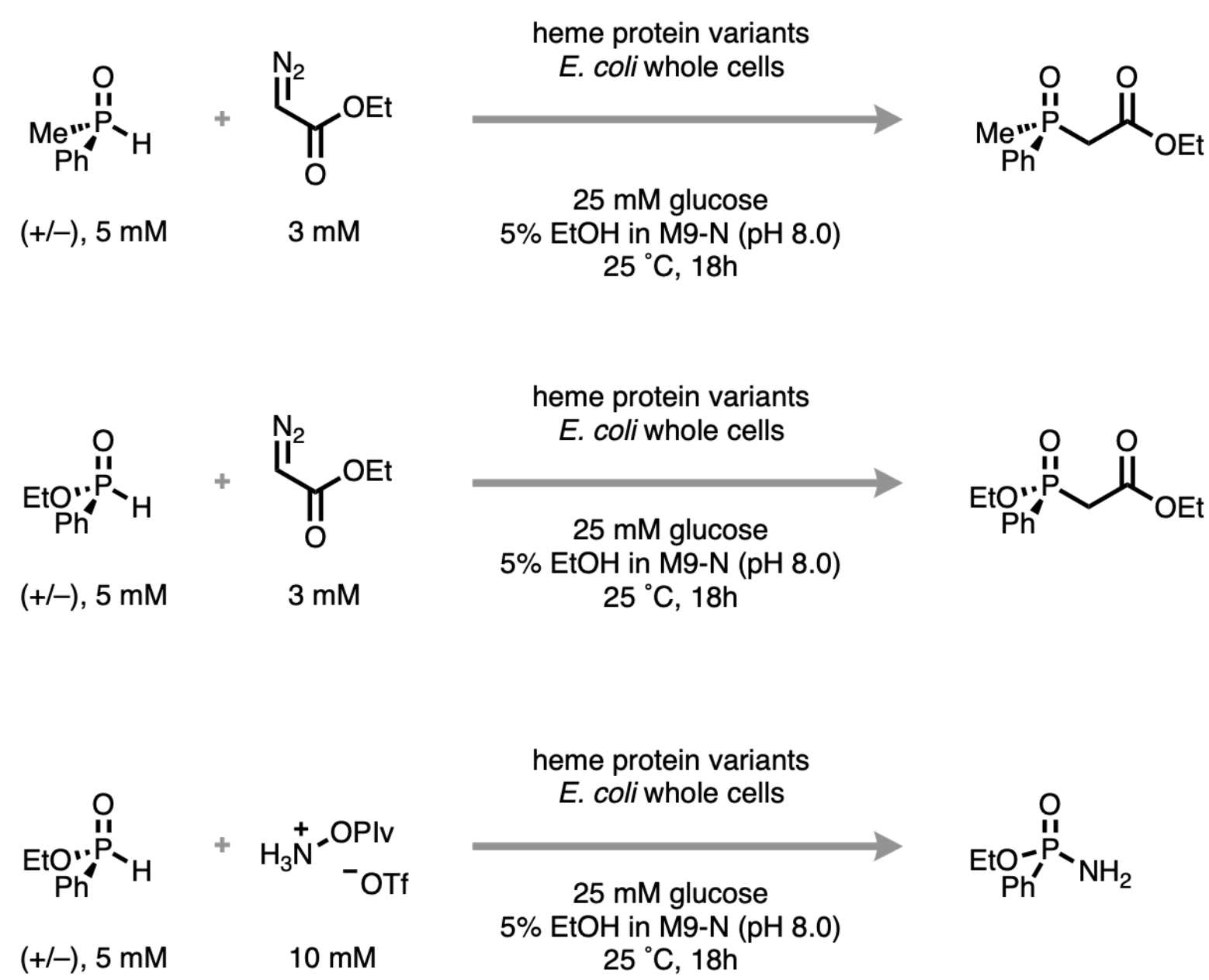

The model reactions were evaluated under control reaction conditions, initial variants, evolved variants, WT *Ape*Pgb, and heme enzymes previously engineered by the Arnold lab to perform main group (X–H) insertion: B–H insertion (4825, 4836),[24,25] Si–H insertion (4830),[26] and N–H insertion (4978).[27]

Across the reactions, WT *Ape*Pgb and heme enzymes previously engineered by the Arnold lab to perform main group (X–H) insertion all performed lower than the hemin background.

Reverse Phase HPLC-MS: Agilent Poroshell 120 EC-C18, 1.5 mL/min, [A] 0.1% AcOH in Water, [B] 0.1% AcOH in Acetonitrile, scan and single ion monitoring $[M+H]^+$, gradient: 0–0.4 min: 5–50% B; 0.4–1.4 min: 50–75% B; 1.40–1.45 min: 75–95% B; 1.45–1.80 min: 95% B; 1.0–1.85 min: 95–5% B; 1.85–2.20 min: 5% B.

Table S17: Evaluating enzymatic reactivity with WT *Ape*Pgb and previously disclosed enzymes.

**Condition/enzyme identity**	**PhMeP(O)H (1a) + EDA**		**Ph(OEt)P(O)H (2a) + EDA**		**Ph(OEt)P(O)H (2a) + PONT**	
	LCMS Area (SIM)	Activity relative to **G0**	LCMS Area (SIM)	Activity relative to **G0**	LCMS Area (SIM)	Activity relative to **G4**
Buffer	384	0.1	106	0.0		0.0
Hemin/DT	3522	0.5	1121	0.1		0.0
G0	7663	1.0	15576	1.0	1244	0.1
G4	154276	20.1	10165	0.7	24844	1.0
G5OEt	132480	17.3	162489	10.4	1724	0.1
G7N	121004	15.8	36983	2.4	32693	1.3
*Ape*Pgb	962	0.1	241	0.0		0.0
4825 (BOR-R1)	1084	0.1	325	0.0		0.0
4830 (TDE)	1067	0.1	205	0.0	112	0.0
4836 (BOR-CF3)	1016	0.1	194	0.0	107	0.0
4978 (P411-L7_FL)	1426	0.2	117	0.0	150	0.0

5. Directed Evolution

The yield and stereoselectivity data presented in this section were collected according to the procedure detailed in Section 2.6. All variants for a given substrate campaign were run in parallel. For each variant, three technical replicates were carried out (three data points for each variant) in addition to a replicate for determination of enantioselectivity.

A summary of the mutations accumulated throughout the lineage and the mutagenesis method for each round of evolution is shown in **Table S18**.

Table S18: Evolved variants for carbene and nitrene insertion into P–H bonds.

Variant	Arnold Lab ID	Mutations as compared to		Mutagenesis method
G0	5462	*Ape*Pgb wt	+ K36E, C45A, W59A, Y60G, G93G, I97V, H136N, I137T, F145G, F156L, K159E, F175L	–
G1	5463	G0	+V63A	epPCR
G2	5464	G1	+ G93C	epPCR
G3	5465	G2	+ P7S, E80A, L156F, E159K, W195R	stEP (epPCR)
G4	5466	G3	+ C93I	SSM (69, 93, 178)
G4OEt	5467	G3	+ G94V, I149L	stEP (epPCR + SSM 69, 71, 73, 149)
G5OEt	5468	G4OEt	+ I19V, L33F	stEP (epPCR)
G5.1	5469	G4OEt	+ A59M	SSM (59, 86, 93, 145, 149, and 181)
G6.1	5470	G5.1	+ G145E	SSM (55, 60, 63, 73, 86, 89, 93, 145, 149, 185)
G7.1	5471	G6.1	+ A63L	SSM (55, 60, 63, 73, 86, 89, 93, 185)
G8.1	5472	G7.1	+ R13H, Q181R	epPCR
G9.1	5473	G8.1	+ L149F	SSM (59, 60, 73, 86, 93, 139
G10.1	5474	G9.1	+ L86M, R181C	SSM (56, 60, 72, 73, 85, 86, 90, 97, 181) → SSM of L86M @ 181 and SSM of R181C @ 86
G11.1	5475	G10.1	+ V116I	SSM (60, 116) and epPCR
G4-I93S	5476	G4	+ I93S	SSM (see SI 5.3)
G4-I93T	5477	G4	+ I93T	SSM (see SI 5.3)
G5OEt-A59I	5478	G5OEt	+ A59I	SSM (see SI 5.3)
G5OEt-A59L	5479	G5OEt	+ A59L	SSM (see SI 5.3)
G5OEt-A59T	5480	G5OEt	+ A59T	SSM (see SI 5.3)
G5OEt-L86Q	5481	G5OEt	+ L86Q	SSM (see SI 5.3)
G5OEt-C93S	5482	G5OEt	+ C93S	SSM (see SI 5.3)
G11.1-M59L	5483	G11.1	+ M59L	SSM (see SI 5.3)
G11.1-F149V	5484	G11.1	+ F149V	SSM (see SI 5.3)
G11.1-C181R	5485	G11.1	+ C181R	SSM (see SI 5.3)
G5N	5486	G4	+ I93L	SSM (59, 86, 93, 145, 149)
G6N	5487	G5N	+ P188R	SSM (73, 89, 181, 185, 188)
G7N	5488	G6N	+ L58Q, Y71W	epPCR
G6N.1	5489	G5N	+ G145A, I149M	SSM (145/149 double site)

5.1 Carbene P–H Insertion: PhMeP(O)H (1a)

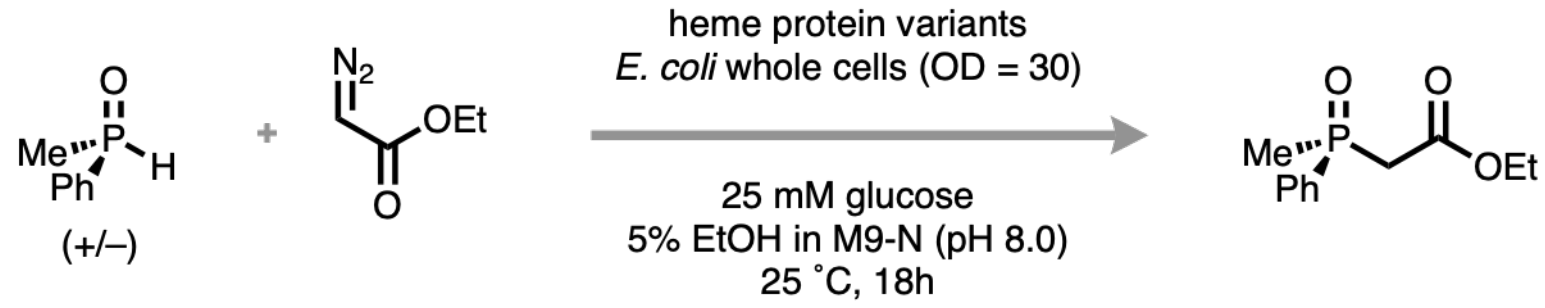

Reactions were performed according to the validation procedure (three biological replicates, with 2–3 technical replicates for assay yield and one technical replicate for enantioselectivity).

Because variants G4, G4OEt, G5OEt, G6.1, and G11.1 were lysed to test reactions ran with clarified lysate instead of whole cells, when could determine the heme concentration using the hemochrome assay which serves as an effective concentration of active enzyme. Within these variants, heme concentration of the clarified lysate varied from 11 to 19 µM. Lysate reactions were performed with 1 equiv of sodium dithionite (as a solution in dH_2O) with respect to [P].

Reverse Phase HPLC-MS: Agilent Poroshell 120 EC-C18, 1.5 mL/min, [A] 0.1% AcOH in Water, [B] 0.1% AcOH in Acetonitrile, scan and single ion monitoring $[M+H]^+$, gradient: 0-0.2 min: 5-50% B; 0.2-0.4 min: 5–20% B; 0.4–2.5 min: 20–50% B; 2.5–2.6 min: 50–95% B; 2.6–3.0 min: 95% B; 3.0–3.1 min: 95–5% B; 3.1–3.5 min: 5% B.

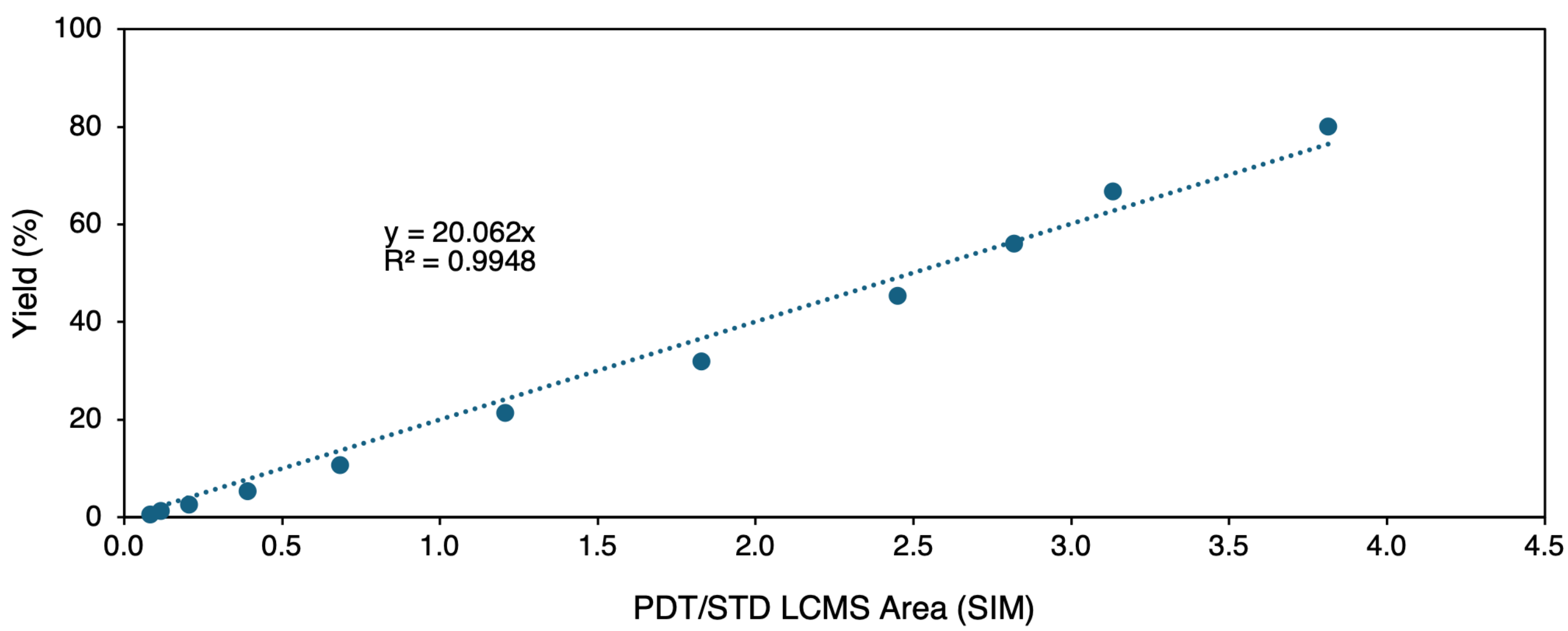

Figure S5: Calibration curve using authentic sample and papaverine as an external standard.

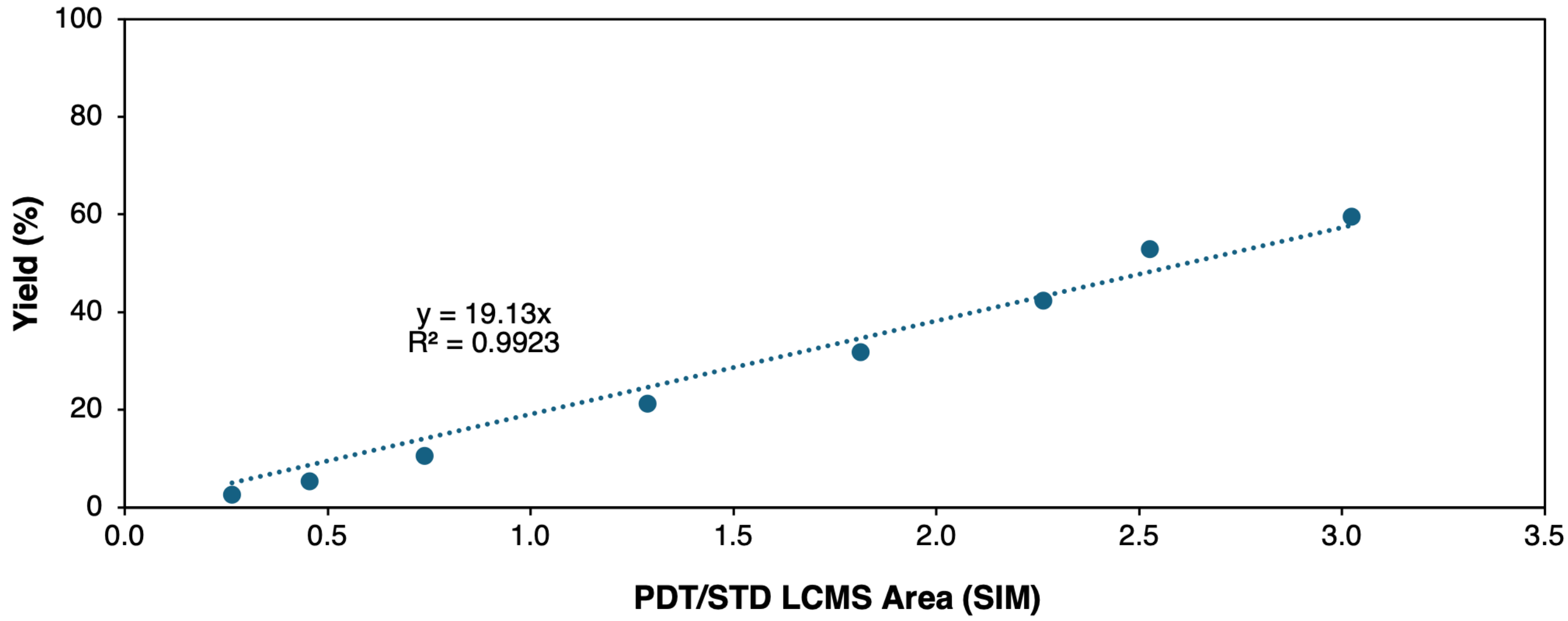

Figure S6: Calibration curve using authentic sample and papaverine as an external standard.

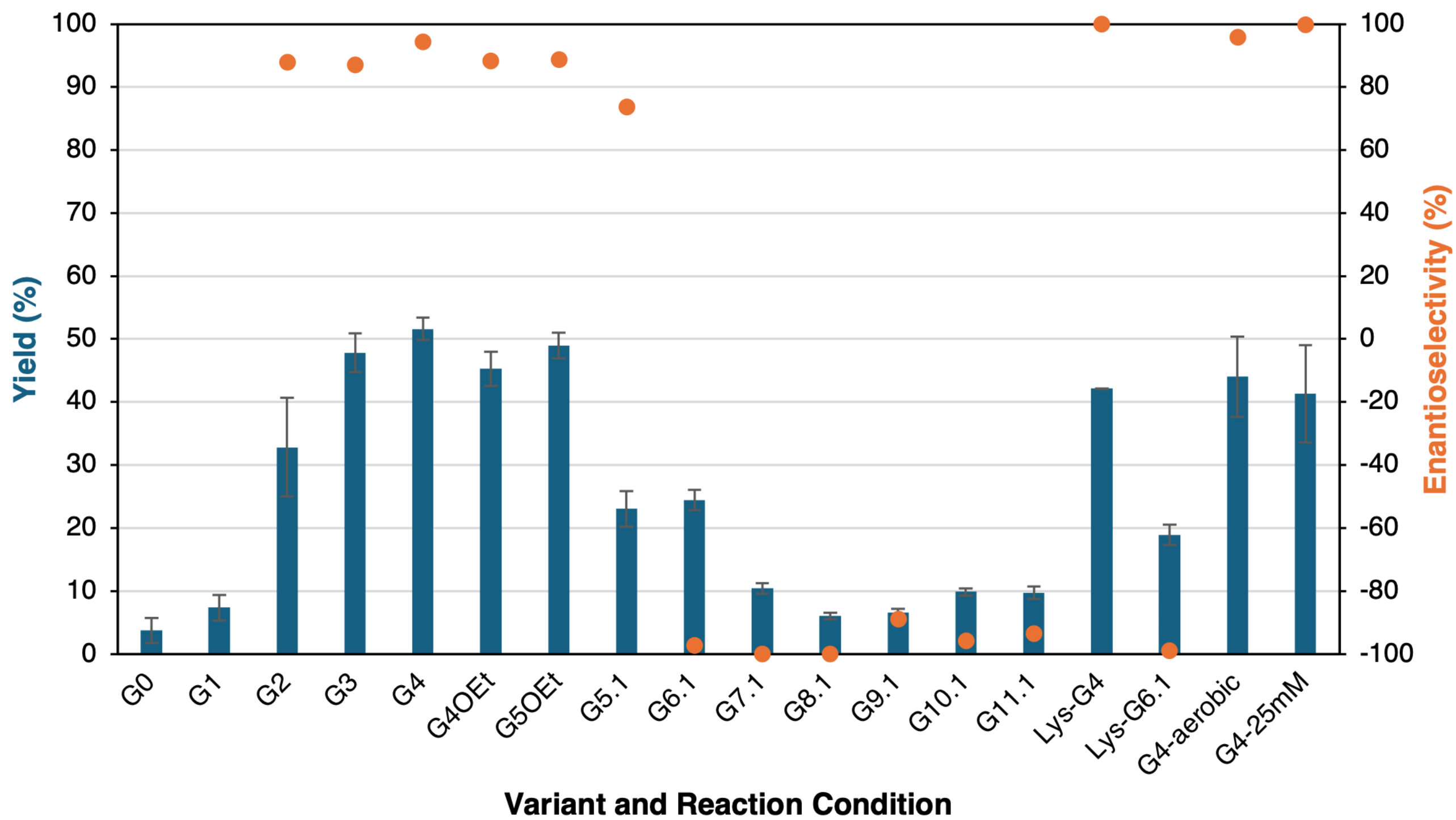

Figure S7: Visualization of the yield and enantioselectivity of variants and selected reaction conditions for the reaction of PhMeP(O)H and EDA.

Table S19: Yield and enantioselectivity of variants and selected reaction conditions for the reactions of PhMeP(O)H and EDA.

Variant/conditions	Avg. Yield (%)	Std Dev Yield (%)	Avg. TTN (%)	Avg. E.E. (%)	Std Dev E.E. (%)
G0	4	2			
G1	7	2			
G2	33	8		88	2
G3	48	3		87	0
G4	52	2	185	94	1
G4OEt	45	3	134	88	1
G5OEt	49	2	146	89	1
G5.1	23	3		74	1
G6.1	24	2	126	-97	0
G7.1	10	1		-100	0
G8.1	6	1		-100	0
G9.1	7	1		-89	0
G10.1	10	1		-96	4
G11.1	10	1	35	-94	0
Lys-G4	42	0	150	100	0
Lys-G6.1	19	2		-99	2
G4-aerobic	44	6	157	96	0
G4-25mM	41	8	731	100	1

*G6.1-M59A was a variant created during curation of the SSM compilation plate and TIC was 0.46x of G6.1 (E.E. was not determined)

5.2 Carbene P–H Insertion: Ph(OEt)P(O)H (2a)

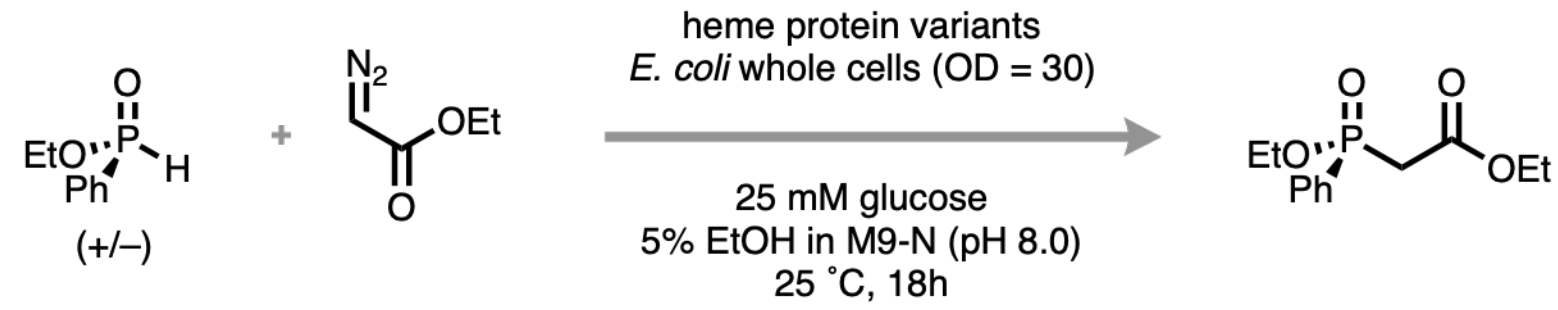

Reactions were performed according to the validation procedure (three technical replicates for assay yield; one technical replicate for enantioselectivity).

Because variants G4, G4OEt, G5OEt, G6.1, and G11.1 were lysed to test reactions ran with clarified lysate instead of whole cells, when could determine the heme concentration using the hemochrome assay which serves as an effective concentration of active enzyme. Within these variants, heme concentration of the clarified lysate varied from 11 to 19 µM. Lysate reactions were performed with 1 equiv of sodium dithionite (as a solution in dH_2O) with respect to [P].

Reverse Phase HPLC-MS: Agilent Poroshell 120 EC-C18, 1.5 mL/min, [A] 0.1% AcOH in Water, [B] 0.1% AcOH in Acetonitrile, scan and single ion monitoring $[M+H]^+$, gradient: 0-0.2 min: 5-50% B; 0.2-0.4 min: 5–20% B; 0.4–2.5 min: 20–50% B; 2.5–2.6 min: 50–95% B; 2.6–3.0 min: 95% B; 3.0–3.1 min: 95–5% B; 3.1–3.5 min: 5% B.

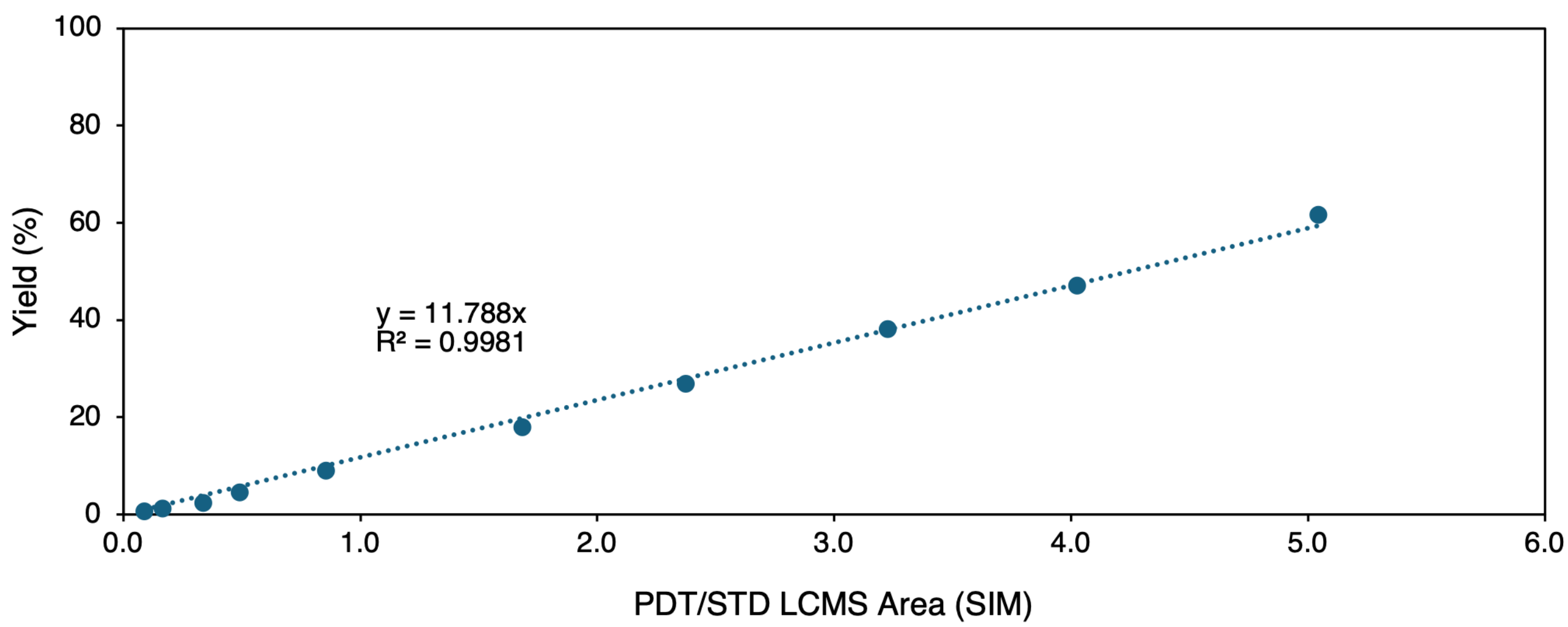

Figure S8: Calibration curve using authentic sample and papaverine as an external standard.

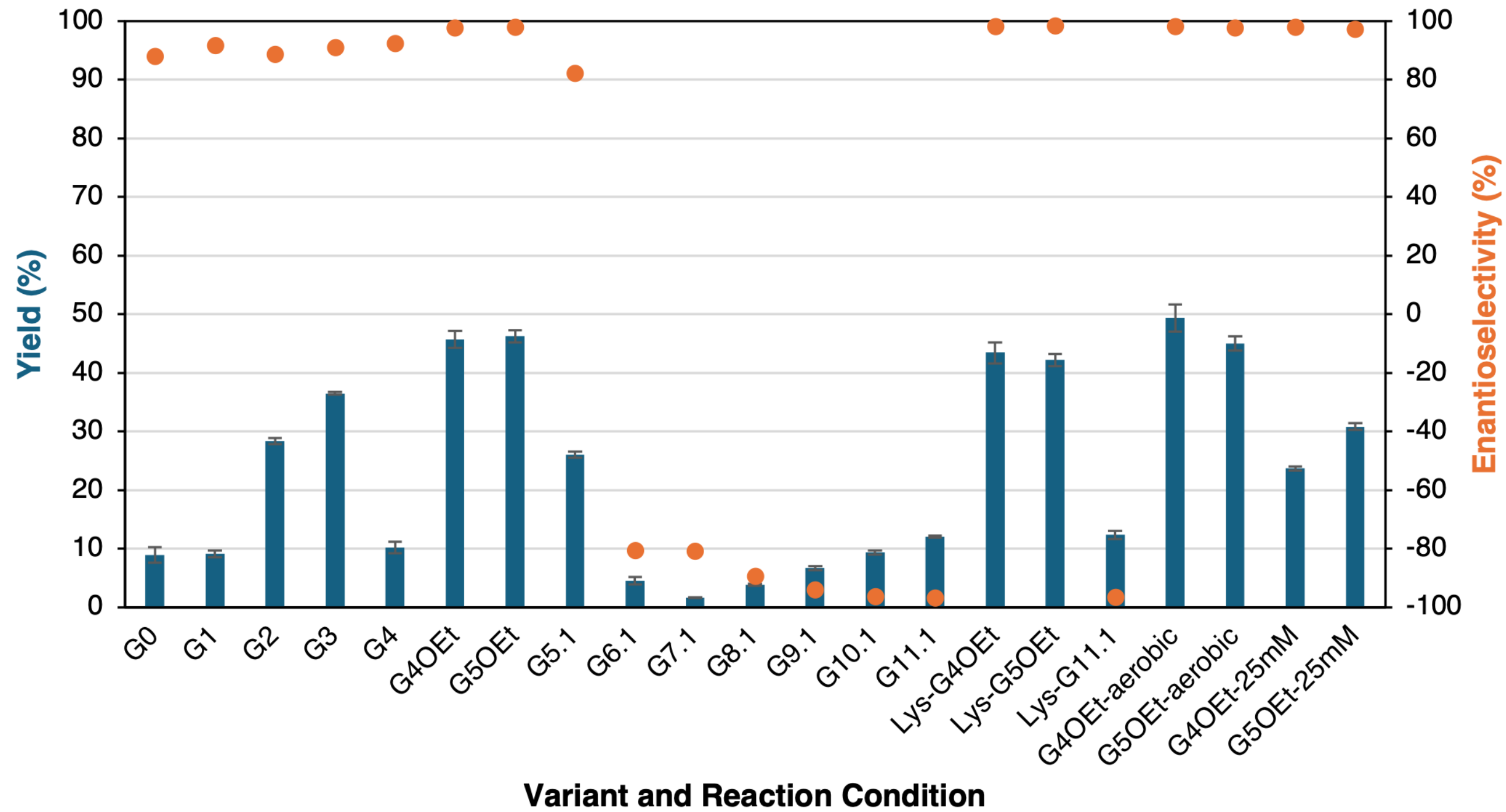

Figure S9: Visualization of the yield and enantioselectivity of variants and selected reaction conditions for the reaction of Ph(OEt)P(O)H and EDA.

Table S20: Yield and enantioselectivity of variants and selected reaction conditions for the reactions of Ph(OEt)P(O)H and EDA.

Variant/conditions	Avg. Yield (%)	Std Dev Yield (%)	Avg. TTN	E.E. (%)
G0	9	1		88
G1	9	1		92
G2	28	0		89
G3	36	0		91
G4	10	1	36	92
G4OEt	46	1	137	98
G5OEt	46	1	137	98
G5.1	26	1		82
G6.1	4	1	21	-81
G7.1	2	0		-81
G8.1	4	0		-89
G9.1	7	0		-94
G10.1	9	0		-96
G11.1	12	0	42	-97
Lys-G4OEt	43	2	128	98
Lys-G5OEt	42	1	125	98
Lys-G11.1	12	1	42	-97
G4OEt-aerobic	49	2	146	98
G5OEt-aerobic	45	1	134	98
G4OEt-25mM	24	0	358	98
G5OEt-25mM	31	1	462	97

*G6.1-M59A was a variant created during curation of the SSM compilation plate and TIC was 0.07x of G6.1 (E.E. was not determined)

5.3 SSM Compilation Plate Screening for Substrate Scope

SSM compilation plates were prepared for four optimized variants (G4, G5OEt, G6.1, and G11.1) by targeting positions selected for their significant impact on yield and/or selectivity. The SSM sites targeted were A59X, G145X, I149X, I93X, and L86X for G4; A59X, L86X, C93X, G145X, L149X, and Q181X for G5OEt; M59X, L86X, E145X, L149X, and Q181X for G6.1; and M59X, M86X, F149X, and C181X for G11.1.

Following sequencing and re-arraying, variants were initially evaluated using two model reactions: PhMeP(O)H with EDA (Tables S21-S23) and Ph(OEt)P(O)H with EDA (Tables S24-S26). Those displaying greater than 10% of parent activity (190 variants) were advanced and screened against three additional substrate-reagent combinations: Ph(OEt)P(O)H with LAD (Tables S27-S29), Ph(OEt)P(O)H with DAN (Tables S30-S32), and PhCH2CH2(OEt)P(O)H with EDA (Tables S33-S34).

Unique top-performing variants from each parent were then consolidated into a master compilation plate comprising 15x G4 (including G4OEt), 18x G5OEt, 8x G6.1, and 5x G11.1 variants. This Master Compilation Plate (P11, Table S35) was screened across all substrate-reagent combinations included in the substrate scope (Table S36), and optimal variants for each combination were subsequently validated for yield and selectivity.

Table S21: Ranked LCMS area (SIM) of the reaction of PhMeP(O)H with EDA across variants (P5).

	1	2	3	4	5	6	7	8	9	10	11	12
A		G5OEt	G6.1	G6.1-E145A	G6.1-E145C	G6.1-E145D	G6.1-E145F	G6.1-E145G	G6.1-E145I	G6.1-E145K	G6.1-E145L	G6.1-E145M
B	G6.1-E145N	G6.1-E145P	G6.1-E145Q	G6.1-E145R	G6.1-E145T	G6.1-E145V	G6.1-E145W	G6.1-E145Y	G6.1-L149A	G6.1-L149C	G6.1-L149D	G6.1-L149E
C	G6.1-L149G	G6.1-L149H	G6.1-L149I	G6.1-L149K	G6.1-L149M	G6.1-L149N	G6.1-L149Q	G6.1-L149R	G6.1-L149S	G6.1-L149V	G6.1-L149W	G6.1-L149Y
D	G6.1-L86A	G6.1-L86C	G6.1-L86D	G6.1-L86E	G6.1-L86F	G6.1-L86G	G6.1-L86H	G6.1-L86I	G6.1-L86K	G6.1-L86M	G6.1-L86P	G6.1-L86Q
E	G6.1-L86R	G6.1-L86S	G6.1-L86T	G6.1-L86V	G6.1-L86W	G6.1-L86Y	G6.1-M59A	G6.1-M59C	G6.1-M59D	G6.1-M59E	G6.1-M59F	G6.1-M59G
F	G6.1-M59H	G6.1-M59I	G6.1-M59L	G6.1-M59N	G6.1-M59P	G6.1-M59Q	G6.1-M59R	G6.1-M59T	G6.1-M59V	G6.1-M59W	G6.1-M59Y	G6.1-Q181A
G	G6.1-Q181C	G6.1-Q181D	G6.1-Q181E	G6.1-Q181F	G6.1-Q181G	G6.1-Q181H	G6.1-Q181I	G6.1-Q181K	G6.1-Q181L	G6.1-Q181M	G6.1-Q181N	G6.1-Q181R
H		G6.1-Q181S	G6.1-Q181T	G6.1-Q181V	G6.1-Q181W	G6.1-Q181Y	G5OEt	G5OEt-C93D	G5OEt-C93E	G5OEt-C93F	G5OEt-C93G	G5OEt-C93H

Table S22: Ranked LCMS area (SIM) of the reaction of PhMeP(O)H with EDA across variants (P6).

	1	2	3	4	5	6	7	8	9	10	11	12
A		G5OEt	G6.1	G5OEt-C93I	G5OEt-C93K	G5OEt-C93L	G5OEt-C93M	G5OEt-C93N	G5OEt-C93P	G5OEt-C93Q	G5OEt-C93R	G5OEt-C93S
B	G5OEt-C93V	G5OEt-C93W	G5OEt-C93Y	G5OEt-L86A	G5OEt-L86C	G5OEt-L86D	G5OEt-L86E	G5OEt-L86F	G5OEt-L86G	G5OEt-L86H	G5OEt-L86I	G5OEt-L86K
C	G5OEt-L86M	G5OEt-L86N	G5OEt-L86P	G5OEt-L86Q	G5OEt-L86S	G5OEt-L86V	G5OEt-L86W	G5OEt-L86Y	G4	G4-I149P	G4-A59D	G4-A59E
D	G4-A59F	G4-A59G	G4-A59H	G4-A59K	G4-A59L	G4-A59N	G4-A59P	G4-A59Q	G4-A59R	G4-A59S	G4-A59T	G4-A59V
E	G4-A59W	G4-A59Y	G4-G145A	G4-G145C	G4-G145E	G4-G145F	G4-G145H	G4-G145I	G4-G145L	G4-G145M	G4-G145P	G4-G145Q
F	G4-G145R	G4-G145S	G4-G145T	G4-G145V	G4-G145W	G4-G145Y	G4-I149A	G4-I149C	G4-I149E	G4-I149G	G4-I149H	G4-I149K
G	G4-I149L	G4-I149M	G4-I149N	G4-A59C	G4-I149Q	G4-I149R	G4-I149S	G4-I149T	G4-I149V	G4-I149W	G4-I149Y	G4-I93C
H		G4-I93D	G4-I93E	G4-I93F	G4-I93H	G4-I93K	G4-I93L	G4-I93M	G4-I93N	G4-I93P	G4-I93Q	G4-I93R

Table S23: Ranked LCMS area (SIM) of the reaction of PhMeP(O)H with EDA across variants (P7).

	1	2	3	4	5	6	7	8	9	10	11	12
A		G5OEt	G6.1	G4-I93S	G4-I93T	G4-I93V	G4-I93Y	G4-L86A	G4-L86C	G4-L86E	G4-L86F	G4-L86G
B	G4-L86H	G4-L86I	G4-L86K	G4-L86M	G4-L86N	G4-L86P	G4-L86Q	G4-L86R	G4-L86S	G4-L86T	G4-L86V	G4-L86W
C	G4-L86Y	G11.1-G11.1	G11.1-C181A	G11.1-C181D	G11.1-C181E	G11.1-C181F	G11.1-C181G	G11.1-C181H	G4	G11.1-C181K	G11.1-C181L	G11.1-C181M
D	G11.1-C181N	G11.1-C181P	G11.1-C181R	G11.1-C181S	G11.1-C181T	G11.1-C181V	G11.1-C181W	G11.1-C181Y	G11.1-F149C	G11.1-F149D	G11.1-F149E	G11.1-F149G
E	G11.1-F149I	G11.1-F149K	G11.1-F149L	G11.1-F149M	G11.1-F149N	G11.1-F149P	G11.1-F149Q	G11.1-F149R	G11.1-F149S	G11.1-F149V	G11.1-F149W	G11.1-F149Y
F	G11.1-M59A	G11.1-M59C	G11.1-M59D	G11.1-M59E	G11.1-M59G	G11.1-M59H	G11.1-M59I	G11.1-M59K	G11.1-M59L	G11.1-M59N	G11.1-M59P	G11.1-M59Q
G	G11.1-M59R	G11.1-M59S	G11.1-M59T	G11.1-M59V	G11.1-M59W	G11.1-M59Y	G11.1-M86A	G11.1-M86C	G11.1-M86D		G11.1-M86F	G11.1-M86G
H		G11.1-M86H	G11.1-M86I	G11.1-M86K	G11.1-M86L	G11.1-M86Q	G11.1-M86R	G11.1-M86S	G11.1-M86T	G11.1-M86V	G11.1-M86W	G11.1-M86E

Table S24: Ranked LCMS area (SIM) of the reaction of PhOEtP(O)H with EDA across variants (P5).

	1	2	3	4	5	6	7	8	9	10	11	12
A		G5OEt	G6.1	G6.1-E145A	G6.1-E145C	G6.1-E145D	G6.1-E145F	G6.1-E145G	G6.1-E145I	G6.1-E145K	G6.1-E145L	G6.1-E145M
B	G6.1-E145N	G6.1-E145P	G6.1-E145Q	G6.1-E145R	G6.1-E145T	G6.1-E145V	G6.1-E145W	G6.1-E145Y	G6.1-L149A	G6.1-L149C	G6.1-L149D	G6.1-L149E
C	G6.1-L149G	G6.1-L149H	G6.1-L149I	G6.1-L149K	G6.1-L149M	G6.1-L149N	G6.1-L149Q	G6.1-L149R	G6.1-L149S	G6.1-L149V	G6.1-L149W	G6.1-L149Y
D	G6.1-L86A	G6.1-L86C	G6.1-L86D	G6.1-L86E	G6.1-L86F	G6.1-L86G	G6.1-L86H	G6.1-L86I	G6.1-L86K	G6.1-L86M	G6.1-L86P	G6.1-L86Q
E	G6.1-L86R	G6.1-L86S	G6.1-L86T	G6.1-L86V	G6.1-L86W	G6.1-L86Y	G6.1-M59A	G6.1-M59C	G6.1-M59D	G6.1-M59E	G6.1-M59F	G6.1-M59G
F	G6.1-M59H	G6.1-M59I	G6.1-M59L	G6.1-M59N	G6.1-M59P	G6.1-M59Q	G6.1-M59R	G6.1-M59T	G6.1-M59V	G6.1-M59W	G6.1-M59Y	G6.1-Q181A
G	G6.1-Q181C	G6.1-Q181D	G6.1-Q181E	G6.1-Q181F	G6.1-Q181G	G6.1-Q181H	G6.1-Q181I	G6.1-Q181K	G6.1-Q181L	G6.1-Q181M	G6.1-Q181N	G6.1-Q181R
H		G6.1-Q181S	G6.1-Q181T	G6.1-Q181V	G6.1-Q181W	G6.1-Q181Y	G5OEt	G5OEt-C93D	G5OEt-C93E	G5OEt-C93F	G5OEt-C93G	G5OEt-C93H

Table S25: Ranked LCMS area (SIM) of the reaction of PhOEtP(O)H with EDA across variants (P6).

	1	2	3	4	5	6	7	8	9	10	11	12
A		G5OEt	G6.1	G5OEt-C93I	G5OEt-C93K	G5OEt-C93L	G5OEt-C93M	G5OEt-C93N	G5OEt-C93P	G5OEt-C93Q	G5OEt-C93R	G5OEt-C93S
B	G5OEt-C93V	G5OEt-C93W	G5OEt-C93Y	G5OEt-L86A	G5OEt-L86C	G5OEt-L86D	G5OEt-L86E	G5OEt-L86F	G5OEt-L86G	G5OEt-L86H	G5OEt-L86I	G5OEt-L86K
C	G5OEt-L86M	G5OEt-L86N	G5OEt-L86P	G5OEt-L86Q	G5OEt-L86S	G5OEt-L86V	G5OEt-L86W	G5OEt-L86Y	G4	G4-I149P	G4-A59D	G4-A59E
D	G4-A59F	G4-A59G	G4-A59H	G4-A59K	G4-A59L	G4-A59N	G4-A59P	G4-A59Q	G4-A59R	G4-A59S	G4-A59T	G4-A59V
E	G4-A59W	G4-A59Y	G4-G145A	G4-G145C	G4-G145E	G4-G145F	G4-G145H	G4-G145I	G4-G145L	G4-G145M	G4-G145P	G4-G145Q
F	G4-G145R	G4-G145S	G4-G145T	G4-G145V	G4-G145W	G4-G145Y	G4-I149A	G4-I149C	G4-I149E	G4-I149G	G4-I149H	G4-I149K
G	G4-I149L	G4-I149M	G4-I149N	G4-A59C	G4-I149Q	G4-I149R	G4-I149S	G4-I149T	G4-I149V	G4-I149W	G4-I149Y	G4-I93C
H		G4-I93D	G4-I93E	G4-I93F	G4-I93H	G4-I93K	G4-I93L	G4-I93M	G4-I93N	G4-I93P	G4-I93Q	G4-I93R

Table S26: Ranked LCMS area (SIM) of the reaction of PhOEtP(O)H with EDA across variants (P7).

	1	2	3	4	5	6	7	8	9	10	11	12
A		G5OEt	G6.1	G4-I93S	G4-I93T	G4-I93V	G4-I93Y	G4-L86A	G4-L86C	G4-L86E	G4-L86F	G4-L86G
B	G4-L86H	G4-L86I	G4-L86K	G4-L86M	G4-L86N	G4-L86P	G4-L86Q	G4-L86R	G4-L86S	G4-L86T	G4-L86V	G4-L86W
C	G4-L86Y	G11.1-G11.1	G11.1-C181A	G11.1-C181D	G11.1-C181E	G11.1-C181F	G11.1-C181G	G11.1-C181H	G4	G11.1-C181K	G11.1-C181L	G11.1-C181M
D	G11.1-C181N	G11.1-C181P	G11.1-C181R	G11.1-C181S	G11.1-C181T	G11.1-C181V	G11.1-C181W	G11.1-C181Y	G11.1-F149C	G11.1-F149D	G11.1-F149E	G11.1-F149G
E	G11.1-F149I	G11.1-F149K	G11.1-F149L	G11.1-F149M	G11.1-F149N	G11.1-F149P	G11.1-F149Q	G11.1-F149R	G11.1-F149S	G11.1-F149V	G11.1-F149W	G11.1-F149Y
F	G11.1-M59A	G11.1-M59C	G11.1-M59D	G11.1-M59E	G11.1-M59G	G11.1-M59H	G11.1-M59I	G11.1-M59K	G11.1-M59L	G11.1-M59N	G11.1-M59P	G11.1-M59Q
G	G11.1-M59R	G11.1-M59S	G11.1-M59T	G11.1-M59V	G11.1-M59W	G11.1-M59Y	G11.1-M86A	G11.1-M86C	G11.1-M86D		G11.1-M86F	G11.1-M86G
H		G11.1-M86H	G11.1-M86I	G11.1-M86K	G11.1-M86L	G11.1-M86Q	G11.1-M86R	G11.1-M86S	G11.1-M86T	G11.1-M86V	G11.1-M86W	G11.1-M86E

Table S27: Ranked LCMS area (SIM) of the reaction of PhOEtP(O)H with LAD across variants (P8).

	1	2	3	4	5	6	7	8	9	10	11	12
A		G0	G1	G2	G3	G4	G4OEt	G5OEt	G6.1	G7.1	G8.1	G9.1
B	G10.1	G11.1	G6.1	G6.1-E145A	G6.1-E145C	G6.1-E145D	G6.1-E145G	G6.1-E145I	G6.1-E145M	G6.1-E145N	G6.1-E145P	G6.1-E145Q
C	G6.1-E145T	G6.1-E145V	G6.1-L149I	G6.1-L149M	G6.1-L149V	G6.1-L86A	G6.1-L86C	G6.1-L86D	G6.1-L86E	G6.1-L86F	G6.1-L86G	G6.1-L86H
D	G6.1-L86I	G6.1-L86K	G6.1-L86M	G6.1-L86Q	G6.1-L86R	G6.1-L86S	G6.1-L86T	G6.1-L86V	G6.1-L86W	G6.1-L86Y	G6.1-M59A	G6.1-M59C
E	G6.1-M59F	G6.1-M59L	G6.1-M59Q	G6.1-M59T	G6.1-M59W	G6.1-Q181A	G6.1-Q181C	G6.1-Q181H	G6.1-Q181M	G6.1-Q181R	G6.1-Q181S	G6.1-Q181T
F	G11.1-G11.1	G11.1-C181A	G11.1-C181I	G11.1-C181M	G11.1-C181N	G11.1-C181R	G11.1-C181S	G11.1-C181T	G11.1-C181V	G11.1-C181W	G11.1-F149I	G11.1-F149L
G	G11.1-F149M	G11.1-F149V	G11.1-M59A	G11.1-M59C	G11.1-M59L	G11.1-M59N	G11.1-M59Q	G11.1-M59S	G11.1-M59T	G11.1-M86A	G11.1-M86C	G11.1-M86F
H		G11.1-M86H	G11.1-M86I	G11.1-M86K	G11.1-M86L	G11.1-M86Q	G11.1-M86R	G11.1-M86S	G11.1-M86T	G11.1-M86V	G11.1-M86W	G11.1-M86E

Table S28: Ranked LCMS area (SIM) of the reaction of PhOEtP(O)H with LAD across variants (P9).

	1	2	3	4	5	6	7	8	9	10	11	12
A		G4	G5OEt	G6.1	G11.1	G4-G4	G4-A59D	G4-A59E	G4-A59F	G4-A59G	G4-A59K	G4-A59L
B	G4-A59N	G4-A59P	G4-A59Q	G4-A59S	G4-A59T	G4-A59V	G4-A59W	G4-G145A	G4-G145C	G4-G145S	G4-G145T	G4-G145V
C	G4-I149A	G4-I149L	G4-I149M	G4-I149Q	G4-I149V	G4-I149W	G4-I93C	G4-I93F	G4-I93L	G4-I93M	G4-I93N	G4-I93S
D	G4-I93T	G4-I93V	G4-L86A	G4-L86E	G4-L86G	G4-L86H	G4-L86K	G4-L86N	G4-L86S	G4-L86T	G4-L86V	G4-L86W

Table S29: Ranked LCMS area (SIM) of the reaction of PhOEtP(O)H with LAD across variants (P10).

	1	2	3	4	5	6	7	8	9	10	11	12
A		G4	G5OEt	G6.1	G11.1	G5OEt-A59C	G5OEt-A59D	G5OEt-A59E	G5OEt-A59F	G5OEt-A59G	G5OEt-A59I	G5OEt-A59K
B	G5OEt-A59L	G5OEt-A59M	G5OEt-A59N	G5OEt-A59P	G5OEt-A59Q	G5OEt-A59S	G5OEt-A59T	G5OEt-A59V	G5OEt-A59W	G5OEt-A59Y	G5OEt-G145A	G5OEt-G145C
C	G5OEt-G145D	G5OEt-G145E	G5OEt-G145F	G5OEt-G145I	G5OEt-G145K	G5OEt-G145L	G5OEt-G145M	G5OEt-G145N	G5OEt-G145P	G5OEt-G145Q	G5OEt-G145S	G5OEt-G145T
D	G5OEt-G145V	G5OEt-G145W	G5OEt-L149C	G5OEt-L149D	G5OEt-L149E	G5OEt-L149F	G5OEt-L149H	G5OEt-L149I	G5OEt-L149K	G5OEt-L149M	G5OEt-L149P	G5OEt-L149Q
E	G5OEt-L149S	G5OEt-L149T	G5OEt-L149V	G5OEt-L149W	G5OEt-L149Y	G5OEt-Q181A	G5OEt-Q181C	G5OEt-Q181D	G5OEt-Q181E	G5OEt-Q181F	G5OEt-Q181G	G5OEt-Q181H
F	G5OEt-Q181I	G5OEt-Q181K	G5OEt-Q181L	G5OEt-Q181M	G5OEt-Q181N	G5OEt-Q181P	G5OEt-Q181R	G5OEt-Q181S	G5OEt-Q181T	G5OEt-Q181V	G5OEt-Q181W	G5OEt-Q181Y
G	G5OEt-C93D	G5OEt-C93G	G5OEt-C93I	G5OEt-C93L	G5OEt-C93N	G5OEt-C93S	G5OEt-C93V	G5OEt-L86A	G5OEt-L86C	G5OEt-L86E	G5OEt-L86F	G5OEt-L86G
H		G5OEt-L86H	G5OEt-L86I	G5OEt-L86K	G5OEt-L86M	G5OEt-L86N	G5OEt-L86P	G5OEt-L86Q	G5OEt-L86S	G5OEt-L86V	G5OEt-L86W	G5OEt-L86Y

Table S30: Ranked LCMS area (SIM) of the reaction of PhOEtP(O)H with DAN across variants (P8).

	1	2	3	4	5	6	7	8	9	10	11	12
A		G0	G1	G2	G3	G4	G4OEt	G5OEt	G6.1	G7.1	G8.1	G9.1
B	G10.1	G11.1	G6.1	G6.1-E145A	G6.1-E145C	G6.1-E145D	G6.1-E145G	G6.1-E145I	G6.1-E145M	G6.1-E145N	G6.1-E145P	G6.1-E145Q
C	G6.1-E145T	G6.1-E145V	G6.1-L149I	G6.1-L149M	G6.1-L149V	G6.1-L86A	G6.1-L86C	G6.1-L86D	G6.1-L86E	G6.1-L86F	G6.1-L86G	G6.1-L86H
D	G6.1-L86I	G6.1-L86K	G6.1-L86M	G6.1-L86Q	G6.1-L86R	G6.1-L86S	G6.1-L86T	G6.1-L86V	G6.1-L86W	G6.1-L86Y	G6.1-M59A	G6.1-M59C
E	G6.1-M59F	G6.1-M59L	G6.1-M59Q	G6.1-M59T	G6.1-M59W	G6.1-Q181A	G6.1-Q181C	G6.1-Q181H	G6.1-Q181M	G6.1-Q181R	G6.1-Q181S	G6.1-Q181T
F	G11.1-G11.1	G11.1-C181A	G11.1-C181I	G11.1-C181M	G11.1-C181N	G11.1-C181R	G11.1-C181S	G11.1-C181T	G11.1-C181V	G11.1-C181W	G11.1-F149I	G11.1-F149L
G	G11.1-F149M	G11.1-F149V	G11.1-M59A	G11.1-M59C	G11.1-M59L	G11.1-M59N	G11.1-M59Q	G11.1-M59S	G11.1-M59T	G11.1-M86A	G11.1-M86C	G11.1-M86F
H		G11.1-M86H	G11.1-M86I	G11.1-M86K	G11.1-M86L	G11.1-M86Q	G11.1-M86R	G11.1-M86S	G11.1-M86T	G11.1-M86V	G11.1-M86W	G11.1-M86E

Table S31: Ranked LCMS area (SIM) of the reaction of PhOEtP(O)H with DAN across variants (P9).

	1	2	3	4	5	6	7	8	9	10	11	12
A		G4	G5OEt	G6.1	G11.1	G4-G4	G4-A59D	G4-A59E	G4-A59F	G4-A59G	G4-A59K	G4-A59L
B	G4-A59N	G4-A59P	G4-A59Q	G4-A59S	G4-A59T	G4-A59V	G4-A59W	G4-G145A	G4-G145C	G4-G145S	G4-G145T	G4-G145V
C	G4-I149A	G4-I149L	G4-I149M	G4-I149Q	G4-I149V	G4-I149W	G4-I93C	G4-I93F	G4-I93L	G4-I93M	G4-I93N	G4-I93S
D	G4-I93T	G4-I93V	G4-L86A	G4-L86E	G4-L86G	G4-L86H	G4-L86K	G4-L86N	G4-L86S	G4-L86T	G4-L86V	G4-L86W

Table S32: Ranked LCMS area (SIM) of the reaction of PhOEtP(O)H with DAN across variants (P10).

	1	2	3	4	5	6	7	8	9	10	11	12
A		G4	G5OEt	G6.1	G11.1	G5OEt-A59C	G5OEt-A59D	G5OEt-A59E	G5OEt-A59F	G5OEt-A59G	G5OEt-A59I	G5OEt-A59K
B	G5OEt-A59L	G5OEt-A59M	G5OEt-A59N	G5OEt-A59P	G5OEt-A59Q	G5OEt-A59S	G5OEt-A59T	G5OEt-A59V	G5OEt-A59W	G5OEt-A59Y	G5OEt-G145A	G5OEt-G145C
C	G5OEt-G145D	G5OEt-G145E	G5OEt-G145F	G5OEt-G145I	G5OEt-G145K	G5OEt-G145L	G5OEt-G145M	G5OEt-G145N	G5OEt-G145P	G5OEt-G145Q	G5OEt-G145S	G5OEt-G145T
D	G5OEt-G145V	G5OEt-G145W	G5OEt-L149C	G5OEt-L149D	G5OEt-L149E	G5OEt-L149F	G5OEt-L149H	G5OEt-L149I	G5OEt-L149K	G5OEt-L149M	G5OEt-L149P	G5OEt-L149Q
E	G5OEt-L149S	G5OEt-L149T	G5OEt-L149V	G5OEt-L149W	G5OEt-L149Y	G5OEt-Q181A	G5OEt-Q181C	G5OEt-Q181D	G5OEt-Q181E	G5OEt-Q181F	G5OEt-Q181G	G5OEt-Q181H
F	G5OEt-Q181I	G5OEt-Q181K	G5OEt-Q181L	G5OEt-Q181M	G5OEt-Q181N	G5OEt-Q181P	G5OEt-Q181R	G5OEt-Q181S	G5OEt-Q181T	G5OEt-Q181V	G5OEt-Q181W	G5OEt-Q181Y
G	G5OEt-C93D	G5OEt-C93G	G5OEt-C93I	G5OEt-C93L	G5OEt-C93N	G5OEt-C93S	G5OEt-C93V	G5OEt-L86A	G5OEt-L86C	G5OEt-L86E	G5OEt-L86F	G5OEt-L86G
H		G5OEt-L86H	G5OEt-L86I	G5OEt-L86K	G5OEt-L86M	G5OEt-L86N	G5OEt-L86P	G5OEt-L86Q	G5OEt-L86S	G5OEt-L86V	G5OEt-L86W	G5OEt-L86Y

Table S33: Ranked LCMS area (SIM) of the reaction of PhCH2CH2(OEt)P(O)H with EDA across variants (P8).

	1	2	3	4	5	6	7	8	9	10	11	12
A		G0	G1	G2	G3	G4	G4OEt	G5OEt	G6.1	G7.1	G8.1	G9.1
B	G10.1	G11.1	G6.1	G6.1-E145A	G6.1-E145C	G6.1-E145D	G6.1-E145G	G6.1-E145I	G6.1-E145M	G6.1-E145N	G6.1-E145P	G6.1-E145Q
C	G6.1-E145T	G6.1-E145V	G6.1-L149I	G6.1-L149M	G6.1-L149V	G6.1-L86A	G6.1-L86C	G6.1-L86D	G6.1-L86E	G6.1-L86F	G6.1-L86G	G6.1-L86H
D	G6.1-L86I	G6.1-L86K	G6.1-L86M	G6.1-L86Q	G6.1-L86R	G6.1-L86S	G6.1-L86T	G6.1-L86V	G6.1-L86W	G6.1-L86Y	G6.1-M59A	G6.1-M59C
E	G6.1-M59F	G6.1-M59L	G6.1-M59Q	G6.1-M59T	G6.1-M59W	G6.1-Q181A	G6.1-Q181C	G6.1-Q181H	G6.1-Q181M	G6.1-Q181R	G6.1-Q181S	G6.1-Q181T
F	G11.1-G11.1	G11.1-C181A	G11.1-C181I	G11.1-C181M	G11.1-C181N	G11.1-C181R	G11.1-C181S	G11.1-C181T	G11.1-C181V	G11.1-C181W	G11.1-F149I	G11.1-F149L
G	G11.1-F149M	G11.1-F149V	G11.1-M59A	G11.1-M59C	G11.1-M59L	G11.1-M59N	G11.1-M59Q	G11.1-M59S	G11.1-M59T	G11.1-M86A	G11.1-M86C	G11.1-M86F
H		G11.1-M86H	G11.1-M86I	G11.1-M86K	G11.1-M86L	G11.1-M86Q	G11.1-M86R	G11.1-M86S	G11.1-M86T	G11.1-M86V	G11.1-M86W	G11.1-M86E

Table S34: Ranked LCMS area (SIM) of the reaction of PhCH2CH2(OEt)P(O)H with EDA across variants (P10).

	1	2	3	4	5	6	7	8	9	10	11	12
A		G4	G5OEt	G6.1	G11.1	G5OEt-A59C	G5OEt-A59D	G5OEt-A59E	G5OEt-A59F	G5OEt-A59G	G5OEt-A59I	G5OEt-A59K
B	G5OEt-A59L	G5OEt-A59M	G5OEt-A59N	G5OEt-A59P	G5OEt-A59Q	G5OEt-A59S	G5OEt-A59T	G5OEt-A59V	G5OEt-A59W	G5OEt-A59Y	G5OEt-G145A	G5OEt-G145C
C	G5OEt-G145D	G5OEt-G145E	G5OEt-G145F	G5OEt-G145I	G5OEt-G145K	G5OEt-G145L	G5OEt-G145M	G5OEt-G145N	G5OEt-G145P	G5OEt-G145Q	G5OEt-G145S	G5OEt-G145T
D	G5OEt-G145V	G5OEt-G145W	G5OEt-L149C	G5OEt-L149D	G5OEt-L149E	G5OEt-L149F	G5OEt-L149H	G5OEt-L149I	G5OEt-L149K	G5OEt-L149M	G5OEt-L149P	G5OEt-L149Q
E	G5OEt-L149S	G5OEt-L149T	G5OEt-L149V	G5OEt-L149W	G5OEt-L149Y	G5OEt-Q181A	G5OEt-Q181C	G5OEt-Q181D	G5OEt-Q181E	G5OEt-Q181F	G5OEt-Q181G	G5OEt-Q181H
F	G5OEt-Q181I	G5OEt-Q181K	G5OEt-Q181L	G5OEt-Q181M	G5OEt-Q181N	G5OEt-Q181P	G5OEt-Q181R	G5OEt-Q181S	G5OEt-Q181T	G5OEt-Q181V	G5OEt-Q181W	G5OEt-Q181Y
G	G5OEt-C93D	G5OEt-C93G	G5OEt-C93I	G5OEt-C93L	G5OEt-C93N	G5OEt-C93S	G5OEt-C93V	G5OEt-L86A	G5OEt-L86C	G5OEt-L86E	G5OEt-L86F	G5OEt-L86G
H		G5OEt-L86H	G5OEt-L86I	G5OEt-L86K	G5OEt-L86M	G5OEt-L86N	G5OEt-L86P	G5OEt-L86Q	G5OEt-L86S	G5OEt-L86V	G5OEt-L86W	G5OEt-L86Y

Table S35: Master SSM compilation plate for substrate scope screening (P11).

	1	2	3	4	5	6	7	8	9	10	11	12
**A**	G3	G4	G4OEt	G5OEt	G6.1	G11.1	G4-A59G	G4-A59T	G4-L86N	G4-L86A	G4-L86G	G4-I93C
**B**	G4-I93F	G4-I93L	G4-I93M	G4-I93N	G4-I93S	G4-I93T	G4-I93V	G4-I149M	G5OEt-A59I	G5OEt-A59L	G5OEt-A59M	G5OEt-A59T
**C**	G5OEt-A59V	G5OEt-L86A	G5OEt-L86E	G5OEt-L86F	G5OEt-L86G	G5OEt-L86K	G5OEt-L86Q	G5OEt-L86Y	G5OEt-C93G	G5OEt-C93I	G5OEt-C93L	G5OEt-C93S
**D**	G6.1-M59L	G6.1-M59F	G6.1-L86M	G6.1-L86Y	G6.1-E145G	G6.1-L149V	G6.1-Q181A	G11.1-M59L	G11.1-F149L	G11.1-F149V	G11.1-C181R	BLANK
**E**	G3	G4	G4OEt	G5OEt	G6.1	G11.1	G4-A59G	G4-A59T	G4-L86N	G4-L86A	G4-L86G	G4-I93C
**F**	G4-I93F	G4-I93L	G4-I93M	G4-I93N	G4-I93S	G4-I93T	G4-I93V	G4-I149M	G5OEt-A59I	G5OEt-A59L	G5OEt-A59M	G5OEt-A59T
**G**	G5OEt-A59V	G5OEt-L86A	G5OEt-L86E	G5OEt-L86F	G5OEt-L86G	G5OEt-L86K	G5OEt-L86Q	G5OEt-L86Y	G5OEt-C93G	G5OEt-C93I	G5OEt-C93L	G5OEt-C93S
**H**	G6.1-M59L	G6.1-M59F	G6.1-L86M	G6.1-L86Y	G6.1-E145G	G6.1-L149V	G6.1-Q181A	G11.1-M59L	G11.1-F149L	G11.1-F149V	G11.1-C181R	BLANK

Table S36: Screening results (LCMS area, SIM) for the substrate scope with EDA using the master SSM compilation plate (P11)

Well ID	Variant	3a PhBnP(O)H	4a PhiPrP(O)H	5a PhCyP(O)H	6a Ph(4-FPh)P(O)H	7a Ph(oAn)P(O)H	8a Ph(2-Np)P(O)H	9a 4-FPh(OEt)P(O)H	10a 4MePh(OEt)P(O)H	11a 2-Np(OEt)P(O)H	12a PhCH2CH2(OEt)P(O)H	13a iBu(OEt)P(O)H
A1	G3	5.7E+06	4.9E+06	5.1E+06	8.2E+05	1.5E+05	5.6E+05		1.7E+05	5.7E+04	2.1E+04	5.5E+05
A2	G4	4.5E+06	1.1E+06	1.6E+06	5.5E+05	7.2E+04	6.4E+05		1.7E+05	4.5E+05	2.4E+04	2.9E+05
A3	G4OEt	4.4E+06	6.0E+06	6.9E+06	5.5E+05	1.8E+05	7.1E+05	9.0E+05	7.5E+05	2.0E+05	8.5E+04	1.3E+06
A4	G5OEt	4.2E+06	6.5E+06	7.6E+06	5.3E+05	2.6E+05	7.4E+05	1.2E+06	8.2E+05	1.7E+05	1.0E+05	1.4E+06
A5	G6.1	5.8E+06	1.1E+06	2.9E+06	7.8E+05	8.5E+04	2.0E+06	1.8E+05	7.1E+05	9.6E+05	5.3E+05	7.5E+04
A6	G11.1	1.9E+06	1.7E+06	2.9E+06	2.6E+06	2.5E+05	4.5E+06	1.1E+06	5.4E+06	4.7E+06	1.9E+06	2.8E+05
A7	G4-A59G	2.1E+06	3.4E+06	4.7E+05	3.0E+05	6.4E+04	5.3E+05		6.5E+04	1.7E+05	1.0E+04	1.2E+05
A8	G4-A59T	5.3E+06	7.0E+06	7.9E+06	1.9E+06	1.6E+05	7.6E+05	1.2E+06	3.8E+05	1.8E+05	1.1E+05	2.4E+06
A9	G4-L86N	4.3E+06	6.1E+06	1.2E+06	1.3E+06	5.8E+04	7.7E+05		7.7E+04	1.7E+05	1.1E+04	4.0E+05
A10	G4-L86A	4.3E+06	1.1E+06	1.2E+06	8.5E+05	8.1E+04	6.2E+05		2.0E+05	4.1E+05	3.1E+04	2.7E+05
A11	G4-L86G	3.0E+06	1.8E+06	8.6E+05	6.3E+05	7.6E+04	6.5E+05		2.7E+05	3.4E+05	3.5E+04	2.1E+05
A12	G4-I93C	5.7E+06	3.6E+06	4.5E+06	1.3E+06	1.5E+05	2.1E+06	4.3E+05	1.0E+06	1.5E+06	2.3E+05	3.7E+05
B1	G4-I93F	1.6E+06	4.6E+05	2.1E+06	4.1E+05	8.3E+04	1.4E+06		1.9E+05	4.2E+05	2.5E+04	6.7E+04
B2	G4-I93L	4.0E+06	1.2E+06	2.0E+06	4.5E+05	1.0E+05	9.0E+05	1.4E+05	2.8E+05	4.3E+05	2.0E+04	4.7E+05
B3	G4-I93M	1.2E+06	2.0E+05	5.8E+05	1.0E+05	5.6E+04	6.4E+05		7.1E+04	1.1E+05	6.2E+03	3.3E+04
B4	G4-I93N	2.4E+06	7.8E+05	2.1E+06	2.9E+05	1.2E+05	2.1E+06		6.9E+04	8.6E+04	9.7E+03	1.6E+05
B5	G4-I93S	2.0E+06	3.2E+06	5.0E+06	1.6E+06	5.2E+05	2.7E+06	1.3E+05	7.5E+04	6.0E+04	1.1E+04	2.3E+05
B6	G4-I93T	5.8E+06	5.0E+06	3.3E+06	1.0E+06	2.5E+05	1.5E+06	1.6E+05	1.3E+05	5.1E+04	1.6E+04	4.0E+05
B7	G4-I93V	5.0E+06	1.2E+06	1.2E+06	4.1E+05	9.6E+04	1.2E+06		1.0E+05	1.2E+05	1.5E+04	1.4E+05
B8	G4-I149M	4.7E+06	6.4E+06	9.5E+05	5.0E+05	6.6E+04	6.3E+05		8.2E+04	9.6E+04	3.9E+04	2.9E+05
B9	G5OEt-A59I	6.6E+05	6.0E+05	3.2E+06	3.3E+05	1.8E+05	6.3E+05	7.6E+05	6.0E+05	1.3E+05	2.4E+04	1.6E+06
B10	G5OEt-A59L	4.5E+05	1.2E+06	4.5E+06	7.0E+05	6.2E+05	7.9E+05	2.1E+06	9.9E+05	1.7E+05	4.1E+04	2.9E+06
B11	G5OEt-A59M	4.8E+05	2.2E+05	3.4E+06	3.7E+05	2.5E+05	6.3E+05	1.0E+06	7.6E+05	2.3E+05	6.3E+04	1.1E+06
B12	G5OEt-A59T	4.2E+05	7.7E+05	9.8E+05	2.2E+05	9.5E+04	6.0E+05	1.2E+05	1.6E+05	1.2E+05	1.9E+04	2.2E+05
C1	G5OEt-A59V	6.4E+05	3.2E+06	3.5E+06	4.9E+05	3.0E+05	6.4E+05	8.1E+05	5.1E+05	1.1E+05	2.5E+04	1.4E+06
C2	G5OEt-L86A	3.2E+06	6.5E+06	6.8E+06	7.5E+05	4.5E+05	1.2E+06	1.3E+06	8.9E+05	2.2E+05	7.0E+04	1.6E+06
C3	G5OEt-L86E	3.4E+06	5.3E+06	5.3E+06	3.7E+05	2.4E+05	8.4E+05	5.8E+05	5.9E+05	1.5E+05	7.6E+04	1.8E+06
C4	G5OEt-L86F	3.5E+06	4.9E+06	4.8E+06	4.7E+05	3.1E+05	6.6E+05	5.8E+05	4.5E+05	1.2E+05	5.2E+04	1.1E+06
C5	G5OEt-L86G	3.0E+06	5.8E+06	4.7E+06	2.5E+05	2.3E+05	5.5E+05	8.8E+05	6.8E+05	1.5E+05	5.7E+04	1.2E+06
C6	G5OEt-L86K	4.7E+06	6.5E+06	7.3E+06	3.4E+05	2.8E+05	5.3E+05	1.2E+06	6.7E+05	2.0E+05	1.1E+05	1.5E+06
C7	G5OEt-L86Q	5.1E+06	7.0E+06	8.1E+06	6.4E+05	3.6E+05	6.4E+05	1.3E+06	1.1E+06	3.1E+05	1.7E+05	1.8E+06
C8	G5OEt-L86Y	4.5E+06	6.3E+06	6.8E+06	5.6E+05	3.4E+05	5.8E+05	7.8E+05	5.5E+05	1.6E+05	9.3E+04	1.6E+06
C9	G5OEt-C93G	1.7E+06	6.5E+06	6.6E+06	1.5E+06	9.3E+05	2.7E+06	4.5E+05	5.8E+05	3.1E+05	2.1E+04	4.4E+05
C10	G5OEt-C93I	3.0E+06	2.2E+06	2.5E+06	6.2E+05	2.1E+05	1.4E+06	1.6E+05	2.3E+05	3.5E+05	2.4E+04	4.3E+05
C11	G5OEt-C93L	3.0E+06	1.9E+06	1.9E+06	3.0E+05	1.3E+05	8.5E+05	3.9E+05	2.4E+05	3.6E+05	2.7E+04	8.0E+05
C12	G5OEt-C93S	2.9E+06	6.5E+06	7.2E+06	2.8E+06	1.8E+06	2.5E+06	5.7E+05	4.3E+05	3.6E+05	2.9E+04	7.8E+05
D1	G6.1-M59L	2.4E+06	6.1E+05	1.5E+06	5.4E+05	1.3E+05	1.8E+06	1.5E+05	7.3E+05	1.5E+06	4.7E+05	4.3E+04
D2	G6.1-M59F	2.2E+06	5.4E+05	2.6E+06	1.0E+06	1.2E+05	2.4E+06		8.9E+05	1.6E+06	2.0E+05	3.0E+04
D3	G6.1-L86M	6.2E+06	8.6E+05	3.1E+06	1.3E+06	9.3E+04	4.9E+06	1.2E+05	8.2E+05	2.0E+06	5.4E+05	4.5E+04
D4	G6.1-L86Y	5.5E+06	1.5E+06	3.7E+06	1.5E+06	5.4E+05	2.8E+06	1.7E+05	7.6E+05	1.0E+06	2.9E+05	8.4E+04
D5	G6.1-E145G	1.4E+06	4.3E+06	3.1E+06	3.8E+05	1.9E+05	5.7E+05	5.6E+05	5.5E+05	2.0E+05	8.2E+04	9.3E+05
D6	G6.1-L149V	5.6E+06	1.5E+06	4.4E+06	8.1E+05	6.2E+04	1.7E+06	1.2E+05	4.6E+05	4.4E+05	8.5E+04	7.6E+04
D7	G6.1-Q181A	1.0E+06	2.6E+05	1.1E+06	4.2E+05	9.7E+04	1.5E+06		3.9E+05	6.3E+05	7.9E+04	2.5E+04
D8	G11.1-M59L	1.9E+06	4.2E+05	4.0E+06	3.6E+06	6.3E+05	4.4E+06	1.0E+06	5.9E+06	5.0E+06	1.5E+06	2.3E+05
D9	G11.1-F149L	4.5E+06	8.8E+05	5.9E+06	4.3E+06	5.9E+05	4.6E+06	7.5E+05	5.1E+06	4.3E+06	1.6E+06	3.2E+05
D10	G11.1-F149V	5.8E+06	1.4E+06	7.2E+06	4.9E+06	1.6E+06	6.4E+06	8.5E+05	5.2E+06	3.5E+06	1.2E+06	4.9E+05
D11	G11.1-C181R	1.1E+06	1.4E+06	5.6E+06	3.1E+06	4.7E+05	4.2E+06	8.8E+05	5.7E+06	5.1E+06	1.6E+06	3.9E+05
D12	BLANK	2.6E+05	1.9E+05	3.9E+05	9.9E+04	4.6E+04	7.8E+05		1.0E+05	1.9E+05	1.7E+04	1.5E+04

5.4 Nitrene P–H Insertion: Ph(OEt)P(O)H (2a)

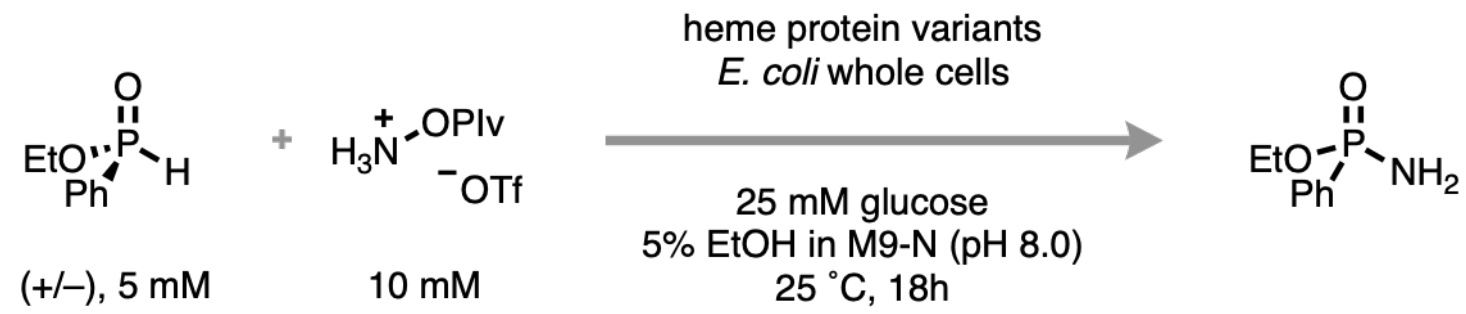

Reactions were performed according to the validation procedure (three biological replicates, with three technical replicates for assay yield, and one technical replicate for enantioselectivity).

Reverse Phase HPLC-MS: Agilent Poroshell 120 EC-C18, 1.5 mL/min, [A] 0.1% AcOH in Water, [B] 0.1% AcOH in Acetonitrile, scan and single ion monitoring $[M+H]^+$, gradient: 0-0.2 min: 5-50% B; 0.2-0.4 min: 5–20% B; 0.4–2.5 min: 20–50% B; 2.5–2.6 min: 50–95% B; 2.6–3.0 min: 95% B; 3.0–3.1 min: 95–5% B; 3.1–3.5 min: 5% B.

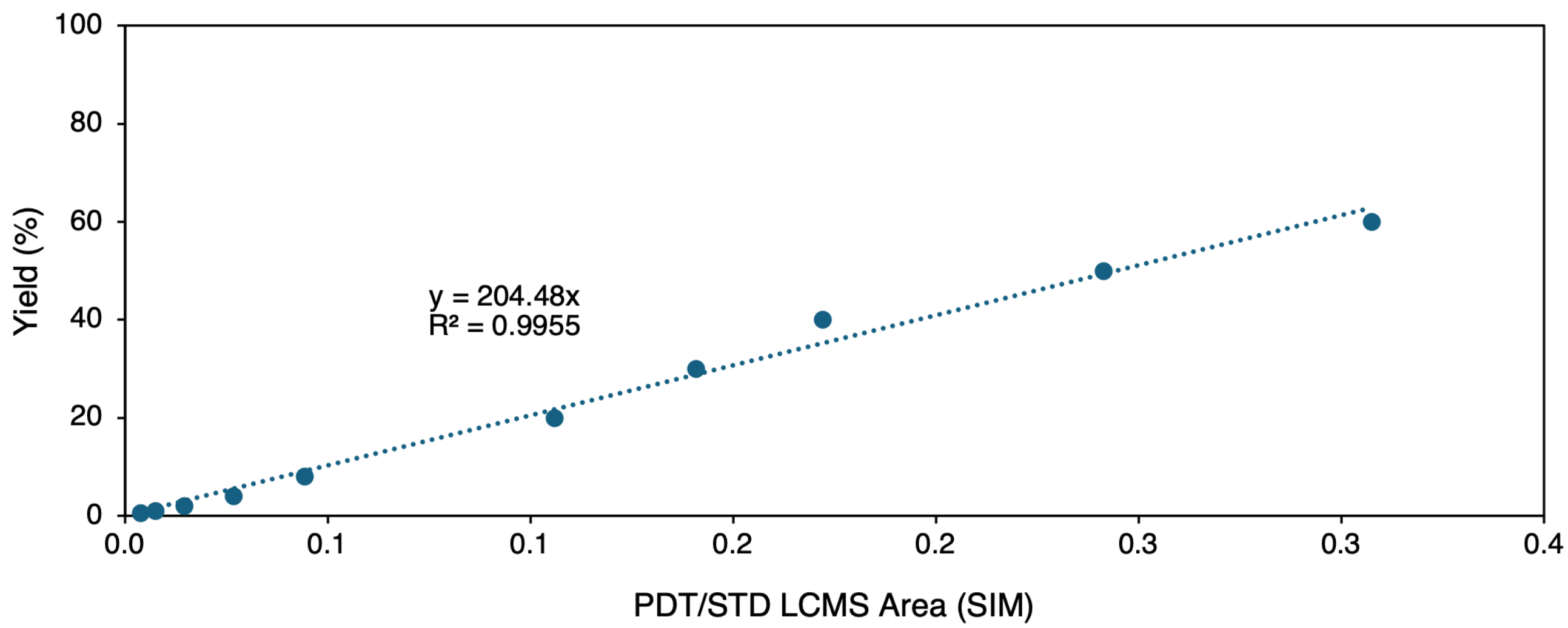

Figure S10: Calibration curve using authentic sample and papaverine as an external standard.

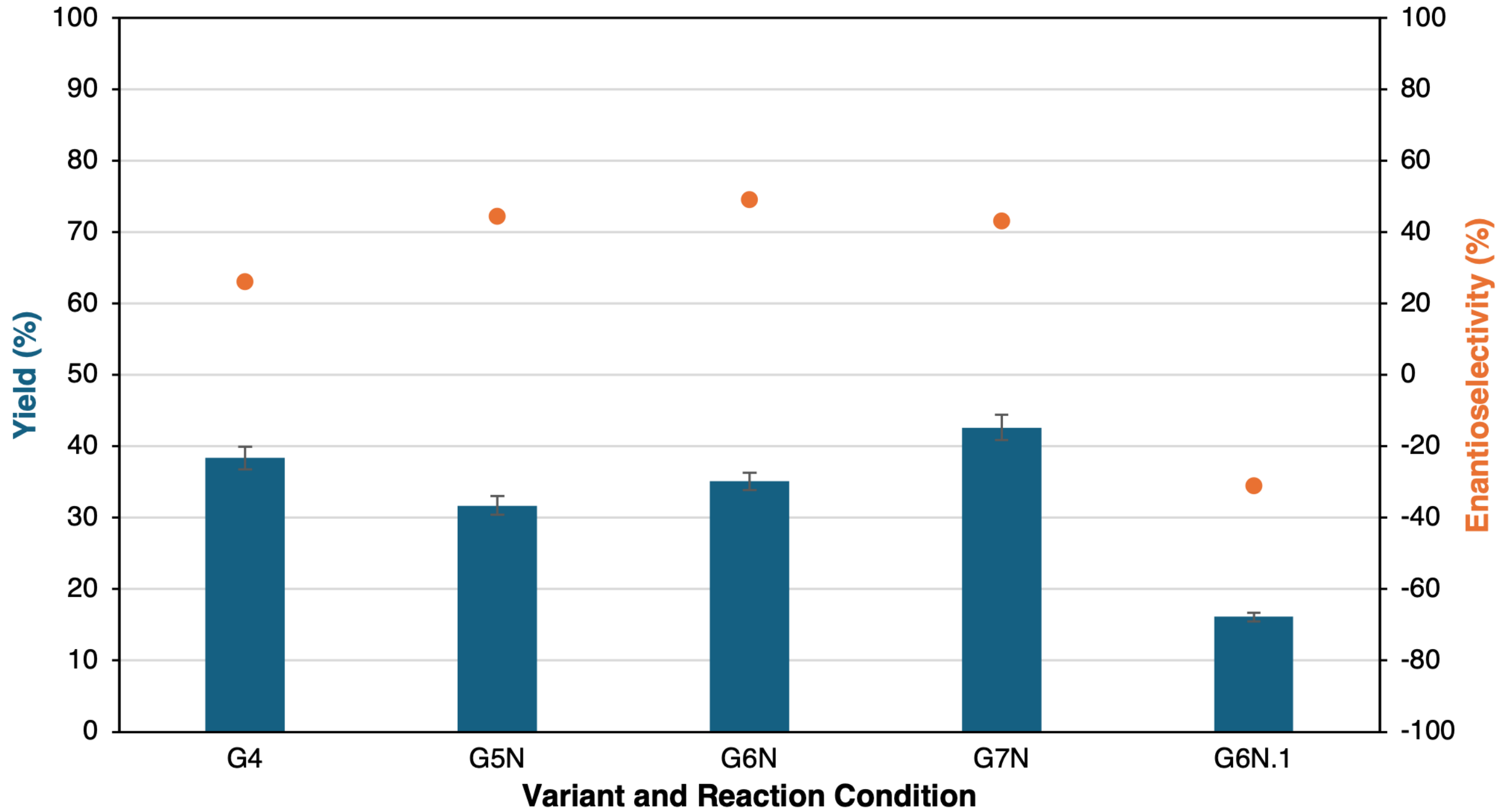

Figure S11: Visualization of the yield and enantioselectivity of variants and selected reaction conditions for the reaction of Ph(OEt)P(O)H and PONT.

Table S36: Yield and enantioselectivity of variants and selected reaction conditions for the reactions of Ph(OEt)P(O)H and PONT.

Variant/conditions	Avg. Yield (%)	Std Dev Yield (%)	Avg. E.E. (%)
G4	38	2	26
G5N	32	1	45
G6N	35	1	49
G7N	43	2	43
G6N.1	16	1	-31

6. DNA Sequences of Evolved Variants

6.1 Carbene P–H lineage

G0-5462

ATGACTCCCTCGGACATCCCGGGGTATGATTATGGGCGTGTCGAGAAGTCACCCATCACGGACCTTG
AGTTTGACCTTCTGAAGAAGACTGTCATGTTAGGTGAAGAGGACGTAATGTACTTGAAAAAGGCGG
CTGACGTTCTGAAAGATCAAGTTGATGAGATCCTTGACCTGGCGGGTGGTTGGGTAGCATCAAATGA
GCATTTGATTTATTACTTCTCCAATCCGGATACAGGAGAGCCTATTAAAGAATACCTGGAACGTGTA
CGCGCTCGCGGTGGAGCCTGGGTTCTGGACACTACCTGCCGCGACTATAACCGTGAATGGTTAGACT
ACCAGTACGAAGTTGGGCTTCGTCATCACCGTTCAAAGAAAGGGGTCACAGACGGAGTACGCACCG
TGCCCAATACCCCACTTCGTTATCTTATCGCAGGTATCTATCCTATCACCGCCACTATCAAGCCACTT
TTAGCTGAGAAAGGTGGCTCTCCGGAAGACATCGAAGGGATGTACAACGCTTGGCTCAAGTCTGTA
GTTCTACAAGTTGCCATCTGGTCACACCCTTATACTAAGGAGAATGACTGGCTCGAGCACCACCACC
ACCACCACTGA

MTPSDIPGYDYGRVEKSPITDLEFDLLKKTVMLGEEDVMYLKKAADVLKDQVDEILDLAGGWVASNEH
LIYYFSNPDTGEPIKEYLERVRARGGAWVLDTTCRDYNREWLDYQYEVGLRHHRSKKGVTDGVRTVPN
TPLRYLIAGIYPITATIKPLLAEKGGSPEDIEGMYNAWLKSVVLQVAIWSHPYTKENDWLEHHHHHH*

G1-5463

ATGACTCCCTCGGACATCCCGGGGTATGATTATGGGCGTGTCGAGAAGTCACCCATCACGGACCTTG
AGTTTGACCTTCTGAAGAAGACTGTCATGTTAGGTGAAGAGGACGTAATGTACTTGAAAAAGGCGG
CTGACGTTCTGAAAGATCAAGTTGATGAGATCCTTGACCTGGCGGGTGGTTGGGCAGCATCAAATG
AGCATTTGATTTATTACTTCTCCAATCCGGATACAGGAGAGCCTATTAAAGAATACCTGGAACGTGT
ACGCGCTCGCGGTGGAGCCTGGGTTCTGGACACTACCTGCCGCGACTATAACCGTGAATGGTTAGAC
TACCAGTACGAAGTTGGGCTTCGTCATCACCGTTCAAAGAAAGGGGTCACAGACGGAGTACGCACC
GTGCCCAATACCCCACTTCGTTATCTTATCGCAGGTATCTATCCTATCACCGCCACTATCAAGCCACT
TTTAGCTGAGAAAGGTGGCTCTCCGGAAGACATCGAAGGGATGTACAACGCTTGGCTCAAGTCTGT
AGTTCTACAAGTTGCCATCTGGTCACACCCTTATACTAAGGAGAATGACTGGCTCGAGCACCACCAC
CACCACCACTGA

MTPSDIPGYDYGRVEKSPITDLEFDLLKKTVMLGEEDVMYLKKAADVLKDQVDEILDLAGGWAASNEH
LIYYFSNPDTGEPIKEYLERVRARGGAWVLDTTCRDYNREWLDYQYEVGLRHHRSKKGVTDGVRTVPN
TPLRYLIAGIYPITATIKPLLAEKGGSPEDIEGMYNAWLKSVVLQVAIWSHPYTKENDWLEHHHHHH*

G2-5464

ATGACTCCCTCGGACATCCCGGGGTATGATTATGGGCGTGTCGAGAAGTCACCCATCACGGACCTTG
AGTTTGACCTTCTGAAGAAGACTGTCATGTTAGGTGAAGAGGACGTAATGTACTTGAAAAAGGCGG
CTGACGTTCTGAAAGATCAAGTTGATGAGATCCTTGACCTGGCGGGTGGTTGGGCAGCATCAAATG
AGCATTTGATTTATTACTTCTCCAATCCGGATACAGGAGAGCCTATTAAAGAATACCTGGAACGTGT
ACGCGCTCGCTGTGGAGCCTGGGTTCTGGACACTACCTGCCGCGACTATAACCGTGAATGGTTAGAC
TACCAGTACGAAGTTGGGCTTCGTCATCACCGTTCAAAGAAAGGGGTCACAGACGGAGTACGCACC
GTGCCCAATACCCCACTTCGTTATCTTATCGCAGGTATCTATCCTATCACCGCCACTATCAAGCCACT
TTTAGCTGAGAAAGGTGGCTCTCCGGAAGACATCGAAGGGATGTACAACGCTTGGCTCAAGTCTGT
AGTTCTACAAGTTGCCATCTGGTCACACCCTTATACTAAGGAGAATGACTGGCTCGAGCACCACCAC
CACCACCACTGA

MTPSDIPGYDYGRVEKSPITDLEFDLLKKTVMLGEEDVMYLKKAADVLKDQVDEILDLAGGWAASNEH
LIYYFSNPDTGEPIKEYLERVRARCGAWVLDTTCRDYNREWLDYQYEVGLRHHRSKKGVTDGVRTVPN
TPLRYLIAGIYPITATIKPLLAEKGGSPEDIEGMYNAWLKSVVLQVAIWSHPYTKENDWLEHHHHHH*

G3-5465

ATGACTCCCTCGGACATCTCGGGGTATGATTATGGGCGTGTCGAGAAGTCACCCATCACGGACCTTG
AGTTTGACCTTCTGAAGAAGACTGTCATGTTAGGTGAAGAGGACGTAATGTACTTGAAAAAGGCGG
CTGACGTTCTGAAAGATCAAGTTGATGAGATCCTTGACCTGGCGGGTGGTTGGGCAGCATCAAATG
AGCATTTGATTTATTACTTCTCCAATCCGGATACAGGAGCGCCTATTAAAGAATACCTGGAACGTGT
ACGCGCTCGCTGTGGAGCCTGGGTTCTGGACACTACCTGCCGCGACTATAACCGTGAATGGTTAGAC
TACCAGTACGAAGTTGGGCTTCGTCATCACCGTTCAAAGAAAGGGGTCACAGACGGAGTACGCACC
GTGCCCAATACCCCACTTCGTTATCTTATCGCAGGTATCTATCCTATCACCGCCACTATCAAGCCATT
TTTAGCTAAGAAAGGTGGCTCTCCGGAGGACATCGAAGGGATGTACAACGCTTGGCTCAAGTCTGT
AGTTCTACAAGTTGCCATCTGGTCACACCCTTATACTAAGGAGAATGACCGGCTCGAGCACCACCAC
CACCACCACTGA

MTPSDISGYDYGRVEKSPITDLEFDLLKKTVMLGEEDVMYLKKAADVLKDQVDEILDLAGGWAASNEH
LIYYFSNPDTGAPIKEYLERVRARCGAWVLDTTCRDYNREWLDYQYEVGLRHHRSKKGVTDGVRTVPN
TPLRYLIAGIYPITATIKPFLAKKGGSPEDIEGMYNAWLKSVVLQVAIWSHPYTKENDRLEHHHHHH*

G4-5466

ATGACTCCCTCGGACATCTCGGGGTATGATTATGGGCGTGTCGAGAAGTCACCCATCACGGACCTTG
AGTTTGACCTTCTGAAGAAGACTGTCATGTTAGGTGAAGAGGACGTAATGTACTTGAAAAAGGCGG
CTGACGTTCTGAAAGATCAAGTTGATGAGATCCTTGACCTGGCGGGTGGTTGGGCAGCATCAAATG
AGCATTTGATTTATTACTTCTCCAATCCGGATACAGGAGCGCCTATTAAAGAATACCTGGAACGTGT
ACGCGCTCGCATTGGAGCCTGGGTTCTGGACACTACCTGCCGCGACTATAACCGTGAATGGTTAGAC
TACCAGTACGAAGTTGGGCTTCGTCATCACCGTTCAAAGAAAGGGGTCACAGACGGAGTACGCACC
GTGCCCAATACCCCACTTCGTTATCTTATCGCAGGTATCTATCCTATCACCGCCACTATCAAGCCATT
TTTAGCTAAGAAAGGTGGCTCTCCGGAGGACATCGAAGGGATGTACAACGCTTGGCTCAAGTCTGT
AGTTCTACAAGTTGCCATCTGGTCACACCCTTATACTAAGGAGAATGACCGGCTCGAGCACCACCAC
CACCACCACTGA

MTPSDISGYDYGRVEKSPITDLEFDLLKKTVMLGEEDVMYLKKAADVLKDQVDEILDLAGGWAASNEH
LIYYFSNPDTGAPIKEYLERVRARIGAWVLDTTCRDYNREWLDYQYEVGLRHHRSKKGVTDGVRTVPN
TPLRYLIAGIYPITATIKPFLAKKGGSPEDIEGMYNAWLKSVVLQVAIWSHPYTKENDRLEHHHHHH*

G4OEt-5467

ATGACTCCCTCGGACATCTCGGGGTATGATTATGGGCGTGTCGAGAAGTCACCCATCACGGACCTTG
AGTTTGACCTTCTGAAGAAGACTGTCATGTTAGGTGAAGAGGACGTAATGTACTTGAAAAAGGCGG
CTGACGTTCTGAAAGATCAAGTTGATGAGATCCTTGACCTGGCGGGTGGTTGGGCAGCATCAAATG
AGCATTTGATTTATTACTTCTCCAATCCGGATACAGGAGCGCCTATTAAAGAATACCTGGAACGTGT
ACGCGCTCGCTGTGTAGCCTGGGTTCTGGACACTACCTGCCGCGACTATAACCGTGAATGGTTAGAC
TACCAGTACGAAGTTGGGCTTCGTCATCACCGTTCAAAGAAAGGGGTCACAGACGGAGTACGCACC
GTGCCCAATACCCCACTTCGTTATCTTATCGCAGGTATCTATCCTCTGACCGCCACTATCAAGCCATT
TTTAGCTAAGAAAGGTGGCTCTCCGGAGGACATCGAAGGGATGTACAACGCTTGGCTCAAGTCTGT
AGTTCTACAAGTTGCCATCTGGTCACACCCTTATACTAAGGAGAATGACCGGCTCGAGCACCACCAC
CACCACCACTGA

MTPSDISGYDYGRVEKSPITDLEFDLLKKTVMLGEEDVMYLKKAADVLKDQVDEILDLAGGWAASNEH
LIYYFSNPDTGAPIKEYLERVRARCVAWVLDTTCRDYNREWLDYQYEVGLRHHRSKKGVTDGVRTVPN
TPLRYLIAGIYPLTATIKPFLAKKGGSPEDIEGMYNAWLKSVVLQVAIWSHPYTKENDRLEHHHHHH*

G5OEt-5468

ATGACTCCCTCGGACATCTCGGGGTATGATTATGGGCGTGTCGAGAAGTCACCCGTCACGGACCTCG
AGTTTGACCTCCTGAAGAAGACTGTCATGTTTGGTGAAGAGGACGTAATGTACTTGAAAAAGGCGG
CTGACGTTCTGAAAGATCAAGTTGATGAGATCCTTGACCTGGCGGGTGGTTGGGCAGCATCAAATG
AGCATTTGGTTTATTACTTCTCCAATCCGGATACAGGAGCGCCTATTAAAGAATACCTGGAACGTGT
ACGCGCTCGCTGTGTAGCCTGGGTTCTGGACACCACCTGCCGCGACTATAACCGTGAATGGTTAGAC
TACCAGTACGAAGTTGGGCTTCGTCATCACCGTTCAAAGAAAGGGGTCACAGACGGAGTACGCACC
GTGCCCAATACCCCACTTCGATATCTTATCGCAGGTATCTATCCTCTGACCGCCACTATCAAGCCATT
TTTAGCTAAGAAAGGTGGCTCTCCGGAGGACATCGAAGGGATGTACAACGCTTGGCTCAAGTCTGT
AGTTCTACAAGTTGCCATCTGGTCACACCCTTATACTAAGGAGAATGACCGGCTCGAGCACCACCAC
CACCACCACTGA

MTPSDISGYDYGRVEKSPVTDLEFDLLKKTVMFGEEDVMYLKKAADVLKDQVDEILDLAGGWAASNE
HLVYYFSNPDTGAPIKEYLERVRARCVAWVLDTTCRDYNREWLDYQYEVGLRHHRSKKGVTDGVRTV
PNTPLRYLIAGIYPLTATIKPFLAKKGGSPEDIEGMYNAWLKSVVLQVAIWSHPYTKENDRLEHHHHHH*

G5.1-5469

ATGACTCCCTCGGACATCTCGGGGTATGATTATGGGCGTGTCGAGAAGTCACCCATCACGGACCTTG
AGTTTGACCTTCTGAAGAAGACTGTCATGTTAGGTGAAGAGGACGTAATGTACTTGAAAAAGGCGG
CTGACGTTCTGAAAGATCAAGTTGATGAGATCCTTGACCTGATGGGTGGTTGGGCAGCATCAAATGA
GCATTTGATTTATTACTTCTCCAATCCGGATACAGGAGCGCCTATTAAAGAATACCTGGAACGTGTA
CGCGCTCGCTGTGTAGCCTGGGTTCTGGACACTACCTGCCGCGACTATAACCGTGAATGGTTAGACT
ACCAGTACGAAGTTGGGCTTCGTCATCACCGTTCAAAGAAAGGGGTCACAGACGGAGTACGCACCG
TGCCCAATACCCCACTTCGTTATCTTATCGCAGGTATCTATCCTCTGACCGCCACTATCAAGCCATTT
TTAGCTAAGAAAGGTGGCTCTCCGGAGGACATCGAAGGGATGTACAACGCTTGGCTCAAGTCTGTA
GTTCTACAAGTTGCCATCTGGTCACACCCTTATACTAAGGAGAATGACCGGCTCGAGCACCACCACC
ACCACCACTGA

MTPSDISGYDYGRVEKSPITDLEFDLLKKTVMLGEEDVMYLKKAADVLKDQVDEILDLMGGWAASNEH
LIYYFSNPDTGAPIKEYLERVRARCVAWVLDTTCRDYNREWLDYQYEVGLRHHRSKKGVTDGVRTVPN
TPLRYLIAGIYPLTATIKPFLAKKGGSPEDIEGMYNAWLKSVVLQVAIWSHPYTKENDRLEHHHHHH*

G6.1-5470

ATGACTCCCTCGGACATCTCGGGGTATGATTATGGGCGTGTCGAGAAGTCACCCATCACGGACCTTG
AGTTTGACCTTCTGAAGAAGACTGTCATGTTAGGTGAAGAGGACGTAATGTACTTGAAAAAGGCGG
CTGACGTTCTGAAAGATCAAGTTGATGAGATCCTTGACCTGATGGGTGGTTGGGCAGCATCAAATGA
GCATTTGATTTATTACTTCTCCAATCCGGATACAGGAGCGCCTATTAAAGAATACCTGGAACGTGTA
CGCGCTCGCTGTGTAGCCTGGGTTCTGGACACTACCTGCCGCGACTATAACCGTGAATGGTTAGACT
ACCAGTACGAAGTTGGGCTTCGTCATCACCGTTCAAAGAAAGGGGTCACAGACGGAGTACGCACCG
TGCCCAATACCCCACTTCGTTATCTTATCGCAGAGATCTATCCTCTGACCGCCACTATCAAGCCATTT
TTAGCTAAGAAAGGTGGCTCTCCGGAGGACATCGAAGGGATGTACAACGCTTGGCTCAAGTCTGTA
GTTCTACAAGTTGCCATCTGGTCACACCCTTATACTAAGGAGAATGACCGGCTCGAGCACCACCACC
ACCACCACTGA

MTPSDISGYDYGRVEKSPITDLEFDLLKKTVMLGEEDVMYLKKAADVLKDQVDEILDLMGGWAASNEH
LIYYFSNPDTGAPIKEYLERVRARCVAWVLDTTCRDYNREWLDYQYEVGLRHHRSKKGVTDGVRTVPN
TPLRYLIAEIYPLTATIKPFLAKKGGSPEDIEGMYNAWLKSVVLQVAIWSHPYTKENDRLEHHHHHH*

G7.1-5471

ATGACTCCCTCGGACATCTCGGGGTATGATTATGGGCGTGTCGAGAAGTCACCCATCACGGACCTTG
AGTTTGACCTTCTGAAGAAGACTGTCATGTTAGGTGAAGAGGACGTAATGTACTTGAAAAAGGCGG
CTGACGTTCTGAAAGATCAAGTTGATGAGATCCTTGACCTGATGGGTGGTTGGCTGGCATCAAATGA
GCATTTGATTTATTACTTCTCCAATCCGGATACAGGAGCGCCTATTAAAGAATACCTGGAACGTGTA
CGCGCTCGCTGTGTAGCCTGGGTTCTGGACACTACCTGCCGCGACTATAACCGTGAATGGTTAGACT
ACCAGTACGAAGTTGGGCTTCGTCATCACCGTTCAAAGAAAGGGGTCACAGACGGAGTACGCACCG
TGCCCAATACCCCACTTCGTTATCTTATCGCAGAGATCTATCCTCTGACCGCCACTATCAAGCCATTT
TTAGCTAAGAAAGGTGGCTCTCCGGAGGACATCGAAGGGATGTACAACGCTTGGCTCAAGTCTGTA
GTTCTACAAGTTGCCATCTGGTCACACCCTTATACTAAGGAGAATGACCGGCTCGAGCACCACCACC
ACCACCACTGA

MTPSDISGYDYGRVEKSPITDLEFDLLKKTVMLGEEDVMYLKKAADVLKDQVDEILDLMGGWLASNEH
LIYYFSNPDTGAPIKEYLERVRARCVAWVLDTTCRDYNREWLDYQYEVGLRHHRSKKGVTDGVRTVPN
TPLRYLIAEIYPLTATIKPFLAKKGGSPEDIEGMYNAWLKSVVLQVAIWSHPYTKENDRLEHHHHHH*

G8.1-5472

ATGACTCCCTCGGACATCTCGGGGTATGATTATGGGCATGTCGAGAAGTCACCCATCACGGACCTTG
AGTTTGACCTTCTGAAGAAGACTGTCATGTTAGGTGAAGAGGACGTAATGTACTTGAAAAAGGCGG
CTGACGTTCTGAAAGATCAAGTTGATGAGATCCTTGACCTGATGGGTGGTTGGCTGGCATCAAATGA
GCATTTGATTTATTACTTCTCCAATCCGGATACAGGAGCGCCTATTAAAGAATACCTGGAACGTGTA
CGCGCTCGCTGTGTAGCCTGGGTTCTGGACACTACCTGCCGCGACTATAACCGTGAATGGTTAGACT
ACCAGTACGAAGTTGGGCTTCGTCATCACCGTTCAAAGAAAGGGGTCACAGACGGAGTACGCACCG
TGCCCAATACCCCACTTCGTTATCTTATCGCAGAGATCTATCCTCTGACCGCCACTATCAAGCCATTT
TTAGCTAAGAAAGGTGGCTCTCCGGAGGACATCGAAGGGATGTACAACGCTTGGCTCAAGTCTGTA
GTTCTACGAGTTGCCATCTGGTCACACCCTTATACTAAGGAGAATGACCGGCTCGAGCACCACCACC
ACCACCACTGA

MTPSDISGYDYGHVEKSPITDLEFDLLKKTVMLGEEDVMYLKKAADVLKDQVDEILDLMGGWLASNEH
LIYYFSNPDTGAPIKEYLERVRARCVAWVLDTTCRDYNREWLDYQYEVGLRHHRSKKGVTDGVRTVPN
TPLRYLIAEIYPLTATIKPFLAKKGGSPEDIEGMYNAWLKSVVLRVAIWSHPYTKENDRLEHHHHHH*

G9.1-5473

ATGACTCCCTCGGACATCTCGGGGTATGATTATGGGCATGTCGAGAAGTCACCCATCACGGACCTTG
AGTTTGACCTTCTGAAGAAGACTGTCATGTTAGGTGAAGAGGACGTAATGTACTTGAAAAAGGCGG
CTGACGTTCTGAAAGATCAAGTTGATGAGATCCTTGACCTGATGGGTGGTTGGCTGGCATCAAATGA
GCATTTGATTTATTACTTCTCCAATCCGGATACAGGAGCGCCTATTAAAGAATACCTGGAACGTGTA
CGCGCTCGCTGTGTAGCCTGGGTTCTGGACACTACCTGCCGCGACTATAACCGTGAATGGTTAGACT
ACCAGTACGAAGTTGGGCTTCGTCATCACCGTTCAAAGAAAGGGGTCACAGACGGAGTACGCACCG
TGCCCAATACCCCACTTCGTTATCTTATCGCAGAGATCTATCCTTTTACCGCCACTATCAAGCCATTT
TTAGCTAAGAAAGGTGGCTCTCCGGAGGACATCGAAGGGATGTACAACGCTTGGCTCAAGTCTGTA
GTTCTACGAGTTGCCATCTGGTCACACCCTTATACTAAGGAGAATGACCGGCTCGAGCACCACCACC
ACCACCACTGA

MTPSDISGYDYGHVEKSPITDLEFDLLKKTVMLGEEDVMYLKKAADVLKDQVDEILDLMGGWLASNEH
LIYYFSNPDTGAPIKEYLERVRARCVAWVLDTTCRDYNREWLDYQYEVGLRHHRSKKGVTDGVRTVPN
TPLRYLIAEIYPFTATIKPFLAKKGGSPEDIEGMYNAWLKSVVLRVAIWSHPYTKENDRLEHHHHHH*

G10.1-5474

ATGACTCCCTCGGACATCTCGGGGTATGATTATGGGCATGTCGAGAAGTCACCCATCACGGACCTTG
AGTTTGACCTTCTGAAGAAGACTGTCATGTTAGGTGAAGAGGACGTAATGTACTTGAAAAAGGCGG
CTGACGTTCTGAAAGATCAAGTTGATGAGATCCTTGACCTGATGGGTGGTTGGCTGGCATCAAATGA
GCATTTGATTTATTACTTCTCCAATCCGGATACAGGAGCGCCTATTAAAGAATACATGGAACGTGTA
CGCGCTCGCTGTGTAGCCTGGGTTCTGGACACTACCTGCCGCGACTATAACCGTGAATGGTTAGACT
ACCAGTACGAAGTTGGGCTTCGTCATCACCGTTCAAAGAAAGGGGTCACAGACGGAGTACGCACCG
TGCCCAATACCCCACTTCGTTATCTTATCGCAGAGATCTATCCTTTTACCGCCACTATCAAGCCATTT
TTAGCTAAGAAAGGTGGCTCTCCGGAGGACATCGAAGGGATGTACAACGCTTGGCTCAAGTCTGTA
GTTCTATGTGTTGCCATCTGGTCACACCCTTATACTAAGGAGAATGACCGGCTCGAGCACCACCACC
ACCACCACTGA

MTPSDISGYDYGHVEKSPITDLEFDLLKKTVMLGEEDVMYLKKAADVLKDQVDEILDLMGGWLASNEH
LIYYFSNPDTGAPIKEYMERVRARCVAWVLDTTCRDYNREWLDYQYEVGLRHHRSKKGVTDGVRTVP
NTPLRYLIAEIYPFTATIKPFLAKKGGSPEDIEGMYNAWLKSVVLCVAIWSHPYTKENDRLEHHHHHH*

G11.1-5475

ATGACTCCCTCGGACATCTCGGGGTATGATTATGGGCATGTCGAGAAGTCACCCATCACGGACCTTG
AGTTTGACCTTCTGAAGAAGACTGTCATGTTAGGTGAAGAGGACGTAATGTACTTGAAAAAGGCGG
CTGACGTTCTGAAAGATCAAGTTGATGAGATCCTTGACCTGATGGGTGGTTGGCTGGCATCAAATGA
GCATTTGATTTATTACTTCTCCAATCCGGATACAGGAGCGCCTATTAAAGAATACATGGAACGTGTA
CGCGCTCGCTGTGTAGCCTGGGTTCTGGACACTACCTGCCGCGACTATAACCGTGAATGGTTAGACT
ACCAGTACGAAATTGGGCTTCGTCATCACCGTTCAAAGAAAGGGGTCACAGACGGAGTACGCACCG
TGCCCAATACCCCACTTCGTTATCTTATCGCAGAGATCTATCCTTTTACCGCCACTATCAAGCCATTT
TTAGCTAAGAAAGGTGGCTCTCCGGAGGACATCGAAGGGATGTACAACGCTTGGCTCAAGTCTGTA
GTTCTATGTGTTGCCATCTGGTCACACCCTTATACTAAGGAGAATGACCGGCTCGAGCACCACCACC
ACCACCACTGA

MTPSDISGYDYGHVEKSPITDLEFDLLKKTVMLGEEDVMYLKKAADVLKDQVDEILDLMGGWLASNEH
LIYYFSNPDTGAPIKEYMERVRARCVAWVLDTTCRDYNREWLDYQYEIGLRHHRSKKGVTDGVRTVPN
TPLRYLIAEIYPFTATIKPFLAKKGGSPEDIEGMYNAWLKSVVLCVAIWSHPYTKENDRLEHHHHHH*

6.2 Selected variants from compilation plate screening

G4-I93S-5476

ATGACTCCCTCGGACATCTCGGGGTATGATTATGGGCGTGTCGAGAAGTCACCCATCACGGACCTTG
AGTTTGACCTTCTGAAGAAGACTGTCATGTTAGGTGAAGAGGACGTAATGTACTTGAAAAAGGCGG
CTGACGTTCTGAAAGATCAAGTTGATGAGATCCTTGACCTGGCGGGTGGTTGGGCAGCATCAAATG
AGCATTTGATTTATTACTTCTCCAATCCGGATACAGGAGCGCCTATTAAAGAATACCTGGAACGTGT
ACGCGCTCGCAGTGGAGCCTGGGTTCTGGACACTACCTGCCGCGACTATAACCGTGAATGGTTAGAC
TACCAGTACGAAGTTGGGCTTCGTCATCACCGTTCAAAGAAAGGGGTCACAGACGGAGTACGCACC
GTGCCCAATACCCCACTTCGTTATCTTATCGCAGGTATCTATCCTATCACCGCCACTATCAAGCCATT
TTTAGCTAAGAAAGGTGGCTCTCCGGAGGACATCGAAGGGATGTACAACGCTTGGCTCAAGTCTGT
AGTTCTACAAGTTGCCATCTGGTCACACCCTTATACTAAGGAGAATGACCGGCTCGAGCACCACCAC
CACCACCACTGA

MTPSDISGYDYGRVEKSPITDLEFDLLKKTVMLGEEDVMYLKKAADVLKDQVDEILDLAGGWAASNEH
LIYYFSNPDTGAPIKEYLERVRARSGAWVLDTTCRDYNREWLDYQYEVGLRHHRSKKGVTDGVRTVPN
TPLRYLIAGIYPITATIKPFLAKKGGSPEDIEGMYNAWLKSVVLQVAIWSHPYTKENDRLEHHHHHH*

G4-I93T-5477

ATGACTCCCTCGGACATCTCGGGGTATGATTATGGGCGTGTCGAGAAGTCACCCATCACGGACCTTG
AGTTTGACCTTCTGAAGAAGACTGTCATGTTAGGTGAAGAGGACGTAATGTACTTGAAAAAGGCGG
CTGACGTTCTGAAAGATCAAGTTGATGAGATCCTTGACCTGGCGGGTGGTTGGGCAGCATCAAATG
AGCATTTGATTTATTACTTCTCCAATCCGGATACAGGAGCGCCTATTAAAGAATACCTGGAACGTGT
ACGCGCTCGCACGGGAGCCTGGGTTCTGGACACTACCTGCCGCGACTATAACCGTGAATGGTTAGA
CTACCAGTACGAAGTTGGGCTTCGTCATCACCGTTCAAAGAAAGGGGTCACAGACGGAGTACGCAC
CGTGCCCAATACCCCACTTCGTTATCTTATCGCAGGTATCTATCCTATCACCGCCACTATCAAGCCAT
TTTTAGCTAAGAAAGGTGGCTCTCCGGAGGACATCGAAGGGATGTACAACGCTTGGCTCAAGTCTGT
AGTTCTACAAGTTGCCATCTGGTCACACCCTTATACTAAGGAGAATGACCGGCTCGAGCACCACCAC
CACCACCACTGA

MTPSDISGYDYGRVEKSPITDLEFDLLKKTVMLGEEDVMYLKKAADVLKDQVDEILDLAGGWAASNEH
LIYYFSNPDTGAPIKEYLERVRARTGAWVLDTTCRDYNREWLDYQYEVGLRHHRSKKGVTDGVRTVPN
TPLRYLIAGIYPITATIKPFLAKKGGSPEDIEGMYNAWLKSVVLQVAIWSHPYTKENDRLEHHHHHH*

G5OEt-A59I-5478

ATGACTCCCTCGGACATCTCGGGGTATGATTATGGGCGTGTCGAGAAGTCACCCGTCACGGACCTCG
AGTTTGACCTCCTGAAGAAGACTGTCATGTTTGGTGAAGAGGACGTAATGTACTTGAAAAAGGCGG
CTGACGTTCTGAAAGATCAAGTTGATGAGATCCTTGACCTGATTGGTGGTTGGGCAGCATCAAATGA
GCATTTGGTTTATTACTTCTCCAATCCGGATACAGGAGCGCCTATTAAAGAATACCTGGAACGTGTA
CGCGCTCGCTGTGTAGCCTGGGTTCTGGACACCACCTGCCGCGACTATAACCGTGAATGGTTAGACT
ACCAGTACGAAGTTGGGCTTCGTCATCACCGTTCAAAGAAAGGGGTCACAGACGGAGTACGCACCG
TGCCCAATACCCCACTTCGATATCTTATCGCAGGTATCTATCCTCTGACCGCCACTATCAAGCCATTT
TTAGCTAAGAAAGGTGGCTCTCCGGAGGACATCGAAGGGATGTACAACGCTTGGCTCAAGTCTGTA
GTTCTACAAGTTGCCATCTGGTCACACCCTTATACTAAGGAGAATGACCGGCTCGAGCACCACCACC
ACCACCACTGA

MTPSDISGYDYGRVEKSPVTDLEFDLLKKTVMFGEEDVMYLKKAADVLKDQVDEILDLIGGWAASNEH
LVYYFSNPDTGAPIKEYLERVRARCVAWVLDTTCRDYNREWLDYQYEVGLRHHRSKKGVTDGVRTVP
NTPLRYLIAGIYPLTATIKPFLAKKGGSPEDIEGMYNAWLKSVVLQVAIWSHPYTKENDRLEHHHHHH*

G5OEt-A59L-5479

ATGACTCCCTCGGACATCTCGGGGTATGATTATGGGCGTGTCGAGAAGTCACCCGTCACGGACCTCG
AGTTTGACCTCCTGAAGAAGACTGTCATGTTTGGTGAAGAGGACGTAATGTACTTGAAAAAGGCGG
CTGACGTTCTGAAAGATCAAGTTGATGAGATCCTTGACCTGCTTGGTGGTTGGGCAGCATCAAATGA
GCATTTGGTTTATTACTTCTCCAATCCGGATACAGGAGCGCCTATTAAAGAATACCTGGAACGTGTA
CGCGCTCGCTGTGTAGCCTGGGTTCTGGACACCACCTGCCGCGACTATAACCGTGAATGGTTAGACT
ACCAGTACGAAGTTGGGCTTCGTCATCACCGTTCAAAGAAAGGGGTCACAGACGGAGTACGCACCG
TGCCCAATACCCCACTTCGATATCTTATCGCAGGTATCTATCCTCTGACCGCCACTATCAAGCCATTT
TTAGCTAAGAAAGGTGGCTCTCCGGAGGACATCGAAGGGATGTACAACGCTTGGCTCAAGTCTGTA
GTTCTACAAGTTGCCATCTGGTCACACCCTTATACTAAGGAGAATGACCGGCTCGAGCACCACCACC
ACCACCACTGA

MTPSDISGYDYGRVEKSPVTDLEFDLLKKTVMFGEEDVMYLKKAADVLKDQVDEILDLLGGWAASNEH
LVYYFSNPDTGAPIKEYLERVRARCVAWVLDTTCRDYNREWLDYQYEVGLRHHRSKKGVTDGVRTVP
NTPLRYLIAGIYPLTATIKPFLAKKGGSPEDIEGMYNAWLKSVVLQVAIWSHPYTKENDRLEHHHHHH*

G5OEt-A59T-5480

ATGACTCCCTCGGACATCTCGGGGTATGATTATGGGCGTGTCGAGAAGTCACCCGTCACGGACCTCG
AGTTTGACCTCCTGAAGAAGACTGTCATGTTTGGTGAAGAGGACGTAATGTACTTGAAAAAGGCGG
CTGACGTTCTGAAAGATCAAGTTGATGAGATCCTTGACCTGACGGGTGGTTGGGCAGCATCAAATG
AGCATTTGGTTTATTACTTCTCCAATCCGGATACAGGAGCGCCTATTAAAGAATACCTGGAACGTGT
ACGCGCTCGCTGTGTAGCCTGGGTTCTGGACACCACCTGCCGCGACTATAACCGTGAATGGTTAGAC
TACCAGTACGAAGTTGGGCTTCGTCATCACCGTTCAAAGAAAGGGGTCACAGACGGAGTACGCACC
GTGCCCAATACCCCACTTCGATATCTTATCGCAGGTATCTATCCTCTGACCGCCACTATCAAGCCATT
TTTAGCTAAGAAAGGTGGCTCTCCGGAGGACATCGAAGGGATGTACAACGCTTGGCTCAAGTCTGT
AGTTCTACAAGTTGCCATCTGGTCACACCCTTATACTAAGGAGAATGACCGGCTCGAGCACCACCAC
CACCACCACTGA

MTPSDISGYDYGRVEKSPVTDLEFDLLKKTVMFGEEDVMYLKKAADVLKDQVDEILDLTGGWAASNEH
LVYYFSNPDTGAPIKEYLERVRARCVAWVLDTTCRDYNREWLDYQYEVGLRHHRSKKGVTDGVRTVP
NTPLRYLIAGIYPLTATIKPFLAKKGGSPEDIEGMYNAWLKSVVLQVAIWSHPYTKENDRLEHHHHHH*

G5OEt-L86Q-5481

ATGACTCCCTCGGACATCTCGGGGTATGATTATGGGCGTGTCGAGAAGTCACCCGTCACGGACCTCG
AGTTTGACCTCCTGAAGAAGACTGTCATGTTTGGTGAAGAGGACGTAATGTACTTGAAAAAGGCGG
CTGACGTTCTGAAAGATCAAGTTGATGAGATCCTTGACCTGGCGGGTGGTTGGGCAGCATCAAATG
AGCATTTGGTTTATTACTTCTCCAATCCGGATACAGGAGCGCCTATTAAAGAATACCAGGAACGTGT
ACGCGCTCGCTGTGTAGCCTGGGTTCTGGACACCACCTGCCGCGACTATAACCGTGAATGGTTAGAC
TACCAGTACGAAGTTGGGCTTCGTCATCACCGTTCAAAGAAAGGGGTCACAGACGGAGTACGCACC
GTGCCCAATACCCCACTTCGATATCTTATCGCAGGTATCTATCCTCTGACCGCCACTATCAAGCCATT
TTTAGCTAAGAAAGGTGGCTCTCCGGAGGACATCGAAGGGATGTACAACGCTTGGCTCAAGTCTGT
AGTTCTACAAGTTGCCATCTGGTCACACCCTTATACTAAGGAGAATGACCGGCTCGAGCACCACCAC
CACCACCACTGA

MTPSDISGYDYGRVEKSPVTDLEFDLLKKTVMFGEEDVMYLKKAADVLKDQVDEILDLAGGWAASNE
HLVYYFSNPDTGAPIKEYQERVRARCVAWVLDTTCRDYNREWLDYQYEVGLRHHRSKKGVTDGVRTV
PNTPLRYLIAGIYPLTATIKPFLAKKGGSPEDIEGMYNAWLKSVVLQVAIWSHPYTKENDRLEHHHHHH*

G5OEt-C93S-5482

ATGACTCCCTCGGACATCTCGGGGTATGATTATGGGCGTGTCGAGAAGTCACCCGTCACGGACCTCG
AGTTTGACCTCCTGAAGAAGACTGTCATGTTTGGTGAAGAGGACGTAATGTACTTGAAAAAGGCGG
CTGACGTTCTGAAAGATCAAGTTGATGAGATCCTTGACCTGGCGGGTGGTTGGGCAGCATCAAATG
AGCATTTGGTTTATTACTTCTCCAATCCGGATACAGGAGCGCCTATTAAAGAATACCTGGAACGTGT
ACGCGCTCGCAGTGTAGCCTGGGTTCTGGACACCACCTGCCGCGACTATAACCGTGAATGGTTAGAC
TACCAGTACGAAGTTGGGCTTCGTCATCACCGTTCAAAGAAAGGGGTCACAGACGGAGTACGCACC
GTGCCCAATACCCCACTTCGATATCTTATCGCAGGTATCTATCCTCTGACCGCCACTATCAAGCCATT
TTTAGCTAAGAAAGGTGGCTCTCCGGAGGACATCGAAGGGATGTACAACGCTTGGCTCAAGTCTGT
AGTTCTACAAGTTGCCATCTGGTCACACCCTTATACTAAGGAGAATGACCGGCTCGAGCACCACCAC
CACCACCACTGA

MTPSDISGYDYGRVEKSPVTDLEFDLLKKTVMFGEEDVMYLKKAADVLKDQVDEILDLAGGWAASNE
HLVYYFSNPDTGAPIKEYLERVRARSVAWVLDTTCRDYNREWLDYQYEVGLRHHRSKKGVTDGVRTV
PNTPLRYLIAGIYPLTATIKPFLAKKGGSPEDIEGMYNAWLKSVVLQVAIWSHPYTKENDRLEHHHHHH*

G11.1-M59L-5483

ATGACTCCCTCGGACATCTCGGGGTATGATTATGGGCATGTCGAGAAGTCACCCATCACGGACCTTG
AGTTTGACCTTCTGAAGAAGACTGTCATGTTAGGTGAAGAGGACGTAATGTACTTGAAAAAGGCGG
CTGACGTTCTGAAAGATCAAGTTGATGAGATCCTTGACCTGCTTGGTGGTTGGCTGGCATCAAATGA
GCATTTGATTTATTACTTCTCCAATCCGGATACAGGAGCGCCTATTAAAGAATACATGGAACGTGTA
CGCGCTCGCTGTGTAGCCTGGGTTCTGGACACTACCTGCCGCGACTATAACCGTGAATGGTTAGACT
ACCAGTACGAAATTGGGCTTCGTCATCACCGTTCAAAGAAAGGGGTCACAGACGGAGTACGCACCG
TGCCCAATACCCCACTTCGTTATCTTATCGCAGAGATCTATCCTTTTACCGCCACTATCAAGCCATTT
TTAGCTAAGAAAGGTGGCTCTCCGGAGGACATCGAAGGGATGTACAACGCTTGGCTCAAGTCTGTA
GTTCTATGTGTTGCCATCTGGTCACACCCTTATACTAAGGAGAATGACCGGCTCGAGCACCACCACC
ACCACCACTGA

MTPSDISGYDYGHVEKSPITDLEFDLLKKTVMLGEEDVMYLKKAADVLKDQVDEILDLLGGWLASNEH
LIYYFSNPDTGAPIKEYMERVRARCVAWVLDTTCRDYNREWLDYQYEIGLRHHRSKKGVTDGVRTVPN
TPLRYLIAEIYPFTATIKPFLAKKGGSPEDIEGMYNAWLKSVVLCVAIWSHPYTKENDRLEHHHHHH*

G11.1-F149V-5484

ATGACTCCCTCGGACATCTCGGGGTATGATTATGGGCATGTCGAGAAGTCACCCATCACGGACCTTG
AGTTTGACCTTCTGAAGAAGACTGTCATGTTAGGTGAAGAGGACGTAATGTACTTGAAAAAGGCGG
CTGACGTTCTGAAAGATCAAGTTGATGAGATCCTTGACCTGATGGGTGGTTGGCTGGCATCAAATGA
GCATTTGATTTATTACTTCTCCAATCCGGATACAGGAGCGCCTATTAAAGAATACATGGAACGTGTA
CGCGCTCGCTGTGTAGCCTGGGTTCTGGACACTACCTGCCGCGACTATAACCGTGAATGGTTAGACT
ACCAGTACGAAATTGGGCTTCGTCATCACCGTTCAAAGAAAGGGGTCACAGACGGAGTACGCACCG
TGCCCAATACCCCACTTCGTTATCTTATCGCAGAGATCTATCCTGTTACCGCCACTATCAAGCCATTT
TTAGCTAAGAAAGGTGGCTCTCCGGAGGACATCGAAGGGATGTACAACGCTTGGCTCAAGTCTGTA
GTTCTATGTGTTGCCATCTGGTCACACCCTTATACTAAGGAGAATGACCGGCTCGAGCACCACCACC
ACCACCACTGA

MTPSDISGYDYGHVEKSPITDLEFDLLKKTVMLGEEDVMYLKKAADVLKDQVDEILDLMGGWLASNEH
LIYYFSNPDTGAPIKEYMERVRARCVAWVLDTTCRDYNREWLDYQYEIGLRHHRSKKGVTDGVRTVPN
TPLRYLIAEIYPVTATIKPFLAKKGGSPEDIEGMYNAWLKSVVLCVAIWSHPYTKENDRLEHHHHHH*

G11.1-C181R-5485

ATGACTCCCTCGGACATCTCGGGGTATGATTATGGGCATGTCGAGAAGTCACCCATCACGGACCTTG
AGTTTGACCTTCTGAAGAAGACTGTCATGTTAGGTGAAGAGGACGTAATGTACTTGAAAAAGGCGG
CTGACGTTCTGAAAGATCAAGTTGATGAGATCCTTGACCTGATGGGTGGTTGGCTGGCATCAAATGA
GCATTTGATTTATTACTTCTCCAATCCGGATACAGGAGCGCCTATTAAAGAATACATGGAACGTGTA
CGCGCTCGCTGTGTAGCCTGGGTTCTGGACACTACCTGCCGCGACTATAACCGTGAATGGTTAGACT
ACCAGTACGAAATTGGGCTTCGTCATCACCGTTCAAAGAAAGGGGTCACAGACGGAGTACGCACCG
TGCCCAATACCCCACTTCGTTATCTTATCGCAGAGATCTATCCTTTTACCGCCACTATCAAGCCATTT
TTAGCTAAGAAAGGTGGCTCTCCGGAGGACATCGAAGGGATGTACAACGCTTGGCTCAAGTCTGTA
GTTCTACGTGTTGCCATCTGGTCACACCCTTATACTAAGGAGAATGACCGGCTCGAGCACCACCACC
ACCACCACTGA

MTPSDISGYDYGHVEKSPITDLEFDLLKKTVMLGEEDVMYLKKAADVLKDQVDEILDLMGGWLASNEH
LIYYFSNPDTGAPIKEYMERVRARCVAWVLDTTCRDYNREWLDYQYEIGLRHHRSKKGVTDGVRTVPN
TPLRYLIAEIYPFTATIKPFLAKKGGSPEDIEGMYNAWLKSVVLRVAIWSHPYTKENDRLEHHHHHH*

6.3 Nitrene P–H lineage

G5N-5486

ATGACTCCCTCGGACATCTCGGGGTATGATTATGGGCGTGTCGAGAAGTCACCCATCACGGACCTTG
AGTTTGACCTTCTGAAGAAGACTGTCATGTTAGGTGAAGAGGACGTAATGTACTTGAAAAAGGCGG
CTGACGTTCTGAAAGATCAAGTTGATGAGATCCTTGACCTGGCGGGTGGTTGGGCAGCATCAAATG
AGCATTTGATTTATTACTTCTCCAATCCGGATACAGGAGCGCCTATTAAAGAATACCTGGAACGTGT
ACGCGCTCGCCTGGGAGCCTGGGTTCTGGACACTACCTGCCGCGACTATAACCGTGAATGGTTAGAC
TACCAGTACGAAGTTGGGCTTCGTCATCACCGTTCAAAGAAAGGGGTCACAGACGGAGTACGCACC
GTGCCCAATACCCCACTTCGTTATCTTATCGCAGGTATCTATCCTATCACCGCCACTATCAAGCCATT
TTTAGCTAAGAAAGGTGGCTCTCCGGAGGACATCGAAGGGATGTACAACGCTTGGCTCAAGTCTGT
AGTTCTACAAGTTGCCATCTGGTCACACCCTTATACTAAGGAGAATGACCGGCTCGAGCACCACCAC
CACCACCACTGA

MTPSDISGYDYGRVEKSPITDLEFDLLKKTVMLGEEDVMYLKKAADVLKDQVDEILDLAGGWAASNEH
LIYYFSNPDTGAPIKEYLERVRARLGAWVLDTTCRDYNREWLDYQYEVGLRHHRSKKGVTDGVRTVPN
TPLRYLIAGIYPITATIKPFLAKKGG SPEDIEGMYNAWLKSVVLQVAIWSHPYTKENDRLEHHHHHH*

G6N-5487

ATGACTCCCTCGGACATCTCGGGGTATGATTATGGGCGTGTCGAGAAGTCACCCATCACGGACCTTG
AGTTTGACCTTCTGAAGAAGACTGTCATGTTAGGTGAAGAGGACGTAATGTACTTGAAAAAGGCGG
CTGACGTTCTGAAAGATCAAGTTGATGAGATCCTTGACCTGGCGGGTGGTTGGGCAGCATCAAATG
AGCATTTGATTTATTACTTCTCCAATCCGGATACAGGAGCGCCTATTAAAGAATACCTGGAACGTGT
ACGCGCTCGCCTGGGAGCCTGGGTTCTGGACACTACCTGCCGCGACTATAACCGTGAATGGTTAGAC
TACCAGTACGAAGTTGGGCTTCGTCATCACCGTTCAAAGAAAGGGGTCACAGACGGAGTACGCACC
GTGCCCAATACCCCACTTCGTTATCTTATCGCAGGTATCTATCCTATCACCGCCACTATCAAGCCATT
TTTAGCTAAGAAAGGTGGCTCTCCGGAGGACATCGAAGGGATGTACAACGCTTGGCTCAAGTCTGT
AGTTCTACAAGTTGCCATCTGGTCACACCGGTATACTAAGGAGAATGACCGGCTCGAGCACCACCA
CCACCACCACTGA

MTPSDISGYDYGRVEKSPITDLEFDLLKKTVMLGEEDVMYLKKAADVLKDQVDEILDLAGGWAASNEH
LIYYFSNPDTGAPIKEYLERVRARLGAWVLDTTCRDYNREWLDYQYEVGLRHHRSKKGVTDGVRTVPN
TPLRYLIAGIYPITATIKPFLAKKGGSPEDIEGMYNAWLKSVVLQVAIWSHRYTKENDRLEHHHHHH*

G7N-5488

ATGACTCCCTCGGACATCTCGGGGTATGATTATGGGCGTGTCGAGAAGTCACCCATCACGGACCTTG
AGTTTGACCTTCTGAAGAAGACTGTCATGTTAGGTGAAGAGGACGTAATGTACTTGAAAAAGGCGG
CTGACGTTCTGAAAGATCAAGTTGATGAGATCCTTGACCAGGCGGGTGGTTGGGCAGCATCAAATG
AGCATTTGATTTGGTACTTCTCCAATCCGGATACAGGAGCGCCTATTAAAGAATACCTGGAACGTGT
ACGCGCTCGCCTGGGAGCCTGGGTTCTGGACACTACCTGCCGCGACTATAACCGTGAATGGTTAGAC
TACCAGTACGAAGTTGGGCTTCGTCATCACCGTTCAAAGAAAGGGGTCACTGACGGAGTACGCACC
GTGCCCAATACCCCACTTCGTTATCTTATCGCAGGTATCTATCCTATCACCGCCACTATCAAGCCATT
TTTAGCTAAGAAAGGTGGCTCTCCGGAGGACATCGAAGGGATGTACAACGCTTGGCTCAAGTCTGT
AGTTCTACAAGTTGCCATCTGGTCACACCGGTATACTAAGGAGAATGACCGGCTCGAGCACCACCA
CCACCACCACTGA

MTPSDISGYDYGRVEKSPITDLEFDLLKKTVMLGEEDVMYLKKAADVLKDQVDEILDQAGGWAASNEH
LIWYFSNPDTGAPIKEYLERVRARLGAWVLDTTCRDYNREWLDYQYEVGLRHHRSKKGVTDGVRTVP
NTPLRYLIAGIYPITATIKPFLAKKGGSPEDIEGMYNAWLKSVVLQVAIWSHRYTKENDRLEHHHHHH*

G6N.1-5489

ATGACTCCCTCGGACATCTCGGGGTATGATTATGGGCGTGTCGAGAAGTCACCCATCACGGACCTTG
AGTTTGACCTTCTGAAGAAGACTGTCATGTTAGGTGAAGAGGACGTAATGTACTTGAAAAAGGCGG
CTGACGTTCTGAAAGATCAAGTTGATGAGATCCTTGACCTGGCGGGTGGTTGGGCAGCATCAAATG
AGCATTTGATTTATTACTTCTCCAATCCGGATACAGGAGCGCCTATTAAAGAATACCTGGAACGTGT
ACGCGCTCGCCTGGGAGCCTGGGTTCTGGACACTACCTGCCGCGACTATAACCGTGAATGGTTAGAC
TACCAGTACGAAGTTGGGCTTCGTCATCACCGTTCAAAGAAAGGGGTCACAGACGGAGTACGCACC
GTGCCCAATACCCCACTTCGTTATCTTATCGCAGCTATCTATCCTATGACCGCCACTATCAAGCCATT
TTTAGCTAAGAAAGGTGGCTCTCCGGAGGACATCGAAGGGATGTACAACGCTTGGCTCAAGTCTGT
AGTTCTACAAGTTGCCATCTGGTCACACCCTTATACTAAGGAGAATGACCGGCTCGAGCACCACCAC
CACCACCACTGA

MTPSDISGYDYGRVEKSPITDLEFDLLKKTVMLGEEDVMYLKKAADVLKDQVDEILDLAGGWAASNEH
LIYYFSNPDTGAPIKEYLERVRARLGAWVLDTTCRDYNREWLDYQYEVGLRHHRSKKGVTDGVRTVPN
TPLRYLIAAIYPMTATIKPFLAKKGGSPEDIEGMYNAWLKSVVLQVAIWSHPYTKENDRLEHHHHHH*

7. Substrate Scope

7.1 PhMeP(O)–CH_2CO_2Et (1b)

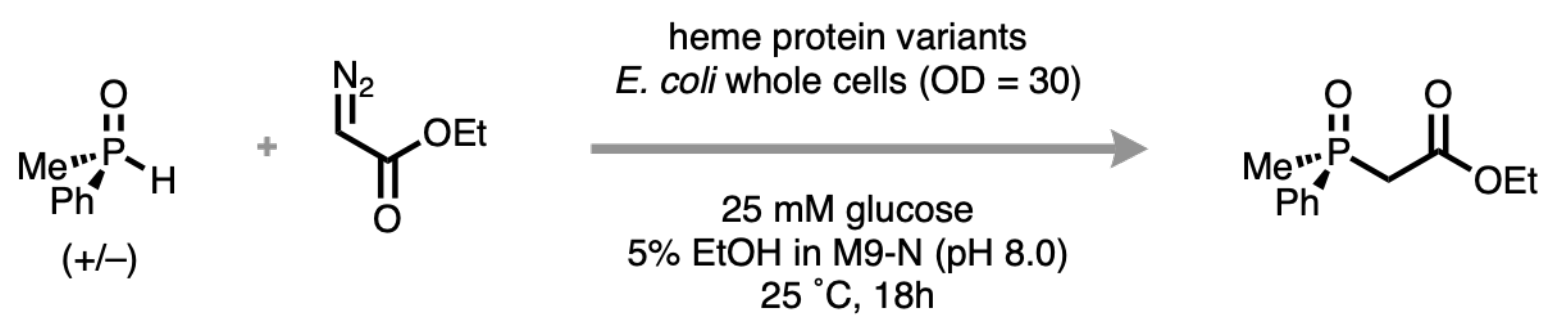

Reactions were performed according to the validation procedure, see Section 5.1 for assay yields and enantioselectivity across the whole lineage. Absolute configuration of product assigned based on the assignment and consumption of substrate (Section 8.1 and Section 8.4) as well as product isolation (Section 8.2).

Large Scale (1 mmol): The standard validation procedure was followed using a larger scale, except the reaction was performed aerobically and stirred using a magnetic stir bar (800 rpm). Normalized cell suspension (G4, 36 mL, OD_{600} = 30, M9-N, pH 8.0) was added to a 100-mL round bottom flask. To the flask were added glucose (2 mL, 500 mM, $[glc]_{final}$ = 25 mM) and a solution of PhMeP(O)H and EDA in EtOH (2 mL, 500 mM PhMeP(O)H, 375 mM EDA, $[P]_{final}$ = 25 mM, $[EDA]_{final}$ = 18.75 mM). The flask was sealed with a septum and stirred at room temperature for 24 h. The reaction was quenched with IPA (4 mL), and a 400-µL aliquot was removed for chiral HPLC analysis (99% ee). The reaction was extracted with EtOAc (4x 30 mL) – centrifugation at 5000xg for 1 minute was used to aid layer formation. The combined organics were dried with Na_2SO_4 and solvent removed *in vacuo*. The reaction outcome was analyzed by adding nitrobenzene as an analytical external standard (40 µL), and an aliquot was removed to determine the reaction yield (^{1}H NMR Yield, 30%).

Column: The resulting crude residue was purified by silica gel chromatography (10g SiliaFlash R60 SiO_2, packed w/ 80% EtOAc). The crude residue was loaded in a minimal amount of DCM (200 µL x 3), and the product was eluted with a gradient (0–15% MeOH in EtOAc over 15 CV). The fractions with product were concentrated in vacuo (30 ˚C). The product was obtained as a clear oil (65.5 mg, 29%, 98% ee).

^{1}H NMR: (400 MHz, $CDCl_3$) δ 7.80 – 7.70 (m, 2H), 7.61 – 7.54 (m, 1H), 7.54 – 7.42 (m, 2H), 4.09 (qd, *J* = 7.2, 3.3 Hz, 2H), 3.28 – 2.93 (m, 2H), 1.94 (d, *J* = 13.5 Hz, 3H), 1.15 (t, *J* = 7.2 Hz, 3H).

^{31}P NMR: (162 MHz, $CDCl_3$) δ 31.89.

Optical Rotation: $[\alpha]^{23}_D$ = – 27.5 (c = 1, MeOH).

Reverse Phase HPLC-MS: Agilent Poroshell 120 EC-C18, 1.5 mL/min, [A] 0.1% AcOH in Water, [B] 0.1% AcOH in Acetonitrile, scan and single ion monitoring $[M+H]^+$, gradient: 0-0.2 min: 5-50% B; 0.2-0.4 min: 5–20% B; 0.4–2.5 min: 20–50% B; 2.5–2.6 min: 50–95% B; 2.6–3.0 min: 95% B; 3.0–3.1 min: 95–5% B; 3.1–3.5 min: 5% B.

Chiral HPLC: Chiralpak IB and IC in series (Hex/EtOH = 85:15, 1 mL/min). Using the columns within our inventory, two columns were necessary to resolve the enantiomers of both RSM and product (Chiralpak IC is sufficient to resolve the enantiomers of product, but the peaks overlap with RSM).

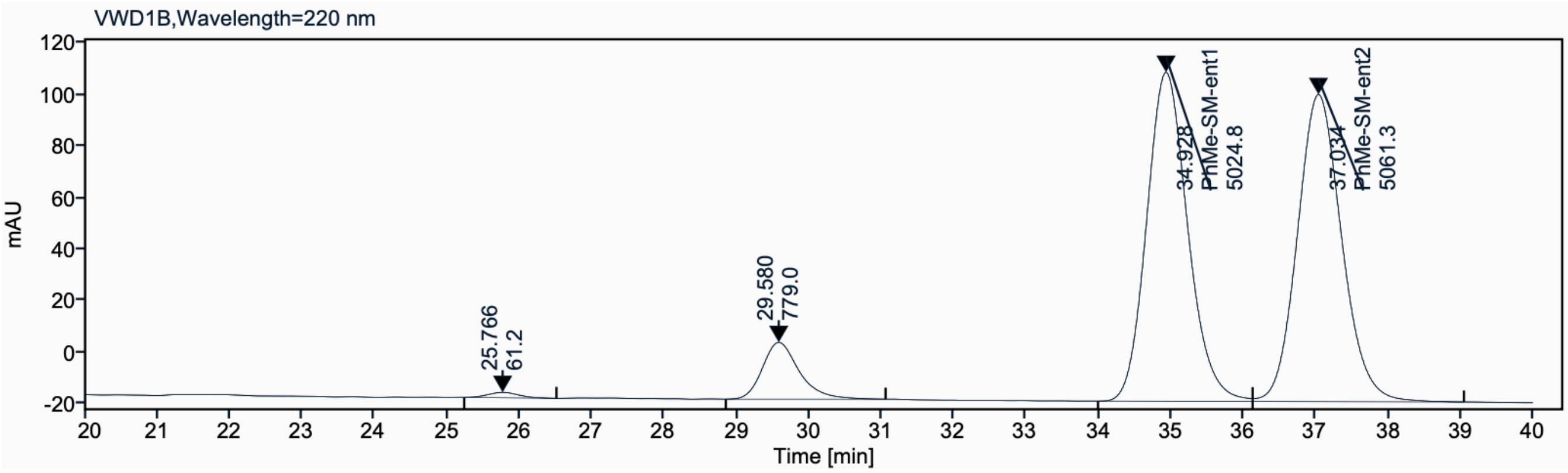

Name	RT [min]	Area
PhMe-SM-ent1	34.928	5024.7772
PhMe-SM-ent2	37.034	5061.3113

Figure S12: Chiral HPLC trace of racemic PhMeP(O)H.

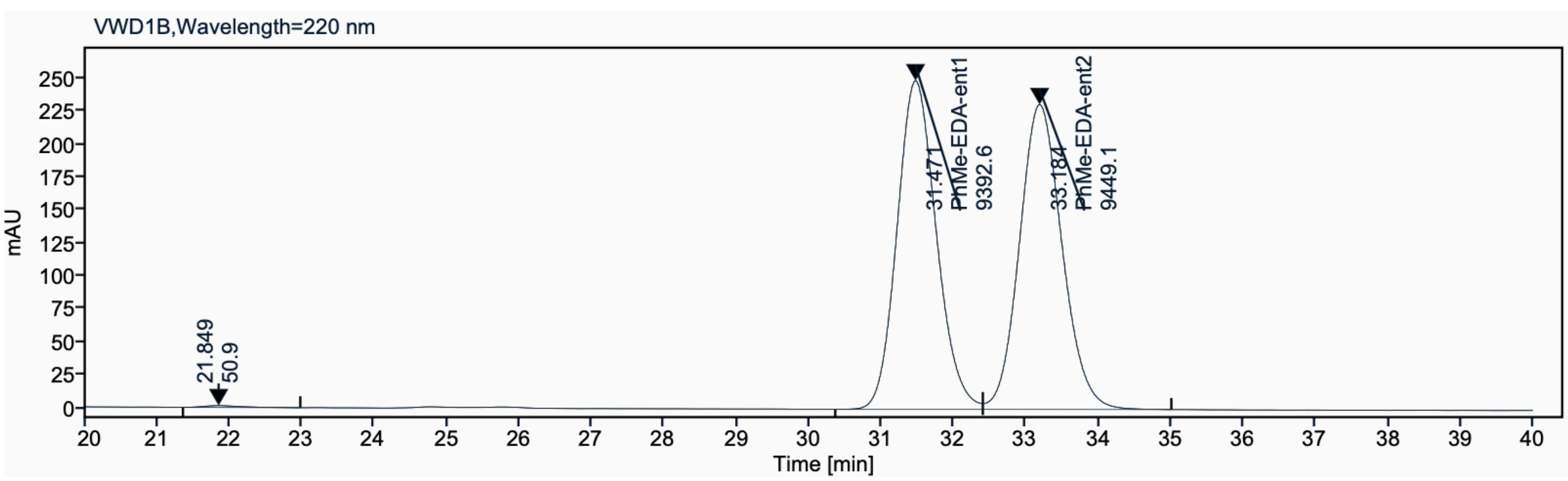

Name	RT [min]	Area
PhMe-EDA-ent1	31.471	9392.6117
PhMe-EDA-ent2	33.184	9449.0648

Figure S13: Chiral HPLC trace of racemic PhMeP(O)–CH_2CO_2Et.

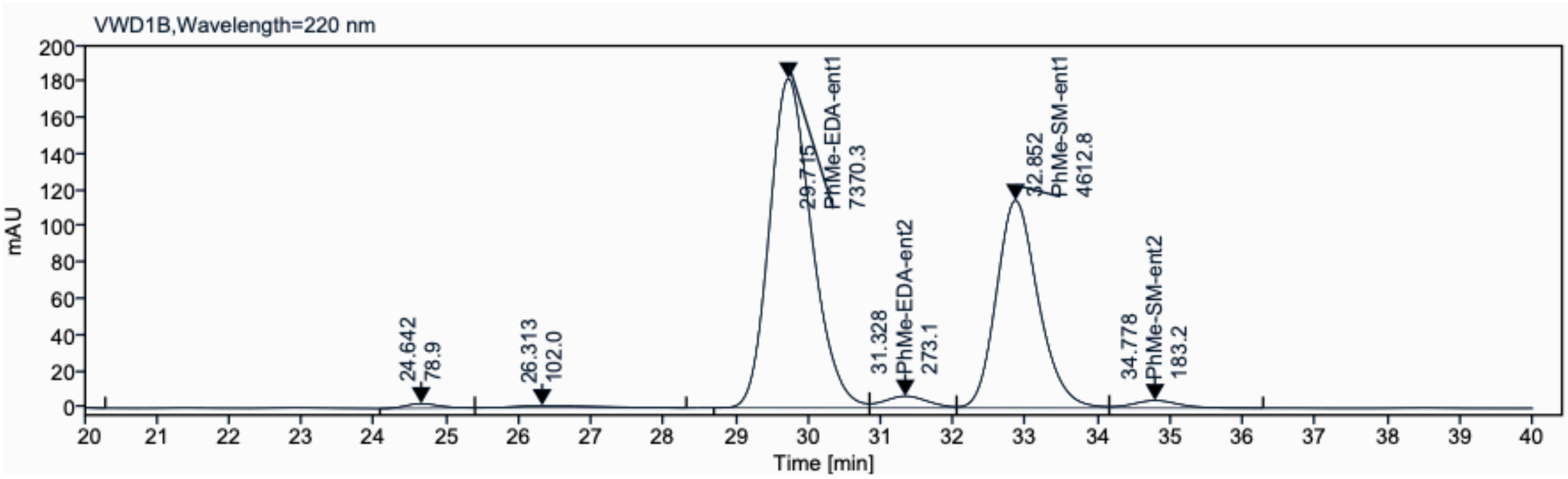

Name	RT [min]	Area
PhMe-EDA-ent1	29.715	7370.2894
PhMe-EDA-ent2	31.328	273.0970
PhMe-SM-ent1	32.852	4612.8051
PhMe-SM-ent2	34.778	183.2075

Figure S14: Chiral HPLC trace of reaction catalyzed by G4, PhMeP(O)H and EDA.

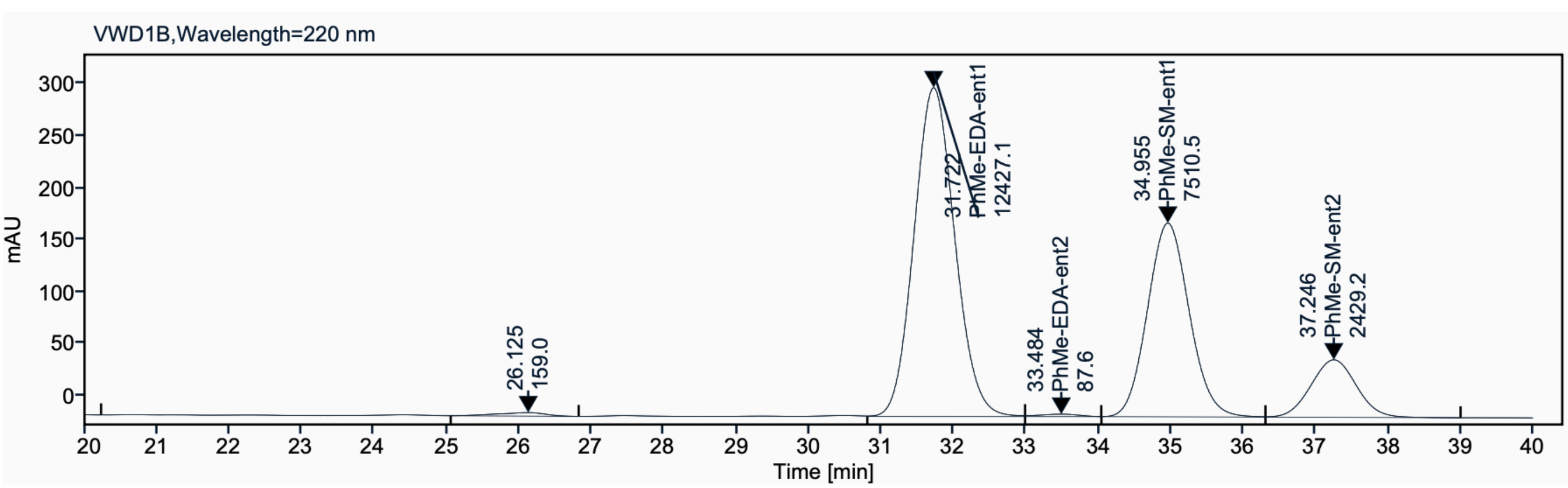

Name	RT [min]	Area
PhMe-EDA-ent1	31.722	12427.1442
PhMe-EDA-ent2	33.484	87.5707
PhMe-SM-ent1	34.955	7510.5263
PhMe-SM-ent2	37.246	2429.2309

Figure S15: Chiral HPLC trace of large-scale reaction catalyzed by G4, PhMeP(O)H and EDA.

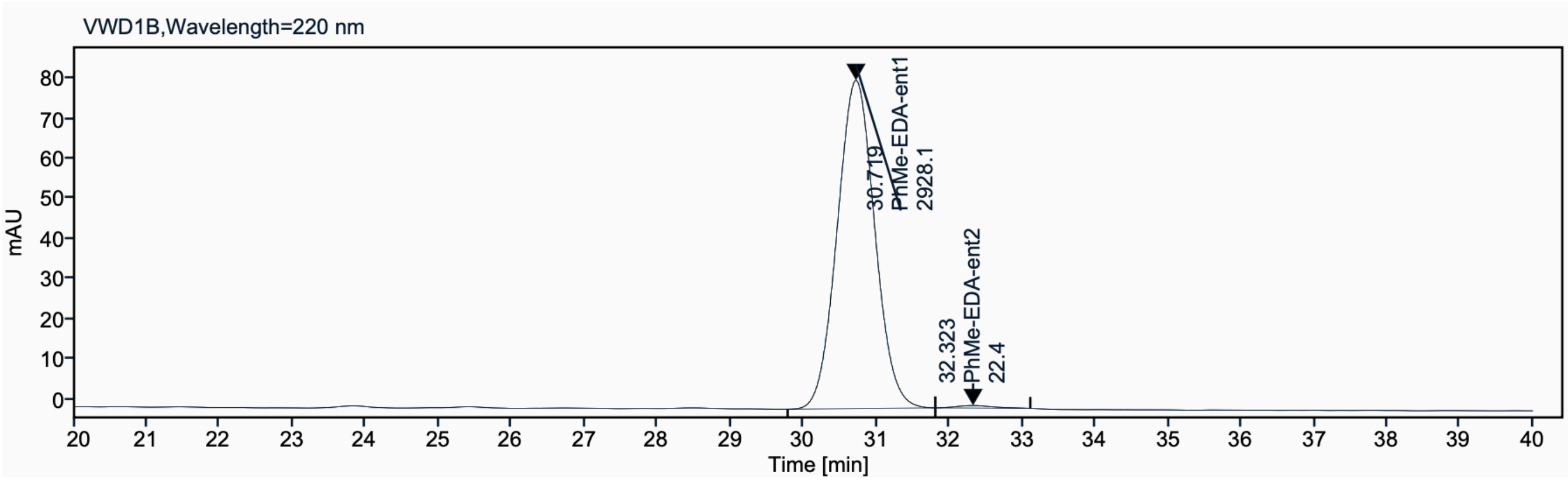

Name	RT [min]	Area
PhMe-EDA-ent1	30.719	2928.0991
PhMe-EDA-ent2	32.323	22.3889

Figure S16: Chiral HPLC trace of purified product from large scale reaction catalyzed by G4, PhMeP(O)H and EDA.

7.2 PhBnP(O)–CH_2CO_2Et (3b)

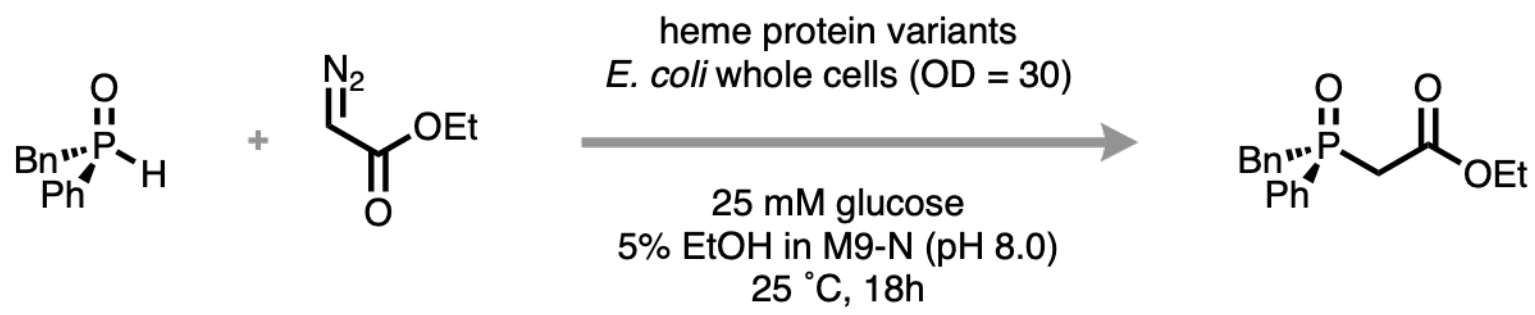

PhBnP(O)H and EDA was screened against the Master Compilation Plate (P11) and three variants were chosen with which to perform validation, G5OEt, G4-I93T, and G6.1. Absolute configuration of product assigned based on the assignment and consumption of substrate (Section 8.4).

Reactions were performed according to the validation procedure (three technical replicates for assay yield; two technical replicates for enantioselectivity).

Table S37: Yield and enantioselectivity of variants and selected reaction conditions for the reactions of PhBnP(O)H and EDA.

Variant/conditions	Avg. Yield (%)	Std Dev Yield (%)	Avg. E.E. (%)	Std Dev E.E. (%)
G5OEt	56	1	67	< 1%
G4-I93T	49	2	90	< 1%
G6.1	49	1	-100	< 1%

Large Scale (0.4 mmol): The standard validation procedure was followed using a larger scale, except the reaction was performed in a 40-mL vial. A 40-mL vial was charged with normalized cell suspension (G6.1, 18 mL, OD_{600} = 30, M9-N, pH 8.0) and glucose (1 mL, 500 mM, $[glc]_{final}$ = 25 mM). The vial was brought into the anaerobic COY chamber, and a solution of PhBnP(O)H and EDA in EtOH (1 mL, 400 mM PhMeP(O)H, 300 mM EDA, $[P]_{final}$ = 20 mM, $[EDA]_{final}$ = 15 mM) was added. The vial was sealed with a PTFE lined cap and shaken at room temperature for 24 h. The reaction was quenched with IPA (2 mL) and a 400-µL aliquot was removed for chiral HPLC analysis (>99% ee). The reaction was extracted with EtOAc (4x 20 mL) – centrifugation at 5000xg for 1 minute was used to aid layer formation. The combined organics were dried with Na_2SO_4 and solvent removed *in vacuo*. The reaction outcome was analyzed by adding nitrobenzene as an analytical external standard (20 µL), and an aliquot was removed to determine the reaction yield (^{1}H NMR Yield, 46%, 38% RSM).

Column: The resulting crude residue was purified by silica gel chromatography (10g SiO_2, packed w/ 75% (5% IPA in EtOAc) in Hex). The crude residue was loaded in a minimal amount of DCM (200 µL x 3), and the product was eluted with a gradient (75–100% (5% IPA in EtOAc) in Hex over 15 CV). The fractions with product were concentrated in vacuo (30 ˚C). The product was obtained as a clear oil (47.6 mg, 39%, >99% ee). Residual starting material was obtained as a white solid (30.8 mg, 36% RSM, 99% ee).

^{1}H NMR: (400 MHz, $CDCl_3$) δ 7.71 – 7.61 (m, 2H), 7.58 – 7.51 (m, 1H), 7.50 – 7.40 (m, 2H), 4.09 (qd, *J* = 7.1, 3.9 Hz, 2H), 3.81 – 3.44 (m, 2H), 3.24 – 2.99 (m, 2H), 1.14 (t, *J* = 7.1 Hz, 3H).

^{31}P NMR: (162 MHz, $CDCl_3$) δ 32.19.

RSM ^{1}H NMR: (400 MHz, $CDCl_3$) δ 8.15 – 6.85 (m, 1H), 7.59 – 7.39 (m, 5H), 7.31 – 7.20 (m, 3H), 7.06 (dt, *J* = 7.3, 2.3 Hz, 2H), 3.57 – 3.26 (m, 2H).

RSM ^{31}P NMR: (162 MHz, $CDCl_3$) δ 29.68.

Optical Rotation: $[\alpha]^{23}_{D}$ = – 19.7 (c = 1, MeOH).

RSM Optical Rotation: $[\alpha]^{23}_{D}$ = – 2.7 (c = 1, MeOH).

Reverse Phase HPLC-MS: Agilent Poroshell 120 EC-C18, 1.5 mL/min, [A] 0.1% AcOH in Water, [B] 0.1% AcOH in Acetonitrile, scan and single ion monitoring $[M+H]^+$, gradient: 0-0.2 min: 5-50% B; 0.2-0.4 min: 5–20% B; 0.4–2.5 min: 20–50% B; 2.5–2.6 min: 50–95% B; 2.6–3.0 min: 95% B; 3.0–3.1 min: 95–5% B; 3.1–3.5 min: 5% B.

Chiral HPLC: Chiralpak IA (Hex/EtOH = 91:09, 1 mL/min).

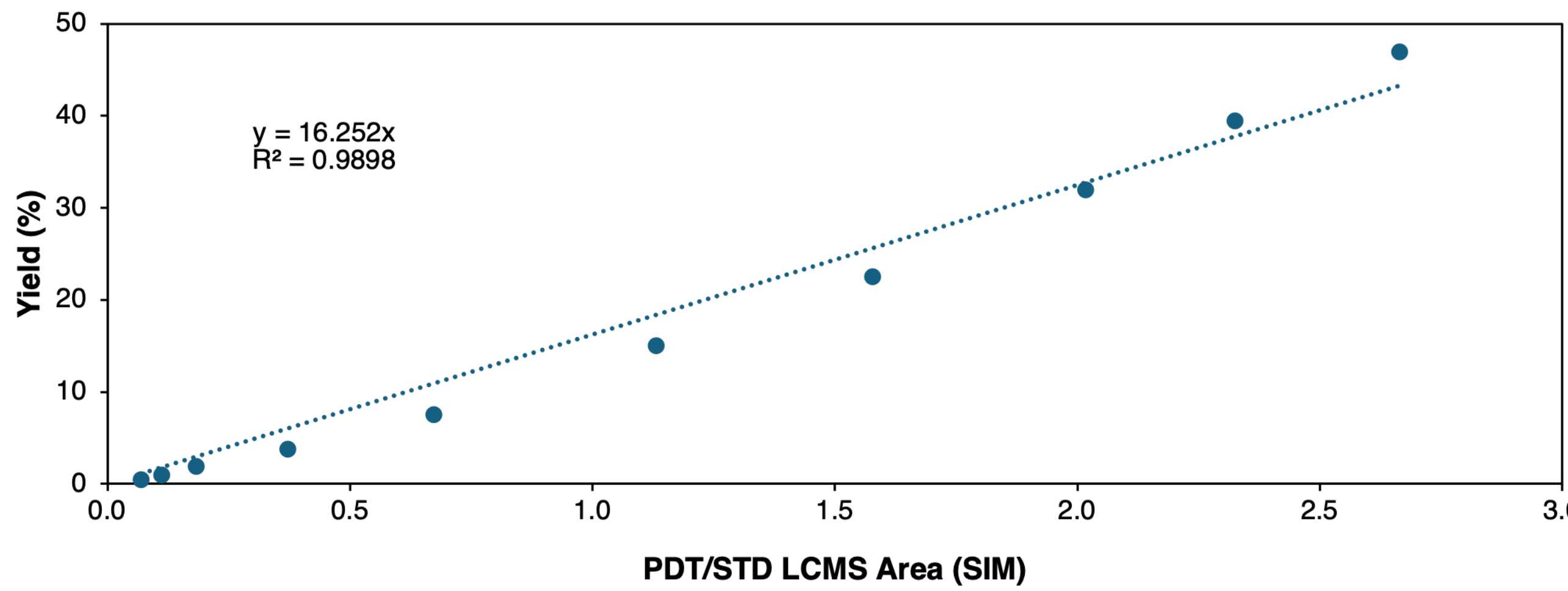

Figure S17: Calibration curve using authentic sample and papaverine as an external standard.

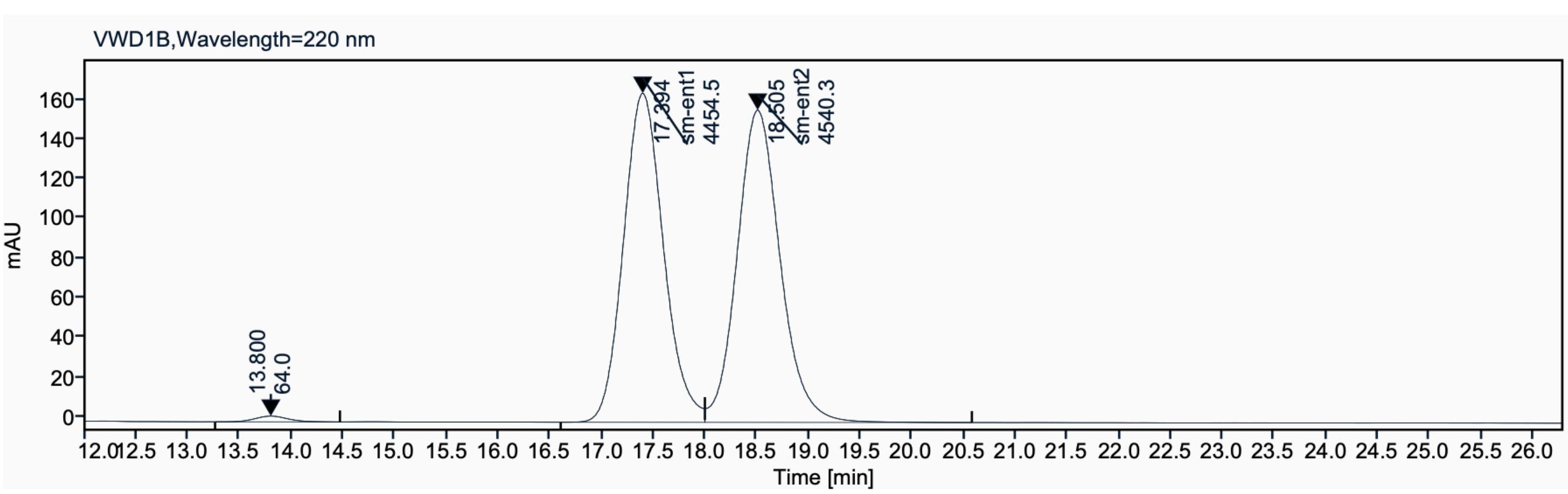

Name	RT [min]	Area
sm-ent1	17.394	4454.4797
sm-ent2	18.505	4540.2821

Figure S18: Chiral HPLC trace of racemic PhBnP(O)H.

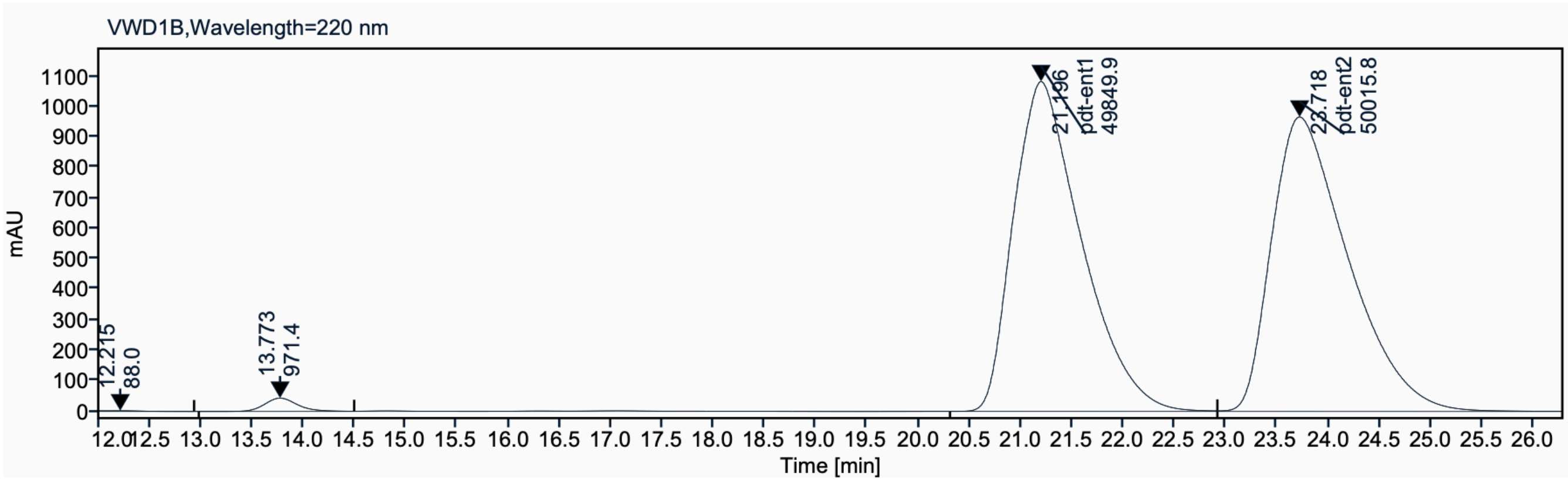

Name	RT [min]	Area
pdt-ent1	21.196	49849.9027
pdt-ent2	23.718	50015.7898

Figure S19: Chiral HPLC trace of racemic PhBnP(O)–CH_2CO_2Et.

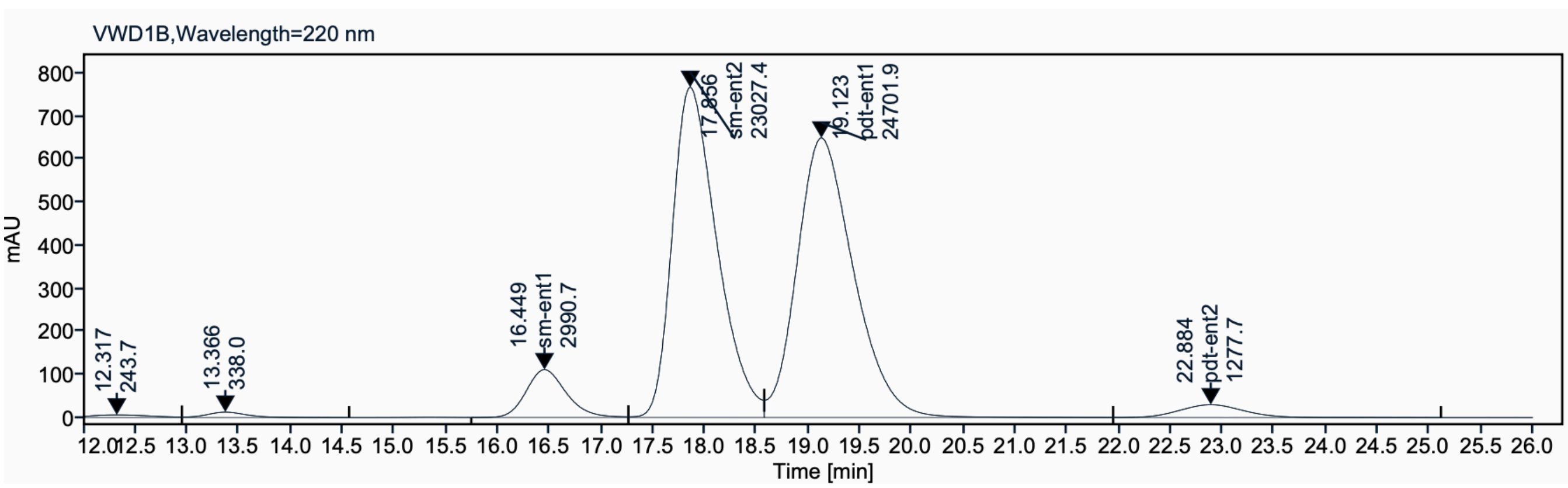

Name	RT [min]	Area
sm-ent1	16.449	2990.6759
sm-ent2	17.856	23027.3923
pdt-ent1	19.123	24701.8683
pdt-ent2	22.884	1277.6627

Figure S20: Chiral HPLC trace of reaction catalyzed by G4-I93T, PhBnP(O)H and EDA.

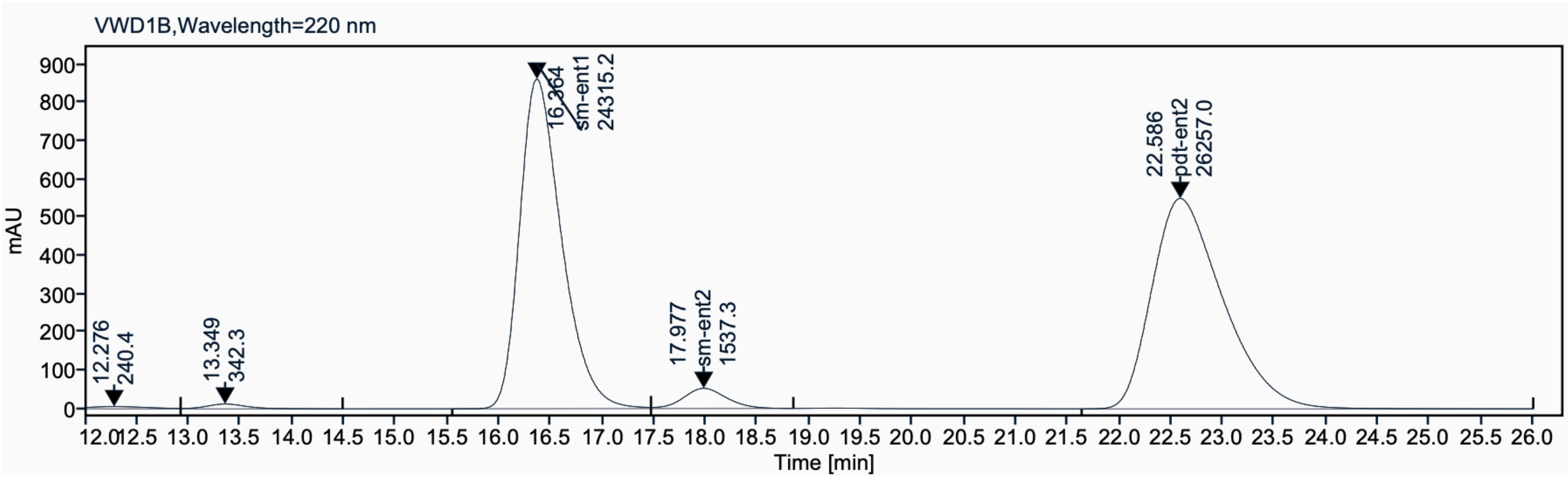

Name	RT [min]	Area
sm-ent1	16.364	24315.2462
sm-ent2	17.977	1537.3051
pdt-ent2	22.586	26257.0087

Figure S21: Chiral HPLC trace of reaction catalyzed by G6.1, PhBnP(O)H and EDA.

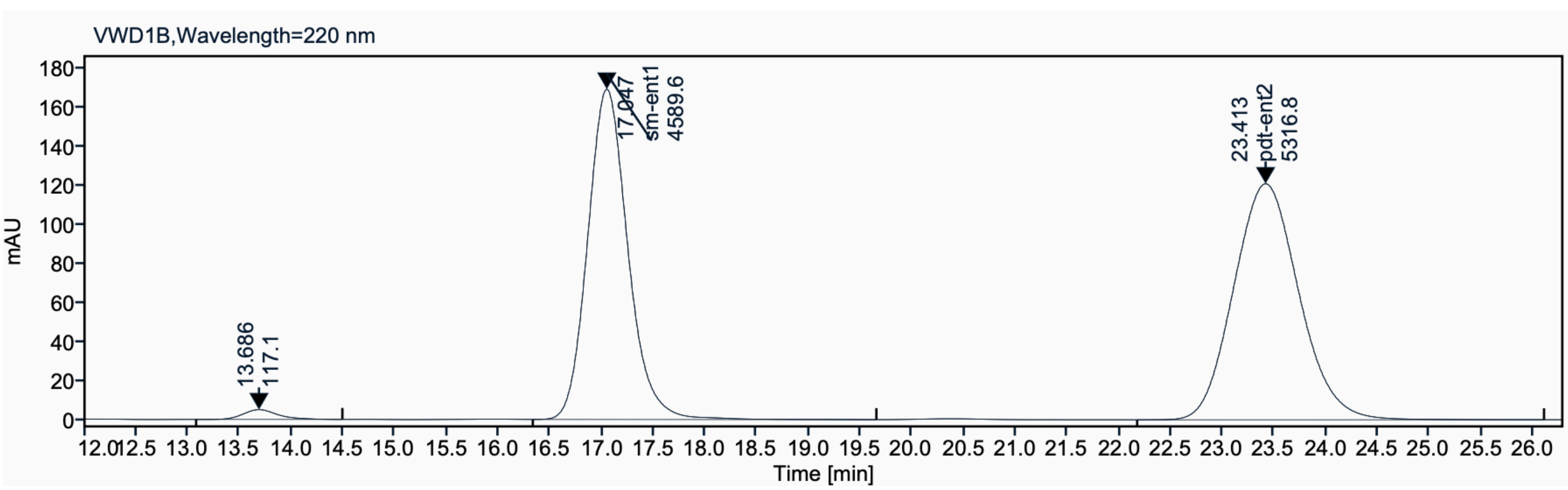

Name	RT [min]	Area
sm-ent1	17.047	4589.6159
pdt-ent2	23.413	5316.8484

Figure S22: Chiral HPLC trace of large-scale reaction catalyzed by G6.1, PhBnP(O)H and EDA.

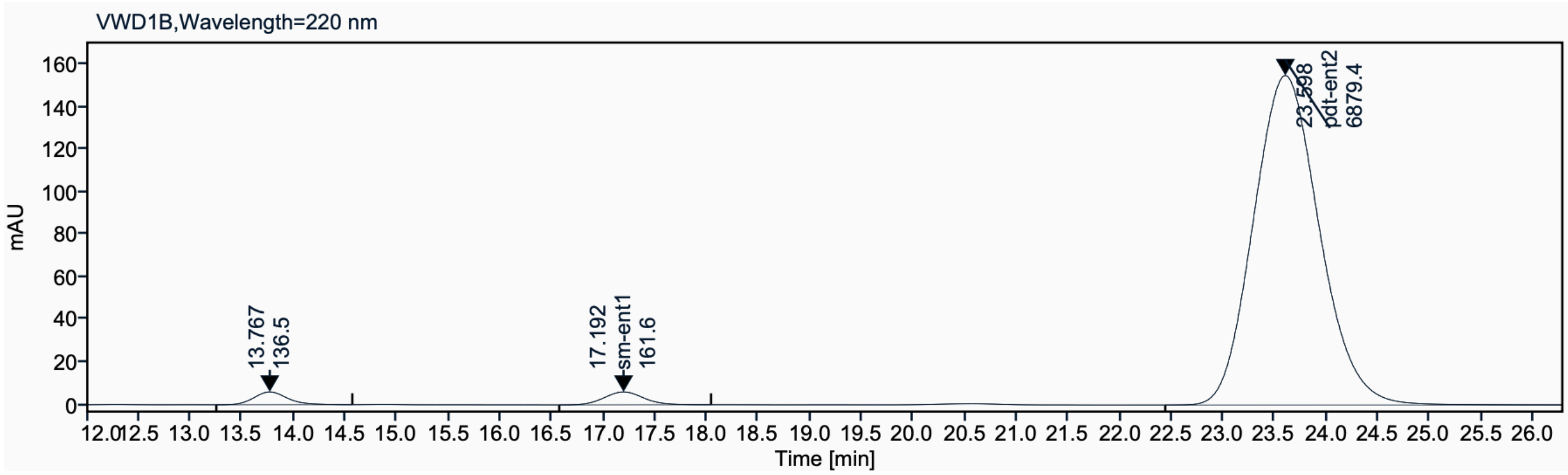

Name	RT [min]	Area
sm-ent1	17.192	161.6397
pdt-ent2	23.598	6879.4260

Figure S23: Chiral HPLC trace of purified product from large scale reaction catalyzed by G6.1, PhBnP(O)H and EDA.

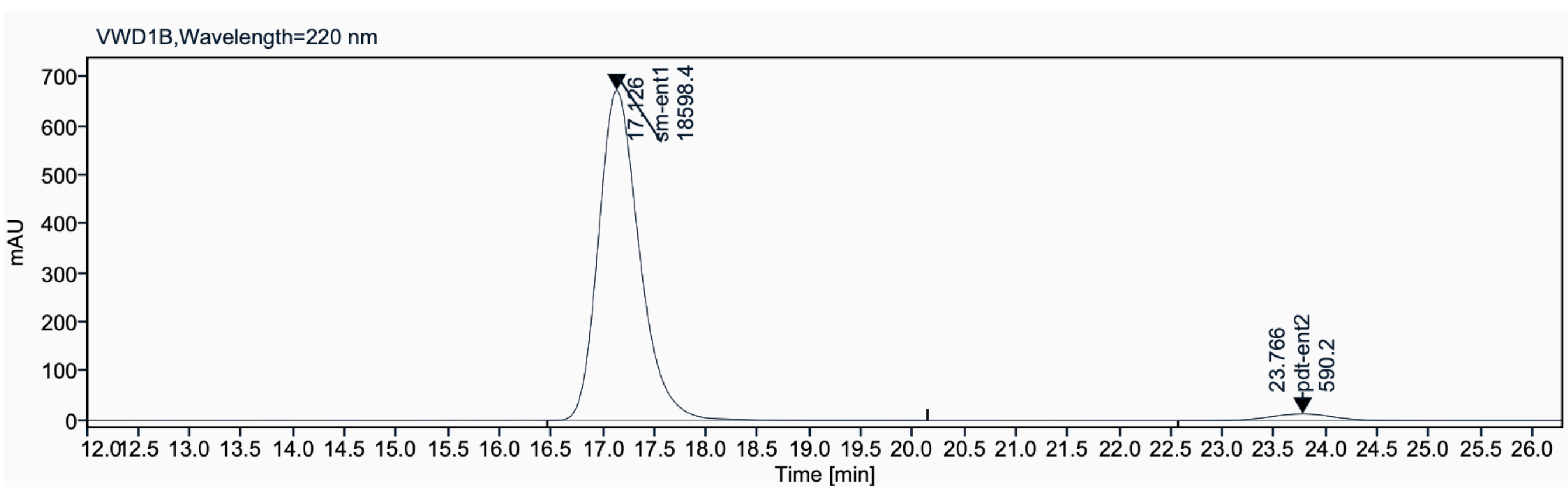

Name	RT [min]	Area
sm-ent1	17.126	18598.3620
pdt-ent2	23.766	590.2334

Figure S24: Chiral HPLC trace of purified RSM from large scale reaction catalyzed by G6.1, PhBnP(O)H and EDA.

7.3 Ph^{i}PrP(O)–CH_2CO_2Et (4b)

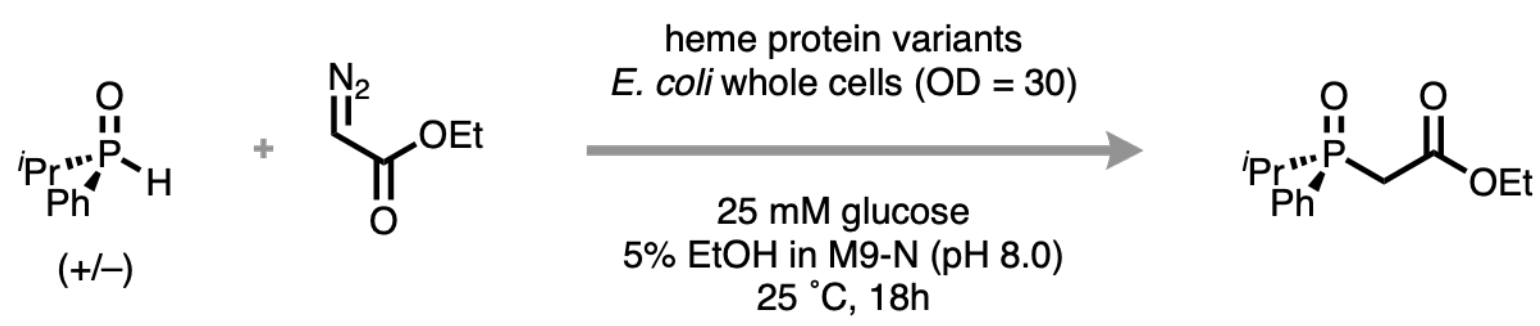

Ph^{i}PrP(O)H and EDA was screened against the Master Compilation Plate (P11) and four variants were chosen with which to perform validation with: G5OEt, G5OEt-A59T, G5OEt-L86Q, and G11.1. Absolute configuration of product assigned based on comparison to the reactivity of selected variants with other secondary phosphine oxide substrates.

Reactions were performed according to the validation procedure (three technical replicates for assay yield; two technical replicates for enantioselectivity).

Table S38: Yield and enantioselectivity of variants and selected reaction conditions for the reactions of Ph^{i}PrP(O)H and EDA.

Variant/conditions	Avg. Yield (%)	Std Dev Yield (%)	Avg. E.E. (%)	Std Dev E.E. (%)
G5OEt	52	2	-94	< 1
G5OEt-A59T	55	5	-82	–
G5OEt-L86Q	53	1	-86	< 1
G11.1	7	1	100	< 1

Large Scale (0.4 mmol): The standard validation procedure was followed using a larger scale, except the reaction was performed in a 40-mL vial. A 40-mL vial was charged with normalized cell suspension (G5OEt, 18 mL, OD_{600} = 30, M9-N, pH 8.0) and glucose (1 mL, 500 mM, $[glc]_{final}$ = 25 mM). The vial was brought into the anaerobic COY chamber, and a solution of Ph^{i}PrP(O)H and EDA in EtOH (1 mL, 400 Ph^{i}PrP(O)H, 300 mM EDA, $[P]_{final}$ = 20 mM, $[EDA]_{final}$ = 15 mM) was added. The vial was sealed with a PTFE lined cap and shaken at room temperature for 24 h. The reaction was quenched with IPA (2 mL) and a 400-µL aliquot was removed for chiral HPLC analysis (>99% ee). The reaction was extracted with EtOAc (4x 20 mL) – centrifugation at 5000xg for 1 minute was used to aid layer formation. The combined organics were dried with Na_2SO_4 and solvent removed *in vacuo*. The reaction outcome was analyzed by adding nitrobenzene as an analytical external standard (20 µL), and an aliquot was removed to determine the reaction yield (^{1}H NMR Yield, 51%).

Column: The resulting crude residue was purified by silica gel chromatography (10g SiliaFlash R60 SiO_2, packed w/ EtOAc). The crude residue was loaded in a minimal amount of DCM (200 µL x 3), and the product was eluted with a gradient (0–30% MeOH in EtOAc over 15 CV). The fractions with product were concentrated in vacuo (30 ˚C). The product was obtained as a clear oil (46.3 mg, 46%, >99% ee).

^{1}H NMR: (400 MHz, $CDCl_3$) δ 7.79 – 7.70 (m, 2H), 7.62 – 7.54 (m, 1H), 7.54 – 7.46 (m, 2H), 4.25 – 3.95 (m, 2H), 3.60 – 2.99 (m, 2H), 2.46 (h, J = 7.2 Hz, 1H), 1.36 (dd, J = 17.0, 7.1 Hz, 3H), 1.16 – 1.04 (m, 6H).

^{31}P NMR: (162 MHz, $CDCl_3$) δ 44.69.

Optical Rotation: $[\alpha]^{23}_{D}$ = – 18.0 (c = 1, MeOH).

Reverse Phase HPLC-MS: Agilent Poroshell 120 EC-C18, 1.5 mL/min, [A] 0.1% AcOH in Water, [B] 0.1% AcOH in Acetonitrile, scan and single ion monitoring $[M+H]^+$, gradient: 0-0.2 min: 5-50% B; 0.2-0.4 min: 5–20% B; 0.4–2.5 min: 20–50% B; 2.5–2.6 min: 50–95% B; 2.6–3.0 min: 95% B; 3.0–3.1 min: 95–5% B; 3.1–3.5 min: 5% B.

Chiral HPLC: Chiralpak IA (Hex/EtOH = 94:06, 1 mL/min).

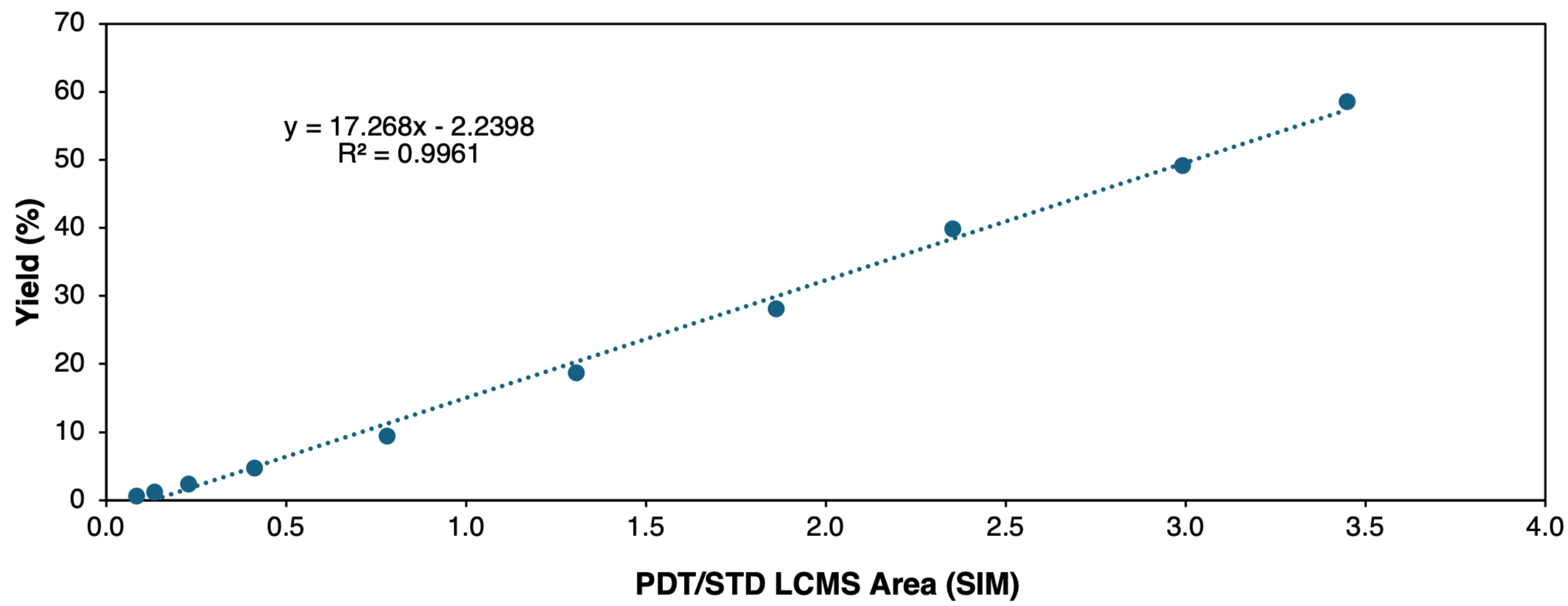

Figure S25: Calibration curve using authentic sample and papaverine as an external standard.

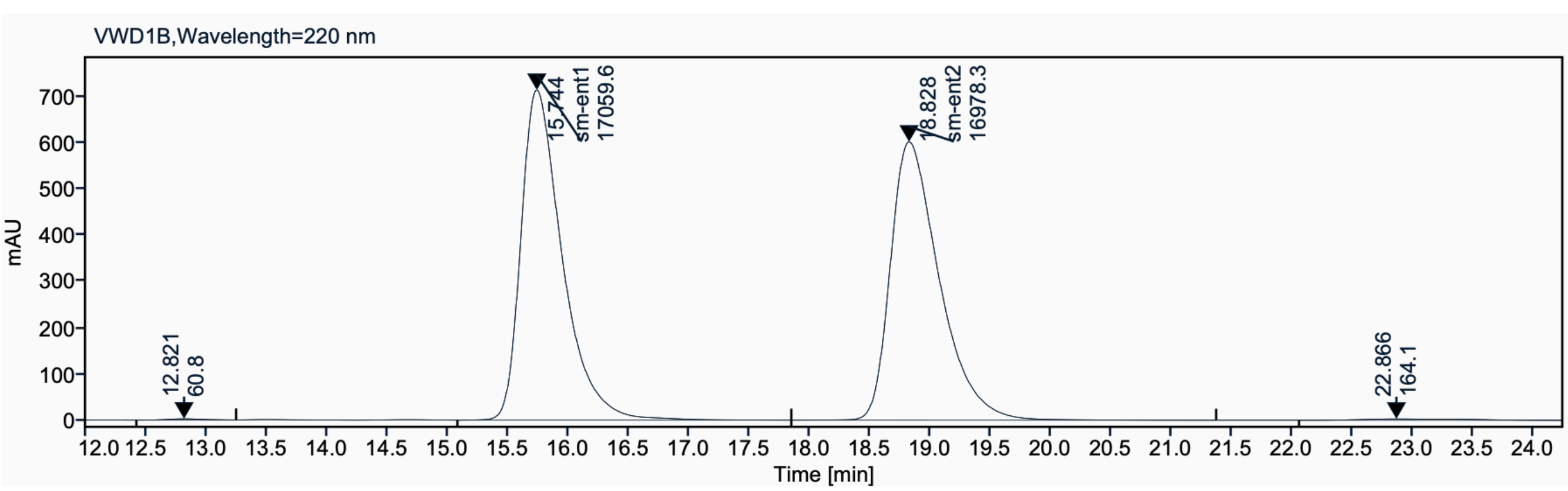

Name	RT [min]	Area
sm-ent1	15.744	17059.6457
sm-ent2	18.828	16978.2843

Figure S26: Chiral HPLC trace of racemic Ph^{i}PrP(O)H.

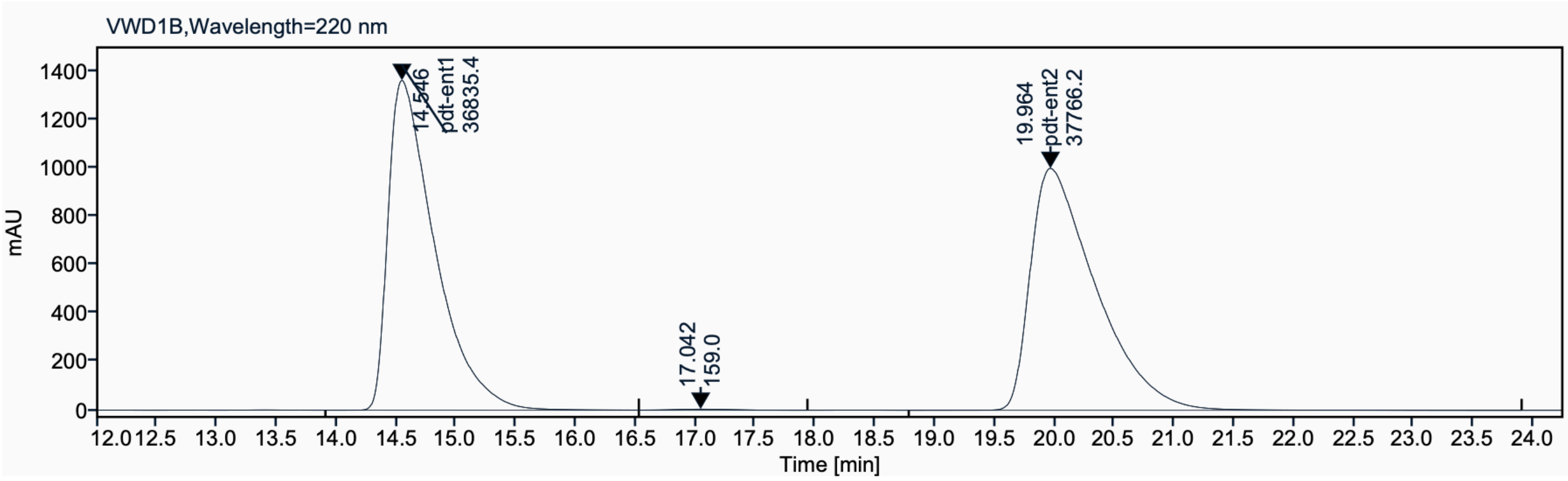

Name	RT [min]	Area
pdt-ent1	14.546	36835.3526
pdt-ent2	19.964	37766.2174

Figure S27: Chiral HPLC trace of racemic Ph^{i}PrP(O)–CH_2CO_2Et.

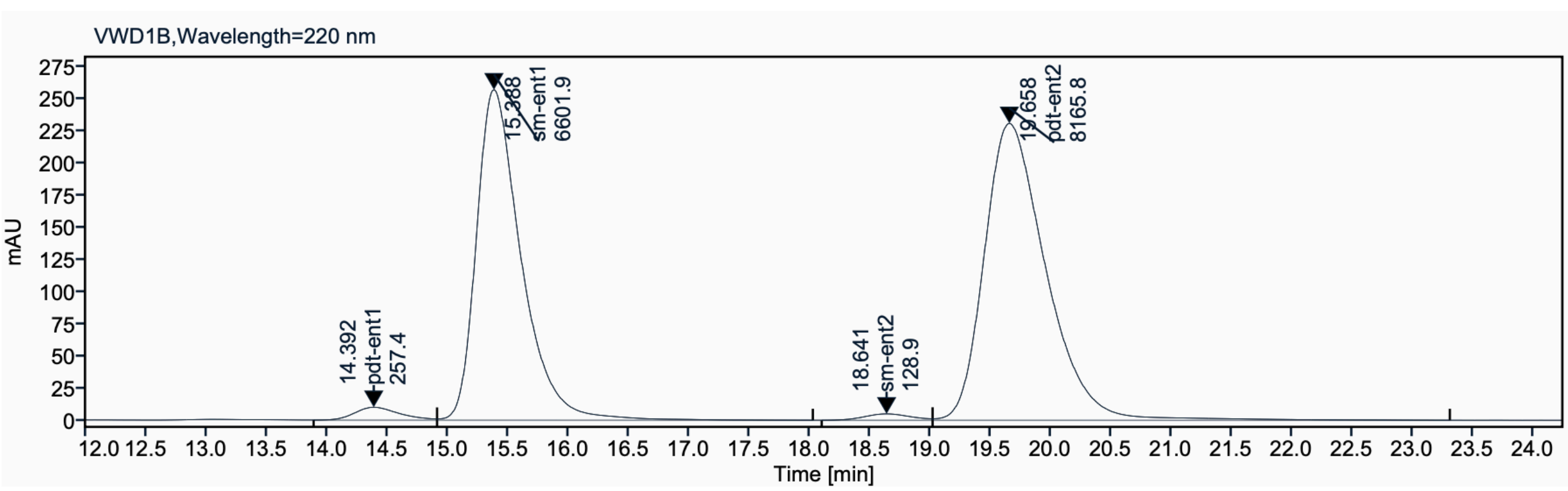

Name	RT [min]	Area
pdt-ent1	14.392	257.4024
sm-ent1	15.388	6601.9462
sm-ent2	18.641	128.9382
pdt-ent2	19.658	8165.8272

Figure S28: Chiral HPLC trace of reaction catalyzed by G5OEt, Ph^{i}PrP(O)H and EDA.

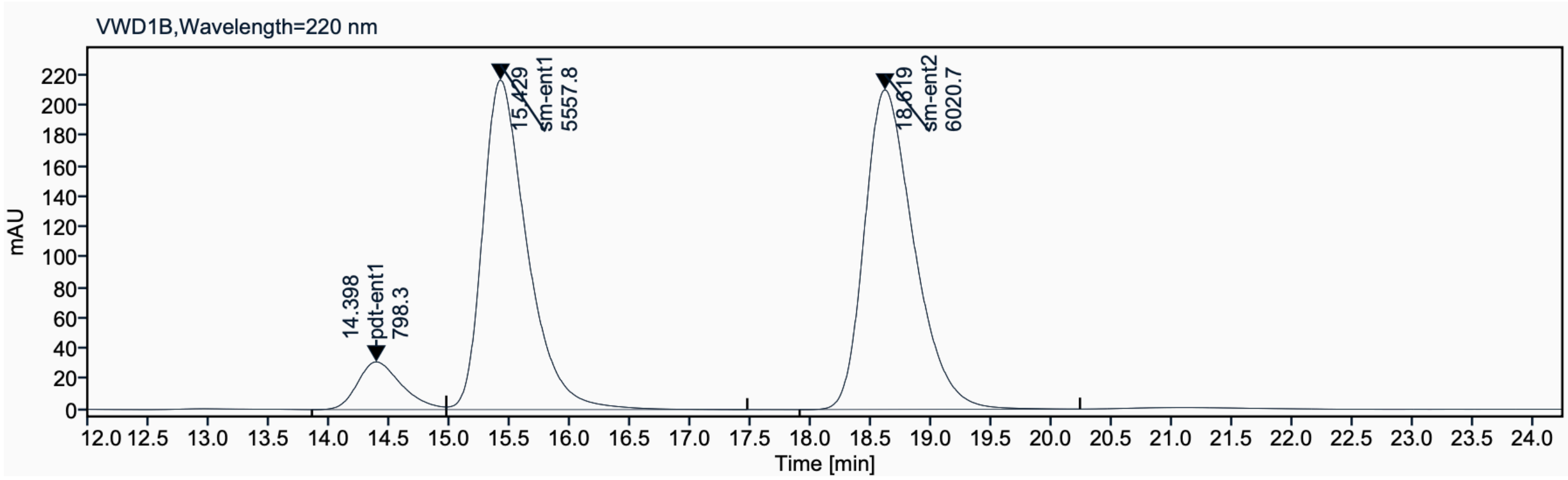

Name	RT [min]	Area
pdt-ent1	14.398	798.2771
sm-ent1	15.429	5557.7978
sm-ent2	18.619	6020.6582

Figure S29: Chiral HPLC trace of reaction catalyzed by G11.1, Ph^{i}PrP(O)H and EDA.

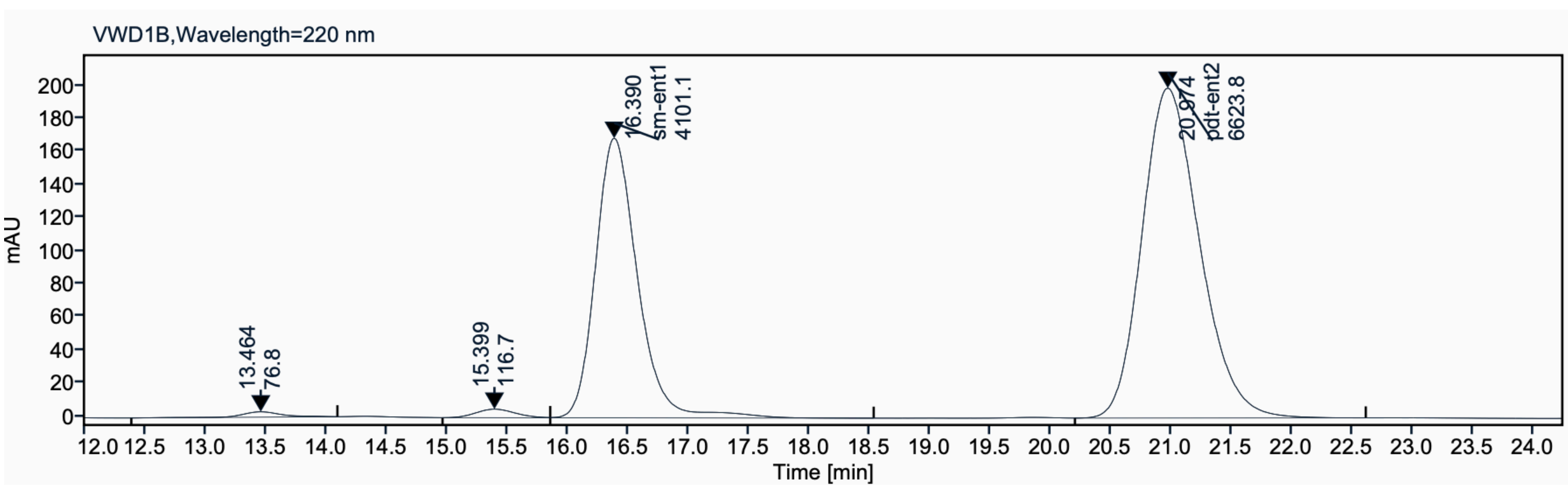

Name	RT [min]	Area
sm-ent1	16.390	4101.0584
pdt-ent2	20.974	6623.7502

Figure S30: Chiral HPLC trace of large-scale reaction catalyzed by G5OEt, Ph^{i}PrP(O)H and EDA.

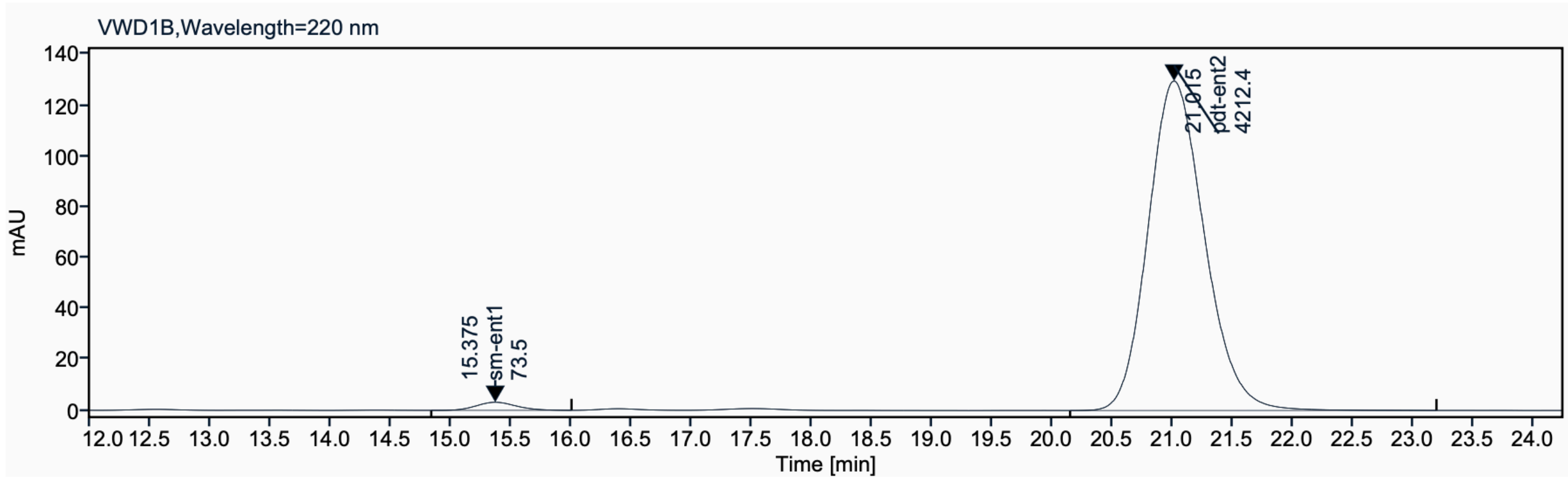

Name	RT [min]	Area
sm-ent1	15.375	73.5292
pdt-ent2	21.015	4212.4185

Figure S31: Chiral HPLC trace of purified product from large scale reaction catalyzed by G5OEt, Ph^{i}PrP(O)H and EDA.

7.4 PhCyP(O)–CH_2CO_2Et (5b)

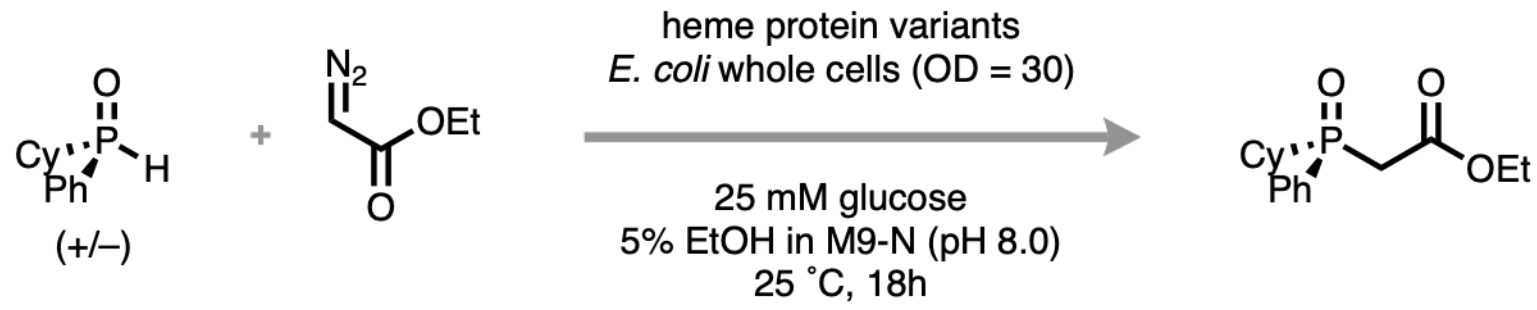

PhCyP(O)H and EDA was screened against the Master Compilation Plate (P11) and five variants were chosen with which to perform validation with: G5OEt, G5OEt-C93S, G11.1, G11.1-F149L, and G11.1-F149V. Absolute configuration of product assigned based on the assignment and consumption of substrate (Section 8.4).

Reactions were performed according to the validation procedure (three technical replicates for assay yield, one or two technical replicates for enantioselectivity). Due to lower than expected e.e. for the G5OEt variants, the reaction was repeated at 1 and 3 h timepoints using 2.75 mM EDA (0.55 equiv.).

Table S39: Yield and enantioselectivity of variants and selected reaction conditions for the reactions of PhCyP(O)H and EDA.

Variant/conditions	Avg. Yield (%)	Std Dev Yield (%)	Avg. E.E. (%)	Std Dev E.E. (%)
G5OEt	69	1	-44	< 1
G5OEt-1h	59	2	-48	–
G5OEt-3h	59	1	-43	< 1
G5OEt-C93S	65	2	-53	2
G5OEt-C93S-1h	52	1	-86	–
G5OEt-C93S-3h	55	0	-59	–
G11.1	19	4	100	< 1
G11.1-F149L	19	1	100	< 1
G11.1-F149V	45	1	100	< 1

Large Scale (0.4 mmol): The standard validation procedure was followed using a larger scale, except the reaction was performed in a 40-mL vial. A 40-mL vial was charged with normalized cell suspension normalized cell suspension (G11.1-F149V, 18 mL, OD_{600} = 30, M9-N, pH 8.0) and glucose (1 mL, 500 mM, $[glc]_{final}$ = 25 mM). The vial was brought into the anaerobic COY chamber and a solution of PhCyP(O)H and EDA in EtOH (1 mL, 400 PhCyP(O)H, 300 mM EDA, $[P]_{final}$ = 20 mM, $[EDA]_{final}$ = 15 mM) was added. The vial was sealed with a PTFE lined cap and shaken at room temperature for 24 h. The reaction was quenched with IPA (2 mL), and a 400-µL aliquot was removed for chiral HPLC analysis (>99% ee). The reaction was extracted with EtOAc (4x 20 mL) – centrifugation at 5000xg for 1 minute was used to aid layer formation. The combined organics were dried with Na_2SO_4 and solvent removed *in vacuo*. The reaction outcome was analyzed by adding nitrobenzene as an analytical external standard (20 µL), and an aliquot was removed to determine the reaction yield (^{1}H NMR Yield, 28%).

Column: The resulting crude residue was purified by silica gel chromatography (10g SiliaFlash R60 SiO_2, packed w/ EtOAc). The crude residue was loaded in a minimal amount of DCM (200 µL x 3), and the product was eluted with a gradient (0–5% MeOH in EtOAc over 15 CV). The fractions with product were concentrated in vacuo (30 ˚C). The product was obtained as a clear oil (27.4 mg, 23%, >99% ee).

^{1}H NMR: (400 MHz, $CDCl_3$) δ 7.72 (ddt, J = 11.1, 6.9, 1.5 Hz, 2H), 7.62 – 7.52 (m, 1H), 7.52 – 7.43 (m, 2H), 4.21 – 3.90 (m, 2H), 3.31 – 2.97 (m, 2H), 2.19 – 2.01 (m, 2H), 1.99 – 1.85 (m, 1H), 1.83 – 1.47 (m, 6H), 1.45 – 1.17 (m, 2H), 1.12 (t, J = 7.1 Hz, 3H).

^{31}P NMR: (162 MHz, $CDCl_3$) δ 38.39.

Optical Rotation: $[\alpha]^{23}_{D}$ = + 10.8 (c = 1, MeOH).

Reverse Phase HPLC-MS: Agilent Poroshell 120 EC-C18, 1.5 mL/min, [A] 0.1% AcOH in Water, [B] 0.1% AcOH in Acetonitrile, scan and single ion monitoring $[M+H]^+$, gradient: 0-0.2 min: 5-50% B; 0.2-0.4 min: 5–20% B; 0.4–2.5 min: 20–50% B; 2.5–2.6 min: 50–95% B; 2.6–3.0 min: 95% B; 3.0–3.1 min: 95–5% B; 3.1–3.5 min: 5% B.

Chiral HPLC: Chiralpak IA (Hex/EtOH = 92:08, 1 mL/min).

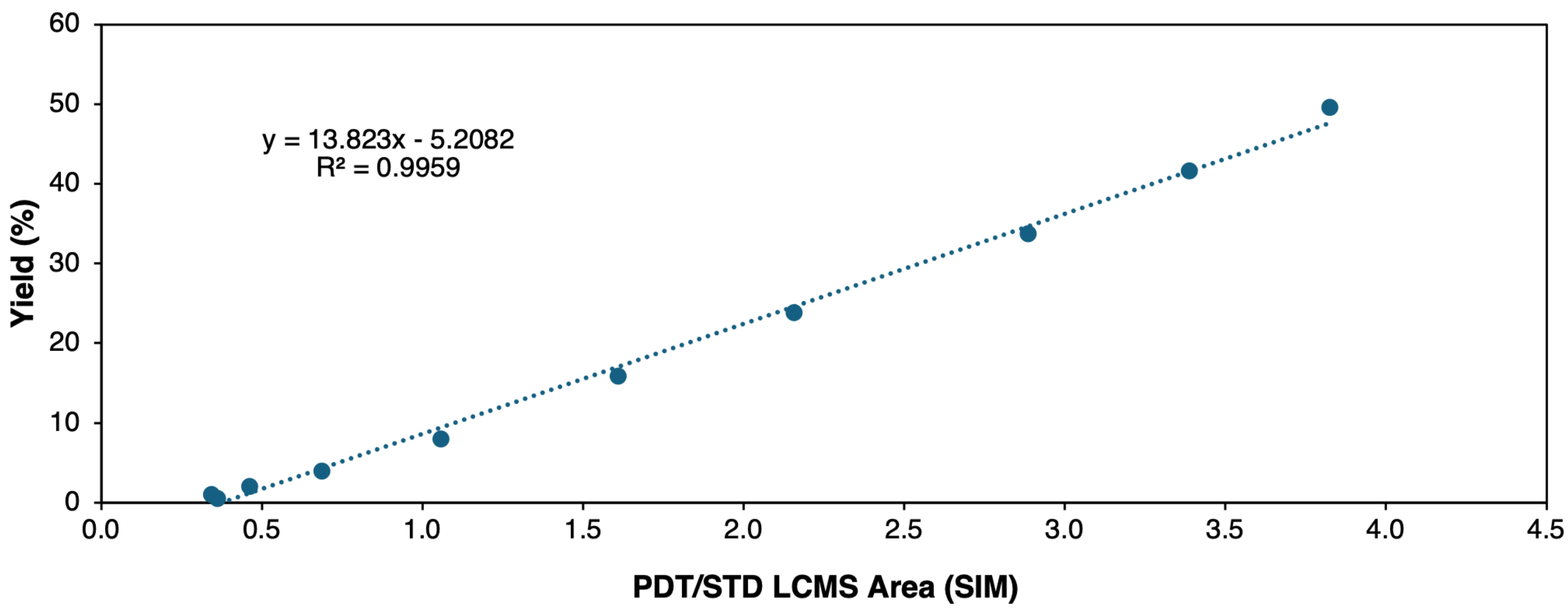

Figure S32: Calibration curve using authentic sample and papaverine as an external standard (for 18h time points).

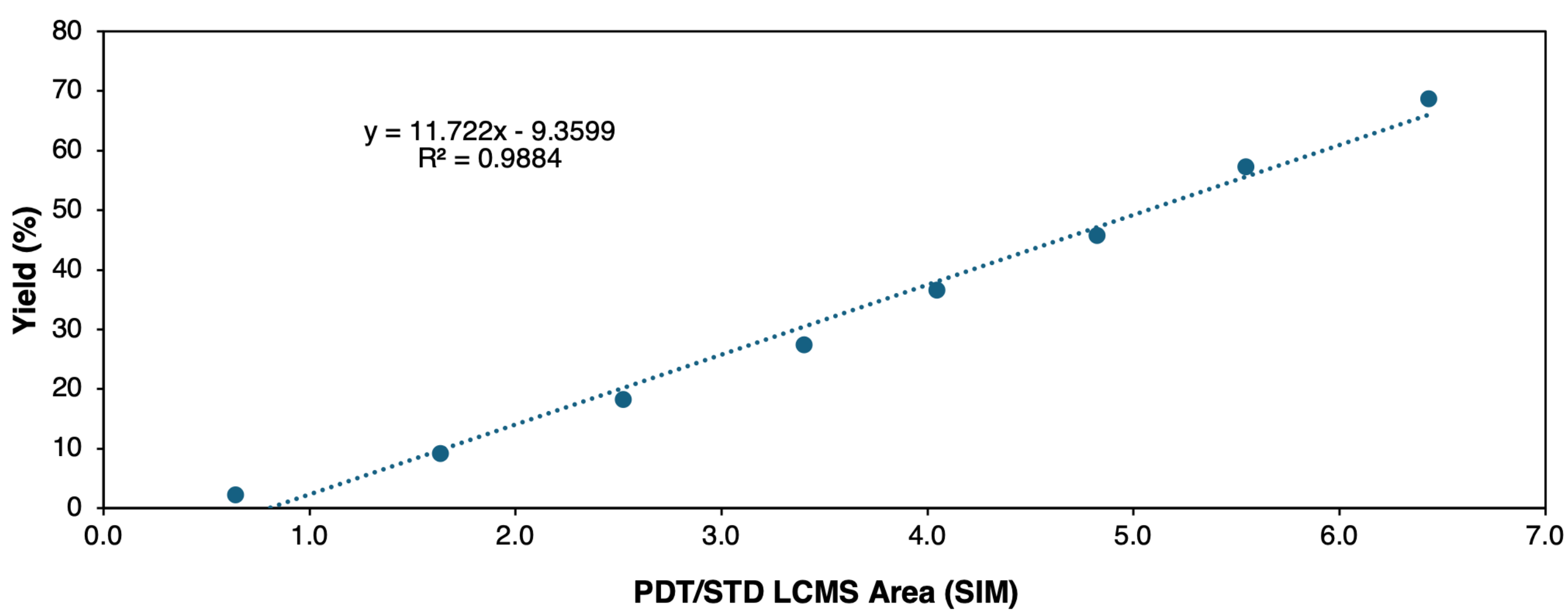

Figure S33: Calibration curve using authentic sample and papaverine as an external standard (for 1 and 3h time points).

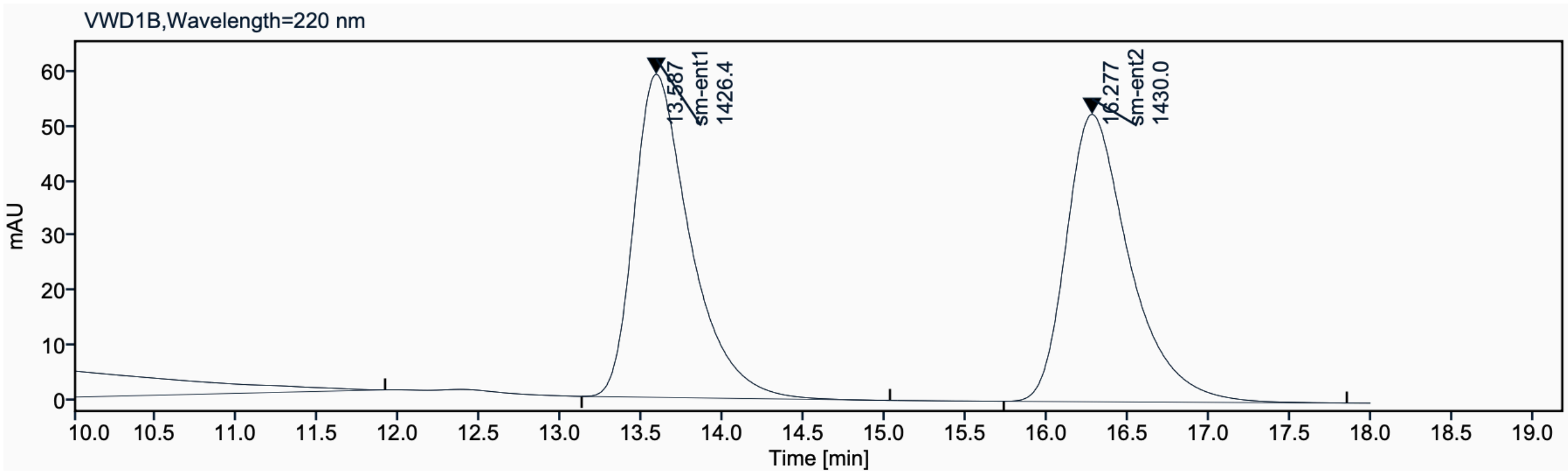

Name	RT [min]	Area
sm-ent1	13.587	1426.3500
sm-ent2	16.277	1430.0186

Figure S34: Chiral HPLC trace of racemic PhCyP(O)H.

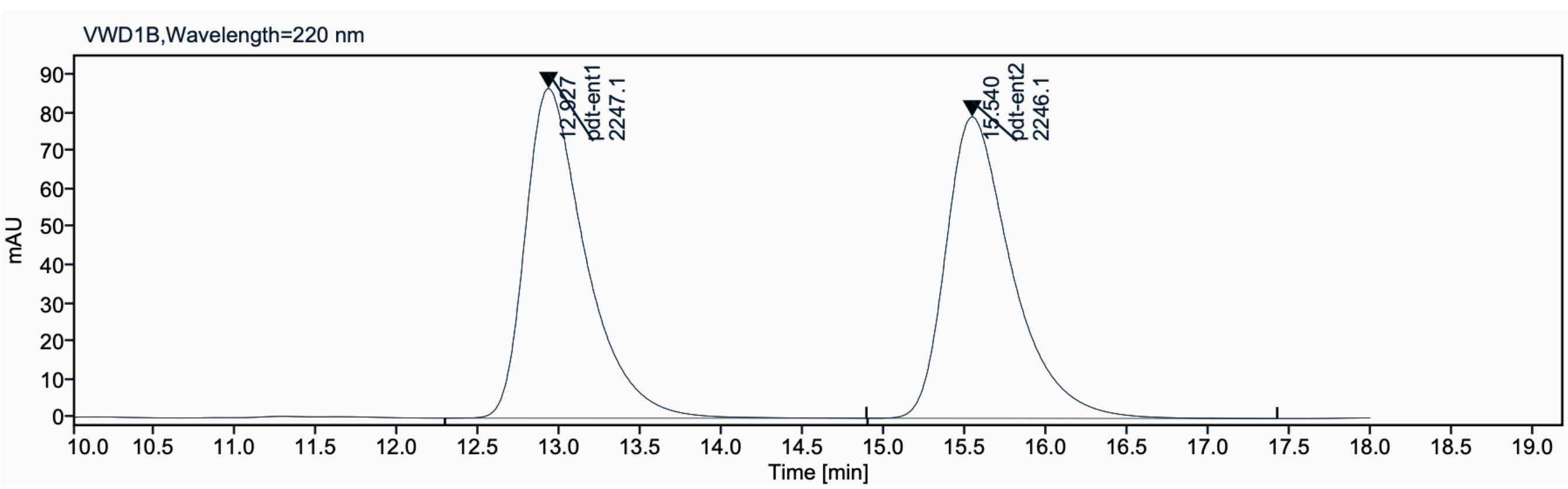

Name	RT [min]	Area
pdt-ent1	12.927	2247.1159
pdt-ent2	15.540	2246.0611

Figure S35: Chiral HPLC trace of racemic PhCyP(O)–CH_2CO_2Et.

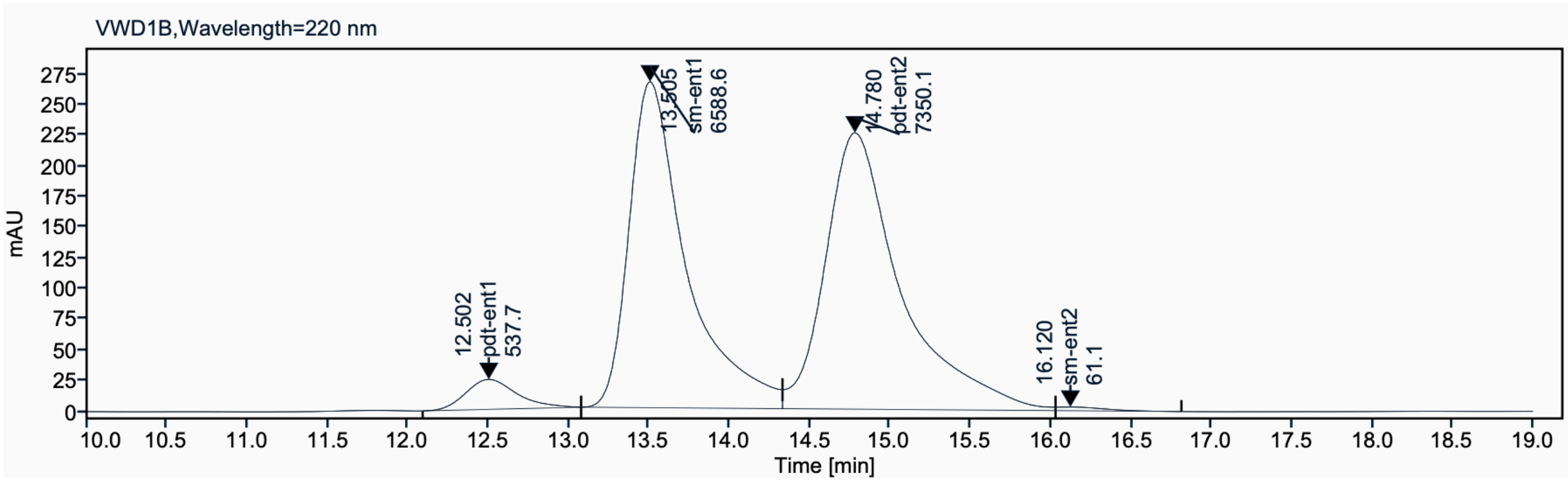

Name	RT [min]	Area
pdt-ent1	12.502	537.6707
sm-ent1	13.505	6588.5604
pdt-ent2	14.780	7350.0866
sm-ent2	16.120	61.0832

Figure S36: Chiral HPLC trace of reaction catalyzed by G5OEt-C93S at 1h, PhCyP(O)H and EDA.

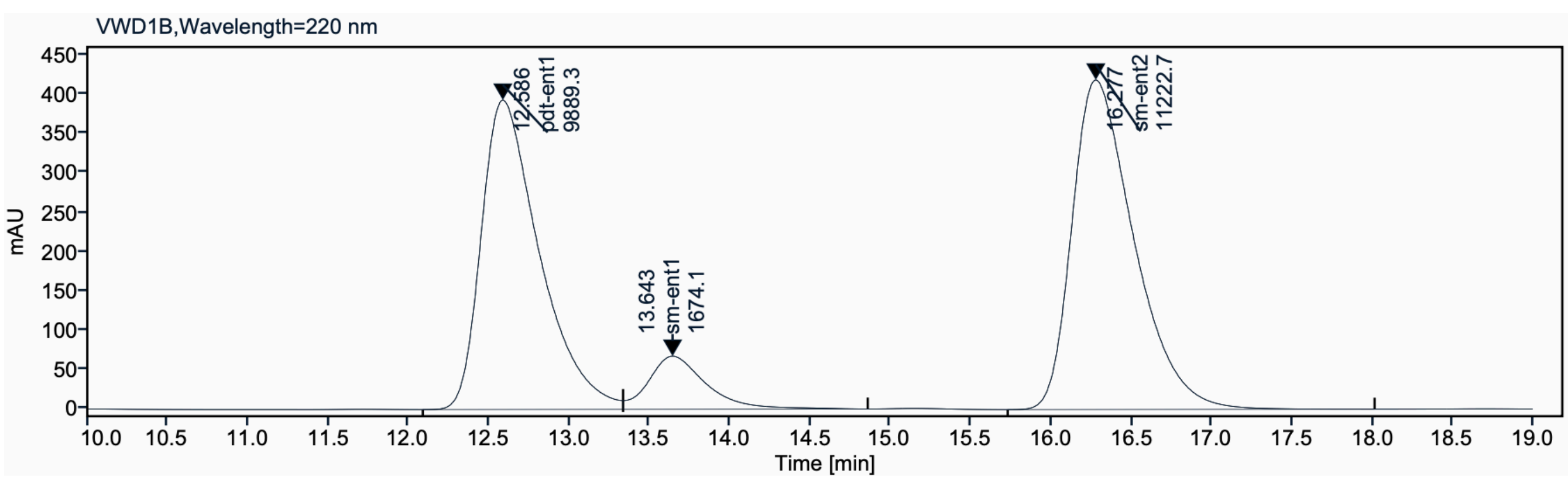

Name	RT [min]	Area
pdt-ent1	12.586	9889.2606
sm-ent1	13.643	1674.1360
sm-ent2	16.277	11222.7326

Figure S37: Chiral HPLC trace of reaction catalyzed by G11.1-F149V, PhCyP(O)H and EDA.

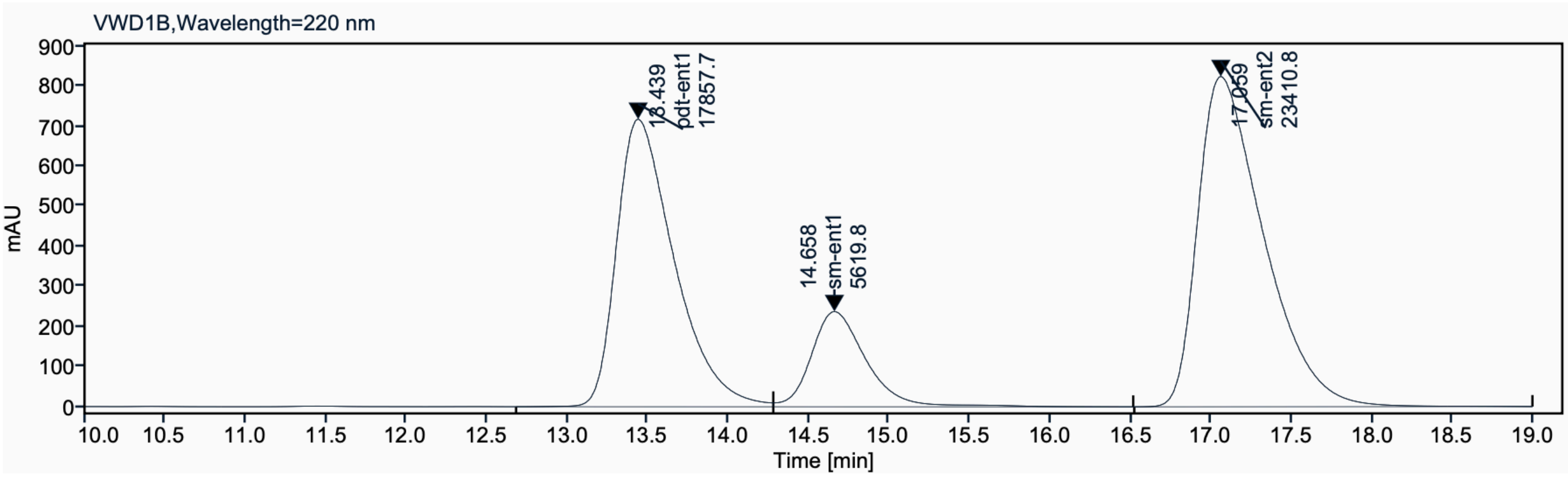

Name	RT [min]	Area
pdt-ent1	13.439	17857.6926
sm-ent1	14.658	5619.7805
sm-ent2	17.059	23410.8255

Figure S38: Chiral HPLC trace of large-scale reaction catalyzed by G11.1-F149V, PhCyP(O)H and EDA.

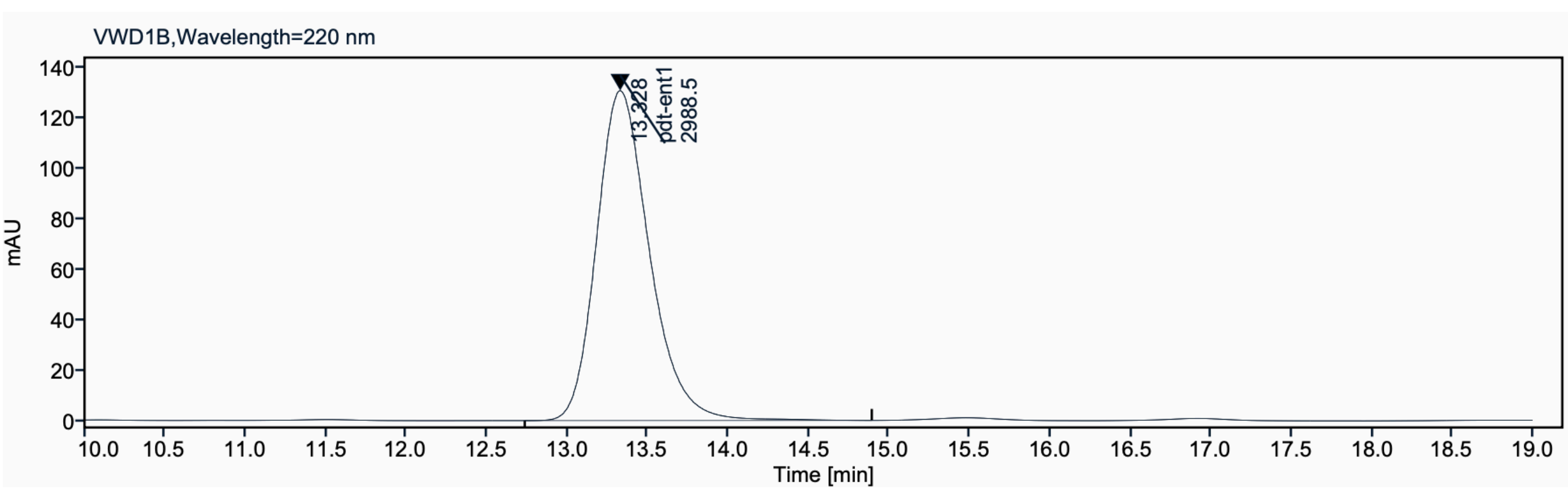

Name	RT [min]	Area
pdt-ent1	13.328	2988.4615

Figure S39: Chiral HPLC trace of purified product from large scale reaction catalyzed by G11.1-F149V, PhCyP(O)H and EDA.

7.5 Ph(4-FPh)P(O)–CH_2CO_2Et (6b)

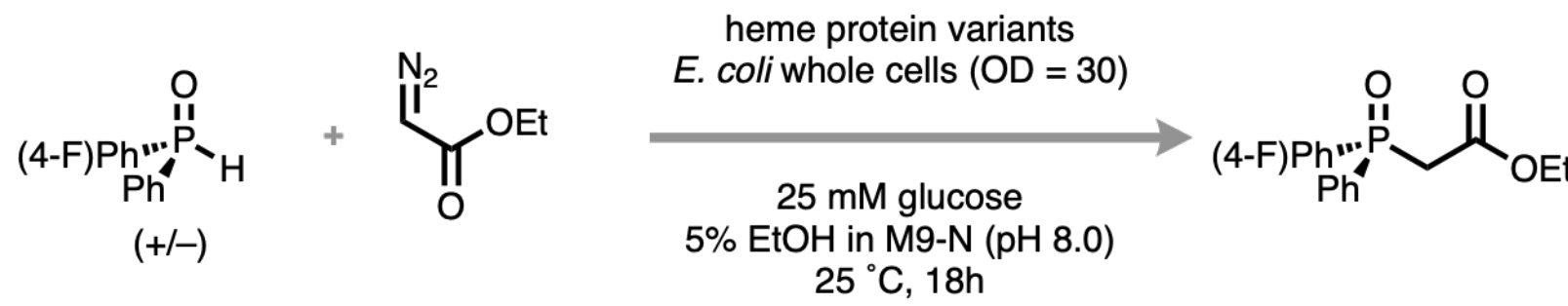

Ph(4-FPh)P(O)H and EDA was screened against the Master Compilation Plate (P11) and three variants were chosen with which to perform validation with: G5OEt-C93S, G11.1, and G11.1-M59L. Absolute configuration of product assigned based on comparison to the reactivity of selected variants with other secondary phosphine oxide substrates.

Reactions were performed according to the validation procedure (three technical replicates for assay yield; one technical replicate for enantioselectivity). Due to expected overprocessing resulting in diminished e.e., the reactions were also conducted at 1 h using 2.75 mM EDA (0.55 equiv.).

Table S40: Yield and enantioselectivity of variants and selected reaction conditions for the reactions of Ph(4-FPh)P(O)H and EDA.

Variant/conditions	Avg. Yield (%)	Std Dev Yield (%)	Avg. E.E. (%)
G11.1	38	2	-49
G11.1-1h	37	1	-48
G11.1-M59L	20	1	-60
G11.1-M59L-1h	21	1	-60
G5OEt-C93S	69	1	11
G5OEt-C93S-1h	49	1	31

Reverse Phase HPLC-MS: Agilent Poroshell 120 EC-C18, 1.5 mL/min, [A] 0.1% AcOH in Water, [B] 0.1% AcOH in Acetonitrile, scan and single ion monitoring $[M+H]^+$, gradient: 0-0.2 min: 5-50% B; 0.2-0.4 min: 5–20% B; 0.4–2.5 min: 20–50% B; 2.5–2.6 min: 50–95% B; 2.6–3.0 min: 95% B; 3.0–3.1 min: 95–5% B; 3.1–3.5 min: 5% B.

Chiral HPLC: Chiralpak IA (Hex/IPA = 75:25, 1 mL/min).

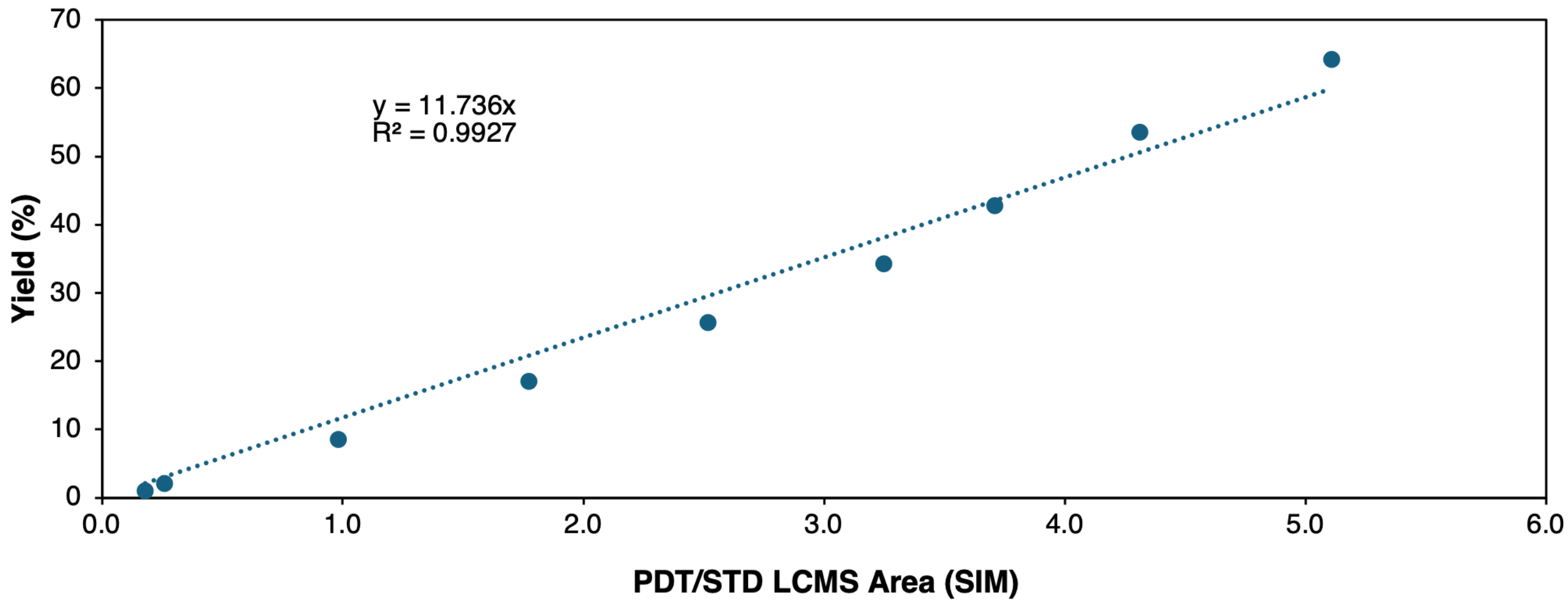

Figure S40: Calibration curve using authentic sample and papaverine as an external standard.

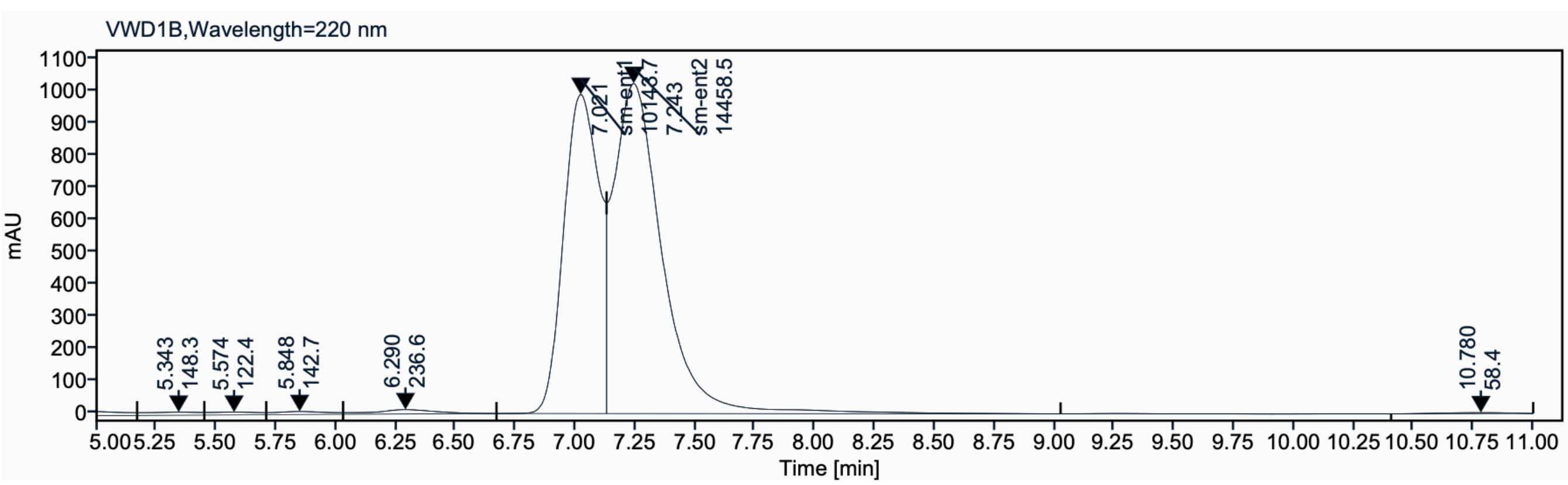

Name	RT [min]	Area
sm-ent1	7.021	10143.6732
sm-ent2	7.243	14458.5211

Figure S41: Chiral HPLC trace of racemic Ph(4-FPh)P(O)H.

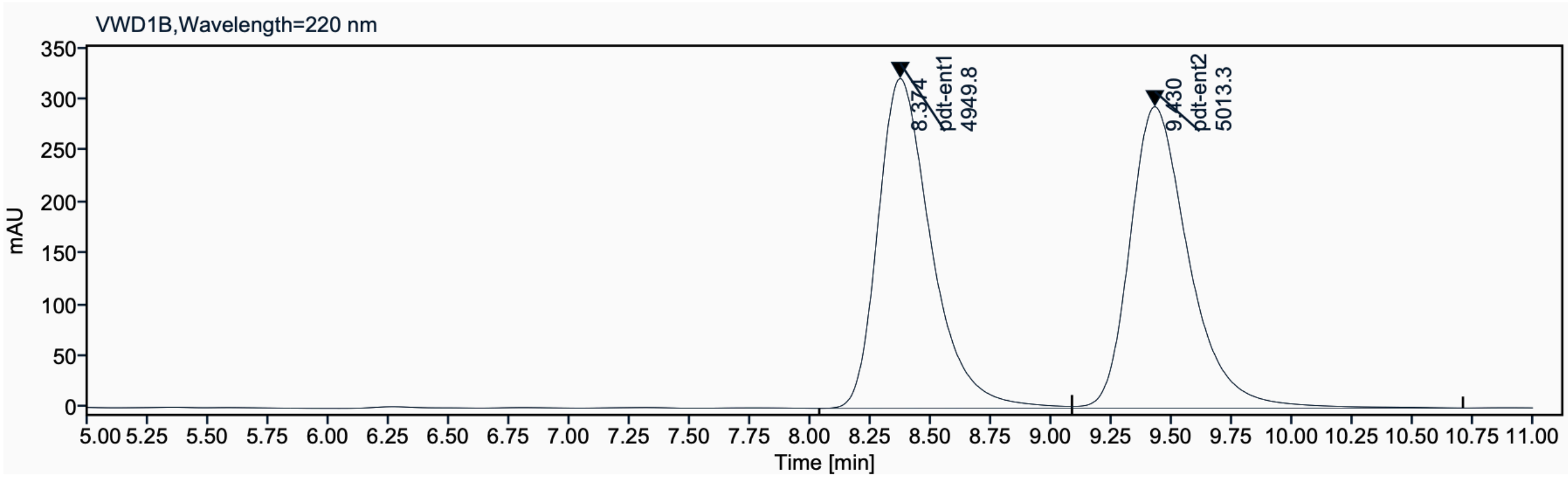

Name	RT [min]	Area
pdt-ent1	8.374	4949.8256
pdt-ent2	9.430	5013.2534

Figure S42: Chiral HPLC trace of racemic Ph(4-FPh)P(O)–CH_2CO_2Et.

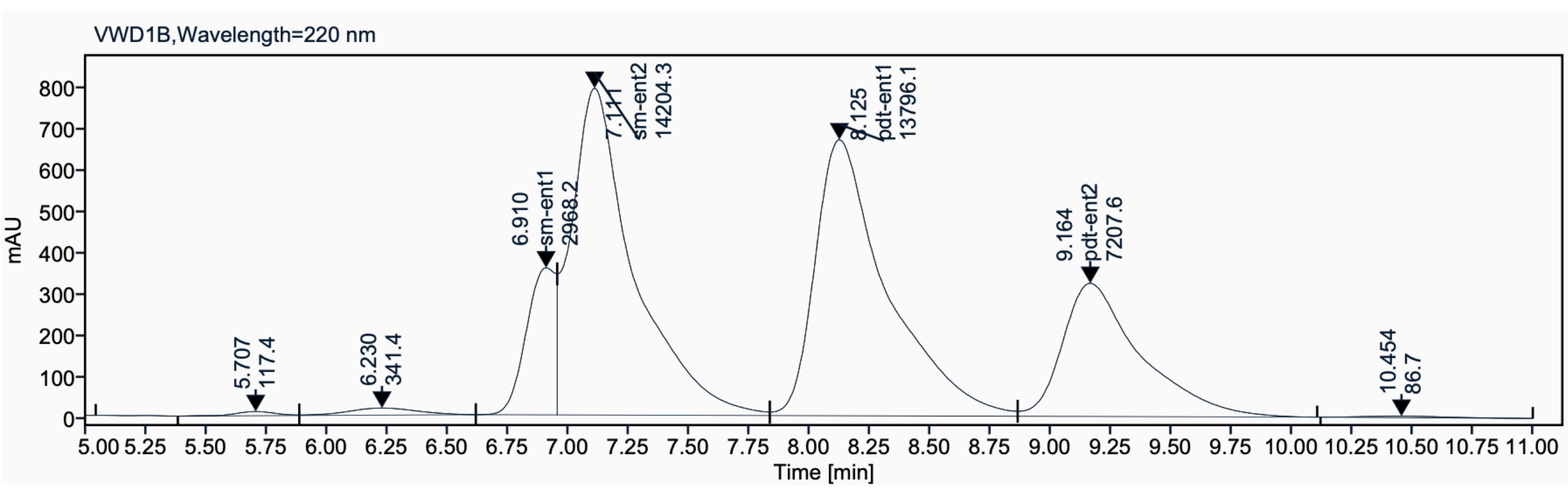

Name	RT [min]	Area
sm-ent1	6.910	2968.2083
sm-ent2	7.111	14204.3056
pdt-ent1	8.125	13796.0904
pdt-ent2	9.164	7207.6378

Figure S43: Chiral HPLC trace of reaction catalyzed by G5OEt-C93S at 1h, Ph(4-FPh)P(O)H and EDA.

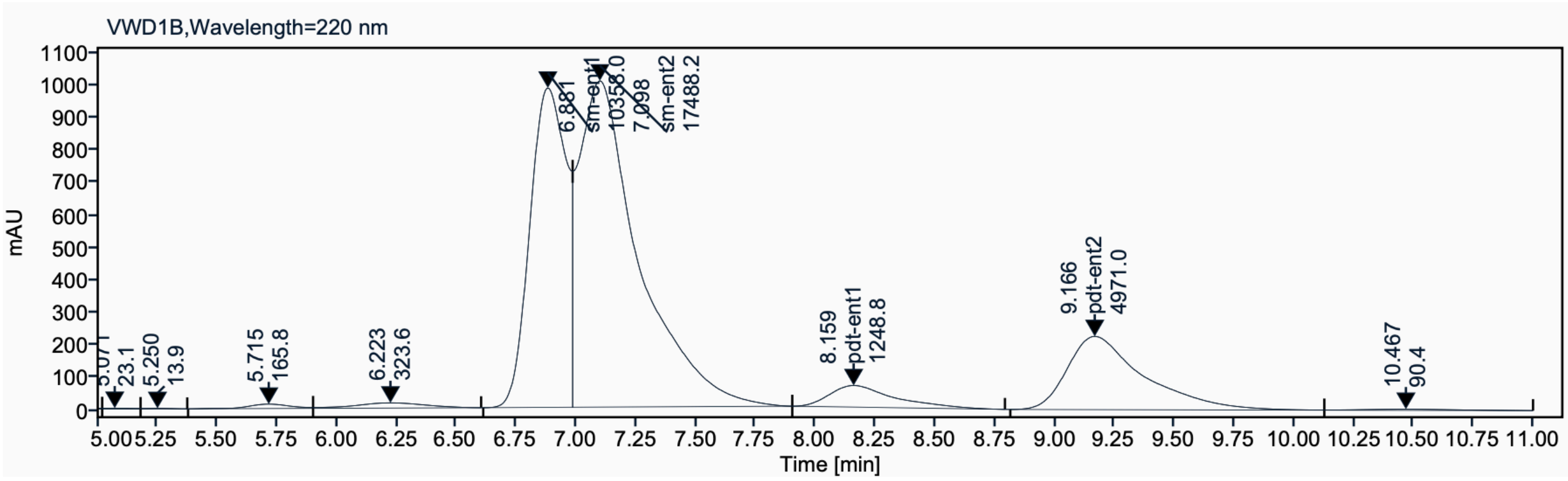

Name	RT [min]	Area
sm-ent1	6.881	10358.0447
sm-ent2	7.098	17488.2327
pdt-ent1	8.159	1248.7549
pdt-ent2	9.166	4970.9734

Figure S44: Chiral HPLC trace of reaction catalyzed by G11.1-M59L at 1h, Ph(4-FPh)P(O)H and EDA.

7.6 Ph^{o}AnP(O)–CH_2CO_2Et (7b)

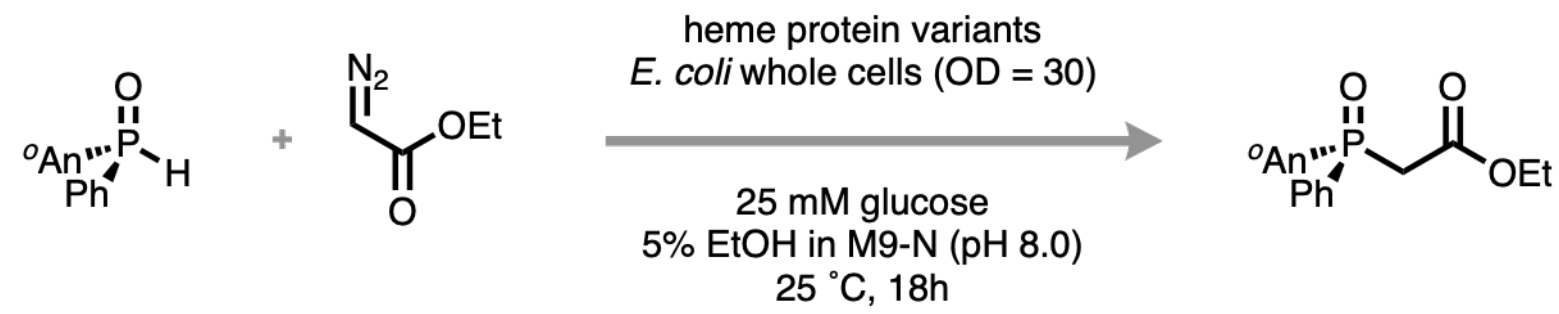

Ph(^{o}An)P(O)H and EDA was screened against the Master Compilation Plate (P11) and two variants were chosen with which to perform validation with: G4-I93S and G5OEt-C93S. Absolute configuration of product assigned based on the assignment and consumption of substrate (Section 8.4).

Reactions were performed according to the validation procedure (three technical replicates for assay yield, one technical replicate for enantioselectivity). Due to expected overprocessing resulting in diminished e.e., the reactions were also conducted at 1 h using 2.75 mM EDA (0.55 equiv.).

Table S41: Yield and enantioselectivity of variants and selected reaction conditions for the reactions of Ph(^{o}An)P(O)H and EDA.

Variant/conditions	Avg. Yield (%)	Std Dev Yield (%)	Avg. E.E. (%)
G4-I93S	14	2	71
G4-I93S-1h	9	4	63
G5OEt-C93S	40	1	60
G5OEt-C93S-1h	34	1	61

Reverse Phase HPLC-MS: Agilent Poroshell 120 EC-C18, 1.5 mL/min, [A] 0.1% AcOH in Water, [B] 0.1% AcOH in Acetonitrile, scan and single ion monitoring $[M+H]^+$, gradient: 0-0.2 min: 5-50% B; 0.2-0.4 min: 5–20% B; 0.4–2.5 min: 20–50% B; 2.5–2.6 min: 50–95% B; 2.6–3.0 min: 95% B; 3.0–3.1 min: 95–5% B; 3.1–3.5 min: 5% B.

Chiral HPLC: Chiralpak IC (Hex/IPA = 60:40, 1 mL/min).

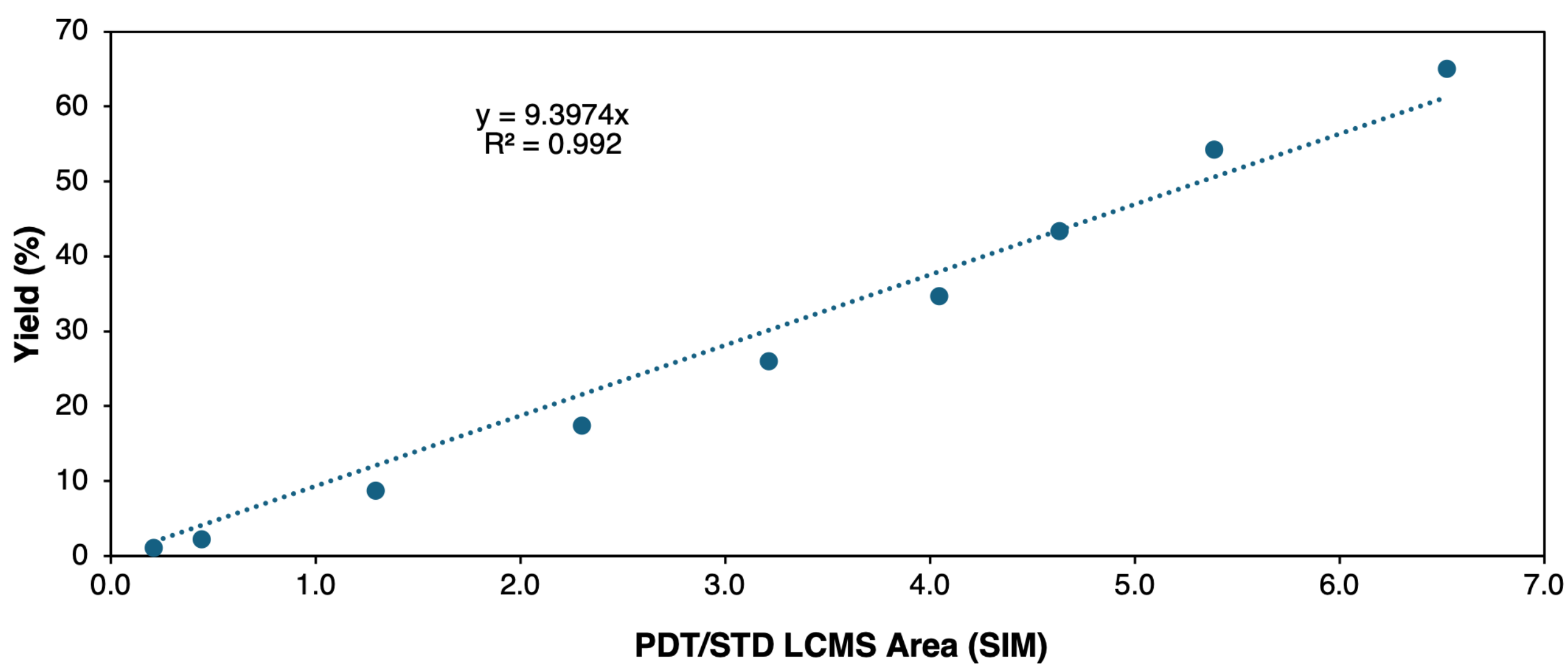

Figure S45: Calibration curve using authentic sample and papaverine as an external standard.

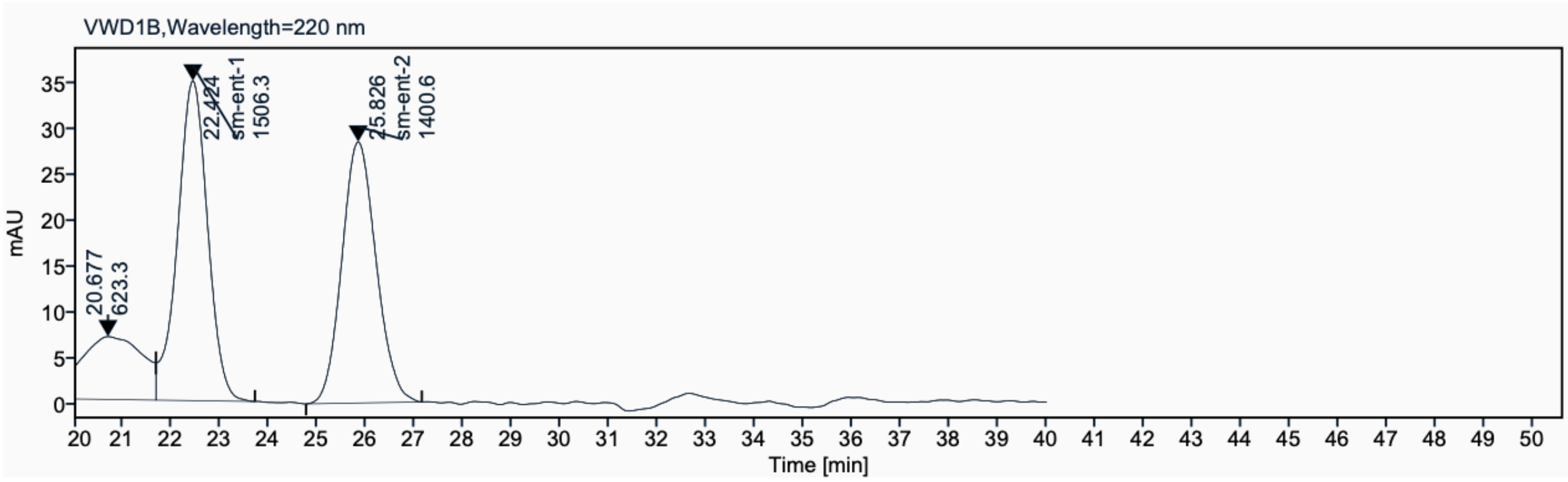

Name	RT [min]	Area
sm-ent-1	22.424	1506.3171
sm-ent-2	25.826	1400.6420

Figure S46: Chiral HPLC trace of racemic Ph(^{o}An)P(O)H.

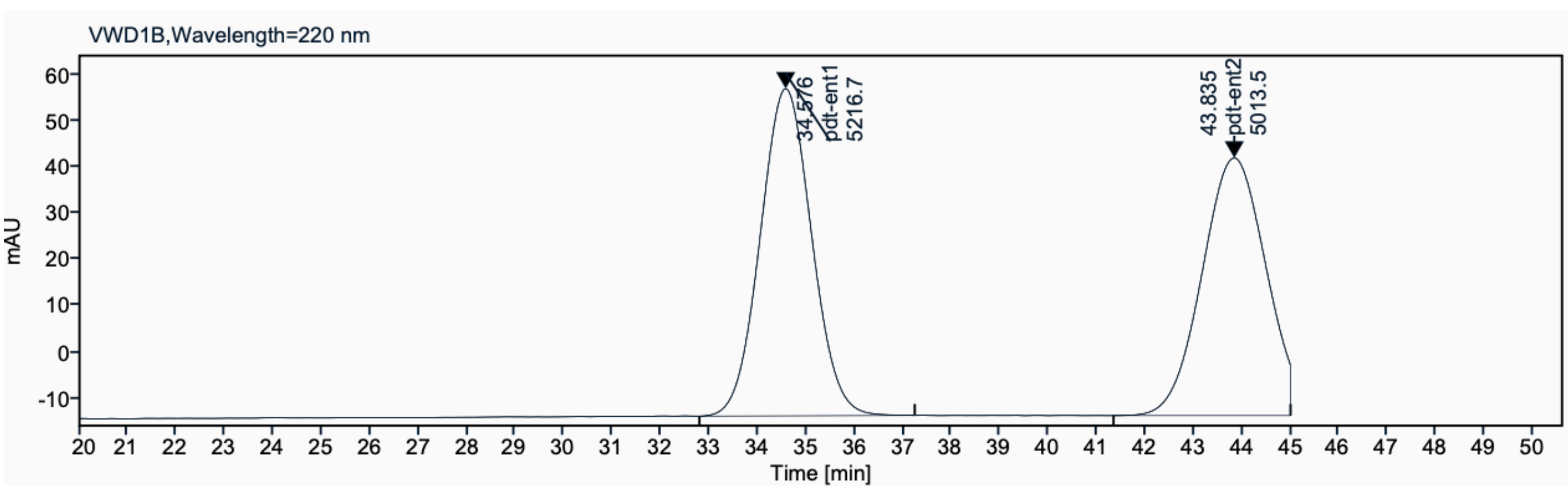

Name	RT [min]	Area
pdt-ent1	34.576	5216.7226
pdt-ent2	43.835	5013.4550

Figure S47: Chiral HPLC trace of racemic Ph(^{o}An)P(O)–CH_2CO_2Et.

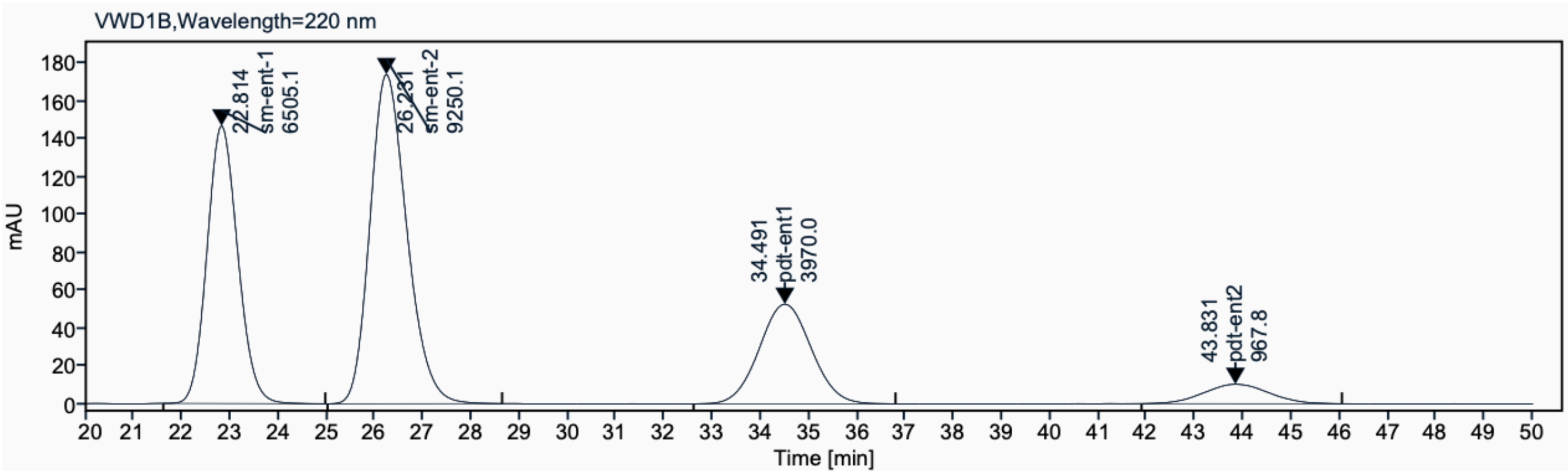

Name	RT [min]	Area
sm-ent-1	22.814	6505.1458
sm-ent-2	26.231	9250.0729
pdt-ent1	34.491	3969.9658
pdt-ent2	43.831	967.8365

Figure S48: Chiral HPLC trace of reaction catalyzed by G5OEt-C93S at 1h, Ph(^{o}An)P(O)H and EDA.

7.7 Ph(2-Np)P(O)–CH_2CO_2Et (8b)

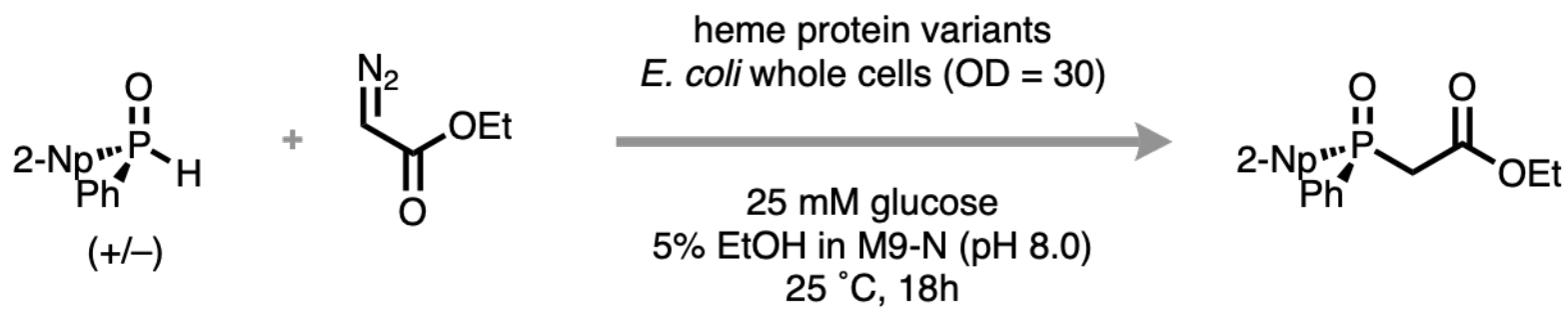

Ph(2-Np)P(O)H and EDA was screened against the Master Compilation Plate (P11) and three variants were chosen with which to perform validation with: G5OEt, G6.1, and G11.1. Absolute configuration of product assigned based on the assignment and consumption of substrate (Section 8.4).

Reactions were performed according to the validation procedure (three technical replicates for assay yield; two technical replicates for enantioselectivity).

Table S42: Yield and enantioselectivity of variants and selected reaction conditions for the reactions of Ph(2-Np)P(O)H and EDA.

Variant/conditions	Avg. Yield (%)	Std Dev Yield (%)	Avg. E.E. (%)	Std Dev E.E. (%)
G5OEt	18	5	-14	1
G6.1	40	4	-84	< 1
G11.1	43	0	-91	< 1

Reverse Phase HPLC-MS: Agilent Poroshell 120 EC-C18, 1.5 mL/min, [A] 0.1% AcOH in Water, [B] 0.1% AcOH in Acetonitrile, scan and single ion monitoring $[M+H]^+$, gradient: 0-0.2 min: 5-50% B; 0.2-0.4 min: 5–20% B; 0.4–2.5 min: 20–50% B; 2.5–2.6 min: 50–95% B; 2.6–3.0 min: 95% B; 3.0–3.1 min: 95–5% B; 3.1–3.5 min: 5% B.

Chiral HPLC: Chiralpak IA (Hex/IPA = 75:25, 1 mL/min).

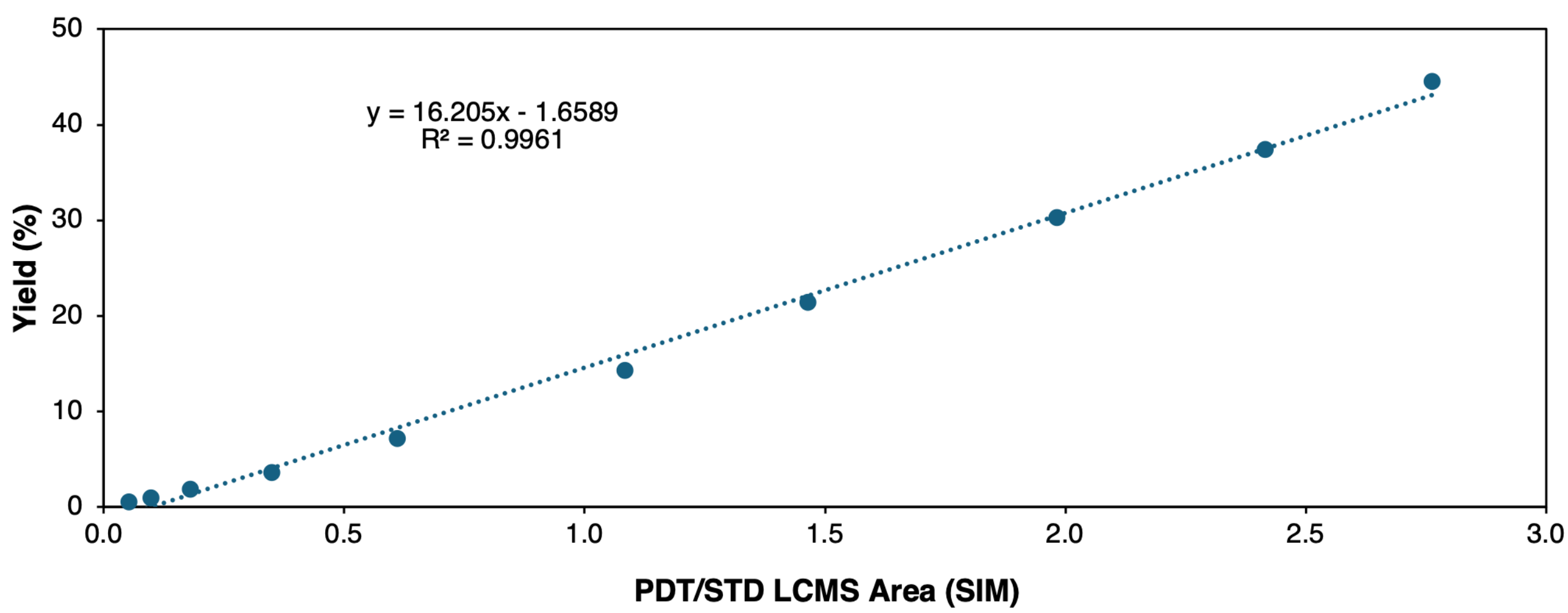

Figure S49: Calibration curve using authentic sample and papaverine as an external standard.

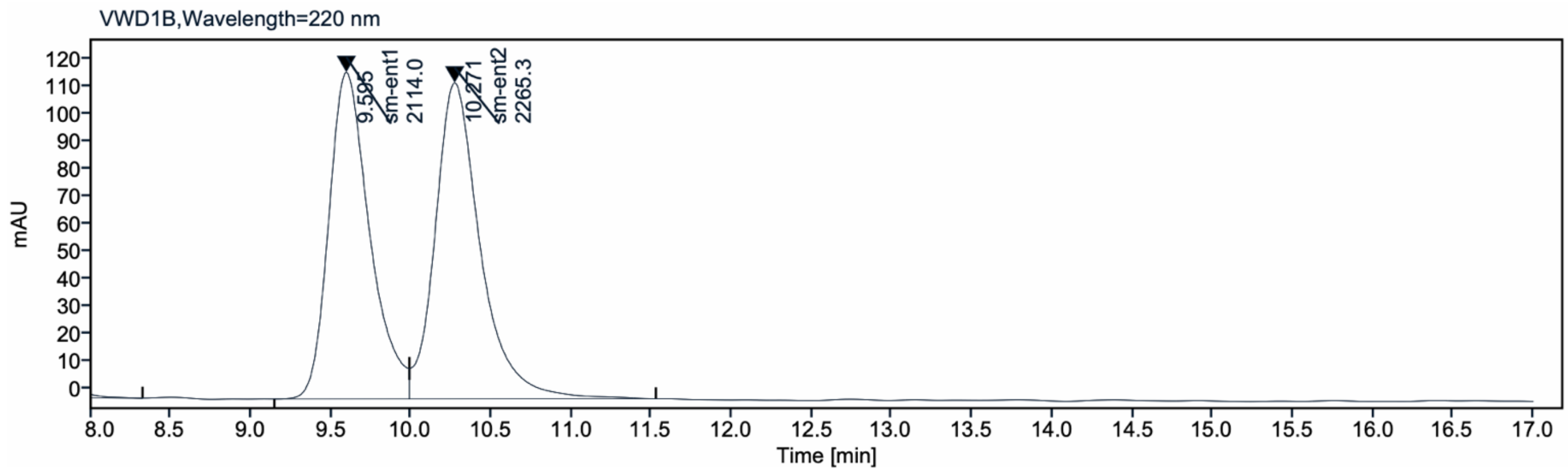

Name	RT [min]	Area
sm-ent1	9.595	2114.0427
sm-ent2	10.271	2265.2847

Figure S50: Chiral HPLC trace of racemic Ph(2-Np)P(O)H.

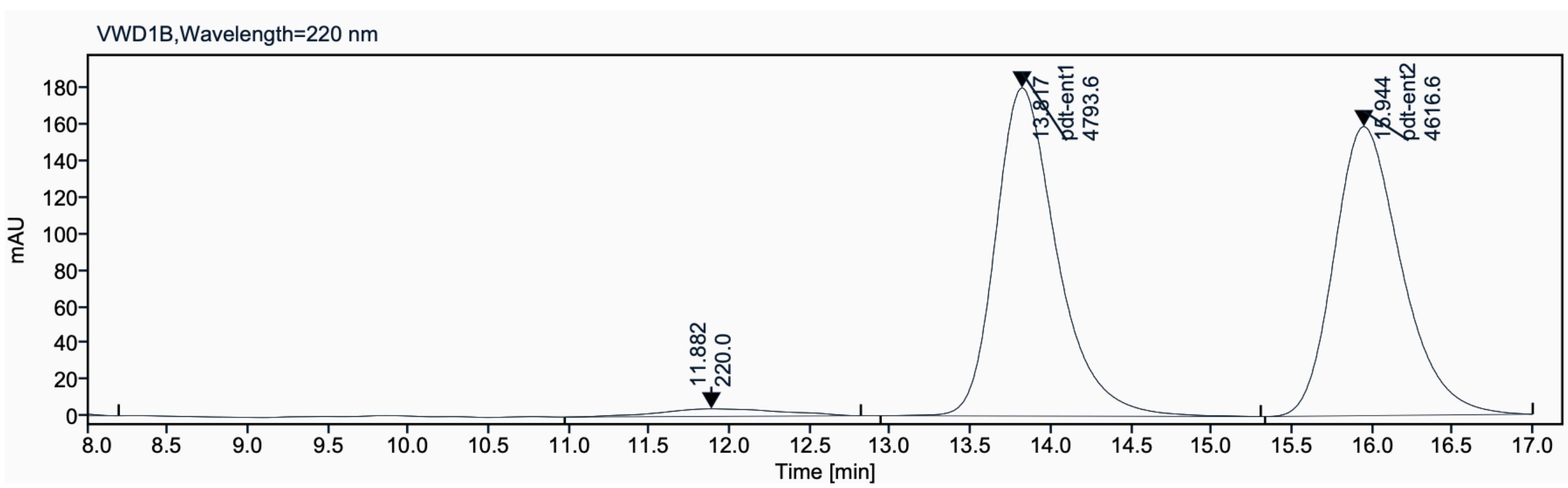

Name	RT [min]	Area
pdt-ent1	13.817	4793.5681
pdt-ent2	15.944	4616.6453

Figure S51: Chiral HPLC trace of racemic Ph(2-Np)P(O)–CH_2CO_2Et.

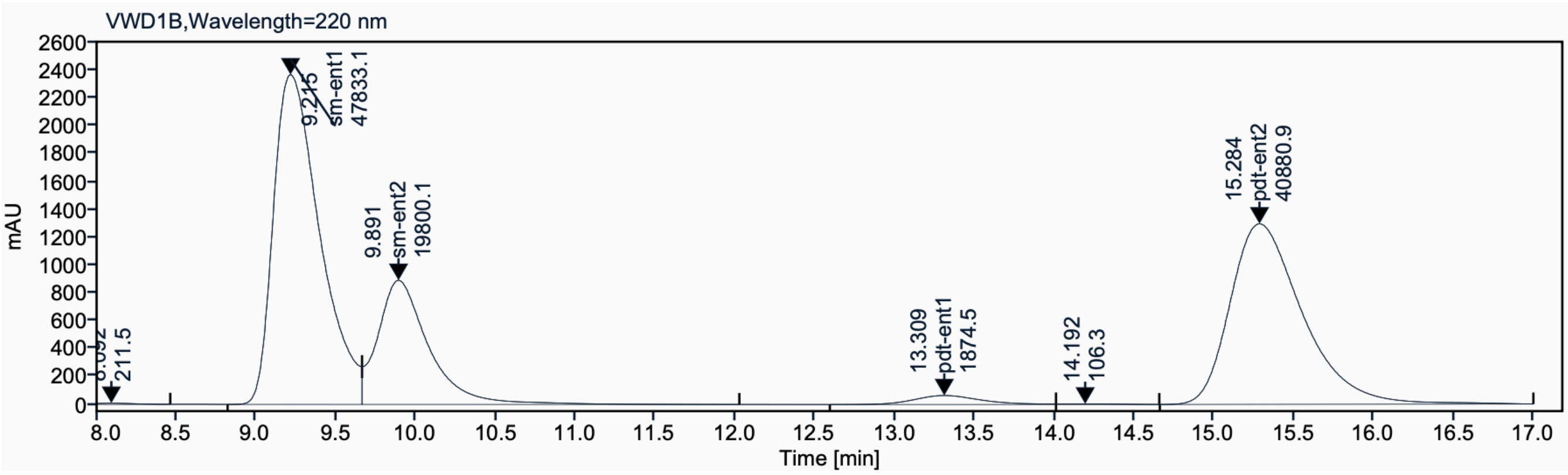

Name	RT [min]	Area
sm-ent1	9.215	47833.0560
sm-ent2	9.891	19800.0809
pdt-ent1	13.309	1874.4664
pdt-ent2	15.284	40880.8587

Figure S52: Chiral HPLC trace of reaction catalyzed by G11.1, Ph(2-Np)P(O)H and EDA.

7.8 Ph(OEt)P(O)–CH_2CO_2Et (2b)

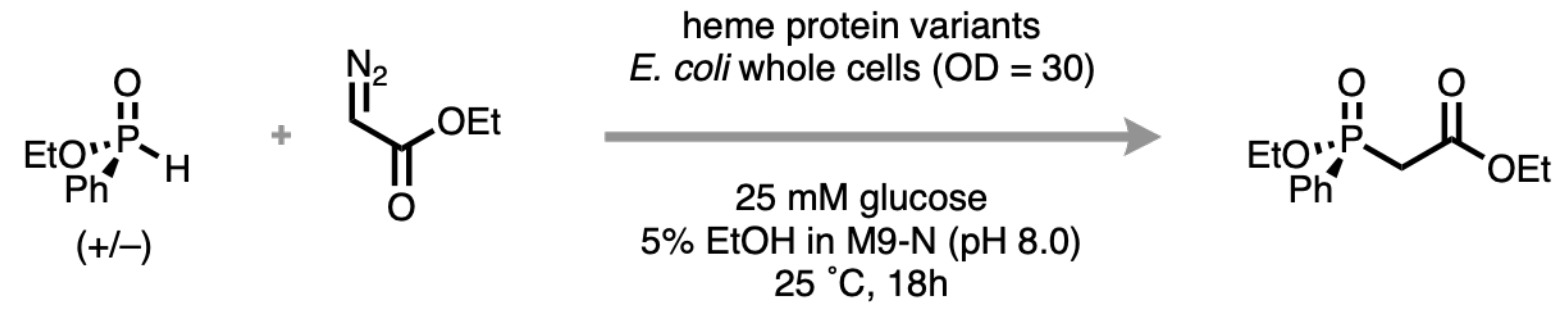

Reactions were performed according to the validation procedure, see Section 5.2 for assay yields and enantioselectivity across the whole lineage. Absolute configuration of product assigned based on product isolation (Section 8.2).

Large Scale (1 mmol): The standard validation procedure was followed using a larger scale, except the reaction was performed aerobically and stirred using a magnetic stir bar (800 rpm). Normalized cell suspension (G5OEt, 36 mL, OD_{600} = 30, M9-N, pH 8.0) was added to a 100-mL round bottom flask. To the flask was added glucose (2 mL, 500 mM, $[glc]_{final}$ = 25 mM) and a solution of Ph(OEt)P(O)H and EDA in EtOH (2 mL, 500 mM Ph(OEt)P(O)H, 375 mM EDA, $[P]_{final}$ = 25 mM, $[EDA]_{final}$ = 18.75 mM). The flask was sealed with a septum and stirred at room temperature for 24 h. The reaction was quenched with IPA (4 mL). The reaction was extracted with EtOAc (4x 30 mL) – centrifugation at 5000xg for 1 minute was used to aid layer formation. The combined organics were dried with Na_2SO_4 and solvent removed *in vacuo*. The reaction outcome was analyzed by adding nitrobenzene as an analytical external standard (40 µL) and an aliquot was removed to determine the reaction yield (^{1}H NMR Yield, 38%).

Column: The resulting crude residue was purified by silica gel chromatography (25g SiO_2, packed w/ 50% EtOAc in Hex). The crude residue was loaded in a minimal amount of DCM (200 µL x 3), and the product was eluted with a gradient (50–100% EtOAc in Hex over 10 CV). The fractions with product were concentrated in vacuo (30 ˚C). The product was isolated as mixture with RSM (97% ee).

Reverse Phase HPLC-MS: Agilent Poroshell 120 EC-C18, 1.5 mL/min, [A] 0.1% AcOH in Water, [B] 0.1% AcOH in Acetonitrile, scan and single ion monitoring $[M+H]^+$, gradient: 0-0.2 min: 5-50% B; 0.2-0.4 min: 5–20% B; 0.4–2.5 min: 20–50% B; 2.5–2.6 min: 50–95% B; 2.6–3.0 min: 95% B; 3.0–3.1 min: 95–5% B; 3.1–3.5 min: 5% B.

Chiral HPLC: Chiralpak IC in series (Hex/IPA = 50:50, 1 mL/min).

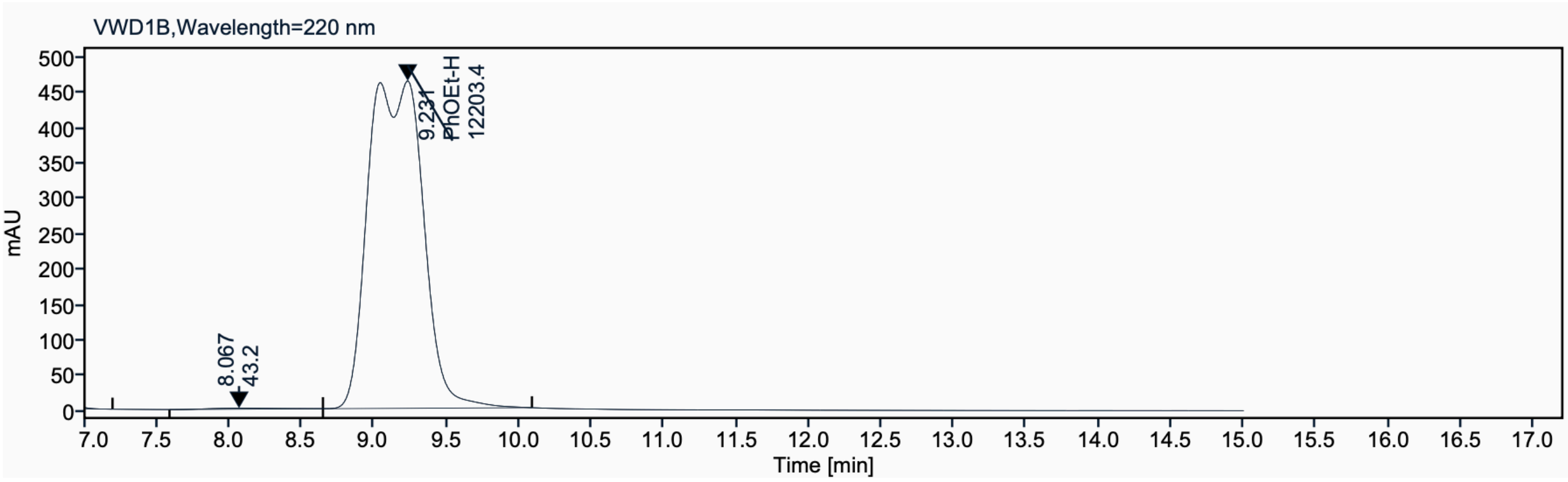

Name	RT [min]	Area
PhOEt-H	9.231	12203.4077

Figure S53: Chiral HPLC trace of racemic Ph(OEt)P(O)H.

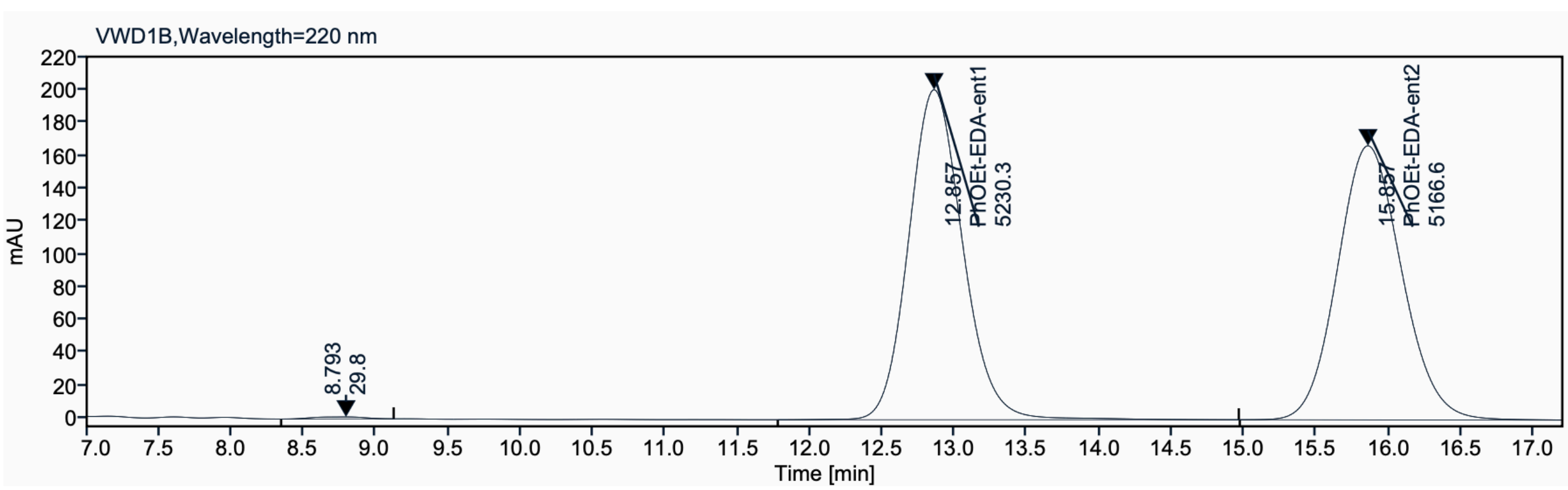

Name	RT [min]	Area
PhOEt-EDA-ent1	12.857	5230.2549
PhOEt-EDA-ent2	15.857	5166.6069

Figure S54: Chiral HPLC trace of racemic Ph(OEt)P(O)–CH_2CO_2Et.

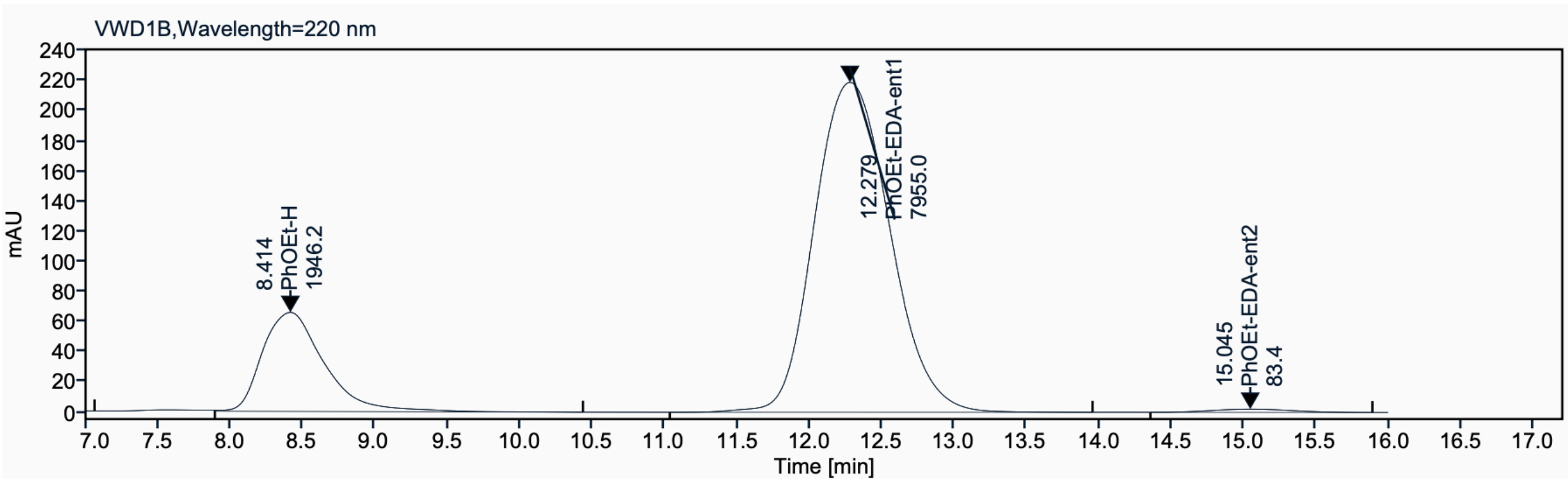

Name	RT [min]	Area
PhOEt-H	8.414	1946.2009
PhOEt-EDA-ent1	12.279	7954.9795
PhOEt-EDA-ent2	15.045	83.4116

Figure S55: Chiral HPLC trace of reaction catalyzed by G5OEt, Ph(OEt)P(O)H and EDA.

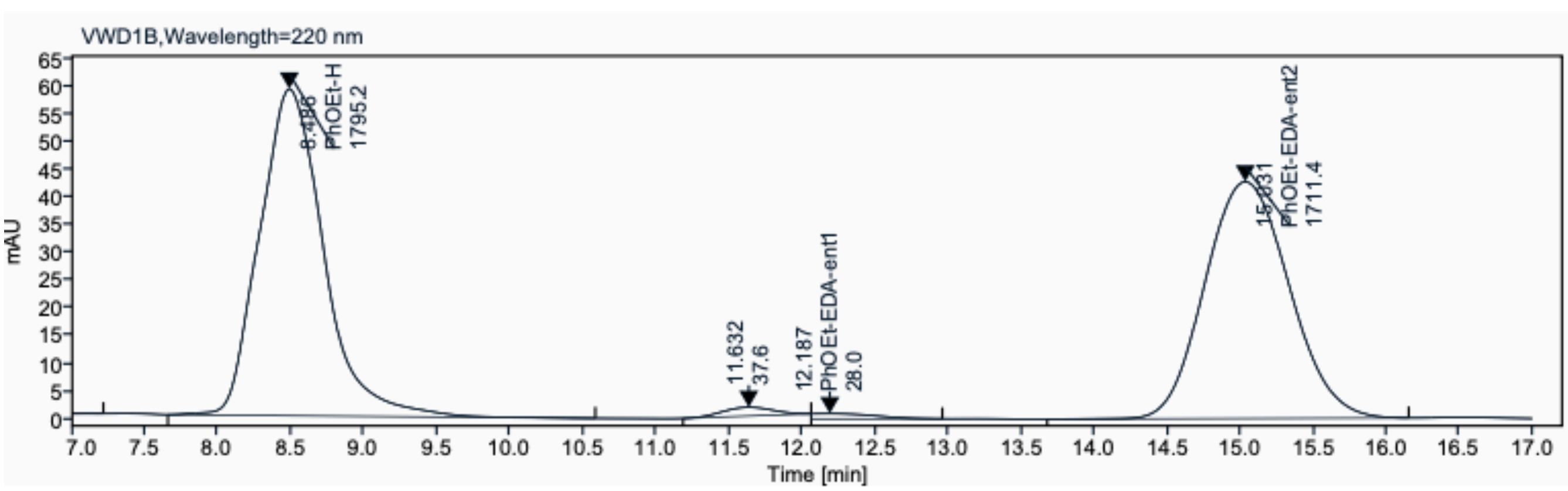

Name	RT [min]	Area
PhOEt-H	8.486	1795.2362
PhOEt-EDA-ent1	12.187	28.0183
PhOEt-EDA-ent2	15.031	1711.3831

Figure S56: Chiral HPLC trace of reaction catalyzed by G11.1, Ph(OEt)P(O)H and EDA.

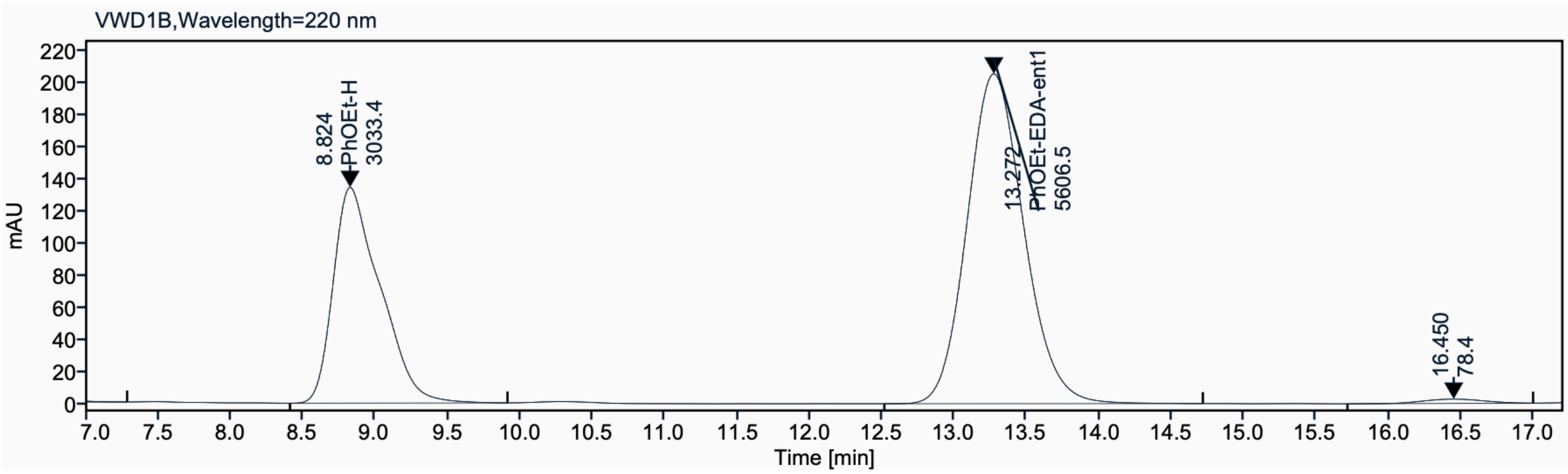

Name	RT [min]	Area
PhOEt-H	8.824	3033.3790
PhOEt-EDA-ent1	13.272	5606.4926

Figure S57: Chiral HPLC trace of purified product from large scale reaction catalyzed by G5OEt, Ph(OEt)P(O)H and EDA.

7.9 (4-F)Ph(OEt)P(O)–CH_2CO_2Et (9b)

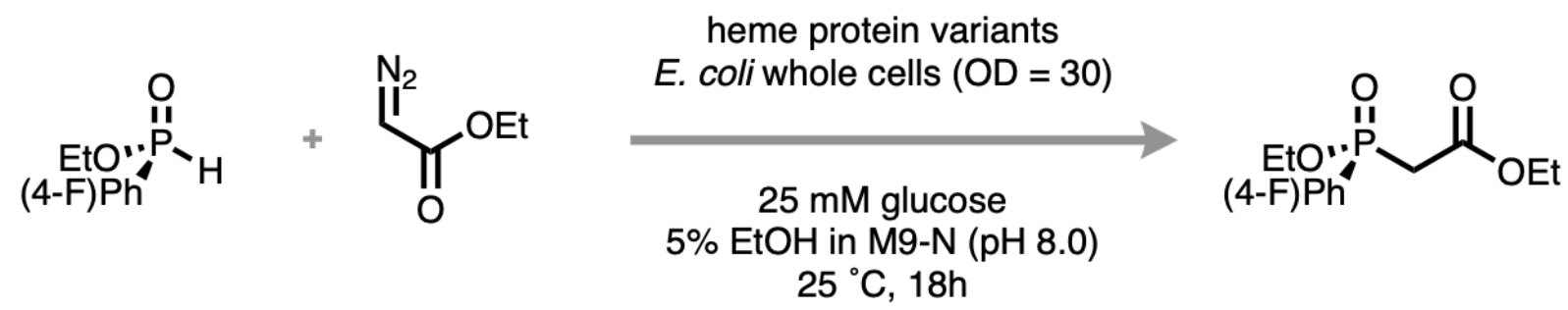

(4-F)Ph(OEt)P(O)H and EDA was screened against the Master Compilation Plate (P11) and two variants were chosen with which to perform validation with, G5OEt and G11.1. Absolute configuration of product assigned based on comparison to the reactivity of selected variants with other H-phosphinates substrates.

Reactions were performed according to the validation procedure (three technical replicates for assay yield; one technical replicate for enantioselectivity).

Table S43: Yield and enantioselectivity of variants and selected reaction conditions for the reactions of (4-F)Ph(OEt)P(O)H and EDA.

Variant/conditions	Avg. Yield (%)	Std Dev Yield (%)	Avg. E.E. (%)
G5OEt	52	1	96
G11.1	19	2	-86

Large Scale (0.4 mmol): The standard validation procedure was followed using a larger scale, except the reaction was performed in a 40-mL vial. A 40-mL vial was charged with normalized cell suspension normalized cell suspension (G5OEt, 18 mL, OD_{600} = 30, M9-N, pH 8.0) and glucose (1 mL, 500 mM, $[glc]_{final}$ = 25 mM). The vial was brought into the anaerobic COY chamber, and a solution of (4-F)Ph(OEt)P(O)H and EDA in EtOH (1 mL, 400 mM (4-F)Ph(OEt)P(O)H, 300 mM EDA, $[P]_{final}$ = 20 mM, $[EDA]_{final}$ = 15 mM) was added. The vial was sealed with a PTFE-lined cap and shaken at room temperature for 24 h. The reaction was quenched with IPA (2 mL). The reaction was extracted with EtOAc (4x 20 mL) – centrifugation at 5000xg for 1 minute was used to aid layer formation. The combined organics were dried with Na_2SO_4 and solvent removed *in vacuo*. The reaction outcome was analyzed by adding nitrobenzene as an analytical external standard (20 µL), and an aliquot was removed to determine the reaction yield (^{1}H NMR Yield, 23%).

Column: The resulting crude residue was purified by silica gel chromatography (10g SiO_2, packed w/ 50% EtOAc in Hex). The crude residue was loaded in a minimal amount of DCM (200 µL x 3), and the product was eluted with a gradient (50–100% EtOAc in Hex over 10 CV followed by 0–10% MeOH in EtOAc). The fractions with product were concentrated in vacuo (30 ˚C). The product was obtained as a clear oil (22.5 mg, 21%, 96% ee).

^{1}H NMR: (400 MHz, $CDCl_3$) δ 7.83 (dddd, *J* = 11.9, 6.4, 5.5, 2.0 Hz, 2H), 7.23 – 7.07 (m, 2H), 4.30 – 3.96 (m, 4H), 3.12 (dd, *J* = 18.1, 3.2 Hz, 2H), 1.34 (t, *J* = 7.0 Hz, 3H), 1.16 (t, *J* = 7.1 Hz, 3H).

^{19}F NMR (376 MHz, $CDCl_3$) δ -105.27 (s, 1F).

^{31}P NMR: (162 MHz, $CDCl_3$) δ 33.01.

Optical Rotation: $[\alpha]^{23}_D$ = + 8.6 (c = 1, MeOH).

Reverse Phase HPLC-MS: Agilent Poroshell 120 EC-C18, 1.5 mL/min, [A] 0.1% AcOH in Water, [B] 0.1% AcOH in Acetonitrile, scan and single ion monitoring $[M+H]^+$, gradient: 0-0.2 min: 5-50% B; 0.2-0.4 min: 5–20% B; 0.4–2.5 min: 20–50% B; 2.5–2.6 min: 50–95% B; 2.6–3.0 min: 95% B; 3.0–3.1 min: 95–5% B; 3.1–3.5 min: 5% B.

Chiral HPLC: Chiralpak IB (Hex/IPA = 50:50, 1 mL/min).

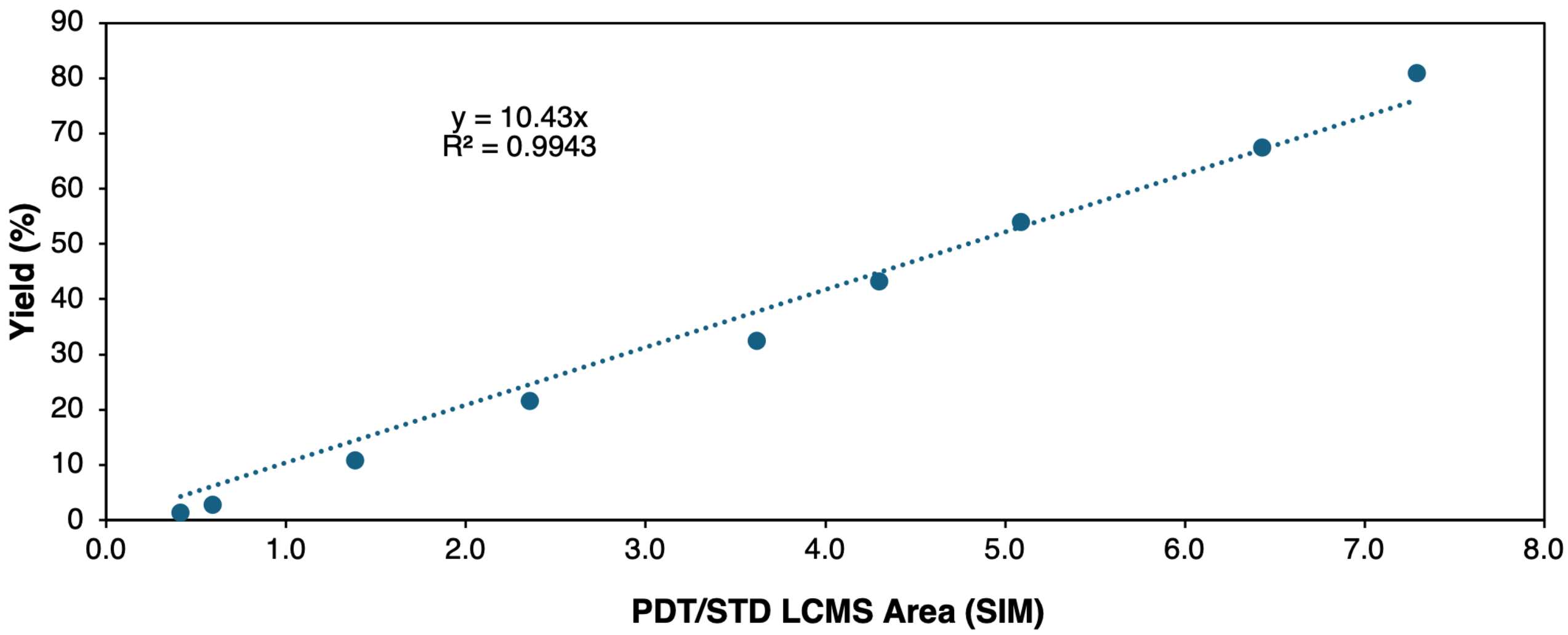

Figure S58: Calibration curve using authentic sample and papaverine as an external standard.

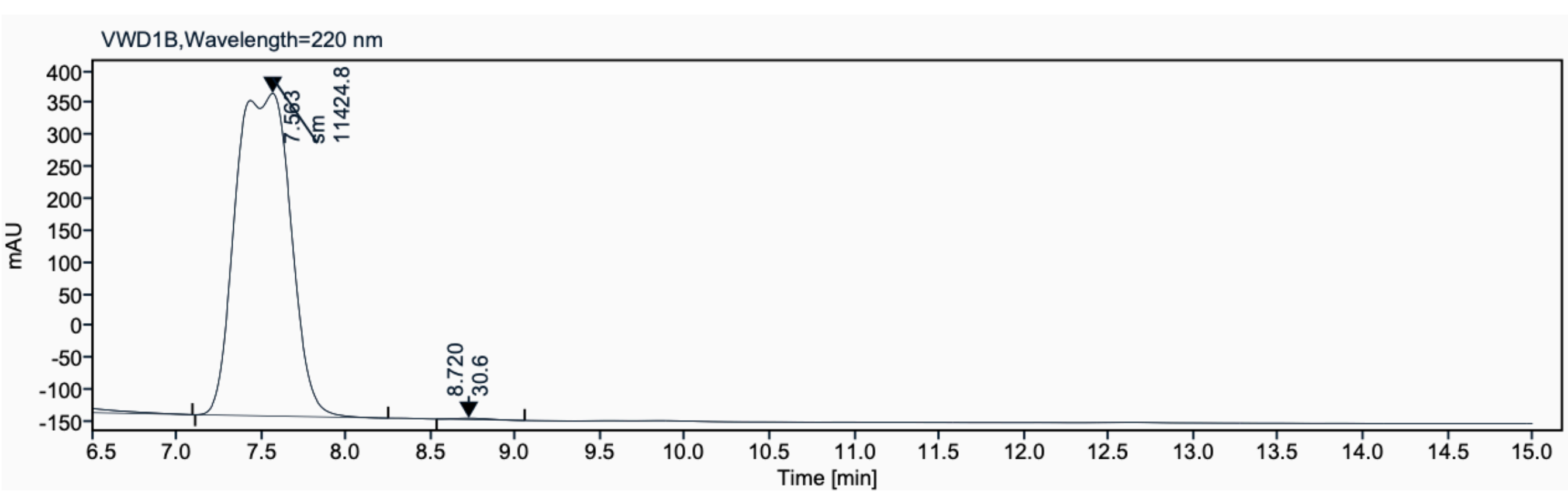

Name	RT [min]	Area
sm	7.563	11424.7898

Figure S59: Chiral HPLC trace of racemic (4-F)Ph(OEt)P(O)H.

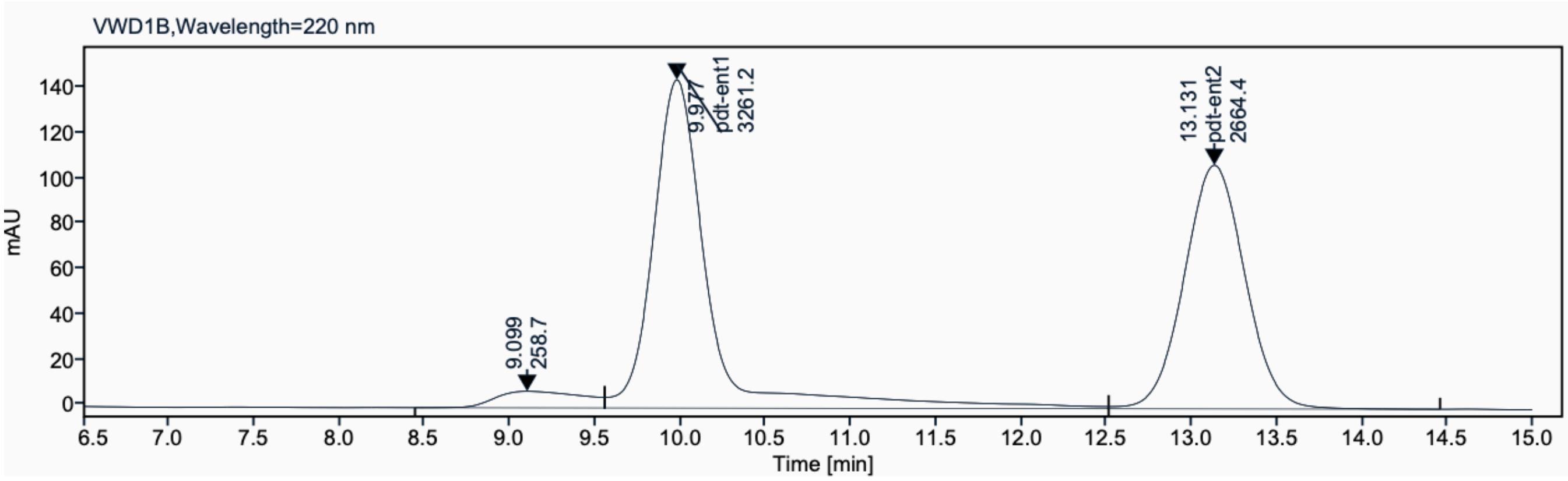

Name	RT [min]	Area
pdt-ent1	9.977	3261.2084
pdt-ent2	13.131	2664.4039

Figure S60: Chiral HPLC trace of racemic (4-F)Ph(OEt)P(O)–CH_2CO_2Et.

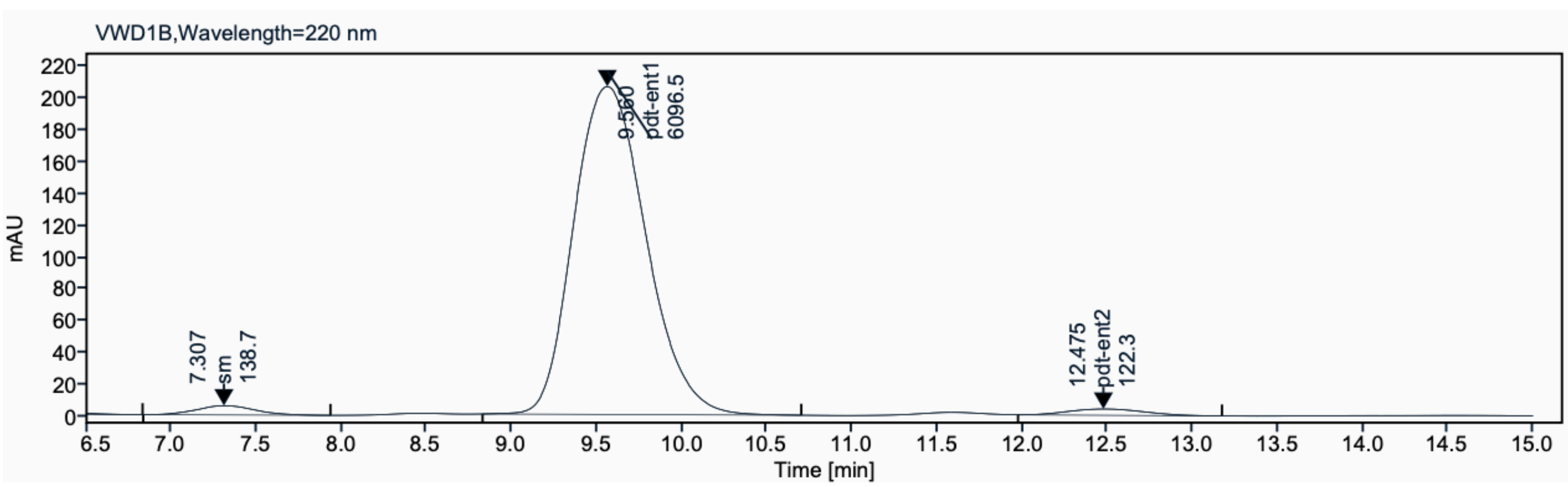

Name	RT [min]	Area
sm	7.307	138.6760
pdt-ent1	9.560	6096.5465
pdt-ent2	12.475	122.2603

Figure S61: Chiral HPLC trace of reaction catalyzed by G5OEt, (4-F)Ph(OEt)P(O)H and EDA.

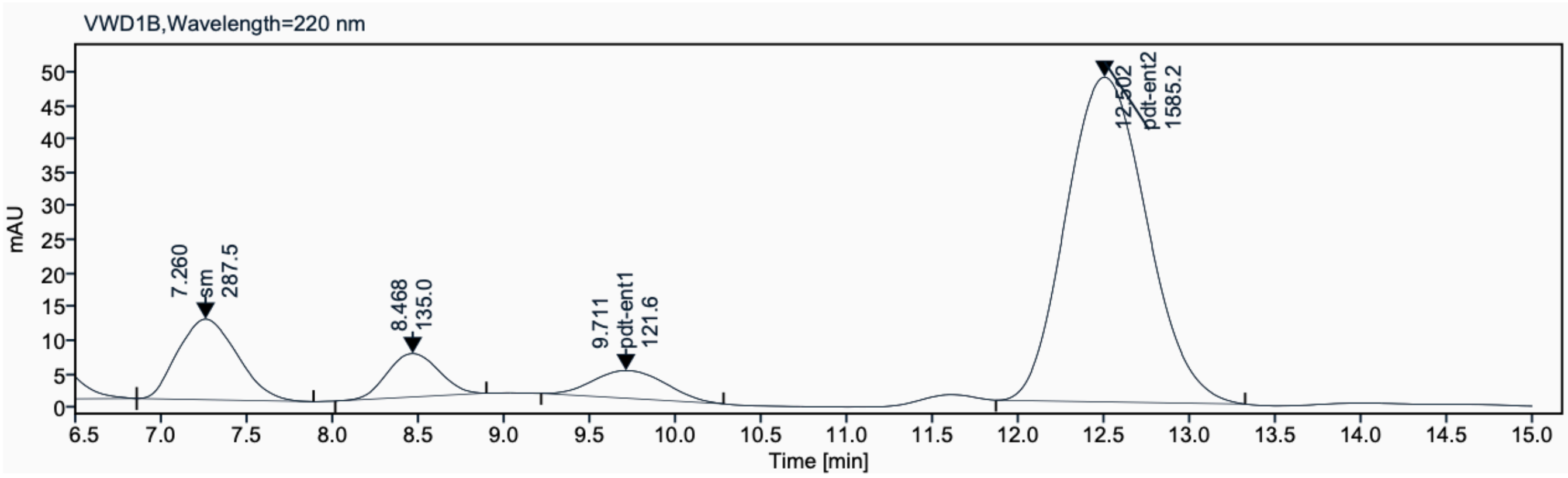

Name	RT [min]	Area
sm	7.260	287.4893
pdt-ent1	9.711	121.6012
pdt-ent2	12.502	1585.2475

Figure S62: Chiral HPLC trace of reaction catalyzed by G11.1, (4-F)Ph(OEt)P(O)H and EDA.

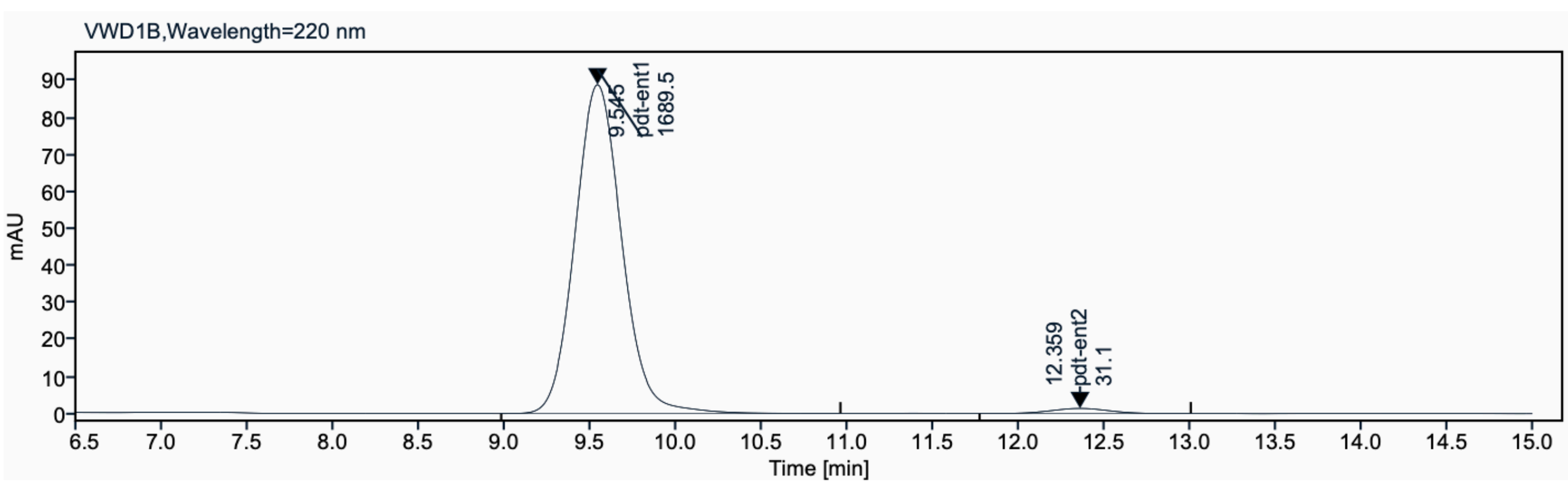

Name	RT [min]	Area
pdt-ent1	9.545	1689.5275
pdt-ent2	12.359	31.1455

Figure S63: Chiral HPLC trace of large-scale reaction catalyzed by G5OEt, (4-F)Ph(OEt)P(O)H and EDA.

7.10 ^{p}Tol(OEt)P(O)–CH_2CO_2Et (10b)

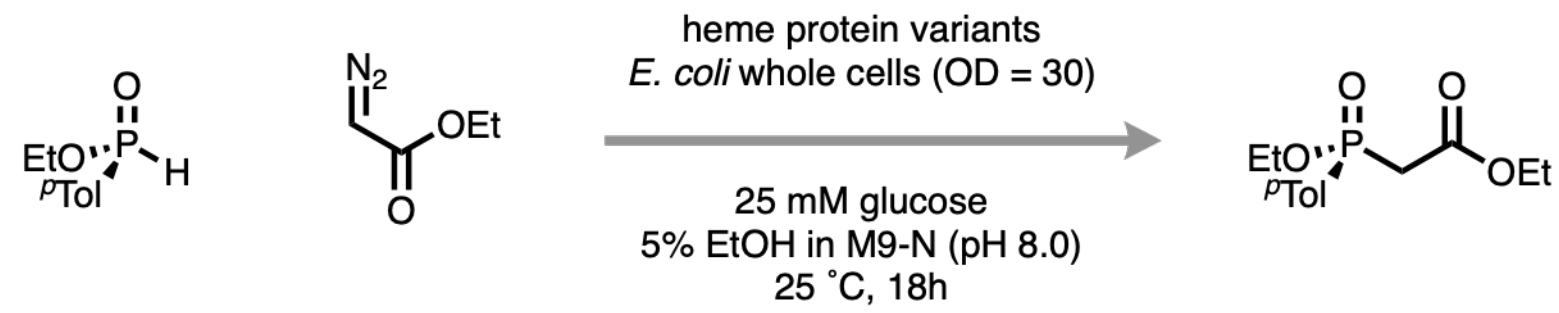

^{p}Tol(OEt)P(O)H and EDA was screened against the Master Compilation Plate (P11) and three variants were chosen with which to perform validation with, G5OEt, G5OEt-L86Q, and G11.1. Absolute configuration of product assigned based on comparison to the reactivity of selected variants with other H-phosphinates substrates.

Reactions were performed according to the validation procedure (three technical replicates for assay yield, one technical replicate for enantioselectivity).

Table S44: Yield and enantioselectivity of variants and selected reaction conditions for the reactions of ^{p}Tol(OEt)P(O)H and EDA.

Variant/conditions	Avg. Yield (%)	Std Dev Yield (%)	Avg. E.E. (%)
G5OEt	10	2	80
G5OEt-L86Q	12	< 1	83
G11.1	16	< 1	-99

Reverse Phase HPLC-MS: Agilent Poroshell 120 EC-C18, 1.5 mL/min, [A] 0.1% AcOH in Water, [B] 0.1% AcOH in Acetonitrile, scan and single ion monitoring $[M+H]^+$, gradient: 0-0.2 min: 5-50% B; 0.2-0.4 min: 5–20% B; 0.4–2.5 min: 20–50% B; 2.5–2.6 min: 50–95% B; 2.6–3.0 min: 95% B; 3.0–3.1 min: 95–5% B; 3.1–3.5 min: 5% B.

Chiral HPLC: Chiralpak IC (Hex/IPA = 75:25, 1 mL/min).

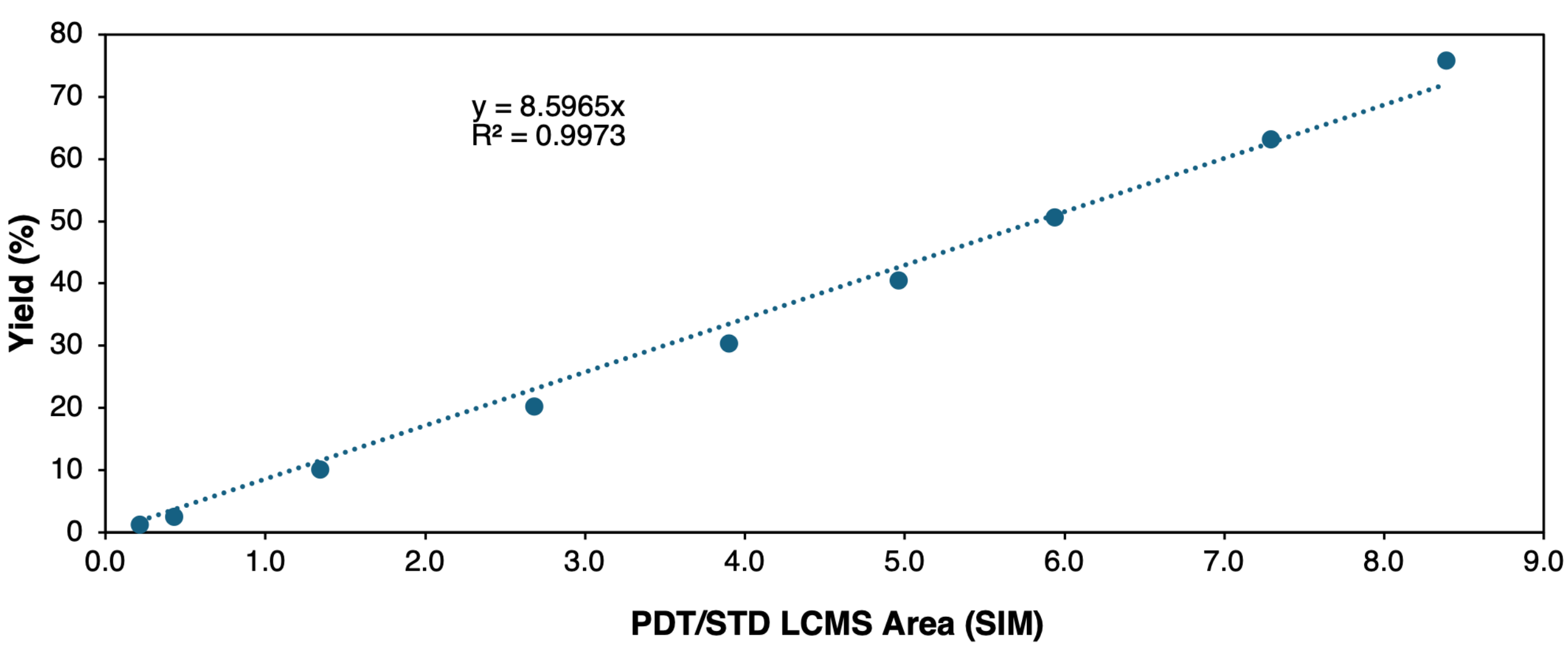

Figure S64: Calibration curve using authentic sample and papaverine as an external standard.

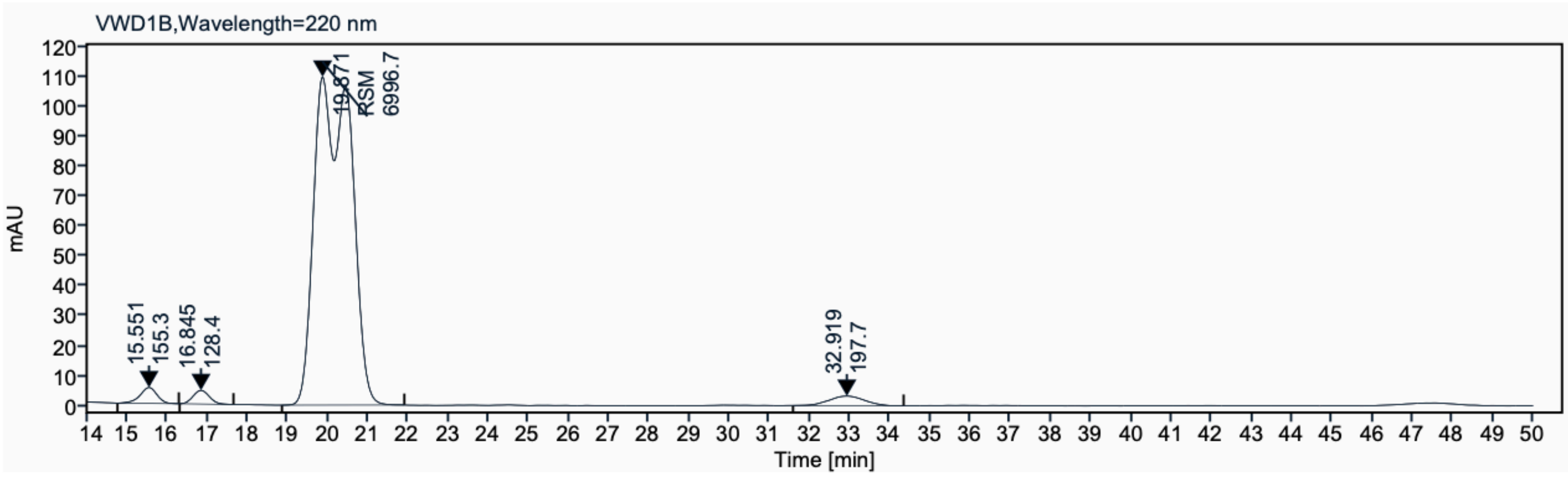

Name	RT [min]	Area
RSM	19.871	6996.6933

Figure S65: Chiral HPLC trace of racemic ^{p}Tol(OEt)P(O)H.

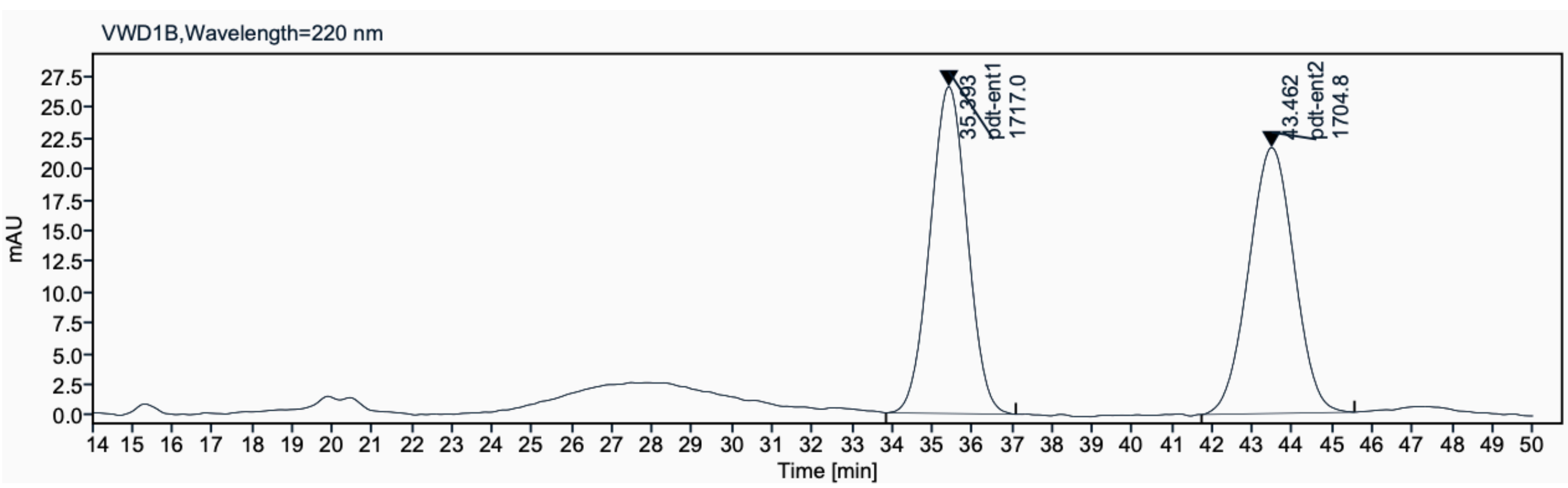

Name	RT [min]	Area
pdt-ent1	35.393	1716.9595
pdt-ent2	43.462	1704.7803

Figure S66: Chiral HPLC trace of racemic ^{p}Tol(OEt)P(O)–CH_2CO_2Et.

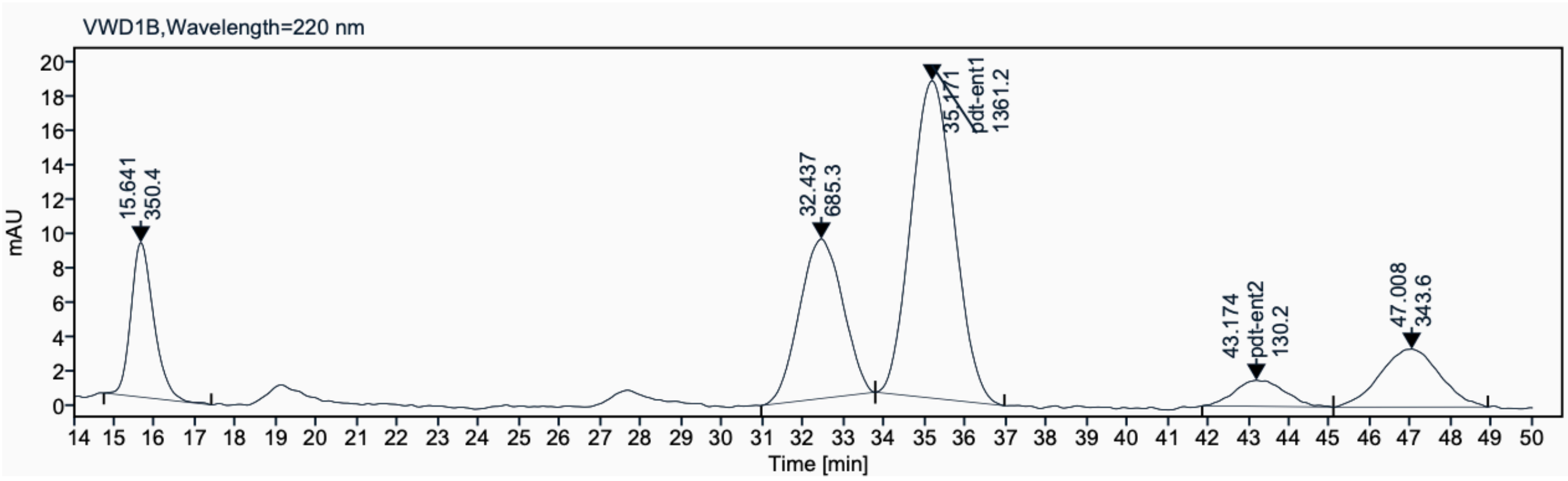

Name	RT [min]	Area
pdt-ent1	35.171	1361.2006
pdt-ent2	43.174	130.1682

Figure S67: Chiral HPLC trace of reaction catalyzed by G5OEt-L86Q, ^{p}Tol(OEt)P(O)H and EDA.

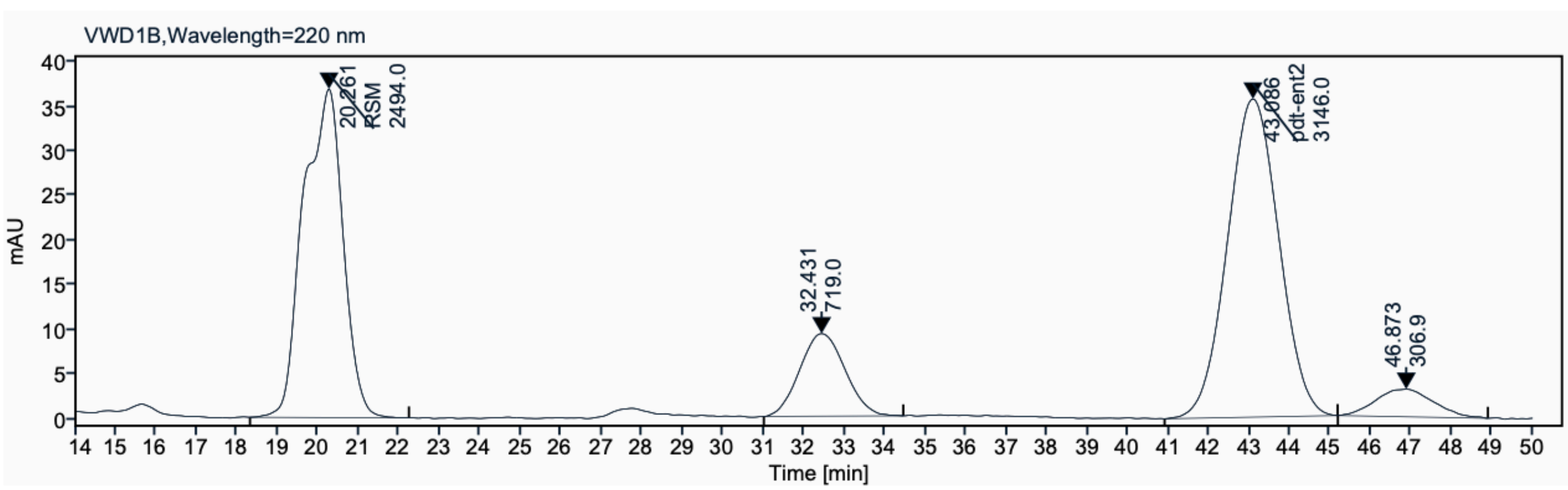

Name	RT [min]	Area
RSM	20.261	2493.9860
pdt-ent2	43.086	3146.0331

Figure S68: Chiral HPLC trace of reaction catalyzed by G11.1, ^{p}Tol(OEt)P(O)H and EDA.

7.11 (2-Np)(OEt)P(O)–CH_2CO_2Et (11b)

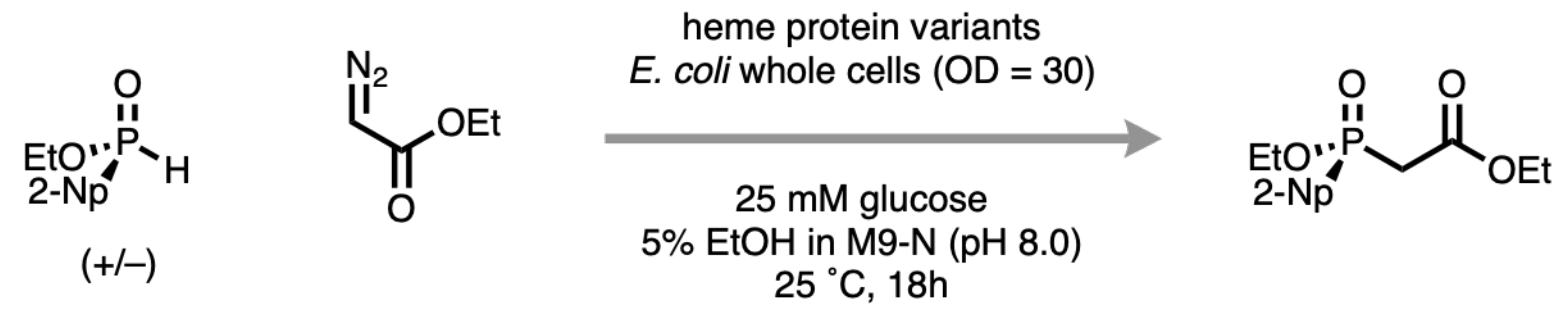

2-Np(OEt)P(O)H and EDA was screened against the Master Compilation Plate (P11) and two variants were chosen with which to perform validation with, G11.1 and G11.1-C181R. Absolute configuration of product assigned based on comparison to the reactivity of selected variants with other H-phosphinates substrates.

Reactions were performed according to the validation procedure (three technical replicates for assay yield; one technical replicate for enantioselectivity).

Table S45: Yield and enantioselectivity of variants and selected reaction conditions for the reactions of 2-Np(OEt)P(O)H and EDA.

Variant/conditions	Avg. Yield (%)	Std Dev Yield (%)	Avg. E.E. (%)
G11.1	25	1	-99
G11.1-C181R	27	< 1	N.D.

Reverse Phase HPLC-MS: Agilent Poroshell 120 EC-C18, 1.5 mL/min, [A] 0.1% AcOH in Water, [B] 0.1% AcOH in Acetonitrile, scan and single ion monitoring $[M+H]^+$, gradient: 0-0.2 min: 5-50% B; 0.2-0.4 min: 5–20% B; 0.4–2.5 min: 20–50% B; 2.5–2.6 min: 50–95% B; 2.6–3.0 min: 95% B; 3.0–3.1 min: 95–5% B; 3.1–3.5 min: 5% B.

Chiral HPLC: Chiralpak IC (Hex/IPA = 75:25, 1 mL/min).

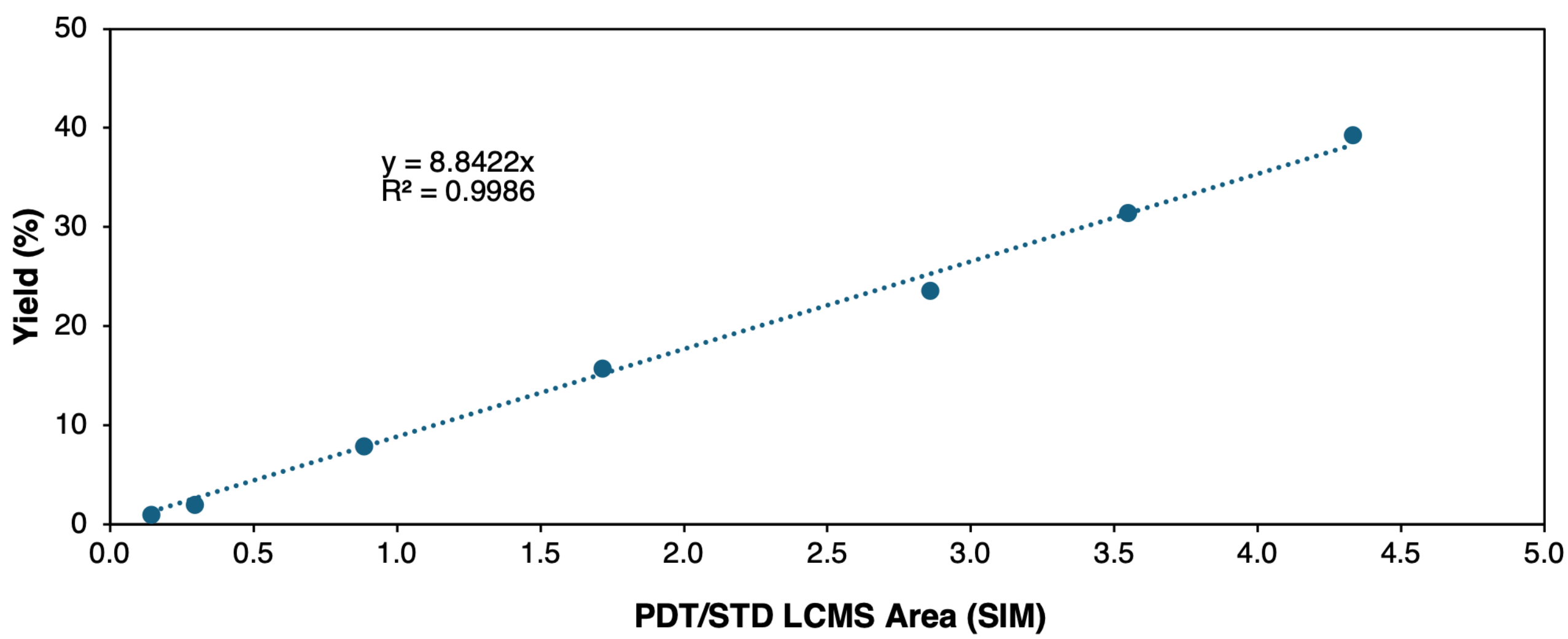

Figure S69: Calibration curve using authentic sample and papaverine as an external standard.

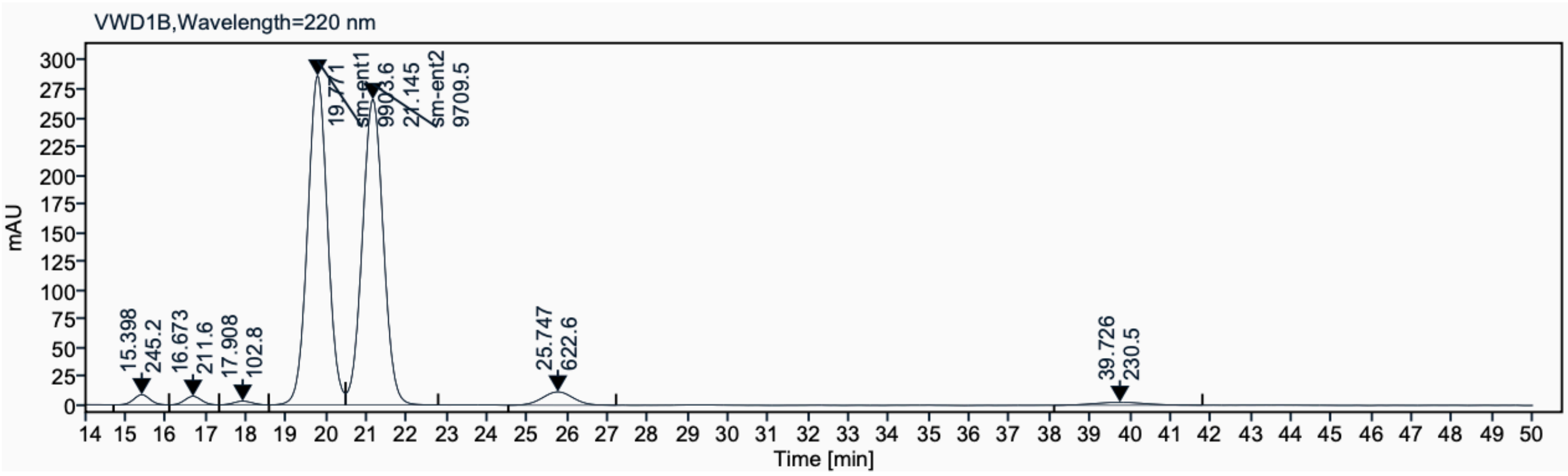

Name	RT [min]	Area
sm-ent1	19.771	9903.5525
sm-ent2	21.145	9709.5044

Figure S70: Chiral HPLC trace of racemic 2-Np(OEt)P(O)H.

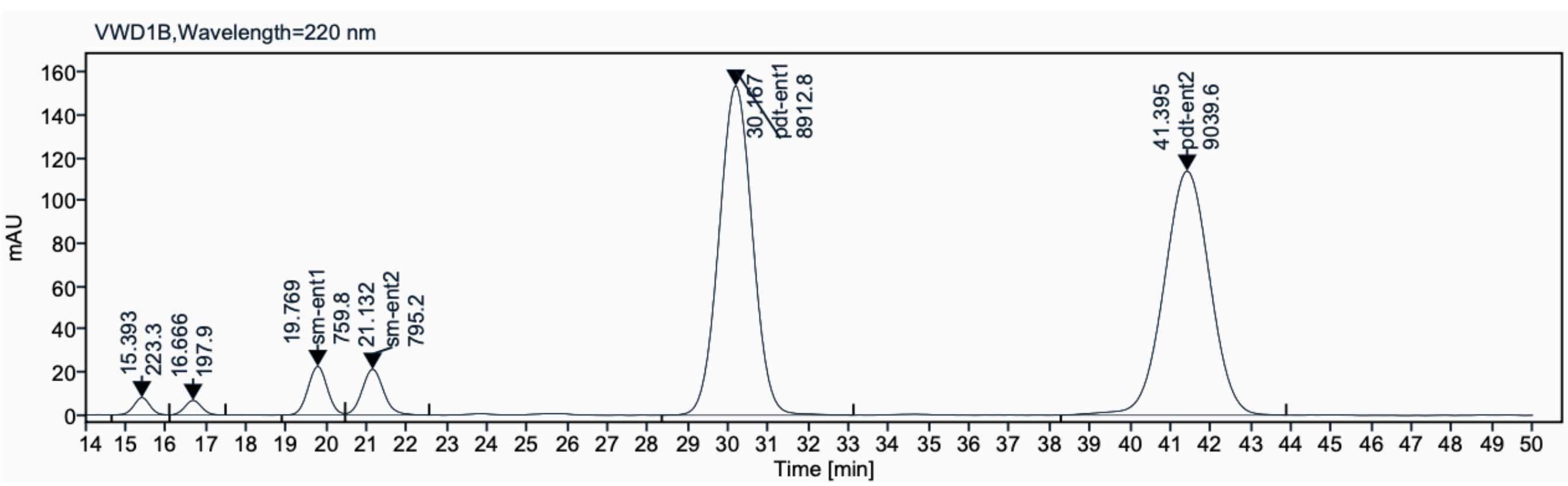

Name	RT [min]	Area
sm-ent1	19.769	759.8253
sm-ent2	21.132	795.2431
pdt-ent1	30.167	8912.7714
pdt-ent2	41.395	9039.6162

Figure S71: Chiral HPLC trace of racemic 2-Np(OEt)P(O)–CH_2CO_2Et.

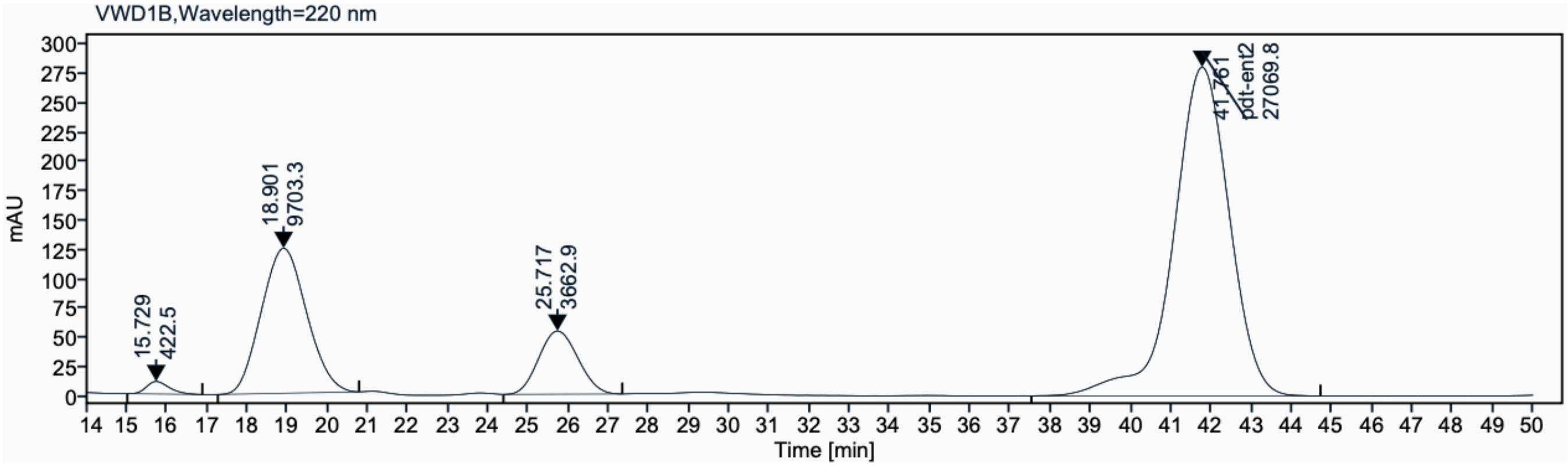

Name	RT [min]	Area
pdt-ent2	41.761	27069.7735

Figure S72: Chiral HPLC trace of reaction catalyzed by G11.1, 2-Np(OEt)P(O)H and EDA.

7.12 $PhCH_2CH_2(OEt)P(O)–CH_2CO_2Et$ (12b)

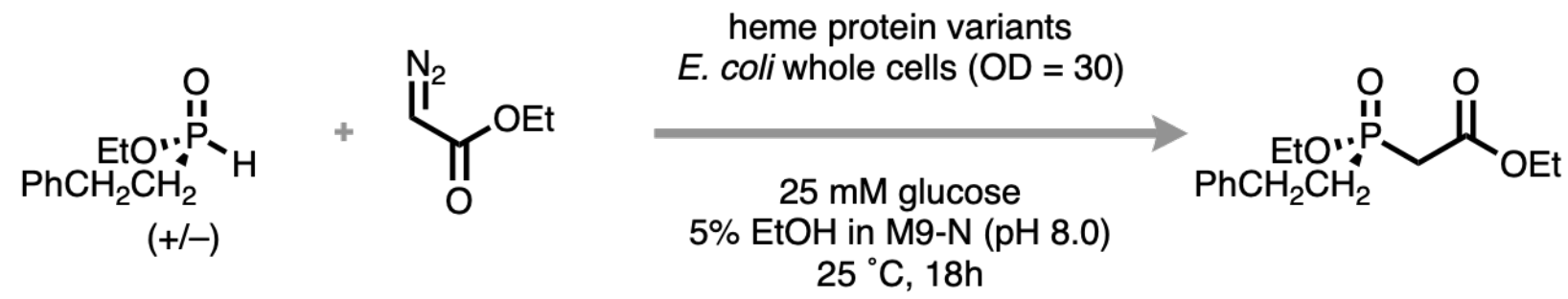

$PhCH_2CH_2(OEt)P(O)H$ and EDA was screened against the Master Compilation Plate (P11) and two variants were chosen with which to perform validation with: G5OEt and G11.1. Absolute configuration of product assigned based on comparison to the reactivity of selected variants with other H-phosphinates substrates; specifically the favored reactivity with G11.1.

Reactions were performed according to the validation procedure (three technical replicates for assay yield, one technical replicate for enantioselectivity).

Table S46: Yield and enantioselectivity of variants and selected reaction conditions for the reactions of $PhCH_2CH_2(OEt)P(O)H$ and EDA.

Variant/conditions	Avg. Yield (%)	Std Dev Yield (%)	Avg. E.E. (%)
G5OEt	19	4	44
G11.1	49	2	> 99

Large Scale (0.4 mmol): The standard validation procedure was followed using a larger scale, except the reaction was performed in a 40-mL vial. A 40-mL vial was charged with normalized cell suspension normalized cell suspension (G11.1, 18 mL, OD_{600} = 30, M9-N, pH 8.0) and glucose (1 mL, 500 mM, $[glc]_{final}$ = 25 mM). The vial was brought into the anaerobic COY chamber and a solution of $PhCH_2CH_2(OEt)P(O)H$ and EDA in EtOH (1 mL, 400 $PhCH_2CH_2(OEt)P(O)H$, 300 mM EDA, $[P]_{final}$ = 20 mM, $[EDA]_{final}$ = 15 mM) was added. The vial was sealed with a PTFE lined cap and shaken at room temperature for 24 h. The reaction was quenched with IPA (2 mL) and a 400-µL aliquot was removed for chiral HPLC analysis (>99% ee). The reaction was extracted with EtOAc (4x 20 mL) – centrifugation at 5000xg for 1 minute was used to aid layer formation. The combined organics were dried with Na_2SO_4 and solvent removed *in vacuo*. The reaction outcome was analyzed by adding nitrobenzene as an analytical external standard (20 µL) and an aliquot was removed to determine the reaction yield (^{1}H NMR Yield, 21%).

Column: The resulting crude residue was purified by silica gel chromatography (10g SiO_2, packed w/ 60% EtOAc in Hex). The crude residue was loaded in a minimal amount of DCM (200 µL x 3), and the product was eluted with a gradient (60–100% EtOAc in Hex over 15 CV followed by 100% EtOAc). The fractions with product were concentrated *in vacuo* (30 ˚C). The product was obtained as a clear oil (15.7 mg, 14%, >99% ee).

^{1}H NMR: (400 MHz, $CDCl_3$) δ 7.33 – 7.27 (m, 2H), 7.25 – 7.18 (m, 3H), 4.28 – 4.00 (m, 4H), 3.06 – 2.83 (m, 4H), 2.21 (dddd, J = 14.7, 10.8, 6.8, 2.0 Hz, 2H), 1.33 (t, J = 7.0 Hz, 3H), 1.28 (t, J = 7.2 Hz, 3H).

^{31}P NMR: (162 MHz, $CDCl_3$) δ 46.64.

Optical Rotation: $[\alpha]^{23}_D$ = + 3.2 (c = 1, MeOH).

Reverse Phase HPLC-MS: Agilent Poroshell 120 EC-C18, 1.5 mL/min, [A] 0.1% AcOH in Water, [B] 0.1% AcOH in Acetonitrile, scan and single ion monitoring $[M+H]^+$, gradient: 0-0.2 min: 5-50% B; 0.2-0.4 min: 5–20% B; 0.4–2.5 min: 20–50% B; 2.5–2.6 min: 50–95% B; 2.6–3.0 min: 95% B; 3.0–3.1 min: 95–5% B; 3.1–3.5 min: 5% B.

Chiral SFC: (IF-3, 2.5 mL/min, 15% IPA in CO_2, MSD): t_R (major) = 4.9 min, t_R (minor) = 5.5 min.

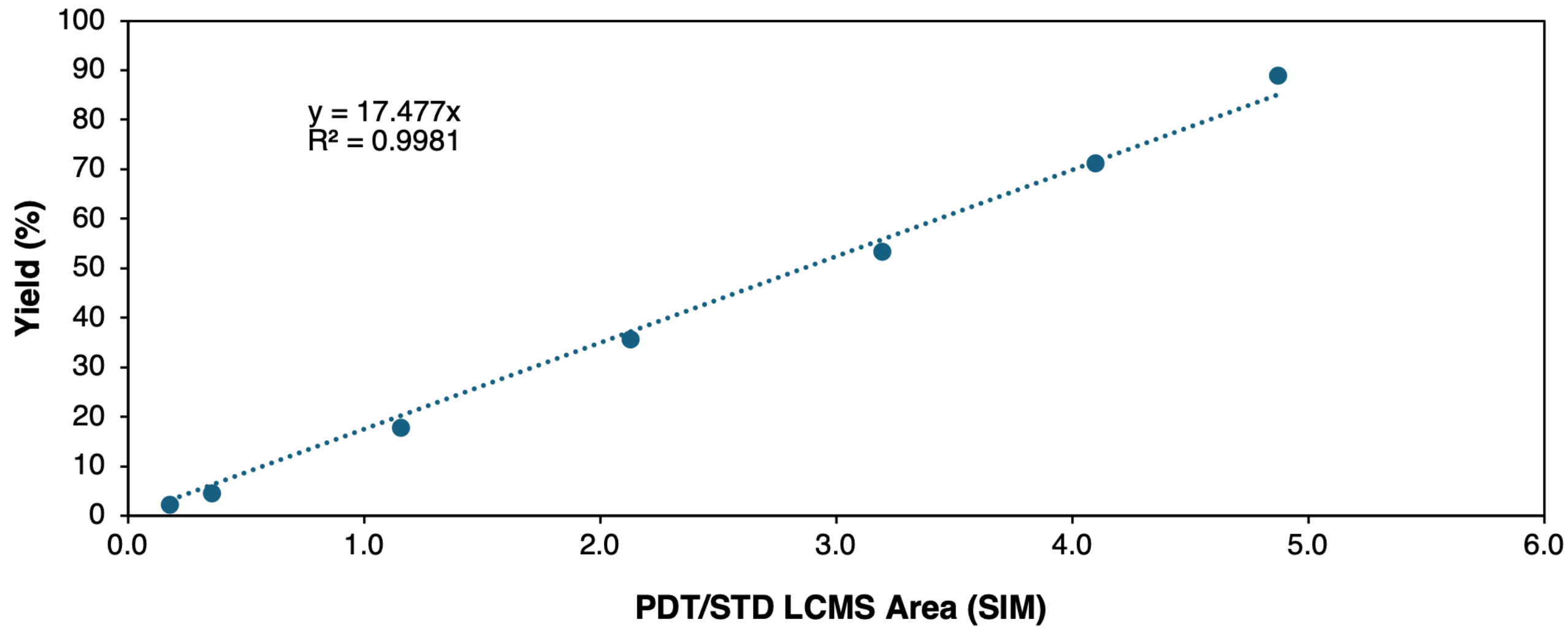

Figure S73: Calibration curve using authentic sample and papaverine as an external standard.

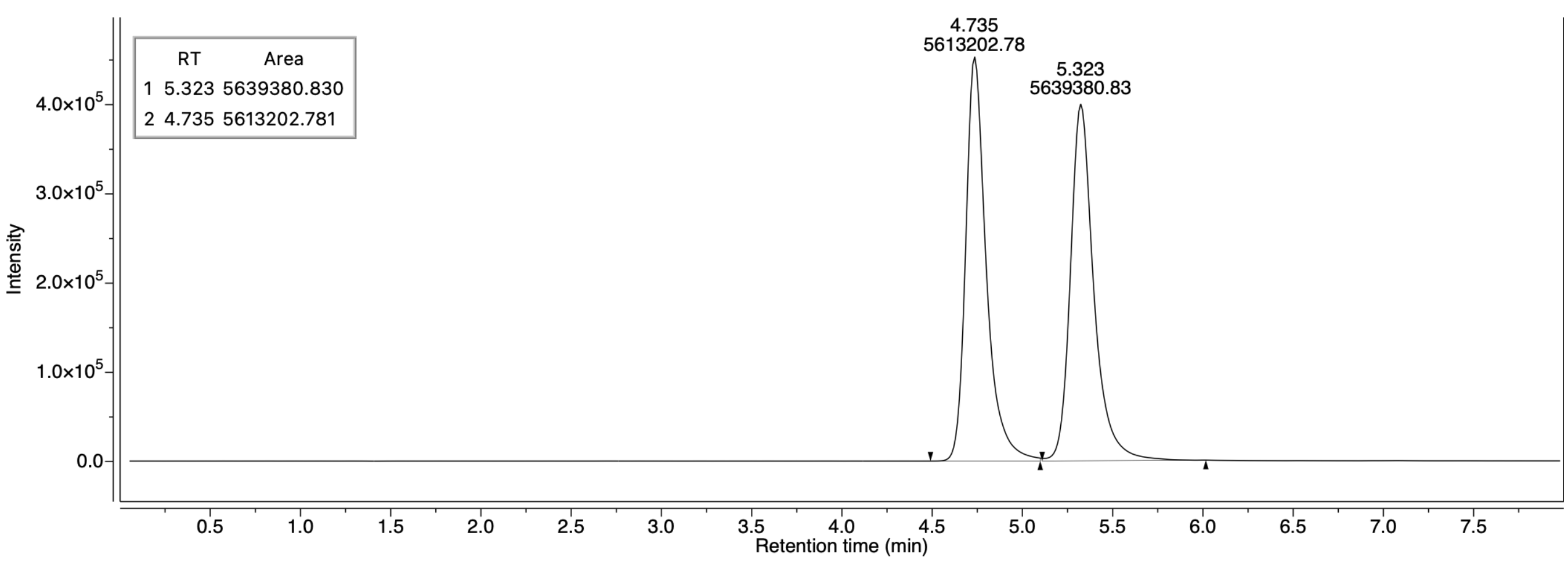

Figure S74: Chiral HPLC trace of racemic $PhCH_2CH_2(OEt)P(O)–CH_2CO_2Et$.

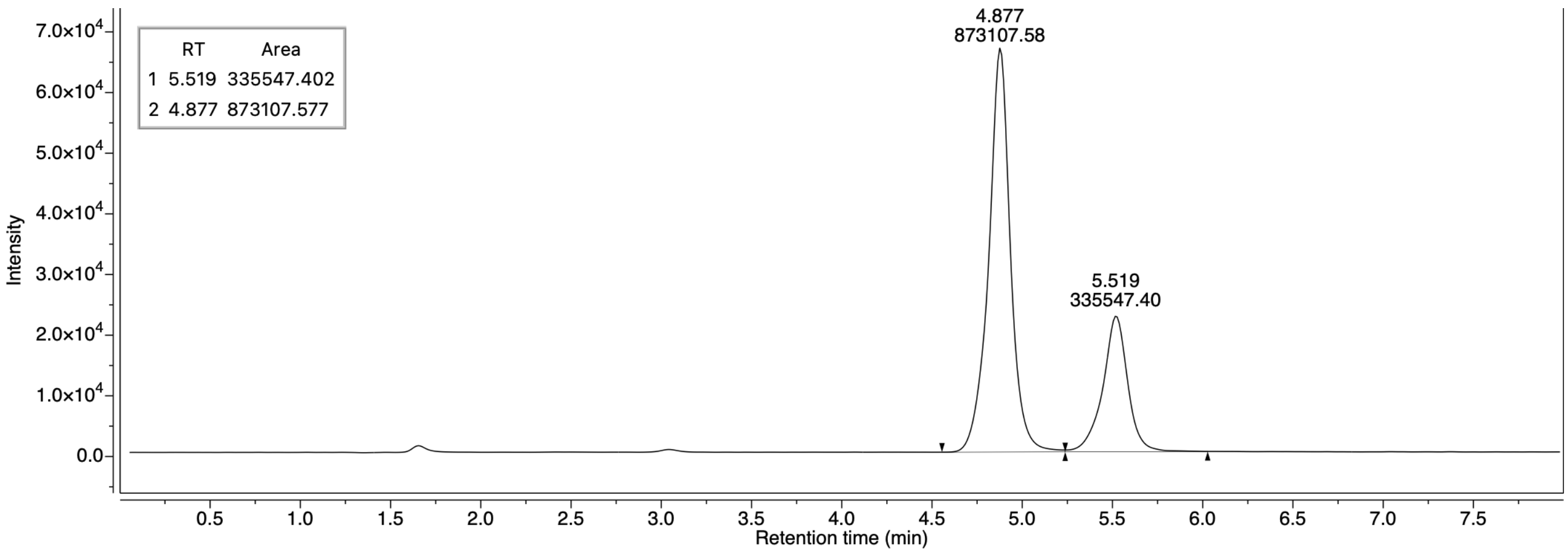

Figure S75: Chiral HPLC trace of reaction catalyzed by G5OEt, $PhCH_2CH_2(OEt)P(O)H$ and EDA.

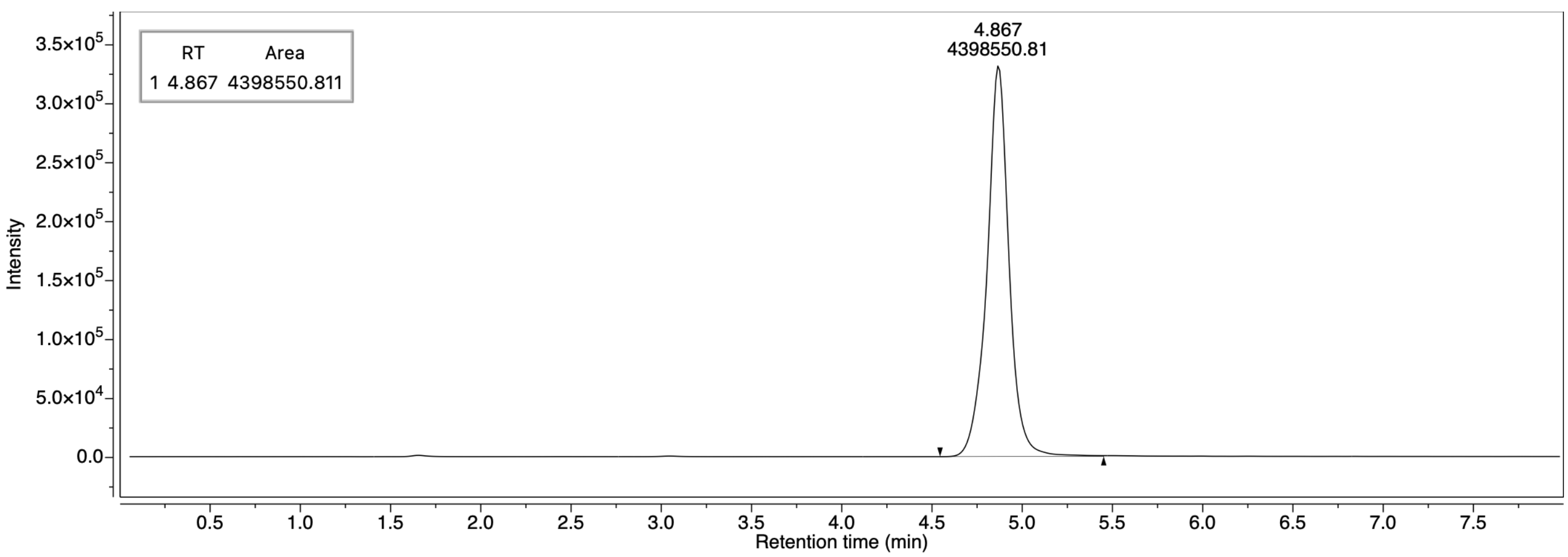

Figure S76: Chiral HPLC trace of reaction catalyzed by G11.1, $PhCH_2CH_2(OEt)P(O)H$ and EDA.

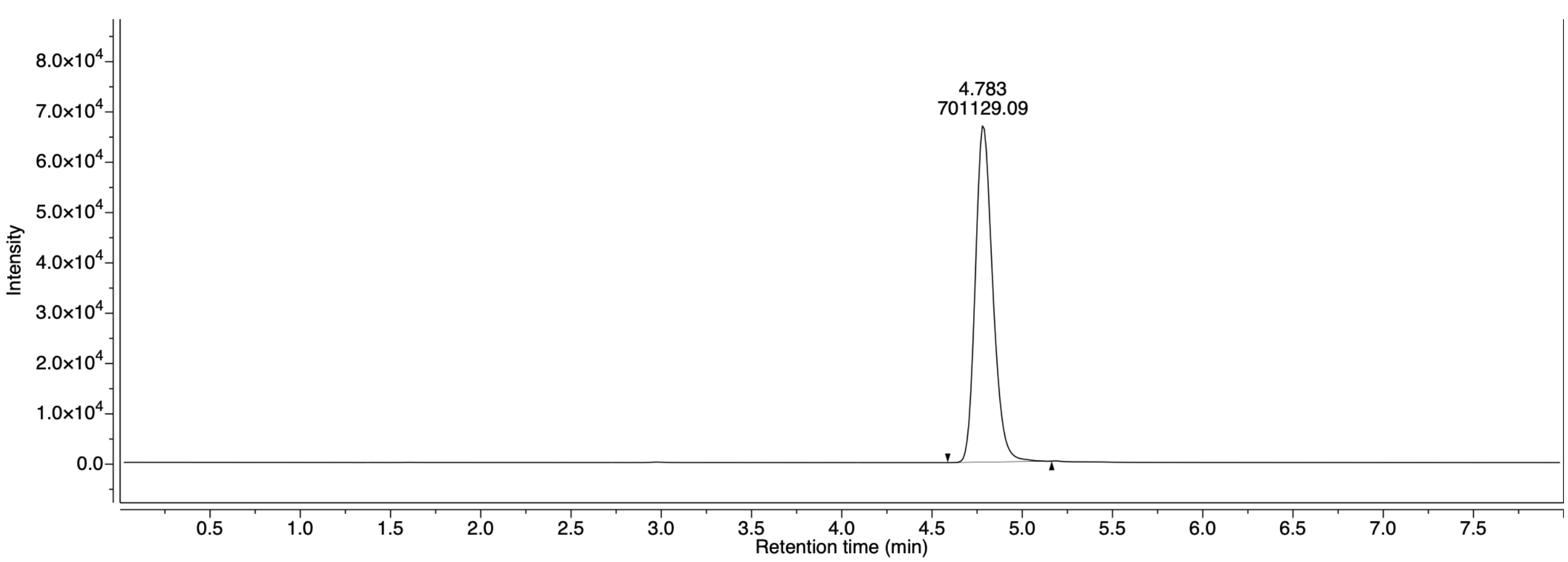

Figure S77: Chiral HPLC trace of large-scale reaction catalyzed by G11.1, $PhCH_2CH_2(OEt)P(O)H$ and EDA.

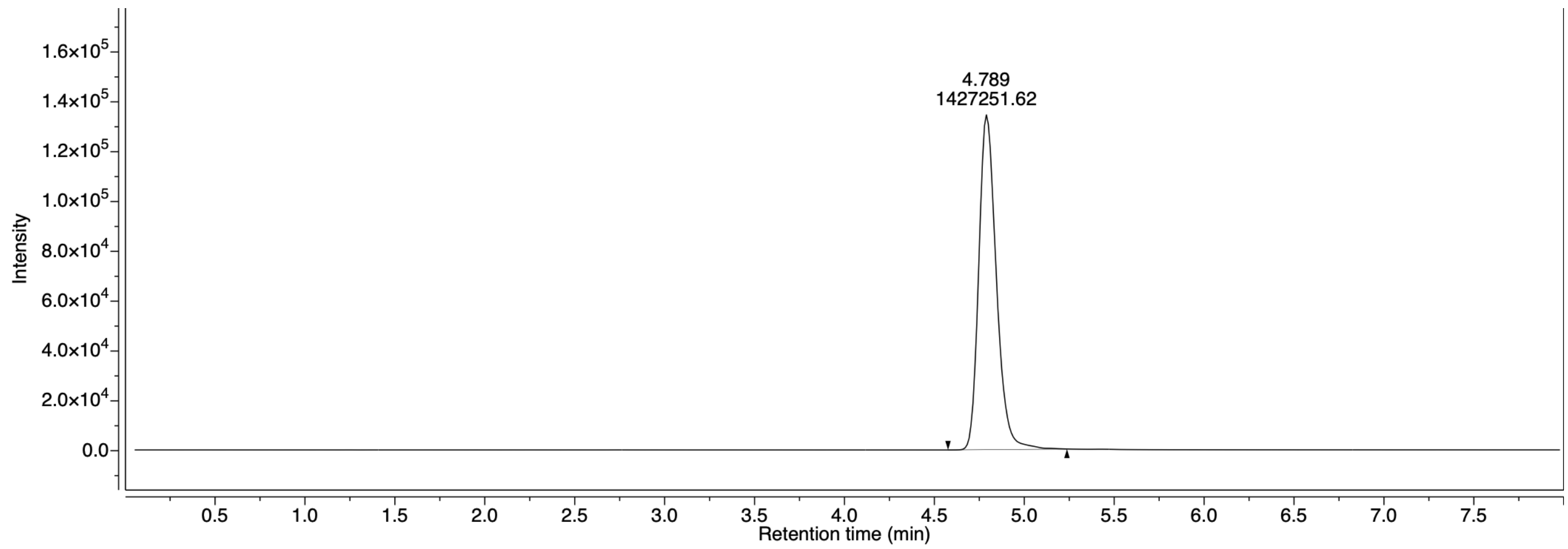

Figure S78: Chiral HPLC trace of purified product from large scale reaction catalyzed by G11.1, $PhCH_2CH_2(OEt)P(O)H$ and EDA.

7.13 ^{i}Bu(OEt)P(O)–CH_2CO_2Et (13b)

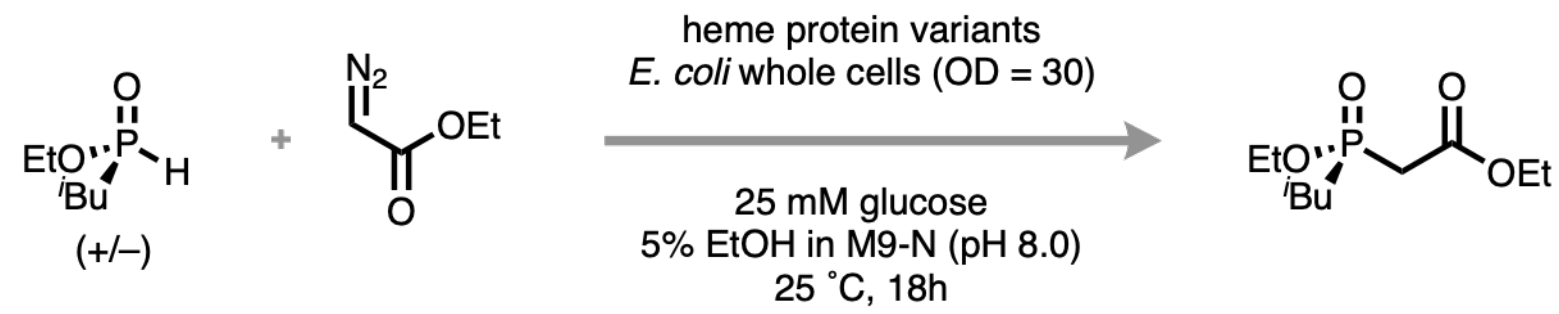

^{i}Bu(OEt)P(O)H and EDA was screened against the Master Compilation Plate (P11) and four variants were chosen with which to perform validation with G5OEt, G5OEt-A59L, G5OEt-A59T, and G11.1. Absolute configuration of product assigned based on comparison to the reactivity of selected variants with other H-phosphinates substrates; specifically the favored reactivity with G5OEt variants.

Reactions were performed according to the validation procedure (three technical replicates for assay yield, one technical replicate for enantioselectivity).

Table S47: Yield and enantioselectivity of variants and selected reaction conditions for the reactions of ^{i}Bu(OEt)P(O)H and EDA.

Variant/conditions	Avg. Yield (%)	Std Dev Yield (%)	Avg. E.E. (%)
G5OEt	17	1	87
G5OEt-A59L	15	< 1	90
G5OEt-A59T	22	< 1	93
G11.1	1	< 1	19

Reverse Phase HPLC-MS: Agilent Poroshell 120 EC-C18, 1.5 mL/min, [A] 0.1% AcOH in Water, [B] 0.1% AcOH in Acetonitrile, scan and single ion monitoring $[M+H]^+$, gradient: 0-0.2 min: 5-50% B; 0.2-0.4 min: 5–20% B; 0.4–2.5 min: 20–50% B; 2.5–2.6 min: 50–95% B; 2.6–3.0 min: 95% B; 3.0–3.1 min: 95–5% B; 3.1–3.5 min: 5% B.

Chiral SFC: (IH-3, 2.5 mL/min, 10% IPA in CO_2, MSD): t_R (major) = 3.4 min, t_R (minor) = 4.6 min.

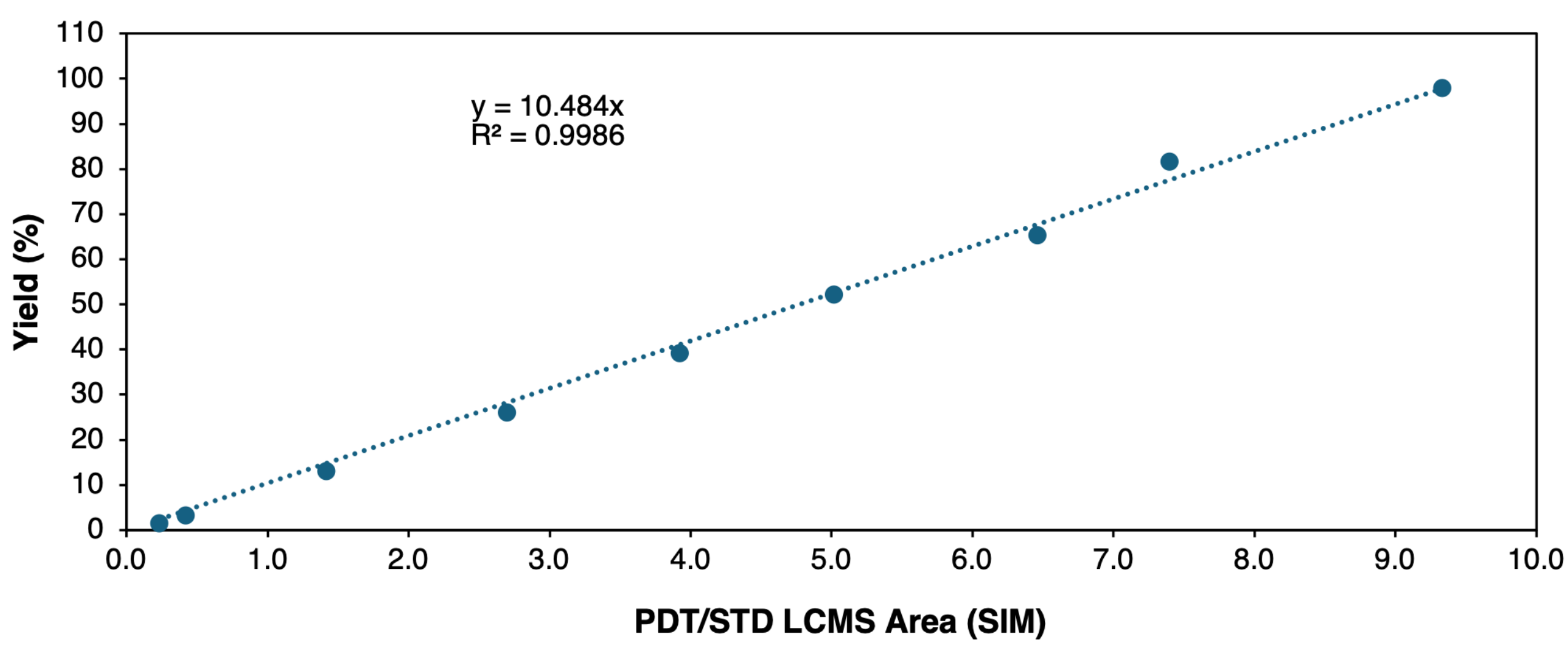

Figure S79: Calibration curve using authentic sample and papaverine as an external standard.

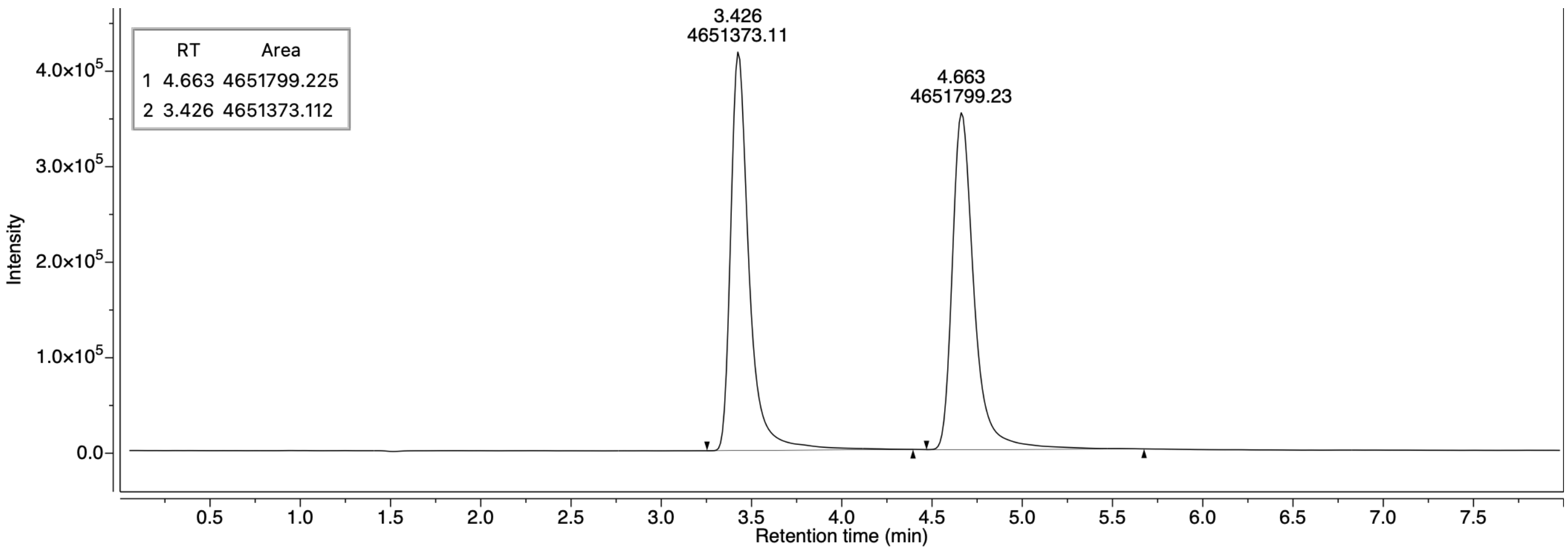

Figure S80: Chiral HPLC trace of racemic ^{i}Bu(OEt)P(O)–CH_2CO_2Et.

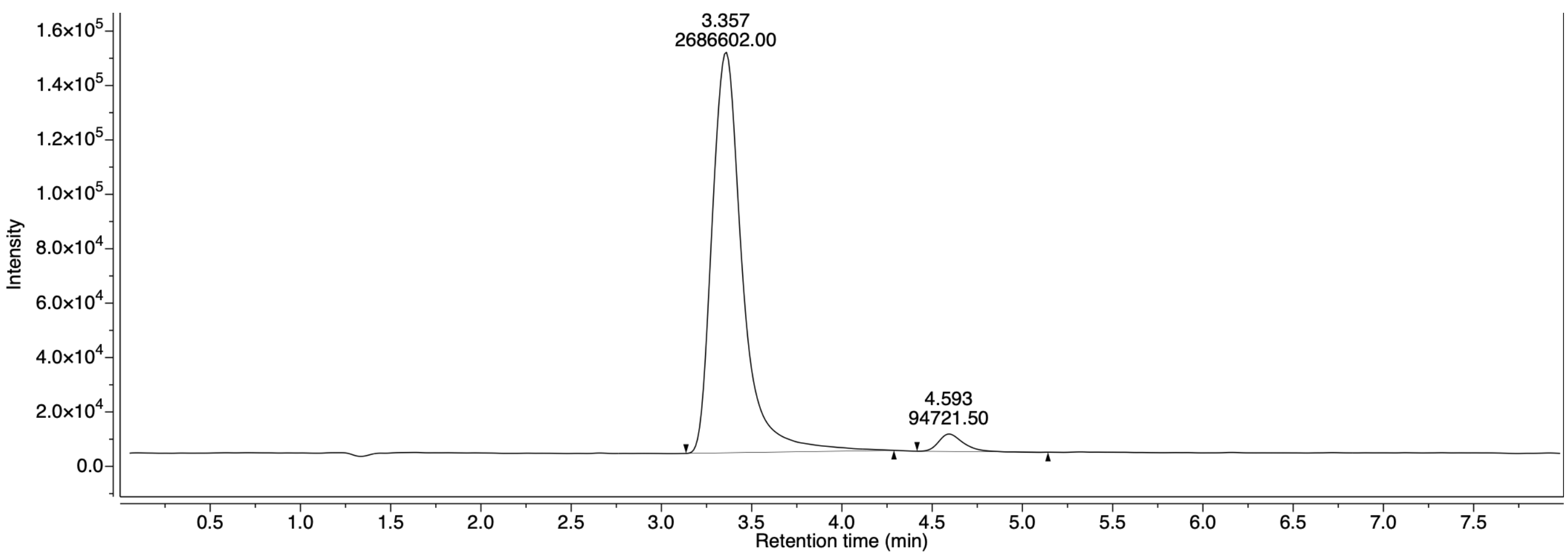

Figure S81: Chiral HPLC trace of reaction catalyzed by G5OEt-A59T, ^{i}Bu(OEt)P(O)H and EDA.

7.14 Ph(OEt)P(O)–CH_2CN (2d)

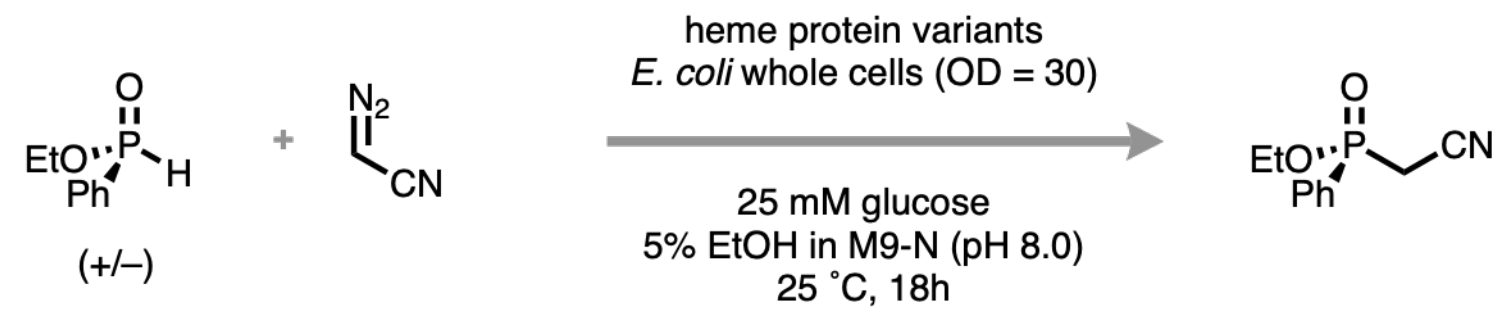

Ph(OEt)P(O)H and DAN was screened against the Master Compilation Plate (P11) and four variants were chosen with which to perform validation with: G5OEt, G5OEt-A59L, and G6.1. Absolute configuration of product assigned based on comparison to the reactivity of selected variants with other H-phosphinates substrates; specifically the favored reactivity with G5OEt variants.

Reactions were performed according to the validation procedure (three technical replicates for assay yield, two technical replicates for enantioselectivity).

Table S48: Yield and enantioselectivity of variants and selected reaction conditions for the reactions of Ph(OEt)P(O)H and DAN.

Variant/conditions	Avg. Yield (%)	Std Dev Yield (%)	Avg. E.E. (%)	Std Dev E.E. (%)
G5OEt	3	1	>99	<1
G5OEt-A59L	8	3	>99	<1
G6.1	4	0	91	<1

Reverse Phase HPLC-MS: Agilent Poroshell 120 EC-C18, 1.5 mL/min, [A] 0.1% AcOH in Water, [B] 0.1% AcOH in Acetonitrile, scan and single ion monitoring $[M+H]^+$, gradient: 0-0.2 min: 5-50% B; 0.2-0.4 min: 5–20% B; 0.4–2.5 min: 20–50% B; 2.5–2.6 min: 50–95% B; 2.6–3.0 min: 95% B; 3.0–3.1 min: 95–5% B; 3.1–3.5 min: 5% B.

Chiral HPLC: Chiralpak IC (Hex/EtOH = 75:25, 1 mL/min).

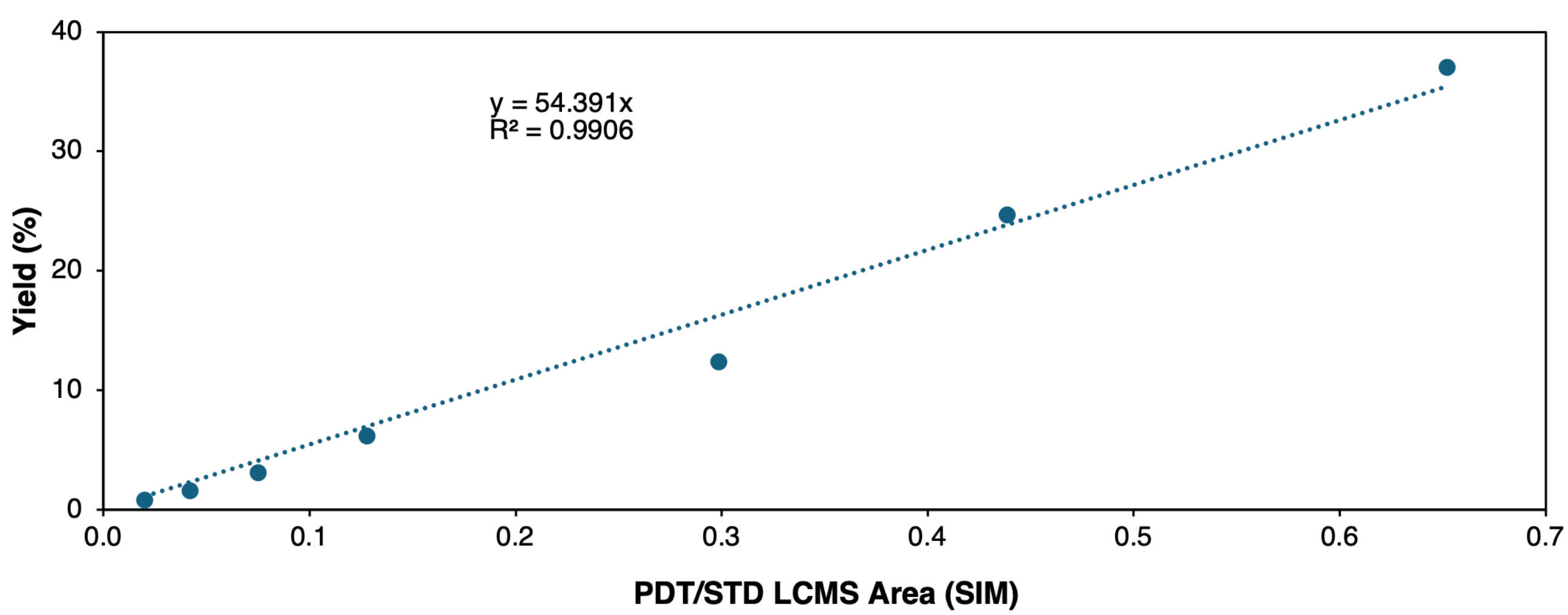

Figure S82: Calibration curve using authentic sample and papaverine as an external standard.

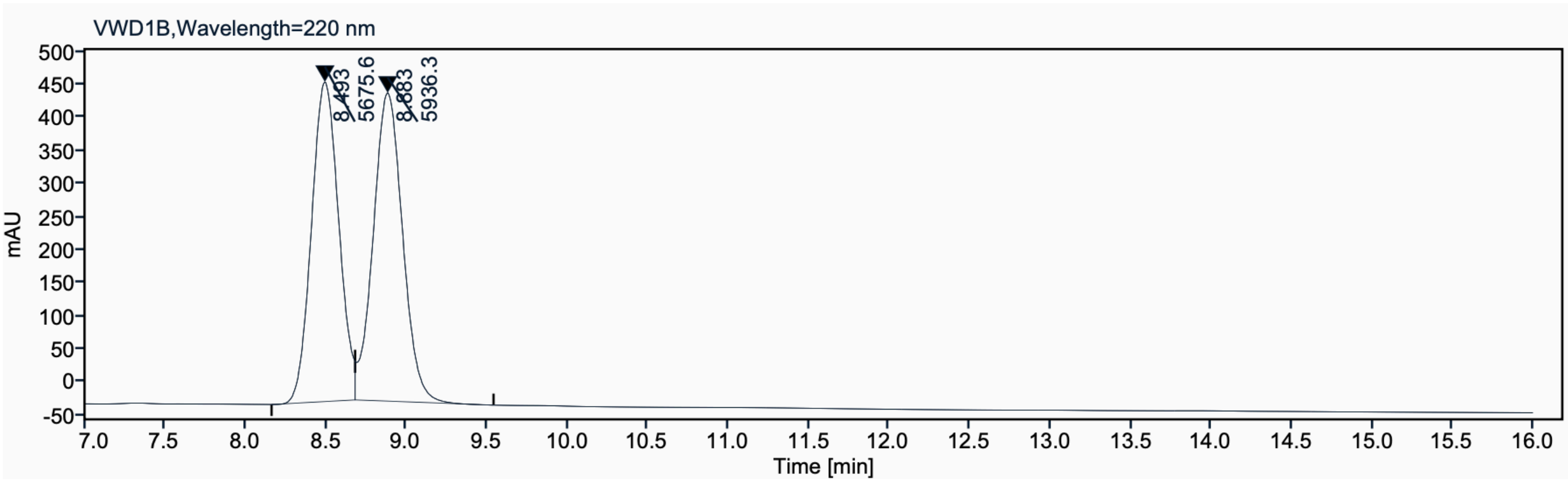

Figure S83: Chiral HPLC trace of racemic Ph(OEt)P(O)H.

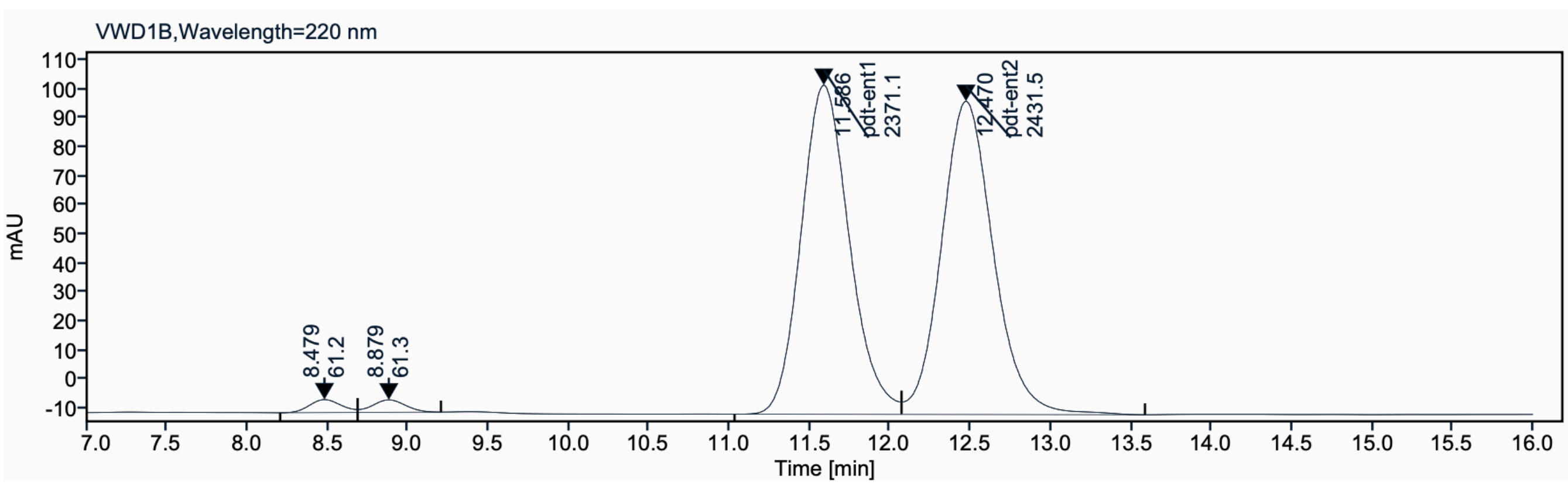

Name	RT [min]	Area
pdt-ent1	11.586	2371.0562
pdt-ent2	12.470	2431.4502

Figure S84: Chiral HPLC trace of racemic Ph(OEt)P(O)–CH_2CN.

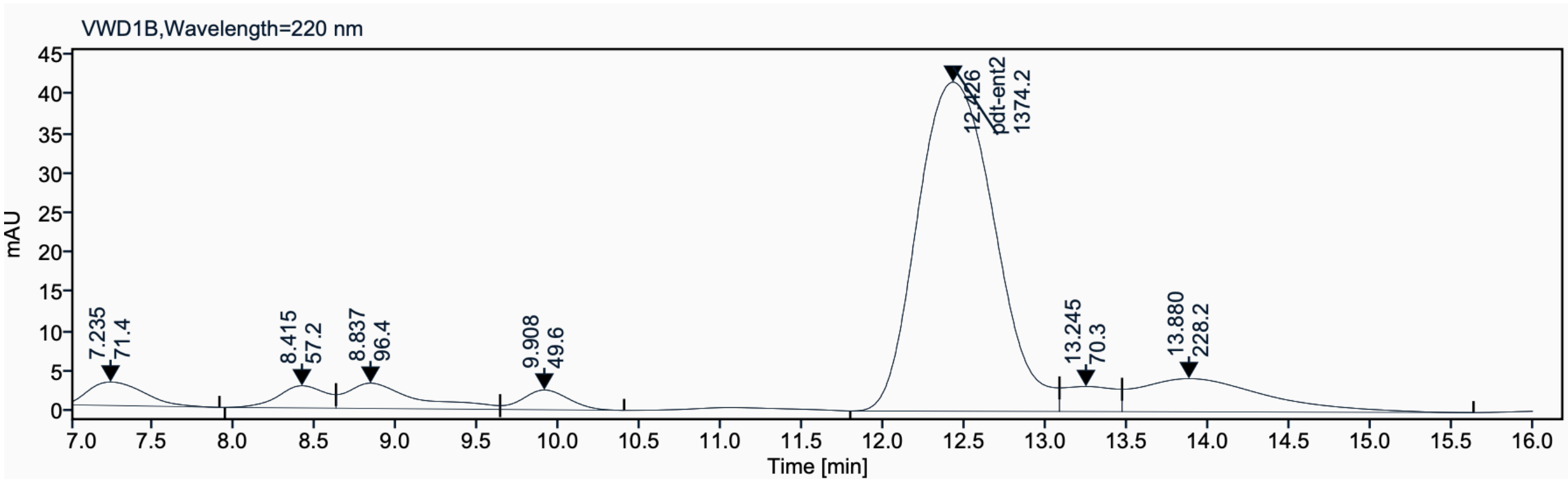

Name	RT [min]	Area
pdt-ent2	12.426	1374.2060

Figure S85: Chiral HPLC trace of reaction catalyzed by G5OEt-A59L, Ph(OEt)P(O)H and DAN.

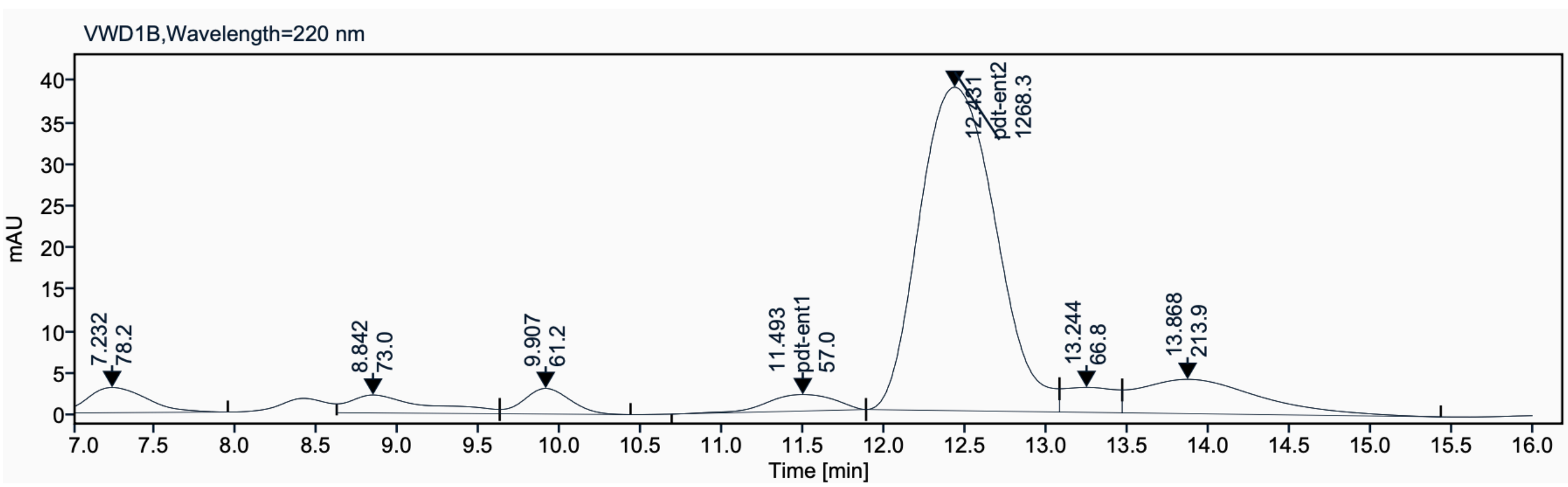

Name	RT [min]	Area
pdt-ent1	11.493	56.9808
pdt-ent2	12.431	1268.3433

Figure S86: Chiral HPLC trace of reaction catalyzed by G6.1, Ph(OEt)P(O)H and DAN.

7.15 Ph(OEt)P(O)–alpha butyrolactone (2e)

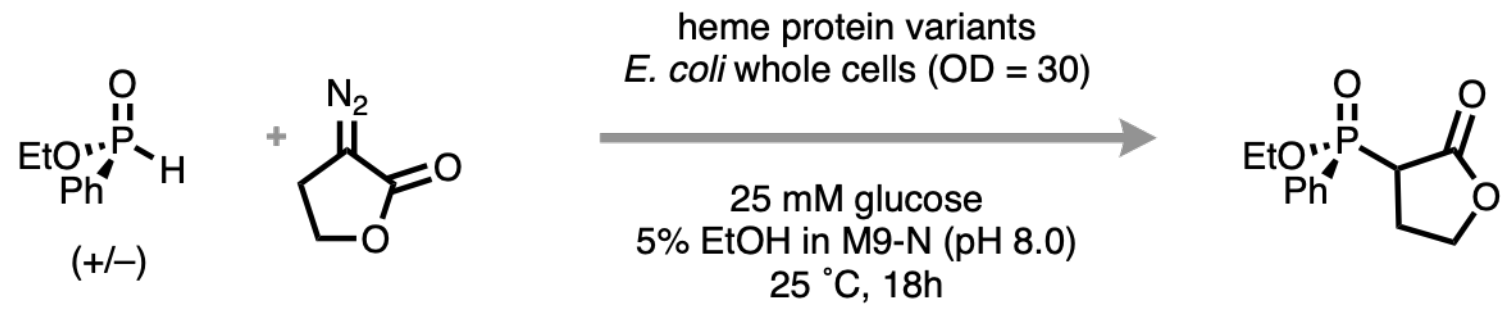

Ph(OEt)P(O)H and LAD was screened against the Master Compilation Plate (P11), and four variants were chosen with which to perform validation with: G5OEt, G5OEt-A59I, and G6.1. Absolute configuration of product assigned based on comparison to the reactivity of selected variants with other H-phosphinates substrates; specifically the favored reactivity with G5OEt variants.

Reactions were performed according to the validation procedure (three technical replicates for assay yield; one technical replicate for enantioselectivity). Once the Chiral SFC was acquired, the SFC samples for G5OEt and G5OEt-A59I were combined and solvent removed *en vacuo*. The residue was dissolved in $CDCl_3$ and a ^{31}P NMR was acquired to determine if the reaction was > 97% ee with 1:1 dr or 0% ee with 98:2 dr. The ^{31}P NMR had peaks corresponding to both diasteromers in ~1:1 ratio, thus revealing the reaction is enantioselective with poor diastereoselectivity. Due to all other reactions displaying no racemization at P(V), this is likely due to racemization at the acidic postion alpha to P(V) and the ester.

Table S49: Yield and enantioselectivity of variants and selected reaction conditions for the reactions of Ph(OEt)P(O)H and LAD.

Variant/conditions	Avg. Yield (%)	Std Dev Yield (%)	Avg. E.E. (%)
G5OEt	6	2	97
G5OEt-A59I	14	1	98
G6.1	< 1	< 1	N.D.

Reverse Phase HPLC-MS: Agilent Poroshell 120 EC-C18, 1.5 mL/min, [A] 0.1% AcOH in Water, [B] 0.1% AcOH in Acetonitrile, scan and single ion monitoring $[M+H]^+$, gradient: 0-0.2 min: 5-50% B; 0.2-0.4 min: 5–20% B; 0.4–2.5 min: 20–50% B; 2.5–2.6 min: 50–95% B; 2.6–3.0 min: 95% B; 3.0–3.1 min: 95–5% B; 3.1–3.5 min: 5% B.

Chiral SFC: (IH-3, 2.5 mL/min, 5% MeOH in CO_2, MSD): t_R (major) = 5.7 min, t_R (major) = 7.5 min, t_R (minor) = 8.7 min, t_R (minor) = 10.1 min.

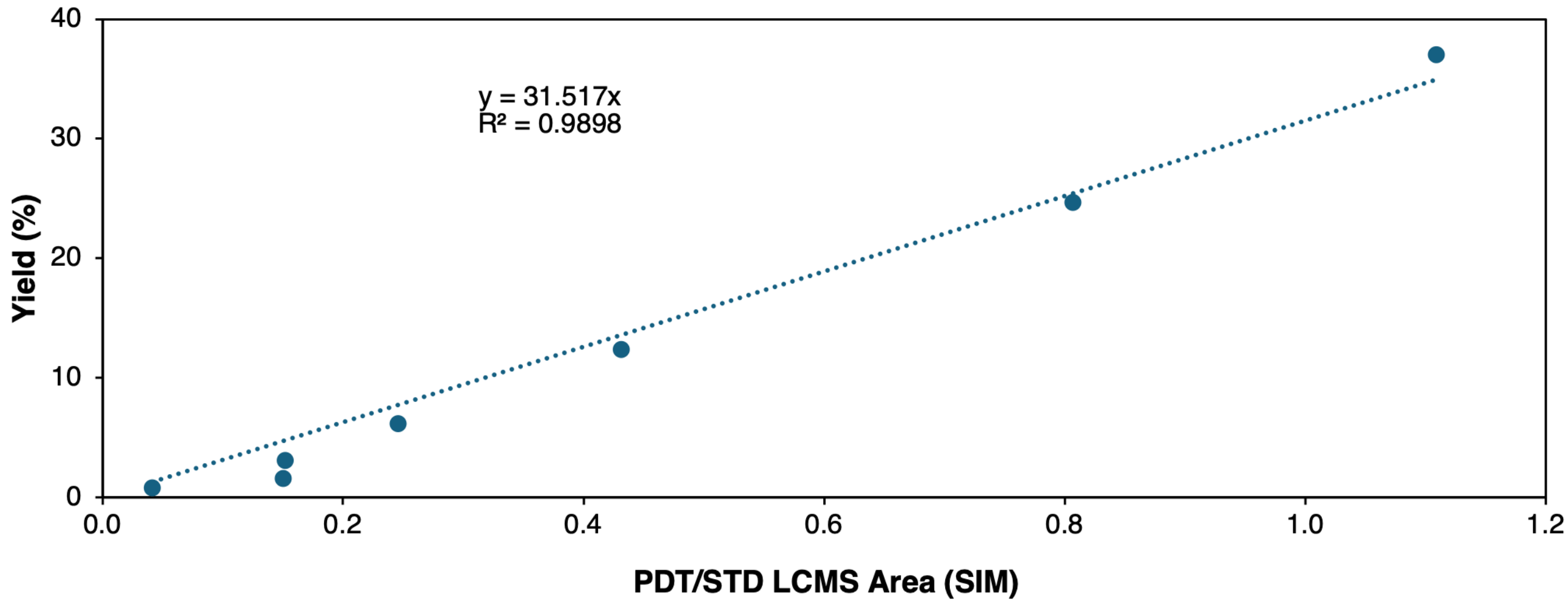

Figure S87: Calibration curve using authentic sample and papaverine as an external standard.

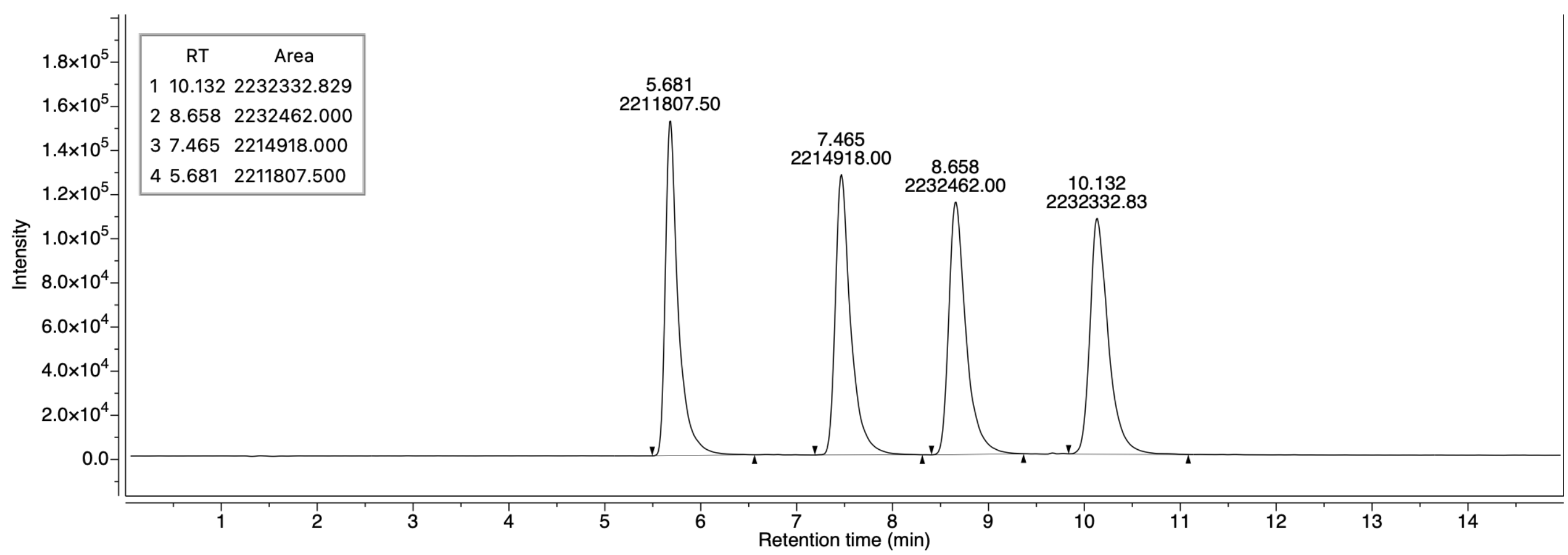

Figure S88: Chiral HPLC trace of racemic Ph(OEt)P(O)–alpha butyrolactone.

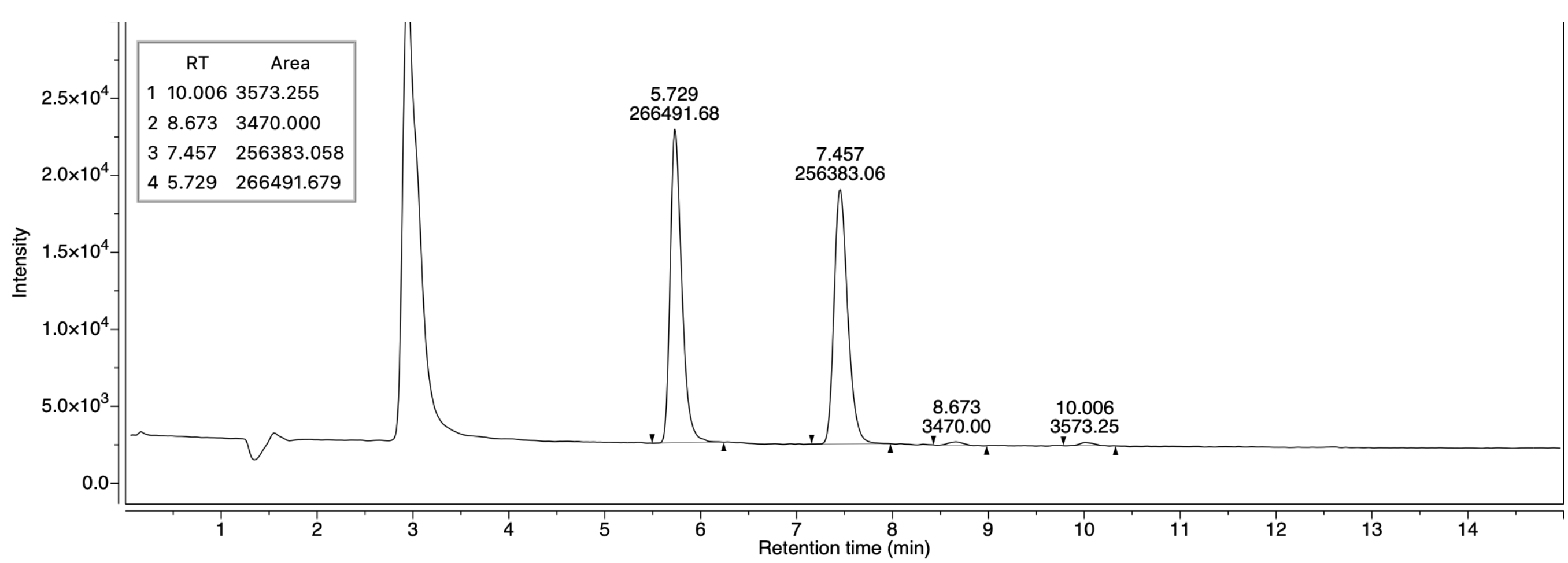

Figure S89: Chiral HPLC trace of reaction catalyzed by G5OEt, Ph(OEt)P(O)H and LAD.

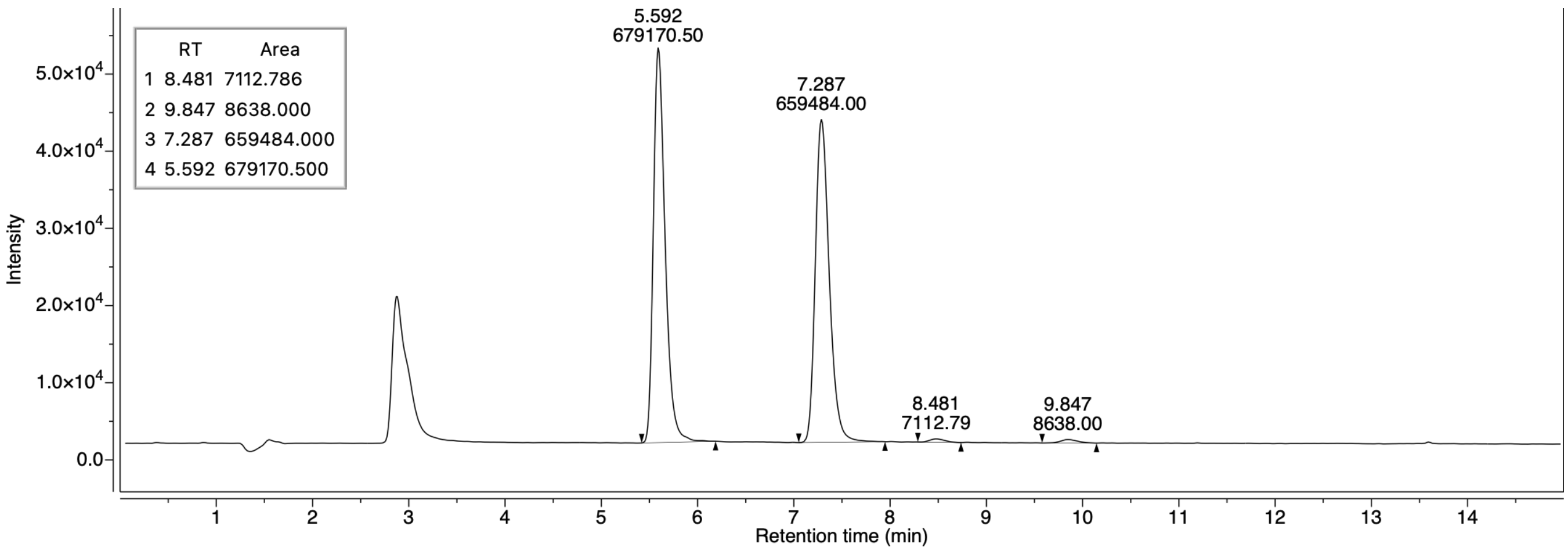

Figure S90: Chiral HPLC trace of reaction catalyzed by G5OEt-A59I, Ph(OEt)P(O)H and LAD.

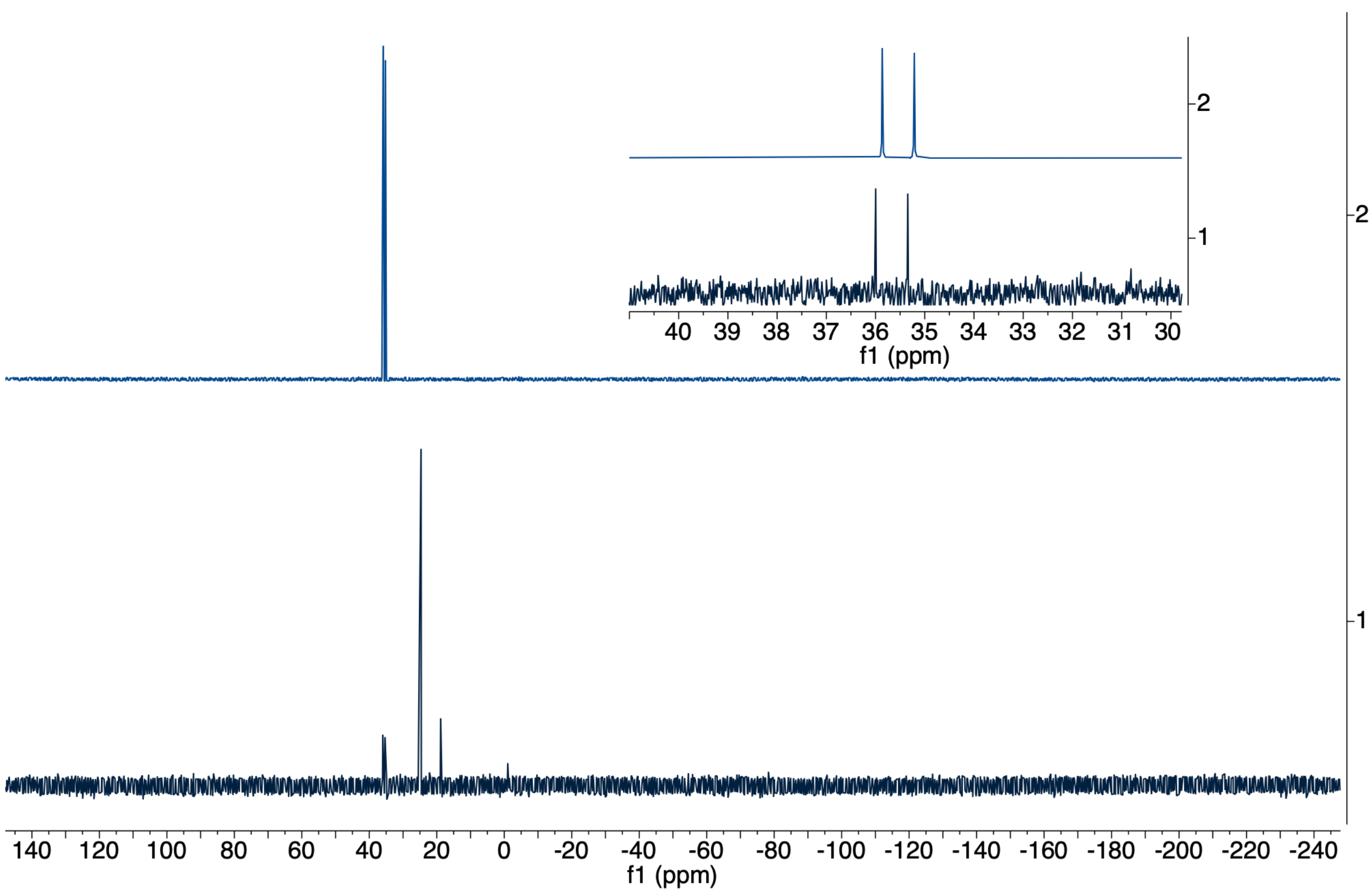

Figure S91: ^{31}P stack plot of authentic Ph(OEt)P(O)–alpha butyrolactone (2) and the combined reactions catalyzed by G5OEt and G5OEt-A59I, Ph(OEt)P(O)H and LAD (1).

7.16 Ph(OEt)P(O)–NH2 (2f)

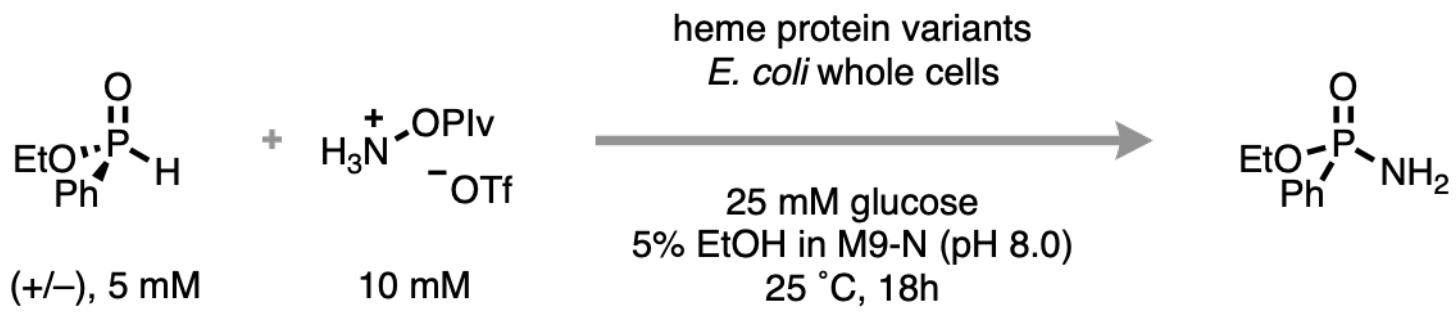

Reactions were performed according to the validation procedure, see Section 5.3 for assay yields and enantioselectivity across the whole lineage. Absolute configuration of product assigned based on comparison to the reactivity of selected variants with other H-phosphinates substrates; specifically the lineage beginning from G5OEt which favored the consumption of **P_R-2a** and subsequent mutation at site 145 leading to favored consumption of **P_S-2a**.

Chiral HPLC: Chiralpak IA (Hex/EtOH = 95:05, 1 mL/min).

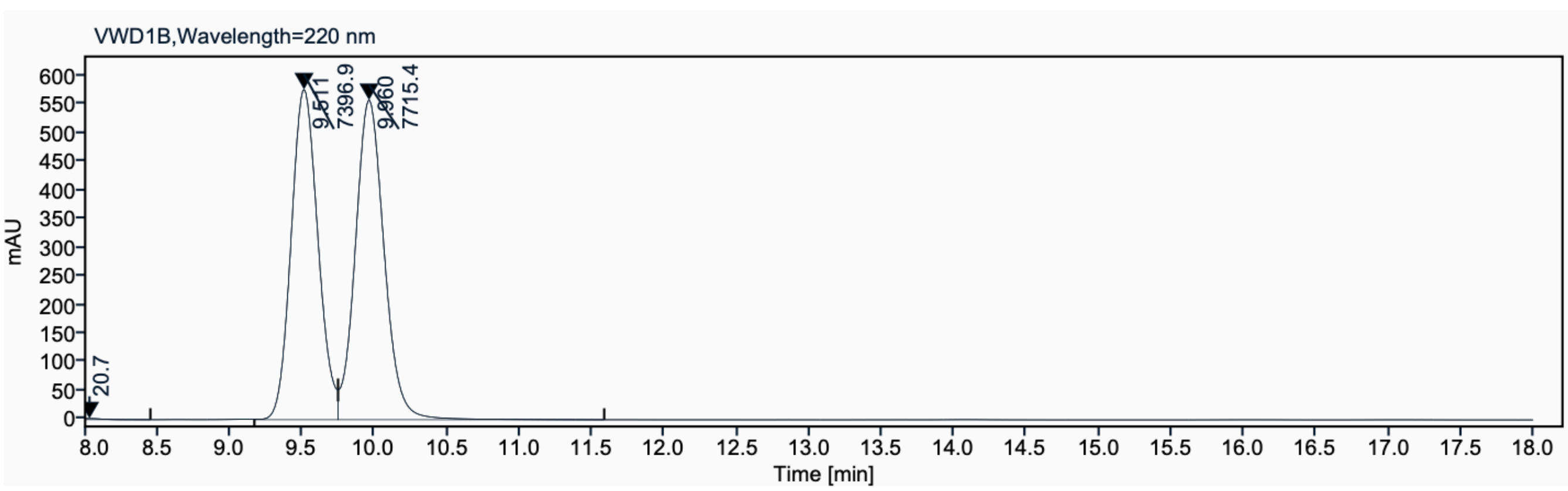

Name	RT [min]	Area

Figure S92: Chiral HPLC trace of racemic Ph(OEt)P(O)H.

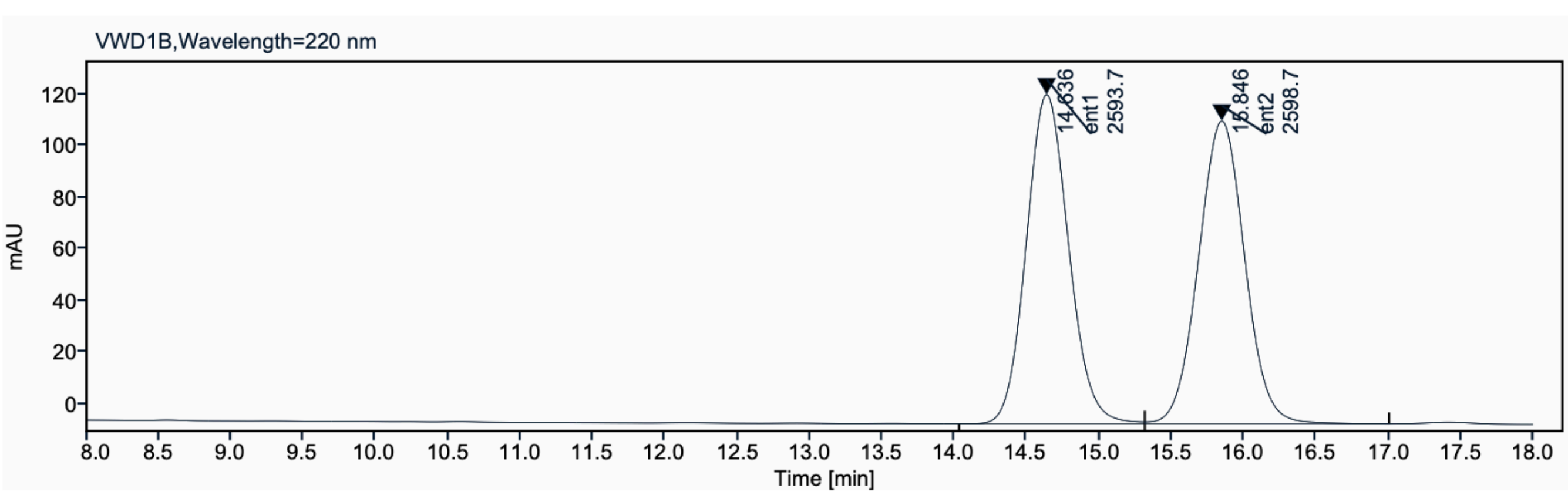

Name	RT [min]	Area
ent1	14.636	2593.6594
ent2	15.846	2598.6866

Figure S93: Chiral HPLC trace of racemic Ph(OEt)P(O)–NH_2.

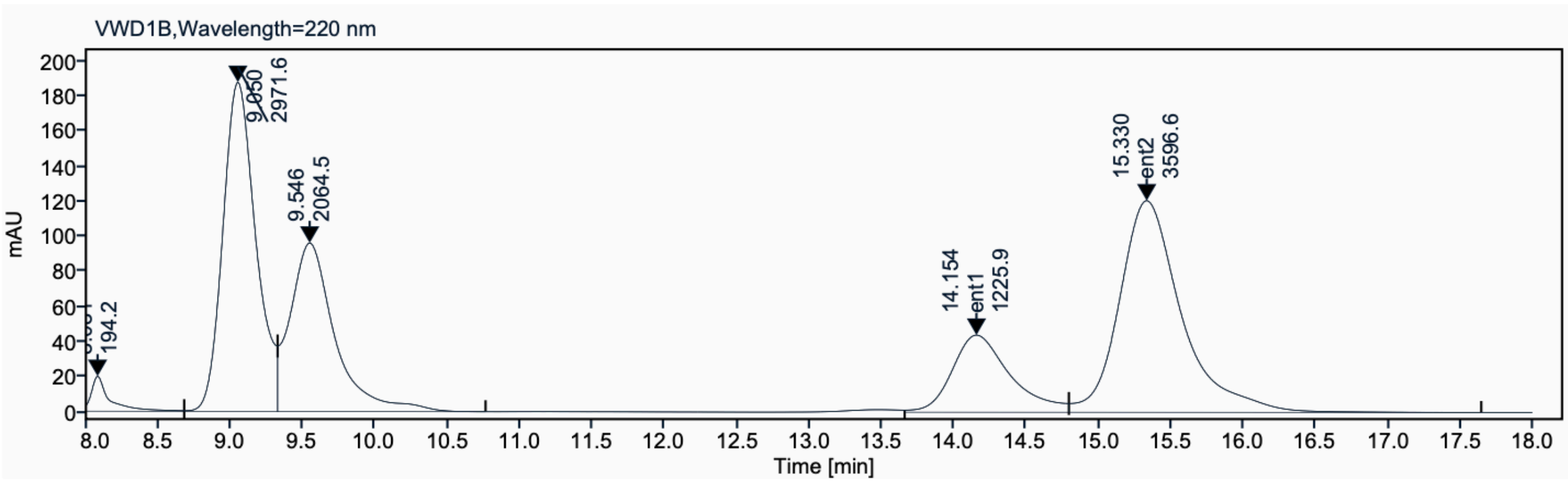

Name	RT [min]	Area
ent1	14.154	1225.8548
ent2	15.330	3596.6358

Figure S94: Chiral HPLC trace of reaction catalyzed by G6N, Ph(OEt)P(O)H and PONT.

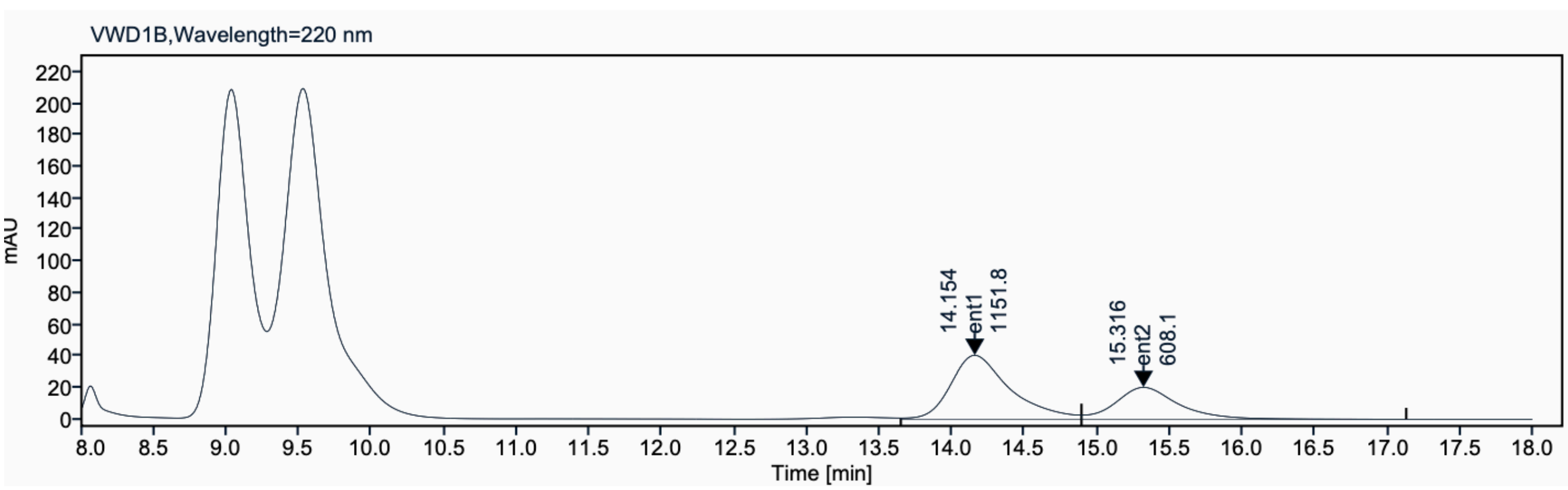

Name	RT [min]	Area
ent1	14.154	1151.7789
ent2	15.316	608.0925

Figure S95: Chiral HPLC trace of reaction catalyzed by G6N.1, Ph(OEt)P(O)H and PONT.

7.17 Ph^{i}PrP(O)–NH$_2$ (4c)

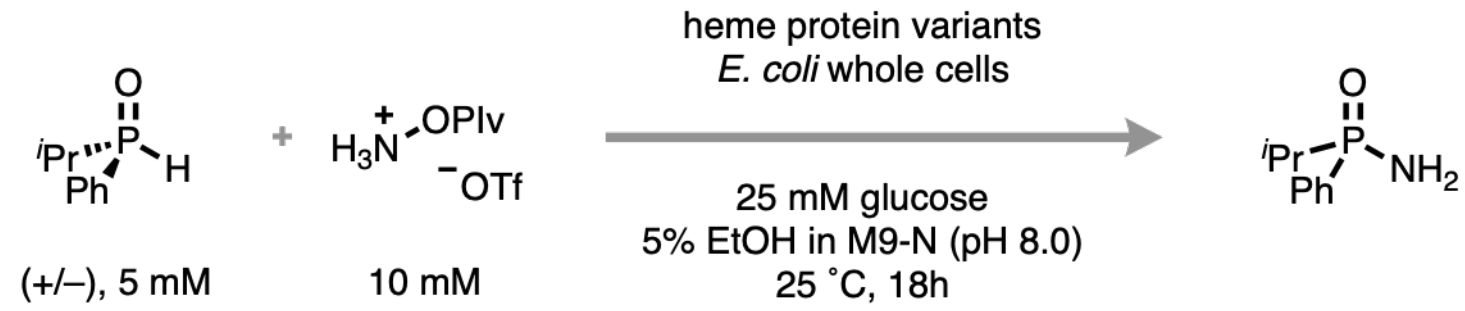

Validation of Ph^{i}PrP(O)H and PONT were performed with G6N. Absolute configuration of product assigned based on comparison to the reactivity of selected variants with other H-phosphinates substrates; specifically the lineage beginning from G5OEt which favored the consumption of **P$_R$-2a** and subsequent mutation at site 145 leading to favored consumption of **P$_S$-2a**.

Reactions were performed according to the validation procedure (three technical replicates for assay yield, one technical replicate for enantioselectivity).

Table S50: Yield and enantioselectivity of variants and selected reaction conditions for the reactions of Ph^{i}PrP(O)H and PONT.

Variant/conditions	Avg. Yield (%)	Std Dev Yield (%)	Avg. E.E. (%)
G7N	34	1	27

Reverse Phase HPLC-MS: Agilent Poroshell 120 EC-C18, 1.5 mL/min, [A] 0.1% AcOH in Water, [B] 0.1% AcOH in Acetonitrile, scan and single ion monitoring $[M+H]^+$, gradient: 0-0.2 min: 5-50% B; 0.2-0.4 min: 5–20% B; 0.4–2.5 min: 20–50% B; 2.5–2.6 min: 50–95% B; 2.6–3.0 min: 95% B; 3.0–3.1 min: 95–5% B; 3.1–3.5 min: 5% B.

Chiral HPLC: Chiralpak IB and IC in series (Hex/EtOH = 85:15, 1 mL/min): t_R (RSM-S$_P$) = 35.3 min, t_R (RSM-R$_P$) = 37.4 min. Using the columns within our inventory, two columns were necessary to resolve the enantiomers of both RSM and product.

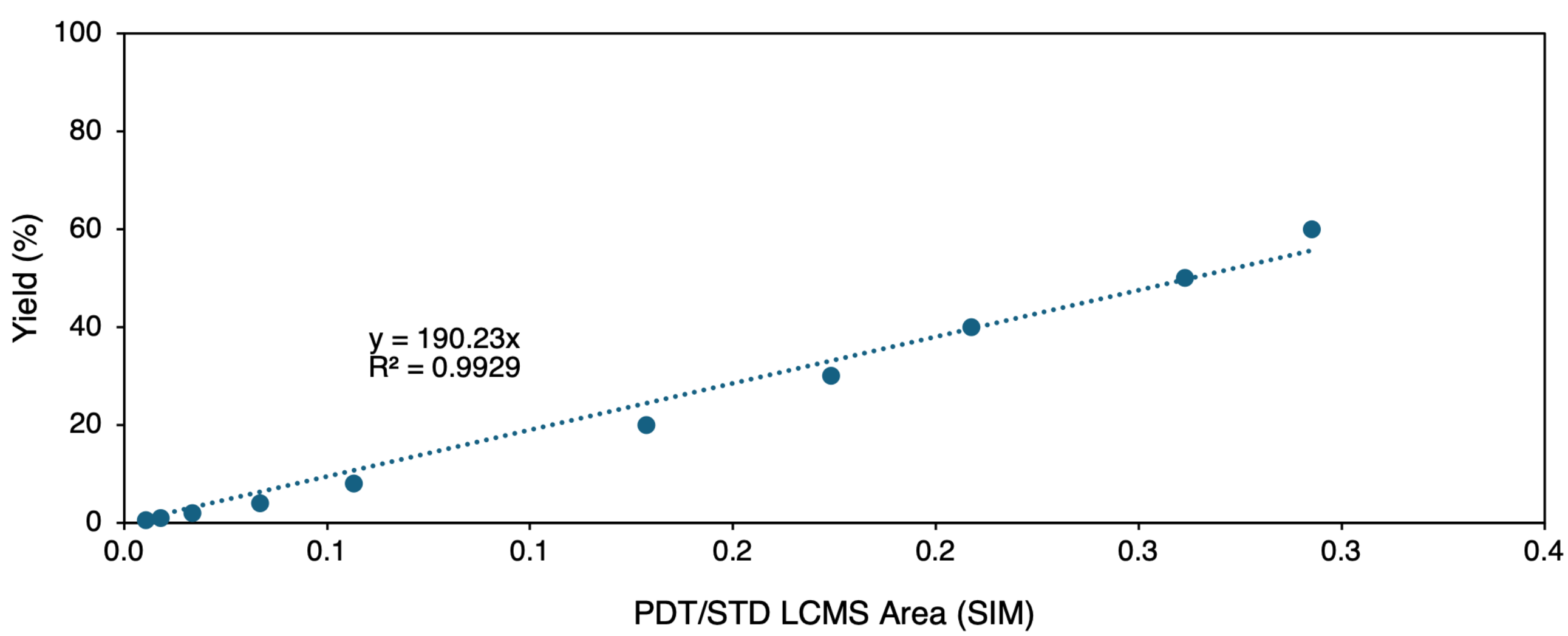

Figure S96: Calibration curve using authentic sample and papaverine as an external standard.

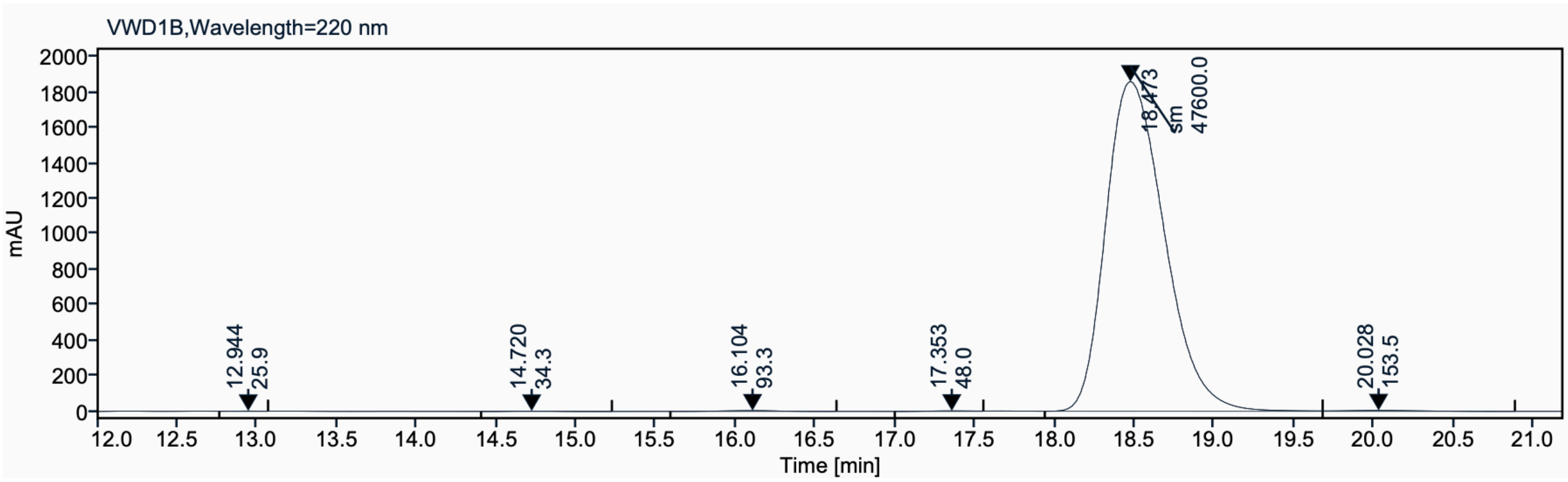

Name	RT [min]	Area
sm	18.473	47599.9706

Figure S97: Chiral HPLC trace of racemic Ph^{i}PrP(O)H.

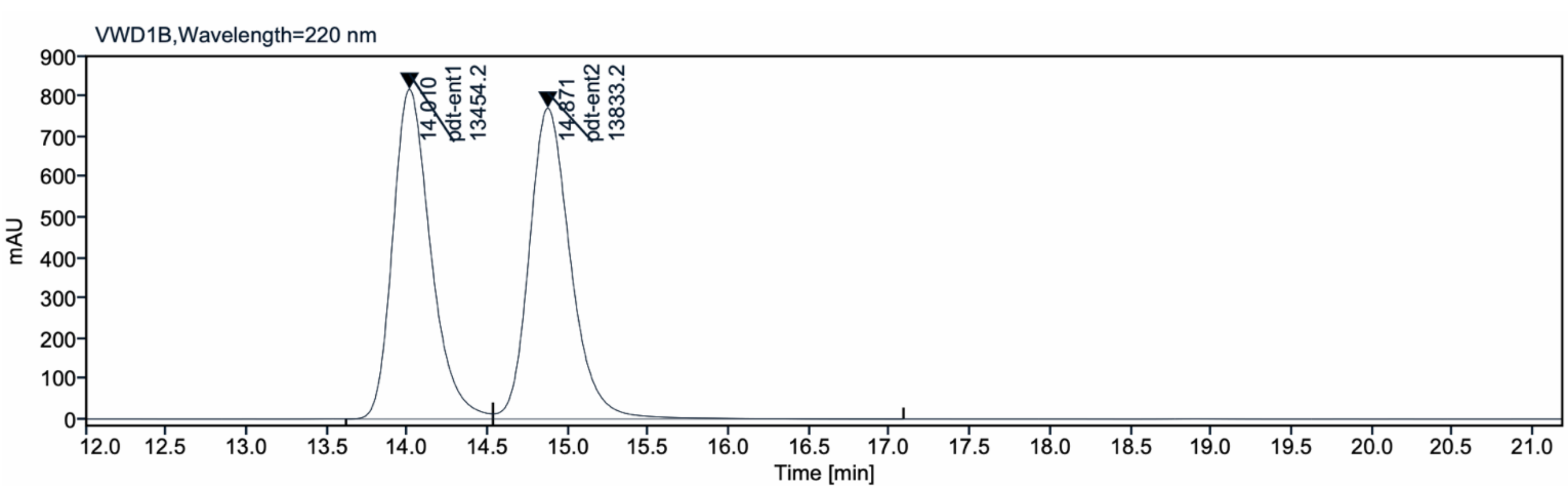

Name	RT [min]	Area
pdt-ent1	14.010	13454.2392
pdt-ent2	14.871	13833.2037

Figure S98: Chiral HPLC trace of racemic Ph^{i}PrP(O)–NH_2.

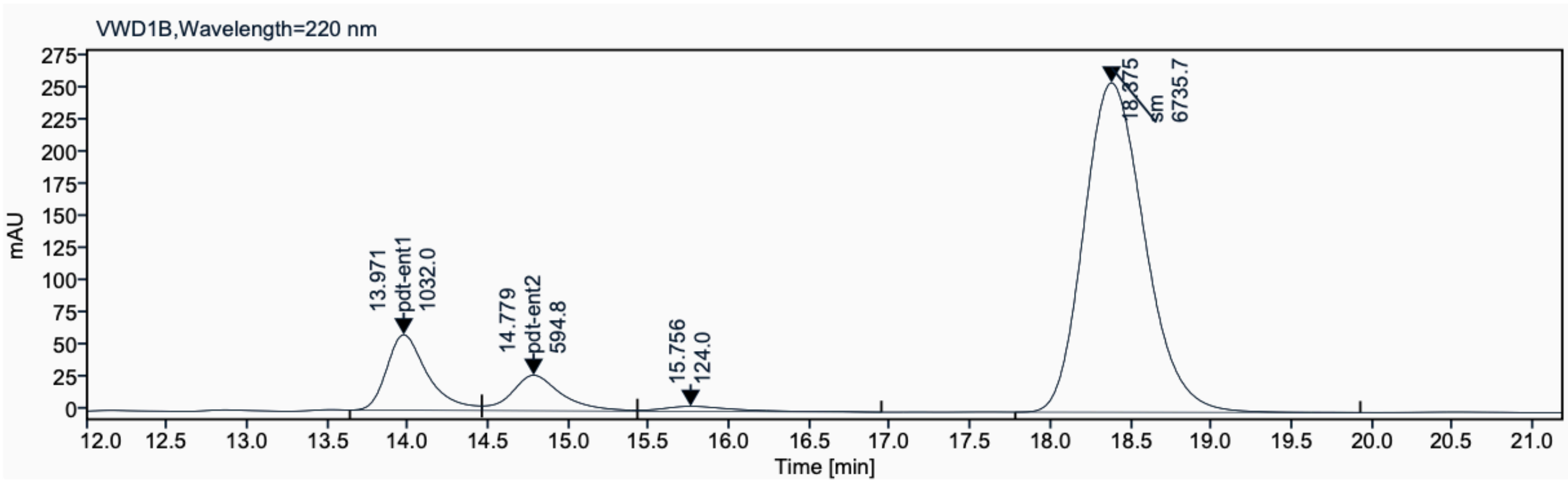

Name	RT [min]	Area
pdt-ent1	13.971	1032.0349
pdt-ent2	14.779	594.8100
sm	18.375	6735.6905

Figure S99: Chiral HPLC trace of reaction catalyzed by G7N, Ph^{i}PrP(O)H and PONT.

8. Mechanistic Studies and Absolute Stereochemistry

8.1 Synthesis and subjection of enantioenriched PhMeP(O)H (1a)

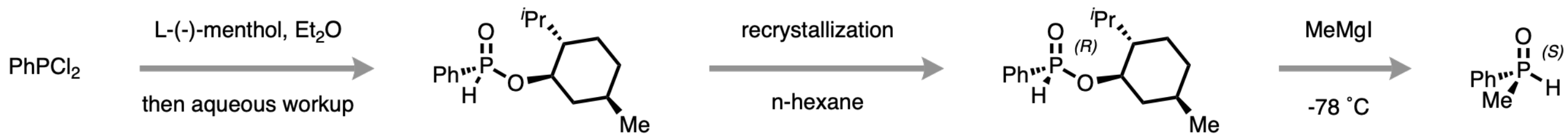

Enantioenriched PhMeP(O)H was prepared from (-)-menthyl phenylphosphinate and (+)-menthyl phenylphosphinate according to previously reported procedures.[28]

(L)-(-)-menthol and (D)-(+)-menthol were reacted with commercially available $PhPCl_2$ in the presence of pyridine in Et_2O. Following an aqueous workup with hexanes, the organic phases were washed with brine, dried over Na_2SO_4, filtered, and concentrated *in vacuo*. In our hands, the recrystallization to provide enantiomeric enriched R_P and S_P menthyl phenyl-phosphinate, respectively, was not as easy as reported. Recrystallization was unsuccessful, so the material was first purified by silica gel chromatography (hexanes/ethyl acetate). The purified material was dissolved in anhydrous n-hexane (0.5 g/mL) and placed in a –20 ˚C freezer for two days, resulting in clear crystals. R_P and S_P menthyl phenyl-phosphinate were determined to be 89:11 and 7:93 R_P/R_S, respectively. All characterization data agreed with previously reported values.

^{31}P NMR: (162 MHz, $CDCl_3$) δ 24.7 (major diastereomer), 21.3 (minor diastereomer).

The enriched menthyl phenyl-phosphinates were reacted with 2.1 equiv of MeMgI in THF at –78 ˚C. The reaction was quenched with saturated NH_4Cl and allowed to warm to room temperature. The mixture was extracted with hexanes and then three time with DCM. The combined DCM extractions were washed with brine, dried over Na_2SO_4, filtered, and concentrated *in vacuo*. The crude product was purified by silica gel chromatography (ethyl acetate/methanol) to afford the desired enantioenriched PhMeP(O)H. R_P and S_P menthyl phenyl-phosphinate afforded S_P and R_P methyl(phenyl)phosphine oxide. All characterization data agreed with previously reported values and matched the authentic standard preprepared in Section 3.1.

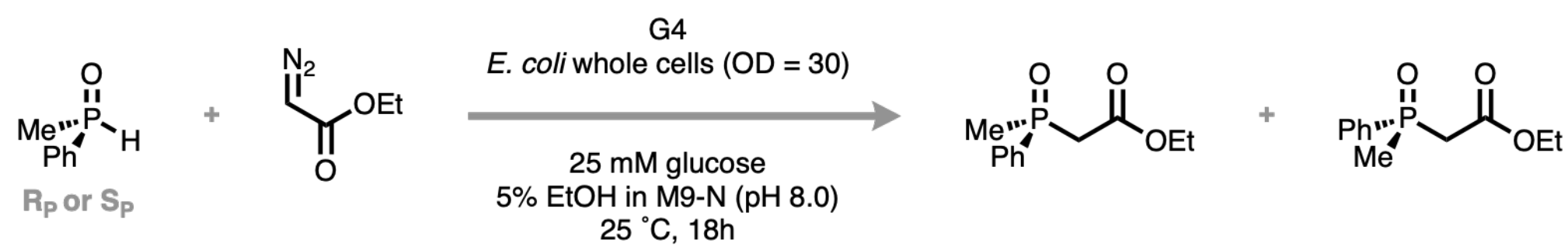

Enzymatic reactions were performed according to the validation procedure with enzyme variant G4 in whole-cell conditions (two technical replicates for assay yield; one technical replicate for enantioselectivity).

Table S51: Yield and enantioselectivity of G4 and selected reaction conditions for the reactions of PhMeP(O)H and EDA.

Variant/conditions	Avg. Yield (%)	Std Dev Yield (%)	Avg. E.E. (%)	RSM Avg. E.E. (%)
G4–S_P	19	1	86	98
G4–R_P	75	5	99	50

From this data, the reaction with G4 selectively consumes R_P methyl(phenyl)phosphine oxide.

Reverse Phase HPLC-MS: Agilent Poroshell 120 EC-C18, 1.5 mL/min, [A] 0.1% AcOH in Water, [B] 0.1% AcOH in Acetonitrile, scan and single ion monitoring $[M+H]^+$, gradient: 0–0.2 min: 5–50% B; 0.2–0.4 min: 5–20% B; 0.4–2.5 min: 20–50% B; 2.5–2.6 min: 50–95% B; 2.6–3.0 min: 95% B; 3.0–3.1 min: 95–5% B; 3.1–3.5 min: 5% B.

Chiral HPLC: Chiralpak IB and IC in series (Hex/EtOH = 85:15, 1 mL/min): t_R (RSM-S_P) = 35.3 min, t_R (RSM-R_P) = 37.4 min. Using the columns within our inventory, two columns were necessary to resolve the enantiomers of both RSM and product (Chiralpak IC is sufficient to resolve the enantiomers of product, but the peaks overlap with RSM).

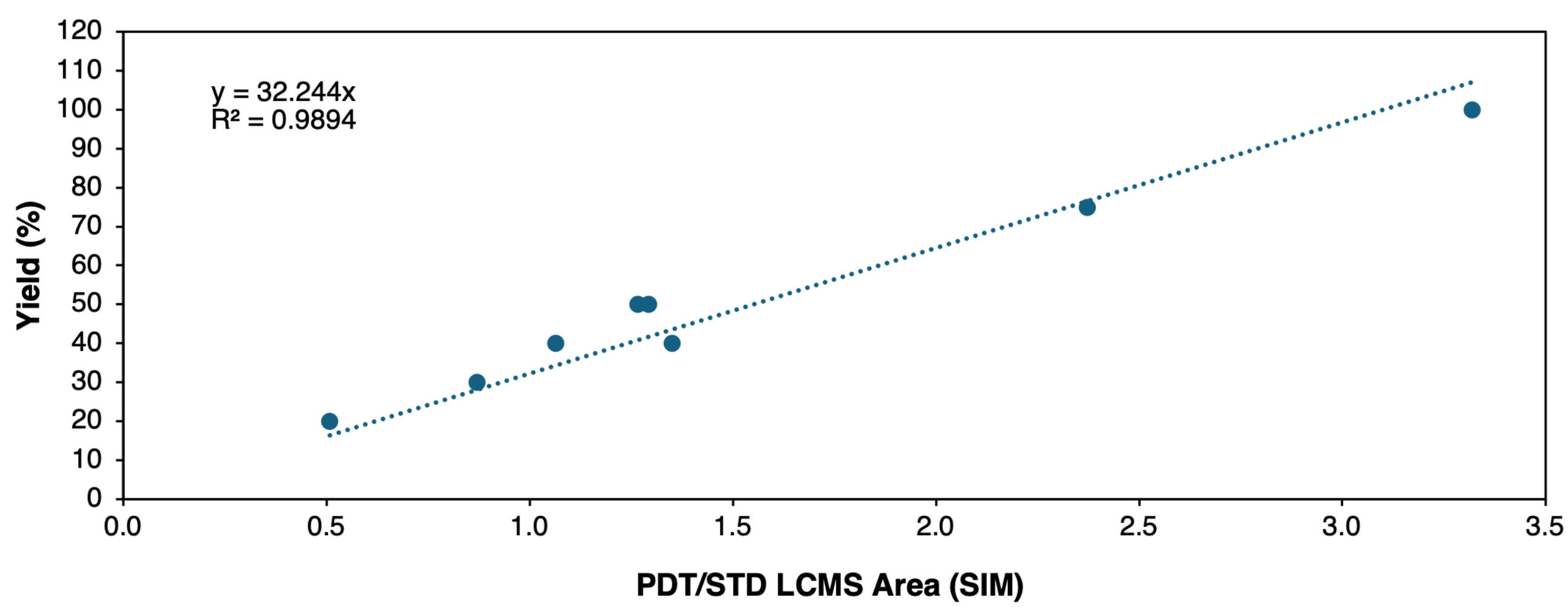

Figure S100: Calibration curve using authentic sample and papaverine as an external standard.

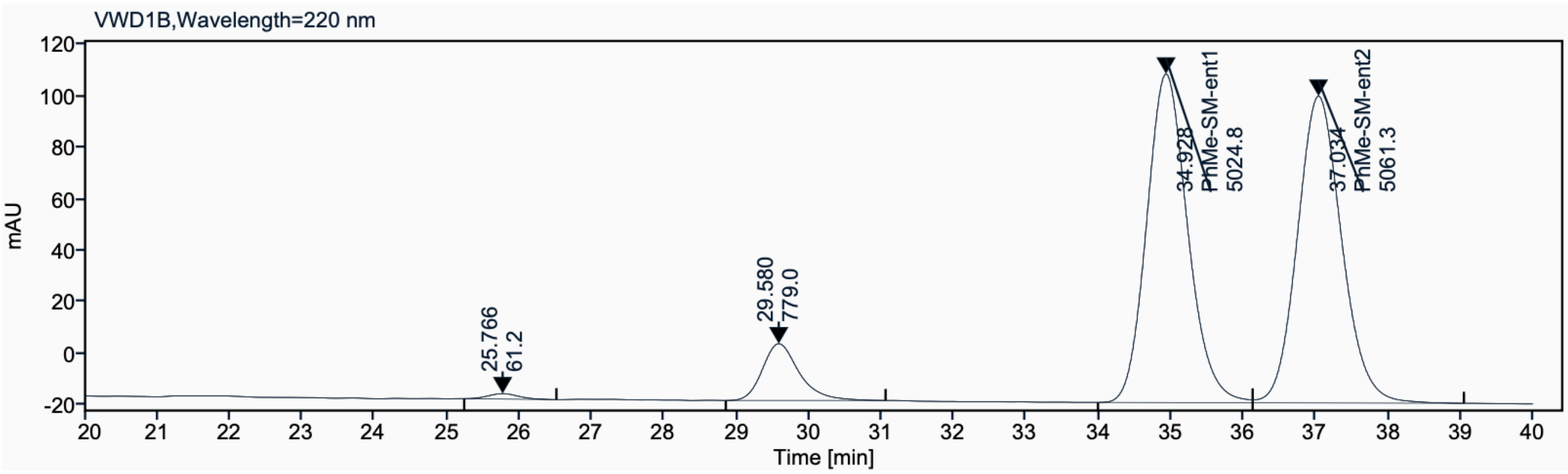

Name	RT [min]	Area
PhMe-SM-ent1	34.928	5024.7772
PhMe-SM-ent2	37.034	5061.3113

Figure S101: Chiral HPLC trace of racemic PhMeP(O)H.

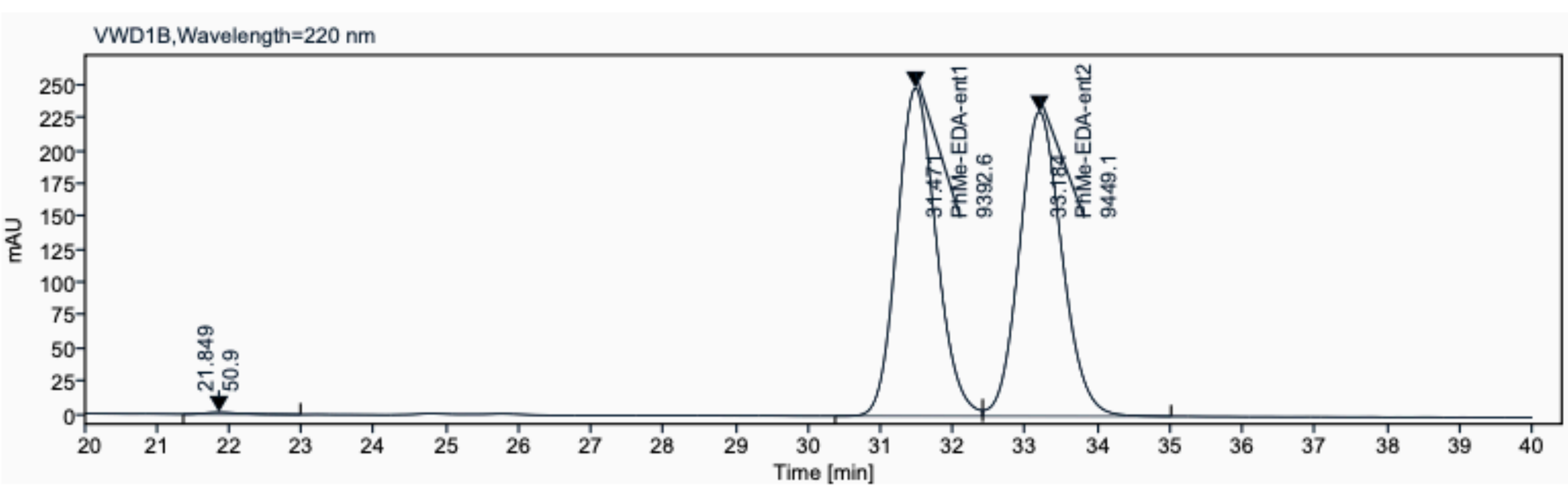

Name	RT [min]	Area
PhMe-EDA-ent1	31.471	9392.6117
PhMe-EDA-ent2	33.184	9449.0648

Figure S102: Chiral HPLC trace of racemic PhMeP(O)–CO_2Et.

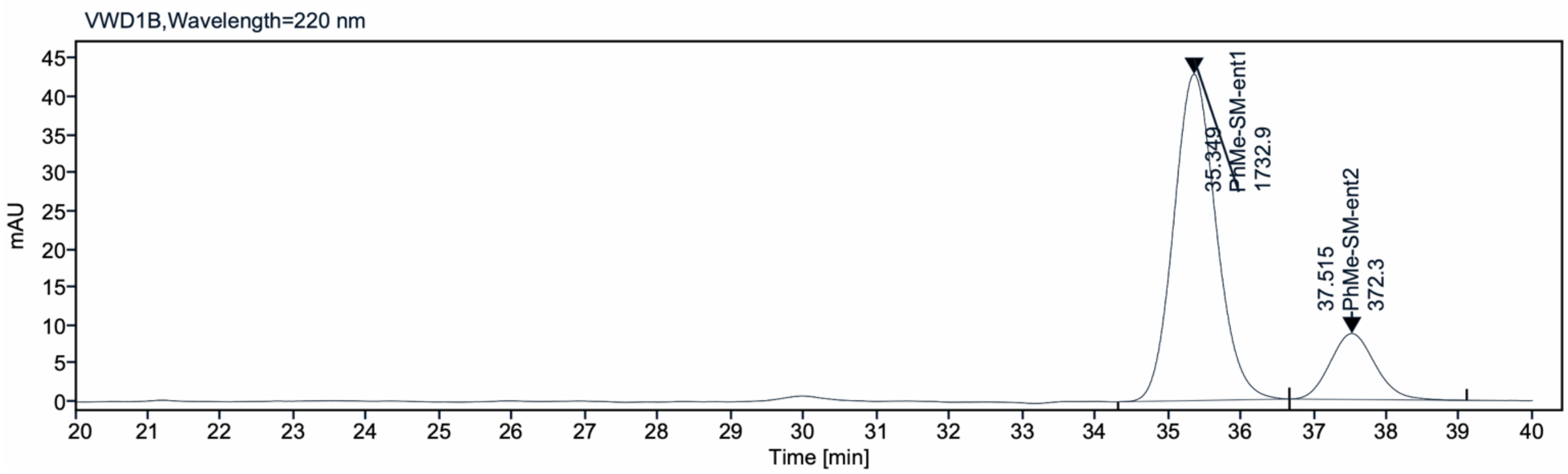

Name	RT [min]	Area
PhMe-SM-ent1	35.349	1732.9352
PhMe-SM-ent2	37.515	372.2761

Figure S103: Chiral HPLC trace of enantioenriched S_P–PhMeP(O)H from (L)-(–)-menthyl (R_P)-phenylphosphinate, 65% ee.

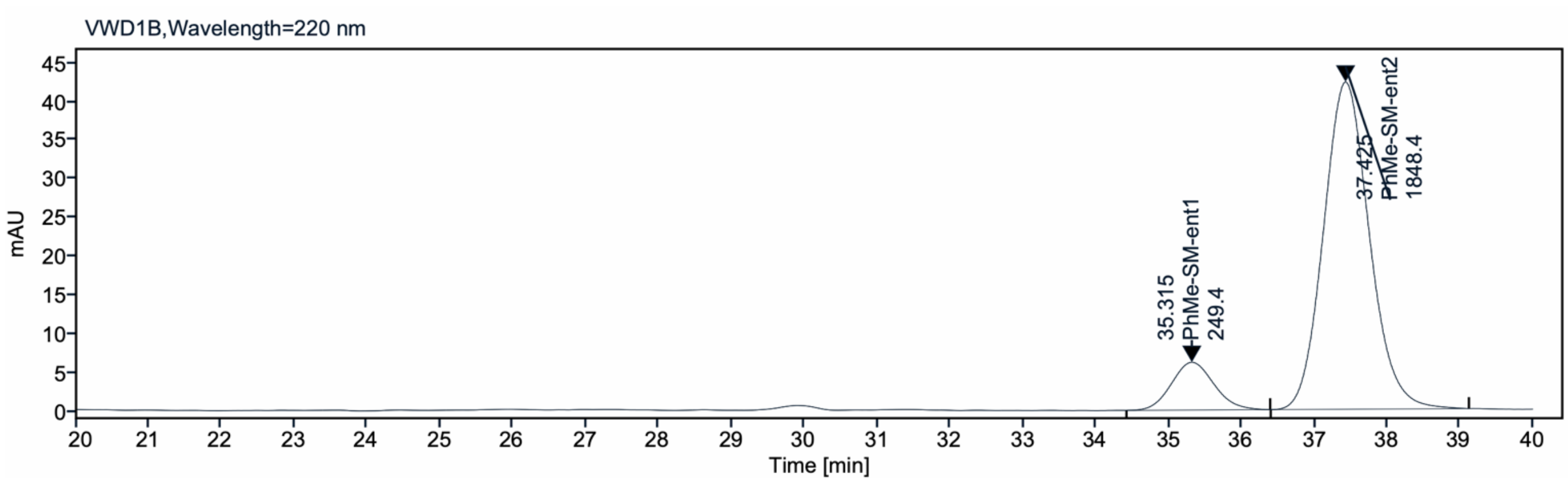

Name	RT [min]	Area
PhMe-SM-ent1	35.315	249.4147
PhMe-SM-ent2	37.425	1848.3514

Figure S104: Chiral HPLC trace of enantioenriched R_P–PhMeP(O)H from (D)-(+)-menthyl (S_P)-phenylphosphinate, – 76% ee.

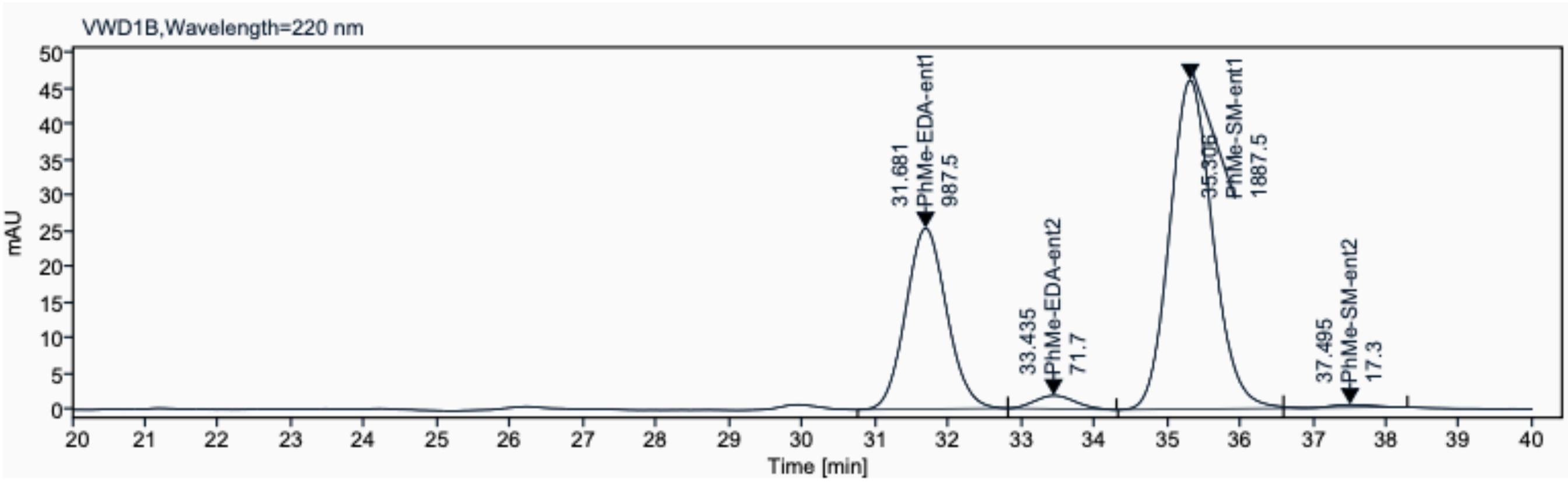

Name	RT [min]	Area
PhMe-EDA-ent1	31.681	987.5307
PhMe-EDA-ent2	33.435	71.6917
PhMe-SM-ent1	35.306	1887.5005
PhMe-SM-ent2	37.495	17.2764

Figure S105: Chiral HPLC trace of reaction catalyzed by G4, S_P–PhMeP(O)H and EDA.

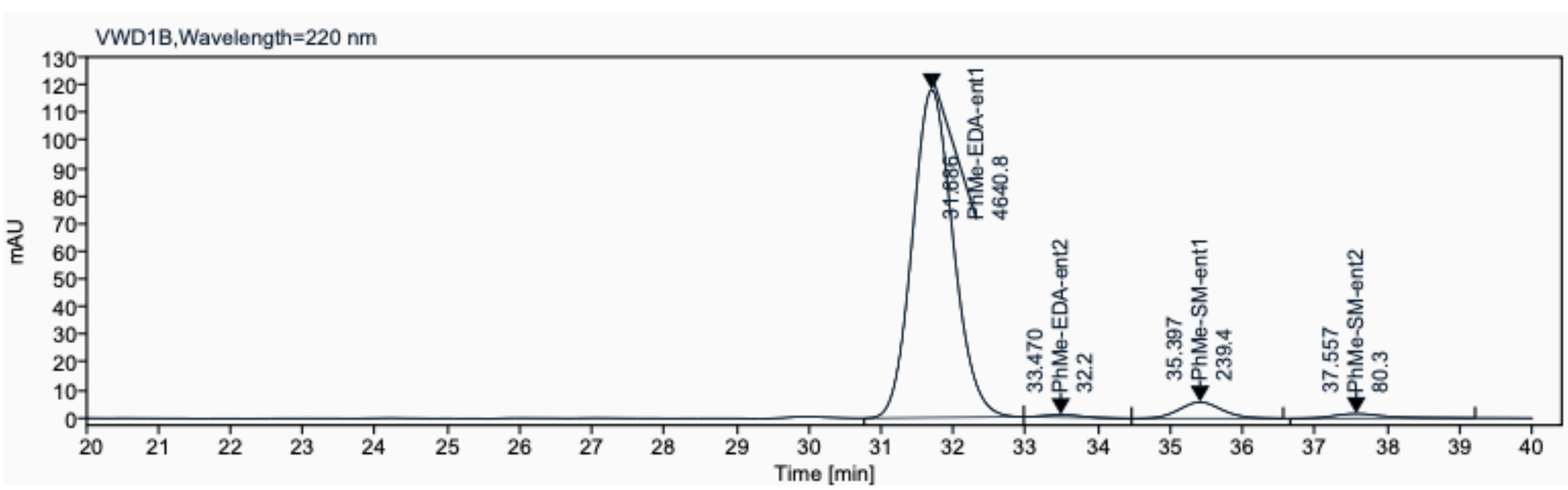

Name	RT [min]	Area
PhMe-EDA-ent1	31.686	4640.7559
PhMe-EDA-ent2	33.470	32.1675
PhMe-SM-ent1	35.397	239.4039
PhMe-SM-ent2	37.557	80.3445

Figure S106: Chiral HPLC trace of reaction catalyzed by G4, R_P–PhMeP(O)H and EDA.

8.2 Scale up and isolation of PhMeP(O)–CH_2CO_2Me (1c)

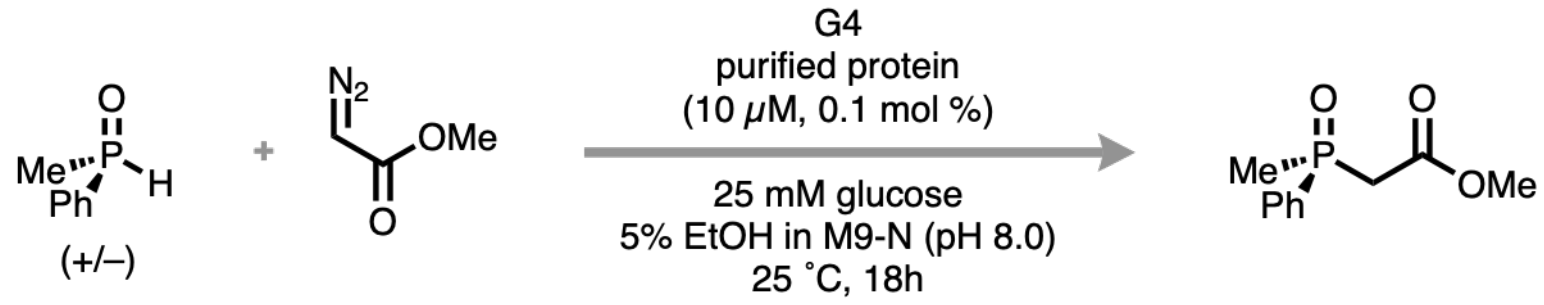

Large Scale (0.2 mmol): The standard validation procedure using whole cells or purified protein on a larger scale (20 ml, $[P]_{final}$ = 20 mM) failed to produce sufficient quantities of desired product due to poor conversion. After brief screening, it was determined that the MeDA was causing the reaction to scale poorly due to high residual solvent quantities and whole-cell conditions were resulting in unknown side product formation. Technical replicates (25x, 0.8 mL, $[P]_{final}$ = 10 mM) were used to isolate enough product. An HPLC vial was charged with M9-N buffer (643 µL, pH 8.0) and purified protein (G4, 77 µL, 104 µM, $[G4]_{final}$ = 10 µM). The vials were brought into the anaerobic COY chamber, and a solution of sodium dithionite (40 µL, 400 mM, $[DT]_{final}$ = 20 mM) and PhMeP(O)H/MeDA in EtOH (40 µL, 200 mM PhMeP(O)H, 150 mM MeDA, $[P]_{final}$ = 10 mM, $[MeDA]_{final}$ = 7.5 mM) was added. The vials were sealed with a septum cap and shaken at room temperature for 24 h. The reaction was quenched with IPA (40 µL), and an aliquot was removed for chiral HPLC analysis (>99% ee). The samples were pooled and extracted with EtOAc (4x 20 mL) – centrifugation at 5000xg for 1 minute was used to aid layer formation. The combined organics were dried with Na_2SO_4, and the solvent was removed *in vacuo*. The reaction outcome was analyzed by adding nitrobenzene as an analytical external standard (20 µL) and an aliquot was removed to determine the reaction yield (^{1}H NMR Yield, 23%).

Notes: G4 was purified according to the procedure outlined in Section 2.8 and concentration determined using hemochrome as described in Section 2.7.

Column: The resulting crude residue was purified by silica gel chromatography (10g SiliaFlash R60 SiO_2, packed w/ 80% EtOAc). The crude residue was loaded in a minimal amount of DCM (200 µL x 3), and the product was eluted with a gradient (0–15% MeOH in EtOAc over 15 CV). The fractions with product were concentrated *in vacuo* (30 ˚C). The product was insufficiently pure for optical rotation, so a prep TLC plate was used to further purify the material. A yield was not determined (>99% ee).

^{1}H NMR: (400 MHz, $CDCl_3$) δ 7.75 – 7.60 (m, 2H), 7.60 – 7.36 (m, 3H), 3.59 (s, 3H), 3.27 – 2.84 (m, 2H), 1.87 (d, J = 13.5 Hz, 3H).

^{31}P NMR: (162 MHz, $CDCl_3$) δ 31.87.

Optical Rotation: $[\alpha]^{23}_D$ = – 31.4 (c = 1, MeOH).[29]

Chiral HPLC: Chiralpak OJH (Hex/EtOH = 90:10, 1 mL/min).

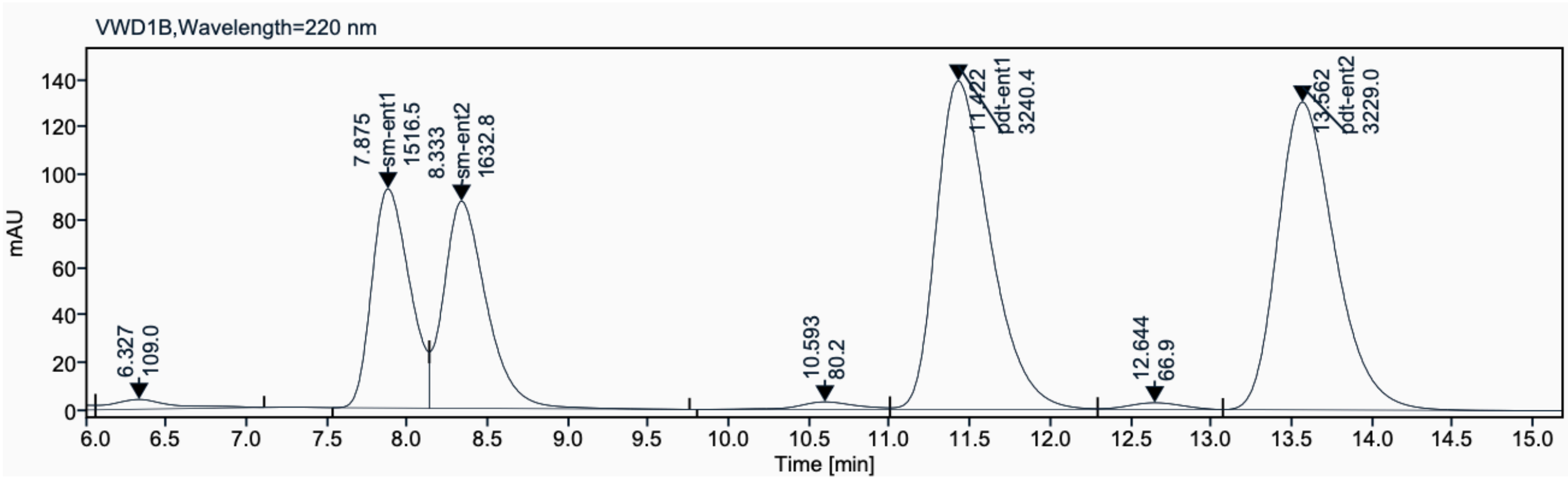

Name	RT [min]	Area
sm-ent1	7.875	1516.5345
sm-ent2	8.333	1632.8006
pdt-ent1	11.422	3240.4127
pdt-ent2	13.562	3228.9522

Figure S107: Chiral HPLC trace of racemic PhMeP(O)H and PhMeP(O)–CH_2CO_2Me.

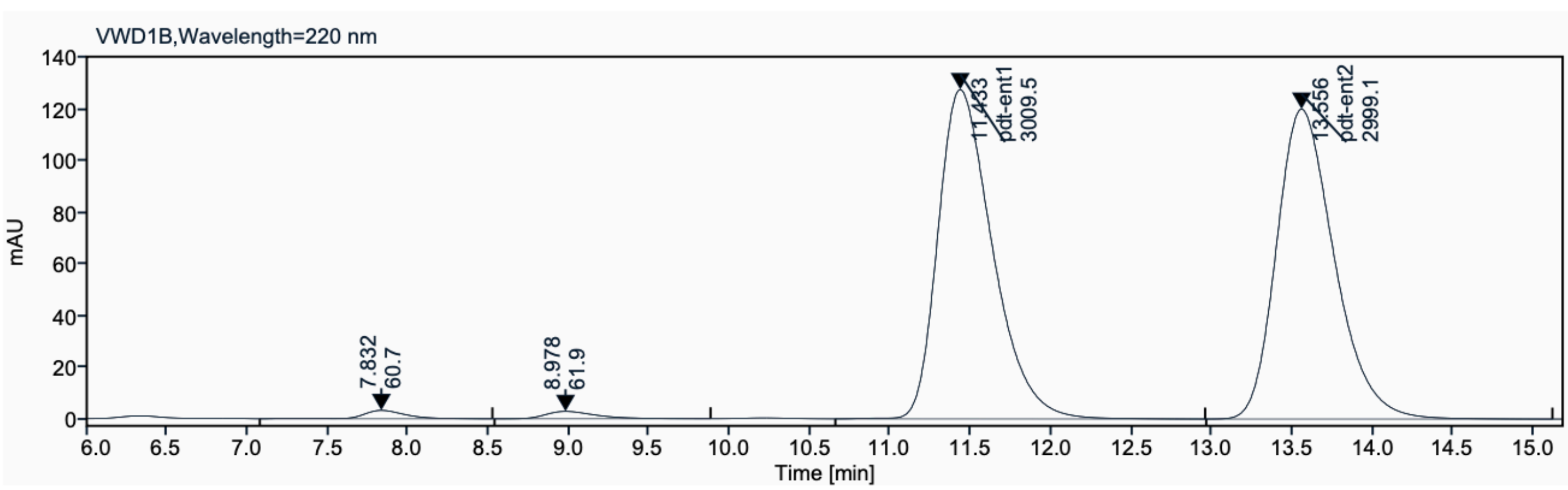

Name	RT [min]	Area
pdt-ent1	11.433	3009.5185
pdt-ent2	13.556	2999.0934

Figure S108: Chiral HPLC trace of racemic PhMeP(O)–CH_2CO_2Me.

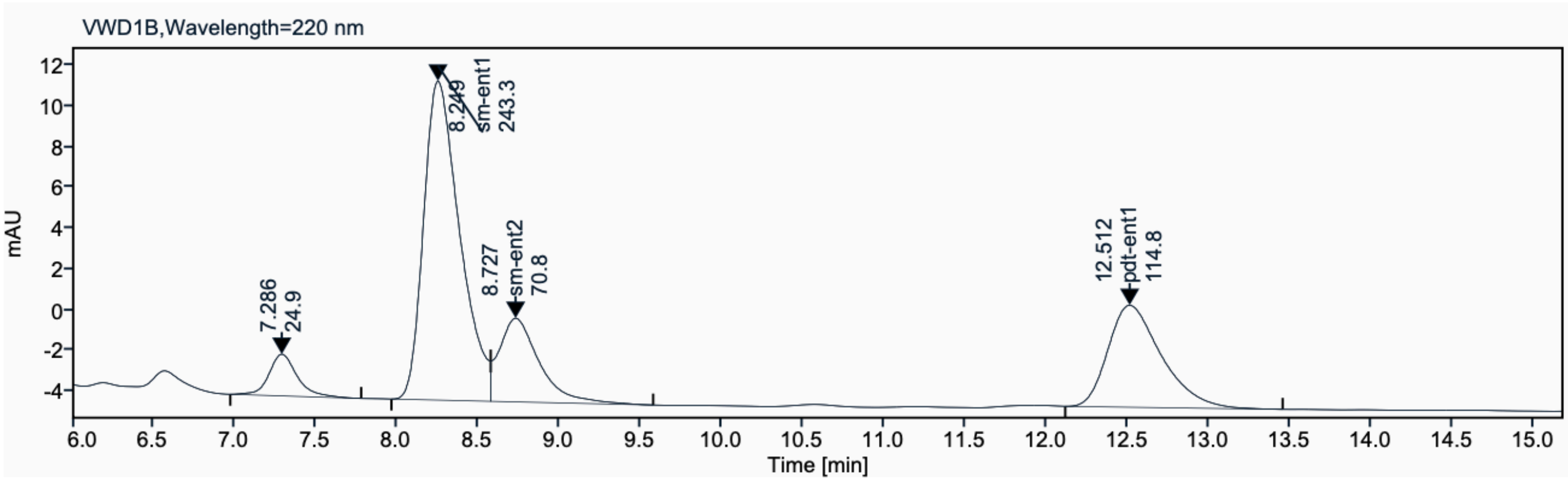

Name	RT [min]	Area
sm-ent1	8.249	243.3229
sm-ent2	8.727	70.8301
pdt-ent1	12.512	114.7779

Figure S109: Chiral HPLC trace of reaction catalyzed by G4, PhMeP(O)H and MeDA.

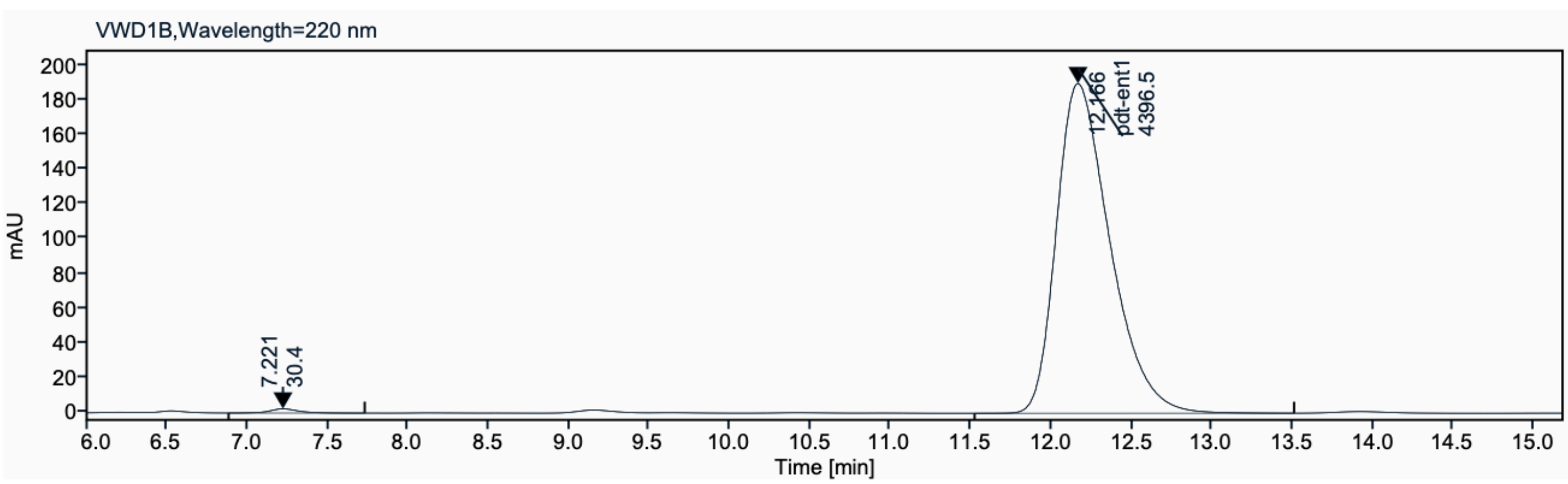

Name	RT [min]	Area
pdt-ent1	12.166	4396.5419

Figure S110: Chiral HPLC trace of purified product from large scale reaction catalyzed by G4, PhMeP(O)H and MeDA.

8.3 Scale-up and isolation of Ph(OEt)P(O)–CH_2CO_2Me (2c)

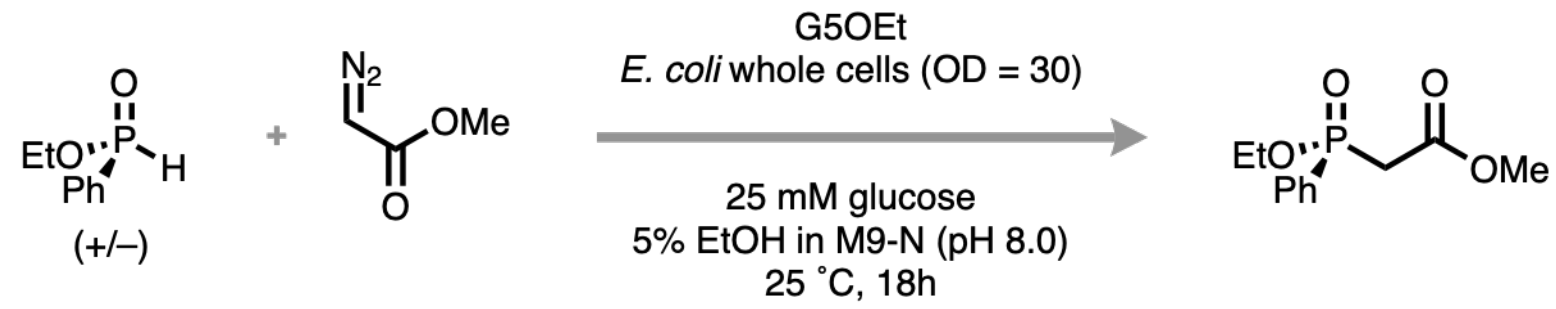

Large Scale (0.4 mmol): The standard validation procedure using whole cells or purified protein on a larger scale (20 ml, $[P]_{final}$ = 20 mM) failed to produce sufficient quantities of desired product due to poor conversion. After brief screening it was determined that the MeDA was causing the reaction to scale poorly due to high residual solvent quantities. Technical replicates (50x, 0.8 mL, $[P]_{final}$ = 10 mM) were used to isolate enough product.

An HPLC vial was charged with normalized cell suspension (G5OEt, 720 µL, OD_{600} = 30, M9-N, pH 8.0) and glucose (40 µL, 500 mM, $[glc]_{final}$ = 25 mM). The vials were brought into the anaerobic COY chamber and a solution of Ph(OEt)P(O)H and MeDA in EtOH (40 µL, 200 mM Ph(OEt)P(O)H, 150 mM MeDA, $[P]_{final}$ = 10 mM, $[MeDA]_{final}$ = 7.5 mM) was added. The vials were sealed with a septum cap and shaken at room temperature for 24 h. The reaction was quenched with IPA (40 µL), and an aliquot was removed for chiral HPLC analysis (>99% ee). The samples were pooled and extracted with EtOAc (4x 20 mL) – centrifugation at 5000xg for 1 minute was used to aid layer formation. The combined organics were dried with Na_2SO_4, and the solvent was removed *in vacuo*. The reaction outcome was analyzed by adding nitrobenzene as an analytical external standard (20 µL), and an aliquot was removed to determine the reaction yield (^{1}H NMR Yield, 12%).

Column: The resulting crude residue was purified by silica gel chromatography (10g SiliaFlash R60 SiO_2, packed w/ 80% EtOAc). The crude residue was loaded in a minimal amount of DCM (200 µL x 3), and the product was eluted with a gradient (0–15% MeOH in EtOAc over 15 CV). The fractions with product were concentrated in vacuo (30 ˚C). The product was insufficiently pure for optical rotation, so a prep TLC plate was used to further purify the material. A yield was not determined (>99% ee).

^{1}H NMR: (400 MHz, $CDCl_3$) δ 7.92 – 7.69 (m, 2H), 7.66 – 7.56 (m, 1H), 7.54 – 7.41 (m, 2H), 4.27 – 4.11 (m, 1H), 4.03 (ddq, *J* = 10.1, 8.2, 7.1 Hz, 1H), 3.64 (d, *J* = 0.6 Hz, 3H), 3.14 (dd, *J* = 17.8, 0.7 Hz, 2H), 1.35 (t, *J* = 7.0 Hz, 3H).

^{31}P NMR: (162 MHz, $CDCl_3$) δ 33.82.

Optical Rotation: $[\alpha]^{23}_D$ = 14.5 (c = 1, MeOH).[30]

Chiral HPLC: Chiralpak IC (Hex/IPA = 50:50, 1 mL/min).

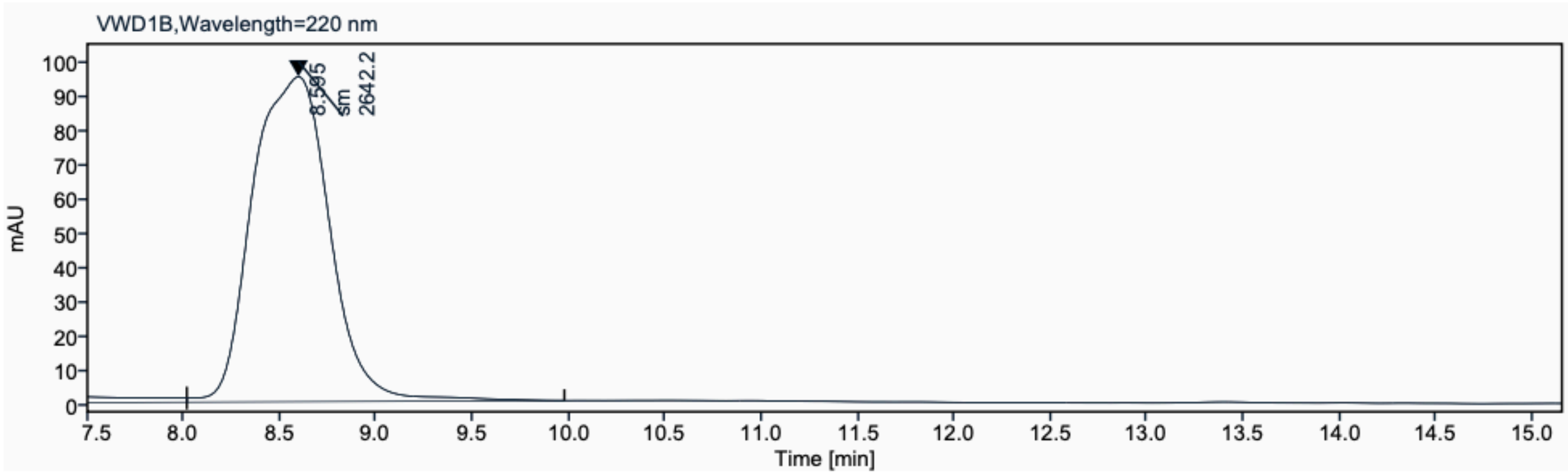

Name	RT [min]	Area
sm	8.595	2642.2473

Figure S111: Chiral HPLC trace of racemic Ph(OEt)P(O)H.

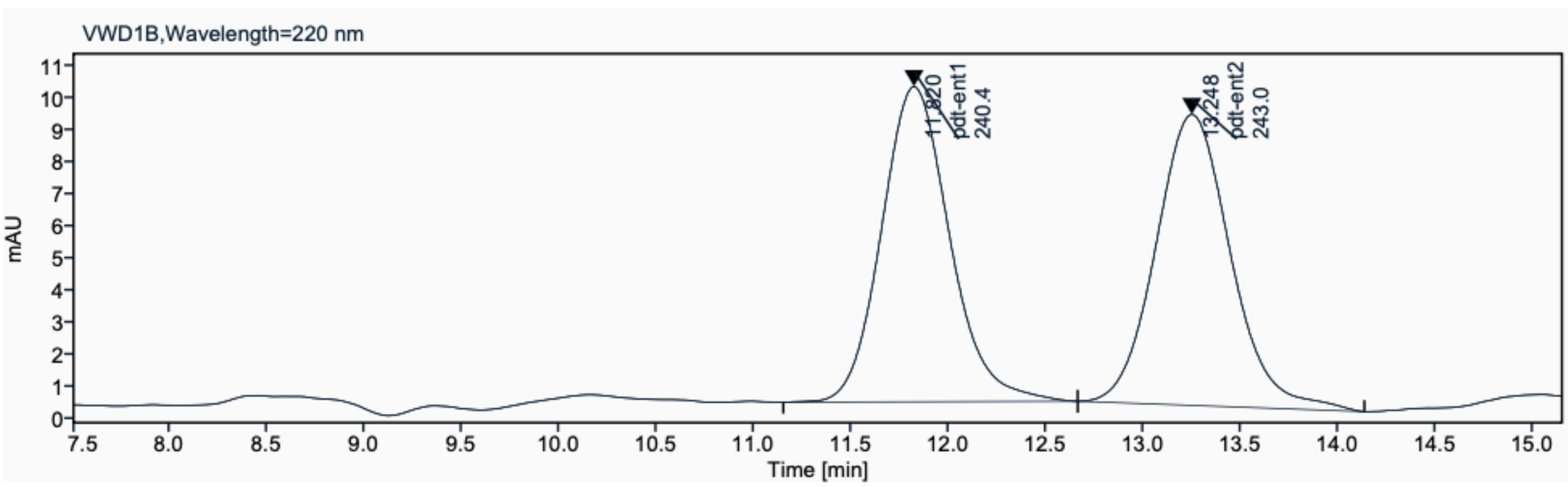

Name	RT [min]	Area
pdt-ent1	11.820	240.3764
pdt-ent2	13.248	243.0292

Figure S112: Chiral HPLC trace of racemic Ph(OEt)P(O)–CH_2CO_2Me.

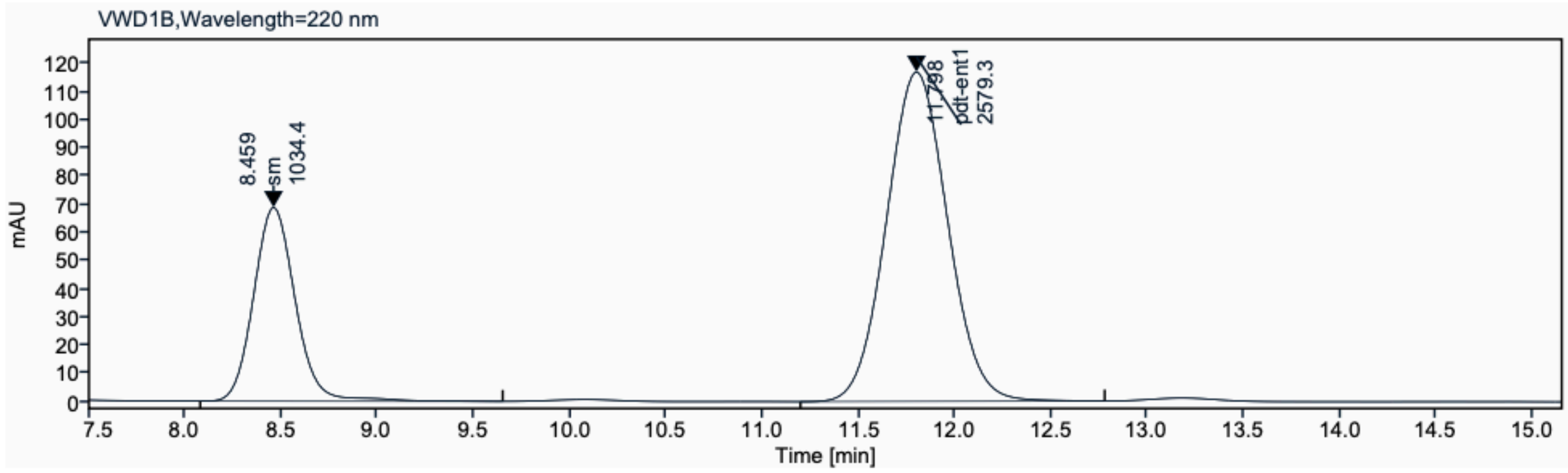

Name	RT [min]	Area
sm	8.459	1034.4143
pdt-ent1	11.798	2579.2948

Figure S113: Chiral HPLC trace of reaction catalyzed by G5OEt, Ph(OEt)P(O)H and MeDA.

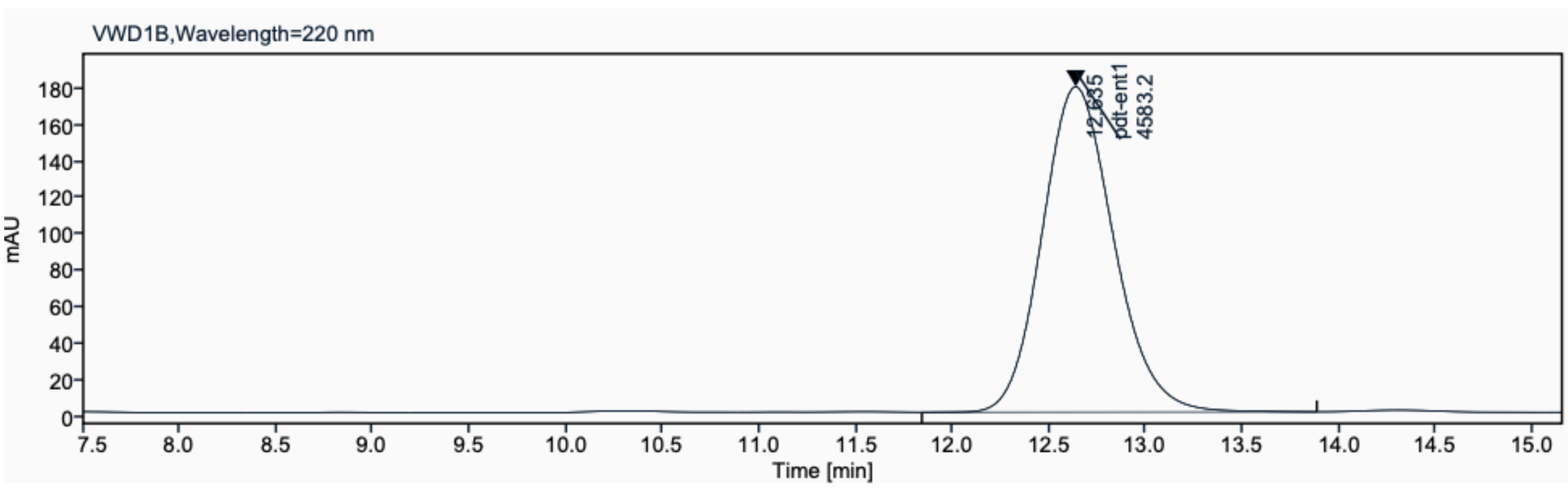

Name	RT [min]	Area
pdt-ent1	12.635	4583.2301

Figure S114: Chiral HPLC trace of purified product from large scale reaction catalyzed by G5OEt, Ph(OEt)P(O)H and MeDA.

8.4 Synthesis of enantioenriched phosphine oxides to assign absolute stereochemistry

The mechanistic studies in Sections 8.1–8.4 supported an enzyme catalyzed stereospecific, stereoretentive P–H insertion. However, due to the varied substituents analyzed within the scope we wanted to provide additional evidence for assigning the absolute stereochemistry of catalyst-substrate pairs. We sought to utilize the knowledge that the enzymatic reaction is stereoretentive to assign absolute stereochemistry based on the selective consumption of starting material. However, we did not have access to the chiral columns utilized by various publications to assign the stereochemistry of the starting material based on comparison of chiral HPLC traces. Thus, we utilized a reported method for resolution of secondary phosphine oxides with spiro-TADDOL [(R,R)-2] complexes.[10] Conditions were chosen that provided the best selectivity and moderate recovery upon one crystallization.

Note: Ph(2-Np)P(O)H was assigned based on analogy to Ph(1-Np)P(O)H.

Table S52: Synthesis of enantioenriched substrates and assignment of absolute stereochemistry.

PhRP(O)H	recrystallization specifications	reported ee and abs. configuration	chiral HPLC conditions	observed ee	assignment
PhMeP(O)H	2 equiv TADDOL<br>6 mL / g TADDOL<br>IPA	61% ee<br>P_R	Chiralpak IB and IC in series (Hex/EtOH = 85:15, 1 mL/min)	71% ee	t_R (P_S) = 33.3 min, t_R (P_R) = 35.1 min
PhBnP(O)H	2 equiv TADDOL<br>6 mL / g TADDOL<br>Toluene then Hexane	39% ee<br>P_S	Chiralpak IA (Hex/EtOH = 91:09, 1 mL/min).	27% ee	t_R (P_R) = 16.4 min, t_R (P_S) = 17.9 min
PhCyP(O)H	0.5 equiv TADDOL<br>5 mL / g TADDOL<br>Toluene then Hexane	76% ee<br>P_S	Chiralpak IA (Hex/EtOH = 92:08, 1 mL/min).	27% ee	t_R (P_S) = 13.6 min, t_R (P_R) = 16.4 min
Ph(*o*An)P(O)H	0.5 equiv TADDOL<br>6 mL / g TADDOL<br>IPA	28% ee<br>P_R	Chiralpak IC (Hex/IPA = 60:40, 1 mL/min).	42% ee	t_R (P_S) = 22.9 min, t_R (P_R) = 26.4 min
Ph(2-Np)P(O)H	0.5 equiv TADDOL<br>7.5 mL / g TADDOL<br>Toluene then Hexane	N.D.	Chiralpak IA (Hex/IPA = 75:25, 1 mL/min).	45% ee	t_R (P_S) = 9.0 min, t_R (P_R) = 9.5 min
Ph(1-Np)P(O)H	0.5 equiv TADDOL<br>7.5 mL / g TADDOL<br>Toluene then Hexane	33% ee<br>P_R	N.D.	N.D.	N.D

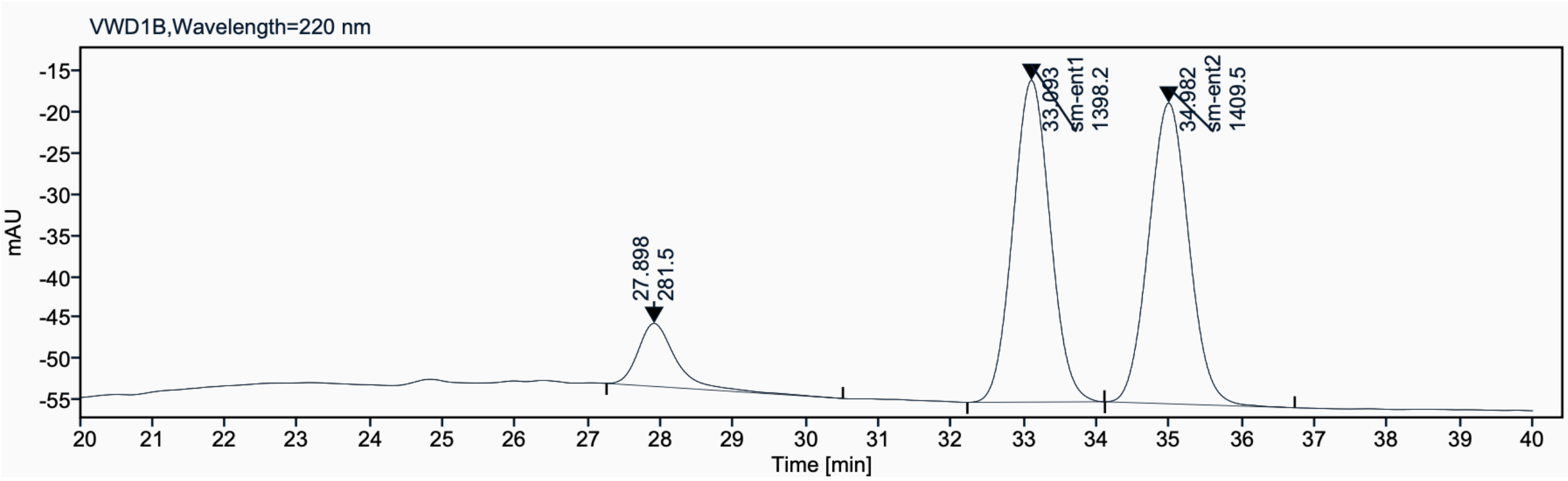

Name	RT [min]	Area
sm-ent1	33.093	1398.2126
sm-ent2	34.982	1409.4517

Figure S115: Chiral HPLC trace of racemic PhMeP(O)H.

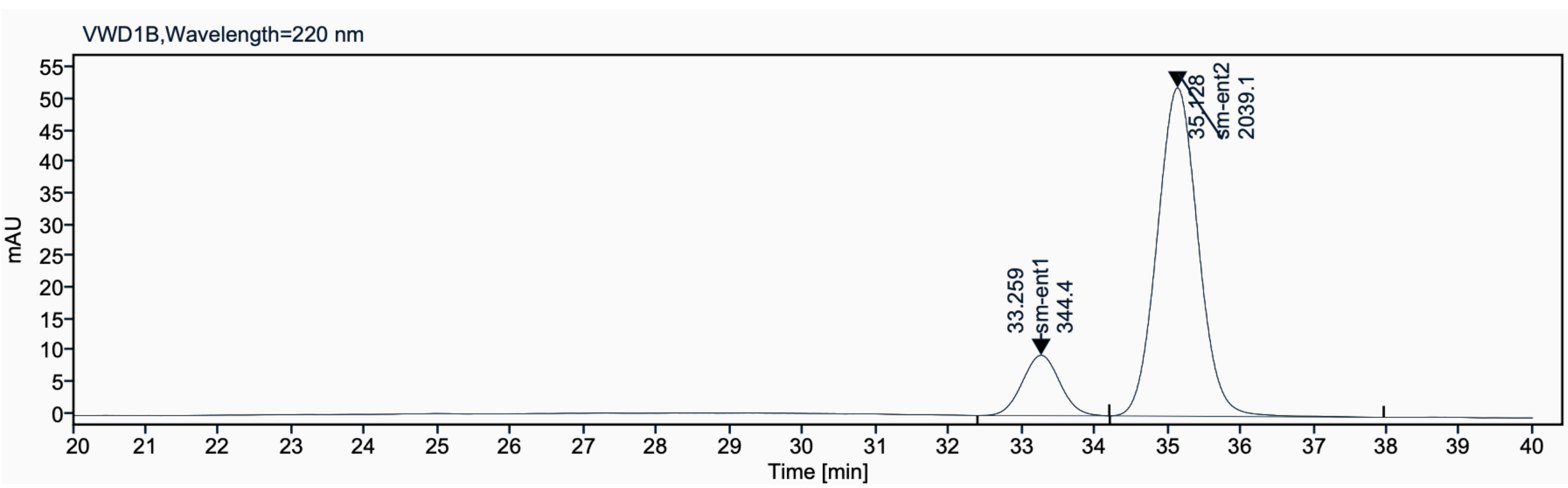

Name	RT [min]	Area
sm-ent1	33.259	344.3644
sm-ent2	35.128	2039.0913

Figure S116: Chiral HPLC trace enantioenriched PhMeP(O)H.

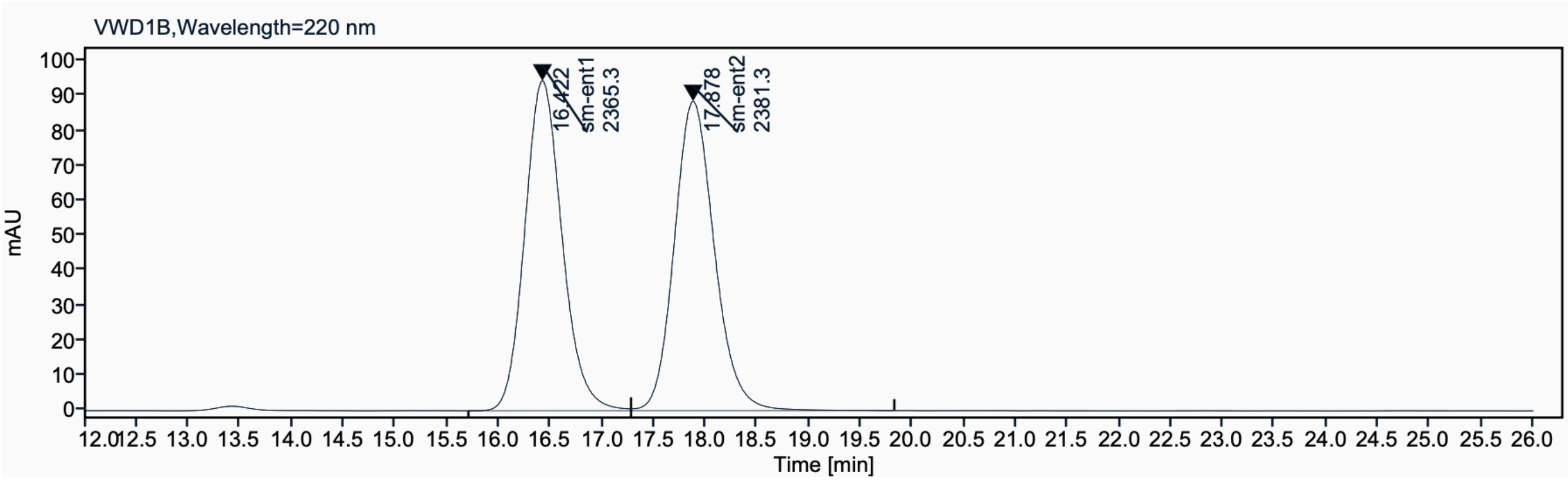

Name	RT [min]	Area
sm-ent1	16.422	2365.2748
sm-ent2	17.878	2381.2774

Figure S117: Chiral HPLC trace of racemic PhBnP(O)H.

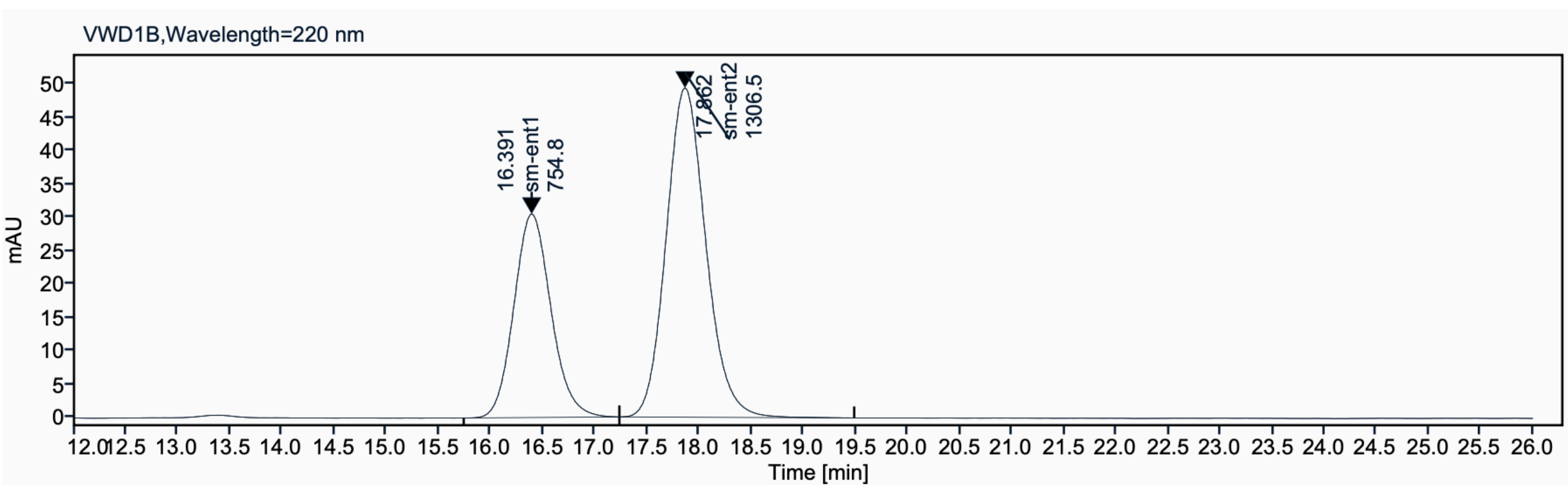

Name	RT [min]	Area
sm-ent1	16.391	754.8424
sm-ent2	17.862	1306.4890

Figure S118: Chiral HPLC trace enantioenriched PhBnP(O)H.

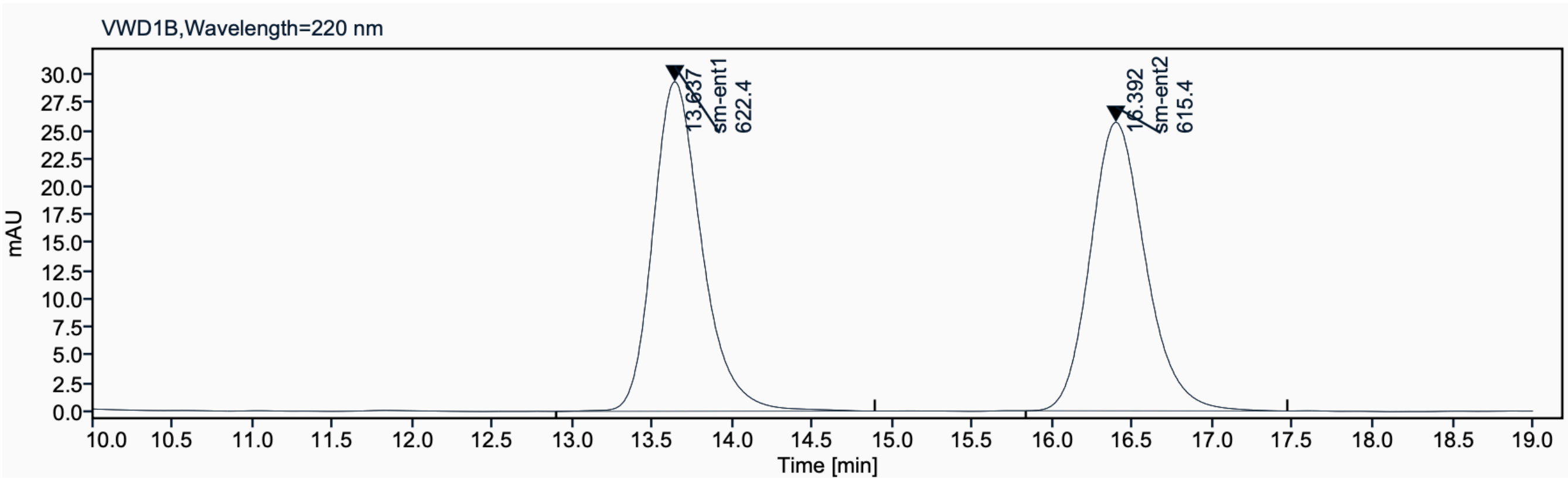

Name	RT [min]	Area
sm-ent1	13.637	622.4039
sm-ent2	16.392	615.3625

Figure S119: Chiral HPLC trace of racemic PhCyP(O)H.

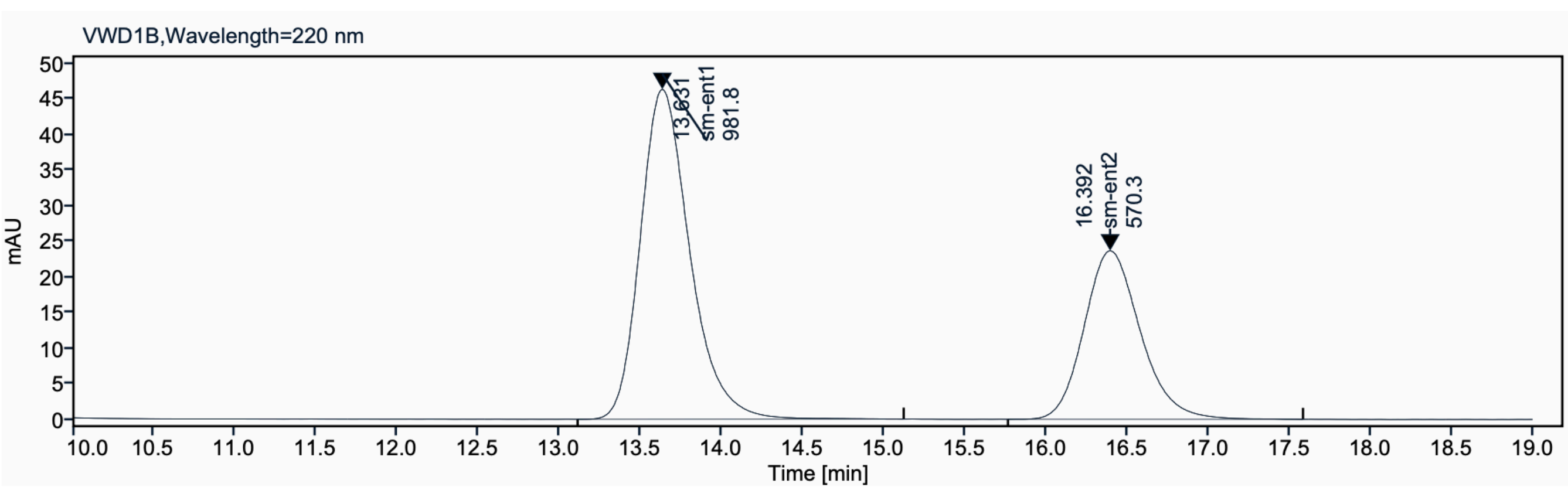

Name	RT [min]	Area
sm-ent1	13.631	981.7903
sm-ent2	16.392	570.3399

Figure S120: Chiral HPLC trace enantioenriched PhCyP(O)H.

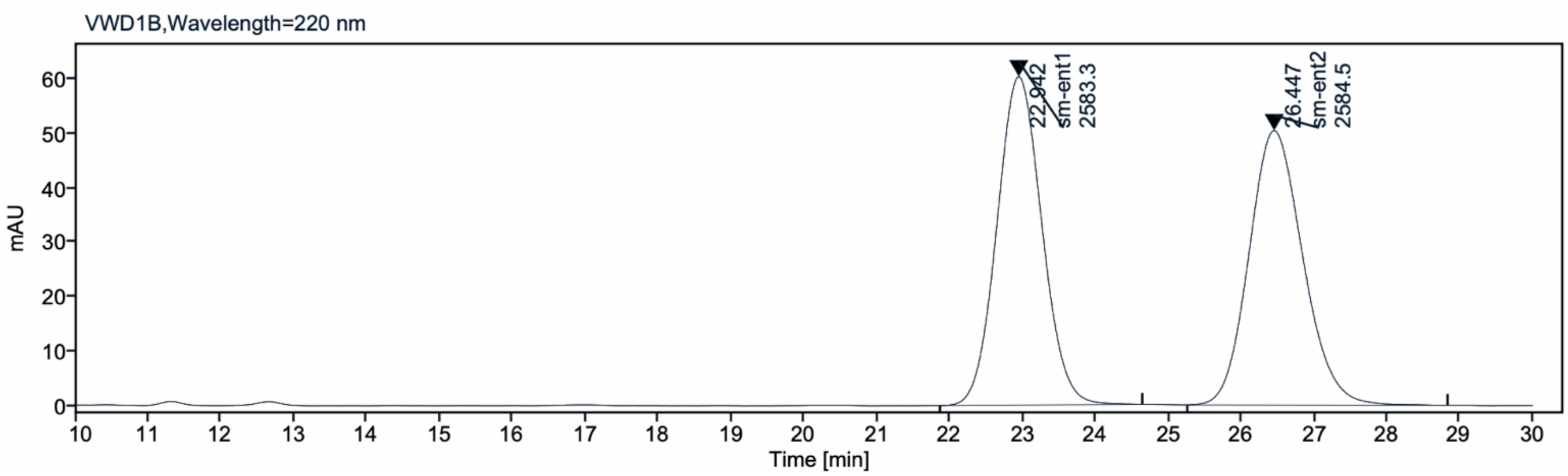

Name	RT [min]	Area
sm-ent1	22.942	2583.3481
sm-ent2	26.447	2584.4611

Figure S121: Chiral HPLC trace of racemic Ph^{o}AnP(O)H.

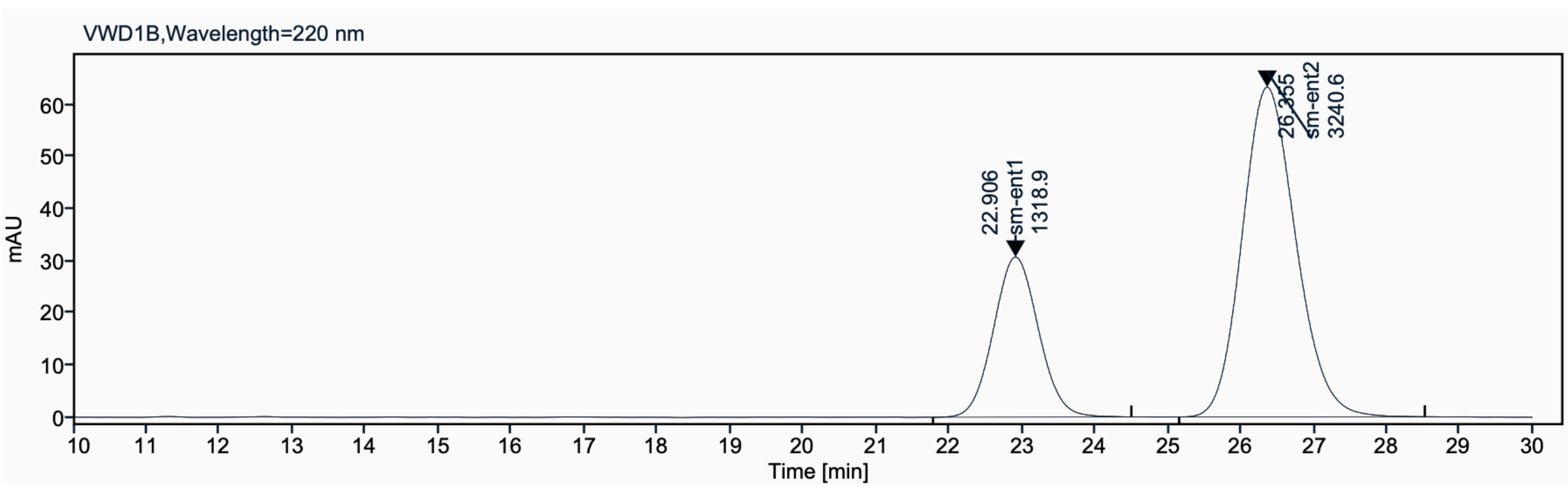

Name	RT [min]	Area
sm-ent1	22.906	1318.9228
sm-ent2	26.355	3240.6125

Figure S122: Chiral HPLC trace enantioenriched Ph^{o}AnP(O)H.

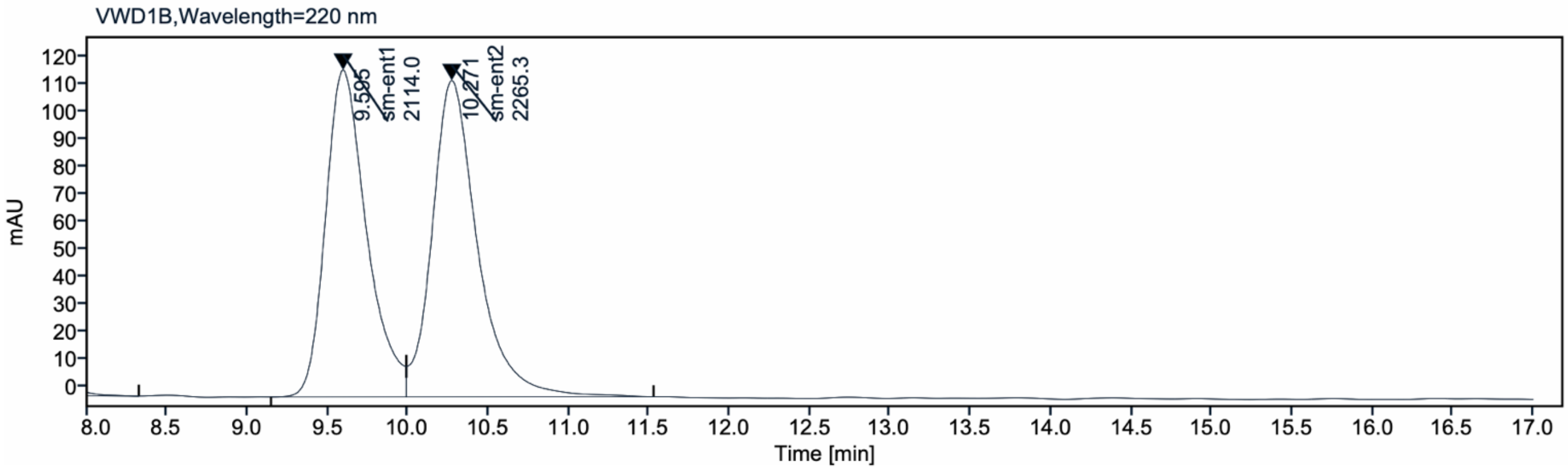

Name	RT [min]	Area
sm-ent1	9.595	2114.0427
sm-ent2	10.271	2265.2847

Figure S123: Chiral HPLC trace of racemic Ph(2-Np)P(O)H.

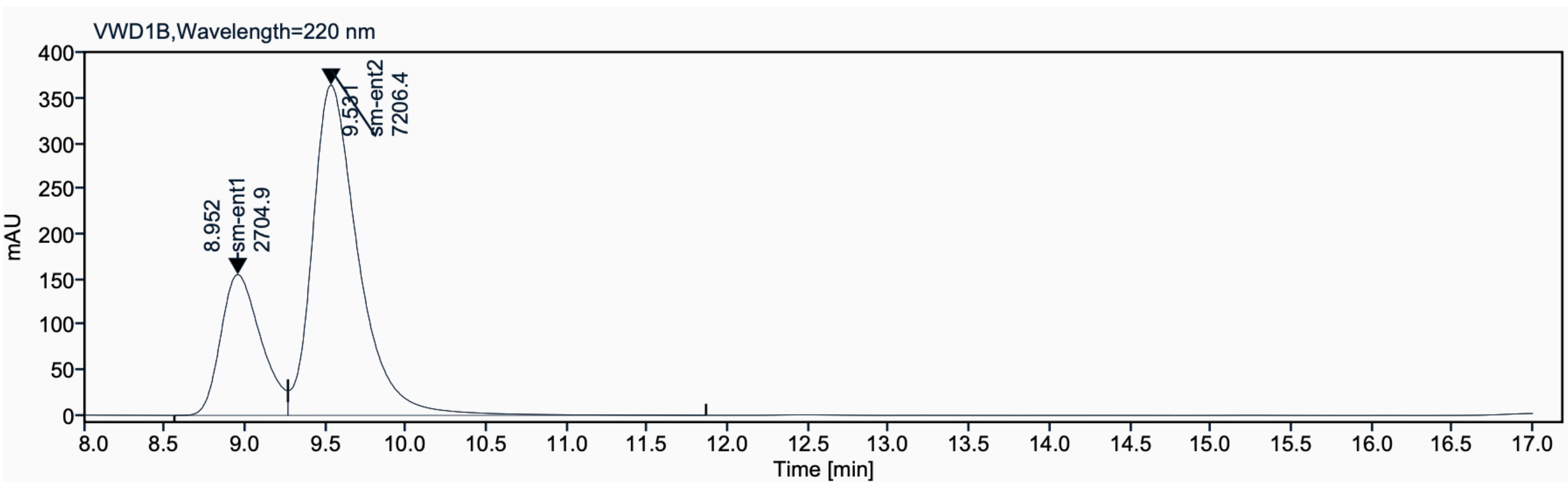

Name	RT [min]	Area
sm-ent1	8.952	2704.9414
sm-ent2	9.531	7206.3732

Figure S124: Chiral HPLC trace enantioenriched Ph(2-Np)P(O)H.

9. References

(1) Mao, R.; Taylor, D. M.; Wackelin, D. J.; Rogge, T.; Wu, S. J.; Sicinski, K. M.; Houk, K. N.; Arnold, F. H. Biocatalytic, Stereoconvergent Alkylation of (Z/E)-Trisubstituted Silyl Enol Ethers. *Nat. Synth.* **2023**, *3* (2), 256–264. https://doi.org/10.1038/s44160-023-00431-2.
(2) Sattely, E. S.; Meek, S. J.; Malcolmson, S. J.; Schrock, R. R.; Hoveyda, A. H. Design and Stereoselective Preparation of a New Class of Chiral Olefin Metathesis Catalysts and Application to Enantioselective Synthesis of Quebrachamine: Catalyst Development Inspired by Natural Product Synthesis. *J. Am. Chem. Soc.* **2009**, *131* (3), 943–953. https://doi.org/10.1021/ja8084934.
(3) Gibson, D. G.; Young, L.; Chuang, R.-Y.; Venter, J. C.; Hutchison, C. A.; Smith, H. O. Enzymatic Assembly of DNA Molecules up to Several Hundred Kilobases. *Nat. Methods* **2009**, *6* (5), 343–345. https://doi.org/10.1038/nmeth.1318.
(4) Boville, C. E.; Scheele, R. A.; Koch, P.; Brinkmann-Chen, S.; Buller, A. R.; Arnold, F. H. Engineered Biosynthesis of β-Alkyl Tryptophan Analogues. *Angew. Chem. Int. Ed.* **2018**, *57* (45), 14764–14768. https://doi.org/10.1002/anie.201807998.
(5) Zhao, H.; Giver, L.; Shao, Z.; Affholter, J. A.; Arnold, F. H. Molecular Evolution by Staggered Extension Process (StEP) in Vitro Recombination. *Nat. Biotechnol.* **1998**, *16* (3), 258–261. https://doi.org/10.1038/nbt0398-258.
(6) Long, Y.; Mora, A.; Li, F.-Z.; Gürsoy, E.; Johnston, K. E.; Arnold, F. H. LevSeq: Rapid Generation of Sequence-Function Data for Directed Evolution and Machine Learning. *ACS Synth. Biol.* **2025**, *14* (1), 230–238. https://doi.org/10.1021/acssynbio.4c00625.
(7) Barr, I.; Guo, F. Pyridine Hemochromagen Assay for Determining the Concentration of Heme in Purified Protein Solutions. *BIO-Protoc.* **2015**, *5* (18). https://doi.org/10.21769/BioProtoc.1594.
(8) *Ligation sequencing DNA V14 (SQK-LSK114)*. Oxford Nanopore Technologies. https://nanoporetech.com/document/genomic-dna-by-ligation-sqk-lsk114 (accessed 2025-07-16).
(9) Wang, B.; Liu, Y.; Jiang, C.; Cao, Z.; Cao, S.; Zhao, X.; Ban, X.; Yin, Y.; Jiang, Z. Catalytic Asymmetric Hydrophosphinylation of 2-Vinylazaarenes to Access P-Chiral 2-Azaaryl-Ethylphosphine Oxides. *Angew. Chem. Int. Ed.* **2023**, *62* (16), e202216605. https://doi.org/10.1002/anie.202216605.
(10) Varga, B.; Szemesi, P.; Nagy, P.; Herbay, R.; Holczbauer, T.; Fogassy, E.; Keglevich, G.; Bagi, P. Enantioseparation of *P* -Stereogenic Secondary Phosphine Oxides and Their Stereospecific Transformation to Various Tertiary Phosphine Oxides and a Thiophosphinate. *J. Org. Chem.* **2021**, *86* (21), 14493–14507. https://doi.org/10.1021/acs.joc.1c01364.
(11) Xiong, B.; Li, M.; Cao, R.; Yue, S.; Xu, W.; Liu, Y.; Zhu, L.; Tang, K.-W. Elemental Sulfur/Selenium-Mediated Metal-Free Phosphinothioation and Phosphinoselenoation of Vinylsulfonium Salts with P–H Bonds. *J. Org. Chem.* **2025**, *90* (1), 275–291. https://doi.org/10.1021/acs.joc.4c02237.
(12) Notar Francesco, I.; Egloff, C.; Wagner, A.; Colobert, F. Stereoselective Addition of Grignard Reagents to New *P* -Chirogenic *N* -Phosphinoylbenzaldimines: Effect of the Phosphorus Substituents on the Stereoselectivity. *Eur. J. Org. Chem.* **2011**, *2011* (20–21), 4037–4045. https://doi.org/10.1002/ejoc.201100380.
(13) Guo, H.; Wu, Q.; Wang, S.; Shu, H.; Shi, E. Facile Synthesis of *H* -Phosphinates from $P(OR)_3$ or $ClP(OR)_2$ via SiO_2 -Promoted Hydrolysis. *J. Org. Chem.* **2024**, *89* (12), 8915–8923. https://doi.org/10.1021/acs.joc.4c00760.
(14) Jiang, H.; Jin, H.; Abdukader, A.; Lin, A.; Cheng, Y.; Zhu, C. Catalyst-Controlled Switchable Phosphination of α-Diazoesters. *Org. Biomol. Chem.* **2013**, *11* (22), 3612. https://doi.org/10.1039/c3ob40429c.
(15) Kaźmierczak, A.; Kusy, D.; Niinivehmas, S. P.; Gmach, J.; Joachimiak, Ł.; Pentikäinen, O. T.; Gendaszewska-Darmach, E.; Błażewska, K. M. Identification of the Privileged Position in the

Imidazo[1,2- *a*]Pyridine Ring of Phosphonocarboxylates for Development of Rab Geranylgeranyl Transferase (RGGT) Inhibitors. *J. Med. Chem.* **2017**, *60* (21), 8781–8800. https://doi.org/10.1021/acs.jmedchem.7b00811.
(16) Cecconi, F.; Dominguez, S.; Masciocchi, N.; Midollini, S.; Sironi, A.; Vacca, A. Complexation of Beryllium(II) Ion by Phosphinate Ligands in Aqueous Solution. Synthesis and XRPD Structure Determination of Be[$(PhPO_2)_2CH_2$]$(H_2O)_2$. *Inorg. Chem.* **2003**, *42* (7), 2350–2356. https://doi.org/10.1021/ic0261979.
(17) Nocentini, A.; Gratteri, P.; Supuran, C. T. Phosphorus versus Sulfur: Discovery of Benzenephosphonamidates as Versatile Sulfonamide-Mimic Chemotypes Acting as Carbonic Anhydrase Inhibitors. *Chem. – Eur. J.* **2019**, *25* (5), 1188–1192. https://doi.org/10.1002/chem.201805039.
(18) Kim, Y. R.; Cho, S.; Lee, P. H. Metal-Free Azaphosphaannulation of Phosphonamides through Intramolecular Oxidative C–N Bond Formation. *Org. Lett.* **2014**, *16* (11), 3098–3101. https://doi.org/10.1021/ol501207w.
(19) Kennemur, J. L.; Long, Y.; Ko, C. J.; Das, A.; Arnold, F. H. Enzymatic Stereodivergent Synthesis of Azaspiro[2.y]Alkanes. *J. Am. Chem. Soc.* **2025**, *147* (31), 27165–27171. https://doi.org/10.1021/jacs.5c07015.
(20) Schaus, L.; Das, A.; Knight, A. M.; Jimenez-Osés, G.; Houk, K. N.; Garcia-Borràs, M.; Arnold, F. H.; Huang, X. Protoglobin-Catalyzed Formation of *Cis* -Trifluoromethyl-Substituted Cyclopropanes by Carbene Transfer. *Angew. Chem. Int. Ed.* **2023**, *62* (4), e202208936. https://doi.org/10.1002/anie.202208936.
(21) Gao, S.; Das, A.; Alfonzo, E.; Sicinski, K. M.; Rieger, D.; Arnold, F. H. Enzymatic Nitrogen Incorporation Using Hydroxylamine. *J. Am. Chem. Soc.* **2023**, *145* (37), 20196–20201. https://doi.org/10.1021/jacs.3c08053.
(22) Miller, D. C.; Lal, R. G.; Marchetti, L. A.; Arnold, F. H. Biocatalytic One-Carbon Ring Expansion of Aziridines to Azetidines via a Highly Enantioselective [1,2]-Stevens Rearrangement. *J. Am. Chem. Soc.* **2022**, *144* (11), 4739–4745. https://doi.org/10.1021/jacs.2c00251.
(23) Alfonzo, E.; Hanley, D.; Li, Z.-Q.; Sicinski, K. M.; Gao, S.; Arnold, F. H. Biocatalytic Synthesis of α-Amino Esters via Nitrene C–H Insertion. *J. Am. Chem. Soc.* **2024**, *146* (40), 27267–27273. https://doi.org/10.1021/jacs.4c09989.
(24) Huang, X.; Garcia-Borràs, M.; Miao, K.; Kan, S. B. J.; Zutshi, A.; Houk, K. N.; Arnold, F. H. A Biocatalytic Platform for Synthesis of Chiral *α*- Trifluoromethylated Organoborons. *ACS Cent. Sci.* **2019**, *5* (2), 270–276. https://doi.org/10.1021/acscentsci.8b00679.
(25) Kan, S. B. J.; Huang, X.; Gumulya, Y.; Chen, K.; Arnold, F. H. Genetically Programmed Chiral Organoborane Synthesis. *Nature* **2017**, *552* (7683), 132–136. https://doi.org/10.1038/nature24996.
(26) Kan, S. B. J.; Lewis, R. D.; Chen, K.; Arnold, F. H. Directed Evolution of Cytochrome c for Carbon–Silicon Bond Formation: Bringing Silicon to Life. *Science* **2016**, *354* (6315), 1048–1051. https://doi.org/10.1126/science.aah6219.
(27) Liu, Z.; Calvó-Tusell, C.; Zhou, A. Z.; Chen, K.; Garcia-Borràs, M.; Arnold, F. H. Dual-Function Enzyme Catalysis for Enantioselective Carbon–Nitrogen Bond Formation. *Nat. Chem.* **2021**, *13* (12), 1166–1172. https://doi.org/10.1038/s41557-021-00794-z.
(28) Xu, Q.; Zhao, C.-Q.; Han, L.-B. Stereospecific Nucleophilic Substitution of Optically Pure *H* -Phosphinates: A General Way for the Preparation of Chiral P-Stereogenic Phosphine Oxides. *J. Am. Chem. Soc.* **2008**, *130* (38), 12648–12655. https://doi.org/10.1021/ja804412k.
(29) Kiełbasiński, P.; Ź;urawiński, R.; Michał Pietrusiewicz, K.; Zabłocka, M.; Mikołajczyk, M. Enzymatic Resolution of Racemic Phosphinoylacetates Having a Stereogenic Phosphorus Atom. *Tetrahedron Lett.* **1994**, *35* (38), 7081–7084. https://doi.org/10.1016/0040-4039(94)88231-2.

(30) Kiełbasiński, P.; Góralczyk, P.; Mikołajczyk, M.; Wieczorek, M. W.; Majzner, W. R. Enzyme-Promoted Kinetic Resolution of Racemic, P-Chiral Phosphonyl and Phosphorylacetates. *Tetrahedron Asymmetry* **1998**, *9* (15), 2641–2650. https://doi.org/10.1016/S0957-4166(98)00258-4.
